# Supplementary material for: Advanced Glycation End Products of Bovine Serum Albumin Suppressed Th1/Th2 Cytokine but Enhanced Monocyte IL-6 Gene Expression via MAPK-ERK and MyD88 Transduced NF-κB p50 Signaling Pathways
Source: Molecules. 2019 Jul 4;24(13):2461. doi: 10.3390/molecules24132461 (PMC6652144; doi:10.3390/molecules24132461)
Supplement: Supplementary file 1 [file molecules-24-02461-s001.zip › Supplement 2.pdf]

Supplement 2 : Glycation Site Identification Report

Glycation Site Identification Report

Sponsor:

臺大醫院內科部

Document Number: FC19012901R01
Date Issued: Mar. 12, 2019

Pharmaceutical Analysis Center, Mithra Biotechnology Inc.
7F., No. 104, Sec. 1, Xintai 5th Rd., Xizhi Dist., New Taipei City 22102, Taiwan
Tel: 886-2-26962669 Fax: 886-2-26962543

Service Item: LC-MS/MS+Database search

CUSTOMER INFORMATION	
**Contact Name**	沈玠妤
**Document Number**	FC19012901R01
**Institute/Department**	臺大醫院/內科部
**Phone**	886-2-23123456

SAMPLE INFORMATION	
**Sample Name**	1. BSA-1<br>2. BSA-2<br>3. BSA-3
**Sample Received**	Jan. 29, 2019
**Sample Analyzed**	Feb. 14, 2019

ANALYSIS DETAILS	
**Sample Treatment**	Trypsin and chymotrypsin digestion
**Search Engine**	Mascot database (Version: 2.6.0)
**Database**	BSA
**Taxonomy**	N.A
**Instrument**	Thermo Q-Exactive LC-MS
**Software**	Thermo Xcalibur 4.0
**Column**	Thermo C18 column
**Peak List File Name**	1. BSA_1(Trypsin digest): P2019021807.mgf<br>2. BSA_1(Chymotrypsin digest): P2019021808.mgf<br>3. BSA_2(Trypsin digest): P2019021809.mgf<br>4. BSA_2(Chymotrypsin digest): P2019021810.mgf

	5. BSA_3(Trypsin digest): P2019021811.mgf<br>6. BSA_3(Chymotrypsin digest):P2019021812.mgf
**Data Processing Software**	Proteome Discoverer 1.4
**Analyst**	Li Chuan Huang

**DATABASE SEARCH PARAMETERS**	
**Type of Search**	MS/MS Ion Search
**Enzyme**	1. Trypsin<br>2. Chymotrypsin
**Fixed modifications**	Carbamidomethyl (C)
**Variable Modifications**	Glycation (K), Glycation (R), Glycation (C)
**Mass Values**	Monoisotopic
**Protein Mass**	68386
**Peptide Mass Tolerance**	±10 ppm
**Fragment Mass Tolerance**	±0.05 Da
**Max Missed Cleavages**	4
**Instrument Type**	ESI-TRAP
**Number of Queries**	1. BSA_1(Trypsin digest): 6388<br>2. BSA_1(Chymotrypsin digest): 5636<br>3. BSA_2(Trypsin digest): 4463<br>4. BSA_2(Chymotrypsin digest): 3911<br>5. BSA_3(Trypsin digest): 6425<br>6. BSA_3(Chymotrypsin digest): 4810
**Cut-off Score**	13

RESULT
The three samples were desalted with Amicon Ultra Centrifugal Filter (MW cut-off : 10 kDa). Afterwards, the three samples were digested with trypsin or chymotrypsin, followed by LC-MS/MS survey scan. Raw data was processed into peak list by Proteome Discoverer 1.4 for Mascot database search. In total, 43, 56 and 56 glycated sites were identified in BSA-1, BSA-2 and BSA-3, respectively. The results are listed in Tables 1- 2.

NOTE: The report is for research purpose only, not for regulatory submission.

Table 1. Summary for glycation sites analysis

Sample	Trypsin digestion	Chymotrypsin digest
BSA_1	Appendix 1	Appendix 2
BSA_2	Appendix 3	Appendix 4
BSA_3	Appendix 5	Appendix 6

Sample	BSA_1	BSA_2	BSA_3
Total K	59		
N-terminal	1		
Observed K	55	56	56
Glycation site ID	43	56	56

BSA Sequence: Total 583 amino acids containing 59 lysines

```
1    DTHKSEIAHR  FKDLGEEHFK  GLVLIAFSQY  LQQCPFDEHV  KLVNELTEFA
51   KTCVADESHA  GCEKSLHTLF  GDELCKVASL  RETYGDMADC  CEKQEPERNE
101  CFLSHKDDSP  DLPKLKPDPN  TLCDEFKADE  KKFWGKYLYE  IARRHPYFYA
151  PELLYYANKY  NGVFQECCQA  EDKGACLLPK  IETMREKVLA  SSARQRLRCA
201  SIQKFGERAL  KAWSVARLSQ  KFPKAEFVEV  TKLVTDLTKV  HKECCHGDLL
251  ECADDRADLA  KYICDNQDTI  SSKLKECCDK  PLLEKSHCIA  EVEKDAIPEN
301  LPPLTADFAE  DKDVCKNYQE  AKDAFLGSFL  YEYSRRHPEY  AVSVLLRLAK
351  EYEATLEECC  AKDDPHACYS  TVFDKLKHLV  DEPQNLIKQN  CDQFEKLGEY
401  GFQNALIVRY  TRKVPQVSTP  TLVEVSRSLG  KVGTRCCTKP  ESERMPCTED
451  YLSLILNRLC  VLHEKTPVSE  KVTKCCTESL  VNRRPCFSAL  TPDETYVPKA
501  FDEKLFTFHA  DICTLPDTEK  QIKKQTALVE  LLKHKPKATE  EQLKTVMENF
551  VAFVDKCCAA  DDKEACFAVE  GPKLVVSTQT  ALA
```

Table 2. Summary of identified glycation sites for the three samples with different enzymes

K site	BSA_1	BSA_2	BSA_3	K site	BSA_1	BSA_2	BSA_3
N-term	O*	O*	O*	K280	O	O	O
K4	O*	O*	O*	K285		O	O
K12	O	O	O	K294	O	O	O
K20		O*	O*	K312	O	O	O
K41	O*	O*	O*	K316	O	O	O
K51	O	O	O	K322	O	O	O
K64	O	O	O	K350	O	O	O
K76	O	O	O	K362	O	O	O
K93		O	O	K375	O	O	O
K106		O	O	K377	O	O	O
K114	O	O	O	K388	O	O	O
K116	O	O	O	K396		O	O
K127	O	O	O	K413		O	O
K131	O*	O	O	K431			
K132	O	O	O	K439			
K136	O	O	O	K465	O	O	O
K159		O	O	K471		O	O
K173	O	O	O	K474	O	O	O
K180	O	O	O	K499	O	O	O
K187				K504	O	O	O
K204	O	O	O	K520		O*	O
K211	O	O	O	K523	O	O	O
K221	O	O	O	K524	O	O	O
K224		O	O	K533			
K232	O	O	O	K535		O	O
K239	O	O	O	K537		O	O
K242	O	O	O	K544		O	O
K261	O	O	O	K556	O*	O	O
K273	O	O	O	K563	O	O	O
K275	O	O	O	K573	O*	O	O

***:** Glycation sites were identified from chymotrypsin digestion

Table 2. Summary of identified glycation sites for the three samples with different enzymes

R site	BSA_1	BSA_2	BSA_3
R10			
R81			
R98			
R143			
R144			
R185			
R194			
R196			
R198			
R208			
R217			
R256	O*	O*	O*
R335			
R336			O
R347	O*		O*
R409			
R412			O*
R427			
R435			
R444			
R458			
R483			
R484			

***:** Glycation sites were identified from chymotrypsin digestion

Appendix 1. Mascot search results of trypsin digestion for BSA-1

MATRIX SCIENCE MASCOT Search Results

Protein View: >gi|00000

BSA

**Database:**	BSA
**Score:**	66729
**Monoisotopic mass (M_r):**	68386
**Calculated pI:**	5.60

Sequence similarity is available as **an NCBI BLAST search of >gi|00000 against nr**.

Search parameters

**MS data file:**	P2019021807.mgf
**Enzyme:**	Trypsin/P: cuts C-term side of KR.
**Fixed modifications:**	Carbamidomethyl (C)
**Variable modifications:**	Glycation (K), Glycation (Protein N-term)

Protein sequence coverage: 90%

Matched peptides shown in ***bold red***.

```
  1 DTHKSEIAHR FKDLGEEHFK GLVLIAFSQY LQQCPFDEHV KLVNELTEFA
 51 KTCVADESHA GCEKSLHTLF GDELCKVASL RETYGDMADC CEKQEPERNE
101 CFLSHKDDSP DLPKLKPDPN TLCDEFKADE KKFWGKYLYE IARRHPYFYA
151 PELLYYANKY NGVFQECCQA EDKGACLLPK IETMREKVLA SSARQRLRCA
201 SIQKFGERAL KAWSVARLSQ KFPKAEFVEV TKLVTDLTKV HKECCHGDLL
251 ECADDRADLA KYICDNQDTI SSKLKECCDK PLLEKSHCIA EVEKDAIPEN
301 LPPLTADFAE DKDVCKNYQE AKDAFLGSFL YEYSRRHPEY AVSVLLRLAK
351 EYEATLEECC AKDDPHACYS TVFDKLKHLV DEPQNLIKQN CDQFEKLGEY
401 GFQNALIVRY TRKVPQVSTP TLVEVSRSLG KVGTRCCTKP ESERMPCTED
451 YLSLILNRLC VLHEKTPVSE KVTKCCTESL VNRRPCFSAL TPDETYVPKA
501 FDEKLFTFHA DICTLPDTEK QIKKQTALVE LLKHKPKATE EQLKTVMENF
551 VAFVDKCCAA DDKEACFAVE GPKLVVSTQT ALA
```

Unformatted sequence string: **583 residues** (for pasting into other applications).

Sort by ◉ residue number ○ increasing mass ○ decreasing mass
Show ◉ matched peptides only ○ predicted peptides also

Query	Start – End	Observed	Mr(expt)	Mr(calc)	ppm	M	Score	Expect	Rank	U	Peptide
1938	11 – 20	313.1605	1248.6129	1248.6139	-0.82	1	20	0.01	1	U	R.FKDLGEEHFK.G
1939	11 – 20	313.1605	1248.6130	1248.6139	-0.72	1	13	0.049	1	U	R.FKDLGEEHFK.G
1940	11 – 20	313.1605	1248.6130	1248.6139	-0.72	1	20	0.011	1	U	R.FKDLGEEHFK.G
1941	11 – 20	417.2116	1248.6130	1248.6139	-0.70	1	47	2.1e-005	1	U	R.FKDLGEEHFK.G
1942	11 – 20	625.3138	1248.6130	1248.6139	-0.70	1	49	1.2e-005	1	U	R.FKDLGEEHFK.G
1943	11 – 20	417.2116	1248.6131	1248.6139	-0.63	1	40	9.4e-005	1	U	R.FKDLGEEHFK.G
1944	11 – 20	417.2116	1248.6131	1248.6139	-0.63	1	31	0.00074	1	U	R.FKDLGEEHFK.G
1945	11 – 20	313.1606	1248.6131	1248.6139	-0.62	1	13	0.045	1	U	R.FKDLGEEHFK.G
1946	11 – 20	313.1606	1248.6131	1248.6139	-0.62	1	21	0.0073	1	U	R.FKDLGEEHFK.G
1947	11 – 20	625.3138	1248.6131	1248.6139	-0.60	1	40	0.0001	1	U	R.FKDLGEEHFK.G
1948	11 – 20	417.2117	1248.6132	1248.6139	-0.56	1	31	0.0008	1	U	R.FKDLGEEHFK.G
1949	11 – 20	417.2117	1248.6132	1248.6139	-0.56	1	47	2.1e-005	1	U	R.FKDLGEEHFK.G
1950	11 – 20	313.1606	1248.6132	1248.6139	-0.53	1	20	0.0099	1	U	R.FKDLGEEHFK.G
1951	11 – 20	313.1606	1248.6132	1248.6139	-0.53	1	20	0.0095	1	U	R.FKDLGEEHFK.G
1952	11 – 20	625.3139	1248.6132	1248.6139	-0.50	1	49	1.1e-005	1	U	R.FKDLGEEHFK.G
1953	11 – 20	313.1606	1248.6133	1248.6139	-0.43	1	16	0.024	1	U	R.FKDLGEEHFK.G
1954	11 – 20	417.2118	1248.6135	1248.6139	-0.34	1	32	0.0007	1	U	R.FKDLGEEHFK.G
1955	11 – 20	417.2118	1248.6135	1248.6139	-0.34	1	33	0.00053	1	U	R.FKDLGEEHFK.G
1956	11 – 20	313.1606	1248.6135	1248.6139	-0.34	1	19	0.014	1	U	R.FKDLGEEHFK.G
1957	11 – 20	313.1606	1248.6135	1248.6139	-0.34	1	21	0.0079	1	U	R.FKDLGEEHFK.G
1958	11 – 20	625.3140	1248.6135	1248.6139	-0.29	1	49	1.1e-005	1	U	R.FKDLGEEHFK.G
1959	11 – 20	625.3140	1248.6135	1248.6139	-0.29	1	47	2e-005	1	U	R.FKDLGEEHFK.G
1960	11 – 20	417.2118	1248.6135	1248.6139	-0.27	1	47	2.1e-005	1	U	R.FKDLGEEHFK.G
1961	11 – 20	417.2118	1248.6135	1248.6139	-0.27	1	29	0.0014	1	U	R.FKDLGEEHFK.G
1962	11 – 20	313.1607	1248.6136	1248.6139	-0.21	1	17	0.02	1	U	R.FKDLGEEHFK.G
1963	11 – 20	417.2118	1248.6136	1248.6139	-0.20	1	47	2e-005	1	U	R.FKDLGEEHFK.G
1964	11 – 20	417.2118	1248.6136	1248.6139	-0.20	1	44	4.4e-005	1	U	R.FKDLGEEHFK.G
1965	11 – 20	313.1607	1248.6137	1248.6139	-0.11	1	21	0.0081	1	U	R.FKDLGEEHFK.G
1966	11 – 20	313.1607	1248.6137	1248.6139	-0.11	1	18	0.015	1	U	R.FKDLGEEHFK.G
1967	11 – 20	313.1607	1248.6137	1248.6139	-0.11	1	20	0.011	1	U	R.FKDLGEEHFK.G
1968	11 – 20	625.3142	1248.6137	1248.6139	-0.10	1	21	0.0078	1	U	R.FKDLGEEHFK.G
1969	11 – 20	625.3142	1248.6137	1248.6139	-0.10	1	49	1.1e-005	1	U	R.FKDLGEEHFK.G
1970	11 – 20	625.3142	1248.6137	1248.6139	-0.10	1	48	1.5e-005	1	U	R.FKDLGEEHFK.G
1971	11 – 20	625.3142	1248.6137	1248.6139	-0.10	1	56	2.7e-006	1	U	R.FKDLGEEHFK.G
1972	11 – 20	625.3142	1248.6137	1248.6139	-0.10	1	49	1.1e-005	1	U	R.FKDLGEEHFK.G
1973	11 – 20	417.2119	1248.6138	1248.6139	-0.051	1	43	4.6e-005	1	U	R.FKDLGEEHFK.G
1974	11 – 20	417.2119	1248.6138	1248.6139	-0.051	1	43	4.5e-005	1	U	R.FKDLGEEHFK.G
1975	11 – 20	417.2119	1248.6138	1248.6139	-0.051	1	47	1.9e-005	1	U	R.FKDLGEEHFK.G
1976	11 – 20	313.1607	1248.6139	1248.6139	-0.016	1	17	0.02	1	U	R.FKDLGEEHFK.G
1977	11 – 20	625.3142	1248.6139	1248.6139	-0.0064	1	48	1.6e-005	1	U	R.FKDLGEEHFK.G
1978	11 – 20	417.2119	1248.6139	1248.6139	0.021	1	47	2.1e-005	1	U	R.FKDLGEEHFK.G
1979	11 – 20	313.1608	1248.6140	1248.6139	0.080	1	18	0.017	1	U	R.FKDLGEEHFK.G
1980	11 – 20	417.2119	1248.6140	1248.6139	0.093	1	47	2e-005	1	U	R.FKDLGEEHFK.G
1981	11 – 20	313.1608	1248.6141	1248.6139	0.18	1	26	0.0027	1	U	R.FKDLGEEHFK.G

Query	Start - End	Observed	Mr(expt)	Mr(calc)	ppm	M	Score	Expect	Rank	U	Peptide
1982	11 - 20	625.3143	1248.6141	1248.6139	0.19	1	49	1.2e-005	1	U	R.FKDLGEEHFK.G
1983	11 - 20	625.3143	1248.6141	1248.6139	0.19	1	50	1.1e-005	1	U	R.FKDLGEEHFK.G
1984	11 - 20	625.3143	1248.6141	1248.6139	0.19	1	49	1.1e-005	1	U	R.FKDLGEEHFK.G
1985	11 - 20	417.2120	1248.6142	1248.6139	0.26	1	47	2e-005	1	U	R.FKDLGEEHFK.G
1986	11 - 20	417.2120	1248.6143	1248.6139	0.33	1	35	0.00029	1	U	R.FKDLGEEHFK.G
1987	11 - 20	417.2121	1248.6144	1248.6139	0.41	1	47	1.9e-005	1	U	R.FKDLGEEHFK.G
1988	11 - 20	625.3145	1248.6145	1248.6139	0.47	1	37	0.00022	1	U	R.FKDLGEEHFK.G
1989	11 - 20	417.2121	1248.6145	1248.6139	0.48	1	47	2e-005	1	U	R.FKDLGEEHFK.G
1990	11 - 20	417.2122	1248.6147	1248.6139	0.69	1	32	0.00057	1	U	R.FKDLGEEHFK.G
1991	11 - 20	417.2124	1248.6153	1248.6139	1.13	1	47	2e-005	1	U	R.FKDLGEEHFK.G
1992	11 - 20	313.1611	1248.6153	1248.6139	1.14	1	18	0.015	1	U	R.FKDLGEEHFK.G
1993	11 - 20	625.3149	1248.6153	1248.6139	1.16	1	49	1.1e-005	1	U	R.FKDLGEEHFK.G
1994	11 - 20	417.2125	1248.6156	1248.6139	1.34	1	30	0.001	1	U	R.FKDLGEEHFK.G
1995	11 - 20	625.3153	1248.6161	1248.6139	1.76	1	30	0.0011	1	U	R.FKDLGEEHFK.G
1996	11 - 20	625.3153	1248.6161	1248.6139	1.76	1	19	0.014	1	U	R.FKDLGEEHFK.G
1997	11 - 20	625.3161	1248.6176	1248.6139	3.02	1	18	0.014	1	U	R.FKDLGEEHFK.G
2577	11 - 20	706.3402	1410.6659	1410.6667	-0.59	1	36	0.00027	1	U	R.FKDLGEEHFK.G + Glycation (K)
2578	11 - 20	706.3402	1410.6659	1410.6667	-0.59	1	33	0.00055	1	U	R.FKDLGEEHFK.G + Glycation (K)
2579	11 - 20	706.3403	1410.6660	1410.6667	-0.51	1	30	0.0011	1	U	R.FKDLGEEHFK.G + Glycation (K)
2580	11 - 20	706.3404	1410.6662	1410.6667	-0.34	1	40	0.00011	1	U	R.FKDLGEEHFK.G + Glycation (K)
2581	11 - 20	353.6739	1410.6665	1410.6667	-0.17	1	31	0.00081	1	U	R.FKDLGEEHFK.G + Glycation (K)
2582	11 - 20	353.6739	1410.6666	1410.6667	-0.089	1	34	0.00036	1	U	R.FKDLGEEHFK.G + Glycation (K)
2583	11 - 20	353.6739	1410.6666	1410.6667	-0.089	1	42	6.3e-005	1	U	R.FKDLGEEHFK.G + Glycation (K)
2584	11 - 20	353.6739	1410.6667	1410.6667	-0.0043	1	35	0.00034	1	U	R.FKDLGEEHFK.G + Glycation (K)
2585	11 - 20	471.2296	1410.6668	1410.6667	0.085	1	35	0.00029	1	U	R.FKDLGEEHFK.G + Glycation (K)
2586	11 - 20	706.3407	1410.6668	1410.6667	0.10	1	43	4.6e-005	1	U	R.FKDLGEEHFK.G + Glycation (K)
2587	11 - 20	471.2296	1410.6671	1410.6667	0.28	1	51	8.6e-006	1	U	R.FKDLGEEHFK.G + Glycation (K)
2588	11 - 20	471.2296	1410.6671	1410.6667	0.28	1	28	0.0015	1	U	R.FKDLGEEHFK.G + Glycation (K)
2589	11 - 20	471.2296	1410.6671	1410.6667	0.28	1	47	2.2e-005	1	U	R.FKDLGEEHFK.G + Glycation (K)
2590	11 - 20	471.2297	1410.6674	1410.6667	0.49	1	32	0.00061	1	U	R.FKDLGEEHFK.G + Glycation (K)
927	13 - 20	325.4906	973.4499	973.4505	-0.63	0	24	0.0037	1	U	K.DLGEEHFK.G
929	13 - 20	487.7324	973.4502	973.4505	-0.28	0	21	0.0087	1	U	K.DLGEEHFK.G
930	13 - 20	487.7324	973.4503	973.4505	-0.22	0	22	0.0066	1	U	K.DLGEEHFK.G
931	13 - 20	325.4907	973.4503	973.4505	-0.17	0	34	0.00041	1	U	K.DLGEEHFK.G
932	13 - 20	325.4907	973.4503	973.4505	-0.17	0	24	0.0037	1	U	K.DLGEEHFK.G
934	13 - 20	487.7324	973.4503	973.4505	-0.16	0	22	0.0057	1	U	K.DLGEEHFK.G
935	13 - 20	487.7324	973.4503	973.4505	-0.16	0	22	0.0064	1	U	K.DLGEEHFK.G
936	13 - 20	487.7325	973.4504	973.4505	-0.095	0	20	0.009	1	U	K.DLGEEHFK.G
937	13 - 20	487.7325	973.4504	973.4505	-0.095	0	24	0.0042	1	U	K.DLGEEHFK.G
939	13 - 20	325.4907	973.4504	973.4505	-0.080	0	24	0.0036	1	U	K.DLGEEHFK.G
940	13 - 20	487.7325	973.4505	973.4505	-0.033	0	21	0.0088	1	U	K.DLGEEHFK.G
941	13 - 20	325.4908	973.4505	973.4505	0.012	0	26	0.0027	1	U	K.DLGEEHFK.G
942	13 - 20	487.7326	973.4506	973.4505	0.090	0	20	0.011	1	U	K.DLGEEHFK.G
943	13 - 20	487.7326	973.4506	973.4505	0.090	0	21	0.0086	1	U	K.DLGEEHFK.G
944	13 - 20	325.4908	973.4506	973.4505	0.10	0	26	0.0027	1	U	K.DLGEEHFK.G
946	13 - 20	487.7326	973.4507	973.4505	0.23	0	24	0.004	1	U	K.DLGEEHFK.G
947	13 - 20	487.7326	973.4507	973.4505	0.23	0	23	0.0045	1	U	K.DLGEEHFK.G
948	13 - 20	487.7327	973.4508	973.4505	0.30	0	22	0.0061	1	U	K.DLGEEHFK.G
950	13 - 20	487.7327	973.4508	973.4505	0.36	0	24	0.0041	1	U	K.DLGEEHFK.G
952	13 - 20	487.7328	973.4510	973.4505	0.48	0	15	0.029	1	U	K.DLGEEHFK.G
953	13 - 20	487.7328	973.4510	973.4505	0.48	0	22	0.0059	1	U	K.DLGEEHFK.G
5563	21 - 41	623.8201	2491.2512	2491.2570	-2.33	0	43	5.5e-005	1	U	K.GLVLIAFSQYLQQCPFDEHVK.L
5564	21 - 41	831.4252	2491.2539	2491.2570	-1.25	0	75	2.9e-008	1	U	K.GLVLIAFSQYLQQCPFDEHVK.L
5565	21 - 41	831.4253	2491.2540	2491.2570	-1.18	0	73	4.6e-008	1	U	K.GLVLIAFSQYLQQCPFDEHVK.L
5566	21 - 41	623.8208	2491.2541	2491.2570	-1.15	0	40	9.9e-005	1	U	K.GLVLIAFSQYLQQCPFDEHVK.L
5567	21 - 41	623.8209	2491.2543	2491.2570	-1.06	0	37	0.00018	1	U	K.GLVLIAFSQYLQQCPFDEHVK.L
5568	21 - 41	831.4255	2491.2546	2491.2570	-0.95	0	68	1.6e-007	1	U	K.GLVLIAFSQYLQQCPFDEHVK.L
5569	21 - 41	831.4255	2491.2548	2491.2570	-0.88	0	69	1.1e-007	1	U	K.GLVLIAFSQYLQQCPFDEHVK.L
5570	21 - 41	831.4256	2491.2550	2491.2570	-0.80	0	67	2.2e-007	1	U	K.GLVLIAFSQYLQQCPFDEHVK.L
5571	21 - 41	623.8216	2491.2573	2491.2570	0.11	0	43	4.8e-005	1	U	K.GLVLIAFSQYLQQCPFDEHVK.L
1677	42 - 51	582.3186	1162.6226	1162.6234	-0.62	0	57	2e-006	1	U	K.LVNELTEFAK.T
1678	42 - 51	582.3187	1162.6229	1162.6234	-0.39	0	62	6.6e-007	1	U	K.LVNELTEFAK.T
1679	42 - 51	582.3188	1162.6230	1162.6234	-0.29	0	57	2.1e-006	1	U	K.LVNELTEFAK.T
1680	42 - 51	582.3188	1162.6230	1162.6234	-0.29	0	62	6.7e-007	1	U	K.LVNELTEFAK.T
1681	42 - 51	582.3189	1162.6231	1162.6234	-0.19	0	57	2.1e-006	1	U	K.LVNELTEFAK.T
1682	42 - 51	582.3189	1162.6231	1162.6234	-0.19	0	39	0.00013	1	U	K.LVNELTEFAK.T
1683	42 - 51	582.3189	1162.6231	1162.6234	-0.19	0	58	1.7e-006	1	U	K.LVNELTEFAK.T
1684	42 - 51	582.3189	1162.6231	1162.6234	-0.19	0	58	1.7e-006	1	U	K.LVNELTEFAK.T
1685	42 - 51	582.3189	1162.6233	1162.6234	-0.085	0	44	4.3e-005	1	U	K.LVNELTEFAK.T
1686	42 - 51	582.3189	1162.6233	1162.6234	-0.085	0	62	6.9e-007	1	U	K.LVNELTEFAK.T
1687	42 - 51	582.3189	1162.6233	1162.6234	-0.085	0	58	1.8e-006	1	U	K.LVNELTEFAK.T
1688	42 - 51	582.3189	1162.6233	1162.6234	-0.085	0	57	1.8e-006	1	U	K.LVNELTEFAK.T
1689	42 - 51	582.3189	1162.6233	1162.6234	-0.085	0	58	1.8e-006	1	U	K.LVNELTEFAK.T
1690	42 - 51	582.3190	1162.6234	1162.6234	0.018	0	41	8.6e-005	1	U	K.LVNELTEFAK.T
1691	42 - 51	582.3190	1162.6234	1162.6234	0.018	0	62	7e-007	1	U	K.LVNELTEFAK.T
1692	42 - 51	582.3190	1162.6234	1162.6234	0.018	0	62	6.8e-007	1	U	K.LVNELTEFAK.T
1693	42 - 51	582.3190	1162.6234	1162.6234	0.018	0	62	6.8e-007	1	U	K.LVNELTEFAK.T
1694	42 - 51	582.3190	1162.6234	1162.6234	0.018	0	58	1.7e-006	1	U	K.LVNELTEFAK.T
1695	42 - 51	582.3190	1162.6235	1162.6234	0.12	0	57	1.9e-006	1	U	K.LVNELTEFAK.T
1696	42 - 51	582.3190	1162.6235	1162.6234	0.12	0	62	6.9e-007	1	U	K.LVNELTEFAK.T
1697	42 - 51	582.3190	1162.6235	1162.6234	0.12	0	62	6.7e-007	1	U	K.LVNELTEFAK.T
1698	42 - 51	582.3190	1162.6235	1162.6234	0.12	0	58	1.7e-006	1	U	K.LVNELTEFAK.T
1699	42 - 51	582.3191	1162.6236	1162.6234	0.22	0	45	3.3e-005	1	U	K.LVNELTEFAK.T
1700	42 - 51	582.3191	1162.6236	1162.6234	0.22	0	61	7.1e-007	1	U	K.LVNELTEFAK.T
1701	42 - 51	582.3192	1162.6237	1162.6234	0.33	0	62	7e-007	1	U	K.LVNELTEFAK.T
1702	42 - 51	582.3192	1162.6237	1162.6234	0.33	0	45	3.5e-005	1	U	K.LVNELTEFAK.T
1703	42 - 51	582.3192	1162.6237	1162.6234	0.33	0	62	6.7e-007	1	U	K.LVNELTEFAK.T
1704	42 - 51	582.3193	1162.6240	1162.6234	0.53	0	45	3.4e-005	1	U	K.LVNELTEFAK.T
1705	42 - 51	582.3193	1162.6240	1162.6234	0.53	0	57	1.9e-006	1	U	K.LVNELTEFAK.T
1706	42 - 51	582.3193	1162.6241	1162.6234	0.65	0	57	1.9e-006	1	U	K.LVNELTEFAK.T

Query	Start – End	Observed	Mr(expt)	Mr(calc)	ppm	M	Score	Expect	Rank	U	Peptide
1707	42 – 51	582.3194	1162.6242	1162.6234	0.76	0	61	7.2e-007	1	U	K.LVNELTEFAK.T
1708	42 – 51	582.3195	1162.6245	1162.6234	0.96	0	57	2.2e-006	1	U	K.LVNELTEFAK.T
1709	42 – 51	582.3196	1162.6246	1162.6234	1.07	0	58	1.7e-006	1	U	K.LVNELTEFAK.T
1710	42 – 51	582.3199	1162.6252	1162.6234	1.60	0	62	7e-007	1	U	K.LVNELTEFAK.T
1711	42 – 51	582.3199	1162.6253	1162.6234	1.70	0	62	6.8e-007	1	U	K.LVNELTEFAK.T
1712	42 – 51	582.3203	1162.6261	1162.6234	2.32	0	62	6.9e-007	1	U	K.LVNELTEFAK.T
5758	42 – 64	924.0888	2769.2446	2769.2473	-0.98	1	112	5.7e-012	1	U	K.LVNELTEFAKTCVADESHAGCEK.S + Glycatio
5759	42 – 64	693.3185	2769.2448	2769.2473	-0.90	1	88	1.6e-009	1	U	K.LVNELTEFAKTCVADESHAGCEK.S + Glycatio
5760	42 – 64	693.3185	2769.2451	2769.2473	-0.82	1	88	1.6e-009	1	U	K.LVNELTEFAKTCVADESHAGCEK.S + Glycatio
5761	42 – 64	924.0890	2769.2451	2769.2473	-0.78	1	96	2.6e-010	1	U	K.LVNELTEFAKTCVADESHAGCEK.S + Glycatio
5762	42 – 64	924.0892	2769.2459	2769.2473	-0.52	1	112	5.7e-012	1	U	K.LVNELTEFAKTCVADESHAGCEK.S + Glycatio
5763	42 – 64	693.3188	2769.2461	2769.2473	-0.45	1	88	1.6e-009	1	U	K.LVNELTEFAKTCVADESHAGCEK.S + Glycatio
5764	42 – 64	693.3189	2769.2465	2769.2473	-0.28	1	76	2.3e-008	1	U	K.LVNELTEFAKTCVADESHAGCEK.S + Glycatio
5765	42 – 64	693.3190	2769.2470	2769.2473	-0.11	1	68	1.4e-007	1	U	K.LVNELTEFAKTCVADESHAGCEK.S + Glycatio
2884	52 – 64	732.2969	1462.5793	1462.5817	-1.61	0	77	2e-008	1	U	K.TCVADESHAGCEK.S
2885	52 – 64	732.2973	1462.5800	1462.5817	-1.12	0	91	8.2e-010	1	U	K.TCVADESHAGCEK.S
2886	52 – 64	732.2974	1462.5802	1462.5817	-1.03	0	77	2.1e-008	1	U	K.TCVADESHAGCEK.S
2887	52 – 64	732.2975	1462.5804	1462.5817	-0.86	0	91	8.7e-010	1	U	K.TCVADESHAGCEK.S
2888	52 – 64	732.2975	1462.5804	1462.5817	-0.86	0	91	8.4e-010	1	U	K.TCVADESHAGCEK.S
2889	52 – 64	488.5341	1462.5804	1462.5817	-0.85	0	63	5.1e-007	1	U	K.TCVADESHAGCEK.S
2890	52 – 64	488.5341	1462.5805	1462.5817	-0.78	0	63	5.3e-007	1	U	K.TCVADESHAGCEK.S
2891	52 – 64	732.2976	1462.5805	1462.5817	-0.77	0	91	8e-010	1	U	K.TCVADESHAGCEK.S
2892	52 – 64	732.2976	1462.5805	1462.5817	-0.77	0	91	8.1e-010	1	U	K.TCVADESHAGCEK.S
2893	52 – 64	732.2976	1462.5807	1462.5817	-0.69	0	78	1.8e-008	1	U	K.TCVADESHAGCEK.S
2894	52 – 64	732.2976	1462.5807	1462.5817	-0.69	0	91	8.3e-010	1	U	K.TCVADESHAGCEK.S
2895	52 – 64	732.2977	1462.5808	1462.5817	-0.61	0	91	8.2e-010	1	U	K.TCVADESHAGCEK.S
2896	52 – 64	732.2977	1462.5808	1462.5817	-0.61	0	91	8.4e-010	1	U	K.TCVADESHAGCEK.S
2897	52 – 64	488.5342	1462.5808	1462.5817	-0.60	0	40	9.6e-005	1	U	K.TCVADESHAGCEK.S
2898	52 – 64	488.5342	1462.5809	1462.5817	-0.54	0	63	5e-007	1	U	K.TCVADESHAGCEK.S
2899	52 – 64	488.5342	1462.5809	1462.5817	-0.54	0	63	5e-007	1	U	K.TCVADESHAGCEK.S
2900	52 – 64	732.2977	1462.5809	1462.5817	-0.53	0	91	7.9e-010	1	U	K.TCVADESHAGCEK.S
2901	52 – 64	732.2977	1462.5809	1462.5817	-0.53	0	91	8e-010	1	U	K.TCVADESHAGCEK.S
2902	52 – 64	732.2977	1462.5809	1462.5817	-0.53	0	91	8.3e-010	1	U	K.TCVADESHAGCEK.S
2903	52 – 64	732.2977	1462.5809	1462.5817	-0.53	0	91	8.2e-010	1	U	K.TCVADESHAGCEK.S
2904	52 – 64	732.2978	1462.5810	1462.5817	-0.45	0	91	8.1e-010	1	U	K.TCVADESHAGCEK.S
2905	52 – 64	488.5343	1462.5811	1462.5817	-0.42	0	63	4.7e-007	1	U	K.TCVADESHAGCEK.S
2906	52 – 64	488.5343	1462.5812	1462.5817	-0.35	0	58	1.5e-006	1	U	K.TCVADESHAGCEK.S
2907	52 – 64	488.5344	1462.5813	1462.5817	-0.29	0	79	1.3e-008	1	U	K.TCVADESHAGCEK.S
2908	52 – 64	488.5344	1462.5813	1462.5817	-0.23	0	63	5e-007	1	U	K.TCVADESHAGCEK.S
2909	52 – 64	488.5344	1462.5813	1462.5817	-0.23	0	76	2.5e-008	1	U	K.TCVADESHAGCEK.S
2910	52 – 64	488.5344	1462.5813	1462.5817	-0.23	0	76	2.4e-008	1	U	K.TCVADESHAGCEK.S
2911	52 – 64	732.2980	1462.5814	1462.5817	-0.20	0	77	2.1e-008	1	U	K.TCVADESHAGCEK.S
2912	52 – 64	732.2980	1462.5814	1462.5817	-0.20	0	73	5.3e-008	1	U	K.TCVADESHAGCEK.S
2913	52 – 64	488.5344	1462.5814	1462.5817	-0.17	0	72	5.8e-008	1	U	K.TCVADESHAGCEK.S
2914	52 – 64	488.5344	1462.5815	1462.5817	-0.11	0	63	5e-007	1	U	K.TCVADESHAGCEK.S
2915	52 – 64	488.5344	1462.5815	1462.5817	-0.11	0	59	1.4e-006	1	U	K.TCVADESHAGCEK.S
2916	52 – 64	488.5344	1462.5815	1462.5817	-0.11	0	76	2.4e-008	1	U	K.TCVADESHAGCEK.S
2917	52 – 64	488.5344	1462.5815	1462.5817	-0.11	0	76	2.5e-008	1	U	K.TCVADESHAGCEK.S
2918	52 – 64	488.5345	1462.5816	1462.5817	-0.046	0	76	2.4e-008	1	U	K.TCVADESHAGCEK.S
2919	52 – 64	488.5345	1462.5816	1462.5817	-0.046	0	76	2.4e-008	1	U	K.TCVADESHAGCEK.S
2920	52 – 64	488.5345	1462.5817	1462.5817	0.036	0	39	0.00011	1	U	K.TCVADESHAGCEK.S
2921	52 – 64	488.5346	1462.5818	1462.5817	0.097	0	64	3.6e-007	1	U	K.TCVADESHAGCEK.S
2922	52 – 64	488.5346	1462.5818	1462.5817	0.097	0	76	2.4e-008	1	U	K.TCVADESHAGCEK.S
2923	52 – 64	732.2982	1462.5819	1462.5817	0.14	0	72	6.2e-008	1	U	K.TCVADESHAGCEK.S
2924	52 – 64	488.5346	1462.5819	1462.5817	0.16	0	75	3.5e-008	1	U	K.TCVADESHAGCEK.S
2925	52 – 64	488.5347	1462.5822	1462.5817	0.34	0	76	2.5e-008	1	U	K.TCVADESHAGCEK.S
2926	52 – 64	488.5348	1462.5825	1462.5817	0.59	0	76	2.5e-008	1	U	K.TCVADESHAGCEK.S
2927	52 – 64	488.5351	1462.5835	1462.5817	1.23	0	63	4.8e-007	1	U	K.TCVADESHAGCEK.S
5852	52 – 76	606.0684	3025.3054	3025.3103	-1.63	1	44	4.4e-005	1	U	K.TCVADESHAGCEKSLHTLFGDELCK.V + Glycat
5853	52 – 76	757.3339	3025.3066	3025.3103	-1.25	1	59	1.1e-006	1	U	K.TCVADESHAGCEKSLHTLFGDELCK.V + Glycat
5854	52 – 76	1009.4432	3025.3077	3025.3103	-0.87	1	76	2.6e-008	1	U	K.TCVADESHAGCEKSLHTLFGDELCK.V + Glycat
5855	52 – 76	1009.4434	3025.3083	3025.3103	-0.69	1	81	8.2e-009	1	U	K.TCVADESHAGCEKSLHTLFGDELCK.V + Glycat
5856	52 – 76	757.3343	3025.3083	3025.3103	-0.68	1	35	0.00034	1	U	K.TCVADESHAGCEKSLHTLFGDELCK.V + Glycat
5857	52 – 76	1009.4435	3025.3086	3025.3103	-0.57	1	67	2.1e-007	1	U	K.TCVADESHAGCEKSLHTLFGDELCK.V + Glycat
5858	52 – 76	606.0690	3025.3088	3025.3103	-0.52	1	45	3e-005	1	U	K.TCVADESHAGCEKSLHTLFGDELCK.V + Glycat
5860	52 – 76	606.0691	3025.3091	3025.3103	-0.42	1	49	1.2e-005	1	U	K.TCVADESHAGCEKSLHTLFGDELCK.V + Glycat
5861	52 – 76	757.3347	3025.3095	3025.3103	-0.27	1	53	4.7e-006	1	U	K.TCVADESHAGCEKSLHTLFGDELCK.V + Glycat
5862	52 – 76	606.0692	3025.3097	3025.3103	-0.22	1	49	1.2e-005	1	U	K.TCVADESHAGCEKSLHTLFGDELCK.V + Glycat
5863	52 – 76	606.0692	3025.3097	3025.3103	-0.22	1	49	1.2e-005	1	U	K.TCVADESHAGCEKSLHTLFGDELCK.V + Glycat
5864	52 – 76	606.0693	3025.3103	3025.3103	-0.0096	1	43	4.8e-005	1	U	K.TCVADESHAGCEKSLHTLFGDELCK.V + Glycat
5865	52 – 76	1009.4441	3025.3104	3025.3103	0.031	1	78	1.4e-008	1	U	K.TCVADESHAGCEKSLHTLFGDELCK.V + Glycat
5866	52 – 76	757.3352	3025.3117	3025.3103	0.46	1	50	9.2e-006	1	U	K.TCVADESHAGCEKSLHTLFGDELCK.V + Glycat
2612	65 – 76	710.3496	1418.6845	1418.6864	-1.31	0	62	7e-007	1	U	K.SLHTLFGDELCK.V
2613	65 – 76	710.3498	1418.6851	1418.6864	-0.88	0	66	2.6e-007	1	U	K.SLHTLFGDELCK.V
2614	65 – 76	710.3499	1418.6853	1418.6864	-0.80	0	66	2.6e-007	1	U	K.SLHTLFGDELCK.V
2615	65 – 76	710.3499	1418.6853	1418.6864	-0.80	0	66	2.7e-007	1	U	K.SLHTLFGDELCK.V
2616	65 – 76	710.3500	1418.6854	1418.6864	-0.70	0	64	4.3e-007	1	U	K.SLHTLFGDELCK.V
2617	65 – 76	710.3500	1418.6854	1418.6864	-0.70	0	64	3.7e-007	1	U	K.SLHTLFGDELCK.V
2618	65 – 76	710.3500	1418.6855	1418.6864	-0.62	0	68	1.7e-007	1	U	K.SLHTLFGDELCK.V
2619	65 – 76	710.3501	1418.6856	1418.6864	-0.53	0	66	2.5e-007	1	U	K.SLHTLFGDELCK.V
2620	65 – 76	710.3501	1418.6856	1418.6864	-0.53	0	66	2.3e-007	1	U	K.SLHTLFGDELCK.V
2621	65 – 76	710.3502	1418.6858	1418.6864	-0.45	0	64	4.4e-007	1	U	K.SLHTLFGDELCK.V
2622	65 – 76	710.3502	1418.6858	1418.6864	-0.45	0	66	2.5e-007	1	U	K.SLHTLFGDELCK.V
2623	65 – 76	710.3502	1418.6858	1418.6864	-0.45	0	65	3.3e-007	1	U	K.SLHTLFGDELCK.V
2624	65 – 76	473.9026	1418.6859	1418.6864	-0.39	0	44	3.5e-005	1	U	K.SLHTLFGDELCK.V
2625	65 – 76	710.3502	1418.6859	1418.6864	-0.36	0	64	3.7e-007	1	U	K.SLHTLFGDELCK.V
2626	65 – 76	710.3502	1418.6859	1418.6864	-0.36	0	63	5.2e-007	1	U	K.SLHTLFGDELCK.V
2627	65 – 76	710.3503	1418.6860	1418.6864	-0.28	0	64	3.7e-007	1	U	K.SLHTLFGDELCK.V
2628	65 – 76	473.9026	1418.6860	1418.6864	-0.26	0	59	1.4e-006	1	U	K.SLHTLFGDELCK.V

Query	Start – End	Observed	Mr(expt)	Mr(calc)	ppm	M	Score	Expect	Rank	U	Peptide
2629	65 – 76	473.9027	1418.6862	1418.6864	-0.13	0	36	0.00026	1	U	K.SLHTLFGDELCK.V
2630	65 – 76	473.9027	1418.6862	1418.6864	-0.13	0	46	2.7e-005	1	U	K.SLHTLFGDELCK.V
2631	65 – 76	710.3504	1418.6862	1418.6864	-0.11	0	64	3.9e-007	1	U	K.SLHTLFGDELCK.V
2632	65 – 76	710.3504	1418.6862	1418.6864	-0.11	0	62	6.2e-007	1	U	K.SLHTLFGDELCK.V
2633	65 – 76	473.9027	1418.6863	1418.6864	-0.070	0	42	6.2e-005	1	U	K.SLHTLFGDELCK.V
2634	65 – 76	473.9027	1418.6864	1418.6864	-0.0063	0	44	3.8e-005	1	U	K.SLHTLFGDELCK.V
2635	65 – 76	473.9028	1418.6865	1418.6864	0.057	0	33	0.00049	1	U	K.SLHTLFGDELCK.V
2636	65 – 76	473.9028	1418.6865	1418.6864	0.057	0	40	0.0001	1	U	K.SLHTLFGDELCK.V
2637	65 – 76	473.9028	1418.6865	1418.6864	0.057	0	24	0.0038	1	U	K.SLHTLFGDELCK.V
2638	65 – 76	710.3505	1418.6865	1418.6864	0.061	0	64	4.4e-007	1	U	K.SLHTLFGDELCK.V
2639	65 – 76	473.9028	1418.6866	1418.6864	0.12	0	29	0.0013	1	U	K.SLHTLFGDELCK.V
2640	65 – 76	473.9028	1418.6866	1418.6864	0.12	0	51	7.5e-006	1	U	K.SLHTLFGDELCK.V
2641	65 – 76	473.9028	1418.6866	1418.6864	0.12	0	50	9.8e-006	1	U	K.SLHTLFGDELCK.V
2642	65 – 76	473.9028	1418.6866	1418.6864	0.12	0	55	3e-006	1	U	K.SLHTLFGDELCK.V
2643	65 – 76	473.9028	1418.6867	1418.6864	0.18	0	45	3.5e-005	1	U	K.SLHTLFGDELCK.V
2644	65 – 76	473.9028	1418.6867	1418.6864	0.18	0	27	0.0019	1	U	K.SLHTLFGDELCK.V
2645	65 – 76	473.9028	1418.6867	1418.6864	0.18	0	55	3e-006	1	U	K.SLHTLFGDELCK.V
2646	65 – 76	473.9029	1418.6868	1418.6864	0.25	0	42	6.2e-005	1	U	K.SLHTLFGDELCK.V
2647	65 – 76	473.9029	1418.6868	1418.6864	0.25	0	47	1.9e-005	1	U	K.SLHTLFGDELCK.V
2648	65 – 76	473.9029	1418.6868	1418.6864	0.31	0	48	1.5e-005	1	U	K.SLHTLFGDELCK.V
2649	65 – 76	473.9029	1418.6869	1418.6864	0.37	0	37	0.00021	1	U	K.SLHTLFGDELCK.V
2650	65 – 76	473.9029	1418.6869	1418.6864	0.37	0	49	1.4e-005	1	U	K.SLHTLFGDELCK.V
2651	65 – 76	473.9029	1418.6869	1418.6864	0.37	0	46	2.3e-005	1	U	K.SLHTLFGDELCK.V
2652	65 – 76	473.9029	1418.6870	1418.6864	0.44	0	49	1.3e-005	1	U	K.SLHTLFGDELCK.V
2653	65 – 76	710.3509	1418.6872	1418.6864	0.58	0	55	3.2e-006	1	U	K.SLHTLFGDELCK.V
2654	65 – 76	473.9032	1418.6877	1418.6864	0.90	0	49	1.3e-005	1	U	K.SLHTLFGDELCK.V
2655	65 – 76	473.9035	1418.6888	1418.6864	1.66	0	49	1.2e-005	1	U	K.SLHTLFGDELCK.V
2656	65 – 76	473.9040	1418.6901	1418.6864	2.57	0	46	2.5e-005	1	U	K.SLHTLFGDELCK.V
2657	65 – 76	473.9041	1418.6905	1418.6864	2.89	0	47	1.8e-005	1	U	K.SLHTLFGDELCK.V
5355	65 – 81	527.7730	2107.0627	2107.0620	0.35	1	26	0.0022	1	U	K.SLHTLFGDELCKVASLR.E + Glycation (K)
5356	65 – 81	527.7730	2107.0627	2107.0620	0.35	1	40	0.0001	1	U	K.SLHTLFGDELCKVASLR.E + Glycation (K)
5357	65 – 81	527.7730	2107.0629	2107.0620	0.47	1	37	0.00021	1	U	K.SLHTLFGDELCKVASLR.E + Glycation (K)
5358	65 – 81	527.7730	2107.0629	2107.0620	0.47	1	37	0.00021	1	U	K.SLHTLFGDELCKVASLR.E + Glycation (K)
5359	65 – 81	527.7731	2107.0634	2107.0620	0.70	1	32	0.00066	1	U	K.SLHTLFGDELCKVASLR.E + Glycation (K)
5360	65 – 81	703.3626	2107.0660	2107.0620	1.92	1	38	0.00016	1	U	K.SLHTLFGDELCKVASLR.E + Glycation (K)
5361	65 – 81	703.3629	2107.0669	2107.0620	2.36	1	52	6.6e-006	1	U	K.SLHTLFGDELCKVASLR.E + Glycation (K)
5362	65 – 81	703.3633	2107.0680	2107.0620	2.88	1	56	2.5e-006	1	U	K.SLHTLFGDELCKVASLR.E + Glycation (K)
3029	82 – 93	739.7641	1477.5136	1477.5160	-1.57	0	63	5.3e-007	1	U	R.ETYGDMADCCEK.Q
3030	82 – 93	739.7646	1477.5147	1477.5160	-0.82	0	80	1.1e-008	1	U	R.ETYGDMADCCEK.Q
3031	82 – 93	739.7647	1477.5149	1477.5160	-0.74	0	79	1.1e-008	1	U	R.ETYGDMADCCEK.Q
3032	82 – 93	739.7647	1477.5149	1477.5160	-0.74	0	80	1.1e-008	1	U	R.ETYGDMADCCEK.Q
3033	82 – 93	739.7648	1477.5150	1477.5160	-0.66	0	66	2.4e-007	1	U	R.ETYGDMADCCEK.Q
3034	82 – 93	739.7649	1477.5152	1477.5160	-0.50	0	66	2.5e-007	1	U	R.ETYGDMADCCEK.Q
3035	82 – 93	739.7649	1477.5152	1477.5160	-0.50	0	80	1.1e-008	1	U	R.ETYGDMADCCEK.Q
3036	82 – 93	739.7650	1477.5155	1477.5160	-0.33	0	51	7.9e-006	1	U	R.ETYGDMADCCEK.Q
3037	82 – 93	739.7650	1477.5155	1477.5160	-0.33	0	66	2.3e-007	1	U	R.ETYGDMADCCEK.Q
3038	82 – 93	739.7652	1477.5158	1477.5160	-0.076	0	66	2.3e-007	1	U	R.ETYGDMADCCEK.Q
3039	82 – 93	739.7653	1477.5160	1477.5160	0.0047	0	66	2.4e-007	1	U	R.ETYGDMADCCEK.Q
3040	82 – 93	739.7655	1477.5164	1477.5160	0.33	0	79	1.2e-008	1	U	R.ETYGDMADCCEK.Q
3041	82 – 93	739.7658	1477.5169	1477.5160	0.67	0	66	2.3e-007	1	U	R.ETYGDMADCCEK.Q
3042	82 – 93	739.7659	1477.5173	1477.5160	0.91	0	63	5.4e-007	1	U	R.ETYGDMADCCEK.Q
4023	94 – 106	837.3874	1672.7603	1672.7627	-1.42	1	26	0.0028	1	U	K.QEPERNECFLSHK.D
4024	94 – 106	837.3875	1672.7605	1672.7627	-1.35	1	36	0.00027	1	U	K.QEPERNECFLSHK.D
4025	94 – 106	837.3876	1672.7607	1672.7627	-1.21	1	27	0.0019	1	U	K.QEPERNECFLSHK.D
4026	94 – 106	837.3880	1672.7614	1672.7627	-0.76	1	38	0.00018	1	U	K.QEPERNECFLSHK.D
4043	94 – 106	837.3887	1672.7629	1672.7627	0.11	1	25	0.0029	1	U	K.QEPERNECFLSHK.D
5635	94 – 114	847.7260	2540.1562	2540.1601	-1.55	2	22	0.0061	1	U	K.QEPERNECFLSHKDDSPDLPK.L
5636	94 – 114	847.7261	2540.1564	2540.1601	-1.48	2	21	0.0083	1	U	K.QEPERNECFLSHKDDSPDLPK.L
5637	94 – 114	847.7266	2540.1580	2540.1601	-0.83	2	17	0.021	1	U	K.QEPERNECFLSHKDDSPDLPK.L
5639	94 – 114	636.0469	2540.1584	2540.1601	-0.68	2	20	0.011	1	U	K.QEPERNECFLSHKDDSPDLPK.L
5640	94 – 114	636.0469	2540.1584	2540.1601	-0.68	2	20	0.0098	1	U	K.QEPERNECFLSHKDDSPDLPK.L
5641	94 – 114	509.0390	2540.1585	2540.1601	-0.66	2	15	0.03	1	U	K.QEPERNECFLSHKDDSPDLPK.L
5642	94 – 114	847.7268	2540.1586	2540.1601	-0.61	2	29	0.0014	1	U	K.QEPERNECFLSHKDDSPDLPK.L
5643	94 – 114	847.7270	2540.1591	2540.1601	-0.39	2	16	0.024	1	U	K.QEPERNECFLSHKDDSPDLPK.L
5644	94 – 114	636.0471	2540.1594	2540.1601	-0.30	2	18	0.016	1	U	K.QEPERNECFLSHKDDSPDLPK.L
5646	94 – 114	636.0472	2540.1599	2540.1601	-0.11	2	20	0.0097	1	U	K.QEPERNECFLSHKDDSPDLPK.L
5647	94 – 114	636.0473	2540.1601	2540.1601	-0.018	2	20	0.01	1	U	K.QEPERNECFLSHKDDSPDLPK.L
5648	94 – 114	636.0474	2540.1603	2540.1601	0.077	2	15	0.032	1	U	K.QEPERNECFLSHKDDSPDLPK.L
5649	94 – 114	509.0394	2540.1605	2540.1601	0.13	2	16	0.023	1	U	K.QEPERNECFLSHKDDSPDLPK.L
5650	94 – 114	509.0394	2540.1605	2540.1601	0.13	2	28	0.0017	1	U	K.QEPERNECFLSHKDDSPDLPK.L
5651	94 – 114	636.0474	2540.1606	2540.1601	0.17	2	20	0.0099	1	U	K.QEPERNECFLSHKDDSPDLPK.L
5652	94 – 114	636.0474	2540.1606	2540.1601	0.17	2	27	0.002	1	U	K.QEPERNECFLSHKDDSPDLPK.L
5653	94 – 114	509.0396	2540.1614	2540.1601	0.48	2	17	0.019	1	U	K.QEPERNECFLSHKDDSPDLPK.L
5654	94 – 114	509.0396	2540.1615	2540.1601	0.54	2	17	0.018	1	U	K.QEPERNECFLSHKDDSPDLPK.L
5655	94 – 114	509.0396	2540.1618	2540.1601	0.66	2	28	0.0017	1	U	K.QEPERNECFLSHKDDSPDLPK.L
1170	99 – 106	517.7392	1033.4638	1033.4651	-1.21	0	31	0.00076	1	U	R.NECFLSHK.D
1171	99 – 106	345.4953	1033.4640	1033.4651	-1.08	0	19	0.012	1	U	R.NECFLSHK.D
1172	99 – 106	517.7394	1033.4643	1033.4651	-0.75	0	34	0.00038	1	U	R.NECFLSHK.D
1173	99 – 106	345.4954	1033.4644	1033.4651	-0.65	0	38	0.00017	1	U	R.NECFLSHK.D
1174	99 – 106	517.7396	1033.4646	1033.4651	-0.52	0	22	0.0065	1	U	R.NECFLSHK.D
1175	99 – 106	517.7396	1033.4646	1033.4651	-0.52	0	26	0.0024	1	U	R.NECFLSHK.D
1176	99 – 106	345.4955	1033.4646	1033.4651	-0.48	0	34	0.00036	1	U	R.NECFLSHK.D
1177	99 – 106	345.4955	1033.4646	1033.4651	-0.48	0	26	0.0027	1	U	R.NECFLSHK.D
1178	99 – 106	517.7396	1033.4647	1033.4651	-0.40	0	34	0.00038	1	U	R.NECFLSHK.D
1179	99 – 106	517.7396	1033.4647	1033.4651	-0.40	0	34	0.00038	1	U	R.NECFLSHK.D
1181	99 – 106	345.4955	1033.4647	1033.4651	-0.39	0	34	0.00036	1	U	R.NECFLSHK.D
1182	99 – 106	345.4955	1033.4647	1033.4651	-0.39	0	34	0.00036	1	U	R.NECFLSHK.D
1183	99 – 106	345.4955	1033.4648	1033.4651	-0.30	0	27	0.0021	1	U	R.NECFLSHK.D
1184	99 – 106	345.4955	1033.4648	1033.4651	-0.30	0	27	0.0019	1	U	R.NECFLSHK.D
1185	99 – 106	517.7397	1033.4648	1033.4651	-0.27	0	34	0.00038	1	U	R.NECFLSHK.D

Query	Start – End	Observed	Mr(expt)	Mr(calc)	ppm	M	Score	Expect	Rank	U	Peptide
1186	99 – 106	345.4956	1033.4649	1033.4651	-0.21	0	26	0.0024	1	U	R.NECFLSHK.D
1187	99 – 106	517.7397	1033.4649	1033.4651	-0.15	0	34	0.00039	1	U	R.NECFLSHK.D
1188	99 – 106	517.7397	1033.4649	1033.4651	-0.15	0	34	0.00038	1	U	R.NECFLSHK.D
1189	99 – 106	517.7397	1033.4649	1033.4651	-0.15	0	34	0.00038	1	U	R.NECFLSHK.D
1190	99 – 106	345.4956	1033.4650	1033.4651	-0.098	0	19	0.013	1	U	R.NECFLSHK.D
1191	99 – 106	345.4956	1033.4650	1033.4651	-0.098	0	26	0.0024	1	U	R.NECFLSHK.D
1192	99 – 106	345.4956	1033.4650	1033.4651	-0.098	0	30	0.00099	1	U	R.NECFLSHK.D
1193	99 – 106	517.7398	1033.4651	1033.4651	-0.034	0	34	0.00043	1	U	R.NECFLSHK.D
1194	99 – 106	517.7398	1033.4651	1033.4651	-0.034	0	34	0.0004	1	U	R.NECFLSHK.D
1195	99 – 106	517.7398	1033.4651	1033.4651	-0.034	0	34	0.00037	1	U	R.NECFLSHK.D
1196	99 – 106	517.7398	1033.4651	1033.4651	-0.034	0	34	0.00037	1	U	R.NECFLSHK.D
1197	99 – 106	345.4956	1033.4651	1033.4651	-0.011	0	27	0.0022	1	U	R.NECFLSHK.D
1198	99 – 106	517.7399	1033.4652	1033.4651	0.082	0	34	0.00043	1	U	R.NECFLSHK.D
1199	99 – 106	345.4957	1033.4653	1033.4651	0.16	0	38	0.00017	1	U	R.NECFLSHK.D
1200	99 – 106	517.7401	1033.4655	1033.4651	0.43	0	34	0.00042	1	U	R.NECFLSHK.D
4757	99 – 114	951.4365	1900.8584	1900.8625	-2.18	1	64	4.1e-007	1	U	R.NECFLSHKDDSPDLPK.L
4758	99 – 114	951.4369	1900.8592	1900.8625	-1.73	1	61	8.5e-007	1	U	R.NECFLSHKDDSPDLPK.L
4759	99 – 114	951.4377	1900.8609	1900.8625	-0.84	1	63	5e-007	1	U	R.NECFLSHKDDSPDLPK.L
4760	99 – 114	951.4379	1900.8612	1900.8625	-0.70	1	63	5.1e-007	1	U	R.NECFLSHKDDSPDLPK.L
4761	99 – 114	951.4379	1900.8612	1900.8625	-0.70	1	64	4.2e-007	1	U	R.NECFLSHKDDSPDLPK.L
4762	99 – 114	634.6278	1900.8616	1900.8625	-0.48	1	56	2.3e-006	1	U	R.NECFLSHKDDSPDLPK.L
4763	99 – 114	476.2227	1900.8617	1900.8625	-0.45	1	40	9e-005	1	U	R.NECFLSHKDDSPDLPK.L
4764	99 – 114	634.6279	1900.8620	1900.8625	-0.29	1	62	6.4e-007	1	U	R.NECFLSHKDDSPDLPK.L
4765	99 – 114	634.6279	1900.8620	1900.8625	-0.29	1	50	1e-005	1	U	R.NECFLSHKDDSPDLPK.L
4766	99 – 114	634.6280	1900.8621	1900.8625	-0.20	1	62	6.3e-007	1	U	R.NECFLSHKDDSPDLPK.L
4767	99 – 114	634.6280	1900.8621	1900.8625	-0.20	1	49	1.2e-005	1	U	R.NECFLSHKDDSPDLPK.L
4768	99 – 114	476.2228	1900.8623	1900.8625	-0.14	1	46	2.3e-005	1	U	R.NECFLSHKDDSPDLPK.L
4769	99 – 114	476.2228	1900.8623	1900.8625	-0.14	1	46	2.4e-005	1	U	R.NECFLSHKDDSPDLPK.L
4770	99 – 114	476.2228	1900.8623	1900.8625	-0.14	1	41	8.5e-005	1	U	R.NECFLSHKDDSPDLPK.L
4771	99 – 114	634.6281	1900.8623	1900.8625	-0.10	1	48	1.8e-005	1	U	R.NECFLSHKDDSPDLPK.L
4772	99 – 114	634.6281	1900.8625	1900.8625	-0.0084	1	49	1.2e-005	1	U	R.NECFLSHKDDSPDLPK.L
4773	99 – 114	476.2229	1900.8626	1900.8625	0.052	1	31	0.00077	1	U	R.NECFLSHKDDSPDLPK.L
4774	99 – 114	476.2229	1900.8626	1900.8625	0.052	1	33	0.00049	1	U	R.NECFLSHKDDSPDLPK.L
4775	99 – 114	476.2229	1900.8626	1900.8625	0.052	1	43	4.7e-005	1	U	R.NECFLSHKDDSPDLPK.L
4776	99 – 114	476.2229	1900.8626	1900.8625	0.052	1	43	4.6e-005	1	U	R.NECFLSHKDDSPDLPK.L
4777	99 – 114	634.6282	1900.8627	1900.8625	0.086	1	56	2.6e-006	1	U	R.NECFLSHKDDSPDLPK.L
4778	99 – 114	476.2230	1900.8627	1900.8625	0.11	1	36	0.00027	1	U	R.NECFLSHKDDSPDLPK.L
4779	99 – 114	476.2230	1900.8627	1900.8625	0.11	1	31	0.00076	1	U	R.NECFLSHKDDSPDLPK.L
4780	99 – 114	476.2230	1900.8627	1900.8625	0.11	1	31	0.00081	1	U	R.NECFLSHKDDSPDLPK.L
4781	99 – 114	476.2230	1900.8627	1900.8625	0.11	1	41	8.8e-005	1	U	R.NECFLSHKDDSPDLPK.L
4782	99 – 114	476.2230	1900.8627	1900.8625	0.11	1	40	9e-005	1	U	R.NECFLSHKDDSPDLPK.L
4783	99 – 114	634.6282	1900.8629	1900.8625	0.18	1	59	1.3e-006	1	U	R.NECFLSHKDDSPDLPK.L
4784	99 – 114	476.2230	1900.8630	1900.8625	0.24	1	40	9e-005	1	U	R.NECFLSHKDDSPDLPK.L
4785	99 – 114	634.6283	1900.8631	1900.8625	0.29	1	62	6.3e-007	1	U	R.NECFLSHKDDSPDLPK.L
4786	99 – 114	634.6284	1900.8633	1900.8625	0.39	1	62	6.5e-007	1	U	R.NECFLSHKDDSPDLPK.L
4787	99 – 114	476.2231	1900.8633	1900.8625	0.43	1	40	8.9e-005	1	U	R.NECFLSHKDDSPDLPK.L
4788	99 – 114	634.6284	1900.8634	1900.8625	0.48	1	47	1.9e-005	1	U	R.NECFLSHKDDSPDLPK.L
4789	99 – 114	476.2231	1900.8635	1900.8625	0.49	1	43	4.7e-005	1	U	R.NECFLSHKDDSPDLPK.L
4790	99 – 114	634.6287	1900.8643	1900.8625	0.95	1	61	7.1e-007	1	U	R.NECFLSHKDDSPDLPK.L
4791	99 – 114	634.6289	1900.8647	1900.8625	1.16	1	48	1.4e-005	1	U	R.NECFLSHKDDSPDLPK.L
4792	99 – 114	634.6290	1900.8653	1900.8625	1.44	1	49	1.2e-005	1	U	R.NECFLSHKDDSPDLPK.L
611	107 – 114	443.7111	885.4076	885.4080	-0.46	0	42	5.7e-005	1	U	K.DDSPDLPK.L
612	107 – 114	443.7111	885.4077	885.4080	-0.26	0	42	5.7e-005	1	U	K.DDSPDLPK.L
613	107 – 114	443.7111	885.4077	885.4080	-0.26	0	45	2.9e-005	1	U	K.DDSPDLPK.L
614	107 – 114	443.7112	885.4078	885.4080	-0.19	0	36	0.00023	1	U	K.DDSPDLPK.L
615	107 – 114	443.7112	885.4078	885.4080	-0.19	0	33	0.00045	1	U	K.DDSPDLPK.L
616	107 – 114	443.7112	885.4079	885.4080	-0.12	0	41	8.2e-005	1	U	K.DDSPDLPK.L
617	107 – 114	443.7113	885.4080	885.4080	0.016	0	39	0.00012	1	U	K.DDSPDLPK.L
618	107 – 114	443.7113	885.4080	885.4080	0.016	0	36	0.00023	1	U	K.DDSPDLPK.L
619	107 – 114	443.7113	885.4080	885.4080	0.016	0	39	0.00014	1	U	K.DDSPDLPK.L
620	107 – 114	443.7113	885.4080	885.4080	0.016	0	39	0.00014	1	U	K.DDSPDLPK.L
621	107 – 114	443.7113	885.4080	885.4080	0.016	0	43	5.6e-005	1	U	K.DDSPDLPK.L
622	107 – 114	443.7113	885.4080	885.4080	0.084	0	45	2.9e-005	1	U	K.DDSPDLPK.L
623	107 – 114	443.7113	885.4081	885.4080	0.15	0	39	0.00014	1	U	K.DDSPDLPK.L
624	107 – 114	443.7114	885.4082	885.4080	0.22	0	46	2.8e-005	1	U	K.DDSPDLPK.L
625	107 – 114	443.7114	885.4082	885.4080	0.22	0	46	2.8e-005	1	U	K.DDSPDLPK.L
626	107 – 114	443.7114	885.4082	885.4080	0.31	0	45	2.9e-005	1	U	K.DDSPDLPK.L
627	107 – 114	443.7115	885.4084	885.4080	0.51	0	43	5.6e-005	1	U	K.DDSPDLPK.L
628	107 – 114	443.7123	885.4100	885.4080	2.30	0	42	5.6e-005	1	U	K.DDSPDLPK.L
5710	107 – 127	869.4101	2605.2085	2605.2105	-0.79	2	78	1.5e-008	1	U	K.DDSPDLPKLKPDPNTLCDEFK.A + Glycation (
5711	107 – 127	869.4104	2605.2094	2605.2105	-0.44	2	73	5.2e-008	1	U	K.DDSPDLPKLKPDPNTLCDEFK.A + Glycation (
5712	107 – 127	869.4105	2605.2097	2605.2105	-0.30	2	88	1.5e-009	1	U	K.DDSPDLPKLKPDPNTLCDEFK.A + Glycation (
5713	107 – 127	652.3104	2605.2126	2605.2105	0.79	2	51	8e-006	1	U	K.DDSPDLPKLKPDPNTLCDEFK.A + Glycation (
5714	107 – 127	652.3109	2605.2145	2605.2105	1.54	2	81	7.7e-009	1	U	K.DDSPDLPKLKPDPNTLCDEFK.A + Glycation (
3589	115 – 127	526.2597	1575.7573	1575.7603	-1.91	1	19	0.014	1	U	K.LKPDPNTLCDEFK.A
3590	115 – 127	788.8863	1575.7581	1575.7603	-1.36	1	40	0.00011	1	U	K.LKPDPNTLCDEFK.A
3591	115 – 127	788.8863	1575.7581	1575.7603	-1.36	1	52	5.8e-006	1	U	K.LKPDPNTLCDEFK.A
3592	115 – 127	788.8863	1575.7581	1575.7603	-1.36	1	69	1.3e-007	1	U	K.LKPDPNTLCDEFK.A
3593	115 – 127	788.8865	1575.7585	1575.7603	-1.11	1	56	2.3e-006	1	U	K.LKPDPNTLCDEFK.A
3594	115 – 127	788.8865	1575.7585	1575.7603	-1.11	1	62	7.1e-007	1	U	K.LKPDPNTLCDEFK.A
3595	115 – 127	788.8865	1575.7585	1575.7603	-1.11	1	83	5.5e-009	1	U	K.LKPDPNTLCDEFK.A
3596	115 – 127	788.8866	1575.7586	1575.7603	-1.04	1	62	6.6e-007	1	U	K.LKPDPNTLCDEFK.A
3597	115 – 127	788.8866	1575.7586	1575.7603	-1.04	1	96	2.4e-010	1	U	K.LKPDPNTLCDEFK.A
3598	115 – 127	788.8867	1575.7588	1575.7603	-0.96	1	43	5e-005	1	U	K.LKPDPNTLCDEFK.A
3599	115 – 127	788.8867	1575.7588	1575.7603	-0.96	1	61	7.3e-007	1	U	K.LKPDPNTLCDEFK.A
3600	115 – 127	788.8867	1575.7589	1575.7603	-0.89	1	61	7.2e-007	1	U	K.LKPDPNTLCDEFK.A
3601	115 – 127	788.8867	1575.7589	1575.7603	-0.89	1	58	1.6e-006	1	U	K.LKPDPNTLCDEFK.A
3602	115 – 127	788.8868	1575.7590	1575.7603	-0.81	1	65	3.3e-007	1	U	K.LKPDPNTLCDEFK.A
3603	115 – 127	788.8868	1575.7590	1575.7603	-0.81	1	70	9.9e-008	1	U	K.LKPDPNTLCDEFK.A
3604	115 – 127	788.8868	1575.7590	1575.7603	-0.81	1	83	5.4e-009	1	U	K.LKPDPNTLCDEFK.A

Query	Start – End	Observed	Mr(expt)	Mr(calc)	ppm	M	Score	Expect	Rank	U	Peptide
3605	115 – 127	788.8868	1575.7591	1575.7603	-0.73	1	65	3.2e-007	1	U	K.LKPDPNTLCDEFK.A
3606	115 – 127	788.8868	1575.7591	1575.7603	-0.73	1	66	2.8e-007	1	U	K.LKPDPNTLCDEFK.A
3607	115 – 127	788.8870	1575.7595	1575.7603	-0.51	1	66	2.8e-007	1	U	K.LKPDPNTLCDEFK.A
3608	115 – 127	788.8870	1575.7595	1575.7603	-0.51	1	87	2e-009	1	U	K.LKPDPNTLCDEFK.A
3609	115 – 127	788.8870	1575.7595	1575.7603	-0.51	1	61	7.2e-007	1	U	K.LKPDPNTLCDEFK.A
3610	115 – 127	788.8870	1575.7595	1575.7603	-0.51	1	62	7e-007	1	U	K.LKPDPNTLCDEFK.A
3611	115 – 127	788.8871	1575.7596	1575.7603	-0.43	1	65	3.3e-007	1	U	K.LKPDPNTLCDEFK.A
3612	115 – 127	788.8873	1575.7600	1575.7603	-0.19	1	66	2.7e-007	1	U	K.LKPDPNTLCDEFK.A
3613	115 – 127	526.2606	1575.7600	1575.7603	-0.16	1	30	0.00092	1	U	K.LKPDPNTLCDEFK.A
3614	115 – 127	526.2606	1575.7600	1575.7603	-0.16	1	54	3.7e-006	1	U	K.LKPDPNTLCDEFK.A
3615	115 – 127	526.2607	1575.7604	1575.7603	0.069	1	37	0.00019	1	U	K.LKPDPNTLCDEFK.A
3616	115 – 127	526.2607	1575.7604	1575.7603	0.069	1	47	1.9e-005	1	U	K.LKPDPNTLCDEFK.A
3617	115 – 127	526.2608	1575.7606	1575.7603	0.18	1	44	4.2e-005	1	U	K.LKPDPNTLCDEFK.A
3618	115 – 127	526.2608	1575.7606	1575.7603	0.18	1	48	1.5e-005	1	U	K.LKPDPNTLCDEFK.A
3619	115 – 127	526.2608	1575.7606	1575.7603	0.18	1	36	0.00028	1	U	K.LKPDPNTLCDEFK.A
3620	115 – 127	526.2608	1575.7606	1575.7603	0.18	1	55	3.1e-006	1	U	K.LKPDPNTLCDEFK.A
3621	115 – 127	526.2608	1575.7606	1575.7603	0.18	1	61	8.6e-007	1	U	K.LKPDPNTLCDEFK.A
3622	115 – 127	526.2608	1575.7606	1575.7603	0.18	1	59	1.3e-006	1	U	K.LKPDPNTLCDEFK.A
3623	115 – 127	526.2609	1575.7608	1575.7603	0.30	1	37	0.00019	1	U	K.LKPDPNTLCDEFK.A
3624	115 – 127	526.2609	1575.7608	1575.7603	0.30	1	52	5.7e-006	1	U	K.LKPDPNTLCDEFK.A
3625	115 – 127	526.2609	1575.7608	1575.7603	0.30	1	54	4.1e-006	1	U	K.LKPDPNTLCDEFK.A
3626	115 – 127	526.2609	1575.7608	1575.7603	0.30	1	45	2.9e-005	1	U	K.LKPDPNTLCDEFK.A
3627	115 – 127	526.2609	1575.7610	1575.7603	0.43	1	57	2.1e-006	1	U	K.LKPDPNTLCDEFK.A
3628	115 – 127	526.2609	1575.7610	1575.7603	0.43	1	55	2.9e-006	1	U	K.LKPDPNTLCDEFK.A
3629	115 – 127	526.2609	1575.7610	1575.7603	0.43	1	50	1e-005	1	U	K.LKPDPNTLCDEFK.A
3630	115 – 127	526.2610	1575.7613	1575.7603	0.66	1	45	3.1e-005	1	U	K.LKPDPNTLCDEFK.A
3631	115 – 127	526.2610	1575.7613	1575.7603	0.66	1	50	1.1e-005	1	U	K.LKPDPNTLCDEFK.A
3632	115 – 127	526.2610	1575.7613	1575.7603	0.66	1	67	1.9e-007	1	U	K.LKPDPNTLCDEFK.A
3633	115 – 127	788.8879	1575.7613	1575.7603	0.66	1	70	1.1e-007	1	U	K.LKPDPNTLCDEFK.A
3634	115 – 127	526.2613	1575.7620	1575.7603	1.12	1	39	0.00014	1	U	K.LKPDPNTLCDEFK.A
3635	115 – 127	526.2617	1575.7632	1575.7603	1.82	1	57	2.1e-006	1	U	K.LKPDPNTLCDEFK.A
3636	115 – 127	526.2617	1575.7633	1575.7603	1.93	1	57	2.2e-006	1	U	K.LKPDPNTLCDEFK.A
3637	115 – 127	526.2618	1575.7635	1575.7603	2.05	1	55	3.5e-006	1	U	K.LKPDPNTLCDEFK.A
3638	115 – 127	526.2620	1575.7642	1575.7603	2.51	1	47	2.1e-005	1	U	K.LKPDPNTLCDEFK.A
4257	115 – 127	869.9130	1737.8114	1737.8131	-1.00	1	31	0.00085	1	U	K.LKPDPNTLCDEFK.A + Glycation (K)
4258	115 – 127	869.9130	1737.8115	1737.8131	-0.93	1	39	0.00012	1	U	K.LKPDPNTLCDEFK.A + Glycation (K)
4259	115 – 127	869.9131	1737.8117	1737.8131	-0.78	1	45	3.3e-005	1	U	K.LKPDPNTLCDEFK.A + Glycation (K)
4260	115 – 127	580.2783	1737.8130	1737.8131	-0.091	1	34	0.00042	1	U	K.LKPDPNTLCDEFK.A + Glycation (K)
4261	115 – 127	869.9138	1737.8130	1737.8131	-0.082	1	39	0.00013	1	U	K.LKPDPNTLCDEFK.A + Glycation (K)
4262	115 – 127	580.2783	1737.8131	1737.8131	0.013	1	29	0.0012	1	U	K.LKPDPNTLCDEFK.A + Glycation (K)
4263	115 – 127	580.2786	1737.8139	1737.8131	0.43	1	36	0.00024	1	U	K.LKPDPNTLCDEFK.A + Glycation (K)
4264	115 – 127	580.2787	1737.8144	1737.8131	0.75	1	26	0.0027	1	U	K.LKPDPNTLCDEFK.A + Glycation (K)
5137	115 – 131	673.9932	2018.9577	2018.9619	-2.10	2	57	2.1e-006	1	U	K.LKPDPNTLCDEFKADEK.K
5138	115 – 131	673.9940	2018.9602	2018.9619	-0.82	2	72	6.2e-008	1	U	K.LKPDPNTLCDEFKADEK.K
5139	115 – 131	1010.4874	2018.9603	2018.9619	-0.78	2	69	1.2e-007	1	U	K.LKPDPNTLCDEFKADEK.K
5140	115 – 131	505.7474	2018.9605	2018.9619	-0.67	2	77	1.8e-008	1	U	K.LKPDPNTLCDEFKADEK.K
5141	115 – 131	673.9942	2018.9608	2018.9619	-0.55	2	72	5.8e-008	1	U	K.LKPDPNTLCDEFKADEK.K
5142	115 – 131	673.9942	2018.9608	2018.9619	-0.55	2	84	3.6e-009	1	U	K.LKPDPNTLCDEFKADEK.K
5143	115 – 131	673.9943	2018.9610	2018.9619	-0.46	2	72	6.1e-008	1	U	K.LKPDPNTLCDEFKADEK.K
5144	115 – 131	505.7475	2018.9610	2018.9619	-0.43	2	51	8.4e-006	1	U	K.LKPDPNTLCDEFKADEK.K
5145	115 – 131	1010.4878	2018.9610	2018.9619	-0.42	2	80	9e-009	1	U	K.LKPDPNTLCDEFKADEK.K
5146	115 – 131	1010.4879	2018.9611	2018.9619	-0.36	2	97	1.9e-010	1	U	K.LKPDPNTLCDEFKADEK.K
5147	115 – 131	673.9944	2018.9613	2018.9619	-0.28	2	84	3.6e-009	1	U	K.LKPDPNTLCDEFKADEK.K
5148	115 – 131	1010.4881	2018.9616	2018.9619	-0.12	2	65	3.1e-007	1	U	K.LKPDPNTLCDEFKADEK.K
5149	115 – 131	673.9946	2018.9619	2018.9619	-0.00099	2	65	3.5e-007	1	U	K.LKPDPNTLCDEFKADEK.K
5150	115 – 131	673.9946	2018.9621	2018.9619	0.088	2	84	3.6e-009	1	U	K.LKPDPNTLCDEFKADEK.K
5151	115 – 131	673.9947	2018.9624	2018.9619	0.27	2	89	1.4e-009	1	U	K.LKPDPNTLCDEFKADEK.K
5152	115 – 131	505.7479	2018.9625	2018.9619	0.28	2	54	4.3e-006	1	U	K.LKPDPNTLCDEFKADEK.K
5153	115 – 131	505.7480	2018.9629	2018.9619	0.48	2	64	3.8e-007	1	U	K.LKPDPNTLCDEFKADEK.K
5154	115 – 131	673.9949	2018.9630	2018.9619	0.53	2	78	1.6e-008	1	U	K.LKPDPNTLCDEFKADEK.K
5155	115 – 131	673.9949	2018.9630	2018.9619	0.53	2	77	1.8e-008	1	U	K.LKPDPNTLCDEFKADEK.K
5156	115 – 131	505.7480	2018.9630	2018.9619	0.54	2	50	1.1e-005	1	U	K.LKPDPNTLCDEFKADEK.K
5157	115 – 131	505.7481	2018.9632	2018.9619	0.66	2	50	9.7e-006	1	U	K.LKPDPNTLCDEFKADEK.K
5390	115 – 131	728.0109	2181.0108	2181.0147	-1.81	2	30	0.00099	1	U	K.LKPDPNTLCDEFKADEK.K + Glycation (K)
5391	115 – 131	728.0116	2181.0130	2181.0147	-0.80	2	15	0.034	1	U	K.LKPDPNTLCDEFKADEK.K + Glycation (K)
5392	115 – 131	728.0118	2181.0137	2181.0147	-0.47	2	14	0.037	1	U	K.LKPDPNTLCDEFKADEK.K + Glycation (K)
5393	115 – 131	728.0120	2181.0142	2181.0147	-0.22	2	17	0.018	1	U	K.LKPDPNTLCDEFKADEK.K + Glycation (K)
5394	115 – 131	728.0122	2181.0148	2181.0147	0.043	2	28	0.0016	1	U	K.LKPDPNTLCDEFKADEK.K + Glycation (K)
127	132 – 136	333.1917	664.3688	664.3697	-1.30	1	24	0.0037	1	U	K.KFWGK.Y
128	132 – 136	333.1917	664.3689	664.3697	-1.21	1	24	0.0039	1	U	K.KFWGK.Y
129	132 – 136	333.1919	664.3692	664.3697	-0.76	1	21	0.0082	1	U	K.KFWGK.Y
130	132 – 136	333.1919	664.3692	664.3697	-0.76	1	21	0.0084	1	U	K.KFWGK.Y
131	132 – 136	333.1919	664.3692	664.3697	-0.67	1	21	0.0083	1	U	K.KFWGK.Y
132	132 – 136	333.1919	664.3692	664.3697	-0.67	1	24	0.0039	1	U	K.KFWGK.Y
133	132 – 136	333.1919	664.3693	664.3697	-0.55	1	21	0.0085	1	U	K.KFWGK.Y
134	132 – 136	333.1919	664.3693	664.3697	-0.55	1	24	0.0044	1	U	K.KFWGK.Y
135	132 – 136	333.1919	664.3693	664.3697	-0.55	1	21	0.0084	1	U	K.KFWGK.Y
136	132 – 136	333.1919	664.3693	664.3697	-0.55	1	24	0.004	1	U	K.KFWGK.Y
137	132 – 136	333.1919	664.3693	664.3697	-0.55	1	24	0.004	1	U	K.KFWGK.Y
138	132 – 136	333.1919	664.3693	664.3697	-0.55	1	21	0.0084	1	U	K.KFWGK.Y
139	132 – 136	333.1919	664.3693	664.3697	-0.55	1	21	0.0084	1	U	K.KFWGK.Y
140	132 – 136	333.1919	664.3693	664.3697	-0.55	1	21	0.0085	1	U	K.KFWGK.Y
141	132 – 136	333.1920	664.3694	664.3697	-0.46	1	18	0.015	1	U	K.KFWGK.Y
142	132 – 136	333.1920	664.3694	664.3697	-0.37	1	24	0.004	1	U	K.KFWGK.Y
143	132 – 136	333.1920	664.3694	664.3697	-0.37	1	24	0.004	1	U	K.KFWGK.Y
144	132 – 136	333.1920	664.3694	664.3697	-0.37	1	24	0.004	1	U	K.KFWGK.Y
145	132 – 136	333.1920	664.3694	664.3697	-0.37	1	24	0.004	1	U	K.KFWGK.Y
146	132 – 136	333.1920	664.3694	664.3697	-0.37	1	21	0.0085	1	U	K.KFWGK.Y
148	132 – 136	333.1920	664.3695	664.3697	-0.28	1	24	0.004	1	U	K.KFWGK.Y
149	132 – 136	333.1920	664.3695	664.3697	-0.28	1	24	0.0039	1	U	K.KFWGK.Y

Query	Start - End	Observed	Mr(expt)	Mr(calc)	ppm	M	Score	Expect	Rank	U	Peptide
150	132 - 136	333.1920	664.3695	664.3697	-0.28	1	21	0.0086	1	U	K.KFWGK.Y
151	132 - 136	333.1920	664.3695	664.3697	-0.28	1	21	0.0084	1	U	K.KFWGK.Y
153	132 - 136	333.1920	664.3695	664.3697	-0.19	1	21	0.0083	1	U	K.KFWGK.Y
154	132 - 136	333.1920	664.3695	664.3697	-0.19	1	24	0.0042	1	U	K.KFWGK.Y
155	132 - 136	333.1920	664.3695	664.3697	-0.19	1	21	0.0083	1	U	K.KFWGK.Y
156	132 - 136	333.1920	664.3695	664.3697	-0.19	1	21	0.0085	1	U	K.KFWGK.Y
159	132 - 136	333.1921	664.3697	664.3697	-0.011	1	23	0.0045	1	U	K.KFWGK.Y
160	132 - 136	333.1921	664.3697	664.3697	-0.011	1	24	0.004	1	U	K.KFWGK.Y
161	132 - 136	333.1921	664.3697	664.3697	-0.011	1	21	0.0083	1	U	K.KFWGK.Y
164	132 - 136	333.1921	664.3697	664.3697	0.080	1	24	0.0041	1	U	K.KFWGK.Y
165	132 - 136	333.1921	664.3697	664.3697	0.080	1	21	0.0084	1	U	K.KFWGK.Y
166	132 - 136	333.1922	664.3698	664.3697	0.17	1	21	0.0084	1	U	K.KFWGK.Y
4242	132 - 143	868.4545	1734.8944	1734.8981	-2.12	2	57	1.9e-006	1	U	K.KFWGKYLYEIAR.R + Glycation (K)
4243	132 - 143	868.4553	1734.8960	1734.8981	-1.21	2	57	1.9e-006	1	U	K.KFWGKYLYEIAR.R + Glycation (K)
4244	132 - 143	868.4555	1734.8965	1734.8981	-0.92	2	57	1.9e-006	1	U	K.KFWGKYLYEIAR.R + Glycation (K)
4245	132 - 143	868.4561	1734.8975	1734.8981	-0.30	2	43	4.7e-005	1	U	K.KFWGKYLYEIAR.R + Glycation (K)
4246	132 - 143	434.7317	1734.8979	1734.8981	-0.10	2	19	0.012	1	U	K.KFWGKYLYEIAR.R + Glycation (K)
4247	132 - 143	579.3066	1734.8979	1734.8981	-0.092	2	43	4.9e-005	1	U	K.KFWGKYLYEIAR.R + Glycation (K)
4248	132 - 143	434.7318	1734.8981	1734.8981	0.037	2	22	0.0061	1	U	K.KFWGKYLYEIAR.R + Glycation (K)
4249	132 - 143	434.7319	1734.8984	1734.8981	0.18	2	20	0.011	1	U	K.KFWGKYLYEIAR.R + Glycation (K)
4250	132 - 143	579.3068	1734.8985	1734.8981	0.22	2	60	9.5e-007	1	U	K.KFWGKYLYEIAR.R + Glycation (K)
4251	132 - 143	434.7321	1734.8991	1734.8981	0.61	2	24	0.0044	1	U	K.KFWGKYLYEIAR.R + Glycation (K)
4252	132 - 143	579.3072	1734.8997	1734.8981	0.96	2	49	1.3e-005	1	U	K.KFWGKYLYEIAR.R + Glycation (K)
4253	132 - 143	579.3074	1734.9003	1734.8981	1.27	2	41	8.1e-005	1	U	K.KFWGKYLYEIAR.R + Glycation (K)
4254	132 - 143	579.3077	1734.9012	1734.8981	1.81	2	60	9.4e-007	1	U	K.KFWGKYLYEIAR.R + Glycation (K)
4255	132 - 143	579.3079	1734.9018	1734.8981	2.12	2	60	9.4e-007	1	U	K.KFWGKYLYEIAR.R + Glycation (K)
4256	132 - 143	579.3079	1734.9019	1734.8981	2.23	2	60	9.4e-007	1	U	K.KFWGKYLYEIAR.R + Glycation (K)
4195	132 - 144	433.2443	1728.9479	1728.9463	0.92	3	36	0.00023	1	U	K.KFWGKYLYEIARR.H
4196	132 - 144	577.3240	1728.9501	1728.9463	2.16	3	25	0.0033	1	U	K.KFWGKYLYEIARR.H
4197	132 - 144	577.3240	1728.9501	1728.9463	2.16	3	32	0.0007	1	U	K.KFWGKYLYEIARR.H
3734	133 - 143	804.4083	1606.8020	1606.8031	-0.71	1	46	2.4e-005	1	U	K.FWGKYLYEIAR.R + Glycation (K)
3735	133 - 143	804.4084	1606.8022	1606.8031	-0.55	1	46	2.4e-005	1	U	K.FWGKYLYEIAR.R + Glycation (K)
3736	133 - 143	804.4088	1606.8029	1606.8031	-0.10	1	36	0.00026	1	U	K.FWGKYLYEIAR.R + Glycation (K)
3737	133 - 143	804.4088	1606.8031	1606.8031	-0.030	1	52	5.7e-006	1	U	K.FWGKYLYEIAR.R + Glycation (K)
3738	133 - 143	536.6083	1606.8032	1606.8031	0.047	1	28	0.0015	1	U	K.FWGKYLYEIAR.R + Glycation (K)
3739	133 - 143	536.6084	1606.8034	1606.8031	0.16	1	27	0.0022	1	U	K.FWGKYLYEIAR.R + Glycation (K)
758	137 - 143	464.2498	926.4851	926.4861	-1.06	0	13	0.049	1	U	K.YLYEIAR.R
759	137 - 143	464.2502	926.4858	926.4861	-0.35	0	33	0.00054	1	U	K.YLYEIAR.R
760	137 - 143	464.2503	926.4861	926.4861	-0.067	0	22	0.007	1	U	K.YLYEIAR.R
761	137 - 143	464.2503	926.4861	926.4861	-0.067	0	18	0.014	1	U	K.YLYEIAR.R
762	137 - 143	464.2503	926.4861	926.4861	-0.067	0	30	0.0009	1	U	K.YLYEIAR.R
763	137 - 143	464.2503	926.4861	926.4861	-0.0022	0	13	0.05	1	U	K.YLYEIAR.R
764	137 - 143	464.2503	926.4861	926.4861	-0.0022	0	18	0.015	1	U	K.YLYEIAR.R
765	137 - 143	464.2503	926.4861	926.4861	-0.0022	0	17	0.018	1	U	K.YLYEIAR.R
766	137 - 143	464.2503	926.4861	926.4861	-0.0022	0	30	0.00094	1	U	K.YLYEIAR.R
767	137 - 143	464.2504	926.4862	926.4861	0.13	0	27	0.0021	1	U	K.YLYEIAR.R
768	137 - 143	464.2504	926.4863	926.4861	0.19	0	14	0.037	1	U	K.YLYEIAR.R
769	137 - 143	464.2504	926.4863	926.4861	0.19	0	18	0.015	1	U	K.YLYEIAR.R
770	137 - 143	464.2504	926.4863	926.4861	0.19	0	28	0.0015	1	U	K.YLYEIAR.R
771	137 - 143	464.2504	926.4863	926.4861	0.19	0	24	0.0037	1	U	K.YLYEIAR.R
772	137 - 143	464.2504	926.4863	926.4861	0.19	0	32	0.00069	1	U	K.YLYEIAR.R
774	137 - 143	464.2504	926.4863	926.4861	0.19	0	27	0.0021	1	U	K.YLYEIAR.R
775	137 - 143	464.2504	926.4863	926.4861	0.19	0	30	0.00099	1	U	K.YLYEIAR.R
776	137 - 143	464.2505	926.4864	926.4861	0.26	0	28	0.0015	1	U	K.YLYEIAR.R
777	137 - 143	464.2505	926.4864	926.4861	0.32	0	30	0.0009	1	U	K.YLYEIAR.R
778	137 - 143	464.2505	926.4864	926.4861	0.32	0	30	0.00093	1	U	K.YLYEIAR.R
779	137 - 143	464.2505	926.4864	926.4861	0.32	0	14	0.044	1	U	K.YLYEIAR.R
780	137 - 143	464.2505	926.4865	926.4861	0.45	0	27	0.0019	1	U	K.YLYEIAR.R
781	137 - 143	464.2505	926.4865	926.4861	0.45	0	26	0.0027	1	U	K.YLYEIAR.R
782	137 - 143	464.2506	926.4866	926.4861	0.52	0	27	0.0022	1	U	K.YLYEIAR.R
784	137 - 143	464.2506	926.4867	926.4861	0.58	0	30	0.00091	1	U	K.YLYEIAR.R
786	137 - 143	464.2508	926.4870	926.4861	0.97	0	29	0.0011	1	U	K.YLYEIAR.R
787	137 - 143	464.2508	926.4871	926.4861	1.03	0	30	0.00094	1	U	K.YLYEIAR.R
5197	144 - 159	682.3464	2044.0173	2044.0206	-1.64	1	35	0.00033	1	U	R.RHPYFYAPELLYYANK.Y
5198	144 - 159	1023.0166	2044.0186	2044.0206	-0.97	1	44	4e-005	1	U	R.RHPYFYAPELLYYANK.Y
5199	144 - 159	1023.0167	2044.0188	2044.0206	-0.91	1	42	6.8e-005	1	U	R.RHPYFYAPELLYYANK.Y
5200	144 - 159	1023.0167	2044.0189	2044.0206	-0.85	1	46	2.4e-005	1	U	R.RHPYFYAPELLYYANK.Y
5201	144 - 159	682.3469	2044.0189	2044.0206	-0.83	1	40	9.8e-005	1	U	R.RHPYFYAPELLYYANK.Y
5202	144 - 159	1023.0169	2044.0191	2044.0206	-0.73	1	46	2.3e-005	1	U	R.RHPYFYAPELLYYANK.Y
5203	144 - 159	1023.0169	2044.0193	2044.0206	-0.67	1	41	7.6e-005	1	U	R.RHPYFYAPELLYYANK.Y
5204	144 - 159	1023.0169	2044.0193	2044.0206	-0.67	1	50	1.1e-005	1	U	R.RHPYFYAPELLYYANK.Y
5205	144 - 159	1023.0169	2044.0193	2044.0206	-0.67	1	38	0.00017	1	U	R.RHPYFYAPELLYYANK.Y
5206	144 - 159	682.3470	2044.0193	2044.0206	-0.64	1	41	7.2e-005	1	U	R.RHPYFYAPELLYYANK.Y
5207	144 - 159	682.3470	2044.0193	2044.0206	-0.64	1	41	7.6e-005	1	U	R.RHPYFYAPELLYYANK.Y
5208	144 - 159	1023.0170	2044.0194	2044.0206	-0.61	1	43	5.5e-005	1	U	R.RHPYFYAPELLYYANK.Y
5209	144 - 159	682.3471	2044.0195	2044.0206	-0.55	1	39	0.00012	1	U	R.RHPYFYAPELLYYANK.Y
5210	144 - 159	1023.0171	2044.0196	2044.0206	-0.49	1	44	3.7e-005	1	U	R.RHPYFYAPELLYYANK.Y
5211	144 - 159	1023.0172	2044.0197	2044.0206	-0.43	1	37	0.00019	1	U	R.RHPYFYAPELLYYANK.Y
5212	144 - 159	1023.0172	2044.0197	2044.0206	-0.43	1	45	3.3e-005	1	U	R.RHPYFYAPELLYYANK.Y
5213	144 - 159	682.3472	2044.0199	2044.0206	-0.38	1	40	0.0001	1	U	R.RHPYFYAPELLYYANK.Y
5214	144 - 159	682.3472	2044.0199	2044.0206	-0.38	1	41	7.7e-005	1	U	R.RHPYFYAPELLYYANK.Y
5215	144 - 159	1023.0172	2044.0199	2044.0206	-0.37	1	44	3.8e-005	1	U	R.RHPYFYAPELLYYANK.Y
5216	144 - 159	682.3473	2044.0200	2044.0206	-0.29	1	33	0.00053	1	U	R.RHPYFYAPELLYYANK.Y
5217	144 - 159	1023.0173	2044.0201	2044.0206	-0.26	1	55	3.5e-006	1	U	R.RHPYFYAPELLYYANK.Y
5218	144 - 159	682.3474	2044.0202	2044.0206	-0.20	1	42	6.3e-005	1	U	R.RHPYFYAPELLYYANK.Y
5219	144 - 159	682.3474	2044.0202	2044.0206	-0.20	1	39	0.00012	1	U	R.RHPYFYAPELLYYANK.Y
5220	144 - 159	682.3474	2044.0202	2044.0206	-0.20	1	41	8.5e-005	1	U	R.RHPYFYAPELLYYANK.Y
5221	144 - 159	682.3474	2044.0202	2044.0206	-0.20	1	32	0.00062	1	U	R.RHPYFYAPELLYYANK.Y
5222	144 - 159	682.3474	2044.0202	2044.0206	-0.20	1	42	6.4e-005	1	U	R.RHPYFYAPELLYYANK.Y

Query	Start - End	Observed	Mr(expt)	Mr(calc)	ppm	M	Score	Expect	Rank	U	Peptide
5223	144 - 159	682.3474	2044.0204	2044.0206	-0.11	1	32	0.00066	1	U	R.RHPYFYAPELLYYANK.Y
5224	144 - 159	682.3474	2044.0204	2044.0206	-0.11	1	31	0.00085	1	U	R.RHPYFYAPELLYYANK.Y
5225	144 - 159	512.0124	2044.0205	2044.0206	-0.085	1	36	0.00025	1	U	R.RHPYFYAPELLYYANK.Y
5226	144 - 159	682.3475	2044.0206	2044.0206	-0.023	1	37	0.0002	1	U	R.RHPYFYAPELLYYANK.Y
5227	144 - 159	512.0124	2044.0207	2044.0206	0.032	1	41	7.4e-005	1	U	R.RHPYFYAPELLYYANK.Y
5228	144 - 159	1023.0176	2044.0207	2044.0206	0.048	1	40	0.00011	1	U	R.RHPYFYAPELLYYANK.Y
5229	144 - 159	682.3475	2044.0208	2044.0206	0.065	1	35	0.00033	1	U	R.RHPYFYAPELLYYANK.Y
5230	144 - 159	512.0125	2044.0209	2044.0206	0.15	1	47	1.8e-005	1	U	R.RHPYFYAPELLYYANK.Y
5231	144 - 159	512.0125	2044.0209	2044.0206	0.15	1	34	0.00036	1	U	R.RHPYFYAPELLYYANK.Y
5232	144 - 159	512.0125	2044.0209	2044.0206	0.15	1	44	3.6e-005	1	U	R.RHPYFYAPELLYYANK.Y
5233	144 - 159	512.0125	2044.0209	2044.0206	0.15	1	40	0.0001	1	U	R.RHPYFYAPELLYYANK.Y
5234	144 - 159	512.0125	2044.0209	2044.0206	0.15	1	40	9.1e-005	1	U	R.RHPYFYAPELLYYANK.Y
5235	144 - 159	682.3476	2044.0210	2044.0206	0.17	1	45	3e-005	1	U	R.RHPYFYAPELLYYANK.Y
5236	144 - 159	682.3476	2044.0210	2044.0206	0.17	1	43	5e-005	1	U	R.RHPYFYAPELLYYANK.Y
5237	144 - 159	682.3477	2044.0212	2044.0206	0.26	1	39	0.00012	1	U	R.RHPYFYAPELLYYANK.Y
5238	144 - 159	682.3477	2044.0212	2044.0206	0.26	1	42	6.2e-005	1	U	R.RHPYFYAPELLYYANK.Y
5239	144 - 159	682.3477	2044.0212	2044.0206	0.26	1	39	0.00012	1	U	R.RHPYFYAPELLYYANK.Y
5240	144 - 159	512.0126	2044.0212	2044.0206	0.27	1	32	0.00069	1	U	R.RHPYFYAPELLYYANK.Y
5241	144 - 159	512.0126	2044.0212	2044.0206	0.27	1	27	0.0021	1	U	R.RHPYFYAPELLYYANK.Y
5242	144 - 159	512.0126	2044.0212	2044.0206	0.27	1	49	1.4e-005	1	U	R.RHPYFYAPELLYYANK.Y
5243	144 - 159	512.0126	2044.0214	2044.0206	0.38	1	32	0.00064	1	U	R.RHPYFYAPELLYYANK.Y
5244	144 - 159	512.0126	2044.0214	2044.0206	0.38	1	50	9.3e-006	1	U	R.RHPYFYAPELLYYANK.Y
5245	144 - 159	682.3478	2044.0217	2044.0206	0.52	1	44	3.6e-005	1	U	R.RHPYFYAPELLYYANK.Y
5246	144 - 159	512.0127	2044.0217	2044.0206	0.52	1	28	0.0015	1	U	R.RHPYFYAPELLYYANK.Y
5247	144 - 159	512.0127	2044.0217	2044.0206	0.52	1	31	0.00078	1	U	R.RHPYFYAPELLYYANK.Y
5248	144 - 159	512.0127	2044.0217	2044.0206	0.52	1	44	3.6e-005	1	U	R.RHPYFYAPELLYYANK.Y
5249	144 - 159	512.0127	2044.0217	2044.0206	0.52	1	49	1.2e-005	1	U	R.RHPYFYAPELLYYANK.Y
5250	144 - 159	512.0128	2044.0219	2044.0206	0.64	1	25	0.0029	1	U	R.RHPYFYAPELLYYANK.Y
5251	144 - 159	512.0128	2044.0219	2044.0206	0.64	1	40	0.0001	1	U	R.RHPYFYAPELLYYANK.Y
5252	144 - 159	512.0128	2044.0219	2044.0206	0.64	1	41	7.6e-005	1	U	R.RHPYFYAPELLYYANK.Y
5253	144 - 159	682.3481	2044.0226	2044.0206	0.96	1	42	6.5e-005	1	U	R.RHPYFYAPELLYYANK.Y
5254	144 - 159	512.0131	2044.0231	2044.0206	1.23	1	39	0.00012	1	U	R.RHPYFYAPELLYYANK.Y
4733	145 - 159	944.9658	1887.9171	1887.9195	-1.29	0	52	5.8e-006	1	U	R.HPYFYAPELLYYANK.Y
4734	145 - 159	944.9659	1887.9172	1887.9195	-1.23	0	63	5e-007	1	U	R.HPYFYAPELLYYANK.Y
4735	145 - 159	944.9661	1887.9177	1887.9195	-0.96	0	52	5.9e-006	1	U	R.HPYFYAPELLYYANK.Y
4736	145 - 159	944.9662	1887.9178	1887.9195	-0.90	0	63	5e-007	1	U	R.HPYFYAPELLYYANK.Y
4737	145 - 159	944.9662	1887.9179	1887.9195	-0.84	0	52	6.6e-006	1	U	R.HPYFYAPELLYYANK.Y
4738	145 - 159	944.9663	1887.9181	1887.9195	-0.77	0	46	2.6e-005	1	U	R.HPYFYAPELLYYANK.Y
4739	145 - 159	630.3134	1887.9182	1887.9195	-0.69	0	45	3.5e-005	1	U	R.HPYFYAPELLYYANK.Y
4740	145 - 159	630.3134	1887.9184	1887.9195	-0.58	0	41	7.8e-005	1	U	R.HPYFYAPELLYYANK.Y
4741	145 - 159	630.3135	1887.9186	1887.9195	-0.48	0	41	7.2e-005	1	U	R.HPYFYAPELLYYANK.Y
4742	145 - 159	944.9667	1887.9188	1887.9195	-0.39	0	50	1.1e-005	1	U	R.HPYFYAPELLYYANK.Y
4743	145 - 159	630.3135	1887.9188	1887.9195	-0.39	0	50	9.3e-006	1	U	R.HPYFYAPELLYYANK.Y
4744	145 - 159	630.3136	1887.9190	1887.9195	-0.29	0	45	3.5e-005	1	U	R.HPYFYAPELLYYANK.Y
4745	145 - 159	630.3136	1887.9190	1887.9195	-0.29	0	48	1.4e-005	1	U	R.HPYFYAPELLYYANK.Y
4746	145 - 159	630.3137	1887.9192	1887.9195	-0.20	0	54	4.2e-006	1	U	R.HPYFYAPELLYYANK.Y
4747	145 - 159	630.3137	1887.9192	1887.9195	-0.20	0	51	8.6e-006	1	U	R.HPYFYAPELLYYANK.Y
4748	145 - 159	630.3137	1887.9193	1887.9195	-0.10	0	50	9e-006	1	U	R.HPYFYAPELLYYANK.Y
4749	145 - 159	630.3138	1887.9195	1887.9195	-0.0069	0	47	2.2e-005	1	U	R.HPYFYAPELLYYANK.Y
4750	145 - 159	630.3138	1887.9197	1887.9195	0.088	0	36	0.00024	1	U	R.HPYFYAPELLYYANK.Y
4751	145 - 159	630.3139	1887.9199	1887.9195	0.18	0	50	9.5e-006	1	U	R.HPYFYAPELLYYANK.Y
4752	145 - 159	630.3142	1887.9206	1887.9195	0.58	0	39	0.00012	1	U	R.HPYFYAPELLYYANK.Y
4753	145 - 159	944.9684	1887.9222	1887.9195	1.42	0	32	0.00067	1	U	R.HPYFYAPELLYYANK.Y
4265	160 - 173	874.3551	1746.6956	1746.6978	-1.21	0	56	2.7e-006	1	U	K.YNGVFQECCQAEDK.G
4266	160 - 173	874.3551	1746.6956	1746.6978	-1.21	0	64	4.4e-007	1	U	K.YNGVFQECCQAEDK.G
4267	160 - 173	874.3552	1746.6958	1746.6978	-1.15	0	59	1.1e-006	1	U	K.YNGVFQECCQAEDK.G
4268	160 - 173	874.3552	1746.6958	1746.6978	-1.15	0	67	1.9e-007	1	U	K.YNGVFQECCQAEDK.G
4269	160 - 173	874.3553	1746.6960	1746.6978	-1.00	0	64	4.3e-007	1	U	K.YNGVFQECCQAEDK.G
4270	160 - 173	874.3554	1746.6961	1746.6978	-0.93	0	66	2.8e-007	1	U	K.YNGVFQECCQAEDK.G
4271	160 - 173	874.3554	1746.6961	1746.6978	-0.93	0	65	2.9e-007	1	U	K.YNGVFQECCQAEDK.G
4272	160 - 173	874.3554	1746.6961	1746.6978	-0.93	0	64	4.3e-007	1	U	K.YNGVFQECCQAEDK.G
4273	160 - 173	874.3555	1746.6964	1746.6978	-0.79	0	54	3.8e-006	1	U	K.YNGVFQECCQAEDK.G
4274	160 - 173	874.3555	1746.6964	1746.6978	-0.79	0	50	9.1e-006	1	U	K.YNGVFQECCQAEDK.G
4275	160 - 173	874.3556	1746.6966	1746.6978	-0.65	0	56	2.7e-006	1	U	K.YNGVFQECCQAEDK.G
4276	160 - 173	874.3556	1746.6967	1746.6978	-0.58	0	62	6.9e-007	1	U	K.YNGVFQECCQAEDK.G
4277	160 - 173	874.3557	1746.6969	1746.6978	-0.52	0	33	0.00047	1	U	K.YNGVFQECCQAEDK.G
4278	160 - 173	874.3558	1746.6970	1746.6978	-0.45	0	42	6.2e-005	1	U	K.YNGVFQECCQAEDK.G
4279	160 - 173	874.3562	1746.6978	1746.6978	0.045	0	54	3.8e-006	1	U	K.YNGVFQECCQAEDK.G
5734	160 - 180	883.7243	2648.1511	2648.1557	-1.74	1	20	0.011	1	U	K.YNGVFQECCQAEDKGACLLPK.I + Glycation
5735	160 - 180	883.7255	2648.1546	2648.1557	-0.42	1	23	0.0053	1	U	K.YNGVFQECCQAEDKGACLLPK.I + Glycation
337	174 - 180	379.7145	757.4145	757.4156	-1.46	0	20	0.011	1	U	K.GACLLPK.I
338	174 - 180	379.7148	757.4151	757.4156	-0.67	0	23	0.0056	1	U	K.GACLLPK.I
339	174 - 180	379.7149	757.4152	757.4156	-0.59	0	23	0.0053	1	U	K.GACLLPK.I
340	174 - 180	379.7149	757.4152	757.4156	-0.59	0	23	0.0054	1	U	K.GACLLPK.I
341	174 - 180	379.7149	757.4152	757.4156	-0.51	0	34	0.00039	1	U	K.GACLLPK.I
342	174 - 180	379.7149	757.4153	757.4156	-0.40	0	22	0.0058	1	U	K.GACLLPK.I
343	174 - 180	379.7149	757.4153	757.4156	-0.40	0	23	0.0055	1	U	K.GACLLPK.I
344	174 - 180	379.7150	757.4154	757.4156	-0.32	0	21	0.008	1	U	K.GACLLPK.I
345	174 - 180	379.7150	757.4154	757.4156	-0.25	0	23	0.0054	1	U	K.GACLLPK.I
346	174 - 180	379.7150	757.4154	757.4156	-0.25	0	32	0.00062	1	U	K.GACLLPK.I
347	174 - 180	379.7150	757.4154	757.4156	-0.25	0	32	0.00062	1	U	K.GACLLPK.I
348	174 - 180	379.7150	757.4155	757.4156	-0.17	0	21	0.0077	1	U	K.GACLLPK.I
349	174 - 180	379.7150	757.4155	757.4156	-0.17	0	20	0.011	1	U	K.GACLLPK.I
350	174 - 180	379.7150	757.4155	757.4156	-0.17	0	23	0.0055	1	U	K.GACLLPK.I
351	174 - 180	379.7150	757.4155	757.4156	-0.17	0	34	0.00039	1	U	K.GACLLPK.I
352	174 - 180	379.7150	757.4155	757.4156	-0.17	0	32	0.00062	1	U	K.GACLLPK.I
353	174 - 180	379.7151	757.4156	757.4156	-0.087	0	23	0.0055	1	U	K.GACLLPK.I
354	174 - 180	379.7151	757.4156	757.4156	-0.087	0	32	0.00062	1	U	K.GACLLPK.I
355	174 - 180	379.7151	757.4156	757.4156	-0.0079	0	22	0.0058	1	U	K.GACLLPK.I
356	174 - 180	379.7151	757.4156	757.4156	-0.0079	0	32	0.00062	1	U	K.GACLLPK.I
357	174 - 180	379.7151	757.4157	757.4156	0.071	0	23	0.0054	1	U	K.GACLLPK.I

Query	Start - End	Observed	Mr(expt)	Mr(calc)	ppm	M	Score	Expect	Rank	U	Peptide
358	174 - 180	379.7152	757.4159	757.4156	0.31	0	21	0.0083	1	U	K.GACLLPK.I
359	174 - 180	379.7152	757.4159	757.4156	0.31	0	32	0.00062	1	U	K.GACLLPK.I
360	174 - 180	379.7152	757.4159	757.4156	0.31	0	34	0.00042	1	U	K.GACLLPK.I
361	174 - 180	379.7152	757.4159	757.4156	0.31	0	32	0.00062	1	U	K.GACLLPK.I
362	174 - 180	379.7152	757.4159	757.4156	0.31	0	32	0.00062	1	U	K.GACLLPK.I
363	174 - 180	379.7152	757.4159	757.4156	0.31	0	32	0.00062	1	U	K.GACLLPK.I
364	174 - 180	379.7153	757.4160	757.4156	0.47	0	23	0.0055	1	U	K.GACLLPK.I
3490	174 - 185	775.8987	1549.7829	1549.7844	-0.94	1	36	0.00026	1	U	K.GACLLPKIETMR.E + Glycation (K)
3491	174 - 185	517.6023	1549.7850	1549.7844	0.43	1	27	0.0021	1	U	K.GACLLPKIETMR.E + Glycation (K)
2354	199 - 208	453.2180	1356.6321	1356.6344	-1.70	1	17	0.019	1	U	R.CASIQKFGER.A + Glycation (K)
2355	199 - 208	453.2183	1356.6331	1356.6344	-0.90	1	24	0.0042	1	U	R.CASIQKFGER.A + Glycation (K)
2356	199 - 208	679.3238	1356.6331	1356.6344	-0.89	1	34	0.00039	1	U	R.CASIQKFGER.A + Glycation (K)
2357	199 - 208	679.3239	1356.6333	1356.6344	-0.80	1	34	0.00039	1	U	R.CASIQKFGER.A + Glycation (K)
2358	199 - 208	679.3240	1356.6334	1356.6344	-0.71	1	35	0.00034	1	U	R.CASIQKFGER.A + Glycation (K)
2359	199 - 208	453.2185	1356.6338	1356.6344	-0.41	1	16	0.025	1	U	R.CASIQKFGER.A + Glycation (K)
2360	199 - 208	453.2186	1356.6341	1356.6344	-0.22	1	21	0.0087	1	U	R.CASIQKFGER.A + Glycation (K)
1713	209 - 217	388.5518	1162.6335	1162.6346	-0.99	1	33	0.00053	1	U	R.ALKAWSVAR.L + Glycation (K)
1714	209 - 217	388.5518	1162.6335	1162.6346	-0.99	1	39	0.00013	1	U	R.ALKAWSVAR.L + Glycation (K)
1715	209 - 217	582.3241	1162.6336	1162.6346	-0.82	1	35	0.00032	1	U	R.ALKAWSVAR.L + Glycation (K)
1716	209 - 217	388.5519	1162.6339	1162.6346	-0.60	1	33	0.00053	1	U	R.ALKAWSVAR.L + Glycation (K)
1717	209 - 217	388.5519	1162.6339	1162.6346	-0.60	1	43	4.9e-005	1	U	R.ALKAWSVAR.L + Glycation (K)
1718	209 - 217	388.5520	1162.6341	1162.6346	-0.44	1	25	0.0032	1	U	R.ALKAWSVAR.L + Glycation (K)
1719	209 - 217	582.3243	1162.6341	1162.6346	-0.40	1	31	0.0008	1	U	R.ALKAWSVAR.L + Glycation (K)
1720	209 - 217	582.3243	1162.6341	1162.6346	-0.40	1	35	0.0003	1	U	R.ALKAWSVAR.L + Glycation (K)
1721	209 - 217	582.3244	1162.6342	1162.6346	-0.30	1	35	0.00031	1	U	R.ALKAWSVAR.L + Glycation (K)
1722	209 - 217	582.3245	1162.6344	1162.6346	-0.20	1	35	0.00029	1	U	R.ALKAWSVAR.L + Glycation (K)
1723	209 - 217	388.5521	1162.6345	1162.6346	-0.057	1	31	0.00073	1	U	R.ALKAWSVAR.L + Glycation (K)
201	212 - 217	345.1899	688.3653	688.3656	-0.55	0	17	0.022	1	U	K.AWSVAR.L
202	212 - 217	345.1899	688.3653	688.3656	-0.46	0	16	0.025	1	U	K.AWSVAR.L
203	212 - 217	345.1899	688.3653	688.3656	-0.46	0	13	0.046	1	U	K.AWSVAR.L
204	212 - 217	345.1899	688.3653	688.3656	-0.46	0	16	0.025	1	U	K.AWSVAR.L
205	212 - 217	345.1900	688.3654	688.3656	-0.37	0	15	0.035	1	U	K.AWSVAR.L
206	212 - 217	345.1900	688.3654	688.3656	-0.37	0	19	0.013	1	U	K.AWSVAR.L
207	212 - 217	345.1900	688.3654	688.3656	-0.37	0	16	0.024	1	U	K.AWSVAR.L
208	212 - 217	345.1900	688.3654	688.3656	-0.37	0	14	0.04	1	U	K.AWSVAR.L
209	212 - 217	345.1900	688.3654	688.3656	-0.29	0	13	0.049	1	U	K.AWSVAR.L
210	212 - 217	345.1900	688.3655	688.3656	-0.20	0	16	0.025	1	U	K.AWSVAR.L
211	212 - 217	345.1900	688.3655	688.3656	-0.20	0	15	0.029	1	U	K.AWSVAR.L
212	212 - 217	345.1900	688.3655	688.3656	-0.20	0	16	0.028	1	U	K.AWSVAR.L
213	212 - 217	345.1901	688.3656	688.3656	-0.11	0	16	0.028	1	U	K.AWSVAR.L
214	212 - 217	345.1901	688.3656	688.3656	-0.11	0	16	0.022	1	U	K.AWSVAR.L
216	212 - 217	345.1901	688.3656	688.3656	-0.11	0	13	0.048	1	U	K.AWSVAR.L
217	212 - 217	345.1901	688.3656	688.3656	-0.11	0	16	0.025	1	U	K.AWSVAR.L
218	212 - 217	345.1901	688.3656	688.3656	-0.026	0	14	0.04	1	U	K.AWSVAR.L
219	212 - 217	345.1901	688.3656	688.3656	-0.026	0	14	0.041	1	U	K.AWSVAR.L
220	212 - 217	345.1901	688.3656	688.3656	-0.026	0	17	0.018	1	U	K.AWSVAR.L
221	212 - 217	345.1901	688.3656	688.3656	-0.026	0	14	0.038	1	U	K.AWSVAR.L
222	212 - 217	345.1901	688.3656	688.3656	-0.026	0	15	0.03	1	U	K.AWSVAR.L
223	212 - 217	345.1901	688.3656	688.3656	-0.026	0	16	0.025	1	U	K.AWSVAR.L
224	212 - 217	345.1901	688.3657	688.3656	0.061	0	16	0.025	1	U	K.AWSVAR.L
225	212 - 217	345.1901	688.3657	688.3656	0.061	0	13	0.048	1	U	K.AWSVAR.L
226	212 - 217	345.1901	688.3657	688.3656	0.061	0	16	0.025	1	U	K.AWSVAR.L
228	212 - 217	345.1902	688.3658	688.3656	0.26	0	16	0.028	1	U	K.AWSVAR.L
229	212 - 217	345.1902	688.3658	688.3656	0.26	0	16	0.022	1	U	K.AWSVAR.L
230	212 - 217	345.1902	688.3659	688.3656	0.35	0	16	0.025	1	U	K.AWSVAR.L
231	212 - 217	345.1902	688.3659	688.3656	0.44	0	16	0.025	1	U	K.AWSVAR.L
232	212 - 217	345.1903	688.3660	688.3656	0.53	0	16	0.028	1	U	K.AWSVAR.L
233	212 - 217	345.1903	688.3660	688.3656	0.53	0	16	0.025	1	U	K.AWSVAR.L
234	212 - 217	345.1904	688.3662	688.3656	0.79	0	15	0.029	1	U	K.AWSVAR.L
235	212 - 217	345.1905	688.3665	688.3656	1.22	0	16	0.025	1	U	K.AWSVAR.L
1056	218 - 224	505.2821	1008.5496	1008.5491	0.49	1	13	0.045	1	U	R.LSQKFPK.A + Glycation (K)
1058	218 - 224	505.2829	1008.5513	1008.5491	2.14	1	14	0.037	1	U	R.LSQKFPK.A + Glycation (K)
740	225 - 232	461.7475	921.4805	921.4807	-0.26	0	43	5.4e-005	1	U	K.AEFVEVTK.L
741	225 - 232	461.7476	921.4806	921.4807	-0.19	0	42	5.9e-005	1	U	K.AEFVEVTK.L
742	225 - 232	461.7476	921.4806	921.4807	-0.12	0	43	5.5e-005	1	U	K.AEFVEVTK.L
743	225 - 232	461.7477	921.4807	921.4807	0.0054	0	30	0.00092	1	U	K.AEFVEVTK.L
744	225 - 232	461.7477	921.4808	921.4807	0.071	0	43	5.5e-005	1	U	K.AEFVEVTK.L
745	225 - 232	461.7478	921.4811	921.4807	0.40	0	43	5.3e-005	1	U	K.AEFVEVTK.L
746	225 - 232	461.7480	921.4815	921.4807	0.81	0	43	5.2e-005	1	U	K.AEFVEVTK.L
747	225 - 232	461.7481	921.4817	921.4807	1.00	0	43	5.4e-005	1	U	K.AEFVEVTK.L
748	225 - 232	461.7485	921.4824	921.4807	1.79	0	43	5.3e-005	1	U	K.AEFVEVTK.L
4666	225 - 239	927.9999	1853.9852	1853.9874	-1.17	1	81	8.7e-009	1	U	K.AEFVEVTKLVTDLTK.V + Glycation (K)
4667	225 - 239	928.0001	1853.9857	1853.9874	-0.91	1	81	8.4e-009	1	U	K.AEFVEVTKLVTDLTK.V + Glycation (K)
4668	225 - 239	928.0004	1853.9862	1853.9874	-0.64	1	72	6.3e-008	1	U	K.AEFVEVTKLVTDLTK.V + Glycation (K)
4669	225 - 239	619.0027	1853.9862	1853.9874	-0.61	1	41	7.7e-005	1	U	K.AEFVEVTKLVTDLTK.V + Glycation (K)
4670	225 - 239	928.0004	1853.9863	1853.9874	-0.58	1	62	6e-007	1	U	K.AEFVEVTKLVTDLTK.V + Glycation (K)
4671	225 - 239	928.0005	1853.9865	1853.9874	-0.45	1	72	6.8e-008	1	U	K.AEFVEVTKLVTDLTK.V + Glycation (K)
4672	225 - 239	619.0029	1853.9868	1853.9874	-0.32	1	51	8e-006	1	U	K.AEFVEVTKLVTDLTK.V + Glycation (K)
4673	225 - 239	619.0029	1853.9868	1853.9874	-0.32	1	50	8.9e-006	1	U	K.AEFVEVTKLVTDLTK.V + Glycation (K)
4674	225 - 239	619.0029	1853.9870	1853.9874	-0.23	1	51	8.2e-006	1	U	K.AEFVEVTKLVTDLTK.V + Glycation (K)
4675	225 - 239	619.0030	1853.9871	1853.9874	-0.13	1	42	5.8e-005	1	U	K.AEFVEVTKLVTDLTK.V + Glycation (K)
4676	225 - 239	619.0031	1853.9873	1853.9874	-0.032	1	48	1.5e-005	1	U	K.AEFVEVTKLVTDLTK.V + Glycation (K)
4677	225 - 239	619.0031	1853.9875	1853.9874	0.065	1	51	8e-006	1	U	K.AEFVEVTKLVTDLTK.V + Glycation (K)
4678	225 - 239	619.0032	1853.9877	1853.9874	0.16	1	47	2.2e-005	1	U	K.AEFVEVTKLVTDLTK.V + Glycation (K)
4679	225 - 239	619.0033	1853.9881	1853.9874	0.37	1	41	8.6e-005	1	U	K.AEFVEVTKLVTDLTK.V + Glycation (K)
4680	225 - 239	619.0034	1853.9883	1853.9874	0.47	1	41	8.7e-005	1	U	K.AEFVEVTKLVTDLTK.V + Glycation (K)
408	233 - 239	395.2393	788.4641	788.4644	-0.36	0	36	0.00026	1	U	K.LVTDLTK.V
409	233 - 239	395.2393	788.4641	788.4644	-0.36	0	36	0.00027	1	U	K.LVTDLTK.V
410	233 - 239	395.2393	788.4641	788.4644	-0.36	0	36	0.00027	1	U	K.LVTDLTK.V

Query	Start - End	Observed	Mr(expt)	Mr(calc)	ppm	M	Score	Expect	Rank	U	Peptide
411	233 - 239	395.2394	788.4642	788.4644	-0.21	0	36	0.00026	1	U	K.LVTDLTK.V
412	233 - 239	395.2394	788.4642	788.4644	-0.21	0	36	0.00026	1	U	K.LVTDLTK.V
413	233 - 239	395.2394	788.4642	788.4644	-0.21	0	36	0.00027	1	U	K.LVTDLTK.V
414	233 - 239	395.2394	788.4643	788.4644	-0.13	0	36	0.00026	1	U	K.LVTDLTK.V
415	233 - 239	395.2394	788.4643	788.4644	-0.13	0	36	0.00027	1	U	K.LVTDLTK.V
416	233 - 239	395.2394	788.4643	788.4644	-0.055	0	36	0.00026	1	U	K.LVTDLTK.V
417	233 - 239	395.2394	788.4643	788.4644	-0.055	0	36	0.00027	1	U	K.LVTDLTK.V
418	233 - 239	395.2394	788.4643	788.4644	-0.055	0	36	0.00026	1	U	K.LVTDLTK.V
419	233 - 239	395.2394	788.4643	788.4644	-0.055	0	36	0.00027	1	U	K.LVTDLTK.V
420	233 - 239	395.2394	788.4643	788.4644	-0.055	0	36	0.00027	1	U	K.LVTDLTK.V
421	233 - 239	395.2395	788.4644	788.4644	0.022	0	36	0.00027	1	U	K.LVTDLTK.V
422	233 - 239	395.2395	788.4644	788.4644	0.022	0	36	0.00027	1	U	K.LVTDLTK.V
423	233 - 239	395.2395	788.4644	788.4644	0.098	0	36	0.00026	1	U	K.LVTDLTK.V
424	233 - 239	395.2395	788.4644	788.4644	0.098	0	36	0.00027	1	U	K.LVTDLTK.V
425	233 - 239	395.2395	788.4644	788.4644	0.098	0	36	0.00027	1	U	K.LVTDLTK.V
426	233 - 239	395.2395	788.4645	788.4644	0.17	0	36	0.00027	1	U	K.LVTDLTK.V
427	233 - 239	395.2395	788.4645	788.4644	0.17	0	36	0.00027	1	U	K.LVTDLTK.V
428	233 - 239	395.2395	788.4645	788.4644	0.17	0	36	0.00026	1	U	K.LVTDLTK.V
429	233 - 239	395.2396	788.4646	788.4644	0.33	0	36	0.00027	1	U	K.LVTDLTK.V
430	233 - 239	395.2396	788.4646	788.4644	0.33	0	36	0.00027	1	U	K.LVTDLTK.V
431	233 - 239	395.2396	788.4647	788.4644	0.40	0	36	0.00026	1	U	K.LVTDLTK.V
432	233 - 239	395.2396	788.4647	788.4644	0.40	0	36	0.00027	1	U	K.LVTDLTK.V
433	233 - 239	395.2396	788.4647	788.4644	0.40	0	36	0.00027	1	U	K.LVTDLTK.V
434	233 - 239	395.2396	788.4647	788.4644	0.48	0	34	0.00044	1	U	K.LVTDLTK.V
435	233 - 239	395.2396	788.4647	788.4644	0.48	0	36	0.00027	1	U	K.LVTDLTK.V
436	233 - 239	395.2396	788.4647	788.4644	0.48	0	36	0.00027	1	U	K.LVTDLTK.V
437	233 - 239	395.2396	788.4647	788.4644	0.48	0	34	0.00042	1	U	K.LVTDLTK.V
438	233 - 239	395.2400	788.4654	788.4644	1.26	0	36	0.00027	1	U	K.LVTDLTK.V
439	233 - 239	395.2400	788.4655	788.4644	1.42	0	35	0.0003	1	U	K.LVTDLTK.V
2221	233 - 242	658.3768	1314.7390	1314.7395	-0.38	1	22	0.0062	1	U	K.LVTDLTKVHK.E + Glycation (K)
2222	233 - 242	439.2537	1314.7392	1314.7395	-0.19	1	21	0.0083	1	U	K.LVTDLTKVHK.E + Glycation (K)
2223	233 - 242	439.2537	1314.7393	1314.7395	-0.12	1	22	0.0069	1	U	K.LVTDLTKVHK.E + Glycation (K)
5437	240 - 256	569.7384	2274.9245	2274.9304	-2.58	1	36	0.00024	1	U	K.VHKECCHGDLLECADDR.A + Glycation (K)
5438	240 - 256	569.7388	2274.9260	2274.9304	-1.93	1	49	1.2e-005	1	U	K.VHKECCHGDLLECADDR.A + Glycation (K)
5439	240 - 256	759.3165	2274.9276	2274.9304	-1.23	1	146	2.7e-015	1	U	K.VHKECCHGDLLECADDR.A + Glycation (K)
5440	240 - 256	759.3167	2274.9283	2274.9304	-0.91	1	119	1.3e-012	1	U	K.VHKECCHGDLLECADDR.A + Glycation (K)
5441	240 - 256	759.3168	2274.9285	2274.9304	-0.83	1	140	1.1e-014	1	U	K.VHKECCHGDLLECADDR.A + Glycation (K)
5442	240 - 256	759.3168	2274.9287	2274.9304	-0.75	1	126	2.4e-013	1	U	K.VHKECCHGDLLECADDR.A + Glycation (K)
5443	240 - 256	569.7396	2274.9294	2274.9304	-0.44	1	36	0.00024	1	U	K.VHKECCHGDLLECADDR.A + Glycation (K)
5444	240 - 256	759.3171	2274.9294	2274.9304	-0.42	1	146	2.5e-015	1	U	K.VHKECCHGDLLECADDR.A + Glycation (K)
5445	240 - 256	455.9932	2274.9296	2274.9304	-0.35	1	23	0.0047	1	U	K.VHKECCHGDLLECADDR.A + Glycation (K)
5446	240 - 256	759.3171	2274.9296	2274.9304	-0.34	1	120	9.5e-013	1	U	K.VHKECCHGDLLECADDR.A + Glycation (K)
5447	240 - 256	569.7397	2274.9299	2274.9304	-0.21	1	24	0.0035	1	U	K.VHKECCHGDLLECADDR.A + Glycation (K)
5448	240 - 256	569.7397	2274.9299	2274.9304	-0.21	1	39	0.00012	1	U	K.VHKECCHGDLLECADDR.A + Glycation (K)
5449	240 - 256	455.9933	2274.9301	2274.9304	-0.13	1	30	0.00099	1	U	K.VHKECCHGDLLECADDR.A + Glycation (K)
5450	240 - 256	455.9934	2274.9308	2274.9304	0.20	1	28	0.0018	1	U	K.VHKECCHGDLLECADDR.A + Glycation (K)
5766	240 - 261	925.4102	2773.2088	2773.2106	-0.62	2	66	2.7e-007	1	U	K.VHKECCHGDLLECADDRADLAK.Y + Glycation
5767	240 - 261	555.6493	2773.2101	2773.2106	-0.18	2	14	0.037	1	U	K.VHKECCHGDLLECADDRADLAK.Y + Glycation
5768	240 - 261	694.3100	2773.2109	2773.2106	0.12	2	27	0.0019	1	U	K.VHKECCHGDLLECADDRADLAK.Y + Glycation
5770	240 - 261	555.6495	2773.2110	2773.2106	0.17	2	21	0.0088	1	U	K.VHKECCHGDLLECADDRADLAK.Y + Glycation
4301	243 - 256	875.3323	1748.6501	1748.6553	-2.93	0	108	1.4e-011	1	U	K.ECCHGDLLECADDR.A
4302	243 - 256	583.8910	1748.6511	1748.6553	-2.35	0	61	8.5e-007	1	U	K.ECCHGDLLECADDR.A
4303	243 - 256	875.3337	1748.6529	1748.6553	-1.33	0	114	3.7e-012	1	U	K.ECCHGDLLECADDR.A
4304	243 - 256	583.8916	1748.6530	1748.6553	-1.31	0	68	1.6e-007	1	U	K.ECCHGDLLECADDR.A
4305	243 - 256	875.3339	1748.6532	1748.6553	-1.20	0	101	8.8e-011	1	U	K.ECCHGDLLECADDR.A
4306	243 - 256	875.3339	1748.6533	1748.6553	-1.13	0	114	3.7e-012	1	U	K.ECCHGDLLECADDR.A
4307	243 - 256	875.3339	1748.6533	1748.6553	-1.13	0	109	1.3e-011	1	U	K.ECCHGDLLECADDR.A
4308	243 - 256	875.3341	1748.6535	1748.6553	-0.98	0	101	8.8e-011	1	U	K.ECCHGDLLECADDR.A
4309	243 - 256	875.3341	1748.6537	1748.6553	-0.91	0	99	1.3e-010	1	U	K.ECCHGDLLECADDR.A
4310	243 - 256	875.3342	1748.6538	1748.6553	-0.84	0	114	3.6e-012	1	U	K.ECCHGDLLECADDR.A
4311	243 - 256	875.3342	1748.6538	1748.6553	-0.84	0	100	8.9e-011	1	U	K.ECCHGDLLECADDR.A
4312	243 - 256	875.3342	1748.6539	1748.6553	-0.77	0	109	1.3e-011	1	U	K.ECCHGDLLECADDR.A
4313	243 - 256	875.3342	1748.6539	1748.6553	-0.77	0	114	3.6e-012	1	U	K.ECCHGDLLECADDR.A
4314	243 - 256	875.3342	1748.6539	1748.6553	-0.77	0	101	8.8e-011	1	U	K.ECCHGDLLECADDR.A
4315	243 - 256	875.3344	1748.6543	1748.6553	-0.57	0	114	3.7e-012	1	U	K.ECCHGDLLECADDR.A
4316	243 - 256	583.8921	1748.6544	1748.6553	-0.47	0	44	4.3e-005	1	U	K.ECCHGDLLECADDR.A
4317	243 - 256	875.3345	1748.6545	1748.6553	-0.43	0	108	1.4e-011	1	U	K.ECCHGDLLECADDR.A
4318	243 - 256	583.8922	1748.6546	1748.6553	-0.36	0	68	1.6e-007	1	U	K.ECCHGDLLECADDR.A
4319	243 - 256	875.3346	1748.6546	1748.6553	-0.36	0	95	3.1e-010	1	U	K.ECCHGDLLECADDR.A
4320	243 - 256	583.8922	1748.6548	1748.6553	-0.26	0	56	2.8e-006	1	U	K.ECCHGDLLECADDR.A
4321	243 - 256	583.8923	1748.6550	1748.6553	-0.16	0	68	1.5e-007	1	U	K.ECCHGDLLECADDR.A
4322	243 - 256	583.8923	1748.6550	1748.6553	-0.16	0	68	1.5e-007	1	U	K.ECCHGDLLECADDR.A
4323	243 - 256	583.8923	1748.6550	1748.6553	-0.16	0	62	5.8e-007	1	U	K.ECCHGDLLECADDR.A
4324	243 - 256	583.8923	1748.6550	1748.6553	-0.16	0	62	5.7e-007	1	U	K.ECCHGDLLECADDR.A
4325	243 - 256	583.8923	1748.6552	1748.6553	-0.055	0	67	1.8e-007	1	U	K.ECCHGDLLECADDR.A
4326	243 - 256	583.8924	1748.6554	1748.6553	0.065	0	68	1.5e-007	1	U	K.ECCHGDLLECADDR.A
4327	243 - 256	583.8924	1748.6554	1748.6553	0.065	0	68	1.6e-007	1	U	K.ECCHGDLLECADDR.A
4328	243 - 256	583.8924	1748.6554	1748.6553	0.065	0	68	1.5e-007	1	U	K.ECCHGDLLECADDR.A
4329	243 - 256	583.8925	1748.6556	1748.6553	0.17	0	81	8.3e-009	1	U	K.ECCHGDLLECADDR.A
4330	243 - 256	583.8925	1748.6557	1748.6553	0.27	0	81	8e-009	1	U	K.ECCHGDLLECADDR.A
4331	243 - 256	583.8925	1748.6557	1748.6553	0.27	0	81	8.2e-009	1	U	K.ECCHGDLLECADDR.A
4332	243 - 256	583.8926	1748.6559	1748.6553	0.37	0	61	7.4e-007	1	U	K.ECCHGDLLECADDR.A
4333	243 - 256	583.8926	1748.6559	1748.6553	0.37	0	68	1.5e-007	1	U	K.ECCHGDLLECADDR.A
4334	243 - 256	583.8926	1748.6561	1748.6553	0.48	0	68	1.5e-007	1	U	K.ECCHGDLLECADDR.A
4335	243 - 256	583.8933	1748.6581	1748.6553	1.63	0	63	5.5e-007	1	U	K.ECCHGDLLECADDR.A
4336	243 - 256	583.8943	1748.6610	1748.6553	3.31	0	68	1.5e-007	1	U	K.ECCHGDLLECADDR.A
5408	243 - 261	749.9842	2246.9307	2246.9354	-2.09	1	19	0.012	1	U	K.ECCHGDLLECADDRADLAK.Y
5409	243 - 261	1124.4730	2246.9315	2246.9354	-1.76	1	86	2.3e-009	1	U	K.ECCHGDLLECADDRADLAK.Y
5412	243 - 261	562.7404	2246.9326	2246.9354	-1.27	1	16	0.027	1	U	K.ECCHGDLLECADDRADLAK.Y

Query	Start – End	Observed	Mr(expt)	Mr(calc)	ppm	M	Score	Expect	Rank	U	Peptide
5413	243 – 261	562.7405	2246.9331	2246.9354	-1.06	1	14	0.041	1	U	K.ECCHGDLLECADDRADLAK.Y
5415	243 – 261	749.9851	2246.9335	2246.9354	-0.86	1	48	1.5e-005	1	U	K.ECCHGDLLECADDRADLAK.Y
5416	243 – 261	749.9852	2246.9339	2246.9354	-0.70	1	25	0.0029	1	U	K.ECCHGDLLECADDRADLAK.Y
5417	243 – 261	749.9852	2246.9339	2246.9354	-0.70	1	28	0.0015	1	U	K.ECCHGDLLECADDRADLAK.Y
5418	243 – 261	749.9852	2246.9339	2246.9354	-0.70	1	41	8.3e-005	1	U	K.ECCHGDLLECADDRADLAK.Y
5419	243 – 261	562.7408	2246.9340	2246.9354	-0.63	1	14	0.036	1	U	K.ECCHGDLLECADDRADLAK.Y
5420	243 – 261	1124.4744	2246.9342	2246.9354	-0.56	1	80	9.8e-009	1	U	K.ECCHGDLLECADDRADLAK.Y
5421	243 – 261	749.9854	2246.9342	2246.9354	-0.54	1	46	2.3e-005	1	U	K.ECCHGDLLECADDRADLAK.Y
5422	243 – 261	749.9854	2246.9344	2246.9354	-0.46	1	53	5.5e-006	1	U	K.ECCHGDLLECADDRADLAK.Y
5423	243 – 261	1124.4747	2246.9349	2246.9354	-0.24	1	75	3.1e-008	1	U	K.ECCHGDLLECADDRADLAK.Y
5424	243 – 261	749.9856	2246.9350	2246.9354	-0.21	1	51	7.9e-006	1	U	K.ECCHGDLLECADDRADLAK.Y
5425	243 – 261	562.7410	2246.9350	2246.9354	-0.19	1	20	0.0092	1	U	K.ECCHGDLLECADDRADLAK.Y
5426	243 – 261	749.9857	2246.9352	2246.9354	-0.13	1	48	1.5e-005	1	U	K.ECCHGDLLECADDRADLAK.Y
5427	243 – 261	562.7411	2246.9353	2246.9354	-0.082	1	18	0.014	1	U	K.ECCHGDLLECADDRADLAK.Y
5428	243 – 261	562.7411	2246.9353	2246.9354	-0.082	1	26	0.0024	1	U	K.ECCHGDLLECADDRADLAK.Y
5429	243 – 261	562.7411	2246.9355	2246.9354	0.025	1	23	0.0051	1	U	K.ECCHGDLLECADDRADLAK.Y
5430	243 – 261	1124.4751	2246.9356	2246.9354	0.093	1	79	1.2e-008	1	U	K.ECCHGDLLECADDRADLAK.Y
5431	243 – 261	562.7412	2246.9357	2246.9354	0.13	1	25	0.0035	1	U	K.ECCHGDLLECADDRADLAK.Y
5432	243 – 261	562.7415	2246.9370	2246.9354	0.68	1	18	0.015	1	U	K.ECCHGDLLECADDRADLAK.Y
5433	243 – 261	1124.4758	2246.9371	2246.9354	0.74	1	91	8.3e-010	1	U	K.ECCHGDLLECADDRADLAK.Y
6143	243 – 273	767.7277	3833.6022	3833.6125	-2.67	2	48	1.6e-005	1	U	K.ECCHGDLLECADDRADLAKYICDNQDTISSK.L +
6144	243 – 273	959.4089	3833.6067	3833.6125	-1.51	2	63	4.5e-007	1	U	K.ECCHGDLLECADDRADLAKYICDNQDTISSK.L +
6145	243 – 273	767.7289	3833.6080	3833.6125	-1.16	2	38	0.00015	1	U	K.ECCHGDLLECADDRADLAKYICDNQDTISSK.L +
6146	243 – 273	959.4097	3833.6098	3833.6125	-0.69	2	59	1.2e-006	1	U	K.ECCHGDLLECADDRADLAKYICDNQDTISSK.L +
6147	243 – 273	767.7293	3833.6102	3833.6125	-0.60	2	60	1e-006	1	U	K.ECCHGDLLECADDRADLAKYICDNQDTISSK.L +
5343	257 – 273	701.9959	2102.9659	2102.9678	-0.88	1	81	8.2e-009	1	U	R.ADLAKYICDNQDTISSK.L + Glycation (K)
5344	257 – 273	1052.4904	2102.9662	2102.9678	-0.76	1	57	1.8e-006	1	U	R.ADLAKYICDNQDTISSK.L + Glycation (K)
5345	257 – 273	701.9960	2102.9663	2102.9678	-0.71	1	94	3.6e-010	1	U	R.ADLAKYICDNQDTISSK.L + Glycation (K)
5346	257 – 273	701.9961	2102.9664	2102.9678	-0.63	1	61	7.3e-007	1	U	R.ADLAKYICDNQDTISSK.L + Glycation (K)
5347	257 – 273	701.9961	2102.9666	2102.9678	-0.54	1	95	3.4e-010	1	U	R.ADLAKYICDNQDTISSK.L + Glycation (K)
5348	257 – 273	701.9961	2102.9666	2102.9678	-0.54	1	89	1.2e-009	1	U	R.ADLAKYICDNQDTISSK.L + Glycation (K)
5349	257 – 273	701.9962	2102.9668	2102.9678	-0.44	1	60	1.1e-006	1	U	R.ADLAKYICDNQDTISSK.L + Glycation (K)
5350	257 – 273	1052.4907	2102.9669	2102.9678	-0.41	1	73	5.4e-008	1	U	R.ADLAKYICDNQDTISSK.L + Glycation (K)
5351	257 – 273	701.9963	2102.9670	2102.9678	-0.35	1	77	2e-008	1	U	R.ADLAKYICDNQDTISSK.L + Glycation (K)
5352	257 – 273	1052.4908	2102.9671	2102.9678	-0.30	1	62	6.3e-007	1	U	R.ADLAKYICDNQDTISSK.L + Glycation (K)
5353	257 – 273	701.9963	2102.9672	2102.9678	-0.27	1	41	7.4e-005	1	U	R.ADLAKYICDNQDTISSK.L + Glycation (K)
5354	257 – 273	701.9964	2102.9674	2102.9678	-0.18	1	60	1.1e-006	1	U	R.ADLAKYICDNQDTISSK.L + Glycation (K)
2773	262 – 273	722.3237	1442.6329	1442.6347	-1.28	0	60	1.1e-006	1	U	K.YICDNQDTISSK.L
2774	262 – 273	722.3238	1442.6331	1442.6347	-1.11	0	63	4.8e-007	1	U	K.YICDNQDTISSK.L
2775	262 – 273	722.3238	1442.6331	1442.6347	-1.11	0	59	1.2e-006	1	U	K.YICDNQDTISSK.L
2776	262 – 273	722.3239	1442.6333	1442.6347	-1.03	0	85	2.9e-009	1	U	K.YICDNQDTISSK.L
2777	262 – 273	722.3240	1442.6334	1442.6347	-0.94	0	68	1.6e-007	1	U	K.YICDNQDTISSK.L
2778	262 – 273	722.3240	1442.6335	1442.6347	-0.85	0	86	2.8e-009	1	U	K.YICDNQDTISSK.L
2779	262 – 273	722.3240	1442.6335	1442.6347	-0.85	0	86	2.7e-009	1	U	K.YICDNQDTISSK.L
2780	262 – 273	722.3241	1442.6336	1442.6347	-0.76	0	72	6.4e-008	1	U	K.YICDNQDTISSK.L
2781	262 – 273	722.3241	1442.6336	1442.6347	-0.76	0	86	2.7e-009	1	U	K.YICDNQDTISSK.L
2782	262 – 273	722.3241	1442.6336	1442.6347	-0.76	0	86	2.7e-009	1	U	K.YICDNQDTISSK.L
2783	262 – 273	722.3242	1442.6338	1442.6347	-0.68	0	86	2.8e-009	1	U	K.YICDNQDTISSK.L
2784	262 – 273	722.3243	1442.6340	1442.6347	-0.51	0	60	1e-006	1	U	K.YICDNQDTISSK.L
2785	262 – 273	722.3243	1442.6340	1442.6347	-0.51	0	55	2.9e-006	1	U	K.YICDNQDTISSK.L
2786	262 – 273	722.3243	1442.6341	1442.6347	-0.43	0	73	5.1e-008	1	U	K.YICDNQDTISSK.L
2787	262 – 273	722.3243	1442.6341	1442.6347	-0.43	0	85	2.8e-009	1	U	K.YICDNQDTISSK.L
2788	262 – 273	722.3243	1442.6341	1442.6347	-0.43	0	86	2.8e-009	1	U	K.YICDNQDTISSK.L
2789	262 – 273	722.3244	1442.6342	1442.6347	-0.35	0	85	2.9e-009	1	U	K.YICDNQDTISSK.L
2790	262 – 273	722.3244	1442.6342	1442.6347	-0.35	0	85	2.9e-009	1	U	K.YICDNQDTISSK.L
2791	262 – 273	722.3244	1442.6342	1442.6347	-0.35	0	86	2.7e-009	1	U	K.YICDNQDTISSK.L
2792	262 – 273	722.3245	1442.6344	1442.6347	-0.26	0	70	9.4e-008	1	U	K.YICDNQDTISSK.L
2793	262 – 273	722.3245	1442.6345	1442.6347	-0.18	0	55	3.3e-006	1	U	K.YICDNQDTISSK.L
2794	262 – 273	722.3245	1442.6345	1442.6347	-0.18	0	85	2.9e-009	1	U	K.YICDNQDTISSK.L
2795	262 – 273	722.3246	1442.6346	1442.6347	-0.098	0	72	6.6e-008	1	U	K.YICDNQDTISSK.L
2796	262 – 273	722.3247	1442.6349	1442.6347	0.082	0	72	5.7e-008	1	U	K.YICDNQDTISSK.L
2797	262 – 273	722.3247	1442.6349	1442.6347	0.082	0	54	3.8e-006	1	U	K.YICDNQDTISSK.L
2798	262 – 273	722.3248	1442.6350	1442.6347	0.16	0	63	4.7e-007	1	U	K.YICDNQDTISSK.L
2799	262 – 273	722.3248	1442.6351	1442.6347	0.25	0	55	3.1e-006	1	U	K.YICDNQDTISSK.L
2800	262 – 273	722.3254	1442.6362	1442.6347	1.01	0	86	2.7e-009	1	U	K.YICDNQDTISSK.L
2801	262 – 273	722.3254	1442.6363	1442.6347	1.09	0	86	2.8e-009	1	U	K.YICDNQDTISSK.L
4661	262 – 275	616.2961	1845.8664	1845.8666	-0.098	1	22	0.0067	1	U	K.YICDNQDTISSKLK.E + Glycation (K)
4662	262 – 275	616.2962	1845.8668	1845.8666	0.098	1	20	0.01	1	U	K.YICDNQDTISSKLK.E + Glycation (K)
3334	274 – 285	766.8932	1531.7719	1531.7738	-1.21	2	69	1.3e-007	1	U	K.LKECCDKPLLEK.S
3335	274 – 285	766.8934	1531.7722	1531.7738	-1.05	2	69	1.3e-007	1	U	K.LKECCDKPLLEK.S
3336	274 – 285	766.8934	1531.7723	1531.7738	-0.98	2	69	1.3e-007	1	U	K.LKECCDKPLLEK.S
3337	274 – 285	766.8935	1531.7724	1531.7738	-0.90	2	75	3.1e-008	1	U	K.LKECCDKPLLEK.S
3338	274 – 285	766.8935	1531.7724	1531.7738	-0.90	2	69	1.3e-007	1	U	K.LKECCDKPLLEK.S
3339	274 – 285	766.8936	1531.7725	1531.7738	-0.82	2	69	1.2e-007	1	U	K.LKECCDKPLLEK.S
3340	274 – 285	766.8936	1531.7725	1531.7738	-0.82	2	69	1.3e-007	1	U	K.LKECCDKPLLEK.S
3341	274 – 285	383.9504	1531.7727	1531.7738	-0.75	2	35	0.00031	1	U	K.LKECCDKPLLEK.S
3342	274 – 285	766.8936	1531.7727	1531.7738	-0.73	2	69	1.3e-007	1	U	K.LKECCDKPLLEK.S
3343	274 – 285	383.9505	1531.7728	1531.7738	-0.67	2	35	0.00029	1	U	K.LKECCDKPLLEK.S
3344	274 – 285	511.5982	1531.7729	1531.7738	-0.60	2	59	1.4e-006	1	U	K.LKECCDKPLLEK.S
3345	274 – 285	383.9505	1531.7729	1531.7738	-0.59	2	35	0.00031	1	U	K.LKECCDKPLLEK.S
3346	274 – 285	766.8937	1531.7729	1531.7738	-0.57	2	75	3.1e-008	1	U	K.LKECCDKPLLEK.S
3347	274 – 285	511.5983	1531.7730	1531.7738	-0.54	2	42	6.8e-005	1	U	K.LKECCDKPLLEK.S
3348	274 – 285	383.9505	1531.7730	1531.7738	-0.51	2	35	0.00033	1	U	K.LKECCDKPLLEK.S
3349	274 – 285	383.9505	1531.7730	1531.7738	-0.51	2	25	0.0031	1	U	K.LKECCDKPLLEK.S
3350	274 – 285	383.9505	1531.7730	1531.7738	-0.51	2	35	0.00033	1	U	K.LKECCDKPLLEK.S
3351	274 – 285	383.9505	1531.7730	1531.7738	-0.51	2	35	0.0003	1	U	K.LKECCDKPLLEK.S
3352	274 – 285	383.9506	1531.7731	1531.7738	-0.44	2	32	0.00064	1	U	K.LKECCDKPLLEK.S
3353	274 – 285	383.9506	1531.7731	1531.7738	-0.44	2	35	0.00031	1	U	K.LKECCDKPLLEK.S
3354	274 – 285	511.5983	1531.7732	1531.7738	-0.42	2	54	4.3e-006	1	U	K.LKECCDKPLLEK.S
3355	274 – 285	383.9506	1531.7733	1531.7738	-0.36	2	22	0.0056	1	U	K.LKECCDKPLLEK.S

Query	Start - End	Observed	Mr(expt)	Mr(calc)	ppm	M	Score	Expect	Rank	U	Peptide
3356	274 - 285	383.9506	1531.7733	1531.7738	-0.36	2	35	0.00031	1	U	K.LKECCDKPLLEK.S
3357	274 - 285	383.9506	1531.7733	1531.7738	-0.36	2	35	0.0003	1	U	K.LKECCDKPLLEK.S
3358	274 - 285	383.9506	1531.7733	1531.7738	-0.36	2	35	0.0003	1	U	K.LKECCDKPLLEK.S
3359	274 - 285	383.9506	1531.7734	1531.7738	-0.28	2	32	0.00069	1	U	K.LKECCDKPLLEK.S
3360	274 - 285	383.9506	1531.7734	1531.7738	-0.28	2	35	0.00031	1	U	K.LKECCDKPLLEK.S
3361	274 - 285	511.5985	1531.7735	1531.7738	-0.18	2	43	5.6e-005	1	U	K.LKECCDKPLLEK.S
3362	274 - 285	511.5985	1531.7737	1531.7738	-0.067	2	58	1.5e-006	1	U	K.LKECCDKPLLEK.S
3363	274 - 285	511.5985	1531.7737	1531.7738	-0.067	2	49	1.2e-005	1	U	K.LKECCDKPLLEK.S
3364	274 - 285	766.8942	1531.7738	1531.7738	-0.010	2	55	3.3e-006	1	U	K.LKECCDKPLLEK.S
3365	274 - 285	511.5985	1531.7738	1531.7738	-0.0078	2	63	5.2e-007	1	U	K.LKECCDKPLLEK.S
3366	274 - 285	383.9507	1531.7739	1531.7738	0.034	2	35	0.0003	1	U	K.LKECCDKPLLEK.S
3367	274 - 285	383.9507	1531.7739	1531.7738	0.034	2	35	0.00031	1	U	K.LKECCDKPLLEK.S
3369	274 - 285	511.5986	1531.7740	1531.7738	0.11	2	52	6.3e-006	1	U	K.LKECCDKPLLEK.S
3370	274 - 285	383.9508	1531.7740	1531.7738	0.14	2	25	0.003	1	U	K.LKECCDKPLLEK.S
3372	274 - 285	511.5986	1531.7741	1531.7738	0.17	2	58	1.6e-006	1	U	K.LKECCDKPLLEK.S
3373	274 - 285	511.5986	1531.7741	1531.7738	0.17	2	58	1.6e-006	1	U	K.LKECCDKPLLEK.S
3374	274 - 285	511.5986	1531.7741	1531.7738	0.17	2	58	1.6e-006	1	U	K.LKECCDKPLLEK.S
3375	274 - 285	383.9508	1531.7741	1531.7738	0.22	2	20	0.01	1	U	K.LKECCDKPLLEK.S
3376	274 - 285	383.9508	1531.7741	1531.7738	0.22	2	32	0.00064	1	U	K.LKECCDKPLLEK.S
3377	274 - 285	383.9508	1531.7741	1531.7738	0.22	2	32	0.0006	1	U	K.LKECCDKPLLEK.S
3378	274 - 285	383.9508	1531.7741	1531.7738	0.22	2	25	0.0032	1	U	K.LKECCDKPLLEK.S
3379	274 - 285	511.5987	1531.7742	1531.7738	0.23	2	42	6.7e-005	1	U	K.LKECCDKPLLEK.S
3380	274 - 285	511.5987	1531.7742	1531.7738	0.23	2	58	1.5e-006	1	U	K.LKECCDKPLLEK.S
3381	274 - 285	511.5988	1531.7745	1531.7738	0.48	2	52	6.2e-006	1	U	K.LKECCDKPLLEK.S
3382	274 - 285	511.5988	1531.7746	1531.7738	0.54	2	51	8e-006	1	U	K.LKECCDKPLLEK.S
3383	274 - 285	511.5989	1531.7747	1531.7738	0.60	2	58	1.6e-006	1	U	K.LKECCDKPLLEK.S
3384	274 - 285	511.5990	1531.7752	1531.7738	0.89	2	63	4.8e-007	1	U	K.LKECCDKPLLEK.S
3385	274 - 285	511.5990	1531.7752	1531.7738	0.89	2	51	8.1e-006	1	U	K.LKECCDKPLLEK.S
3386	274 - 285	511.5991	1531.7754	1531.7738	1.07	2	58	1.6e-006	1	U	K.LKECCDKPLLEK.S
4091	274 - 285	847.9191	1693.8236	1693.8266	-1.80	2	48	1.7e-005	1	U	K.LKECCDKPLLEK.S + Glycation (K)
4092	274 - 285	847.9194	1693.8243	1693.8266	-1.37	2	45	3.2e-005	1	U	K.LKECCDKPLLEK.S + Glycation (K)
4093	274 - 285	424.4636	1693.8255	1693.8266	-0.67	2	22	0.0069	1	U	K.LKECCDKPLLEK.S + Glycation (K)
4094	274 - 285	424.4636	1693.8255	1693.8266	-0.67	2	55	3.1e-006	1	U	K.LKECCDKPLLEK.S + Glycation (K)
4095	274 - 285	565.6158	1693.8255	1693.8266	-0.66	2	21	0.0073	1	U	K.LKECCDKPLLEK.S + Glycation (K)
4096	274 - 285	565.6158	1693.8255	1693.8266	-0.66	2	41	8.4e-005	1	U	K.LKECCDKPLLEK.S + Glycation (K)
4097	274 - 285	565.6158	1693.8257	1693.8266	-0.55	2	31	0.00081	1	U	K.LKECCDKPLLEK.S + Glycation (K)
4098	274 - 285	424.4638	1693.8259	1693.8266	-0.43	2	41	8.1e-005	1	U	K.LKECCDKPLLEK.S + Glycation (K)
4099	274 - 285	847.9203	1693.8260	1693.8266	-0.36	2	48	1.8e-005	1	U	K.LKECCDKPLLEK.S + Glycation (K)
4100	274 - 285	424.4638	1693.8261	1693.8266	-0.29	2	31	0.00079	1	U	K.LKECCDKPLLEK.S + Glycation (K)
4101	274 - 285	424.4638	1693.8261	1693.8266	-0.29	2	39	0.00011	1	U	K.LKECCDKPLLEK.S + Glycation (K)
4102	274 - 285	565.6160	1693.8263	1693.8266	-0.22	2	23	0.0045	1	U	K.LKECCDKPLLEK.S + Glycation (K)
4103	274 - 285	565.6160	1693.8263	1693.8266	-0.22	2	36	0.00023	1	U	K.LKECCDKPLLEK.S + Glycation (K)
4104	274 - 285	565.6161	1693.8266	1693.8266	-0.0047	2	25	0.0034	1	U	K.LKECCDKPLLEK.S + Glycation (K)
4105	274 - 285	424.4640	1693.8269	1693.8266	0.13	2	54	4.3e-006	1	U	K.LKECCDKPLLEK.S + Glycation (K)
4106	274 - 285	847.9208	1693.8270	1693.8266	0.22	2	45	3.2e-005	1	U	K.LKECCDKPLLEK.S + Glycation (K)
4107	274 - 285	424.4641	1693.8273	1693.8266	0.42	2	18	0.017	1	U	K.LKECCDKPLLEK.S + Glycation (K)
2060	276 - 285	646.3040	1290.5935	1290.5948	-1.01	1	46	2.6e-005	1	U	K.ECCDKPLLEK.S
2061	276 - 285	431.2053	1290.5940	1290.5948	-0.65	1	37	0.00021	1	U	K.ECCDKPLLEK.S
2062	276 - 285	646.3043	1290.5940	1290.5948	-0.64	1	54	3.5e-006	1	U	K.ECCDKPLLEK.S
2063	276 - 285	646.3044	1290.5942	1290.5948	-0.45	1	61	7.4e-007	1	U	K.ECCDKPLLEK.S
2064	276 - 285	646.3044	1290.5942	1290.5948	-0.45	1	61	7.3e-007	1	U	K.ECCDKPLLEK.S
2065	276 - 285	646.3045	1290.5944	1290.5948	-0.26	1	45	3.3e-005	1	U	K.ECCDKPLLEK.S
2066	276 - 285	646.3045	1290.5944	1290.5948	-0.26	1	42	5.8e-005	1	U	K.ECCDKPLLEK.S
2067	276 - 285	646.3045	1290.5944	1290.5948	-0.26	1	61	7.4e-007	1	U	K.ECCDKPLLEK.S
2068	276 - 285	431.2054	1290.5945	1290.5948	-0.23	1	37	0.00022	1	U	K.ECCDKPLLEK.S
2069	276 - 285	431.2055	1290.5946	1290.5948	-0.16	1	54	4.4e-006	1	U	K.ECCDKPLLEK.S
2070	276 - 285	431.2055	1290.5947	1290.5948	-0.067	1	31	0.00088	1	U	K.ECCDKPLLEK.S
2071	276 - 285	431.2055	1290.5948	1290.5948	0.0031	1	33	0.00053	1	U	K.ECCDKPLLEK.S
2072	276 - 285	431.2055	1290.5948	1290.5948	0.0031	1	54	4e-006	1	U	K.ECCDKPLLEK.S
2073	276 - 285	646.3047	1290.5948	1290.5948	0.031	1	53	5.4e-006	1	U	K.ECCDKPLLEK.S
2074	276 - 285	646.3047	1290.5948	1290.5948	0.031	1	61	7.3e-007	1	U	K.ECCDKPLLEK.S
2075	276 - 285	431.2056	1290.5950	1290.5948	0.14	1	33	0.00046	1	U	K.ECCDKPLLEK.S
2076	276 - 285	431.2056	1290.5950	1290.5948	0.14	1	34	0.00044	1	U	K.ECCDKPLLEK.S
2077	276 - 285	431.2056	1290.5950	1290.5948	0.14	1	60	1e-006	1	U	K.ECCDKPLLEK.S
2078	276 - 285	431.2056	1290.5950	1290.5948	0.14	1	54	3.9e-006	1	U	K.ECCDKPLLEK.S
2079	276 - 285	431.2056	1290.5950	1290.5948	0.14	1	54	4e-006	1	U	K.ECCDKPLLEK.S
2080	276 - 285	431.2057	1290.5952	1290.5948	0.28	1	41	7.6e-005	1	U	K.ECCDKPLLEK.S
2081	276 - 285	431.2057	1290.5952	1290.5948	0.35	1	54	4e-006	1	U	K.ECCDKPLLEK.S
2082	276 - 285	431.2058	1290.5955	1290.5948	0.56	1	34	0.00044	1	U	K.ECCDKPLLEK.S
2083	276 - 285	646.3051	1290.5957	1290.5948	0.68	1	57	2.1e-006	1	U	K.ECCDKPLLEK.S
2811	276 - 285	727.3304	1452.6463	1452.6476	-0.89	1	41	7.1e-005	1	U	K.ECCDKPLLEK.S + Glycation (K)
1337	286 - 294	358.1740	1071.5001	1071.5019	-1.65	0	30	0.00093	1	U	K.SHCIAEVEK.D
1338	286 - 294	358.1740	1071.5002	1071.5019	-1.56	0	34	0.00038	1	U	K.SHCIAEVEK.D
1339	286 - 294	358.1743	1071.5010	1071.5019	-0.78	0	42	7e-005	1	U	K.SHCIAEVEK.D
1340	286 - 294	536.7578	1071.5011	1071.5019	-0.76	0	15	0.029	1	U	K.SHCIAEVEK.D
1341	286 - 294	536.7579	1071.5012	1071.5019	-0.64	0	24	0.0044	1	U	K.SHCIAEVEK.D
1342	286 - 294	358.1744	1071.5012	1071.5019	-0.61	0	32	0.00057	1	U	K.SHCIAEVEK.D
1343	286 - 294	358.1744	1071.5012	1071.5019	-0.61	0	37	0.00022	1	U	K.SHCIAEVEK.D
1344	286 - 294	536.7579	1071.5013	1071.5019	-0.53	0	32	0.00062	1	U	K.SHCIAEVEK.D
1345	286 - 294	358.1744	1071.5014	1071.5019	-0.44	0	34	0.00037	1	U	K.SHCIAEVEK.D
1346	286 - 294	358.1744	1071.5014	1071.5019	-0.44	0	31	0.00076	1	U	K.SHCIAEVEK.D
1347	286 - 294	358.1744	1071.5015	1071.5019	-0.36	0	39	0.00013	1	U	K.SHCIAEVEK.D
1348	286 - 294	358.1744	1071.5015	1071.5019	-0.36	0	42	6.4e-005	1	U	K.SHCIAEVEK.D
1349	286 - 294	358.1744	1071.5015	1071.5019	-0.36	0	42	6.2e-005	1	U	K.SHCIAEVEK.D
1350	286 - 294	358.1744	1071.5015	1071.5019	-0.36	0	34	0.00036	1	U	K.SHCIAEVEK.D
1351	286 - 294	358.1744	1071.5015	1071.5019	-0.36	0	39	0.00013	1	U	K.SHCIAEVEK.D
1352	286 - 294	358.1744	1071.5015	1071.5019	-0.36	0	39	0.00013	1	U	K.SHCIAEVEK.D
1353	286 - 294	536.7581	1071.5016	1071.5019	-0.29	0	42	6.3e-005	1	U	K.SHCIAEVEK.D
1354	286 - 294	536.7581	1071.5016	1071.5019	-0.29	0	28	0.0015	1	U	K.SHCIAEVEK.D
1355	286 - 294	358.1745	1071.5016	1071.5019	-0.28	0	39	0.00012	1	U	K.SHCIAEVEK.D

Query	Start – End	Observed	Mr(expt)	Mr(calc)	ppm	M	Score	Expect	Rank	U	Peptide
1356	286 – 294	358.1745	1071.5016	1071.5019	-0.28	0	37	0.00022	1	U	K.SHCIAEVEK.D
1357	286 – 294	358.1745	1071.5017	1071.5019	-0.19	0	31	0.00084	1	U	K.SHCIAEVEK.D
1358	286 – 294	358.1745	1071.5017	1071.5019	-0.19	0	39	0.00013	1	U	K.SHCIAEVEK.D
1359	286 – 294	536.7581	1071.5017	1071.5019	-0.18	0	42	6.9e-005	1	U	K.SHCIAEVEK.D
1360	286 – 294	536.7581	1071.5017	1071.5019	-0.18	0	48	1.7e-005	1	U	K.SHCIAEVEK.D
1361	286 – 294	536.7581	1071.5017	1071.5019	-0.18	0	48	1.7e-005	1	U	K.SHCIAEVEK.D
1362	286 – 294	358.1745	1071.5018	1071.5019	-0.11	0	42	6.4e-005	1	U	K.SHCIAEVEK.D
1363	286 – 294	358.1745	1071.5018	1071.5019	-0.11	0	39	0.00012	1	U	K.SHCIAEVEK.D
1364	286 – 294	358.1745	1071.5018	1071.5019	-0.11	0	37	0.00022	1	U	K.SHCIAEVEK.D
1365	286 – 294	536.7582	1071.5018	1071.5019	-0.064	0	39	0.00012	1	U	K.SHCIAEVEK.D
1366	286 – 294	536.7582	1071.5019	1071.5019	0.048	0	53	5.4e-006	1	U	K.SHCIAEVEK.D
1367	286 – 294	536.7583	1071.5020	1071.5019	0.16	0	42	6e-005	1	U	K.SHCIAEVEK.D
1368	286 – 294	536.7583	1071.5020	1071.5019	0.16	0	49	1.3e-005	1	U	K.SHCIAEVEK.D
1369	286 – 294	536.7583	1071.5020	1071.5019	0.16	0	48	1.7e-005	1	U	K.SHCIAEVEK.D
1370	286 – 294	358.1747	1071.5022	1071.5019	0.31	0	36	0.00022	1	U	K.SHCIAEVEK.D
1371	286 – 294	536.7584	1071.5023	1071.5019	0.38	0	39	0.00012	1	U	K.SHCIAEVEK.D
1372	286 – 294	536.7585	1071.5024	1071.5019	0.50	0	27	0.002	1	U	K.SHCIAEVEK.D
5845	286 – 312	1003.8211	3008.4415	3008.4437	-0.72	1	70	1.1e-007	1	U	K.SHCIAEVEKDAIPENLPPLTADFAEDK.D
5846	286 – 312	753.1181	3008.4433	3008.4437	-0.13	1	54	4.3e-006	1	U	K.SHCIAEVEKDAIPENLPPLTADFAEDK.D
5847	286 – 312	753.1181	3008.4433	3008.4437	-0.13	1	55	3.3e-006	1	U	K.SHCIAEVEKDAIPENLPPLTADFAEDK.D
5848	286 – 312	1003.8218	3008.4435	3008.4437	-0.056	1	64	3.9e-007	1	U	K.SHCIAEVEKDAIPENLPPLTADFAEDK.D
5849	286 – 312	753.1182	3008.4435	3008.4437	-0.048	1	54	3.7e-006	1	U	K.SHCIAEVEKDAIPENLPPLTADFAEDK.D
5964	286 – 316	878.6708	3510.6543	3510.6647	-2.96	2	78	1.6e-008	1	U	K.SHCIAEVEKDAIPENLPPLTADFAEDKDVCK.N
5965	286 – 316	703.1383	3510.6552	3510.6647	-2.70	2	82	6.6e-009	1	U	K.SHCIAEVEKDAIPENLPPLTADFAEDKDVCK.N
5966	286 – 316	878.6717	3510.6579	3510.6647	-1.93	2	74	3.6e-008	1	U	K.SHCIAEVEKDAIPENLPPLTADFAEDKDVCK.N
5967	286 – 316	703.1393	3510.6602	3510.6647	-1.26	2	66	2.5e-007	1	U	K.SHCIAEVEKDAIPENLPPLTADFAEDKDVCK.N
5968	286 – 316	878.6724	3510.6606	3510.6647	-1.16	2	81	8.6e-009	1	U	K.SHCIAEVEKDAIPENLPPLTADFAEDKDVCK.N
5969	286 – 316	878.6726	3510.6613	3510.6647	-0.95	2	75	3.4e-008	1	U	K.SHCIAEVEKDAIPENLPPLTADFAEDKDVCK.N
5970	286 – 316	703.1396	3510.6619	3510.6647	-0.79	2	27	0.0021	1	U	K.SHCIAEVEKDAIPENLPPLTADFAEDKDVCK.N
5971	286 – 316	703.1397	3510.6622	3510.6647	-0.71	2	66	2.6e-007	1	U	K.SHCIAEVEKDAIPENLPPLTADFAEDKDVCK.N
5972	286 – 316	878.6729	3510.6623	3510.6647	-0.67	2	77	2e-008	1	U	K.SHCIAEVEKDAIPENLPPLTADFAEDKDVCK.N
5973	286 – 316	703.1398	3510.6628	3510.6647	-0.54	2	45	3.3e-005	1	U	K.SHCIAEVEKDAIPENLPPLTADFAEDKDVCK.N
5974	286 – 316	878.6730	3510.6628	3510.6647	-0.54	2	79	1.4e-008	1	U	K.SHCIAEVEKDAIPENLPPLTADFAEDKDVCK.N
5975	286 – 316	878.6730	3510.6628	3510.6647	-0.54	2	78	1.5e-008	1	U	K.SHCIAEVEKDAIPENLPPLTADFAEDKDVCK.N
5976	286 – 316	878.6730	3510.6628	3510.6647	-0.54	2	76	2.3e-008	1	U	K.SHCIAEVEKDAIPENLPPLTADFAEDKDVCK.N
5977	286 – 316	878.6732	3510.6635	3510.6647	-0.32	2	76	2.7e-008	1	U	K.SHCIAEVEKDAIPENLPPLTADFAEDKDVCK.N
5978	286 – 316	878.6732	3510.6638	3510.6647	-0.25	2	83	4.5e-009	1	U	K.SHCIAEVEKDAIPENLPPLTADFAEDKDVCK.N
5979	286 – 316	878.6733	3510.6640	3510.6647	-0.18	2	68	1.5e-007	1	U	K.SHCIAEVEKDAIPENLPPLTADFAEDKDVCK.N
5980	286 – 316	878.6733	3510.6640	3510.6647	-0.18	2	78	1.5e-008	1	U	K.SHCIAEVEKDAIPENLPPLTADFAEDKDVCK.N
5981	286 – 316	878.6733	3510.6643	3510.6647	-0.11	2	70	9.3e-008	1	U	K.SHCIAEVEKDAIPENLPPLTADFAEDKDVCK.N
5982	286 – 316	703.1401	3510.6643	3510.6647	-0.096	2	78	1.6e-008	1	U	K.SHCIAEVEKDAIPENLPPLTADFAEDKDVCK.N
5983	286 – 316	703.1401	3510.6643	3510.6647	-0.096	2	90	1e-009	1	U	K.SHCIAEVEKDAIPENLPPLTADFAEDKDVCK.N
5984	286 – 316	703.1401	3510.6643	3510.6647	-0.096	2	92	6e-010	1	U	K.SHCIAEVEKDAIPENLPPLTADFAEDKDVCK.N
5985	286 – 316	1171.2288	3510.6645	3510.6647	-0.059	2	79	1.2e-008	1	U	K.SHCIAEVEKDAIPENLPPLTADFAEDKDVCK.N
5986	286 – 316	703.1403	3510.6649	3510.6647	0.075	2	68	1.5e-007	1	U	K.SHCIAEVEKDAIPENLPPLTADFAEDKDVCK.N
5987	286 – 316	1171.2290	3510.6652	3510.6647	0.15	2	77	2.2e-008	1	U	K.SHCIAEVEKDAIPENLPPLTADFAEDKDVCK.N
5988	286 – 316	1171.2290	3510.6652	3510.6647	0.15	2	77	1.9e-008	1	U	K.SHCIAEVEKDAIPENLPPLTADFAEDKDVCK.N
5989	286 – 316	703.1404	3510.6655	3510.6647	0.25	2	75	3e-008	1	U	K.SHCIAEVEKDAIPENLPPLTADFAEDKDVCK.N
5990	286 – 316	1171.2293	3510.6659	3510.6647	0.36	2	83	4.7e-009	1	U	K.SHCIAEVEKDAIPENLPPLTADFAEDKDVCK.N
5991	286 – 316	703.1405	3510.6661	3510.6647	0.42	2	76	2.3e-008	1	U	K.SHCIAEVEKDAIPENLPPLTADFAEDKDVCK.N
5992	286 – 316	1171.2294	3510.6663	3510.6647	0.46	2	87	1.8e-009	1	U	K.SHCIAEVEKDAIPENLPPLTADFAEDKDVCK.N
5993	286 – 316	878.6740	3510.6669	3510.6647	0.65	2	67	1.9e-007	1	U	K.SHCIAEVEKDAIPENLPPLTADFAEDKDVCK.N
5994	286 – 316	1171.2296	3510.6670	3510.6647	0.67	2	77	2.1e-008	1	U	K.SHCIAEVEKDAIPENLPPLTADFAEDKDVCK.N
5995	286 – 316	878.6741	3510.6672	3510.6647	0.72	2	85	3.5e-009	1	U	K.SHCIAEVEKDAIPENLPPLTADFAEDKDVCK.N
5996	286 – 316	586.1185	3510.6675	3510.6647	0.82	2	89	1.3e-009	1	U	K.SHCIAEVEKDAIPENLPPLTADFAEDKDVCK.N
5997	286 – 316	703.1408	3510.6677	3510.6647	0.86	2	90	1.1e-009	1	U	K.SHCIAEVEKDAIPENLPPLTADFAEDKDVCK.N
5998	286 – 316	703.1409	3510.6680	3510.6647	0.94	2	90	1.1e-009	1	U	K.SHCIAEVEKDAIPENLPPLTADFAEDKDVCK.N
5999	286 – 316	878.6744	3510.6684	3510.6647	1.07	2	81	7.4e-009	1	U	K.SHCIAEVEKDAIPENLPPLTADFAEDKDVCK.N
6000	286 – 316	1171.2302	3510.6688	3510.6647	1.19	2	75	3.4e-008	1	U	K.SHCIAEVEKDAIPENLPPLTADFAEDKDVCK.N
6001	286 – 316	1171.2304	3510.6692	3510.6647	1.30	2	77	1.8e-008	1	U	K.SHCIAEVEKDAIPENLPPLTADFAEDKDVCK.N
6086	286 – 316	919.1853	3672.7121	3672.7175	-1.47	2	69	1.4e-007	1	U	K.SHCIAEVEKDAIPENLPPLTADFAEDKDVCK.N +
6087	286 – 316	919.1853	3672.7121	3672.7175	-1.47	2	62	6.6e-007	1	U	K.SHCIAEVEKDAIPENLPPLTADFAEDKDVCK.N +
6088	286 – 316	735.5499	3672.7133	3672.7175	-1.15	2	52	7e-006	1	U	K.SHCIAEVEKDAIPENLPPLTADFAEDKDVCK.N +
6089	286 – 316	919.1858	3672.7141	3672.7175	-0.93	2	49	1.3e-005	1	U	K.SHCIAEVEKDAIPENLPPLTADFAEDKDVCK.N +
6090	286 – 316	735.5502	3672.7148	3672.7175	-0.74	2	38	0.00014	1	U	K.SHCIAEVEKDAIPENLPPLTADFAEDKDVCK.N +
6091	286 – 316	919.1860	3672.7148	3672.7175	-0.74	2	61	8.2e-007	1	U	K.SHCIAEVEKDAIPENLPPLTADFAEDKDVCK.N +
6092	286 – 316	919.1860	3672.7148	3672.7175	-0.74	2	58	1.6e-006	1	U	K.SHCIAEVEKDAIPENLPPLTADFAEDKDVCK.N +
6093	286 – 316	735.5503	3672.7151	3672.7175	-0.66	2	52	7.1e-006	1	U	K.SHCIAEVEKDAIPENLPPLTADFAEDKDVCK.N +
6094	286 – 316	735.5503	3672.7154	3672.7175	-0.58	2	33	0.00048	1	U	K.SHCIAEVEKDAIPENLPPLTADFAEDKDVCK.N +
6095	286 – 316	735.5504	3672.7157	3672.7175	-0.48	2	45	3e-005	1	U	K.SHCIAEVEKDAIPENLPPLTADFAEDKDVCK.N +
6096	286 – 316	735.5505	3672.7160	3672.7175	-0.40	2	38	0.00014	1	U	K.SHCIAEVEKDAIPENLPPLTADFAEDKDVCK.N +
6097	286 – 316	919.1863	3672.7163	3672.7175	-0.33	2	58	1.7e-006	1	U	K.SHCIAEVEKDAIPENLPPLTADFAEDKDVCK.N +
6098	286 – 316	735.5506	3672.7166	3672.7175	-0.24	2	41	7.6e-005	1	U	K.SHCIAEVEKDAIPENLPPLTADFAEDKDVCK.N +
6099	286 – 316	919.1866	3672.7172	3672.7175	-0.073	2	52	6.5e-006	1	U	K.SHCIAEVEKDAIPENLPPLTADFAEDKDVCK.N +
6100	286 – 316	1225.2467	3672.7183	3672.7175	0.21	2	48	1.6e-005	1	U	K.SHCIAEVEKDAIPENLPPLTADFAEDKDVCK.N +
6101	286 – 316	1225.2473	3672.7201	3672.7175	0.71	2	49	1.2e-005	1	U	K.SHCIAEVEKDAIPENLPPLTADFAEDKDVCK.N +
6102	286 – 316	1225.2474	3672.7205	3672.7175	0.82	2	58	1.8e-006	1	U	K.SHCIAEVEKDAIPENLPPLTADFAEDKDVCK.N +
6103	286 – 316	1225.2479	3672.7219	3672.7175	1.21	2	49	1.2e-005	1	U	K.SHCIAEVEKDAIPENLPPLTADFAEDKDVCK.N +
6104	286 – 316	1225.2513	3672.7322	3672.7175	4.00	2	57	1.9e-006	1	U	K.SHCIAEVEKDAIPENLPPLTADFAEDKDVCK.N +
6149	286 – 316	959.6975	3834.7607	3834.7703	-2.51	2	45	3.3e-005	1	U	K.SHCIAEVEKDAIPENLPPLTADFAEDKDVCK.N +
6150	286 – 316	767.9598	3834.7625	3834.7703	-2.03	2	13	0.046	1	U	K.SHCIAEVEKDAIPENLPPLTADFAEDKDVCK.N +
6151	286 – 316	767.9603	3834.7653	3834.7703	-1.31	2	17	0.02	1	U	K.SHCIAEVEKDAIPENLPPLTADFAEDKDVCK.N +
6152	286 – 316	959.7011	3834.7753	3834.7703	1.31	2	43	4.7e-005	1	U	K.SHCIAEVEKDAIPENLPPLTADFAEDKDVCK.N +
6175	286 – 322	735.3473	4406.0401	4406.0570	-3.83	3	29	0.0012	1	U	K.SHCIAEVEKDAIPENLPPLTADFAEDKDVCKNYQEA
6176	286 – 322	735.3478	4406.0434	4406.0570	-3.08	3	27	0.0021	1	U	K.SHCIAEVEKDAIPENLPPLTADFAEDKDVCKNYQEA
6177	286 – 322	882.2167	4406.0473	4406.0570	-2.19	3	38	0.00015	1	U	K.SHCIAEVEKDAIPENLPPLTADFAEDKDVCKNYQEA
6178	286 – 322	735.3494	4406.0526	4406.0570	-1.00	3	35	0.00029	1	U	K.SHCIAEVEKDAIPENLPPLTADFAEDKDVCKNYQEA
6179	286 – 322	882.2186	4406.0565	4406.0570	-0.11	3	48	1.6e-005	1	U	K.SHCIAEVEKDAIPENLPPLTADFAEDKDVCKNYQEA
6180	286 – 322	882.2186	4406.0565	4406.0570	-0.11	3	31	0.00087	1	U	K.SHCIAEVEKDAIPENLPPLTADFAEDKDVCKNYQEA

Query	Start - End	Observed	Mr(expt)	Mr(calc)	ppm	M	Score	Expect	Rank	U	Peptide
6181	286 - 322	882.2188	4406.0574	4406.0570	0.093	3	29	0.0013	1	U	K.SHCIAEVEKDAIPENLPPLTADFAEDKDVCKNYQE
6182	286 - 322	1102.5219	4406.0583	4406.0570	0.30	3	42	5.7e-005	1	U	K.SHCIAEVEKDAIPENLPPLTADFAEDKDVCKNYQE
6183	286 - 322	882.2198	4406.0626	4406.0570	1.27	3	35	0.0003	1	U	K.SHCIAEVEKDAIPENLPPLTADFAEDKDVCKNYQE
6184	286 - 322	882.2205	4406.0662	4406.0570	2.10	3	53	4.9e-006	1	U	K.SHCIAEVEKDAIPENLPPLTADFAEDKDVCKNYQE
6185	286 - 322	1102.5248	4406.0700	4406.0570	2.96	3	43	5e-005	1	U	K.SHCIAEVEKDAIPENLPPLTADFAEDKDVCKNYQE
5001	295 - 312	978.4824	1954.9503	1954.9524	-1.06	0	57	2e-006	1	U	K.DAIPENLPPLTADFAEDK.D
5002	295 - 312	978.4828	1954.9511	1954.9524	-0.62	0	55	3.2e-006	1	U	K.DAIPENLPPLTADFAEDK.D
5522	295 - 316	615.2993	2457.1682	2457.1733	-2.10	1	38	0.00017	1	U	K.DAIPENLPPLTADFAEDKDVCK.N
5523	295 - 316	820.0638	2457.1697	2457.1733	-1.48	1	63	5.4e-007	1	U	K.DAIPENLPPLTADFAEDKDVCK.N
5524	295 - 316	820.0640	2457.1701	2457.1733	-1.34	1	57	2.2e-006	1	U	K.DAIPENLPPLTADFAEDKDVCK.N
5525	295 - 316	820.0642	2457.1708	2457.1733	-1.03	1	70	1.1e-007	1	U	K.DAIPENLPPLTADFAEDKDVCK.N
5526	295 - 316	820.0643	2457.1710	2457.1733	-0.96	1	61	7.9e-007	1	U	K.DAIPENLPPLTADFAEDKDVCK.N
5527	295 - 316	820.0643	2457.1710	2457.1733	-0.96	1	71	7.5e-008	1	U	K.DAIPENLPPLTADFAEDKDVCK.N
5528	295 - 316	820.0643	2457.1712	2457.1733	-0.89	1	64	4.4e-007	1	U	K.DAIPENLPPLTADFAEDKDVCK.N
5529	295 - 316	820.0643	2457.1712	2457.1733	-0.89	1	54	3.6e-006	1	U	K.DAIPENLPPLTADFAEDKDVCK.N
5530	295 - 316	820.0644	2457.1715	2457.1733	-0.74	1	64	4.1e-007	1	U	K.DAIPENLPPLTADFAEDKDVCK.N
5531	295 - 316	820.0644	2457.1715	2457.1733	-0.74	1	63	5.4e-007	1	U	K.DAIPENLPPLTADFAEDKDVCK.N
5532	295 - 316	820.0644	2457.1715	2457.1733	-0.74	1	73	5e-008	1	U	K.DAIPENLPPLTADFAEDKDVCK.N
5533	295 - 316	820.0644	2457.1715	2457.1733	-0.74	1	74	4.1e-008	1	U	K.DAIPENLPPLTADFAEDKDVCK.N
5534	295 - 316	820.0644	2457.1715	2457.1733	-0.74	1	83	5.2e-009	1	U	K.DAIPENLPPLTADFAEDKDVCK.N
5535	295 - 316	615.3002	2457.1716	2457.1733	-0.72	1	42	6.8e-005	1	U	K.DAIPENLPPLTADFAEDKDVCK.N
5536	295 - 316	820.0645	2457.1717	2457.1733	-0.67	1	62	6.7e-007	1	U	K.DAIPENLPPLTADFAEDKDVCK.N
5537	295 - 316	1229.5933	2457.1720	2457.1733	-0.56	1	80	1.1e-008	1	U	K.DAIPENLPPLTADFAEDKDVCK.N
5538	295 - 316	820.0646	2457.1721	2457.1733	-0.51	1	72	5.9e-008	1	U	K.DAIPENLPPLTADFAEDKDVCK.N
5539	295 - 316	820.0647	2457.1723	2457.1733	-0.43	1	71	7.8e-008	1	U	K.DAIPENLPPLTADFAEDKDVCK.N
5540	295 - 316	820.0648	2457.1725	2457.1733	-0.36	1	71	7.9e-008	1	U	K.DAIPENLPPLTADFAEDKDVCK.N
5541	295 - 316	820.0650	2457.1732	2457.1733	-0.068	1	74	4.2e-008	1	U	K.DAIPENLPPLTADFAEDKDVCK.N
5542	295 - 316	1229.5939	2457.1732	2457.1733	-0.062	1	94	4.1e-010	1	U	K.DAIPENLPPLTADFAEDKDVCK.N
5543	295 - 316	1229.5940	2457.1734	2457.1733	0.036	1	76	2.7e-008	1	U	K.DAIPENLPPLTADFAEDKDVCK.N
5544	295 - 316	820.0652	2457.1739	2457.1733	0.24	1	83	5.4e-009	1	U	K.DAIPENLPPLTADFAEDKDVCK.N
5545	295 - 316	1229.5942	2457.1739	2457.1733	0.24	1	77	1.8e-008	1	U	K.DAIPENLPPLTADFAEDKDVCK.N
5546	295 - 316	1229.5947	2457.1749	2457.1733	0.64	1	80	1.1e-008	1	U	K.DAIPENLPPLTADFAEDKDVCK.N
5547	295 - 316	820.0657	2457.1754	2457.1733	0.82	1	57	2.2e-006	1	U	K.DAIPENLPPLTADFAEDKDVCK.N
5548	295 - 316	820.0657	2457.1754	2457.1733	0.82	1	76	2.7e-008	1	U	K.DAIPENLPPLTADFAEDKDVCK.N
5549	295 - 316	1229.5955	2457.1764	2457.1733	1.23	1	92	5.9e-010	1	U	K.DAIPENLPPLTADFAEDKDVCK.N
5719	295 - 316	874.0809	2619.2208	2619.2262	-2.06	1	30	0.001	1	U	K.DAIPENLPPLTADFAEDKDVCK.N + Glycation
5720	295 - 316	874.0813	2619.2221	2619.2262	-1.56	1	56	2.4e-006	1	U	K.DAIPENLPPLTADFAEDKDVCK.N + Glycation
5721	295 - 316	874.0817	2619.2234	2619.2262	-1.07	1	56	2.4e-006	1	U	K.DAIPENLPPLTADFAEDKDVCK.N + Glycation
5722	295 - 316	874.0818	2619.2235	2619.2262	-1.00	1	44	3.7e-005	1	U	K.DAIPENLPPLTADFAEDKDVCK.N + Glycation
5723	295 - 316	874.0818	2619.2235	2619.2262	-1.00	1	54	4e-006	1	U	K.DAIPENLPPLTADFAEDKDVCK.N + Glycation
5724	295 - 316	874.0819	2619.2239	2619.2262	-0.86	1	48	1.7e-005	1	U	K.DAIPENLPPLTADFAEDKDVCK.N + Glycation
5725	295 - 316	874.0819	2619.2239	2619.2262	-0.86	1	60	1e-006	1	U	K.DAIPENLPPLTADFAEDKDVCK.N + Glycation
5726	295 - 316	874.0819	2619.2239	2619.2262	-0.86	1	45	3.2e-005	1	U	K.DAIPENLPPLTADFAEDKDVCK.N + Glycation
5727	295 - 316	874.0820	2619.2243	2619.2262	-0.73	1	47	1.9e-005	1	U	K.DAIPENLPPLTADFAEDKDVCK.N + Glycation
5728	295 - 316	874.0833	2619.2279	2619.2262	0.67	1	52	6.5e-006	1	U	K.DAIPENLPPLTADFAEDKDVCK.N + Glycation
5926	295 - 322	1118.5283	3352.5631	3352.5656	-0.75	2	25	0.0029	1	U	K.DAIPENLPPLTADFAEDKDVCKNYQEAK.D + Gly
5927	295 - 322	839.1482	3352.5639	3352.5656	-0.52	2	28	0.0017	1	U	K.DAIPENLPPLTADFAEDKDVCKNYQEAK.D + Gly
5928	295 - 322	839.1484	3352.5647	3352.5656	-0.29	2	31	0.00089	1	U	K.DAIPENLPPLTADFAEDKDVCKNYQEAK.D + Gly
5930	295 - 322	1118.5291	3352.5653	3352.5656	-0.096	2	20	0.0096	1	U	K.DAIPENLPPLTADFAEDKDVCKNYQEAK.D + Gly
5931	295 - 322	1118.5291	3352.5653	3352.5656	-0.096	2	29	0.0013	1	U	K.DAIPENLPPLTADFAEDKDVCKNYQEAK.D + Gly
5932	295 - 322	839.1486	3352.5654	3352.5656	-0.080	2	15	0.032	1	U	K.DAIPENLPPLTADFAEDKDVCKNYQEAK.D + Gly
5934	295 - 322	1118.5295	3352.5668	3352.5656	0.34	2	18	0.016	1	U	K.DAIPENLPPLTADFAEDKDVCKNYQEAK.D + Gly
3557	323 - 335	784.3743	1566.7341	1566.7354	-0.85	0	82	6e-009	1	U	K.DAFLGSFLYEYSR.R
3558	323 - 335	784.3743	1566.7341	1566.7354	-0.85	0	72	6.9e-008	1	U	K.DAFLGSFLYEYSR.R
3559	323 - 335	784.3744	1566.7342	1566.7354	-0.78	0	73	5.6e-008	1	U	K.DAFLGSFLYEYSR.R
3560	323 - 335	784.3744	1566.7342	1566.7354	-0.78	0	72	7e-008	1	U	K.DAFLGSFLYEYSR.R
3561	323 - 335	784.3745	1566.7345	1566.7354	-0.62	0	72	6.7e-008	1	U	K.DAFLGSFLYEYSR.R
3562	323 - 335	784.3748	1566.7350	1566.7354	-0.30	0	76	2.4e-008	1	U	K.DAFLGSFLYEYSR.R
2693	336 - 347	480.6077	1438.8012	1438.8045	-2.28	1	47	2e-005	1	U	R.RHPEYAVSVLLR.L
2694	336 - 347	720.4082	1438.8018	1438.8045	-1.81	1	49	1.3e-005	1	U	R.RHPEYAVSVLLR.L
2695	336 - 347	720.4084	1438.8022	1438.8045	-1.55	1	49	1.2e-005	1	U	R.RHPEYAVSVLLR.L
2696	336 - 347	720.4085	1438.8025	1438.8045	-1.38	1	54	3.7e-006	1	U	R.RHPEYAVSVLLR.L
2697	336 - 347	480.6082	1438.8026	1438.8045	-1.28	1	48	1.6e-005	1	U	R.RHPEYAVSVLLR.L
2698	336 - 347	720.4088	1438.8029	1438.8045	-1.05	1	54	3.7e-006	1	U	R.RHPEYAVSVLLR.L
2699	336 - 347	480.6083	1438.8030	1438.8045	-1.01	1	63	5.2e-007	1	U	R.RHPEYAVSVLLR.L
2700	336 - 347	360.7080	1438.8031	1438.8045	-0.98	1	57	2.2e-006	1	U	R.RHPEYAVSVLLR.L
2701	336 - 347	720.4088	1438.8031	1438.8045	-0.97	1	54	3.7e-006	1	U	R.RHPEYAVSVLLR.L
2702	336 - 347	720.4089	1438.8032	1438.8045	-0.88	1	54	4e-006	1	U	R.RHPEYAVSVLLR.L
2703	336 - 347	720.4089	1438.8032	1438.8045	-0.88	1	53	4.7e-006	1	U	R.RHPEYAVSVLLR.L
2704	336 - 347	720.4089	1438.8032	1438.8045	-0.88	1	51	8.4e-006	1	U	R.RHPEYAVSVLLR.L
2705	336 - 347	360.7081	1438.8033	1438.8045	-0.81	1	47	2.2e-005	1	U	R.RHPEYAVSVLLR.L
2706	336 - 347	360.7081	1438.8033	1438.8045	-0.81	1	56	2.3e-006	1	U	R.RHPEYAVSVLLR.L
2707	336 - 347	360.7081	1438.8034	1438.8045	-0.72	1	54	4.4e-006	1	U	R.RHPEYAVSVLLR.L
2708	336 - 347	720.4090	1438.8034	1438.8045	-0.70	1	67	1.8e-007	1	U	R.RHPEYAVSVLLR.L
2709	336 - 347	720.4090	1438.8034	1438.8045	-0.70	1	56	2.3e-006	1	U	R.RHPEYAVSVLLR.L
2710	336 - 347	720.4090	1438.8034	1438.8045	-0.70	1	64	3.8e-007	1	U	R.RHPEYAVSVLLR.L
2711	336 - 347	720.4090	1438.8034	1438.8045	-0.70	1	53	4.9e-006	1	U	R.RHPEYAVSVLLR.L
2712	336 - 347	720.4090	1438.8034	1438.8045	-0.70	1	53	4.7e-006	1	U	R.RHPEYAVSVLLR.L
2713	336 - 347	720.4090	1438.8034	1438.8045	-0.70	1	49	1.2e-005	1	U	R.RHPEYAVSVLLR.L
2714	336 - 347	480.6084	1438.8035	1438.8045	-0.69	1	48	1.5e-005	1	U	R.RHPEYAVSVLLR.L
2715	336 - 347	360.7082	1438.8035	1438.8045	-0.64	1	49	1.4e-005	1	U	R.RHPEYAVSVLLR.L
2716	336 - 347	720.4091	1438.8036	1438.8045	-0.62	1	57	1.9e-006	1	U	R.RHPEYAVSVLLR.L
2717	336 - 347	360.7082	1438.8037	1438.8045	-0.56	1	54	4.4e-006	1	U	R.RHPEYAVSVLLR.L
2718	336 - 347	720.4091	1438.8037	1438.8045	-0.54	1	54	3.8e-006	1	U	R.RHPEYAVSVLLR.L
2719	336 - 347	480.6085	1438.8037	1438.8045	-0.51	1	60	9.9e-007	1	U	R.RHPEYAVSVLLR.L
2720	336 - 347	480.6086	1438.8039	1438.8045	-0.38	1	53	4.6e-006	1	U	R.RHPEYAVSVLLR.L
2721	336 - 347	720.4092	1438.8039	1438.8045	-0.37	1	61	8.1e-007	1	U	R.RHPEYAVSVLLR.L
2722	336 - 347	720.4092	1438.8039	1438.8045	-0.37	1	54	3.7e-006	1	U	R.RHPEYAVSVLLR.L
2723	336 - 347	480.6086	1438.8040	1438.8045	-0.32	1	51	8.8e-006	1	U	R.RHPEYAVSVLLR.L

Query	Start - End	Observed	Mr(expt)	Mr(calc)	ppm	M	Score	Expect	Rank	U	Peptide
2724	336 - 347	360.7083	1438.8040	1438.8045	-0.31	1	53	4.5e-006	1	U	R.RHPEYAVSVLLR.L
2725	336 - 347	720.4093	1438.8040	1438.8045	-0.29	1	49	1.3e-005	1	U	R.RHPEYAVSVLLR.L
2726	336 - 347	480.6086	1438.8041	1438.8045	-0.26	1	54	4.4e-006	1	U	R.RHPEYAVSVLLR.L
2727	336 - 347	480.6087	1438.8043	1438.8045	-0.13	1	51	7.8e-006	1	U	R.RHPEYAVSVLLR.L
2728	336 - 347	480.6087	1438.8043	1438.8045	-0.13	1	63	5.6e-007	1	U	R.RHPEYAVSVLLR.L
2729	336 - 347	480.6087	1438.8043	1438.8045	-0.13	1	63	5.1e-007	1	U	R.RHPEYAVSVLLR.L
2730	336 - 347	720.4094	1438.8043	1438.8045	-0.12	1	54	4.3e-006	1	U	R.RHPEYAVSVLLR.L
2731	336 - 347	480.6087	1438.8044	1438.8045	-0.067	1	51	7.7e-006	1	U	R.RHPEYAVSVLLR.L
2732	336 - 347	480.6088	1438.8045	1438.8045	-0.0049	1	51	8.1e-006	1	U	R.RHPEYAVSVLLR.L
2733	336 - 347	480.6088	1438.8045	1438.8045	-0.0049	1	65	2.9e-007	1	U	R.RHPEYAVSVLLR.L
2734	336 - 347	480.6088	1438.8045	1438.8045	-0.0049	1	57	1.9e-006	1	U	R.RHPEYAVSVLLR.L
2735	336 - 347	480.6088	1438.8046	1438.8045	0.079	1	51	8.2e-006	1	U	R.RHPEYAVSVLLR.L
2736	336 - 347	480.6088	1438.8047	1438.8045	0.14	1	49	1.4e-005	1	U	R.RHPEYAVSVLLR.L
2737	336 - 347	480.6088	1438.8047	1438.8045	0.14	1	63	5.2e-007	1	U	R.RHPEYAVSVLLR.L
2738	336 - 347	720.4096	1438.8047	1438.8045	0.15	1	43	5.4e-005	1	U	R.RHPEYAVSVLLR.L
2739	336 - 347	480.6089	1438.8048	1438.8045	0.20	1	51	7.6e-006	1	U	R.RHPEYAVSVLLR.L
2740	336 - 347	480.6089	1438.8048	1438.8045	0.20	1	53	4.6e-006	1	U	R.RHPEYAVSVLLR.L
2741	336 - 347	480.6089	1438.8048	1438.8045	0.20	1	53	4.6e-006	1	U	R.RHPEYAVSVLLR.L
2742	336 - 347	480.6089	1438.8048	1438.8045	0.27	1	51	7.7e-006	1	U	R.RHPEYAVSVLLR.L
2743	336 - 347	480.6089	1438.8048	1438.8045	0.27	1	61	8.8e-007	1	U	R.RHPEYAVSVLLR.L
2744	336 - 347	480.6089	1438.8048	1438.8045	0.27	1	60	1e-006	1	U	R.RHPEYAVSVLLR.L
2745	336 - 347	480.6089	1438.8049	1438.8045	0.33	1	51	7.7e-006	1	U	R.RHPEYAVSVLLR.L
2746	336 - 347	480.6089	1438.8049	1438.8045	0.33	1	54	4.3e-006	1	U	R.RHPEYAVSVLLR.L
2747	336 - 347	480.6090	1438.8051	1438.8045	0.45	1	51	7.6e-006	1	U	R.RHPEYAVSVLLR.L
2748	336 - 347	480.6090	1438.8051	1438.8045	0.45	1	63	5.1e-007	1	U	R.RHPEYAVSVLLR.L
2749	336 - 347	480.6090	1438.8052	1438.8045	0.52	1	51	8.7e-006	1	U	R.RHPEYAVSVLLR.L
2750	336 - 347	480.6091	1438.8054	1438.8045	0.64	1	61	8.5e-007	1	U	R.RHPEYAVSVLLR.L
2751	336 - 347	480.6091	1438.8054	1438.8045	0.64	1	57	1.9e-006	1	U	R.RHPEYAVSVLLR.L
2752	336 - 347	480.6092	1438.8057	1438.8045	0.83	1	63	5.1e-007	1	U	R.RHPEYAVSVLLR.L
2753	336 - 347	480.6094	1438.8065	1438.8045	1.41	1	60	1e-006	1	U	R.RHPEYAVSVLLR.L
2754	336 - 347	480.6095	1438.8067	1438.8045	1.54	1	61	8.7e-007	1	U	R.RHPEYAVSVLLR.L
2755	336 - 347	480.6096	1438.8069	1438.8045	1.66	1	63	5.1e-007	1	U	R.RHPEYAVSVLLR.L
2756	336 - 347	480.6096	1438.8069	1438.8045	1.73	1	66	2.6e-007	1	U	R.RHPEYAVSVLLR.L
2028	337 - 347	642.3578	1282.7011	1282.7034	-1.72	0	59	1.3e-006	1	U	R.HPEYAVSVLLR.L
2029	337 - 347	642.3579	1282.7013	1282.7034	-1.63	0	57	2.1e-006	1	U	R.HPEYAVSVLLR.L
2030	337 - 347	642.3583	1282.7020	1282.7034	-1.05	0	61	8.8e-007	1	U	R.HPEYAVSVLLR.L
2031	337 - 347	642.3583	1282.7021	1282.7034	-0.96	0	55	2.9e-006	1	U	R.HPEYAVSVLLR.L
2032	337 - 347	642.3586	1282.7026	1282.7034	-0.58	0	42	5.8e-005	1	U	R.HPEYAVSVLLR.L
2033	337 - 347	642.3586	1282.7027	1282.7034	-0.49	0	41	7.3e-005	1	U	R.HPEYAVSVLLR.L
2034	337 - 347	642.3586	1282.7027	1282.7034	-0.49	0	57	2.2e-006	1	U	R.HPEYAVSVLLR.L
2035	337 - 347	428.5749	1282.7028	1282.7034	-0.40	0	48	1.7e-005	1	U	R.HPEYAVSVLLR.L
2036	337 - 347	642.3588	1282.7030	1282.7034	-0.30	0	59	1.1e-006	1	U	R.HPEYAVSVLLR.L
2037	337 - 347	428.5750	1282.7032	1282.7034	-0.12	0	57	2.2e-006	1	U	R.HPEYAVSVLLR.L
2038	337 - 347	642.3590	1282.7033	1282.7034	-0.0047	0	49	1.2e-005	1	U	R.HPEYAVSVLLR.L
2039	337 - 347	642.3590	1282.7033	1282.7034	-0.0047	0	61	7.7e-007	1	U	R.HPEYAVSVLLR.L
2040	337 - 347	428.5751	1282.7034	1282.7034	0.022	0	53	5.3e-006	1	U	R.HPEYAVSVLLR.L
2041	337 - 347	428.5751	1282.7035	1282.7034	0.092	0	57	2e-006	1	U	R.HPEYAVSVLLR.L
2042	337 - 347	428.5751	1282.7036	1282.7034	0.16	0	50	9.5e-006	1	U	R.HPEYAVSVLLR.L
2043	337 - 347	428.5752	1282.7037	1282.7034	0.23	0	62	6.4e-007	1	U	R.HPEYAVSVLLR.L
2044	337 - 347	428.5752	1282.7038	1282.7034	0.33	0	65	3e-007	1	U	R.HPEYAVSVLLR.L
5080	348 - 362	988.9432	1975.8718	1975.8754	-1.82	1	69	1.4e-007	1	U	R.LAKEYEATLEECCAK.D + Glycation (K)
5081	348 - 362	988.9439	1975.8733	1975.8754	-1.09	1	73	4.7e-008	1	U	R.LAKEYEATLEECCAK.D + Glycation (K)
5082	348 - 362	659.6317	1975.8733	1975.8754	-1.07	1	54	3.7e-006	1	U	R.LAKEYEATLEECCAK.D + Glycation (K)
5083	348 - 362	659.6318	1975.8737	1975.8754	-0.87	1	54	3.6e-006	1	U	R.LAKEYEATLEECCAK.D + Glycation (K)
5084	348 - 362	988.9442	1975.8739	1975.8754	-0.78	1	73	4.9e-008	1	U	R.LAKEYEATLEECCAK.D + Glycation (K)
5085	348 - 362	659.6319	1975.8739	1975.8754	-0.78	1	67	2.2e-007	1	U	R.LAKEYEATLEECCAK.D + Glycation (K)
5086	348 - 362	988.9445	1975.8744	1975.8754	-0.53	1	66	2.7e-007	1	U	R.LAKEYEATLEECCAK.D + Glycation (K)
5087	348 - 362	659.6321	1975.8746	1975.8754	-0.42	1	50	9.1e-006	1	U	R.LAKEYEATLEECCAK.D + Glycation (K)
5088	348 - 362	659.6321	1975.8746	1975.8754	-0.42	1	55	3.4e-006	1	U	R.LAKEYEATLEECCAK.D + Glycation (K)
5089	348 - 362	659.6324	1975.8755	1975.8754	0.056	1	55	3.4e-006	1	U	R.LAKEYEATLEECCAK.D + Glycation (K)
3199	351 - 362	751.8084	1501.6021	1501.6065	-2.87	0	55	3.3e-006	1	U	K.EYEATLEECCAK.D
3200	351 - 362	751.8093	1501.6040	1501.6065	-1.65	0	68	1.7e-007	1	U	K.EYEATLEECCAK.D
3201	351 - 362	751.8094	1501.6043	1501.6065	-1.41	0	72	6.6e-008	1	U	K.EYEATLEECCAK.D
3202	351 - 362	751.8096	1501.6046	1501.6065	-1.25	0	56	2.8e-006	1	U	K.EYEATLEECCAK.D
3203	351 - 362	751.8096	1501.6046	1501.6065	-1.25	0	56	2.8e-006	1	U	K.EYEATLEECCAK.D
3204	351 - 362	751.8096	1501.6046	1501.6065	-1.25	0	72	6.6e-008	1	U	K.EYEATLEECCAK.D
3205	351 - 362	751.8096	1501.6047	1501.6065	-1.17	0	59	1.2e-006	1	U	K.EYEATLEECCAK.D
3206	351 - 362	751.8096	1501.6047	1501.6065	-1.17	0	59	1.2e-006	1	U	K.EYEATLEECCAK.D
3207	351 - 362	751.8096	1501.6047	1501.6065	-1.17	0	72	6.6e-008	1	U	K.EYEATLEECCAK.D
3208	351 - 362	751.8097	1501.6048	1501.6065	-1.09	0	55	3.4e-006	1	U	K.EYEATLEECCAK.D
3209	351 - 362	751.8097	1501.6048	1501.6065	-1.09	0	44	3.8e-005	1	U	K.EYEATLEECCAK.D
3210	351 - 362	751.8098	1501.6049	1501.6065	-1.01	0	55	3.1e-006	1	U	K.EYEATLEECCAK.D
3211	351 - 362	751.8098	1501.6049	1501.6065	-1.01	0	59	1.2e-006	1	U	K.EYEATLEECCAK.D
3212	351 - 362	751.8098	1501.6049	1501.6065	-1.01	0	72	6.6e-008	1	U	K.EYEATLEECCAK.D
3213	351 - 362	751.8101	1501.6056	1501.6065	-0.59	0	54	3.8e-006	1	U	K.EYEATLEECCAK.D
3214	351 - 362	751.8101	1501.6056	1501.6065	-0.59	0	59	1.2e-006	1	U	K.EYEATLEECCAK.D
3215	351 - 362	751.8101	1501.6056	1501.6065	-0.59	0	54	3.6e-006	1	U	K.EYEATLEECCAK.D
3216	351 - 362	751.8101	1501.6057	1501.6065	-0.51	0	59	1.3e-006	1	U	K.EYEATLEECCAK.D
3217	351 - 362	501.5425	1501.6058	1501.6065	-0.45	0	36	0.00026	1	U	K.EYEATLEECCAK.D
3218	351 - 362	501.5426	1501.6060	1501.6065	-0.33	0	47	2e-005	1	U	K.EYEATLEECCAK.D
3219	351 - 362	751.8104	1501.6062	1501.6065	-0.20	0	59	1.2e-006	1	U	K.EYEATLEECCAK.D
3220	351 - 362	501.5428	1501.6065	1501.6065	-0.0060	0	51	7.4e-006	1	U	K.EYEATLEECCAK.D
3221	351 - 362	751.8106	1501.6067	1501.6065	0.14	0	72	6.7e-008	1	U	K.EYEATLEECCAK.D
3222	351 - 362	751.8112	1501.6078	1501.6065	0.87	0	52	6.4e-006	1	U	K.EYEATLEECCAK.D
3223	351 - 362	751.8112	1501.6079	1501.6065	0.95	0	72	6.6e-008	1	U	K.EYEATLEECCAK.D
5904	351 - 375	800.8291	3199.2873	3199.2944	-2.22	1	19	0.012	1	U	K.EYEATLEECCAKDDPHACYSTVFDK.L + Glycat
3492	363 - 375	777.8283	1553.6419	1553.6457	-2.40	0	29	0.0013	1	U	K.DDPHACYSTVFDK.L
3493	363 - 375	777.8290	1553.6435	1553.6457	-1.38	0	32	0.00057	1	U	K.DDPHACYSTVFDK.L
3494	363 - 375	777.8290	1553.6435	1553.6457	-1.38	0	30	0.001	1	U	K.DDPHACYSTVFDK.L
3495	363 - 375	777.8291	1553.6436	1553.6457	-1.31	0	15	0.031	1	U	K.DDPHACYSTVFDK.L

Query	Start - End	Observed	Mr(expt)	Mr(calc)	ppm	M	Score	Expect	Rank	U	Peptide
3496	363 - 375	777.8291	1553.6436	1553.6457	-1.31	0	37	0.00022	1	U	K.DDPHACYSTVFDK.L
3497	363 - 375	777.8291	1553.6436	1553.6457	-1.31	0	27	0.0022	1	U	K.DDPHACYSTVFDK.L
3498	363 - 375	777.8292	1553.6438	1553.6457	-1.23	0	43	4.8e-005	1	U	K.DDPHACYSTVFDK.L
3499	363 - 375	777.8292	1553.6438	1553.6457	-1.23	0	35	0.00031	1	U	K.DDPHACYSTVFDK.L
3500	363 - 375	777.8292	1553.6438	1553.6457	-1.23	0	36	0.00026	1	U	K.DDPHACYSTVFDK.L
3501	363 - 375	518.8886	1553.6438	1553.6457	-1.19	0	37	0.00018	1	U	K.DDPHACYSTVFDK.L
3502	363 - 375	777.8292	1553.6439	1553.6457	-1.15	0	43	4.8e-005	1	U	K.DDPHACYSTVFDK.L
3503	363 - 375	777.8293	1553.6441	1553.6457	-0.98	0	36	0.00026	1	U	K.DDPHACYSTVFDK.L
3504	363 - 375	777.8295	1553.6445	1553.6457	-0.75	0	26	0.0023	1	U	K.DDPHACYSTVFDK.L
3505	363 - 375	777.8295	1553.6445	1553.6457	-0.75	0	47	2.2e-005	1	U	K.DDPHACYSTVFDK.L
3506	363 - 375	777.8296	1553.6446	1553.6457	-0.67	0	31	0.00088	1	U	K.DDPHACYSTVFDK.L
3507	363 - 375	777.8297	1553.6447	1553.6457	-0.60	0	30	0.001	1	U	K.DDPHACYSTVFDK.L
3508	363 - 375	777.8297	1553.6449	1553.6457	-0.52	0	27	0.002	1	U	K.DDPHACYSTVFDK.L
3509	363 - 375	518.8890	1553.6451	1553.6457	-0.36	0	27	0.0019	1	U	K.DDPHACYSTVFDK.L
3510	363 - 375	518.8891	1553.6455	1553.6457	-0.13	0	49	1.3e-005	1	U	K.DDPHACYSTVFDK.L
3511	363 - 375	518.8892	1553.6457	1553.6457	-0.015	0	42	6.1e-005	1	U	K.DDPHACYSTVFDK.L
3512	363 - 375	777.8301	1553.6457	1553.6457	0.033	0	17	0.02	1	U	K.DDPHACYSTVFDK.L
3513	363 - 375	518.8892	1553.6458	1553.6457	0.10	0	37	0.00018	1	U	K.DDPHACYSTVFDK.L
3514	363 - 375	518.8892	1553.6458	1553.6457	0.10	0	25	0.0029	1	U	K.DDPHACYSTVFDK.L
3515	363 - 375	518.8892	1553.6458	1553.6457	0.10	0	49	1.3e-005	1	U	K.DDPHACYSTVFDK.L
3516	363 - 375	518.8893	1553.6462	1553.6457	0.33	0	57	1.9e-006	1	U	K.DDPHACYSTVFDK.L
3517	363 - 375	777.8304	1553.6463	1553.6457	0.42	0	27	0.0018	1	U	K.DDPHACYSTVFDK.L
3518	363 - 375	518.8895	1553.6468	1553.6457	0.70	0	38	0.00015	1	U	K.DDPHACYSTVFDK.L
3519	363 - 375	518.8898	1553.6477	1553.6457	1.28	0	46	2.6e-005	1	U	K.DDPHACYSTVFDK.L
3520	363 - 375	518.8899	1553.6478	1553.6457	1.39	0	49	1.3e-005	1	U	K.DDPHACYSTVFDK.L
3521	363 - 375	777.8315	1553.6484	1553.6457	1.76	0	19	0.013	1	U	K.DDPHACYSTVFDK.L
5003	363 - 377	979.4448	1956.8750	1956.8775	-1.30	1	18	0.017	1	U	K.DDPHACYSTVFDKLK.H + Glycation (K)
5004	363 - 377	979.4451	1956.8757	1956.8775	-0.92	1	26	0.0028	1	U	K.DDPHACYSTVFDKLK.H + Glycation (K)
5005	363 - 377	653.2992	1956.8757	1956.8775	-0.91	1	14	0.042	1	U	K.DDPHACYSTVFDKLK.H + Glycation (K)
5006	363 - 377	653.2996	1956.8770	1956.8775	-0.25	1	31	0.00088	1	U	K.DDPHACYSTVFDKLK.H + Glycation (K)
5007	363 - 377	653.2996	1956.8770	1956.8775	-0.25	1	26	0.0025	1	U	K.DDPHACYSTVFDKLK.H + Glycation (K)
5009	363 - 377	653.2997	1956.8774	1956.8775	-0.064	1	15	0.031	1	U	K.DDPHACYSTVFDKLK.H + Glycation (K)
5011	363 - 377	490.2268	1956.8780	1956.8775	0.25	1	15	0.033	1	U	K.DDPHACYSTVFDKLK.H + Glycation (K)
4162	376 - 388	854.9768	1707.9389	1707.9407	-1.01	1	38	0.00016	1	U	K.LKHLVDEPQNLIK.Q + Glycation (K)
4163	376 - 388	570.3204	1707.9393	1707.9407	-0.82	1	23	0.0049	1	U	K.LKHLVDEPQNLIK.Q + Glycation (K)
4164	376 - 388	854.9771	1707.9397	1707.9407	-0.59	1	37	0.0002	1	U	K.LKHLVDEPQNLIK.Q + Glycation (K)
4165	376 - 388	570.3207	1707.9402	1707.9407	-0.27	1	27	0.002	1	U	K.LKHLVDEPQNLIK.Q + Glycation (K)
4166	376 - 388	570.3207	1707.9402	1707.9407	-0.27	1	34	0.00038	1	U	K.LKHLVDEPQNLIK.Q + Glycation (K)
4167	376 - 388	427.9923	1707.9403	1707.9407	-0.24	1	19	0.012	1	U	K.LKHLVDEPQNLIK.Q + Glycation (K)
4168	376 - 388	570.3208	1707.9406	1707.9407	-0.060	1	21	0.0071	1	U	K.LKHLVDEPQNLIK.Q + Glycation (K)
4169	376 - 388	427.9925	1707.9409	1707.9407	0.11	1	22	0.0063	1	U	K.LKHLVDEPQNLIK.Q + Glycation (K)
4170	376 - 388	570.3209	1707.9409	1707.9407	0.15	1	28	0.0014	1	U	K.LKHLVDEPQNLIK.Q + Glycation (K)
4171	376 - 388	427.9925	1707.9410	1707.9407	0.18	1	18	0.014	1	U	K.LKHLVDEPQNLIK.Q + Glycation (K)
4172	376 - 388	570.3210	1707.9413	1707.9407	0.36	1	31	0.00086	1	U	K.LKHLVDEPQNLIK.Q + Glycation (K)
2118	378 - 388	653.3603	1304.7060	1304.7088	-2.15	0	30	0.00092	1	U	K.HLVDEPQNLIK.Q
2119	378 - 388	653.3608	1304.7070	1304.7088	-1.40	0	28	0.0014	1	U	K.HLVDEPQNLIK.Q
2120	378 - 388	653.3611	1304.7076	1304.7088	-0.94	0	49	1.2e-005	1	U	K.HLVDEPQNLIK.Q
2121	378 - 388	653.3611	1304.7076	1304.7088	-0.94	0	47	1.9e-005	1	U	K.HLVDEPQNLIK.Q
2123	378 - 388	653.3611	1304.7077	1304.7088	-0.83	0	46	2.8e-005	1	U	K.HLVDEPQNLIK.Q
2125	378 - 388	653.3613	1304.7080	1304.7088	-0.65	0	26	0.0023	1	U	K.HLVDEPQNLIK.Q
2126	378 - 388	653.3613	1304.7080	1304.7088	-0.65	0	47	2.1e-005	1	U	K.HLVDEPQNLIK.Q
2127	378 - 388	653.3614	1304.7083	1304.7088	-0.37	0	46	2.4e-005	1	U	K.HLVDEPQNLIK.Q
2128	378 - 388	653.3615	1304.7085	1304.7088	-0.28	0	48	1.6e-005	1	U	K.HLVDEPQNLIK.Q
2129	378 - 388	653.3615	1304.7085	1304.7088	-0.28	0	49	1.2e-005	1	U	K.HLVDEPQNLIK.Q
2130	378 - 388	653.3615	1304.7085	1304.7088	-0.28	0	41	7.6e-005	1	U	K.HLVDEPQNLIK.Q
2131	378 - 388	653.3615	1304.7085	1304.7088	-0.28	0	49	1.4e-005	1	U	K.HLVDEPQNLIK.Q
2132	378 - 388	653.3616	1304.7086	1304.7088	-0.19	0	45	3e-005	1	U	K.HLVDEPQNLIK.Q
2133	378 - 388	435.9102	1304.7087	1304.7088	-0.14	0	36	0.00023	1	U	K.HLVDEPQNLIK.Q
2134	378 - 388	435.9102	1304.7087	1304.7088	-0.14	0	48	1.6e-005	1	U	K.HLVDEPQNLIK.Q
2135	378 - 388	653.3616	1304.7087	1304.7088	-0.095	0	46	2.3e-005	1	U	K.HLVDEPQNLIK.Q
2136	378 - 388	653.3616	1304.7087	1304.7088	-0.095	0	46	2.3e-005	1	U	K.HLVDEPQNLIK.Q
2137	378 - 388	435.9102	1304.7088	1304.7088		0	48	1.5e-005	1	U	K.HLVDEPQNLIK.Q
2138	378 - 388	435.9103	1304.7089	1304.7088	0.069	0	36	0.00023	1	U	K.HLVDEPQNLIK.Q
2139	378 - 388	435.9103	1304.7089	1304.7088	0.069	0	48	1.5e-005	1	U	K.HLVDEPQNLIK.Q
2140	378 - 388	653.3618	1304.7090	1304.7088	0.10	0	46	2.4e-005	1	U	K.HLVDEPQNLIK.Q
2141	378 - 388	653.3618	1304.7090	1304.7088	0.10	0	46	2.4e-005	1	U	K.HLVDEPQNLIK.Q
2142	378 - 388	435.9103	1304.7090	1304.7088	0.14	0	48	1.5e-005	1	U	K.HLVDEPQNLIK.Q
2143	378 - 388	435.9103	1304.7092	1304.7088	0.28	0	36	0.00023	1	U	K.HLVDEPQNLIK.Q
2144	378 - 388	435.9103	1304.7092	1304.7088	0.28	0	36	0.00023	1	U	K.HLVDEPQNLIK.Q
2145	378 - 388	435.9103	1304.7092	1304.7088	0.28	0	48	1.6e-005	1	U	K.HLVDEPQNLIK.Q
2146	378 - 388	435.9104	1304.7093	1304.7088	0.34	0	48	1.6e-005	1	U	K.HLVDEPQNLIK.Q
2147	378 - 388	435.9104	1304.7095	1304.7088	0.48	0	35	0.0003	1	U	K.HLVDEPQNLIK.Q
2148	378 - 388	435.9104	1304.7095	1304.7088	0.48	0	48	1.5e-005	1	U	K.HLVDEPQNLIK.Q
2149	378 - 388	435.9104	1304.7095	1304.7088	0.48	0	42	5.9e-005	1	U	K.HLVDEPQNLIK.Q
2150	378 - 388	653.3624	1304.7102	1304.7088	1.04	0	46	2.4e-005	1	U	K.HLVDEPQNLIK.Q
2151	378 - 388	653.3626	1304.7107	1304.7088	1.41	0	46	2.4e-005	1	U	K.HLVDEPQNLIK.Q
2152	378 - 388	653.3629	1304.7113	1304.7088	1.88	0	46	2.4e-005	1	U	K.HLVDEPQNLIK.Q
5599	378 - 396	839.7349	2516.1829	2516.1853	-0.94	1	62	6.6e-007	1	U	K.HLVDEPQNLIKQNCDQFEK.L + Glycation (K)
5600	378 - 396	839.7351	2516.1835	2516.1853	-0.72	1	60	1e-006	1	U	K.HLVDEPQNLIKQNCDQFEK.L + Glycation (K)
5601	378 - 396	839.7351	2516.1835	2516.1853	-0.72	1	61	7.9e-007	1	U	K.HLVDEPQNLIKQNCDQFEK.L + Glycation (K)
5602	378 - 396	839.7352	2516.1837	2516.1853	-0.64	1	63	5.5e-007	1	U	K.HLVDEPQNLIKQNCDQFEK.L + Glycation (K)
5603	378 - 396	839.7352	2516.1839	2516.1853	-0.57	1	63	5.3e-007	1	U	K.HLVDEPQNLIKQNCDQFEK.L + Glycation (K)
5604	378 - 396	839.7353	2516.1840	2516.1853	-0.50	1	60	8.9e-007	1	U	K.HLVDEPQNLIKQNCDQFEK.L + Glycation (K)
5605	378 - 396	839.7355	2516.1846	2516.1853	-0.29	1	64	3.6e-007	1	U	K.HLVDEPQNLIKQNCDQFEK.L + Glycation (K)
5606	378 - 396	630.0535	2516.1850	2516.1853	-0.11	1	52	6.3e-006	1	U	K.HLVDEPQNLIKQNCDQFEK.L + Glycation (K)
5607	378 - 396	630.0536	2516.1853	2516.1853	-0.019	1	49	1.3e-005	1	U	K.HLVDEPQNLIKQNCDQFEK.L + Glycation (K)
5608	378 - 396	630.0536	2516.1855	2516.1853	0.077	1	50	9.4e-006	1	U	K.HLVDEPQNLIKQNCDQFEK.L + Glycation (K)
5609	378 - 396	630.0537	2516.1857	2516.1853	0.17	1	57	1.8e-006	1	U	K.HLVDEPQNLIKQNCDQFEK.L + Glycation (K)
1303	389 - 396	534.7240	1067.4334	1067.4342	-0.71	0	26	0.0025	1	U	K.QNCDQFEK.L

Query	Start - End	Observed	Mr(expt)	Mr(calc)	ppm	M	Score	Expect	Rank	U	Peptide
1304	389 - 396	534.7241	1067.4336	1067.4342	-0.60	0	20	0.011	1	U	K.QNCDQFEK.L
1305	389 - 396	534.7242	1067.4338	1067.4342	-0.37	0	26	0.0024	1	U	K.QNCDQFEK.L
1306	389 - 396	534.7242	1067.4339	1067.4342	-0.26	0	26	0.0026	1	U	K.QNCDQFEK.L
1307	389 - 396	534.7243	1067.4340	1067.4342	-0.15	0	20	0.011	1	U	K.QNCDQFEK.L
1308	389 - 396	534.7243	1067.4340	1067.4342	-0.15	0	27	0.0021	1	U	K.QNCDQFEK.L
1309	389 - 396	534.7244	1067.4343	1067.4342	0.097	0	27	0.002	1	U	K.QNCDQFEK.L
1310	389 - 396	534.7244	1067.4343	1067.4342	0.097	0	26	0.0026	1	U	K.QNCDQFEK.L
1311	389 - 396	534.7244	1067.4343	1067.4342	0.097	0	28	0.0016	1	U	K.QNCDQFEK.L
1312	389 - 396	534.7245	1067.4344	1067.4342	0.21	0	26	0.0023	1	U	K.QNCDQFEK.L
1313	389 - 396	534.7245	1067.4344	1067.4342	0.21	0	27	0.002	1	U	K.QNCDQFEK.L
1314	389 - 396	534.7245	1067.4344	1067.4342	0.21	0	26	0.0025	1	U	K.QNCDQFEK.L
1315	389 - 396	534.7245	1067.4344	1067.4342	0.21	0	27	0.002	1	U	K.QNCDQFEK.L
1316	389 - 396	534.7246	1067.4345	1067.4342	0.32	0	18	0.017	1	U	K.QNCDQFEK.L
1317	389 - 396	534.7246	1067.4345	1067.4342	0.32	0	27	0.002	1	U	K.QNCDQFEK.L
1318	389 - 396	534.7246	1067.4347	1067.4342	0.43	0	27	0.002	1	U	K.QNCDQFEK.L
1319	389 - 396	534.7247	1067.4349	1067.4342	0.66	0	26	0.0026	1	U	K.QNCDQFEK.L
1320	389 - 396	534.7247	1067.4349	1067.4342	0.66	0	26	0.0026	1	U	K.QNCDQFEK.L
3043	397 - 409	740.4007	1478.7868	1478.7881	-0.88	0	97	1.9e-010	1	U	K.LGEYGFQNALIVR.Y
3044	397 - 409	740.4008	1478.7871	1478.7881	-0.72	0	97	1.9e-010	1	U	K.LGEYGFQNALIVR.Y
3045	397 - 409	740.4009	1478.7873	1478.7881	-0.55	0	97	1.9e-010	1	U	K.LGEYGFQNALIVR.Y
3046	397 - 409	740.4010	1478.7874	1478.7881	-0.47	0	97	1.9e-010	1	U	K.LGEYGFQNALIVR.Y
3047	397 - 409	740.4011	1478.7876	1478.7881	-0.39	0	97	1.9e-010	1	U	K.LGEYGFQNALIVR.Y
3048	397 - 409	493.9365	1478.7877	1478.7881	-0.28	0	77	1.9e-008	1	U	K.LGEYGFQNALIVR.Y
3049	397 - 409	740.4013	1478.7881	1478.7881	-0.053	0	97	1.9e-010	1	U	K.LGEYGFQNALIVR.Y
3050	397 - 409	493.9366	1478.7881	1478.7881	-0.017	0	80	9e-009	1	U	K.LGEYGFQNALIVR.Y
3051	397 - 409	493.9367	1478.7883	1478.7881	0.10	0	67	1.8e-007	1	U	K.LGEYGFQNALIVR.Y
3052	397 - 409	740.4014	1478.7883	1478.7881	0.11	0	97	1.9e-010	1	U	K.LGEYGFQNALIVR.Y
3053	397 - 409	740.4014	1478.7883	1478.7881	0.11	0	97	1.9e-010	1	U	K.LGEYGFQNALIVR.Y
3054	397 - 409	493.9368	1478.7885	1478.7881	0.23	0	67	1.8e-007	1	U	K.LGEYGFQNALIVR.Y
3055	397 - 409	493.9368	1478.7886	1478.7881	0.29	0	69	1.2e-007	1	U	K.LGEYGFQNALIVR.Y
3854	413 - 427	820.4688	1638.9231	1638.9305	-4.52	1	97	2e-010	1	U	R.KVPQVSTPTLVEVSR.S
3855	413 - 427	820.4701	1638.9256	1638.9305	-2.96	1	67	2.2e-007	1	U	R.KVPQVSTPTLVEVSR.S
3856	413 - 427	547.3162	1638.9268	1638.9305	-2.23	1	34	0.00036	1	U	R.KVPQVSTPTLVEVSR.S
3857	413 - 427	820.4710	1638.9275	1638.9305	-1.84	1	65	3.1e-007	1	U	R.KVPQVSTPTLVEVSR.S
3858	413 - 427	820.4713	1638.9279	1638.9305	-1.55	1	97	2.2e-010	1	U	R.KVPQVSTPTLVEVSR.S
3859	413 - 427	820.4714	1638.9282	1638.9305	-1.40	1	71	8.2e-008	1	U	R.KVPQVSTPTLVEVSR.S
3860	413 - 427	820.4714	1638.9283	1638.9305	-1.31	1	117	2.1e-012	1	U	R.KVPQVSTPTLVEVSR.S
3861	413 - 427	820.4715	1638.9284	1638.9305	-1.24	1	117	2.2e-012	1	U	R.KVPQVSTPTLVEVSR.S
3862	413 - 427	820.4716	1638.9286	1638.9305	-1.17	1	117	2.2e-012	1	U	R.KVPQVSTPTLVEVSR.S
3863	413 - 427	820.4716	1638.9286	1638.9305	-1.17	1	117	2.1e-012	1	U	R.KVPQVSTPTLVEVSR.S
3864	413 - 427	547.3168	1638.9287	1638.9305	-1.11	1	61	7.5e-007	1	U	R.KVPQVSTPTLVEVSR.S
3865	413 - 427	547.3168	1638.9287	1638.9305	-1.11	1	15	0.029	1	U	R.KVPQVSTPTLVEVSR.S
3866	413 - 427	820.4716	1638.9287	1638.9305	-1.09	1	79	1.3e-008	1	U	R.KVPQVSTPTLVEVSR.S
3867	413 - 427	820.4717	1638.9288	1638.9305	-1.02	1	80	1e-008	1	U	R.KVPQVSTPTLVEVSR.S
3868	413 - 427	820.4717	1638.9288	1638.9305	-1.02	1	79	1.4e-008	1	U	R.KVPQVSTPTLVEVSR.S
3869	413 - 427	820.4717	1638.9288	1638.9305	-1.02	1	117	2.1e-012	1	U	R.KVPQVSTPTLVEVSR.S
3870	413 - 427	820.4717	1638.9288	1638.9305	-1.02	1	117	2.1e-012	1	U	R.KVPQVSTPTLVEVSR.S
3871	413 - 427	820.4718	1638.9290	1638.9305	-0.87	1	117	2.2e-012	1	U	R.KVPQVSTPTLVEVSR.S
3872	413 - 427	820.4718	1638.9290	1638.9305	-0.87	1	116	2.2e-012	1	U	R.KVPQVSTPTLVEVSR.S
3873	413 - 427	820.4718	1638.9290	1638.9305	-0.87	1	117	2.2e-012	1	U	R.KVPQVSTPTLVEVSR.S
3874	413 - 427	820.4718	1638.9290	1638.9305	-0.87	1	117	2.2e-012	1	U	R.KVPQVSTPTLVEVSR.S
3875	413 - 427	820.4718	1638.9290	1638.9305	-0.87	1	117	2.1e-012	1	U	R.KVPQVSTPTLVEVSR.S
3876	413 - 427	820.4719	1638.9292	1638.9305	-0.80	1	96	2.3e-010	1	U	R.KVPQVSTPTLVEVSR.S
3877	413 - 427	820.4719	1638.9293	1638.9305	-0.73	1	117	2.1e-012	1	U	R.KVPQVSTPTLVEVSR.S
3878	413 - 427	820.4719	1638.9293	1638.9305	-0.73	1	62	6.3e-007	1	U	R.KVPQVSTPTLVEVSR.S
3879	413 - 427	820.4719	1638.9293	1638.9305	-0.73	1	117	2.2e-012	1	U	R.KVPQVSTPTLVEVSR.S
3880	413 - 427	820.4720	1638.9294	1638.9305	-0.65	1	97	2.1e-010	1	U	R.KVPQVSTPTLVEVSR.S
3881	413 - 427	547.3172	1638.9298	1638.9305	-0.43	1	46	2.8e-005	1	U	R.KVPQVSTPTLVEVSR.S
3882	413 - 427	547.3172	1638.9298	1638.9305	-0.43	1	20	0.0091	1	U	R.KVPQVSTPTLVEVSR.S
3883	413 - 427	820.4722	1638.9298	1638.9305	-0.42	1	83	4.6e-009	1	U	R.KVPQVSTPTLVEVSR.S
3884	413 - 427	547.3173	1638.9300	1638.9305	-0.32	1	93	4.5e-010	1	U	R.KVPQVSTPTLVEVSR.S
3885	413 - 427	547.3173	1638.9301	1638.9305	-0.21	1	52	6e-006	1	U	R.KVPQVSTPTLVEVSR.S
3886	413 - 427	547.3173	1638.9301	1638.9305	-0.21	1	45	3.1e-005	1	U	R.KVPQVSTPTLVEVSR.S
3887	413 - 427	547.3174	1638.9303	1638.9305	-0.10	1	73	4.7e-008	1	U	R.KVPQVSTPTLVEVSR.S
3888	413 - 427	547.3174	1638.9303	1638.9305	-0.10	1	73	5.2e-008	1	U	R.KVPQVSTPTLVEVSR.S
3889	413 - 427	547.3174	1638.9305	1638.9305	0.0067	1	59	1.4e-006	1	U	R.KVPQVSTPTLVEVSR.S
3890	413 - 427	547.3174	1638.9305	1638.9305	0.0067	1	45	3.5e-005	1	U	R.KVPQVSTPTLVEVSR.S
3891	413 - 427	547.3174	1638.9305	1638.9305	0.0067	1	75	3.2e-008	1	U	R.KVPQVSTPTLVEVSR.S
3892	413 - 427	547.3175	1638.9307	1638.9305	0.12	1	84	4.2e-009	1	U	R.KVPQVSTPTLVEVSR.S
3893	413 - 427	547.3175	1638.9307	1638.9305	0.12	1	63	5.4e-007	1	U	R.KVPQVSTPTLVEVSR.S
3894	413 - 427	547.3175	1638.9307	1638.9305	0.12	1	52	6.3e-006	1	U	R.KVPQVSTPTLVEVSR.S
3895	413 - 427	547.3175	1638.9307	1638.9305	0.12	1	85	3e-009	1	U	R.KVPQVSTPTLVEVSR.S
3896	413 - 427	547.3175	1638.9307	1638.9305	0.12	1	73	5.3e-008	1	U	R.KVPQVSTPTLVEVSR.S
3897	413 - 427	547.3175	1638.9307	1638.9305	0.12	1	83	5.3e-009	1	U	R.KVPQVSTPTLVEVSR.S
3898	413 - 427	547.3175	1638.9307	1638.9305	0.12	1	71	8.7e-008	1	U	R.KVPQVSTPTLVEVSR.S
3899	413 - 427	547.3176	1638.9309	1638.9305	0.24	1	50	9e-006	1	U	R.KVPQVSTPTLVEVSR.S
3900	413 - 427	547.3176	1638.9309	1638.9305	0.24	1	57	2.1e-006	1	U	R.KVPQVSTPTLVEVSR.S
3901	413 - 427	547.3177	1638.9312	1638.9305	0.46	1	71	7.1e-008	1	U	R.KVPQVSTPTLVEVSR.S
3902	413 - 427	547.3177	1638.9312	1638.9305	0.46	1	50	9.3e-006	1	U	R.KVPQVSTPTLVEVSR.S
3903	413 - 427	547.3177	1638.9312	1638.9305	0.46	1	67	2.1e-007	1	U	R.KVPQVSTPTLVEVSR.S
3904	413 - 427	547.3177	1638.9312	1638.9305	0.46	1	62	6.5e-007	1	U	R.KVPQVSTPTLVEVSR.S
3905	413 - 427	547.3177	1638.9312	1638.9305	0.46	1	63	5e-007	1	U	R.KVPQVSTPTLVEVSR.S
3906	413 - 427	547.3178	1638.9314	1638.9305	0.57	1	69	1.4e-007	1	U	R.KVPQVSTPTLVEVSR.S
3907	413 - 427	547.3178	1638.9314	1638.9305	0.57	1	54	3.8e-006	1	U	R.KVPQVSTPTLVEVSR.S
3908	413 - 427	547.3178	1638.9314	1638.9305	0.57	1	76	2.8e-008	1	U	R.KVPQVSTPTLVEVSR.S
3909	413 - 427	547.3178	1638.9316	1638.9305	0.68	1	72	6.3e-008	1	U	R.KVPQVSTPTLVEVSR.S
3910	413 - 427	547.3179	1638.9318	1638.9305	0.79	1	47	2.1e-005	1	U	R.KVPQVSTPTLVEVSR.S
3911	413 - 427	547.3179	1638.9318	1638.9305	0.79	1	69	1.2e-007	1	U	R.KVPQVSTPTLVEVSR.S
3912	413 - 427	547.3180	1638.9323	1638.9305	1.12	1	81	7.2e-009	1	U	R.KVPQVSTPTLVEVSR.S
3913	413 - 427	820.4736	1638.9327	1638.9305	1.36	1	79	1.2e-008	1	U	R.KVPQVSTPTLVEVSR.S
3914	413 - 427	547.3182	1638.9327	1638.9305	1.36	1	52	6.8e-006	1	U	R.KVPQVSTPTLVEVSR.S

Query	Start – End	Observed	Mr(expt)	Mr(calc)	ppm	M	Score	Expect	Rank	U	Peptide
3915	413 – 427	547.3183	1638.9331	1638.9305	1.58	1	83	4.7e-009	1	U	R.KVPQVSTPTLVEVSR.S
3916	413 – 427	547.3184	1638.9334	1638.9305	1.80	1	73	5.1e-008	1	U	R.KVPQVSTPTLVEVSR.S
3917	413 – 427	547.3184	1638.9334	1638.9305	1.80	1	71	8.1e-008	1	U	R.KVPQVSTPTLVEVSR.S
3918	413 – 427	547.3187	1638.9342	1638.9305	2.24	1	72	6.4e-008	1	U	R.KVPQVSTPTLVEVSR.S
3919	413 – 427	547.3191	1638.9354	1638.9305	3.03	1	74	3.7e-008	1	U	R.KVPQVSTPTLVEVSR.S
3920	413 – 427	547.3195	1638.9367	1638.9305	3.81	1	73	5.1e-008	1	U	R.KVPQVSTPTLVEVSR.S
3227	414 – 427	756.4236	1510.8327	1510.8355	-1.84	0	104	3.7e-011	1	U	K.VPQVSTPTLVEVSR.S
3228	414 – 427	756.4238	1510.8330	1510.8355	-1.68	0	59	1.2e-006	1	U	K.VPQVSTPTLVEVSR.S
3229	414 – 427	756.4242	1510.8338	1510.8355	-1.12	0	85	2.8e-009	1	U	K.VPQVSTPTLVEVSR.S
3230	414 – 427	756.4242	1510.8338	1510.8355	-1.12	0	119	1.2e-012	1	U	K.VPQVSTPTLVEVSR.S
3231	414 – 427	756.4243	1510.8341	1510.8355	-0.95	0	90	8.9e-010	1	U	K.VPQVSTPTLVEVSR.S
3232	414 – 427	756.4243	1510.8341	1510.8355	-0.95	0	90	9.1e-010	1	U	K.VPQVSTPTLVEVSR.S
3233	414 – 427	756.4244	1510.8342	1510.8355	-0.87	0	104	3.7e-011	1	U	K.VPQVSTPTLVEVSR.S
3234	414 – 427	504.6187	1510.8344	1510.8355	-0.75	0	50	1.1e-005	1	U	K.VPQVSTPTLVEVSR.S
3235	414 – 427	756.4245	1510.8344	1510.8355	-0.71	0	104	3.6e-011	1	U	K.VPQVSTPTLVEVSR.S
3236	414 – 427	756.4246	1510.8346	1510.8355	-0.63	0	85	3.1e-009	1	U	K.VPQVSTPTLVEVSR.S
3237	414 – 427	756.4246	1510.8347	1510.8355	-0.55	0	72	6.5e-008	1	U	K.VPQVSTPTLVEVSR.S
3238	414 – 427	504.6190	1510.8352	1510.8355	-0.19	0	54	4.5e-006	1	U	K.VPQVSTPTLVEVSR.S
3239	414 – 427	504.6191	1510.8355	1510.8355	-0.016	0	45	3e-005	1	U	K.VPQVSTPTLVEVSR.S
4181	445 – 458	862.9185	1723.8224	1723.8273	-2.86	0	103	5.3e-011	1	U	R.MPCTEDYLSLILNR.L
4182	445 – 458	862.9199	1723.8252	1723.8273	-1.24	0	99	1.4e-010	1	U	R.MPCTEDYLSLILNR.L
4183	445 – 458	862.9200	1723.8254	1723.8273	-1.10	0	117	1.8e-012	1	U	R.MPCTEDYLSLILNR.L
4184	445 – 458	862.9202	1723.8258	1723.8273	-0.88	0	99	1.4e-010	1	U	R.MPCTEDYLSLILNR.L
4185	445 – 458	862.9202	1723.8259	1723.8273	-0.81	0	99	1.4e-010	1	U	R.MPCTEDYLSLILNR.L
4186	445 – 458	862.9203	1723.8260	1723.8273	-0.74	0	99	1.4e-010	1	U	R.MPCTEDYLSLILNR.L
4187	445 – 458	575.6161	1723.8264	1723.8273	-0.50	0	36	0.00025	1	U	R.MPCTEDYLSLILNR.L
4188	445 – 458	575.6161	1723.8264	1723.8273	-0.50	0	48	1.5e-005	1	U	R.MPCTEDYLSLILNR.L
4189	445 – 458	575.6164	1723.8273	1723.8273	0.024	0	33	0.00054	1	U	R.MPCTEDYLSLILNR.L
4190	445 – 458	575.6164	1723.8273	1723.8273	0.024	0	33	0.0005	1	U	R.MPCTEDYLSLILNR.L
4191	445 – 458	575.6164	1723.8273	1723.8273	0.024	0	36	0.00025	1	U	R.MPCTEDYLSLILNR.L
4192	445 – 458	575.6164	1723.8273	1723.8273	0.024	0	43	4.9e-005	1	U	R.MPCTEDYLSLILNR.L
636	459 – 465	449.7439	897.4732	897.4742	-1.08	0	23	0.0045	1	U	R.LCVLHEK.T
637	459 – 465	449.7439	897.4732	897.4742	-1.08	0	28	0.0017	1	U	R.LCVLHEK.T
638	459 – 465	449.7439	897.4733	897.4742	-1.01	0	27	0.002	1	U	R.LCVLHEK.T
639	459 – 465	449.7440	897.4734	897.4742	-0.87	0	23	0.0048	1	U	R.LCVLHEK.T
640	459 – 465	300.1651	897.4735	897.4742	-0.83	0	17	0.019	1	U	R.LCVLHEK.T
642	459 – 465	449.7441	897.4736	897.4742	-0.67	0	28	0.0016	1	U	R.LCVLHEK.T
643	459 – 465	449.7441	897.4736	897.4742	-0.67	0	24	0.0036	1	U	R.LCVLHEK.T
644	459 – 465	449.7441	897.4736	897.4742	-0.67	0	24	0.0036	1	U	R.LCVLHEK.T
645	459 – 465	300.1652	897.4737	897.4742	-0.63	0	16	0.025	1	U	R.LCVLHEK.T
646	459 – 465	300.1652	897.4737	897.4742	-0.63	0	16	0.024	1	U	R.LCVLHEK.T
647	459 – 465	300.1652	897.4737	897.4742	-0.63	0	16	0.024	1	U	R.LCVLHEK.T
648	459 – 465	449.7441	897.4737	897.4742	-0.61	0	24	0.0038	1	U	R.LCVLHEK.T
649	459 – 465	449.7441	897.4737	897.4742	-0.61	0	24	0.0037	1	U	R.LCVLHEK.T
650	459 – 465	300.1652	897.4737	897.4742	-0.52	0	14	0.037	1	U	R.LCVLHEK.T
651	459 – 465	300.1652	897.4737	897.4742	-0.52	0	16	0.023	1	U	R.LCVLHEK.T
655	459 – 465	300.1652	897.4738	897.4742	-0.42	0	13	0.048	1	U	R.LCVLHEK.T
656	459 – 465	300.1652	897.4738	897.4742	-0.42	0	16	0.025	1	U	R.LCVLHEK.T
657	459 – 465	300.1652	897.4738	897.4742	-0.42	0	15	0.034	1	U	R.LCVLHEK.T
658	459 – 465	300.1652	897.4738	897.4742	-0.42	0	16	0.024	1	U	R.LCVLHEK.T
659	459 – 465	449.7442	897.4738	897.4742	-0.41	0	28	0.0016	1	U	R.LCVLHEK.T
660	459 – 465	449.7442	897.4739	897.4742	-0.34	0	27	0.0021	1	U	R.LCVLHEK.T
661	459 – 465	449.7442	897.4739	897.4742	-0.34	0	24	0.0039	1	U	R.LCVLHEK.T
662	459 – 465	449.7442	897.4739	897.4742	-0.34	0	24	0.0036	1	U	R.LCVLHEK.T
666	459 – 465	449.7443	897.4740	897.4742	-0.27	0	28	0.0015	1	U	R.LCVLHEK.T
667	459 – 465	449.7443	897.4740	897.4742	-0.27	0	24	0.0036	1	U	R.LCVLHEK.T
672	459 – 465	300.1653	897.4740	897.4742	-0.22	0	16	0.024	1	U	R.LCVLHEK.T
673	459 – 465	300.1653	897.4740	897.4742	-0.22	0	13	0.046	1	U	R.LCVLHEK.T
674	459 – 465	449.7443	897.4740	897.4742	-0.21	0	24	0.0038	1	U	R.LCVLHEK.T
675	459 – 465	449.7443	897.4740	897.4742	-0.21	0	24	0.0036	1	U	R.LCVLHEK.T
676	459 – 465	449.7443	897.4740	897.4742	-0.21	0	24	0.0036	1	U	R.LCVLHEK.T
677	459 – 465	449.7443	897.4741	897.4742	-0.14	0	26	0.0024	1	U	R.LCVLHEK.T
678	459 – 465	449.7443	897.4741	897.4742	-0.14	0	28	0.0015	1	U	R.LCVLHEK.T
679	459 – 465	449.7443	897.4741	897.4742	-0.14	0	28	0.0017	1	U	R.LCVLHEK.T
683	459 – 465	449.7444	897.4741	897.4742	-0.072	0	24	0.0036	1	U	R.LCVLHEK.T
684	459 – 465	449.7444	897.4741	897.4742	-0.072	0	25	0.0029	1	U	R.LCVLHEK.T
687	459 – 465	300.1653	897.4742	897.4742	-0.023	0	16	0.026	1	U	R.LCVLHEK.T
688	459 – 465	449.7444	897.4742	897.4742	-0.0056	0	28	0.0015	1	U	R.LCVLHEK.T
689	459 – 465	449.7444	897.4742	897.4742	-0.0056	0	28	0.0015	1	U	R.LCVLHEK.T
691	459 – 465	300.1654	897.4743	897.4742	0.077	0	16	0.026	1	U	R.LCVLHEK.T
692	459 – 465	449.7444	897.4743	897.4742	0.084	0	24	0.0038	1	U	R.LCVLHEK.T
693	459 – 465	449.7444	897.4743	897.4742	0.15	0	32	0.00065	1	U	R.LCVLHEK.T
697	459 – 465	300.1654	897.4744	897.4742	0.21	0	14	0.036	1	U	R.LCVLHEK.T
698	459 – 465	449.7445	897.4744	897.4742	0.22	0	27	0.0019	1	U	R.LCVLHEK.T
699	459 – 465	449.7445	897.4744	897.4742	0.22	0	25	0.0029	1	U	R.LCVLHEK.T
702	459 – 465	300.1654	897.4745	897.4742	0.31	0	13	0.05	1	U	R.LCVLHEK.T
704	459 – 465	449.7446	897.4746	897.4742	0.48	0	28	0.0015	1	U	R.LCVLHEK.T
705	459 – 465	449.7447	897.4748	897.4742	0.62	0	24	0.0039	1	U	R.LCVLHEK.T
4147	459 – 471	426.2233	1700.8641	1700.8655	-0.81	1	17	0.022	1	U	R.LCVLHEKTPVSEK.V + Glycation (K)
4148	459 – 471	426.2237	1700.8655	1700.8655	0.034	1	32	0.00058	1	U	R.LCVLHEKTPVSEK.V + Glycation (K)
4149	459 – 471	426.2240	1700.8669	1700.8655	0.83	1	18	0.014	1	U	R.LCVLHEKTPVSEK.V + Glycation (K)
3801	472 – 483	814.8837	1627.7528	1627.7546	-1.09	1	59	1.4e-006	1	U	K.VTKCCTESLVNR.R + Glycation (K)
3802	472 – 483	814.8838	1627.7530	1627.7546	-0.94	1	57	1.8e-006	1	U	K.VTKCCTESLVNR.R + Glycation (K)
3803	472 – 483	814.8839	1627.7533	1627.7546	-0.79	1	62	6.8e-007	1	U	K.VTKCCTESLVNR.R + Glycation (K)
3804	472 – 483	814.8840	1627.7534	1627.7546	-0.72	1	57	1.9e-006	1	U	K.VTKCCTESLVNR.R + Glycation (K)
3805	472 – 483	543.5917	1627.7534	1627.7546	-0.72	1	64	4e-007	1	U	K.VTKCCTESLVNR.R + Glycation (K)
3806	472 – 483	814.8843	1627.7540	1627.7546	-0.34	1	77	2.2e-008	1	U	K.VTKCCTESLVNR.R + Glycation (K)
3807	472 – 483	543.5920	1627.7541	1627.7546	-0.28	1	77	2.2e-008	1	U	K.VTKCCTESLVNR.R + Glycation (K)
3808	472 – 483	543.5920	1627.7541	1627.7546	-0.28	1	63	4.9e-007	1	U	K.VTKCCTESLVNR.R + Glycation (K)
3809	472 – 483	543.5920	1627.7541	1627.7546	-0.28	1	57	2e-006	1	U	K.VTKCCTESLVNR.R + Glycation (K)

Query	Start - End	Observed	Mr(expt)	Mr(calc)	ppm	M	Score	Expect	Rank	U	Peptide
3810	472 - 483	543.5921	1627.7545	1627.7546	-0.055	1	67	2e-007	1	U	K.VTKCCTESLVNR.R + Glycation (K)
3811	472 - 483	543.5921	1627.7545	1627.7546	-0.055	1	61	7.2e-007	1	U	K.VTKCCTESLVNR.R + Glycation (K)
3812	472 - 483	543.5922	1627.7547	1627.7546	0.055	1	89	1.2e-009	1	U	K.VTKCCTESLVNR.R + Glycation (K)
3813	472 - 483	543.5922	1627.7548	1627.7546	0.17	1	76	2.4e-008	1	U	K.VTKCCTESLVNR.R + Glycation (K)
1563	475 - 483	569.7521	1137.4897	1137.4907	-0.83	0	60	1e-006	1	U	K.CCTESLVNR.R
1564	475 - 483	569.7524	1137.4902	1137.4907	-0.41	0	58	1.5e-006	1	U	K.CCTESLVNR.R
1565	475 - 483	569.7525	1137.4904	1137.4907	-0.20	0	63	4.7e-007	1	U	K.CCTESLVNR.R
1566	475 - 483	569.7525	1137.4904	1137.4907	-0.20	0	63	4.6e-007	1	U	K.CCTESLVNR.R
1567	475 - 483	569.7526	1137.4906	1137.4907	-0.096	0	60	8.9e-007	1	U	K.CCTESLVNR.R
1568	475 - 483	569.7526	1137.4907	1137.4907	0.0097	0	63	4.9e-007	1	U	K.CCTESLVNR.R
1569	475 - 483	569.7527	1137.4908	1137.4907	0.13	0	60	1e-006	1	U	K.CCTESLVNR.R
1570	475 - 483	569.7527	1137.4909	1137.4907	0.24	0	60	1e-006	1	U	K.CCTESLVNR.R
1571	475 - 483	569.7527	1137.4909	1137.4907	0.24	0	56	2.5e-006	1	U	K.CCTESLVNR.R
1572	475 - 483	569.7527	1137.4909	1137.4907	0.24	0	60	1e-006	1	U	K.CCTESLVNR.R
1573	475 - 483	569.7527	1137.4909	1137.4907	0.24	0	60	8.9e-007	1	U	K.CCTESLVNR.R
1574	475 - 483	569.7527	1137.4909	1137.4907	0.24	0	60	9.7e-007	1	U	K.CCTESLVNR.R
1575	475 - 483	569.7528	1137.4911	1137.4907	0.34	0	63	4.8e-007	1	U	K.CCTESLVNR.R
1576	475 - 483	569.7529	1137.4912	1137.4907	0.45	0	60	9.7e-007	1	U	K.CCTESLVNR.R
1577	475 - 483	569.7529	1137.4913	1137.4907	0.55	0	63	4.8e-007	1	U	K.CCTESLVNR.R
1578	475 - 483	569.7529	1137.4913	1137.4907	0.55	0	58	1.6e-006	1	U	K.CCTESLVNR.R
1579	475 - 483	569.7529	1137.4913	1137.4907	0.55	0	60	1.1e-006	1	U	K.CCTESLVNR.R
1580	475 - 483	569.7530	1137.4914	1137.4907	0.66	0	56	2.8e-006	1	U	K.CCTESLVNR.R
1581	475 - 483	569.7531	1137.4915	1137.4907	0.77	0	56	2.5e-006	1	U	K.CCTESLVNR.R
1582	475 - 483	569.7531	1137.4917	1137.4907	0.87	0	58	1.6e-006	1	U	K.CCTESLVNR.R
1583	475 - 483	569.7532	1137.4918	1137.4907	0.98	0	56	2.7e-006	1	U	K.CCTESLVNR.R
1584	475 - 483	569.7532	1137.4919	1137.4907	1.08	0	52	7e-006	1	U	K.CCTESLVNR.R
1585	475 - 483	569.7534	1137.4922	1137.4907	1.31	0	63	4.9e-007	1	U	K.CCTESLVNR.R
1586	475 - 483	569.7537	1137.4928	1137.4907	1.84	0	60	1.1e-006	1	U	K.CCTESLVNR.R
4685	484 - 499	940.9621	1879.9096	1879.9138	-2.23	1	56	2.8e-006	1	U	R.RPCFSALTPDETYVPK.A
4686	484 - 499	627.6442	1879.9107	1879.9138	-1.68	1	29	0.0012	1	U	R.RPCFSALTPDETYVPK.A
4687	484 - 499	940.9629	1879.9112	1879.9138	-1.39	1	56	2.7e-006	1	U	R.RPCFSALTPDETYVPK.A
4688	484 - 499	940.9631	1879.9116	1879.9138	-1.19	1	39	0.00014	1	U	R.RPCFSALTPDETYVPK.A
4689	484 - 499	940.9631	1879.9116	1879.9138	-1.19	1	45	2.9e-005	1	U	R.RPCFSALTPDETYVPK.A
4690	484 - 499	940.9632	1879.9118	1879.9138	-1.06	1	46	2.4e-005	1	U	R.RPCFSALTPDETYVPK.A
4691	484 - 499	940.9633	1879.9120	1879.9138	-0.99	1	48	1.7e-005	1	U	R.RPCFSALTPDETYVPK.A
4692	484 - 499	940.9633	1879.9121	1879.9138	-0.93	1	49	1.1e-005	1	U	R.RPCFSALTPDETYVPK.A
4693	484 - 499	940.9633	1879.9121	1879.9138	-0.93	1	48	1.7e-005	1	U	R.RPCFSALTPDETYVPK.A
4694	484 - 499	940.9633	1879.9121	1879.9138	-0.93	1	48	1.8e-005	1	U	R.RPCFSALTPDETYVPK.A
4695	484 - 499	940.9633	1879.9121	1879.9138	-0.93	1	47	1.8e-005	1	U	R.RPCFSALTPDETYVPK.A
4696	484 - 499	940.9634	1879.9122	1879.9138	-0.86	1	50	1.1e-005	1	U	R.RPCFSALTPDETYVPK.A
4697	484 - 499	940.9634	1879.9123	1879.9138	-0.80	1	46	2.6e-005	1	U	R.RPCFSALTPDETYVPK.A
4698	484 - 499	627.6448	1879.9125	1879.9138	-0.70	1	32	0.00068	1	U	R.RPCFSALTPDETYVPK.A
4699	484 - 499	627.6448	1879.9127	1879.9138	-0.61	1	27	0.0021	1	U	R.RPCFSALTPDETYVPK.A
4700	484 - 499	627.6448	1879.9127	1879.9138	-0.61	1	36	0.00023	1	U	R.RPCFSALTPDETYVPK.A
4701	484 - 499	627.6449	1879.9129	1879.9138	-0.51	1	31	0.00085	1	U	R.RPCFSALTPDETYVPK.A
4702	484 - 499	627.6450	1879.9131	1879.9138	-0.42	1	37	0.00019	1	U	R.RPCFSALTPDETYVPK.A
4703	484 - 499	940.9638	1879.9131	1879.9138	-0.41	1	48	1.5e-005	1	U	R.RPCFSALTPDETYVPK.A
4704	484 - 499	940.9639	1879.9132	1879.9138	-0.34	1	54	3.9e-006	1	U	R.RPCFSALTPDETYVPK.A
4705	484 - 499	940.9639	1879.9132	1879.9138	-0.34	1	50	9.9e-006	1	U	R.RPCFSALTPDETYVPK.A
4706	484 - 499	627.6450	1879.9132	1879.9138	-0.32	1	39	0.00011	1	U	R.RPCFSALTPDETYVPK.A
4707	484 - 499	627.6451	1879.9134	1879.9138	-0.22	1	36	0.00025	1	U	R.RPCFSALTPDETYVPK.A
4708	484 - 499	627.6451	1879.9134	1879.9138	-0.22	1	35	0.00033	1	U	R.RPCFSALTPDETYVPK.A
4709	484 - 499	627.6451	1879.9134	1879.9138	-0.22	1	40	9.3e-005	1	U	R.RPCFSALTPDETYVPK.A
4710	484 - 499	627.6451	1879.9136	1879.9138	-0.13	1	31	0.00071	1	U	R.RPCFSALTPDETYVPK.A
4711	484 - 499	627.6451	1879.9136	1879.9138	-0.13	1	29	0.0013	1	U	R.RPCFSALTPDETYVPK.A
4712	484 - 499	627.6451	1879.9136	1879.9138	-0.13	1	30	0.0011	1	U	R.RPCFSALTPDETYVPK.A
4713	484 - 499	627.6451	1879.9136	1879.9138	-0.13	1	29	0.0012	1	U	R.RPCFSALTPDETYVPK.A
4714	484 - 499	627.6451	1879.9136	1879.9138	-0.13	1	30	0.0011	1	U	R.RPCFSALTPDETYVPK.A
4715	484 - 499	627.6452	1879.9138	1879.9138	-0.033	1	34	0.00036	1	U	R.RPCFSALTPDETYVPK.A
4716	484 - 499	627.6452	1879.9138	1879.9138	-0.033	1	35	0.00031	1	U	R.RPCFSALTPDETYVPK.A
4717	484 - 499	627.6452	1879.9138	1879.9138	-0.033	1	29	0.0014	1	U	R.RPCFSALTPDETYVPK.A
4718	484 - 499	627.6453	1879.9140	1879.9138	0.063	1	33	0.00047	1	U	R.RPCFSALTPDETYVPK.A
4719	484 - 499	940.9643	1879.9140	1879.9138	0.10	1	40	9.7e-005	1	U	R.RPCFSALTPDETYVPK.A
4720	484 - 499	627.6453	1879.9141	1879.9138	0.16	1	25	0.0034	1	U	R.RPCFSALTPDETYVPK.A
4721	484 - 499	627.6453	1879.9141	1879.9138	0.16	1	38	0.00017	1	U	R.RPCFSALTPDETYVPK.A
4722	484 - 499	627.6453	1879.9141	1879.9138	0.16	1	36	0.00024	1	U	R.RPCFSALTPDETYVPK.A
4723	484 - 499	470.9858	1879.9141	1879.9138	0.16	1	52	5.9e-006	1	U	R.RPCFSALTPDETYVPK.A
4724	484 - 499	627.6454	1879.9143	1879.9138	0.27	1	32	0.00056	1	U	R.RPCFSALTPDETYVPK.A
4725	484 - 499	627.6454	1879.9145	1879.9138	0.37	1	35	0.00035	1	U	R.RPCFSALTPDETYVPK.A
4726	484 - 499	627.6456	1879.9149	1879.9138	0.56	1	38	0.00017	1	U	R.RPCFSALTPDETYVPK.A
4727	484 - 499	940.9653	1879.9160	1879.9138	1.15	1	48	1.5e-005	1	U	R.RPCFSALTPDETYVPK.A
4728	484 - 499	627.6462	1879.9167	1879.9138	1.53	1	43	4.5e-005	1	U	R.RPCFSALTPDETYVPK.A
4729	484 - 499	627.6473	1879.9202	1879.9138	3.38	1	33	0.00052	1	U	R.RPCFSALTPDETYVPK.A
4730	484 - 499	627.6475	1879.9206	1879.9138	3.57	1	40	0.0001	1	U	R.RPCFSALTPDETYVPK.A
4731	484 - 499	627.6477	1879.9213	1879.9138	3.97	1	38	0.00015	1	U	R.RPCFSALTPDETYVPK.A
5730	484 - 504	878.4186	2632.2339	2632.2367	-1.05	2	46	2.6e-005	1	U	R.RPCFSALTPDETYVPKAFDEK.L + Glycation (
5731	484 - 504	659.0661	2632.2353	2632.2367	-0.53	2	35	0.00029	1	U	R.RPCFSALTPDETYVPKAFDEK.L + Glycation (
5732	484 - 504	659.0662	2632.2355	2632.2367	-0.43	2	36	0.00027	1	U	R.RPCFSALTPDETYVPKAFDEK.L + Glycation (
4811	505 - 520	636.6441	1906.9105	1906.9135	-1.59	0	65	3.4e-007	1	U	K.LFTFHADICTLPDTEK.Q
4812	505 - 520	954.4625	1906.9105	1906.9135	-1.59	0	91	7.9e-010	1	U	K.LFTFHADICTLPDTEK.Q
4813	505 - 520	954.4628	1906.9110	1906.9135	-1.32	0	96	2.5e-010	1	U	K.LFTFHADICTLPDTEK.Q
4814	505 - 520	954.4628	1906.9111	1906.9135	-1.26	0	58	1.5e-006	1	U	K.LFTFHADICTLPDTEK.Q
4815	505 - 520	954.4629	1906.9113	1906.9135	-1.14	0	91	8.1e-010	1	U	K.LFTFHADICTLPDTEK.Q
4816	505 - 520	954.4630	1906.9115	1906.9135	-1.07	0	91	7.9e-010	1	U	K.LFTFHADICTLPDTEK.Q
4817	505 - 520	954.4630	1906.9115	1906.9135	-1.07	0	96	2.5e-010	1	U	K.LFTFHADICTLPDTEK.Q
4818	505 - 520	954.4633	1906.9120	1906.9135	-0.81	0	66	2.6e-007	1	U	K.LFTFHADICTLPDTEK.Q
4819	505 - 520	954.4633	1906.9120	1906.9135	-0.81	0	81	7.3e-009	1	U	K.LFTFHADICTLPDTEK.Q
4820	505 - 520	954.4633	1906.9121	1906.9135	-0.75	0	64	3.9e-007	1	U	K.LFTFHADICTLPDTEK.Q
4821	505 - 520	636.6447	1906.9123	1906.9135	-0.64	0	73	4.6e-008	1	U	K.LFTFHADICTLPDTEK.Q
4822	505 - 520	954.4634	1906.9123	1906.9135	-0.62	0	76	2.7e-008	1	U	K.LFTFHADICTLPDTEK.Q

Query	Start - End	Observed	Mr(expt)	Mr(calc)	ppm	M	Score	Expect	Rank	U	Peptide
4823	505 - 520	636.6448	1906.9127	1906.9135	-0.43	0	73	4.5e-008	1	U	K.LFTFHADICTLPDTEK.Q
4824	505 - 520	636.6448	1906.9127	1906.9135	-0.43	0	90	9.4e-010	1	U	K.LFTFHADICTLPDTEK.Q
4825	505 - 520	636.6449	1906.9129	1906.9135	-0.34	0	78	1.7e-008	1	U	K.LFTFHADICTLPDTEK.Q
4826	505 - 520	636.6449	1906.9129	1906.9135	-0.34	0	66	2.6e-007	1	U	K.LFTFHADICTLPDTEK.Q
4827	505 - 520	954.4638	1906.9129	1906.9135	-0.30	0	78	1.5e-008	1	U	K.LFTFHADICTLPDTEK.Q
4828	505 - 520	636.6450	1906.9131	1906.9135	-0.24	0	78	1.7e-008	1	U	K.LFTFHADICTLPDTEK.Q
4829	505 - 520	636.6450	1906.9132	1906.9135	-0.15	0	78	1.7e-008	1	U	K.LFTFHADICTLPDTEK.Q
4830	505 - 520	636.6450	1906.9132	1906.9135	-0.15	0	78	1.8e-008	1	U	K.LFTFHADICTLPDTEK.Q
4831	505 - 520	636.6451	1906.9134	1906.9135	-0.053	0	66	2.6e-007	1	U	K.LFTFHADICTLPDTEK.Q
4832	505 - 520	636.6451	1906.9134	1906.9135	-0.053	0	78	1.7e-008	1	U	K.LFTFHADICTLPDTEK.Q
4833	505 - 520	636.6451	1906.9134	1906.9135	-0.053	0	74	3.6e-008	1	U	K.LFTFHADICTLPDTEK.Q
4834	505 - 520	636.6451	1906.9134	1906.9135	-0.053	0	90	9.4e-010	1	U	K.LFTFHADICTLPDTEK.Q
4835	505 - 520	636.6451	1906.9136	1906.9135	0.041	0	74	3.6e-008	1	U	K.LFTFHADICTLPDTEK.Q
4836	505 - 520	636.6453	1906.9140	1906.9135	0.23	0	59	1.3e-006	1	U	K.LFTFHADICTLPDTEK.Q
4837	505 - 520	636.6453	1906.9140	1906.9135	0.23	0	66	2.7e-007	1	U	K.LFTFHADICTLPDTEK.Q
4838	505 - 520	636.6453	1906.9140	1906.9135	0.23	0	77	1.8e-008	1	U	K.LFTFHADICTLPDTEK.Q
4839	505 - 520	636.6454	1906.9143	1906.9135	0.43	0	78	1.7e-008	1	U	K.LFTFHADICTLPDTEK.Q
4840	505 - 520	636.6454	1906.9145	1906.9135	0.53	0	73	4.5e-008	1	U	K.LFTFHADICTLPDTEK.Q
4841	505 - 520	636.6458	1906.9156	1906.9135	1.10	0	78	1.7e-008	1	U	K.LFTFHADICTLPDTEK.Q
4842	505 - 520	636.6462	1906.9167	1906.9135	1.68	0	90	9.3e-010	1	U	K.LFTFHADICTLPDTEK.Q
4637	521 - 533	459.7698	1835.0501	1835.0503	-0.077	2	27	0.0022	1	U	K.QIKKQTALVELLK.H + 2 Glycation (K)
1591	524 - 533	571.8599	1141.7052	1141.7070	-1.63	1	66	2.3e-007	1	U	K.KQTALVELLK.H
1592	524 - 533	571.8599	1141.7052	1141.7070	-1.63	1	30	0.00092	1	U	K.KQTALVELLK.H
1593	524 - 533	571.8599	1141.7052	1141.7070	-1.63	1	24	0.004	1	U	K.KQTALVELLK.H
1594	524 - 533	571.8599	1141.7053	1141.7070	-1.53	1	56	2.5e-006	1	U	K.KQTALVELLK.H
1595	524 - 533	571.8601	1141.7055	1141.7070	-1.30	1	28	0.0015	1	U	K.KQTALVELLK.H
1596	524 - 533	571.8601	1141.7055	1141.7070	-1.30	1	28	0.0017	1	U	K.KQTALVELLK.H
1597	524 - 533	571.8603	1141.7060	1141.7070	-0.88	1	31	0.00077	1	U	K.KQTALVELLK.H
1598	524 - 533	381.5760	1141.7061	1141.7070	-0.86	1	38	0.00017	1	U	K.KQTALVELLK.H
1599	524 - 533	571.8605	1141.7064	1141.7070	-0.56	1	64	3.8e-007	1	U	K.KQTALVELLK.H
1600	524 - 533	381.5761	1141.7064	1141.7070	-0.54	1	36	0.00023	1	U	K.KQTALVELLK.H
1601	524 - 533	381.5761	1141.7064	1141.7070	-0.54	1	38	0.00016	1	U	K.KQTALVELLK.H
1602	524 - 533	571.8605	1141.7065	1141.7070	-0.46	1	27	0.002	1	U	K.KQTALVELLK.H
1603	524 - 533	571.8606	1141.7066	1141.7070	-0.34	1	64	3.7e-007	1	U	K.KQTALVELLK.H
1604	524 - 533	381.5762	1141.7067	1141.7070	-0.31	1	36	0.00023	1	U	K.KQTALVELLK.H
1605	524 - 533	381.5762	1141.7067	1141.7070	-0.31	1	40	0.0001	1	U	K.KQTALVELLK.H
1606	524 - 533	381.5762	1141.7067	1141.7070	-0.31	1	40	0.0001	1	U	K.KQTALVELLK.H
1607	524 - 533	571.8607	1141.7068	1141.7070	-0.23	1	22	0.007	1	U	K.KQTALVELLK.H
1608	524 - 533	381.5762	1141.7068	1141.7070	-0.23	1	40	0.0001	1	U	K.KQTALVELLK.H
1609	524 - 533	571.8607	1141.7069	1141.7070	-0.13	1	66	2.3e-007	1	U	K.KQTALVELLK.H
1610	524 - 533	571.8607	1141.7069	1141.7070	-0.13	1	17	0.022	1	U	K.KQTALVELLK.H
1613	524 - 533	381.5763	1141.7071	1141.7070	0.088	1	36	0.00025	1	U	K.KQTALVELLK.H
2088	524 - 533	652.8866	1303.7586	1303.7599	-0.93	1	24	0.0045	1	U	K.KQTALVELLK.H + Glycation (K)
2089	524 - 533	652.8866	1303.7586	1303.7599	-0.93	1	32	0.00061	1	U	K.KQTALVELLK.H + Glycation (K)
2090	524 - 533	652.8867	1303.7589	1303.7599	-0.74	1	40	0.0001	1	U	K.KQTALVELLK.H + Glycation (K)
2091	524 - 533	435.5937	1303.7592	1303.7599	-0.54	1	46	2.3e-005	1	U	K.KQTALVELLK.H + Glycation (K)
2092	524 - 533	652.8869	1303.7592	1303.7599	-0.47	1	40	0.00011	1	U	K.KQTALVELLK.H + Glycation (K)
2093	524 - 533	652.8869	1303.7592	1303.7599	-0.47	1	38	0.00016	1	U	K.KQTALVELLK.H + Glycation (K)
2094	524 - 533	652.8870	1303.7594	1303.7599	-0.38	1	38	0.00017	1	U	K.KQTALVELLK.H + Glycation (K)
2095	524 - 533	652.8870	1303.7595	1303.7599	-0.28	1	23	0.0051	1	U	K.KQTALVELLK.H + Glycation (K)
2096	524 - 533	652.8870	1303.7595	1303.7599	-0.28	1	24	0.0036	1	U	K.KQTALVELLK.H + Glycation (K)
2097	524 - 533	652.8870	1303.7595	1303.7599	-0.28	1	42	6.4e-005	1	U	K.KQTALVELLK.H + Glycation (K)
2098	524 - 533	652.8870	1303.7595	1303.7599	-0.28	1	26	0.0028	1	U	K.KQTALVELLK.H + Glycation (K)
2099	524 - 533	652.8870	1303.7595	1303.7599	-0.28	1	38	0.00016	1	U	K.KQTALVELLK.H + Glycation (K)
2100	524 - 533	652.8871	1303.7596	1303.7599	-0.19	1	16	0.024	1	U	K.KQTALVELLK.H + Glycation (K)
2101	524 - 533	652.8871	1303.7596	1303.7599	-0.19	1	40	9.6e-005	1	U	K.KQTALVELLK.H + Glycation (K)
2102	524 - 533	435.5938	1303.7597	1303.7599	-0.13	1	50	1e-005	1	U	K.KQTALVELLK.H + Glycation (K)
2103	524 - 533	435.5938	1303.7597	1303.7599	-0.13	1	44	3.6e-005	1	U	K.KQTALVELLK.H + Glycation (K)
2104	524 - 533	435.5938	1303.7597	1303.7599	-0.13	1	47	2.2e-005	1	U	K.KQTALVELLK.H + Glycation (K)
2105	524 - 533	652.8872	1303.7597	1303.7599	-0.084	1	26	0.0024	1	U	K.KQTALVELLK.H + Glycation (K)
2106	524 - 533	652.8872	1303.7597	1303.7599	-0.084	1	40	0.00011	1	U	K.KQTALVELLK.H + Glycation (K)
2107	524 - 533	435.5939	1303.7598	1303.7599	-0.058	1	47	2.2e-005	1	U	K.KQTALVELLK.H + Glycation (K)
2108	524 - 533	652.8872	1303.7599	1303.7599	0.0084	1	24	0.0036	1	U	K.KQTALVELLK.H + Glycation (K)
2109	524 - 533	435.5939	1303.7600	1303.7599	0.081	1	31	0.00078	1	U	K.KQTALVELLK.H + Glycation (K)
2110	524 - 533	435.5939	1303.7600	1303.7599	0.081	1	43	4.5e-005	1	U	K.KQTALVELLK.H + Glycation (K)
2111	524 - 533	652.8873	1303.7600	1303.7599	0.10	1	28	0.0015	1	U	K.KQTALVELLK.H + Glycation (K)
2112	524 - 533	435.5940	1303.7601	1303.7599	0.15	1	50	1e-005	1	U	K.KQTALVELLK.H + Glycation (K)
2113	524 - 533	652.8873	1303.7601	1303.7599	0.19	1	31	0.00074	1	U	K.KQTALVELLK.H + Glycation (K)
2114	524 - 533	435.5940	1303.7602	1303.7599	0.29	1	48	1.8e-005	1	U	K.KQTALVELLK.H + Glycation (K)
2115	524 - 533	435.5941	1303.7604	1303.7599	0.45	1	46	2.3e-005	1	U	K.KQTALVELLK.H + Glycation (K)
2116	524 - 533	652.8876	1303.7606	1303.7599	0.56	1	45	3.5e-005	1	U	K.KQTALVELLK.H + Glycation (K)
2117	524 - 533	435.5942	1303.7609	1303.7599	0.79	1	50	1e-005	1	U	K.KQTALVELLK.H + Glycation (K)
1088	525 - 533	507.8130	1013.6115	1013.6121	-0.58	0	34	0.0004	1	U	K.QTALVELLK.H
1089	525 - 533	507.8134	1013.6122	1013.6121	0.15	0	52	6.2e-006	1	U	K.QTALVELLK.H
1090	525 - 533	507.8134	1013.6122	1013.6121	0.15	0	50	1.1e-005	1	U	K.QTALVELLK.H
1091	525 - 533	507.8134	1013.6122	1013.6121	0.15	0	54	4.2e-006	1	U	K.QTALVELLK.H
1092	525 - 533	507.8134	1013.6123	1013.6121	0.21	0	46	2.7e-005	1	U	K.QTALVELLK.H
1093	525 - 533	507.8134	1013.6123	1013.6121	0.21	0	54	4.5e-006	1	U	K.QTALVELLK.H
1094	525 - 533	507.8134	1013.6123	1013.6121	0.21	0	53	4.5e-006	1	U	K.QTALVELLK.H
1095	525 - 533	507.8134	1013.6123	1013.6121	0.21	0	56	2.7e-006	1	U	K.QTALVELLK.H
1096	525 - 533	507.8134	1013.6123	1013.6121	0.21	0	54	4.2e-006	1	U	K.QTALVELLK.H
1097	525 - 533	507.8134	1013.6123	1013.6121	0.27	0	38	0.00017	1	U	K.QTALVELLK.H
1098	525 - 533	507.8134	1013.6123	1013.6121	0.27	0	54	4.2e-006	1	U	K.QTALVELLK.H
1099	525 - 533	507.8135	1013.6125	1013.6121	0.39	0	43	4.5e-005	1	U	K.QTALVELLK.H
1100	525 - 533	507.8137	1013.6129	1013.6121	0.80	0	49	1.3e-005	1	U	K.QTALVELLK.H
1101	525 - 533	507.8137	1013.6129	1013.6121	0.80	0	44	4.1e-005	1	U	K.QTALVELLK.H
1102	525 - 533	507.8138	1013.6129	1013.6121	0.86	0	46	2.7e-005	1	U	K.QTALVELLK.H
1103	525 - 533	507.8138	1013.6129	1013.6121	0.86	0	46	2.7e-005	1	U	K.QTALVELLK.H
1104	525 - 533	507.8138	1013.6131	1013.6121	0.98	0	50	9.5e-006	1	U	K.QTALVELLK.H

Query	Start - End	Observed	Mr(expt)	Mr(calc)	ppm	M	Score	Expect	Rank	U	Peptide
2541	545 - 556	700.3494	1398.6842	1398.6853	-0.83	0	85	3.4e-009	1	U	K.TVMENFVAFVDK.C
2542	545 - 556	700.3495	1398.6844	1398.6853	-0.66	0	50	1e-005	1	U	K.TVMENFVAFVDK.C
2543	545 - 556	700.3495	1398.6844	1398.6853	-0.66	0	50	1e-005	1	U	K.TVMENFVAFVDK.C
2544	545 - 556	700.3495	1398.6844	1398.6853	-0.66	0	85	3.2e-009	1	U	K.TVMENFVAFVDK.C
2545	545 - 556	700.3495	1398.6844	1398.6853	-0.66	0	85	3.3e-009	1	U	K.TVMENFVAFVDK.C
2546	545 - 556	700.3496	1398.6845	1398.6853	-0.57	0	53	4.5e-006	1	U	K.TVMENFVAFVDK.C
2547	545 - 556	700.3496	1398.6845	1398.6853	-0.57	0	71	7.6e-008	1	U	K.TVMENFVAFVDK.C
2548	545 - 556	700.3496	1398.6847	1398.6853	-0.49	0	85	3.3e-009	1	U	K.TVMENFVAFVDK.C
2549	545 - 556	700.3497	1398.6848	1398.6853	-0.40	0	85	3.4e-009	1	U	K.TVMENFVAFVDK.C
2550	545 - 556	700.3498	1398.6851	1398.6853	-0.14	0	71	8e-008	1	U	K.TVMENFVAFVDK.C
2551	545 - 556	700.3498	1398.6851	1398.6853	-0.14	0	53	4.7e-006	1	U	K.TVMENFVAFVDK.C
2552	545 - 556	467.2357	1398.6852	1398.6853	-0.077	0	64	3.9e-007	1	U	K.TVMENFVAFVDK.C
2553	545 - 556	467.2358	1398.6854	1398.6853	0.052	0	64	3.9e-007	1	U	K.TVMENFVAFVDK.C
2554	545 - 556	467.2358	1398.6854	1398.6853	0.052	0	77	1.8e-008	1	U	K.TVMENFVAFVDK.C
2555	545 - 556	700.3500	1398.6855	1398.6853	0.13	0	71	8.8e-008	1	U	K.TVMENFVAFVDK.C
4891	557 - 573	643.2676	1926.7811	1926.7910	-5.15	1	33	0.00051	1	U	K.CCAADDKEACFAVEGPK.L
4892	557 - 573	964.4014	1926.7883	1926.7910	-1.41	1	127	2.2e-013	1	U	K.CCAADDKEACFAVEGPK.L
4893	557 - 573	964.4017	1926.7889	1926.7910	-1.09	1	127	2.1e-013	1	U	K.CCAADDKEACFAVEGPK.L
4894	557 - 573	643.2703	1926.7891	1926.7910	-0.98	1	103	4.6e-011	1	U	K.CCAADDKEACFAVEGPK.L
4895	557 - 573	643.2703	1926.7891	1926.7910	-0.98	1	103	4.6e-011	1	U	K.CCAADDKEACFAVEGPK.L
4896	557 - 573	964.4019	1926.7893	1926.7910	-0.90	1	110	9.8e-012	1	U	K.CCAADDKEACFAVEGPK.L
4897	557 - 573	964.4019	1926.7893	1926.7910	-0.90	1	107	2e-011	1	U	K.CCAADDKEACFAVEGPK.L
4898	557 - 573	643.2704	1926.7893	1926.7910	-0.87	1	109	1.3e-011	1	U	K.CCAADDKEACFAVEGPK.L
4899	557 - 573	964.4020	1926.7894	1926.7910	-0.83	1	124	4.2e-013	1	U	K.CCAADDKEACFAVEGPK.L
4900	557 - 573	643.2704	1926.7895	1926.7910	-0.78	1	109	1.2e-011	1	U	K.CCAADDKEACFAVEGPK.L
4901	557 - 573	964.4021	1926.7896	1926.7910	-0.71	1	110	9.4e-012	1	U	K.CCAADDKEACFAVEGPK.L
4902	557 - 573	643.2705	1926.7897	1926.7910	-0.68	1	109	1.2e-011	1	U	K.CCAADDKEACFAVEGPK.L
4903	557 - 573	643.2706	1926.7899	1926.7910	-0.59	1	103	4.7e-011	1	U	K.CCAADDKEACFAVEGPK.L
4904	557 - 573	643.2706	1926.7899	1926.7910	-0.59	1	109	1.2e-011	1	U	K.CCAADDKEACFAVEGPK.L
4905	557 - 573	643.2706	1926.7901	1926.7910	-0.50	1	88	1.5e-009	1	U	K.CCAADDKEACFAVEGPK.L
4906	557 - 573	643.2706	1926.7901	1926.7910	-0.50	1	103	4.6e-011	1	U	K.CCAADDKEACFAVEGPK.L
4907	557 - 573	643.2706	1926.7901	1926.7910	-0.50	1	104	4.4e-011	1	U	K.CCAADDKEACFAVEGPK.L
4908	557 - 573	643.2707	1926.7902	1926.7910	-0.40	1	103	4.6e-011	1	U	K.CCAADDKEACFAVEGPK.L
4909	557 - 573	964.4025	1926.7905	1926.7910	-0.26	1	127	2.1e-013	1	U	K.CCAADDKEACFAVEGPK.L
4910	557 - 573	643.2708	1926.7906	1926.7910	-0.21	1	109	1.3e-011	1	U	K.CCAADDKEACFAVEGPK.L
4911	557 - 573	643.2708	1926.7906	1926.7910	-0.21	1	103	4.6e-011	1	U	K.CCAADDKEACFAVEGPK.L
4912	557 - 573	643.2709	1926.7910	1926.7910	-0.012	1	109	1.3e-011	1	U	K.CCAADDKEACFAVEGPK.L
4913	557 - 573	643.2712	1926.7917	1926.7910	0.36	1	103	4.6e-011	1	U	K.CCAADDKEACFAVEGPK.L
5297	557 - 573	697.2875	2088.8408	2088.8438	-1.46	1	64	3.6e-007	1	U	K.CCAADDKEACFAVEGPK.L + Glycation (K)
1450	564 - 573	554.2604	1106.5062	1106.5066	-0.39	0	86	2.6e-009	1	U	K.EACFAVEGPK.L
1451	564 - 573	554.2605	1106.5064	1106.5066	-0.17	0	81	8.7e-009	1	U	K.EACFAVEGPK.L
1452	564 - 573	554.2605	1106.5064	1106.5066	-0.17	0	85	3e-009	1	U	K.EACFAVEGPK.L
1453	564 - 573	554.2605	1106.5064	1106.5066	-0.17	0	81	8.6e-009	1	U	K.EACFAVEGPK.L
1454	564 - 573	554.2606	1106.5066	1106.5066	-0.063	0	86	2.6e-009	1	U	K.EACFAVEGPK.L
1455	564 - 573	554.2606	1106.5066	1106.5066	-0.063	0	86	2.6e-009	1	U	K.EACFAVEGPK.L
1456	564 - 573	554.2607	1106.5068	1106.5066	0.15	0	86	2.6e-009	1	U	K.EACFAVEGPK.L
1457	564 - 573	554.2607	1106.5068	1106.5066	0.15	0	86	2.6e-009	1	U	K.EACFAVEGPK.L
1458	564 - 573	554.2607	1106.5068	1106.5066	0.15	0	86	2.6e-009	1	U	K.EACFAVEGPK.L
1459	564 - 573	554.2607	1106.5068	1106.5066	0.15	0	81	8.7e-009	1	U	K.EACFAVEGPK.L
1460	564 - 573	554.2608	1106.5070	1106.5066	0.37	0	66	2.4e-007	1	U	K.EACFAVEGPK.L
1461	564 - 573	554.2608	1106.5070	1106.5066	0.37	0	86	2.7e-009	1	U	K.EACFAVEGPK.L
1462	564 - 573	554.2612	1106.5079	1106.5066	1.15	0	71	7.8e-008	1	U	K.EACFAVEGPK.L
1027	574 - 583	501.7950	1001.5755	1001.5757	-0.25	0	16	0.024	1	U	K.LVVSTQTALA.-
1029	574 - 583	501.7950	1001.5755	1001.5757	-0.25	0	15	0.03	1	U	K.LVVSTQTALA.-
1030	574 - 583	501.7951	1001.5756	1001.5757	-0.13	0	18	0.015	1	U	K.LVVSTQTALA.-
1031	574 - 583	501.7951	1001.5756	1001.5757	-0.13	0	22	0.0063	1	U	K.LVVSTQTALA.-
1032	574 - 583	501.7951	1001.5756	1001.5757	-0.13	0	18	0.016	1	U	K.LVVSTQTALA.-
1035	574 - 583	501.7951	1001.5756	1001.5757	-0.066	0	14	0.045	1	U	K.LVVSTQTALA.-
1037	574 - 583	501.7951	1001.5757	1001.5757	0.014	0	17	0.018	1	U	K.LVVSTQTALA.-
1039	574 - 583	501.7952	1001.5758	1001.5757	0.074	0	16	0.023	1	U	K.LVVSTQTALA.-
1040	574 - 583	501.7952	1001.5758	1001.5757	0.074	0	15	0.034	1	U	K.LVVSTQTALA.-
1041	574 - 583	501.7952	1001.5758	1001.5757	0.13	0	14	0.043	1	U	K.LVVSTQTALA.-
1042	574 - 583	501.7952	1001.5758	1001.5757	0.13	0	18	0.016	1	U	K.LVVSTQTALA.-
1043	574 - 583	501.7952	1001.5759	1001.5757	0.19	0	18	0.016	1	U	K.LVVSTQTALA.-
1045	574 - 583	501.7953	1001.5760	1001.5757	0.25	0	18	0.015	1	U	K.LVVSTQTALA.-
1046	574 - 583	501.7953	1001.5761	1001.5757	0.43	0	18	0.016	1	U	K.LVVSTQTALA.-
1047	574 - 583	501.7955	1001.5764	1001.5757	0.73	0	18	0.016	1	U	K.LVVSTQTALA.-
1048	574 - 583	501.7959	1001.5772	1001.5757	1.47	0	17	0.018	1	U	K.LVVSTQTALA.-
1049	574 - 583	501.7960	1001.5774	1001.5757	1.71	0	18	0.015	1	U	K.LVVSTQTALA.-

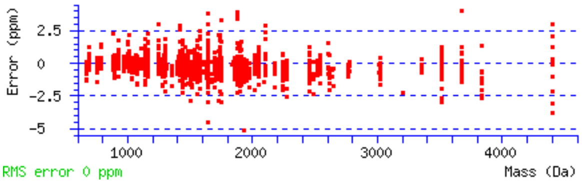

Mascot: http://www.matrixscience.com/

Appendix 2. Mascot search results of chymotrypsin digestion for BSA-1

MATRIX SCIENCE MASCOT Search Results

Protein View: >gi|00000

BSA

**Database:**	BSA
**Score:**	23609
**Monoisotopic mass (M_r):**	68386
**Calculated pI:**	5.60

Sequence similarity is available as **an NCBI BLAST search of >gi|00000 against nr**.

Search parameters

**MS data file:**	P2019021808.mgf
**Enzyme:**	Chymotrypsin: cuts C-term side of FLWY unless next residue is P.
**Fixed modifications:**	Carbamidomethyl (C)
**Variable modifications:**	Glycation (K), Glycation (Protein N-term)

Protein sequence coverage: 74%

Matched peptides shown in ***bold red***.

1 **DTHKSEIAHR** **FKDLGEEHFK** **GLVL**IAFSQY **LQQCPFDEHV** **KLVNELTEFA**
51 **KTCVADESHA** **GCEKSLHTLF** **GDELCKVASL** **RETYGDMADC** **CEKQEPERNE**
101 **CFLSHKDDSP** **DLPKLKPDPN** **TLCDEFKADE** **KKFW**GKYLYE IARRHPY**FYA**
151 **PELLY**YANKY **NGVFQECCQA** **EDKGACLLPK** **IETMREKVL**A SSARQRLRCA
201 SIQKFGERAL KAWSVARL**SQ** **KFPKAEFVEV** **TKLVTDLTKV** **HKECCHGDLL**
251 **ECADDRADLA** **KYICDNQDTI** **SSKLKECCDK** **PLLEKSHCIA** **EVEKDAIPEN**
301 **LPPLTADFAE** **DKDVCKNYQE** **AKDAFLGSFL** **Y**EYSRRHPEY AVSVL**LRLAK**
351 **EYEATLEECC** **AKDDPHACYS** **TVFDKLKHLV** **DEPQNLIKQN** **CDQFEKLGEY**
401 GFQNALIVRY **TRKVPQVSTP** **TLVEVSRSL**G KVGTRCCTKP ESERMPCTED
451 YLSLILNRLC VLHEKTPVSE KVTKCCTESL VNRRPCF**SAL** **TPDETYVPKA**
501 **FDEKLFTFHA** **DICTLPDTEK** **QIKKQTALVE** **LL**KHKPKATE EQL**KTVMENF**
551 **VAFVDKCCAA** **DDKEACFAVE** **GPKLVVSTQT** **ALA**

Unformatted sequence string: **583 residues** (for pasting into other applications).

Sort by ◉ residue number ○ increasing mass ○ decreasing mass
Show ◉ matched peptides only ○ predicted peptides also

Query	Start – End	Observed	Mr(expt)	Mr(calc)	ppm	M	Score	Expect	Rank	U	Peptide
2127	1 – 11	670.8379	1339.6613	1339.6633	-1.45	0	43	4.5e-005	1	U	-.DTHKSEIAHRF.K
2128	1 – 11	670.8381	1339.6616	1339.6633	-1.27	0	44	4.4e-005	1	U	-.DTHKSEIAHRF.K
2129	1 – 11	670.8381	1339.6616	1339.6633	-1.27	0	43	4.6e-005	1	U	-.DTHKSEIAHRF.K
2130	1 – 11	335.9227	1339.6617	1339.6633	-1.19	0	36	0.00026	1	U	-.DTHKSEIAHRF.K
2131	1 – 11	335.9227	1339.6617	1339.6633	-1.19	0	36	0.00024	1	U	-.DTHKSEIAHRF.K
2132	1 – 11	670.8381	1339.6617	1339.6633	-1.18	0	37	0.00018	1	U	-.DTHKSEIAHRF.K
2133	1 – 11	335.9228	1339.6619	1339.6633	-1.01	0	33	0.00053	1	U	-.DTHKSEIAHRF.K
2134	1 – 11	335.9228	1339.6619	1339.6633	-1.01	0	37	0.00022	1	U	-.DTHKSEIAHRF.K
2135	1 – 11	335.9228	1339.6621	1339.6633	-0.92	0	48	1.7e-005	1	U	-.DTHKSEIAHRF.K
2136	1 – 11	335.9228	1339.6621	1339.6633	-0.92	0	48	1.4e-005	1	U	-.DTHKSEIAHRF.K
2137	1 – 11	335.9228	1339.6621	1339.6633	-0.92	0	48	1.4e-005	1	U	-.DTHKSEIAHRF.K
2138	1 – 11	335.9228	1339.6622	1339.6633	-0.83	0	37	0.00021	1	U	-.DTHKSEIAHRF.K
2139	1 – 11	335.9228	1339.6622	1339.6633	-0.83	0	45	3.2e-005	1	U	-.DTHKSEIAHRF.K
2140	1 – 11	670.8384	1339.6622	1339.6633	-0.80	0	43	4.6e-005	1	U	-.DTHKSEIAHRF.K
2141	1 – 11	335.9228	1339.6623	1339.6633	-0.74	0	36	0.00023	1	U	-.DTHKSEIAHRF.K
2142	1 – 11	335.9228	1339.6623	1339.6633	-0.74	0	49	1.4e-005	1	U	-.DTHKSEIAHRF.K
2143	1 – 11	670.8384	1339.6623	1339.6633	-0.71	0	43	4.6e-005	1	U	-.DTHKSEIAHRF.K
2144	1 – 11	447.5614	1339.6624	1339.6633	-0.68	0	40	0.0001	1	U	-.DTHKSEIAHRF.K
2145	1 – 11	447.5614	1339.6624	1339.6633	-0.68	0	44	4.2e-005	1	U	-.DTHKSEIAHRF.K
2146	1 – 11	335.9229	1339.6624	1339.6633	-0.65	0	36	0.00024	1	U	-.DTHKSEIAHRF.K
2147	1 – 11	335.9229	1339.6624	1339.6633	-0.65	0	34	0.00043	1	U	-.DTHKSEIAHRF.K
2148	1 – 11	335.9229	1339.6624	1339.6633	-0.65	0	36	0.00024	1	U	-.DTHKSEIAHRF.K
2149	1 – 11	335.9229	1339.6624	1339.6633	-0.65	0	41	8.7e-005	1	U	-.DTHKSEIAHRF.K
2150	1 – 11	335.9229	1339.6624	1339.6633	-0.65	0	46	2.3e-005	1	U	-.DTHKSEIAHRF.K
2151	1 – 11	335.9229	1339.6624	1339.6633	-0.65	0	45	3.2e-005	1	U	-.DTHKSEIAHRF.K
2152	1 – 11	447.5614	1339.6625	1339.6633	-0.61	0	34	0.00044	1	U	-.DTHKSEIAHRF.K
2153	1 – 11	447.5614	1339.6625	1339.6633	-0.61	0	48	1.6e-005	1	U	-.DTHKSEIAHRF.K
2154	1 – 11	447.5614	1339.6625	1339.6633	-0.61	0	44	4.3e-005	1	U	-.DTHKSEIAHRF.K
2155	1 – 11	335.9229	1339.6625	1339.6633	-0.56	0	33	0.00051	1	U	-.DTHKSEIAHRF.K
2156	1 – 11	335.9229	1339.6625	1339.6633	-0.56	0	42	5.7e-005	1	U	-.DTHKSEIAHRF.K
2157	1 – 11	447.5615	1339.6626	1339.6633	-0.48	0	36	0.00025	1	U	-.DTHKSEIAHRF.K
2158	1 – 11	447.5615	1339.6626	1339.6633	-0.48	0	44	4.2e-005	1	U	-.DTHKSEIAHRF.K
2159	1 – 11	335.9229	1339.6627	1339.6633	-0.47	0	33	0.00049	1	U	-.DTHKSEIAHRF.K
2160	1 – 11	670.8386	1339.6627	1339.6633	-0.44	0	40	0.0001	1	U	-.DTHKSEIAHRF.K
2161	1 – 11	670.8386	1339.6627	1339.6633	-0.44	0	34	0.0004	1	U	-.DTHKSEIAHRF.K
2162	1 – 11	447.5615	1339.6627	1339.6633	-0.41	0	34	0.00041	1	U	-.DTHKSEIAHRF.K
2163	1 – 11	447.5615	1339.6627	1339.6633	-0.41	0	36	0.00025	1	U	-.DTHKSEIAHRF.K
2164	1 – 11	335.9230	1339.6628	1339.6633	-0.38	0	33	0.00052	1	U	-.DTHKSEIAHRF.K
2165	1 – 11	335.9230	1339.6628	1339.6633	-0.38	0	33	0.00053	1	U	-.DTHKSEIAHRF.K
2166	1 – 11	335.9230	1339.6628	1339.6633	-0.38	0	38	0.00015	1	U	-.DTHKSEIAHRF.K
2167	1 – 11	335.9230	1339.6628	1339.6633	-0.38	0	30	0.00094	1	U	-.DTHKSEIAHRF.K
2168	1 – 11	335.9230	1339.6628	1339.6633	-0.38	0	45	3.3e-005	1	U	-.DTHKSEIAHRF.K
2169	1 – 11	447.5616	1339.6628	1339.6633	-0.34	0	30	0.0011	1	U	-.DTHKSEIAHRF.K
2170	1 – 11	447.5616	1339.6628	1339.6633	-0.34	0	28	0.0014	1	U	-.DTHKSEIAHRF.K

Query	Start – End	Observed	Mr(expt)	Mr(calc)	ppm	M	Score	Expect	Rank	U	Peptide
2171	1 – 11	447.5616	1339.6628	1339.6633	-0.34	0	37	0.00019	1	U	-.DTHKSEIAHRF.K
2172	1 – 11	447.5616	1339.6628	1339.6633	-0.34	0	36	0.00026	1	U	-.DTHKSEIAHRF.K
2173	1 – 11	447.5616	1339.6628	1339.6633	-0.34	0	41	8.7e-005	1	U	-.DTHKSEIAHRF.K
2174	1 – 11	335.9230	1339.6629	1339.6633	-0.29	0	48	1.7e-005	1	U	-.DTHKSEIAHRF.K
2175	1 – 11	447.5616	1339.6629	1339.6633	-0.28	0	48	1.6e-005	1	U	-.DTHKSEIAHRF.K
2176	1 – 11	670.8388	1339.6629	1339.6633	-0.25	0	36	0.00024	1	U	-.DTHKSEIAHRF.K
2177	1 – 11	447.5616	1339.6630	1339.6633	-0.21	0	33	0.00052	1	U	-.DTHKSEIAHRF.K
2178	1 – 11	447.5616	1339.6630	1339.6633	-0.21	0	48	1.6e-005	1	U	-.DTHKSEIAHRF.K
2179	1 – 11	335.9230	1339.6630	1339.6633	-0.20	0	29	0.0013	1	U	-.DTHKSEIAHRF.K
2180	1 – 11	335.9230	1339.6630	1339.6633	-0.20	0	40	9.5e-005	1	U	-.DTHKSEIAHRF.K
2181	1 – 11	335.9230	1339.6630	1339.6633	-0.20	0	36	0.00025	1	U	-.DTHKSEIAHRF.K
2182	1 – 11	447.5616	1339.6631	1339.6633	-0.12	0	39	0.00011	1	U	-.DTHKSEIAHRF.K
2183	1 – 11	447.5616	1339.6631	1339.6633	-0.12	0	44	3.8e-005	1	U	-.DTHKSEIAHRF.K
2184	1 – 11	447.5616	1339.6631	1339.6633	-0.12	0	45	3.5e-005	1	U	-.DTHKSEIAHRF.K
2185	1 – 11	335.9231	1339.6632	1339.6633	-0.081	0	31	0.00079	1	U	-.DTHKSEIAHRF.K
2186	1 – 11	447.5617	1339.6632	1339.6633	-0.054	0	30	0.001	1	U	-.DTHKSEIAHRF.K
2187	1 – 11	447.5617	1339.6632	1339.6633	-0.054	0	44	4.2e-005	1	U	-.DTHKSEIAHRF.K
2188	1 – 11	447.5617	1339.6632	1339.6633	-0.054	0	44	4.3e-005	1	U	-.DTHKSEIAHRF.K
2189	1 – 11	335.9231	1339.6633	1339.6633	0.0090	0	23	0.0053	1	U	-.DTHKSEIAHRF.K
2190	1 – 11	447.5617	1339.6634	1339.6633	0.081	0	40	0.00011	1	U	-.DTHKSEIAHRF.K
2191	1 – 11	447.5617	1339.6634	1339.6633	0.081	0	36	0.00028	1	U	-.DTHKSEIAHRF.K
2192	1 – 11	335.9231	1339.6634	1339.6633	0.099	0	33	0.00052	1	U	-.DTHKSEIAHRF.K
2193	1 – 11	335.9231	1339.6634	1339.6633	0.099	0	45	3.2e-005	1	U	-.DTHKSEIAHRF.K
2194	1 – 11	447.5618	1339.6635	1339.6633	0.15	0	35	0.00029	1	U	-.DTHKSEIAHRF.K
2195	1 – 11	670.8391	1339.6635	1339.6633	0.20	0	44	4.4e-005	1	U	-.DTHKSEIAHRF.K
2196	1 – 11	670.8391	1339.6637	1339.6633	0.29	0	39	0.00014	1	U	-.DTHKSEIAHRF.K
2626	1 – 11	376.4361	1501.7154	1501.7161	-0.46	0	37	0.00022	1	U	-.DTHKSEIAHRF.K + Glycation (K)
2627	1 – 11	376.4361	1501.7154	1501.7161	-0.46	0	40	9.6e-005	1	U	-.DTHKSEIAHRF.K + Glycation (K)
2628	1 – 11	376.4361	1501.7154	1501.7161	-0.46	0	37	0.00021	1	U	-.DTHKSEIAHRF.K + Glycation (K)
2629	1 – 11	376.4362	1501.7157	1501.7161	-0.30	0	46	2.8e-005	1	U	-.DTHKSEIAHRF.K + Glycation (Protein N-t
2630	1 – 11	376.4362	1501.7159	1501.7161	-0.14	0	44	3.7e-005	1	U	-.DTHKSEIAHRF.K + Glycation (Protein N-t
2631	1 – 11	376.4363	1501.7163	1501.7161	0.097	0	35	0.00031	1	U	-.DTHKSEIAHRF.K + Glycation (K)
944	12 – 19	487.7322	973.4498	973.4505	-0.71	1	26	0.0025	1	U	F.KDLGEEHF.K
945	12 – 19	325.4906	973.4499	973.4505	-0.63	1	14	0.038	1	U	F.KDLGEEHF.K
947	12 – 19	325.4906	973.4500	973.4505	-0.54	1	19	0.012	1	U	F.KDLGEEHF.K
949	12 – 19	325.4907	973.4502	973.4505	-0.36	1	20	0.01	1	U	F.KDLGEEHF.K
950	12 – 19	487.7324	973.4502	973.4505	-0.34	1	34	0.0004	1	U	F.KDLGEEHF.K
951	12 – 19	487.7324	973.4502	973.4505	-0.34	1	24	0.0042	1	U	F.KDLGEEHF.K
952	12 – 19	487.7324	973.4502	973.4505	-0.34	1	28	0.0016	1	U	F.KDLGEEHF.K
953	12 – 19	487.7324	973.4502	973.4505	-0.28	1	29	0.0013	1	U	F.KDLGEEHF.K
954	12 – 19	487.7324	973.4502	973.4505	-0.28	1	30	0.0011	1	U	F.KDLGEEHF.K
955	12 – 19	487.7324	973.4503	973.4505	-0.22	1	25	0.0035	1	U	F.KDLGEEHF.K
956	12 – 19	487.7324	973.4503	973.4505	-0.22	1	24	0.0038	1	U	F.KDLGEEHF.K
957	12 – 19	325.4907	973.4503	973.4505	-0.17	1	22	0.0061	1	U	F.KDLGEEHF.K
958	12 – 19	487.7324	973.4503	973.4505	-0.16	1	26	0.0027	1	U	F.KDLGEEHF.K
959	12 – 19	487.7324	973.4503	973.4505	-0.16	1	29	0.0014	1	U	F.KDLGEEHF.K
960	12 – 19	487.7325	973.4505	973.4505	-0.033	1	25	0.0035	1	U	F.KDLGEEHF.K
961	12 – 19	487.7325	973.4505	973.4505	-0.033	1	29	0.0012	1	U	F.KDLGEEHF.K
964	12 – 19	487.7326	973.4507	973.4505	0.23	1	30	0.00099	1	U	F.KDLGEEHF.K
965	12 – 19	487.7327	973.4508	973.4505	0.30	1	24	0.0044	1	U	F.KDLGEEHF.K
966	12 – 19	487.7327	973.4508	973.4505	0.30	1	26	0.0026	1	U	F.KDLGEEHF.K
967	12 – 19	487.7327	973.4509	973.4505	0.42	1	30	0.00091	1	U	F.KDLGEEHF.K
968	12 – 19	487.7328	973.4511	973.4505	0.67	1	28	0.0015	1	U	F.KDLGEEHF.K
969	12 – 19	487.7329	973.4512	973.4505	0.73	1	38	0.00016	1	U	F.KDLGEEHF.K
1459	12 – 19	568.7587	1135.5029	1135.5033	-0.37	1	25	0.0034	1	U	F.KDLGEEHF.K + Glycation (K)
1460	12 – 19	568.7589	1135.5031	1135.5033	-0.16	1	27	0.0018	1	U	F.KDLGEEHF.K + Glycation (K)
1462	12 – 19	568.7590	1135.5035	1135.5033	0.16	1	27	0.0018	1	U	F.KDLGEEHF.K + Glycation (K)
1463	12 – 19	568.7590	1135.5035	1135.5033	0.16	1	25	0.0032	1	U	F.KDLGEEHF.K + Glycation (K)
1464	12 – 19	568.7592	1135.5038	1135.5033	0.39	1	25	0.0035	1	U	F.KDLGEEHF.K + Glycation (K)
1904	12 – 22	636.8321	1271.6496	1271.6510	-1.07	2	31	0.00077	1	U	F.KDLGEEHFKGL.V
1905	12 – 22	636.8321	1271.6497	1271.6510	-0.97	2	31	0.00076	1	U	F.KDLGEEHFKGL.V
1906	12 – 22	424.8906	1271.6499	1271.6510	-0.82	2	14	0.042	1	U	F.KDLGEEHFKGL.V
1907	12 – 22	424.8906	1271.6499	1271.6510	-0.82	2	15	0.028	1	U	F.KDLGEEHFKGL.V
1909	12 – 22	636.8323	1271.6500	1271.6510	-0.77	2	31	0.00076	1	U	F.KDLGEEHFKGL.V
1910	12 – 22	424.8906	1271.6501	1271.6510	-0.73	2	19	0.012	1	U	F.KDLGEEHFKGL.V
1911	12 – 22	424.8907	1271.6502	1271.6510	-0.59	2	18	0.017	1	U	F.KDLGEEHFKGL.V
1913	12 – 22	424.8907	1271.6502	1271.6510	-0.59	2	21	0.0086	1	U	F.KDLGEEHFKGL.V
1914	12 – 22	636.8324	1271.6502	1271.6510	-0.58	2	31	0.00076	1	U	F.KDLGEEHFKGL.V
1915	12 – 22	636.8324	1271.6502	1271.6510	-0.58	2	31	0.00076	1	U	F.KDLGEEHFKGL.V
1916	12 – 22	424.8907	1271.6503	1271.6510	-0.51	2	17	0.021	1	U	F.KDLGEEHFKGL.V
1918	12 – 22	424.8908	1271.6504	1271.6510	-0.44	2	13	0.049	1	U	F.KDLGEEHFKGL.V
1919	12 – 22	424.8908	1271.6504	1271.6510	-0.44	2	15	0.031	1	U	F.KDLGEEHFKGL.V
1922	12 – 22	636.8325	1271.6505	1271.6510	-0.39	2	31	0.00077	1	U	F.KDLGEEHFKGL.V
1923	12 – 22	424.8908	1271.6505	1271.6510	-0.37	2	13	0.047	1	U	F.KDLGEEHFKGL.V
1924	12 – 22	424.8908	1271.6505	1271.6510	-0.37	2	14	0.044	1	U	F.KDLGEEHFKGL.V
1927	12 – 22	636.8326	1271.6506	1271.6510	-0.30	2	31	0.00075	1	U	F.KDLGEEHFKGL.V
1928	12 – 22	424.8908	1271.6507	1271.6510	-0.23	2	22	0.0063	1	U	F.KDLGEEHFKGL.V
1929	12 – 22	424.8908	1271.6507	1271.6510	-0.23	2	25	0.003	1	U	F.KDLGEEHFKGL.V
1930	12 – 22	424.8908	1271.6507	1271.6510	-0.23	2	13	0.045	1	U	F.KDLGEEHFKGL.V
1932	12 – 22	424.8908	1271.6507	1271.6510	-0.23	2	22	0.0071	1	U	F.KDLGEEHFKGL.V
1933	12 – 22	424.8908	1271.6507	1271.6510	-0.23	2	16	0.025	1	U	F.KDLGEEHFKGL.V
1935	12 – 22	636.8326	1271.6507	1271.6510	-0.20	2	31	0.00077	1	U	F.KDLGEEHFKGL.V
1936	12 – 22	636.8326	1271.6507	1271.6510	-0.20	2	31	0.00076	1	U	F.KDLGEEHFKGL.V
1937	12 – 22	636.8326	1271.6507	1271.6510	-0.20	2	28	0.0016	1	U	F.KDLGEEHFKGL.V
1938	12 – 22	636.8326	1271.6507	1271.6510	-0.20	2	31	0.00076	1	U	F.KDLGEEHFKGL.V
1939	12 – 22	424.8909	1271.6508	1271.6510	-0.16	2	20	0.011	1	U	F.KDLGEEHFKGL.V
1940	12 – 22	424.8909	1271.6508	1271.6510	-0.16	2	19	0.013	1	U	F.KDLGEEHFKGL.V
1941	12 – 22	424.8909	1271.6508	1271.6510	-0.16	2	16	0.027	1	U	F.KDLGEEHFKGL.V
1944	12 – 22	424.8909	1271.6510	1271.6510	-0.019	2	23	0.0055	1	U	F.KDLGEEHFKGL.V
1945	12 – 22	636.8328	1271.6510	1271.6510	-0.014	2	34	0.00043	1	U	F.KDLGEEHFKGL.V

Query	Start – End	Observed	Mr(expt)	Mr(calc)	ppm	M	Score	Expect	Rank	U	Peptide
1946	12 – 22	636.8328	1271.6510	1271.6510	-0.014	2	31	0.00077	1	U	F.KDLGEEHFKGL.V
1948	12 – 22	636.8328	1271.6511	1271.6510	0.080	2	31	0.00073	1	U	F.KDLGEEHFKGL.V
1949	12 – 22	424.8911	1271.6513	1271.6510	0.26	2	18	0.017	1	U	F.KDLGEEHFKGL.V
1950	12 – 22	424.8911	1271.6513	1271.6510	0.26	2	16	0.027	1	U	F.KDLGEEHFKGL.V
1951	12 – 22	636.8329	1271.6513	1271.6510	0.28	2	30	0.00098	1	U	F.KDLGEEHFKGL.V
1954	12 – 22	424.8914	1271.6523	1271.6510	1.07	2	15	0.033	1	U	F.KDLGEEHFKGL.V
1956	12 – 22	424.8917	1271.6532	1271.6510	1.70	2	18	0.017	1	U	F.KDLGEEHFKGL.V
2486	12 – 22	717.8571	1433.6996	1433.7038	-2.96	2	15	0.032	1	U	F.KDLGEEHFKGL.V + Glycation (K)
2488	12 – 22	717.8585	1433.7024	1433.7038	-1.01	2	28	0.0016	1	U	F.KDLGEEHFKGL.V + Glycation (K)
2489	12 – 22	717.8585	1433.7024	1433.7038	-1.01	2	26	0.0025	1	U	F.KDLGEEHFKGL.V + Glycation (K)
2490	12 – 22	717.8585	1433.7025	1433.7038	-0.92	2	28	0.0017	1	U	F.KDLGEEHFKGL.V + Glycation (K)
2491	12 – 22	717.8586	1433.7027	1433.7038	-0.76	2	28	0.0015	1	U	F.KDLGEEHFKGL.V + Glycation (K)
2492	12 – 22	478.9083	1433.7030	1433.7038	-0.60	2	26	0.0027	1	U	F.KDLGEEHFKGL.V + Glycation (K)
2493	12 – 22	717.8588	1433.7031	1433.7038	-0.49	2	25	0.0031	1	U	F.KDLGEEHFKGL.V + Glycation (K)
2494	12 – 22	478.9083	1433.7032	1433.7038	-0.45	2	34	0.00042	1	U	F.KDLGEEHFKGL.V + Glycation (K)
2495	12 – 22	478.9084	1433.7033	1433.7038	-0.39	2	40	0.00011	1	U	F.KDLGEEHFKGL.V + Glycation (K)
2496	12 – 22	478.9084	1433.7033	1433.7038	-0.33	2	18	0.017	1	U	F.KDLGEEHFKGL.V + Glycation (K)
2497	12 – 22	717.8590	1433.7033	1433.7038	-0.32	2	26	0.0025	1	U	F.KDLGEEHFKGL.V + Glycation (K)
2498	12 – 22	478.9085	1433.7035	1433.7038	-0.20	2	35	0.00029	1	U	F.KDLGEEHFKGL.V + Glycation (K)
2499	12 – 22	478.9085	1433.7035	1433.7038	-0.20	2	39	0.00011	1	U	F.KDLGEEHFKGL.V + Glycation (K)
2500	12 – 22	478.9085	1433.7036	1433.7038	-0.14	2	27	0.0021	1	U	F.KDLGEEHFKGL.V + Glycation (K)
2501	12 – 22	478.9085	1433.7036	1433.7038	-0.14	2	44	4.4e-005	1	U	F.KDLGEEHFKGL.V + Glycation (K)
2502	12 – 22	478.9086	1433.7041	1433.7038	0.17	2	34	0.0004	1	U	F.KDLGEEHFKGL.V + Glycation (K)
2589	12 – 24	742.9085	1483.8023	1483.8035	-0.75	3	45	3.5e-005	1	U	F.KDLGEEHFKGLVL.I
2591	12 – 24	742.9086	1483.8027	1483.8035	-0.51	3	34	0.00044	1	U	F.KDLGEEHFKGLVL.I
2592	12 – 24	742.9086	1483.8027	1483.8035	-0.51	3	39	0.00014	1	U	F.KDLGEEHFKGLVL.I
2593	12 – 24	742.9086	1483.8027	1483.8035	-0.51	3	46	2.6e-005	1	U	F.KDLGEEHFKGLVL.I
2595	12 – 24	742.9087	1483.8028	1483.8035	-0.43	3	42	7e-005	1	U	F.KDLGEEHFKGLVL.I
2599	12 – 24	495.6084	1483.8033	1483.8035	-0.12	3	20	0.011	1	U	F.KDLGEEHFKGLVL.I
2600	12 – 24	742.9089	1483.8033	1483.8035	-0.094	3	38	0.00015	1	U	F.KDLGEEHFKGLVL.I
2601	12 – 24	495.6084	1483.8034	1483.8035	-0.064	3	24	0.004	1	U	F.KDLGEEHFKGLVL.I
2603	12 – 24	495.6084	1483.8035	1483.8035	-0.0034	3	29	0.0013	1	U	F.KDLGEEHFKGLVL.I
2604	12 – 24	495.6085	1483.8036	1483.8035	0.057	3	17	0.021	1	U	F.KDLGEEHFKGLVL.I
2605	12 – 24	495.6085	1483.8036	1483.8035	0.12	3	15	0.03	1	U	F.KDLGEEHFKGLVL.I
2606	12 – 24	495.6085	1483.8036	1483.8035	0.12	3	29	0.0013	1	U	F.KDLGEEHFKGLVL.I
828	15 – 22	458.7299	915.4452	915.4450	0.16	1	22	0.0069	1	U	L.GEEHFKGL.V
829	15 – 22	458.7299	915.4453	915.4450	0.36	1	14	0.041	1	U	L.GEEHFKGL.V
20	20 – 24	265.1889	528.3632	528.3635	-0.64	1	13	0.048	1	U	F.KGLVL.I
22	20 – 24	265.1890	528.3634	528.3635	-0.30	1	15	0.033	1	U	F.KGLVL.I
23	20 – 24	265.1890	528.3634	528.3635	-0.30	1	18	0.014	1	U	F.KGLVL.I
454	31 – 36	396.6889	791.3633	791.3636	-0.39	1	35	0.00034	1	U	Y.LQQCPF.D
455	31 – 36	396.6890	791.3634	791.3636	-0.21	1	40	0.00011	1	U	Y.LQQCPF.D
456	31 – 36	396.6890	791.3634	791.3636	-0.21	1	30	0.00096	1	U	Y.LQQCPF.D
457	31 – 36	396.6890	791.3635	791.3636	-0.14	1	40	0.00011	1	U	Y.LQQCPF.D
458	31 – 36	396.6891	791.3636	791.3636	0.014	1	35	0.00033	1	U	Y.LQQCPF.D
459	31 – 36	396.6891	791.3637	791.3636	0.090	1	19	0.013	1	U	Y.LQQCPF.D
460	31 – 36	396.6891	791.3637	791.3636	0.090	1	20	0.01	1	U	Y.LQQCPF.D
461	31 – 36	396.6891	791.3637	791.3636	0.090	1	30	0.00097	1	U	Y.LQQCPF.D
462	31 – 36	396.6891	791.3637	791.3636	0.17	1	18	0.017	1	U	Y.LQQCPF.D
2684	31 – 42	505.2529	1512.7368	1512.7395	-1.80	2	22	0.0066	1	U	Y.LQQCPFDEHVKL.V
2685	31 – 42	757.3763	1512.7380	1512.7395	-0.99	2	25	0.0035	1	U	Y.LQQCPFDEHVKL.V
2686	31 – 42	757.3763	1512.7380	1512.7395	-0.99	2	26	0.0026	1	U	Y.LQQCPFDEHVKL.V
2687	31 – 42	757.3763	1512.7381	1512.7395	-0.91	2	27	0.0021	1	U	Y.LQQCPFDEHVKL.V
2688	31 – 42	757.3763	1512.7381	1512.7395	-0.91	2	27	0.0022	1	U	Y.LQQCPFDEHVKL.V
2689	31 – 42	757.3764	1512.7382	1512.7395	-0.83	2	29	0.0012	1	U	Y.LQQCPFDEHVKL.V
2690	31 – 42	757.3765	1512.7384	1512.7395	-0.75	2	24	0.0043	1	U	Y.LQQCPFDEHVKL.V
2691	31 – 42	757.3765	1512.7384	1512.7395	-0.75	2	25	0.0034	1	U	Y.LQQCPFDEHVKL.V
2692	31 – 42	757.3765	1512.7384	1512.7395	-0.75	2	28	0.0016	1	U	Y.LQQCPFDEHVKL.V
2693	31 – 42	757.3765	1512.7384	1512.7395	-0.75	2	27	0.0019	1	U	Y.LQQCPFDEHVKL.V
2694	31 – 42	757.3766	1512.7386	1512.7395	-0.58	2	30	0.00099	1	U	Y.LQQCPFDEHVKL.V
2695	31 – 42	757.3766	1512.7386	1512.7395	-0.58	2	27	0.0019	1	U	Y.LQQCPFDEHVKL.V
2696	31 – 42	757.3767	1512.7389	1512.7395	-0.42	2	26	0.0024	1	U	Y.LQQCPFDEHVKL.V
2697	31 – 42	505.2536	1512.7390	1512.7395	-0.35	2	21	0.0085	1	U	Y.LQQCPFDEHVKL.V
2698	31 – 42	505.2537	1512.7393	1512.7395	-0.11	2	26	0.0023	1	U	Y.LQQCPFDEHVKL.V
2699	31 – 42	505.2537	1512.7393	1512.7395	-0.11	2	25	0.003	1	U	Y.LQQCPFDEHVKL.V
2700	31 – 42	505.2537	1512.7393	1512.7395	-0.11	2	25	0.0032	1	U	Y.LQQCPFDEHVKL.V
2701	31 – 42	505.2538	1512.7394	1512.7395	-0.053	2	22	0.007	1	U	Y.LQQCPFDEHVKL.V
2702	31 – 42	505.2538	1512.7394	1512.7395	-0.053	2	25	0.0032	1	U	Y.LQQCPFDEHVKL.V
2703	31 – 42	757.3770	1512.7395	1512.7395	-0.022	2	29	0.0012	1	U	Y.LQQCPFDEHVKL.V
2704	31 – 42	505.2538	1512.7395	1512.7395	0.0066	2	21	0.0078	1	U	Y.LQQCPFDEHVKL.V
2705	31 – 42	505.2538	1512.7396	1512.7395	0.066	2	19	0.012	1	U	Y.LQQCPFDEHVKL.V
2706	31 – 42	505.2538	1512.7396	1512.7395	0.066	2	24	0.0044	1	U	Y.LQQCPFDEHVKL.V
2707	31 – 42	505.2538	1512.7396	1512.7395	0.066	2	22	0.0067	1	U	Y.LQQCPFDEHVKL.V
2708	31 – 42	505.2538	1512.7396	1512.7395	0.066	2	24	0.0037	1	U	Y.LQQCPFDEHVKL.V
2709	31 – 42	505.2539	1512.7397	1512.7395	0.15	2	26	0.0027	1	U	Y.LQQCPFDEHVKL.V
2710	31 – 42	505.2539	1512.7400	1512.7395	0.32	2	19	0.012	1	U	Y.LQQCPFDEHVKL.V
2711	31 – 42	505.2539	1512.7400	1512.7395	0.32	2	19	0.012	1	U	Y.LQQCPFDEHVKL.V
2712	31 – 42	505.2539	1512.7400	1512.7395	0.32	2	20	0.011	1	U	Y.LQQCPFDEHVKL.V
2713	31 – 42	505.2540	1512.7403	1512.7395	0.50	2	25	0.0031	1	U	Y.LQQCPFDEHVKL.V
3172	31 – 42	838.4027	1674.7909	1674.7923	-0.87	2	22	0.006	1	U	Y.LQQCPFDEHVKL.V + Glycation (K)
3173	31 – 42	838.4029	1674.7912	1674.7923	-0.66	2	27	0.0022	1	U	Y.LQQCPFDEHVKL.V + Glycation (K)
3174	31 – 42	838.4030	1674.7915	1674.7923	-0.50	2	22	0.006	1	U	Y.LQQCPFDEHVKL.V + Glycation (K)
4529	31 – 49	1173.5737	2345.1329	2345.1362	-1.40	4	29	0.0012	1	U	Y.LQQCPFDEHVKLVNELTEF.A
4531	31 – 49	1173.5739	2345.1331	2345.1362	-1.30	4	29	0.0012	1	U	Y.LQQCPFDEHVKLVNELTEF.A
4540	31 – 49	1173.5747	2345.1349	2345.1362	-0.56	4	29	0.0012	1	U	Y.LQQCPFDEHVKLVNELTEF.A
4542	31 – 49	1173.5753	2345.1361	2345.1362	-0.041	4	36	0.00024	1	U	Y.LQQCPFDEHVKLVNELTEF.A
4543	31 – 49	1173.5756	2345.1366	2345.1362	0.16	4	29	0.0014	1	U	Y.LQQCPFDEHVKLVNELTEF.A
4544	31 – 49	1173.5758	2345.1371	2345.1362	0.38	4	30	0.0011	1	U	Y.LQQCPFDEHVKLVNELTEF.A
2929	37 – 49	786.8976	1571.7807	1571.7831	-1.53	2	70	1e-007	1	U	F.DEHVKLVNELTEF.A
2930	37 – 49	786.8981	1571.7817	1571.7831	-0.90	2	96	2.6e-010	1	U	F.DEHVKLVNELTEF.A

Query	Start – End	Observed	Mr(expt)	Mr(calc)	ppm	M	Score	Expect	Rank	U	Peptide
2931	37 – 49	786.8982	1571.7818	1571.7831	-0.83	2	91	7.5e-010	1	U	F.DEHVKLVNELTEF.A
2932	37 – 49	786.8983	1571.7821	1571.7831	-0.66	2	82	5.7e-009	1	U	F.DEHVKLVNELTEF.A
2933	37 – 49	786.8984	1571.7822	1571.7831	-0.58	2	78	1.5e-008	1	U	F.DEHVKLVNELTEF.A
2934	37 – 49	786.8984	1571.7823	1571.7831	-0.51	2	70	1e-007	1	U	F.DEHVKLVNELTEF.A
2935	37 – 49	786.8984	1571.7823	1571.7831	-0.51	2	51	8.6e-006	1	U	F.DEHVKLVNELTEF.A
2936	37 – 49	524.9351	1571.7834	1571.7831	0.14	2	64	4.2e-007	1	U	F.DEHVKLVNELTEF.A
2937	37 – 49	524.9351	1571.7835	1571.7831	0.26	2	70	1.1e-007	1	U	F.DEHVKLVNELTEF.A
2938	37 – 49	524.9351	1571.7835	1571.7831	0.26	2	64	4.1e-007	1	U	F.DEHVKLVNELTEF.A
2939	37 – 49	524.9351	1571.7835	1571.7831	0.26	2	59	1.2e-006	1	U	F.DEHVKLVNELTEF.A
2940	37 – 49	524.9352	1571.7837	1571.7831	0.37	2	76	2.4e-008	1	U	F.DEHVKLVNELTEF.A
2941	37 – 49	524.9353	1571.7841	1571.7831	0.60	2	57	1.9e-006	1	U	F.DEHVKLVNELTEF.A
626	43 – 49	426.2108	850.4070	850.4072	-0.28	1	15	0.033	1	U	L.VNELTEF.A
627	43 – 49	426.2108	850.4070	850.4072	-0.28	1	17	0.02	1	U	L.VNELTEF.A
630	43 – 49	426.2108	850.4071	850.4072	-0.18	1	15	0.031	1	U	L.VNELTEF.A
3586	50 – 66	621.6168	1861.8286	1861.8298	-0.65	0	32	0.00068	1	U	F.AKTCVADESHAGCEKSL.H
3588	50 – 66	621.6179	1861.8318	1861.8298	1.03	0	36	0.00023	1	U	F.AKTCVADESHAGCEKSL.H
4556	50 – 70	787.7042	2360.0908	2360.0889	0.81	2	13	0.047	1	U	F.AKTCVADESHAGCEKSLHTLF.G
1363	71 – 80	546.2738	1090.5330	1090.5329	0.18	1	44	3.6e-005	1	U	F.GDELCKVASL.R
1364	71 – 80	546.2739	1090.5332	1090.5329	0.29	1	45	3.5e-005	1	U	F.GDELCKVASL.R
1365	71 – 80	546.2740	1090.5335	1090.5329	0.64	1	24	0.0038	1	U	F.GDELCKVASL.R
1366	71 – 80	546.2742	1090.5339	1090.5329	0.97	1	28	0.0016	1	U	F.GDELCKVASL.R
198	75 – 80	339.1862	676.3579	676.3578	0.15	0	25	0.0029	1	U	L.CKVASL.R
199	75 – 80	339.1867	676.3588	676.3578	1.51	0	25	0.003	1	U	L.CKVASL.R
4910	81 – 102	942.0399	2823.0979	2823.0993	-0.48	1	63	4.7e-007	1	U	L.RETYGDMADCCEKQEPERNECF.L
4911	81 – 102	706.7820	2823.0991	2823.0993	-0.077	1	70	1e-007	1	U	L.RETYGDMADCCEKQEPERNECF.L
4912	81 – 102	706.7820	2823.0991	2823.0993	-0.077	1	71	7.7e-008	1	U	L.RETYGDMADCCEKQEPERNECF.L
4913	81 – 102	706.7822	2823.0998	2823.0993	0.19	1	74	3.9e-008	1	U	L.RETYGDMADCCEKQEPERNECF.L
4914	81 – 102	942.0407	2823.1003	2823.0993	0.36	1	66	2.6e-007	1	U	L.RETYGDMADCCEKQEPERNECF.L
4404	85 – 102	758.9549	2273.8429	2273.8446	-0.75	0	38	0.00015	1	U	Y.GDMADCCEKQEPERNECF.L
4405	85 – 102	758.9551	2273.8436	2273.8446	-0.43	0	59	1.3e-006	1	U	Y.GDMADCCEKQEPERNECF.L
4406	85 – 102	758.9551	2273.8436	2273.8446	-0.43	0	55	2.9e-006	1	U	Y.GDMADCCEKQEPERNECF.L
4407	85 – 102	758.9552	2273.8438	2273.8446	-0.35	0	21	0.0077	1	U	Y.GDMADCCEKQEPERNECF.L
4408	85 – 102	758.9552	2273.8438	2273.8446	-0.35	0	59	1.4e-006	1	U	Y.GDMADCCEKQEPERNECF.L
4409	85 – 102	758.9554	2273.8443	2273.8446	-0.12	0	40	0.0001	1	U	Y.GDMADCCEKQEPERNECF.L
4410	85 – 102	758.9554	2273.8445	2273.8446	-0.036	0	34	0.00035	1	U	Y.GDMADCCEKQEPERNECF.L
4411	85 – 102	758.9554	2273.8445	2273.8446	-0.036	0	63	5.2e-007	1	U	Y.GDMADCCEKQEPERNECF.L
4412	85 – 102	1137.9296	2273.8446	2273.8446	0.0062	0	68	1.6e-007	1	U	Y.GDMADCCEKQEPERNECF.L
4413	85 – 102	758.9555	2273.8447	2273.8446	0.056	0	59	1.4e-006	1	U	Y.GDMADCCEKQEPERNECF.L
4414	85 – 102	1137.9298	2273.8451	2273.8446	0.22	0	62	6.8e-007	1	U	Y.GDMADCCEKQEPERNECF.L
4415	85 – 102	1137.9303	2273.8460	2273.8446	0.65	0	69	1.3e-007	1	U	Y.GDMADCCEKQEPERNECF.L
4416	85 – 102	1137.9305	2273.8465	2273.8446	0.86	0	66	2.7e-007	1	U	Y.GDMADCCEKQEPERNECF.L
4417	85 – 102	1137.9325	2273.8504	2273.8446	2.58	0	21	0.0072	1	U	Y.GDMADCCEKQEPERNECF.L
4588	85 – 103	796.6498	2386.9277	2386.9286	-0.40	1	17	0.022	1	U	Y.GDMADCCEKQEPERNECFL.S
4589	85 – 103	796.6500	2386.9282	2386.9286	-0.17	1	21	0.0074	1	U	Y.GDMADCCEKQEPERNECFL.S
4329	103 – 122	744.0613	2229.1620	2229.1641	-0.93	2	24	0.0041	1	U	F.LSHKDDSPDLPKLKPDPNTL.C
4330	103 – 122	558.2979	2229.1625	2229.1641	-0.70	2	16	0.023	1	U	F.LSHKDDSPDLPKLKPDPNTL.C
4331	103 – 122	558.2982	2229.1638	2229.1641	-0.14	2	18	0.017	1	U	F.LSHKDDSPDLPKLKPDPNTL.C
4332	103 – 122	744.0619	2229.1638	2229.1641	-0.11	2	29	0.0011	1	U	F.LSHKDDSPDLPKLKPDPNTL.C
4333	103 – 122	558.2983	2229.1640	2229.1641	-0.035	2	17	0.022	1	U	F.LSHKDDSPDLPKLKPDPNTL.C
4334	103 – 122	558.2983	2229.1640	2229.1641	-0.035	2	15	0.032	1	U	F.LSHKDDSPDLPKLKPDPNTL.C
4335	103 – 122	744.0620	2229.1642	2229.1641	0.049	2	29	0.0013	1	U	F.LSHKDDSPDLPKLKPDPNTL.C
4336	103 – 122	744.0620	2229.1642	2229.1641	0.049	2	24	0.0037	1	U	F.LSHKDDSPDLPKLKPDPNTL.C
4831	103 – 126	927.7836	2780.3289	2780.3327	-1.37	3	33	0.00054	1	U	F.LSHKDDSPDLPKLKPDPNTLCDEF.K
4836	103 – 126	927.7842	2780.3307	2780.3327	-0.72	3	44	3.6e-005	1	U	F.LSHKDDSPDLPKLKPDPNTLCDEF.K
4837	103 – 126	927.7842	2780.3307	2780.3327	-0.72	3	42	6.7e-005	1	U	F.LSHKDDSPDLPKLKPDPNTLCDEF.K
4838	103 – 126	927.7842	2780.3309	2780.3327	-0.65	3	37	0.0002	1	U	F.LSHKDDSPDLPKLKPDPNTLCDEF.K
4839	103 – 126	927.7842	2780.3309	2780.3327	-0.65	3	34	0.0004	1	U	F.LSHKDDSPDLPKLKPDPNTLCDEF.K
4840	103 – 126	927.7842	2780.3309	2780.3327	-0.65	3	34	0.00043	1	U	F.LSHKDDSPDLPKLKPDPNTLCDEF.K
4841	103 – 126	696.0900	2780.3310	2780.3327	-0.61	3	19	0.011	1	U	F.LSHKDDSPDLPKLKPDPNTLCDEF.K
4842	103 – 126	927.7843	2780.3311	2780.3327	-0.59	3	35	0.00029	1	U	F.LSHKDDSPDLPKLKPDPNTLCDEF.K
4843	103 – 126	927.7844	2780.3313	2780.3327	-0.52	3	39	0.00014	1	U	F.LSHKDDSPDLPKLKPDPNTLCDEF.K
4844	103 – 126	927.7844	2780.3313	2780.3327	-0.52	3	34	0.00042	1	U	F.LSHKDDSPDLPKLKPDPNTLCDEF.K
4845	103 – 126	927.7844	2780.3313	2780.3327	-0.52	3	34	0.00043	1	U	F.LSHKDDSPDLPKLKPDPNTLCDEF.K
4848	103 – 126	696.0901	2780.3315	2780.3327	-0.43	3	13	0.048	1	U	F.LSHKDDSPDLPKLKPDPNTLCDEF.K
4849	103 – 126	696.0901	2780.3315	2780.3327	-0.43	3	14	0.044	1	U	F.LSHKDDSPDLPKLKPDPNTLCDEF.K
4851	103 – 126	557.0736	2780.3317	2780.3327	-0.37	3	14	0.037	1	U	F.LSHKDDSPDLPKLKPDPNTLCDEF.K
4852	103 – 126	696.0902	2780.3317	2780.3327	-0.35	3	26	0.0026	1	U	F.LSHKDDSPDLPKLKPDPNTLCDEF.K
4854	103 – 126	557.0737	2780.3323	2780.3327	-0.16	3	14	0.038	1	U	F.LSHKDDSPDLPKLKPDPNTLCDEF.K
4855	103 – 126	557.0737	2780.3323	2780.3327	-0.16	3	29	0.0013	1	U	F.LSHKDDSPDLPKLKPDPNTLCDEF.K
4856	103 – 126	927.7847	2780.3324	2780.3327	-0.12	3	35	0.0003	1	U	F.LSHKDDSPDLPKLKPDPNTLCDEF.K
4858	103 – 126	557.0738	2780.3326	2780.3327	-0.048	3	31	0.00086	1	U	F.LSHKDDSPDLPKLKPDPNTLCDEF.K
4860	103 – 126	557.0738	2780.3329	2780.3327	0.060	3	28	0.0015	1	U	F.LSHKDDSPDLPKLKPDPNTLCDEF.K
4863	103 – 126	696.0906	2780.3335	2780.3327	0.27	3	16	0.024	1	U	F.LSHKDDSPDLPKLKPDPNTLCDEF.K
4865	103 – 126	557.0740	2780.3338	2780.3327	0.40	3	22	0.0057	1	U	F.LSHKDDSPDLPKLKPDPNTLCDEF.K
4866	103 – 126	557.0740	2780.3338	2780.3327	0.40	3	23	0.0051	1	U	F.LSHKDDSPDLPKLKPDPNTLCDEF.K
4868	103 – 126	557.0741	2780.3341	2780.3327	0.51	3	21	0.0082	1	U	F.LSHKDDSPDLPKLKPDPNTLCDEF.K
4869	103 – 126	696.0908	2780.3342	2780.3327	0.53	3	16	0.024	1	U	F.LSHKDDSPDLPKLKPDPNTLCDEF.K
4870	103 – 126	1391.1746	2780.3346	2780.3327	0.67	3	53	4.6e-006	1	U	F.LSHKDDSPDLPKLKPDPNTLCDEF.K
4871	103 – 126	557.0742	2780.3347	2780.3327	0.73	3	16	0.024	1	U	F.LSHKDDSPDLPKLKPDPNTLCDEF.K
4872	103 – 126	557.0742	2780.3347	2780.3327	0.73	3	20	0.01	1	U	F.LSHKDDSPDLPKLKPDPNTLCDEF.K
4873	103 – 126	1391.1754	2780.3363	2780.3327	1.29	3	50	9e-006	1	U	F.LSHKDDSPDLPKLKPDPNTLCDEF.K
4874	103 – 126	696.0914	2780.3364	2780.3327	1.32	3	26	0.0025	1	U	F.LSHKDDSPDLPKLKPDPNTLCDEF.K
4875	103 – 126	696.0914	2780.3366	2780.3327	1.41	3	23	0.0046	1	U	F.LSHKDDSPDLPKLKPDPNTLCDEF.K
5037	103 – 126	736.6028	2942.3823	2942.3855	-1.11	3	25	0.003	1	U	F.LSHKDDSPDLPKLKPDPNTLCDEF.K + Glycati
5038	103 – 126	736.6029	2942.3825	2942.3855	-1.02	3	23	0.005	1	U	F.LSHKDDSPDLPKLKPDPNTLCDEF.K + Glycati
5039	103 – 126	981.8016	2942.3829	2942.3855	-0.90	3	29	0.0012	1	U	F.LSHKDDSPDLPKLKPDPNTLCDEF.K + Glycati
5040	103 – 126	981.8016	2942.3829	2942.3855	-0.90	3	34	0.00043	1	U	F.LSHKDDSPDLPKLKPDPNTLCDEF.K + Glycati
5041	103 – 126	736.6032	2942.3835	2942.3855	-0.69	3	24	0.0038	1	U	F.LSHKDDSPDLPKLKPDPNTLCDEF.K + Glycati
5042	103 – 126	736.6034	2942.3845	2942.3855	-0.36	3	14	0.043	1	U	F.LSHKDDSPDLPKLKPDPNTLCDEF.K + Glycati
5043	103 – 126	736.6034	2942.3847	2942.3855	-0.28	3	14	0.037	1	U	F.LSHKDDSPDLPKLKPDPNTLCDEF.K + Glycati

Query	Start – End	Observed	Mr(expt)	Mr(calc)	ppm	M	Score	Expect	Rank	U	Peptide
5044	103 – 126	589.4842	2942.3849	2942.3855	-0.22	3	31	0.00085	1	U	F.LSHKDDSPDLPKLKPDPNTLCDEF.K + Glycati
5045	103 – 126	981.8025	2942.3856	2942.3855	0.038	3	32	0.00063	1	U	F.LSHKDDSPDLPKLKPDPNTLCDEF.K + Glycati
5416	103 – 133	907.7030	3626.7829	3626.7926	-2.68	4	40	9.3e-005	1	U	F.LSHKDDSPDLPKLKPDPNTLCDEFKADEKKF.W
5417	103 – 133	605.4725	3626.7912	3626.7926	-0.41	4	20	0.0093	1	U	F.LSHKDDSPDLPKLKPDPNTLCDEFKADEKKF.W
5418	103 – 133	726.3659	3626.7930	3626.7926	0.11	4	26	0.0027	1	U	F.LSHKDDSPDLPKLKPDPNTLCDEFKADEKKF.W
5419	103 – 133	907.7057	3626.7939	3626.7926	0.35	4	41	8.6e-005	1	U	F.LSHKDDSPDLPKLKPDPNTLCDEFKADEKKF.W
5420	103 – 133	605.4730	3626.7941	3626.7926	0.41	4	19	0.013	1	U	F.LSHKDDSPDLPKLKPDPNTLCDEFKADEKKF.W
5421	103 – 133	907.7064	3626.7963	3626.7926	1.02	4	42	6.3e-005	1	U	F.LSHKDDSPDLPKLKPDPNTLCDEFKADEKKF.W
5422	103 – 133	726.3668	3626.7974	3626.7926	1.32	4	30	0.00093	1	U	F.LSHKDDSPDLPKLKPDPNTLCDEFKADEKKF.W
5423	103 – 133	726.3676	3626.8014	3626.7926	2.41	4	25	0.0033	1	U	F.LSHKDDSPDLPKLKPDPNTLCDEFKADEKKF.W
5424	103 – 133	726.3690	3626.8084	3626.7926	4.35	4	30	0.0011	1	U	F.LSHKDDSPDLPKLKPDPNTLCDEFKADEKKF.W
5425	103 – 133	726.3708	3626.8176	3626.7926	6.88	4	14	0.037	1	U	F.LSHKDDSPDLPKLKPDPNTLCDEFKADEKKF.W
2204	104 – 115	451.2331	1350.6775	1350.6779	-0.30	0	17	0.02	1	U	L.SHKDDSPDLPKL.K
2205	104 – 115	451.2332	1350.6776	1350.6779	-0.23	0	15	0.034	1	U	L.SHKDDSPDLPKL.K
2210	104 – 115	451.2333	1350.6780	1350.6779	0.058	0	13	0.046	1	U	L.SHKDDSPDLPKL.K
4122	104 – 122	530.0284	2116.0847	2116.0800	2.19	1	19	0.014	1	U	L.SHKDDSPDLPKLKPDPNTL.C
4752	104 – 126	1334.6285	2667.2425	2667.2486	-2.29	2	55	2.9e-006	1	U	L.SHKDDSPDLPKLKPDPNTLCDEF.K
4753	104 – 126	890.0888	2667.2444	2667.2486	-1.58	2	42	6.3e-005	1	U	L.SHKDDSPDLPKLKPDPNTLCDEF.K
4754	104 – 126	890.0894	2667.2463	2667.2486	-0.90	2	38	0.00018	1	U	L.SHKDDSPDLPKLKPDPNTLCDEF.K
4755	104 – 126	1334.6305	2667.2464	2667.2486	-0.83	2	55	2.9e-006	1	U	L.SHKDDSPDLPKLKPDPNTLCDEF.K
4756	104 – 126	890.0895	2667.2468	2667.2486	-0.69	2	39	0.00013	1	U	L.SHKDDSPDLPKLKPDPNTLCDEF.K
4757	104 – 126	890.0895	2667.2468	2667.2486	-0.69	2	35	0.0003	1	U	L.SHKDDSPDLPKLKPDPNTLCDEF.K
4758	104 – 126	890.0896	2667.2470	2667.2486	-0.63	2	31	0.00085	1	U	L.SHKDDSPDLPKLKPDPNTLCDEF.K
4759	104 – 126	667.8191	2667.2473	2667.2486	-0.52	2	25	0.0032	1	U	L.SHKDDSPDLPKLKPDPNTLCDEF.K
4760	104 – 126	890.0899	2667.2479	2667.2486	-0.29	2	32	0.00059	1	U	L.SHKDDSPDLPKLKPDPNTLCDEF.K
4761	104 – 126	667.8193	2667.2483	2667.2486	-0.14	2	22	0.0063	1	U	L.SHKDDSPDLPKLKPDPNTLCDEF.K
4762	104 – 126	667.8193	2667.2483	2667.2486	-0.14	2	40	0.0001	1	U	L.SHKDDSPDLPKLKPDPNTLCDEF.K
4763	104 – 126	890.0900	2667.2483	2667.2486	-0.14	2	26	0.0023	1	U	L.SHKDDSPDLPKLKPDPNTLCDEF.K
4764	104 – 126	890.0901	2667.2484	2667.2486	-0.075	2	40	9e-005	1	U	L.SHKDDSPDLPKLKPDPNTLCDEF.K
4765	104 – 126	667.8195	2667.2487	2667.2486	0.035	2	33	0.00055	1	U	L.SHKDDSPDLPKLKPDPNTLCDEF.K
4766	104 – 126	667.8196	2667.2495	2667.2486	0.31	2	27	0.0021	1	U	L.SHKDDSPDLPKLKPDPNTLCDEF.K
4767	104 – 126	667.8197	2667.2497	2667.2486	0.40	2	42	5.7e-005	1	U	L.SHKDDSPDLPKLKPDPNTLCDEF.K
4768	104 – 126	667.8197	2667.2497	2667.2486	0.40	2	52	6.2e-006	1	U	L.SHKDDSPDLPKLKPDPNTLCDEF.K
4769	104 – 126	667.8199	2667.2507	2667.2486	0.77	2	31	0.00078	1	U	L.SHKDDSPDLPKLKPDPNTLCDEF.K
4770	104 – 126	890.0914	2667.2523	2667.2486	1.36	2	26	0.0025	1	U	L.SHKDDSPDLPKLKPDPNTLCDEF.K
4771	104 – 126	667.8212	2667.2556	2667.2486	2.60	2	28	0.0017	1	U	L.SHKDDSPDLPKLKPDPNTLCDEF.K
4929	104 – 126	708.3334	2829.3044	2829.3015	1.03	2	17	0.021	1	U	L.SHKDDSPDLPKLKPDPNTLCDEF.K + Glycation
5449	104 – 134	617.6404	3699.7986	3699.7879	2.90	4	27	0.0019	1	U	L.SHKDDSPDLPKLKPDPNTLCDEFKADEKKFW.G
5450	104 – 134	925.9571	3699.7993	3699.7879	3.07	4	37	0.00019	1	U	L.SHKDDSPDLPKLKPDPNTLCDEFKADEKKFW.G
3022	123 – 134	401.4369	1601.7183	1601.7184	-0.049	2	30	0.00094	1	U	L.CDEFKADEKKFW.G
3024	123 – 134	534.9136	1601.7191	1601.7184	0.42	2	39	0.00013	1	U	L.CDEFKADEKKFW.G
3025	123 – 134	801.8669	1601.7192	1601.7184	0.50	2	46	2.5e-005	1	U	L.CDEFKADEKKFW.G
3026	123 – 134	801.8672	1601.7198	1601.7184	0.88	2	42	5.7e-005	1	U	L.CDEFKADEKKFW.G
1148	127 – 134	351.1902	1050.5487	1050.5498	-1.10	1	23	0.0047	1	U	F.KADEKKFW.G
1150	127 – 134	351.1903	1050.5490	1050.5498	-0.75	1	15	0.032	1	U	F.KADEKKFW.G
1152	127 – 134	351.1903	1050.5490	1050.5498	-0.75	1	20	0.011	1	U	F.KADEKKFW.G
1153	127 – 134	526.2818	1050.5490	1050.5498	-0.72	1	37	0.00019	1	U	F.KADEKKFW.G
1154	127 – 134	526.2819	1050.5492	1050.5498	-0.61	1	39	0.00013	1	U	F.KADEKKFW.G
1158	127 – 134	351.1904	1050.5493	1050.5498	-0.50	1	17	0.021	1	U	F.KADEKKFW.G
1160	127 – 134	526.2819	1050.5493	1050.5498	-0.49	1	33	0.00054	1	U	F.KADEKKFW.G
1161	127 – 134	351.1904	1050.5494	1050.5498	-0.41	1	20	0.0098	1	U	F.KADEKKFW.G
1162	127 – 134	351.1904	1050.5494	1050.5498	-0.41	1	24	0.0042	1	U	F.KADEKKFW.G
1163	127 – 134	351.1904	1050.5494	1050.5498	-0.41	1	18	0.014	1	U	F.KADEKKFW.G
1165	127 – 134	351.1904	1050.5495	1050.5498	-0.33	1	19	0.011	1	U	F.KADEKKFW.G
1166	127 – 134	351.1905	1050.5496	1050.5498	-0.24	1	25	0.0032	1	U	F.KADEKKFW.G
1167	127 – 134	351.1905	1050.5496	1050.5498	-0.24	1	25	0.0032	1	U	F.KADEKKFW.G
1168	127 – 134	351.1905	1050.5496	1050.5498	-0.24	1	25	0.0034	1	U	F.KADEKKFW.G
1171	127 – 134	526.2821	1050.5496	1050.5498	-0.15	1	39	0.00013	1	U	F.KADEKKFW.G
1172	127 – 134	526.2821	1050.5496	1050.5498	-0.15	1	36	0.00025	1	U	F.KADEKKFW.G
1173	127 – 134	351.1905	1050.5497	1050.5498	-0.069	1	25	0.0033	1	U	F.KADEKKFW.G
1174	127 – 134	351.1905	1050.5497	1050.5498	-0.069	1	25	0.0029	1	U	F.KADEKKFW.G
1175	127 – 134	526.2822	1050.5498	1050.5498	-0.016	1	37	0.00018	1	U	F.KADEKKFW.G
1176	127 – 134	526.2822	1050.5498	1050.5498	-0.016	1	39	0.00012	1	U	F.KADEKKFW.G
1177	127 – 134	526.2822	1050.5498	1050.5498	-0.016	1	38	0.00017	1	U	F.KADEKKFW.G
1178	127 – 134	526.2822	1050.5499	1050.5498	0.098	1	15	0.028	1	U	F.KADEKKFW.G
1179	127 – 134	526.2822	1050.5499	1050.5498	0.098	1	39	0.00014	1	U	F.KADEKKFW.G
1180	127 – 134	526.2822	1050.5499	1050.5498	0.098	1	39	0.00012	1	U	F.KADEKKFW.G
1181	127 – 134	351.1906	1050.5499	1050.5498	0.10	1	25	0.003	1	U	F.KADEKKFW.G
1182	127 – 134	526.2823	1050.5500	1050.5498	0.21	1	42	6.3e-005	1	U	F.KADEKKFW.G
1183	127 – 134	526.2823	1050.5500	1050.5498	0.21	1	39	0.00012	1	U	F.KADEKKFW.G
1184	127 – 134	526.2823	1050.5500	1050.5498	0.21	1	39	0.00012	1	U	F.KADEKKFW.G
1185	127 – 134	526.2823	1050.5501	1050.5498	0.33	1	39	0.00013	1	U	F.KADEKKFW.G
1186	127 – 134	526.2823	1050.5501	1050.5498	0.33	1	39	0.00012	1	U	F.KADEKKFW.G
1187	127 – 134	526.2825	1050.5504	1050.5498	0.55	1	38	0.00017	1	U	F.KADEKKFW.G
1697	127 – 134	405.2080	1212.6023	1212.6026	-0.28	1	29	0.0012	1	U	F.KADEKKFW.G + Glycation (K)
1698	127 – 134	405.2081	1212.6024	1212.6026	-0.21	1	24	0.0039	1	U	F.KADEKKFW.G + Glycation (K)
1699	127 – 134	405.2081	1212.6025	1212.6026	-0.13	1	23	0.005	1	U	F.KADEKKFW.G + Glycation (K)
1700	127 – 134	405.2081	1212.6026	1212.6026	-0.057	1	26	0.0026	1	U	F.KADEKKFW.G + Glycation (K)
1701	127 – 134	607.3086	1212.6027	1212.6026	0.096	1	22	0.0063	1	U	F.KADEKKFW.G + Glycation (K)
1702	127 – 134	405.2082	1212.6028	1212.6026	0.17	1	28	0.0016	1	U	F.KADEKKFW.G + Glycation (K)
1703	127 – 134	405.2082	1212.6028	1212.6026	0.17	1	16	0.026	1	U	F.KADEKKFW.G + Glycation (K)
1705	127 – 134	607.3088	1212.6030	1212.6026	0.31	1	32	0.00064	1	U	F.KADEKKFW.G + Glycation (K)
1706	127 – 134	607.3090	1212.6034	1212.6026	0.61	1	27	0.0021	1	U	F.KADEKKFW.G + Glycation (K)
1707	127 – 134	607.3091	1212.6037	1212.6026	0.90	1	29	0.0011	1	U	F.KADEKKFW.G + Glycation (K)
1710	127 – 134	607.3097	1212.6048	1212.6026	1.81	1	20	0.011	1	U	F.KADEKKFW.G + Glycation (K)
1711	127 – 134	607.3108	1212.6070	1212.6026	3.63	1	20	0.01	1	U	F.KADEKKFW.G + Glycation (K)
634	148 – 154	426.7287	851.4428	851.4429	-0.12	3	29	0.0012	1	U	Y.FYAPELL.Y
635	148 – 154	426.7287	851.4428	851.4429	-0.045	3	21	0.0082	1	U	Y.FYAPELL.Y
636	148 – 154	426.7287	851.4429	851.4429	0.026	3	29	0.0012	1	U	Y.FYAPELL.Y
637	148 – 154	426.7288	851.4430	851.4429	0.096	3	29	0.0012	1	U	Y.FYAPELL.Y

Query	Start - End	Observed	Mr(expt)	Mr(calc)	ppm	M	Score	Expect	Rank	U	Peptide
638	148 - 154	426.7288	851.4430	851.4429	0.096	3	21	0.0084	1	U	Y.FYAPELL.Y
1051	148 - 155	508.2606	1014.5066	1014.5062	0.35	4	14	0.037	1	U	Y.FYAPELLY.Y
3753	161 - 177	662.6094	1984.8063	1984.8077	-0.72	1	21	0.0082	1	U	Y.NGVFQECCQAEDKGACL.L
3754	161 - 177	993.4105	1984.8065	1984.8077	-0.63	1	75	3.5e-008	1	U	Y.NGVFQECCQAEDKGACL.L
3755	161 - 177	993.4106	1984.8067	1984.8077	-0.51	1	74	3.7e-008	1	U	Y.NGVFQECCQAEDKGACL.L
3756	161 - 177	662.6096	1984.8070	1984.8077	-0.36	1	29	0.0013	1	U	Y.NGVFQECCQAEDKGACL.L
3757	161 - 177	993.4108	1984.8071	1984.8077	-0.32	1	56	2.7e-006	1	U	Y.NGVFQECCQAEDKGACL.L
3758	161 - 177	993.4108	1984.8071	1984.8077	-0.32	1	79	1.3e-008	1	U	Y.NGVFQECCQAEDKGACL.L
3759	161 - 177	993.4113	1984.8079	1984.8077	0.11	1	74	3.7e-008	1	U	Y.NGVFQECCQAEDKGACL.L
3760	161 - 177	662.6101	1984.8083	1984.8077	0.29	1	33	0.00046	1	U	Y.NGVFQECCQAEDKGACL.L
5303	161 - 189	856.6684	3422.6445	3422.6455	-0.28	2	24	0.0039	1	U	Y.NGVFQECCQAEDKGACLLPKIETMREKVL.A
5304	161 - 189	685.5364	3422.6455	3422.6455	0.017	2	29	0.0014	1	U	Y.NGVFQECCQAEDKGACLLPKIETMREKVL.A
5305	161 - 189	685.5364	3422.6455	3422.6455	0.017	2	27	0.0021	1	U	Y.NGVFQECCQAEDKGACLLPKIETMREKVL.A
5306	161 - 189	856.6688	3422.6459	3422.6455	0.14	2	36	0.00028	1	U	Y.NGVFQECCQAEDKGACLLPKIETMREKVL.A
5307	161 - 189	856.6696	3422.6494	3422.6455	1.14	2	26	0.0027	1	U	Y.NGVFQECCQAEDKGACLLPKIETMREKVL.A
5308	161 - 189	685.5374	3422.6507	3422.6455	1.52	2	27	0.0021	1	U	Y.NGVFQECCQAEDKGACLLPKIETMREKVL.A
2890	165 - 177	784.8096	1567.6047	1567.6065	-1.15	0	62	5.9e-007	1	U	F.QECCQAEDKGACL.L
2891	165 - 177	784.8100	1567.6054	1567.6065	-0.67	0	51	8.1e-006	1	U	F.QECCQAEDKGACL.L
2892	165 - 177	784.8113	1567.6080	1567.6065	0.96	0	46	2.5e-005	1	U	F.QECCQAEDKGACL.L
5095	165 - 189	602.0961	3005.4440	3005.4442	-0.086	1	14	0.038	1	U	F.QECCQAEDKGACLLPKIETMREKVL.A
1285	219 - 227	541.2861	1080.5577	1080.5604	-2.47	0	22	0.0056	1	U	L.SQKFPKAEF.V
1286	219 - 227	541.2867	1080.5589	1080.5604	-1.34	0	26	0.0027	1	U	L.SQKFPKAEF.V
1287	219 - 227	361.1936	1080.5590	1080.5604	-1.30	0	13	0.048	1	U	L.SQKFPKAEF.V
1288	219 - 227	541.2869	1080.5592	1080.5604	-1.10	0	18	0.015	1	U	L.SQKFPKAEF.V
1289	219 - 227	361.1937	1080.5593	1080.5604	-1.02	0	17	0.022	1	U	L.SQKFPKAEF.V
1290	219 - 227	541.2869	1080.5593	1080.5604	-0.99	0	19	0.012	1	U	L.SQKFPKAEF.V
1294	219 - 227	541.2871	1080.5595	1080.5604	-0.77	0	23	0.0054	1	U	L.SQKFPKAEF.V
1296	219 - 227	541.2871	1080.5597	1080.5604	-0.66	0	24	0.0038	1	U	L.SQKFPKAEF.V
1297	219 - 227	541.2871	1080.5597	1080.5604	-0.66	0	23	0.0046	1	U	L.SQKFPKAEF.V
1300	219 - 227	541.2872	1080.5598	1080.5604	-0.55	0	22	0.0065	1	U	L.SQKFPKAEF.V
1307	219 - 227	541.2872	1080.5599	1080.5604	-0.44	0	25	0.0034	1	U	L.SQKFPKAEF.V
1308	219 - 227	541.2872	1080.5599	1080.5604	-0.44	0	22	0.0064	1	U	L.SQKFPKAEF.V
1315	219 - 227	541.2873	1080.5601	1080.5604	-0.21	0	25	0.0035	1	U	L.SQKFPKAEF.V
1316	219 - 227	541.2873	1080.5601	1080.5604	-0.21	0	22	0.0069	1	U	L.SQKFPKAEF.V
1317	219 - 227	541.2873	1080.5601	1080.5604	-0.21	0	22	0.0069	1	U	L.SQKFPKAEF.V
1318	219 - 227	541.2873	1080.5601	1080.5604	-0.21	0	22	0.0058	1	U	L.SQKFPKAEF.V
1321	219 - 227	361.1940	1080.5602	1080.5604	-0.19	0	17	0.02	1	U	L.SQKFPKAEF.V
1324	219 - 227	541.2875	1080.5604	1080.5604	0.027	0	22	0.007	1	U	L.SQKFPKAEF.V
1325	219 - 227	541.2875	1080.5604	1080.5604	0.027	0	23	0.005	1	U	L.SQKFPKAEF.V
1326	219 - 227	541.2875	1080.5604	1080.5604	0.027	0	26	0.0024	1	U	L.SQKFPKAEF.V
1327	219 - 227	541.2875	1080.5604	1080.5604	0.027	0	26	0.0025	1	U	L.SQKFPKAEF.V
1328	219 - 227	541.2875	1080.5604	1080.5604	0.027	0	22	0.0058	1	U	L.SQKFPKAEF.V
1329	219 - 227	541.2875	1080.5605	1080.5604	0.14	0	27	0.0019	1	U	L.SQKFPKAEF.V
1330	219 - 227	541.2875	1080.5605	1080.5604	0.14	0	23	0.0046	1	U	L.SQKFPKAEF.V
1333	219 - 227	541.2876	1080.5606	1080.5604	0.25	0	23	0.0051	1	U	L.SQKFPKAEF.V
1334	219 - 227	541.2876	1080.5606	1080.5604	0.25	0	22	0.0059	1	U	L.SQKFPKAEF.V
1336	219 - 227	541.2877	1080.5608	1080.5604	0.36	0	22	0.0058	1	U	L.SQKFPKAEF.V
1337	219 - 227	541.2877	1080.5608	1080.5604	0.36	0	23	0.0049	1	U	L.SQKFPKAEF.V
1338	219 - 227	541.2877	1080.5608	1080.5604	0.36	0	20	0.0097	1	U	L.SQKFPKAEF.V
1339	219 - 227	541.2877	1080.5608	1080.5604	0.36	0	22	0.0064	1	U	L.SQKFPKAEF.V
1340	219 - 227	541.2877	1080.5609	1080.5604	0.47	0	25	0.0035	1	U	L.SQKFPKAEF.V
1341	219 - 227	541.2877	1080.5609	1080.5604	0.47	0	22	0.0057	1	U	L.SQKFPKAEF.V
1342	219 - 227	541.2878	1080.5610	1080.5604	0.58	0	22	0.0059	1	U	L.SQKFPKAEF.V
1777	219 - 227	622.3136	1242.6126	1242.6132	-0.45	0	18	0.017	1	U	L.SQKFPKAEF.V + Glycation (K)
1778	219 - 227	622.3140	1242.6134	1242.6132	0.13	0	14	0.041	1	U	L.SQKFPKAEF.V + Glycation (K)
1779	219 - 227	415.2118	1242.6135	1242.6132	0.27	0	18	0.017	1	U	L.SQKFPKAEF.V + Glycation (K)
1780	219 - 227	415.2118	1242.6136	1242.6132	0.34	0	26	0.0024	1	U	L.SQKFPKAEF.V + Glycation (K)
1781	219 - 227	622.3142	1242.6137	1242.6132	0.44	0	25	0.0032	1	U	L.SQKFPKAEF.V + Glycation (K)
1782	219 - 227	622.3143	1242.6141	1242.6132	0.73	0	30	0.001	1	U	L.SQKFPKAEF.V + Glycation (K)
1783	219 - 227	415.2120	1242.6141	1242.6132	0.73	0	26	0.0024	1	U	L.SQKFPKAEF.V + Glycation (K)
1784	219 - 227	415.2126	1242.6160	1242.6132	2.28	0	19	0.013	1	U	L.SQKFPKAEF.V + Glycation (K)
3350	219 - 233	438.4989	1749.9664	1749.9665	-0.075	1	31	0.00071	1	U	L.SQKFPKAEFVEVTKL.V
3351	219 - 233	584.3294	1749.9664	1749.9665	-0.060	1	49	1.4e-005	1	U	L.SQKFPKAEFVEVTKL.V
3352	219 - 233	584.3294	1749.9664	1749.9665	-0.060	1	56	2.4e-006	1	U	L.SQKFPKAEFVEVTKL.V
3353	219 - 233	584.3295	1749.9666	1749.9665	0.043	1	31	0.00083	1	U	L.SQKFPKAEFVEVTKL.V
3354	219 - 233	584.3295	1749.9666	1749.9665	0.043	1	42	6.6e-005	1	U	L.SQKFPKAEFVEVTKL.V
3355	219 - 233	438.4990	1749.9667	1749.9665	0.13	1	24	0.0039	1	U	L.SQKFPKAEFVEVTKL.V
3356	219 - 233	438.4990	1749.9667	1749.9665	0.13	1	25	0.003	1	U	L.SQKFPKAEFVEVTKL.V
3681	219 - 233	638.3466	1912.0179	1912.0193	-0.77	1	40	0.0001	1	U	L.SQKFPKAEFVEVTKL.V + Glycation (K)
3682	219 - 233	638.3469	1912.0188	1912.0193	-0.30	1	38	0.00016	1	U	L.SQKFPKAEFVEVTKL.V + Glycation (K)
3683	219 - 233	479.0121	1912.0194	1912.0193	0.022	1	17	0.018	1	U	L.SQKFPKAEFVEVTKL.V + Glycation (K)
3684	219 - 233	479.0122	1912.0195	1912.0193	0.085	1	14	0.043	1	U	L.SQKFPKAEFVEVTKL.V + Glycation (K)
3685	219 - 233	638.3472	1912.0197	1912.0193	0.18	1	42	5.9e-005	1	U	L.SQKFPKAEFVEVTKL.V + Glycation (K)
3686	219 - 233	479.0123	1912.0200	1912.0193	0.34	1	17	0.021	1	U	L.SQKFPKAEFVEVTKL.V + Glycation (K)
3687	219 - 233	638.3477	1912.0213	1912.0193	1.05	1	51	7.9e-006	1	U	L.SQKFPKAEFVEVTKL.V + Glycation (K)
219	228 - 233	344.7153	687.4161	687.4167	-0.84	0	33	0.00048	1	U	F.VEVTKL.V
220	228 - 233	344.7154	687.4162	687.4167	-0.67	0	16	0.022	1	U	F.VEVTKL.V
221	228 - 233	344.7154	687.4162	687.4167	-0.67	0	15	0.033	1	U	F.VEVTKL.V
222	228 - 233	344.7154	687.4163	687.4167	-0.58	0	16	0.025	1	U	F.VEVTKL.V
223	228 - 233	344.7154	687.4163	687.4167	-0.58	0	15	0.032	1	U	F.VEVTKL.V
224	228 - 233	344.7154	687.4163	687.4167	-0.49	0	33	0.00047	1	U	F.VEVTKL.V
225	228 - 233	344.7154	687.4163	687.4167	-0.49	0	25	0.0035	1	U	F.VEVTKL.V
226	228 - 233	344.7154	687.4163	687.4167	-0.49	0	19	0.011	1	U	F.VEVTKL.V
227	228 - 233	344.7154	687.4163	687.4167	-0.49	0	19	0.011	1	U	F.VEVTKL.V
228	228 - 233	344.7155	687.4164	687.4167	-0.41	0	19	0.013	1	U	F.VEVTKL.V
229	228 - 233	344.7155	687.4164	687.4167	-0.41	0	15	0.035	1	U	F.VEVTKL.V
230	228 - 233	344.7155	687.4164	687.4167	-0.41	0	18	0.015	1	U	F.VEVTKL.V
231	228 - 233	344.7155	687.4164	687.4167	-0.41	0	18	0.017	1	U	F.VEVTKL.V
232	228 - 233	344.7155	687.4164	687.4167	-0.41	0	16	0.027	1	U	F.VEVTKL.V
233	228 - 233	344.7155	687.4164	687.4167	-0.41	0	19	0.011	1	U	F.VEVTKL.V

Query	Start - End	Observed	Mr(expt)	Mr(calc)	ppm	M	Score	Expect	Rank	U	Peptide
235	228 - 233	344.7155	687.4165	687.4167	-0.29	0	16	0.023	1	U	F.VEVTKL.V
236	228 - 233	344.7155	687.4165	687.4167	-0.29	0	18	0.014	1	U	F.VEVTKL.V
237	228 - 233	344.7155	687.4165	687.4167	-0.29	0	24	0.0037	1	U	F.VEVTKL.V
238	228 - 233	344.7155	687.4165	687.4167	-0.29	0	19	0.014	1	U	F.VEVTKL.V
239	228 - 233	344.7156	687.4165	687.4167	-0.20	0	19	0.013	1	U	F.VEVTKL.V
240	228 - 233	344.7156	687.4165	687.4167	-0.20	0	19	0.011	1	U	F.VEVTKL.V
242	228 - 233	344.7156	687.4166	687.4167	-0.11	0	19	0.014	1	U	F.VEVTKL.V
243	228 - 233	344.7156	687.4166	687.4167	-0.11	0	17	0.02	1	U	F.VEVTKL.V
244	228 - 233	344.7156	687.4166	687.4167	-0.11	0	19	0.013	1	U	F.VEVTKL.V
245	228 - 233	344.7156	687.4166	687.4167	-0.11	0	19	0.013	1	U	F.VEVTKL.V
246	228 - 233	344.7156	687.4167	687.4167	-0.028	0	19	0.011	1	U	F.VEVTKL.V
247	228 - 233	344.7156	687.4167	687.4167	-0.028	0	18	0.015	1	U	F.VEVTKL.V
248	228 - 233	344.7156	687.4167	687.4167	-0.028	0	16	0.023	1	U	F.VEVTKL.V
249	228 - 233	344.7156	687.4167	687.4167	0.060	0	19	0.012	1	U	F.VEVTKL.V
250	228 - 233	344.7157	687.4168	687.4167	0.15	0	17	0.019	1	U	F.VEVTKL.V
610	228 - 233	425.7418	849.4690	849.4695	-0.64	0	26	0.0023	1	U	F.VEVTKL.V + Glycation (K)
611	228 - 233	425.7418	849.4691	849.4695	-0.50	0	26	0.0026	1	U	F.VEVTKL.V + Glycation (K)
612	228 - 233	425.7418	849.4691	849.4695	-0.50	0	23	0.0046	1	U	F.VEVTKL.V + Glycation (K)
613	228 - 233	425.7419	849.4692	849.4695	-0.36	0	26	0.0023	1	U	F.VEVTKL.V + Glycation (K)
614	228 - 233	425.7419	849.4693	849.4695	-0.29	0	26	0.0023	1	U	F.VEVTKL.V + Glycation (K)
615	228 - 233	425.7419	849.4693	849.4695	-0.22	0	23	0.0051	1	U	F.VEVTKL.V + Glycation (K)
616	228 - 233	425.7419	849.4693	849.4695	-0.22	0	26	0.0024	1	U	F.VEVTKL.V + Glycation (K)
617	228 - 233	425.7419	849.4693	849.4695	-0.22	0	26	0.0023	1	U	F.VEVTKL.V + Glycation (K)
618	228 - 233	425.7421	849.4696	849.4695	0.088	0	26	0.0025	1	U	F.VEVTKL.V + Glycation (K)
619	228 - 233	425.7421	849.4696	849.4695	0.16	0	23	0.0048	1	U	F.VEVTKL.V + Glycation (K)
1432	228 - 237	558.8291	1115.6436	1115.6438	-0.14	1	26	0.0027	1	U	F.VEVTKLVTDL.T
1433	228 - 237	558.8298	1115.6450	1115.6438	1.07	1	25	0.003	1	U	F.VEVTKLVTDL.T
3878	234 - 250	1012.9955	2023.9764	2023.9819	-2.74	2	36	0.00026	1	U	L.VTDLTKVHKECCHGDLL.E
3879	234 - 250	1012.9966	2023.9786	2023.9819	-1.65	2	37	0.00018	1	U	L.VTDLTKVHKECCHGDLL.E
3880	234 - 250	675.6671	2023.9793	2023.9819	-1.30	2	34	0.00036	1	U	L.VTDLTKVHKECCHGDLL.E
3881	234 - 250	675.6672	2023.9797	2023.9819	-1.11	2	22	0.0057	1	U	L.VTDLTKVHKECCHGDLL.E
3886	234 - 250	675.6675	2023.9806	2023.9819	-0.66	2	13	0.049	1	U	L.VTDLTKVHKECCHGDLL.E
3893	234 - 250	1012.9979	2023.9813	2023.9819	-0.33	2	36	0.00026	1	U	L.VTDLTKVHKECCHGDLL.E
3894	234 - 250	675.6677	2023.9813	2023.9819	-0.30	2	28	0.0015	1	U	L.VTDLTKVHKECCHGDLL.E
3896	234 - 250	675.6681	2023.9826	2023.9819	0.33	2	35	0.00035	1	U	L.VTDLTKVHKECCHGDLL.E
3898	234 - 250	675.6683	2023.9830	2023.9819	0.51	2	20	0.011	1	U	L.VTDLTKVHKECCHGDLL.E
5134	234 - 259	768.3550	3069.3911	3069.3954	-1.42	3	69	1.3e-007	1	U	L.VTDLTKVHKECCHGDLLECADDRADL.A
5135	234 - 259	1024.1377	3069.3913	3069.3954	-1.35	3	72	5.8e-008	1	U	L.VTDLTKVHKECCHGDLLECADDRADL.A
5136	234 - 259	768.3554	3069.3923	3069.3954	-1.02	3	69	1.3e-007	1	U	L.VTDLTKVHKECCHGDLLECADDRADL.A
5137	234 - 259	1024.1381	3069.3924	3069.3954	-1.00	3	64	3.8e-007	1	U	L.VTDLTKVHKECCHGDLLECADDRADL.A
5139	234 - 259	768.3555	3069.3928	3069.3954	-0.86	3	71	7.6e-008	1	U	L.VTDLTKVHKECCHGDLLECADDRADL.A
5140	234 - 259	768.3555	3069.3930	3069.3954	-0.78	3	62	7e-007	1	U	L.VTDLTKVHKECCHGDLLECADDRADL.A
5141	234 - 259	768.3556	3069.3933	3069.3954	-0.70	3	72	6.2e-008	1	U	L.VTDLTKVHKECCHGDLLECADDRADL.A
5142	234 - 259	768.3558	3069.3940	3069.3954	-0.47	3	80	9.6e-009	1	U	L.VTDLTKVHKECCHGDLLECADDRADL.A
5143	234 - 259	1024.1387	3069.3942	3069.3954	-0.40	3	98	1.5e-010	1	U	L.VTDLTKVHKECCHGDLLECADDRADL.A
5144	234 - 259	768.3559	3069.3945	3069.3954	-0.30	3	98	1.5e-010	1	U	L.VTDLTKVHKECCHGDLLECADDRADL.A
5145	234 - 259	1024.1390	3069.3953	3069.3954	-0.040	3	94	4.2e-010	1	U	L.VTDLTKVHKECCHGDLLECADDRADL.A
5147	234 - 259	1024.1393	3069.3960	3069.3954	0.19	3	105	3.1e-011	1	U	L.VTDLTKVHKECCHGDLLECADDRADL.A
5148	234 - 259	768.3563	3069.3962	3069.3954	0.25	3	47	2.1e-005	1	U	L.VTDLTKVHKECCHGDLLECADDRADL.A
5150	234 - 259	1024.1394	3069.3964	3069.3954	0.31	3	82	6e-009	1	U	L.VTDLTKVHKECCHGDLLECADDRADL.A
5151	234 - 259	768.3564	3069.3964	3069.3954	0.33	3	98	1.8e-010	1	U	L.VTDLTKVHKECCHGDLLECADDRADL.A
5153	234 - 259	768.3565	3069.3967	3069.3954	0.42	3	62	7e-007	1	U	L.VTDLTKVHKECCHGDLLECADDRADL.A
5156	234 - 259	768.3573	3069.4001	3069.3954	1.52	3	53	5e-006	1	U	L.VTDLTKVHKECCHGDLLECADDRADL.A
5157	234 - 259	768.3577	3069.4018	3069.3954	2.09	3	69	1.2e-007	1	U	L.VTDLTKVHKECCHGDLLECADDRADL.A
5245	234 - 259	1078.1560	3231.4462	3231.4482	-0.63	3	84	3.7e-009	1	U	L.VTDLTKVHKECCHGDLLECADDRADL.A + Glyca
5246	234 - 259	808.8688	3231.4463	3231.4482	-0.61	3	61	8.1e-007	1	U	L.VTDLTKVHKECCHGDLLECADDRADL.A + Glyca
5247	234 - 259	808.8689	3231.4465	3231.4482	-0.54	3	96	2.7e-010	1	U	L.VTDLTKVHKECCHGDLLECADDRADL.A + Glyca
5248	234 - 259	808.8694	3231.4484	3231.4482	0.054	3	79	1.2e-008	1	U	L.VTDLTKVHKECCHGDLLECADDRADL.A + Glyca
5249	234 - 259	647.2972	3231.4495	3231.4482	0.40	3	35	0.0003	1	U	L.VTDLTKVHKECCHGDLLECADDRADL.A + Glyca
5250	234 - 259	1078.1577	3231.4513	3231.4482	0.95	3	70	1e-007	1	U	L.VTDLTKVHKECCHGDLLECADDRADL.A + Glyca
5313	234 - 262	1144.8686	3431.5841	3431.5908	-1.95	4	56	2.5e-006	1	U	L.VTDLTKVHKECCHGDLLECADDRADLAKY.I
5314	234 - 262	858.9036	3431.5851	3431.5908	-1.65	4	27	0.0018	1	U	L.VTDLTKVHKECCHGDLLECADDRADLAKY.I
5315	234 - 262	858.9036	3431.5851	3431.5908	-1.65	4	29	0.0012	1	U	L.VTDLTKVHKECCHGDLLECADDRADLAKY.I
5317	234 - 262	858.9043	3431.5881	3431.5908	-0.79	4	27	0.0018	1	U	L.VTDLTKVHKECCHGDLLECADDRADLAKY.I
5318	234 - 262	858.9044	3431.5886	3431.5908	-0.65	4	16	0.025	1	U	L.VTDLTKVHKECCHGDLLECADDRADLAKY.I
5319	234 - 262	858.9044	3431.5886	3431.5908	-0.65	4	30	0.0011	1	U	L.VTDLTKVHKECCHGDLLECADDRADLAKY.I
5320	234 - 262	858.9046	3431.5893	3431.5908	-0.44	4	35	0.00029	1	U	L.VTDLTKVHKECCHGDLLECADDRADLAKY.I
5321	234 - 262	858.9046	3431.5893	3431.5908	-0.44	4	32	0.00057	1	U	L.VTDLTKVHKECCHGDLLECADDRADLAKY.I
5322	234 - 262	1144.8706	3431.5900	3431.5908	-0.23	4	59	1.3e-006	1	U	L.VTDLTKVHKECCHGDLLECADDRADLAKY.I
5323	234 - 262	858.9048	3431.5901	3431.5908	-0.22	4	30	0.001	1	U	L.VTDLTKVHKECCHGDLLECADDRADLAKY.I
5331	234 - 262	1144.8712	3431.5918	3431.5908	0.30	4	56	2.3e-006	1	U	L.VTDLTKVHKECCHGDLLECADDRADLAKY.I
5336	234 - 262	1144.8717	3431.5933	3431.5908	0.72	4	53	4.6e-006	1	U	L.VTDLTKVHKECCHGDLLECADDRADLAKY.I
5398	234 - 262	1198.8864	3593.6372	3593.6436	-1.78	4	50	9.5e-006	1	U	L.VTDLTKVHKECCHGDLLECADDRADLAKY.I + Gl
5399	234 - 262	899.4169	3593.6386	3593.6436	-1.40	4	26	0.0025	1	U	L.VTDLTKVHKECCHGDLLECADDRADLAKY.I + Gl
5400	234 - 262	899.4174	3593.6403	3593.6436	-0.92	4	14	0.037	1	U	L.VTDLTKVHKECCHGDLLECADDRADLAKY.I + Gl
5401	234 - 262	899.4174	3593.6405	3593.6436	-0.86	4	16	0.026	1	U	L.VTDLTKVHKECCHGDLLECADDRADLAKY.I + Gl
5408	234 - 262	899.4180	3593.6430	3593.6436	-0.17	4	13	0.05	1	U	L.VTDLTKVHKECCHGDLLECADDRADLAKY.I + Gl
5415	234 - 262	1198.8917	3593.6533	3593.6436	2.70	4	49	1.3e-005	1	U	L.VTDLTKVHKECCHGDLLECADDRADLAKY.I + Gl
4743	238 - 259	661.2981	2641.1633	2641.1683	-1.90	2	34	0.00042	1	U	L.TKVHKECCHGDLLECADDRADL.A
4744	238 - 259	661.2986	2641.1652	2641.1683	-1.17	2	30	0.0009	1	U	L.TKVHKECCHGDLLECADDRADL.A
1217	251 - 259	532.7191	1063.4237	1063.4240	-0.32	0	23	0.0051	1	U	L.ECADDRADL.A
1218	251 - 259	532.7192	1063.4239	1063.4240	-0.092	0	24	0.004	1	U	L.ECADDRADL.A
1219	251 - 259	532.7192	1063.4239	1063.4240	-0.092	0	26	0.0028	1	U	L.ECADDRADL.A
1220	251 - 259	532.7193	1063.4240	1063.4240	0.021	0	23	0.0054	1	U	L.ECADDRADL.A
1221	251 - 259	532.7193	1063.4240	1063.4240	0.021	0	24	0.0039	1	U	L.ECADDRADL.A
1222	251 - 259	532.7193	1063.4240	1063.4240	0.021	0	26	0.0028	1	U	L.ECADDRADL.A
1223	251 - 259	532.7193	1063.4240	1063.4240	0.021	0	24	0.0039	1	U	L.ECADDRADL.A
1224	251 - 259	532.7194	1063.4242	1063.4240	0.13	0	23	0.0054	1	U	L.ECADDRADL.A
1225	251 - 259	532.7194	1063.4242	1063.4240	0.13	0	26	0.0028	1	U	L.ECADDRADL.A

Query	Start – End	Observed	Mr(expt)	Mr(calc)	ppm	M	Score	Expect	Rank	U	Peptide
1226	251 – 259	532.7194	1063.4243	1063.4240	0.25	0	24	0.004	1	U	L.ECADDRADL.A
1227	251 – 259	532.7195	1063.4244	1063.4240	0.36	0	23	0.0055	1	U	L.ECADDRADL.A
1228	251 – 259	532.7195	1063.4244	1063.4240	0.36	0	23	0.0053	1	U	L.ECADDRADL.A
2449	251 – 262	713.8157	1425.6168	1425.6194	-1.84	1	18	0.015	1	U	L.ECADDRADLAKY.I
2450	251 – 262	713.8162	1425.6179	1425.6194	-1.07	1	30	0.0011	1	U	L.ECADDRADLAKY.I
2451	251 – 262	713.8164	1425.6183	1425.6194	-0.80	1	31	0.00089	1	U	L.ECADDRADLAKY.I
2452	251 – 262	713.8165	1425.6184	1425.6194	-0.72	1	22	0.0064	1	U	L.ECADDRADLAKY.I
2453	251 – 262	713.8165	1425.6185	1425.6194	-0.64	1	30	0.00097	1	U	L.ECADDRADLAKY.I
2454	251 – 262	713.8166	1425.6186	1425.6194	-0.55	1	30	0.00096	1	U	L.ECADDRADLAKY.I
2459	251 – 262	713.8168	1425.6191	1425.6194	-0.22	1	28	0.0018	1	U	L.ECADDRADLAKY.I
2292	263 – 274	697.3339	1392.6533	1392.6555	-1.58	0	59	1.2e-006	1	U	Y.ICDNQDTISSKL.K
2293	263 – 274	697.3341	1392.6535	1392.6555	-1.39	0	66	2.3e-007	1	U	Y.ICDNQDTISSKL.K
2294	263 – 274	697.3342	1392.6538	1392.6555	-1.22	0	57	1.8e-006	1	U	Y.ICDNQDTISSKL.K
2295	263 – 274	697.3343	1392.6540	1392.6555	-1.05	0	60	1.1e-006	1	U	Y.ICDNQDTISSKL.K
2296	263 – 274	697.3344	1392.6543	1392.6555	-0.88	0	58	1.5e-006	1	U	Y.ICDNQDTISSKL.K
2297	263 – 274	697.3344	1392.6543	1392.6555	-0.88	0	61	8.7e-007	1	U	Y.ICDNQDTISSKL.K
2298	263 – 274	697.3344	1392.6543	1392.6555	-0.88	0	67	1.8e-007	1	U	Y.ICDNQDTISSKL.K
2299	263 – 274	697.3345	1392.6544	1392.6555	-0.79	0	63	4.8e-007	1	U	Y.ICDNQDTISSKL.K
2300	263 – 274	697.3345	1392.6545	1392.6555	-0.70	0	59	1.3e-006	1	U	Y.ICDNQDTISSKL.K
2301	263 – 274	697.3345	1392.6545	1392.6555	-0.70	0	66	2.5e-007	1	U	Y.ICDNQDTISSKL.K
2302	263 – 274	697.3345	1392.6545	1392.6555	-0.70	0	67	1.8e-007	1	U	Y.ICDNQDTISSKL.K
2303	263 – 274	697.3346	1392.6546	1392.6555	-0.62	0	57	2.1e-006	1	U	Y.ICDNQDTISSKL.K
2304	263 – 274	697.3346	1392.6546	1392.6555	-0.62	0	67	1.8e-007	1	U	Y.ICDNQDTISSKL.K
2305	263 – 274	697.3347	1392.6548	1392.6555	-0.52	0	58	1.4e-006	1	U	Y.ICDNQDTISSKL.K
2306	263 – 274	697.3347	1392.6548	1392.6555	-0.52	0	63	4.9e-007	1	U	Y.ICDNQDTISSKL.K
2307	263 – 274	697.3348	1392.6550	1392.6555	-0.34	0	67	1.8e-007	1	U	Y.ICDNQDTISSKL.K
2308	263 – 274	697.3348	1392.6551	1392.6555	-0.26	0	69	1.2e-007	1	U	Y.ICDNQDTISSKL.K
2309	263 – 274	697.3348	1392.6551	1392.6555	-0.26	0	60	1e-006	1	U	Y.ICDNQDTISSKL.K
2310	263 – 274	697.3348	1392.6551	1392.6555	-0.26	0	67	1.9e-007	1	U	Y.ICDNQDTISSKL.K
2311	263 – 274	697.3348	1392.6551	1392.6555	-0.26	0	67	1.9e-007	1	U	Y.ICDNQDTISSKL.K
2312	263 – 274	697.3349	1392.6552	1392.6555	-0.17	0	59	1.1e-006	1	U	Y.ICDNQDTISSKL.K
2313	263 – 274	697.3350	1392.6555	1392.6555	0.00072	0	67	1.8e-007	1	U	Y.ICDNQDTISSKL.K
2314	263 – 274	697.3351	1392.6557	1392.6555	0.17	0	42	6.4e-005	1	U	Y.ICDNQDTISSKL.K
2315	263 – 274	697.3355	1392.6563	1392.6555	0.62	0	67	1.9e-007	1	U	Y.ICDNQDTISSKL.K
2316	263 – 274	697.3357	1392.6569	1392.6555	1.05	0	77	1.8e-008	1	U	Y.ICDNQDTISSKL.K
2877	263 – 274	778.3597	1554.7048	1554.7083	-2.25	0	45	3.4e-005	1	U	Y.ICDNQDTISSKL.K + Glycation (K)
2878	263 – 274	778.3619	1554.7092	1554.7083	0.58	0	33	0.00051	1	U	Y.ICDNQDTISSKL.K + Glycation (K)
5510	275 – 318	1038.6912	5188.4194	5188.4501	-5.91	4	16	0.027	1	U	L.KECCDKPLLEKSHCIAEVEKDAIPENLPPLTADFA
5511	275 – 318	1298.1166	5188.4372	5188.4501	-2.48	4	46	2.3e-005	1	U	L.KECCDKPLLEKSHCIAEVEKDAIPENLPPLTADFA
5512	275 – 318	1038.6957	5188.4420	5188.4501	-1.56	4	15	0.029	1	U	L.KECCDKPLLEKSHCIAEVEKDAIPENLPPLTADFA
5513	275 – 318	865.7479	5188.4439	5188.4501	-1.20	4	20	0.009	1	U	L.KECCDKPLLEKSHCIAEVEKDAIPENLPPLTADFA
5514	275 – 318	1038.6962	5188.4445	5188.4501	-1.08	4	18	0.017	1	U	L.KECCDKPLLEKSHCIAEVEKDAIPENLPPLTADFA
5515	275 – 318	865.7481	5188.4446	5188.4501	-1.05	4	30	0.0011	1	U	L.KECCDKPLLEKSHCIAEVEKDAIPENLPPLTADFA
5516	275 – 318	865.7482	5188.4454	5188.4501	-0.91	4	19	0.013	1	U	L.KECCDKPLLEKSHCIAEVEKDAIPENLPPLTADFA
5517	275 – 318	865.7482	5188.4454	5188.4501	-0.91	4	22	0.0065	1	U	L.KECCDKPLLEKSHCIAEVEKDAIPENLPPLTADFA
5519	275 – 318	1038.6964	5188.4457	5188.4501	-0.85	4	18	0.015	1	U	L.KECCDKPLLEKSHCIAEVEKDAIPENLPPLTADFA
5520	275 – 318	1038.6965	5188.4463	5188.4501	-0.74	4	19	0.013	1	U	L.KECCDKPLLEKSHCIAEVEKDAIPENLPPLTADFA
5521	275 – 318	1038.6965	5188.4463	5188.4501	-0.74	4	19	0.013	1	U	L.KECCDKPLLEKSHCIAEVEKDAIPENLPPLTADFA
5524	275 – 318	865.7485	5188.4472	5188.4501	-0.56	4	31	0.0008	1	U	L.KECCDKPLLEKSHCIAEVEKDAIPENLPPLTADFA
5525	275 – 318	865.7485	5188.4472	5188.4501	-0.56	4	33	0.0005	1	U	L.KECCDKPLLEKSHCIAEVEKDAIPENLPPLTADFA
5526	275 – 318	742.2141	5188.4474	5188.4501	-0.51	4	15	0.031	1	U	L.KECCDKPLLEKSHCIAEVEKDAIPENLPPLTADFA
5528	275 – 318	1298.1195	5188.4489	5188.4501	-0.22	4	50	1e-005	1	U	L.KECCDKPLLEKSHCIAEVEKDAIPENLPPLTADFA
5529	275 – 318	1038.6971	5188.4493	5188.4501	-0.15	4	17	0.018	1	U	L.KECCDKPLLEKSHCIAEVEKDAIPENLPPLTADFA
5530	275 – 318	1298.1196	5188.4494	5188.4501	-0.13	4	57	2.1e-006	1	U	L.KECCDKPLLEKSHCIAEVEKDAIPENLPPLTADFA
5532	275 – 318	865.7490	5188.4505	5188.4501	0.072	4	27	0.002	1	U	L.KECCDKPLLEKSHCIAEVEKDAIPENLPPLTADFA
5535	275 – 318	1298.1202	5188.4519	5188.4501	0.34	4	39	0.00012	1	U	L.KECCDKPLLEKSHCIAEVEKDAIPENLPPLTADFA
5536	275 – 318	865.7494	5188.4527	5188.4501	0.50	4	19	0.014	1	U	L.KECCDKPLLEKSHCIAEVEKDAIPENLPPLTADFA
5537	275 – 318	865.7494	5188.4527	5188.4501	0.50	4	21	0.0079	1	U	L.KECCDKPLLEKSHCIAEVEKDAIPENLPPLTADFA
5538	275 – 318	1298.1209	5188.4547	5188.4501	0.88	4	42	6.5e-005	1	U	L.KECCDKPLLEKSHCIAEVEKDAIPENLPPLTADFA
5539	275 – 318	1038.6982	5188.4548	5188.4501	0.91	4	15	0.03	1	U	L.KECCDKPLLEKSHCIAEVEKDAIPENLPPLTADFA
5540	275 – 318	1298.1216	5188.4572	5188.4501	1.37	4	42	6e-005	1	U	L.KECCDKPLLEKSHCIAEVEKDAIPENLPPLTADFA
5541	275 – 318	865.7504	5188.4586	5188.4501	1.63	4	14	0.044	1	U	L.KECCDKPLLEKSHCIAEVEKDAIPENLPPLTADFA
5542	275 – 318	1038.7014	5188.4707	5188.4501	3.98	4	24	0.0044	1	U	L.KECCDKPLLEKSHCIAEVEKDAIPENLPPLTADFA
5587	275 – 318	892.7571	5350.4988	5350.5029	-0.76	4	15	0.03	1	U	L.KECCDKPLLEKSHCIAEVEKDAIPENLPPLTADFA
5588	275 – 318	892.7573	5350.4999	5350.5029	-0.56	4	14	0.041	1	U	L.KECCDKPLLEKSHCIAEVEKDAIPENLPPLTADFA
5592	275 – 318	892.7579	5350.5039	5350.5029	0.19	4	14	0.04	1	U	L.KECCDKPLLEKSHCIAEVEKDAIPENLPPLTADFA
5593	275 – 318	1071.1084	5350.5056	5350.5029	0.51	4	14	0.04	1	U	L.KECCDKPLLEKSHCIAEVEKDAIPENLPPLTADFA
5594	275 – 318	1071.1086	5350.5068	5350.5029	0.73	4	29	0.0013	1	U	L.KECCDKPLLEKSHCIAEVEKDAIPENLPPLTADFA
5500	283 – 318	832.6037	4157.9821	4157.9925	-2.50	3	37	0.0002	1	U	L.LEKSHCIAEVEKDAIPENLPPLTADFAEDKDVCKN
5501	283 – 318	832.6050	4157.9885	4157.9925	-0.96	3	46	2.5e-005	1	U	L.LEKSHCIAEVEKDAIPENLPPLTADFAEDKDVCKN
5502	283 – 318	832.6054	4157.9904	4157.9925	-0.51	3	46	2.4e-005	1	U	L.LEKSHCIAEVEKDAIPENLPPLTADFAEDKDVCKN
1772	309 – 318	621.2759	1240.5373	1240.5394	-1.66	0	27	0.0019	1	U	F.AEDKDVCKNY.Q
1773	309 – 318	621.2766	1240.5385	1240.5394	-0.67	0	28	0.0015	1	U	F.AEDKDVCKNY.Q
1775	309 – 318	621.2770	1240.5395	1240.5394	0.12	0	20	0.01	1	U	F.AEDKDVCKNY.Q
492	319 – 325	404.6952	807.3758	807.3763	-0.62	0	18	0.017	1	U	Y.QEAKDAF.L
494	319 – 325	404.6953	807.3760	807.3763	-0.32	0	16	0.026	1	U	Y.QEAKDAF.L
496	319 – 325	404.6953	807.3761	807.3763	-0.25	0	15	0.03	1	U	Y.QEAKDAF.L
497	319 – 325	404.6954	807.3762	807.3763	-0.022	0	14	0.039	1	U	Y.QEAKDAF.L
1681	319 – 329	606.7982	1211.5818	1211.5822	-0.39	2	21	0.0081	1	U	Y.QEAKDAFLGSF.L
1684	319 – 329	606.7983	1211.5821	1211.5822	-0.097	2	26	0.0028	1	U	Y.QEAKDAFLGSF.L
1686	319 – 329	606.7984	1211.5822	1211.5822	0.0025	2	24	0.004	1	U	Y.QEAKDAFLGSF.L
1687	319 – 329	606.7985	1211.5824	1211.5822	0.10	2	25	0.0033	1	U	Y.QEAKDAFLGSF.L
1688	319 – 329	606.7985	1211.5824	1211.5822	0.10	2	22	0.0068	1	U	Y.QEAKDAFLGSF.L
1689	319 – 329	606.7985	1211.5824	1211.5822	0.10	2	19	0.014	1	U	Y.QEAKDAFLGSF.L
1690	319 – 329	606.7985	1211.5824	1211.5822	0.10	2	24	0.004	1	U	Y.QEAKDAFLGSF.L
1691	319 – 329	606.7985	1211.5824	1211.5822	0.10	2	22	0.0061	1	U	Y.QEAKDAFLGSF.L
1692	319 – 329	606.7985	1211.5825	1211.5822	0.20	2	22	0.0061	1	U	Y.QEAKDAFLGSF.L
1694	319 – 329	606.7985	1211.5825	1211.5822	0.20	2	22	0.0065	1	U	Y.QEAKDAFLGSF.L
1695	319 – 329	606.7986	1211.5827	1211.5822	0.42	2	17	0.019	1	U	Y.QEAKDAFLGSF.L
1696	319 – 329	606.7988	1211.5830	1211.5822	0.61	2	22	0.0063	1	U	Y.QEAKDAFLGSF.L

Query	Start – End	Observed	Mr(expt)	Mr(calc)	ppm	M	Score	Expect	Rank	U	Peptide
2069	319 – 330	663.3400	1324.6655	1324.6663	-0.60	3	20	0.01	1	U	Y.QEAKDAFLGSFL.Y
2070	319 – 330	663.3404	1324.6662	1324.6663	-0.059	3	20	0.01	1	U	Y.QEAKDAFLGSFL.Y
2609	319 – 331	744.8715	1487.7284	1487.7296	-0.85	4	23	0.0051	1	U	Y.QEAKDAFLGSFLY.E
2610	319 – 331	744.8715	1487.7285	1487.7296	-0.77	4	28	0.0014	1	U	Y.QEAKDAFLGSFLY.E
2611	319 – 331	744.8716	1487.7286	1487.7296	-0.69	4	28	0.0017	1	U	Y.QEAKDAFLGSFLY.E
2613	319 – 331	744.8720	1487.7295	1487.7296	-0.11	4	36	0.00027	1	U	Y.QEAKDAFLGSFLY.E
748	346 – 352	298.1797	891.5172	891.5178	-0.58	2	16	0.027	1	U	L.LRLAKEY.E
749	346 – 352	446.7661	891.5175	891.5178	-0.24	2	27	0.0021	1	U	L.LRLAKEY.E
750	346 – 352	298.1798	891.5176	891.5178	-0.18	2	19	0.014	1	U	L.LRLAKEY.E
751	346 – 352	446.7661	891.5176	891.5178	-0.17	2	26	0.0023	1	U	L.LRLAKEY.E
752	346 – 352	446.7661	891.5177	891.5178	-0.10	2	25	0.0035	1	U	L.LRLAKEY.E
753	346 – 352	446.7661	891.5177	891.5178	-0.10	2	27	0.0022	1	U	L.LRLAKEY.E
754	346 – 352	298.1798	891.5177	891.5178	-0.077	2	18	0.016	1	U	L.LRLAKEY.E
755	346 – 352	298.1798	891.5177	891.5178	-0.077	2	21	0.0089	1	U	L.LRLAKEY.E
756	346 – 352	298.1799	891.5179	891.5178	0.12	2	23	0.0054	1	U	L.LRLAKEY.E
4915	347 – 369	708.0598	2828.2099	2828.2204	-3.70	3	43	5.6e-005	1	U	L.RLAKEYEATLEECCAKDDPHACY.S
4916	347 – 369	708.0605	2828.2129	2828.2204	-2.65	3	22	0.006	1	U	L.RLAKEYEATLEECCAKDDPHACY.S
4918	347 – 369	943.7457	2828.2154	2828.2204	-1.76	3	52	6.6e-006	1	U	L.RLAKEYEATLEECCAKDDPHACY.S
4919	347 – 369	943.7460	2828.2161	2828.2204	-1.51	3	49	1.2e-005	1	U	L.RLAKEYEATLEECCAKDDPHACY.S
4920	347 – 369	708.0614	2828.2165	2828.2204	-1.36	3	14	0.041	1	U	L.RLAKEYEATLEECCAKDDPHACY.S
4921	347 – 369	708.0615	2828.2167	2828.2204	-1.28	3	41	7.8e-005	1	U	L.RLAKEYEATLEECCAKDDPHACY.S
4922	347 – 369	708.0616	2828.2175	2828.2204	-1.01	3	40	0.0001	1	U	L.RLAKEYEATLEECCAKDDPHACY.S
4923	347 – 369	566.6509	2828.2180	2828.2204	-0.82	3	19	0.014	1	U	L.RLAKEYEATLEECCAKDDPHACY.S
4924	347 – 369	708.0618	2828.2182	2828.2204	-0.76	3	41	7.5e-005	1	U	L.RLAKEYEATLEECCAKDDPHACY.S
4925	347 – 369	943.7467	2828.2183	2828.2204	-0.74	3	51	8.3e-006	1	U	L.RLAKEYEATLEECCAKDDPHACY.S
4926	347 – 369	943.7467	2828.2183	2828.2204	-0.74	3	56	2.7e-006	1	U	L.RLAKEYEATLEECCAKDDPHACY.S
5081	347 – 369	997.7622	2990.2646	2990.2732	-2.86	3	33	0.00052	1	U	L.RLAKEYEATLEECCAKDDPHACY.S + Glycation
5083	347 – 369	997.7636	2990.2688	2990.2732	-1.46	3	31	0.00088	1	U	L.RLAKEYEATLEECCAKDDPHACY.S + Glycation
5084	347 – 369	748.5748	2990.2700	2990.2732	-1.07	3	42	5.8e-005	1	U	L.RLAKEYEATLEECCAKDDPHACY.S + Glycatio
5085	347 – 369	748.5751	2990.2712	2990.2732	-0.67	3	43	5.2e-005	1	U	L.RLAKEYEATLEECCAKDDPHACY.S + Glycatio
5086	347 – 369	748.5753	2990.2722	2990.2732	-0.34	3	40	0.00011	1	U	L.RLAKEYEATLEECCAKDDPHACY.S + Glycatio
4711	349 – 369	854.0183	2559.0331	2559.0352	-0.81	2	48	1.7e-005	1	U	L.AKEYEATLEECCAKDDPHACY.S
4712	349 – 369	854.0184	2559.0333	2559.0352	-0.74	2	54	3.6e-006	1	U	L.AKEYEATLEECCAKDDPHACY.S
4714	349 – 369	854.0185	2559.0336	2559.0352	-0.60	2	50	1e-005	1	U	L.AKEYEATLEECCAKDDPHACY.S
4715	349 – 369	640.7657	2559.0337	2559.0352	-0.60	2	23	0.0048	1	U	L.AKEYEATLEECCAKDDPHACY.S
4716	349 – 369	854.0186	2559.0338	2559.0352	-0.53	2	37	0.0002	1	U	L.AKEYEATLEECCAKDDPHACY.S
4717	349 – 369	640.7658	2559.0341	2559.0352	-0.41	2	25	0.0029	1	U	L.AKEYEATLEECCAKDDPHACY.S
4718	349 – 369	854.0187	2559.0342	2559.0352	-0.38	2	44	3.8e-005	1	U	L.AKEYEATLEECCAKDDPHACY.S
4719	349 – 369	640.7659	2559.0346	2559.0352	-0.22	2	25	0.0033	1	U	L.AKEYEATLEECCAKDDPHACY.S
4720	349 – 369	640.7659	2559.0346	2559.0352	-0.22	2	30	0.0011	1	U	L.AKEYEATLEECCAKDDPHACY.S
4721	349 – 369	640.7661	2559.0353	2559.0352	0.058	2	28	0.0017	1	U	L.AKEYEATLEECCAKDDPHACY.S
4722	349 – 369	640.7662	2559.0356	2559.0352	0.15	2	35	0.00034	1	U	L.AKEYEATLEECCAKDDPHACY.S
4723	349 – 369	640.7665	2559.0371	2559.0352	0.73	2	18	0.015	1	U	L.AKEYEATLEECCAKDDPHACY.S
3990	353 – 369	1034.9045	2067.7945	2067.7972	-1.30	1	62	6.3e-007	1	U	Y.EATLEECCAKDDPHACY.S
3991	353 – 369	1034.9047	2067.7948	2067.7972	-1.18	1	63	5.4e-007	1	U	Y.EATLEECCAKDDPHACY.S
3992	353 – 369	690.2722	2067.7948	2067.7972	-1.15	1	37	0.00018	1	U	Y.EATLEECCAKDDPHACY.S
3993	353 – 369	690.2723	2067.7950	2067.7972	-1.06	1	27	0.002	1	U	Y.EATLEECCAKDDPHACY.S
3994	353 – 369	1034.9049	2067.7953	2067.7972	-0.94	1	60	9.5e-007	1	U	Y.EATLEECCAKDDPHACY.S
3995	353 – 369	690.2724	2067.7954	2067.7972	-0.89	1	28	0.0017	1	U	Y.EATLEECCAKDDPHACY.S
3996	353 – 369	690.2725	2067.7956	2067.7972	-0.80	1	27	0.0021	1	U	Y.EATLEECCAKDDPHACY.S
3997	353 – 369	690.2725	2067.7957	2067.7972	-0.72	1	27	0.0021	1	U	Y.EATLEECCAKDDPHACY.S
3998	353 – 369	1034.9052	2067.7957	2067.7972	-0.71	1	65	3e-007	1	U	Y.EATLEECCAKDDPHACY.S
3999	353 – 369	690.2726	2067.7959	2067.7972	-0.63	1	22	0.0061	1	U	Y.EATLEECCAKDDPHACY.S
4000	353 – 369	1034.9053	2067.7960	2067.7972	-0.59	1	62	5.9e-007	1	U	Y.EATLEECCAKDDPHACY.S
4002	353 – 369	690.2726	2067.7961	2067.7972	-0.54	1	28	0.0016	1	U	Y.EATLEECCAKDDPHACY.S
4003	353 – 369	690.2726	2067.7961	2067.7972	-0.54	1	19	0.013	1	U	Y.EATLEECCAKDDPHACY.S
4004	353 – 369	690.2726	2067.7961	2067.7972	-0.54	1	26	0.0023	1	U	Y.EATLEECCAKDDPHACY.S
4005	353 – 369	1034.9054	2067.7962	2067.7972	-0.47	1	59	1.3e-006	1	U	Y.EATLEECCAKDDPHACY.S
4006	353 – 369	1034.9054	2067.7962	2067.7972	-0.47	1	66	2.4e-007	1	U	Y.EATLEECCAKDDPHACY.S
4007	353 – 369	1034.9054	2067.7962	2067.7972	-0.47	1	64	4e-007	1	U	Y.EATLEECCAKDDPHACY.S
4008	353 – 369	690.2727	2067.7963	2067.7972	-0.44	1	15	0.033	1	U	Y.EATLEECCAKDDPHACY.S
4009	353 – 369	690.2727	2067.7963	2067.7972	-0.44	1	26	0.0022	1	U	Y.EATLEECCAKDDPHACY.S
4010	353 – 369	690.2728	2067.7965	2067.7972	-0.35	1	13	0.045	1	U	Y.EATLEECCAKDDPHACY.S
4011	353 – 369	690.2728	2067.7965	2067.7972	-0.35	1	14	0.041	1	U	Y.EATLEECCAKDDPHACY.S
4012	353 – 369	690.2728	2067.7965	2067.7972	-0.35	1	26	0.0025	1	U	Y.EATLEECCAKDDPHACY.S
4013	353 – 369	690.2728	2067.7967	2067.7972	-0.27	1	26	0.0024	1	U	Y.EATLEECCAKDDPHACY.S
4014	353 – 369	1034.9056	2067.7967	2067.7972	-0.23	1	63	5.5e-007	1	U	Y.EATLEECCAKDDPHACY.S
4015	353 – 369	690.2729	2067.7968	2067.7972	-0.18	1	17	0.022	1	U	Y.EATLEECCAKDDPHACY.S
4019	353 – 369	690.2730	2067.7970	2067.7972	-0.091	1	14	0.04	1	U	Y.EATLEECCAKDDPHACY.S
4020	353 – 369	690.2730	2067.7970	2067.7972	-0.091	1	29	0.0012	1	U	Y.EATLEECCAKDDPHACY.S
4021	353 – 369	690.2730	2067.7972	2067.7972	-0.0044	1	15	0.03	1	U	Y.EATLEECCAKDDPHACY.S
4022	353 – 369	690.2730	2067.7972	2067.7972	-0.0044	1	25	0.0031	1	U	Y.EATLEECCAKDDPHACY.S
4023	353 – 369	690.2731	2067.7974	2067.7972	0.083	1	19	0.014	1	U	Y.EATLEECCAKDDPHACY.S
4024	353 – 369	690.2731	2067.7976	2067.7972	0.17	1	17	0.018	1	U	Y.EATLEECCAKDDPHACY.S
4025	353 – 369	1034.9061	2067.7977	2067.7972	0.24	1	51	7.8e-006	1	U	Y.EATLEECCAKDDPHACY.S
4026	353 – 369	690.2734	2067.7983	2067.7972	0.53	1	25	0.0029	1	U	Y.EATLEECCAKDDPHACY.S
4028	353 – 369	690.2740	2067.8003	2067.7972	1.50	1	25	0.0035	1	U	Y.EATLEECCAKDDPHACY.S
4029	353 – 369	690.2750	2067.8031	2067.7972	2.82	1	14	0.04	1	U	Y.EATLEECCAKDDPHACY.S
499	370 – 376	405.2233	808.4320	808.4331	-1.27	1	17	0.018	1	U	Y.STVFDKL.K
500	370 – 376	405.2235	808.4325	808.4331	-0.68	1	20	0.01	1	U	Y.STVFDKL.K
501	370 – 376	405.2237	808.4328	808.4331	-0.31	1	22	0.0062	1	U	Y.STVFDKL.K
502	370 – 376	405.2237	808.4328	808.4331	-0.31	1	37	0.00018	1	U	Y.STVFDKL.K
503	370 – 376	405.2237	808.4328	808.4331	-0.31	1	40	9.5e-005	1	U	Y.STVFDKL.K
504	370 – 376	405.2237	808.4329	808.4331	-0.23	1	26	0.0026	1	U	Y.STVFDKL.K
505	370 – 376	405.2237	808.4329	808.4331	-0.23	1	32	0.0007	1	U	Y.STVFDKL.K
506	370 – 376	405.2237	808.4329	808.4331	-0.23	1	34	0.00037	1	U	Y.STVFDKL.K
507	370 – 376	405.2237	808.4329	808.4331	-0.23	1	40	9.3e-005	1	U	Y.STVFDKL.K
508	370 – 376	405.2238	808.4329	808.4331	-0.16	1	34	0.00044	1	U	Y.STVFDKL.K
509	370 – 376	405.2238	808.4329	808.4331	-0.16	1	33	0.00045	1	U	Y.STVFDKL.K
510	370 – 376	405.2238	808.4329	808.4331	-0.16	1	34	0.00036	1	U	Y.STVFDKL.K

Query	Start - End	Observed	Mr(expt)	Mr(calc)	ppm	M	Score	Expect	Rank	U	Peptide
511	370 - 376	405.2238	808.4329	808.4331	-0.16	1	35	0.00035	1	U	Y.STVFDKL.K
512	370 - 376	405.2238	808.4330	808.4331	-0.058	1	35	0.00035	1	U	Y.STVFDKL.K
513	370 - 376	405.2238	808.4331	808.4331	0.016	1	34	0.00037	1	U	Y.STVFDKL.K
514	370 - 376	405.2238	808.4331	808.4331	0.016	1	34	0.00037	1	U	Y.STVFDKL.K
515	370 - 376	405.2238	808.4331	808.4331	0.016	1	35	0.00035	1	U	Y.STVFDKL.K
516	370 - 376	405.2239	808.4331	808.4331	0.090	1	37	0.00019	1	U	Y.STVFDKL.K
517	370 - 376	405.2239	808.4332	808.4331	0.16	1	35	0.00035	1	U	Y.STVFDKL.K
933	370 - 376	486.2501	970.4857	970.4859	-0.22	1	22	0.0064	1	U	Y.STVFDKL.K + Glycation (K)
934	370 - 376	486.2502	970.4859	970.4859	-0.034	1	19	0.014	1	U	Y.STVFDKL.K + Glycation (K)
935	370 - 376	486.2502	970.4859	970.4859	-0.034	1	22	0.0064	1	U	Y.STVFDKL.K + Glycation (K)
936	370 - 376	486.2503	970.4861	970.4859	0.17	1	22	0.0064	1	U	Y.STVFDKL.K + Glycation (K)
937	370 - 376	486.2504	970.4863	970.4859	0.42	1	23	0.0051	1	U	Y.STVFDKL.K + Glycation (K)
2829	374 - 386	774.9213	1547.8280	1547.8307	-1.79	2	46	2.6e-005	1	U	F.DKLKHLVDEPQNL.I
2830	374 - 386	774.9215	1547.8285	1547.8307	-1.46	2	46	2.6e-005	1	U	F.DKLKHLVDEPQNL.I
2831	374 - 386	774.9216	1547.8286	1547.8307	-1.39	2	44	4.1e-005	1	U	F.DKLKHLVDEPQNL.I
2832	374 - 386	774.9216	1547.8286	1547.8307	-1.39	2	41	7.8e-005	1	U	F.DKLKHLVDEPQNL.I
2833	374 - 386	774.9216	1547.8286	1547.8307	-1.39	2	54	4.4e-006	1	U	F.DKLKHLVDEPQNL.I
2834	374 - 386	774.9217	1547.8288	1547.8307	-1.23	2	58	1.5e-006	1	U	F.DKLKHLVDEPQNL.I
2835	374 - 386	774.9217	1547.8289	1547.8307	-1.15	2	24	0.0042	1	U	F.DKLKHLVDEPQNL.I
2836	374 - 386	774.9217	1547.8289	1547.8307	-1.15	2	63	5.3e-007	1	U	F.DKLKHLVDEPQNL.I
2837	374 - 386	774.9218	1547.8291	1547.8307	-1.08	2	46	2.5e-005	1	U	F.DKLKHLVDEPQNL.I
2838	374 - 386	774.9218	1547.8291	1547.8307	-1.08	2	49	1.2e-005	1	U	F.DKLKHLVDEPQNL.I
2839	374 - 386	774.9219	1547.8292	1547.8307	-0.99	2	51	7.8e-006	1	U	F.DKLKHLVDEPQNL.I
2840	374 - 386	774.9219	1547.8292	1547.8307	-0.99	2	46	2.4e-005	1	U	F.DKLKHLVDEPQNL.I
2841	374 - 386	774.9219	1547.8292	1547.8307	-0.99	2	52	5.8e-006	1	U	F.DKLKHLVDEPQNL.I
2842	374 - 386	774.9219	1547.8293	1547.8307	-0.91	2	51	7.6e-006	1	U	F.DKLKHLVDEPQNL.I
2843	374 - 386	774.9219	1547.8293	1547.8307	-0.91	2	47	1.9e-005	1	U	F.DKLKHLVDEPQNL.I
2844	374 - 386	774.9220	1547.8294	1547.8307	-0.83	2	47	1.9e-005	1	U	F.DKLKHLVDEPQNL.I
2845	374 - 386	774.9221	1547.8297	1547.8307	-0.68	2	45	3.1e-005	1	U	F.DKLKHLVDEPQNL.I
2846	374 - 386	387.9647	1547.8298	1547.8307	-0.62	2	23	0.0051	1	U	F.DKLKHLVDEPQNL.I
2847	374 - 386	774.9222	1547.8298	1547.8307	-0.60	2	46	2.4e-005	1	U	F.DKLKHLVDEPQNL.I
2848	374 - 386	774.9222	1547.8298	1547.8307	-0.60	2	55	3.5e-006	1	U	F.DKLKHLVDEPQNL.I
2849	374 - 386	774.9223	1547.8300	1547.8307	-0.44	2	49	1.2e-005	1	U	F.DKLKHLVDEPQNL.I
2850	374 - 386	516.9507	1547.8304	1547.8307	-0.22	2	50	9.8e-006	1	U	F.DKLKHLVDEPQNL.I
2851	374 - 386	387.9649	1547.8305	1547.8307	-0.15	2	19	0.013	1	U	F.DKLKHLVDEPQNL.I
2852	374 - 386	516.9508	1547.8306	1547.8307	-0.085	2	35	0.00035	1	U	F.DKLKHLVDEPQNL.I
2853	374 - 386	516.9508	1547.8306	1547.8307	-0.085	2	25	0.0029	1	U	F.DKLKHLVDEPQNL.I
2854	374 - 386	516.9509	1547.8308	1547.8307	0.031	2	34	0.00036	1	U	F.DKLKHLVDEPQNL.I
2855	374 - 386	516.9509	1547.8308	1547.8307	0.031	2	38	0.00017	1	U	F.DKLKHLVDEPQNL.I
2856	374 - 386	516.9509	1547.8310	1547.8307	0.15	2	27	0.0018	1	U	F.DKLKHLVDEPQNL.I
2857	374 - 386	516.9509	1547.8310	1547.8307	0.15	2	37	0.0002	1	U	F.DKLKHLVDEPQNL.I
2858	374 - 386	516.9510	1547.8311	1547.8307	0.26	2	34	0.00037	1	U	F.DKLKHLVDEPQNL.I
2859	374 - 386	516.9510	1547.8311	1547.8307	0.26	2	35	0.00034	1	U	F.DKLKHLVDEPQNL.I
2860	374 - 386	516.9511	1547.8313	1547.8307	0.38	2	25	0.0033	1	U	F.DKLKHLVDEPQNL.I
2861	374 - 386	516.9511	1547.8313	1547.8307	0.38	2	38	0.00014	1	U	F.DKLKHLVDEPQNL.I
2862	374 - 386	516.9511	1547.8315	1547.8307	0.50	2	41	8.7e-005	1	U	F.DKLKHLVDEPQNL.I
2863	374 - 386	516.9512	1547.8319	1547.8307	0.73	2	31	0.00079	1	U	F.DKLKHLVDEPQNL.I
2864	374 - 386	516.9514	1547.8322	1547.8307	0.96	2	35	0.00032	1	U	F.DKLKHLVDEPQNL.I
2865	374 - 386	516.9515	1547.8326	1547.8307	1.21	2	37	0.00021	1	U	F.DKLKHLVDEPQNL.I
2866	374 - 386	516.9517	1547.8333	1547.8307	1.68	2	35	0.00035	1	U	F.DKLKHLVDEPQNL.I
2867	374 - 386	516.9519	1547.8339	1547.8307	2.03	2	32	0.00065	1	U	F.DKLKHLVDEPQNL.I
3263	374 - 386	855.9479	1709.8813	1709.8836	-1.31	2	19	0.013	1	U	F.DKLKHLVDEPQNL.I + Glycation (K)
3264	374 - 386	855.9484	1709.8823	1709.8836	-0.73	2	16	0.027	1	U	F.DKLKHLVDEPQNL.I + Glycation (K)
3266	374 - 386	855.9486	1709.8827	1709.8836	-0.52	2	20	0.01	1	U	F.DKLKHLVDEPQNL.I + Glycation (K)
3267	374 - 386	855.9486	1709.8827	1709.8836	-0.52	2	28	0.0015	1	U	F.DKLKHLVDEPQNL.I + Glycation (K)
3269	374 - 386	570.9685	1709.8837	1709.8836	0.083	2	19	0.012	1	U	F.DKLKHLVDEPQNL.I + Glycation (K)
3271	374 - 386	570.9686	1709.8841	1709.8836	0.29	2	19	0.012	1	U	F.DKLKHLVDEPQNL.I + Glycation (K)
1597	377 - 386	596.8194	1191.6242	1191.6248	-0.44	1	52	6.3e-006	1	U	L.KHLVDEPQNL.I
1598	377 - 386	398.2154	1191.6244	1191.6248	-0.28	1	73	4.8e-008	1	U	L.KHLVDEPQNL.I
1599	377 - 386	596.8195	1191.6245	1191.6248	-0.24	1	52	6.1e-006	1	U	L.KHLVDEPQNL.I
1600	377 - 386	596.8195	1191.6245	1191.6248	-0.24	1	57	1.9e-006	1	U	L.KHLVDEPQNL.I
1601	377 - 386	596.8195	1191.6245	1191.6248	-0.24	1	54	3.7e-006	1	U	L.KHLVDEPQNL.I
1602	377 - 386	398.2154	1191.6245	1191.6248	-0.21	1	73	4.7e-008	1	U	L.KHLVDEPQNL.I
1603	377 - 386	398.2154	1191.6245	1191.6248	-0.21	1	67	1.9e-007	1	U	L.KHLVDEPQNL.I
1604	377 - 386	398.2154	1191.6245	1191.6248	-0.21	1	73	4.7e-008	1	U	L.KHLVDEPQNL.I
1605	377 - 386	596.8196	1191.6246	1191.6248	-0.14	1	45	2.9e-005	1	U	L.KHLVDEPQNL.I
1606	377 - 386	596.8196	1191.6246	1191.6248	-0.14	1	57	2e-006	1	U	L.KHLVDEPQNL.I
1607	377 - 386	596.8196	1191.6246	1191.6248	-0.14	1	54	3.8e-006	1	U	L.KHLVDEPQNL.I
1608	377 - 386	596.8196	1191.6246	1191.6248	-0.14	1	54	3.8e-006	1	U	L.KHLVDEPQNL.I
1609	377 - 386	398.2155	1191.6246	1191.6248	-0.13	1	73	4.7e-008	1	U	L.KHLVDEPQNL.I
1610	377 - 386	596.8196	1191.6247	1191.6248	-0.036	1	62	6.6e-007	1	U	L.KHLVDEPQNL.I
1611	377 - 386	596.8196	1191.6247	1191.6248	-0.036	1	57	1.9e-006	1	U	L.KHLVDEPQNL.I
1612	377 - 386	596.8196	1191.6247	1191.6248	-0.036	1	62	6.6e-007	1	U	L.KHLVDEPQNL.I
1613	377 - 386	398.2155	1191.6247	1191.6248	-0.033	1	53	4.9e-006	1	U	L.KHLVDEPQNL.I
1614	377 - 386	398.2156	1191.6248	1191.6248	0.043	1	73	4.7e-008	1	U	L.KHLVDEPQNL.I
1615	377 - 386	398.2156	1191.6248	1191.6248	0.043	1	73	4.7e-008	1	U	L.KHLVDEPQNL.I
1616	377 - 386	596.8197	1191.6248	1191.6248	0.065	1	26	0.0026	1	U	L.KHLVDEPQNL.I
1617	377 - 386	596.8198	1191.6250	1191.6248	0.17	1	51	7.2e-006	1	U	L.KHLVDEPQNL.I
1618	377 - 386	596.8198	1191.6250	1191.6248	0.17	1	52	6.2e-006	1	U	L.KHLVDEPQNL.I
1619	377 - 386	398.2156	1191.6250	1191.6248	0.19	1	73	4.8e-008	1	U	L.KHLVDEPQNL.I
1620	377 - 386	596.8198	1191.6251	1191.6248	0.27	1	57	1.9e-006	1	U	L.KHLVDEPQNL.I
1621	377 - 386	398.2156	1191.6251	1191.6248	0.27	1	73	4.9e-008	1	U	L.KHLVDEPQNL.I
1622	377 - 386	596.8199	1191.6253	1191.6248	0.48	1	47	2e-005	1	U	L.KHLVDEPQNL.I
1623	377 - 386	398.2160	1191.6261	1191.6248	1.10	1	41	8.4e-005	1	U	L.KHLVDEPQNL.I
1624	377 - 386	398.2161	1191.6266	1191.6248	1.50	1	67	2.1e-007	1	U	L.KHLVDEPQNL.I
2216	377 - 386	677.8458	1353.6770	1353.6776	-0.46	1	23	0.0045	1	U	L.KHLVDEPQNL.I + Glycation (K)
2217	377 - 386	677.8458	1353.6770	1353.6776	-0.46	1	17	0.022	1	U	L.KHLVDEPQNL.I + Glycation (K)
2218	377 - 386	677.8458	1353.6770	1353.6776	-0.46	1	21	0.0075	1	U	L.KHLVDEPQNL.I + Glycation (K)
2219	377 - 386	677.8458	1353.6771	1353.6776	-0.36	1	18	0.015	1	U	L.KHLVDEPQNL.I + Glycation (K)
2220	377 - 386	452.2330	1353.6773	1353.6776	-0.25	1	25	0.003	1	U	L.KHLVDEPQNL.I + Glycation (K)

Query	Start - End	Observed	Mr(expt)	Mr(calc)	ppm	M	Score	Expect	Rank	U	Peptide
2221	377 - 386	677.8460	1353.6773	1353.6776	-0.18	1	18	0.014	1	U	L.KHLVDEPQNL.I + Glycation (K)
2222	377 - 386	677.8460	1353.6773	1353.6776	-0.18	1	17	0.019	1	U	L.KHLVDEPQNL.I + Glycation (K)
2224	377 - 386	452.2332	1353.6776	1353.6776	0.018	1	20	0.01	1	U	L.KHLVDEPQNL.I + Glycation (K)
1188	387 - 394	526.7449	1051.4753	1051.4757	-0.35	0	23	0.0048	1	U	L.IKQNCDQF.E
1189	387 - 394	526.7451	1051.4756	1051.4757	-0.10	0	24	0.0036	1	U	L.IKQNCDQF.E
1190	387 - 394	526.7451	1051.4757	1051.4757	0.010	0	25	0.0035	1	U	L.IKQNCDQF.E
1191	387 - 394	526.7451	1051.4757	1051.4757	0.010	0	14	0.043	1	U	L.IKQNCDQF.E
1192	387 - 394	526.7452	1051.4758	1051.4757	0.12	0	17	0.019	1	U	L.IKQNCDQF.E
1193	387 - 394	526.7452	1051.4758	1051.4757	0.12	0	23	0.0048	1	U	L.IKQNCDQF.E
1194	387 - 394	526.7452	1051.4759	1051.4757	0.24	0	23	0.0049	1	U	L.IKQNCDQF.E
1195	387 - 394	526.7452	1051.4759	1051.4757	0.24	0	23	0.0047	1	U	L.IKQNCDQF.E
1196	387 - 394	526.7452	1051.4759	1051.4757	0.24	0	23	0.0055	1	U	L.IKQNCDQF.E
1197	387 - 394	526.7452	1051.4759	1051.4757	0.24	0	14	0.037	1	U	L.IKQNCDQF.E
1198	387 - 394	526.7453	1051.4760	1051.4757	0.35	0	21	0.0077	1	U	L.IKQNCDQF.E
1199	387 - 394	526.7454	1051.4762	1051.4757	0.47	0	23	0.0048	1	U	L.IKQNCDQF.E
3402	387 - 400	886.4186	1770.8226	1770.8247	-1.16	2	43	5.2e-005	1	U	L.IKQNCDQFEKLGEY.G
3403	387 - 400	591.2815	1770.8226	1770.8247	-1.14	2	16	0.027	1	U	L.IKQNCDQFEKLGEY.G
3404	387 - 400	886.4186	1770.8227	1770.8247	-1.09	2	33	0.00055	1	U	L.IKQNCDQFEKLGEY.G
3405	387 - 400	886.4187	1770.8228	1770.8247	-1.02	2	33	0.00047	1	U	L.IKQNCDQFEKLGEY.G
3406	387 - 400	886.4187	1770.8228	1770.8247	-1.02	2	41	7.2e-005	1	U	L.IKQNCDQFEKLGEY.G
3407	387 - 400	886.4188	1770.8231	1770.8247	-0.89	2	42	6.5e-005	1	U	L.IKQNCDQFEKLGEY.G
3408	387 - 400	886.4189	1770.8232	1770.8247	-0.82	2	35	0.00033	1	U	L.IKQNCDQFEKLGEY.G
3409	387 - 400	886.4189	1770.8232	1770.8247	-0.82	2	45	3.3e-005	1	U	L.IKQNCDQFEKLGEY.G
3410	387 - 400	591.2817	1770.8232	1770.8247	-0.82	2	29	0.0012	1	U	L.IKQNCDQFEKLGEY.G
3411	387 - 400	886.4190	1770.8233	1770.8247	-0.74	2	36	0.00023	1	U	L.IKQNCDQFEKLGEY.G
3412	387 - 400	886.4190	1770.8235	1770.8247	-0.67	2	36	0.00026	1	U	L.IKQNCDQFEKLGEY.G
3413	387 - 400	886.4191	1770.8236	1770.8247	-0.61	2	31	0.0008	1	U	L.IKQNCDQFEKLGEY.G
3414	387 - 400	886.4192	1770.8238	1770.8247	-0.47	2	36	0.00026	1	U	L.IKQNCDQFEKLGEY.G
3415	387 - 400	886.4195	1770.8244	1770.8247	-0.13	2	24	0.0043	1	U	L.IKQNCDQFEKLGEY.G
3416	387 - 400	591.2822	1770.8247	1770.8247	0.012	2	20	0.01	1	U	L.IKQNCDQFEKLGEY.G
3417	387 - 400	886.4197	1770.8248	1770.8247	0.083	2	15	0.035	1	U	L.IKQNCDQFEKLGEY.G
3418	387 - 400	886.4197	1770.8248	1770.8247	0.083	2	24	0.004	1	U	L.IKQNCDQFEKLGEY.G
3419	387 - 400	591.2822	1770.8249	1770.8247	0.11	2	20	0.0099	1	U	L.IKQNCDQFEKLGEY.G
3420	387 - 400	591.2822	1770.8249	1770.8247	0.11	2	27	0.002	1	U	L.IKQNCDQFEKLGEY.G
3421	387 - 400	591.2823	1770.8250	1770.8247	0.22	2	18	0.015	1	U	L.IKQNCDQFEKLGEY.G
3422	387 - 400	591.2823	1770.8250	1770.8247	0.22	2	15	0.03	1	U	L.IKQNCDQFEKLGEY.G
3423	387 - 400	591.2823	1770.8252	1770.8247	0.32	2	27	0.0022	1	U	L.IKQNCDQFEKLGEY.G
3424	387 - 400	591.2823	1770.8252	1770.8247	0.32	2	26	0.0022	1	U	L.IKQNCDQFEKLGEY.G
3425	387 - 400	591.2823	1770.8252	1770.8247	0.32	2	37	0.0002	1	U	L.IKQNCDQFEKLGEY.G
3426	387 - 400	591.2824	1770.8254	1770.8247	0.42	2	19	0.013	1	U	L.IKQNCDQFEKLGEY.G
3427	387 - 400	591.2824	1770.8254	1770.8247	0.42	2	40	0.0001	1	U	L.IKQNCDQFEKLGEY.G
3428	387 - 400	591.2824	1770.8254	1770.8247	0.42	2	37	0.00022	1	U	L.IKQNCDQFEKLGEY.G
3429	387 - 400	591.2824	1770.8254	1770.8247	0.42	2	36	0.00023	1	U	L.IKQNCDQFEKLGEY.G
3430	387 - 400	591.2826	1770.8259	1770.8247	0.72	2	29	0.0014	1	U	L.IKQNCDQFEKLGEY.G
3431	387 - 400	591.2826	1770.8259	1770.8247	0.72	2	37	0.00021	1	U	L.IKQNCDQFEKLGEY.G
3432	387 - 400	591.2829	1770.8269	1770.8247	1.25	2	37	0.00022	1	U	L.IKQNCDQFEKLGEY.G
3709	387 - 400	967.4448	1932.8751	1932.8775	-1.24	2	27	0.0021	1	U	L.IKQNCDQFEKLGEY.G + Glycation (K)
3710	387 - 400	967.4450	1932.8755	1932.8775	-1.04	2	26	0.0023	1	U	L.IKQNCDQFEKLGEY.G + Glycation (K)
3712	387 - 400	967.4451	1932.8756	1932.8775	-0.98	2	26	0.0025	1	U	L.IKQNCDQFEKLGEY.G + Glycation (K)
3713	387 - 400	967.4451	1932.8757	1932.8775	-0.92	2	24	0.0041	1	U	L.IKQNCDQFEKLGEY.G + Glycation (K)
3714	387 - 400	967.4454	1932.8762	1932.8775	-0.67	2	25	0.0035	1	U	L.IKQNCDQFEKLGEY.G + Glycation (K)
3715	387 - 400	645.2994	1932.8763	1932.8775	-0.61	2	32	0.0006	1	U	L.IKQNCDQFEKLGEY.G + Glycation (K)
3716	387 - 400	645.2994	1932.8765	1932.8775	-0.51	2	32	0.00057	1	U	L.IKQNCDQFEKLGEY.G + Glycation (K)
3717	387 - 400	645.2996	1932.8770	1932.8775	-0.24	2	29	0.0012	1	U	L.IKQNCDQFEKLGEY.G + Glycation (K)
3718	387 - 400	967.4458	1932.8770	1932.8775	-0.23	2	26	0.0025	1	U	L.IKQNCDQFEKLGEY.G + Glycation (K)
3719	387 - 400	645.2997	1932.8772	1932.8775	-0.14	2	15	0.033	1	U	L.IKQNCDQFEKLGEY.G + Glycation (K)
4069	411 - 429	699.7378	2096.1915	2096.1954	-1.82	1	53	5.2e-006	1	U	Y.TRKVPQVSTPTLVEVSRSL.G
4070	411 - 429	699.7386	2096.1939	2096.1954	-0.68	1	72	6.3e-008	1	U	Y.TRKVPQVSTPTLVEVSRSL.G
439	423 - 429	395.2267	788.4387	788.4392	-0.58	0	20	0.0091	1	U	L.VEVSRSL.G
440	423 - 429	395.2267	788.4388	788.4392	-0.51	0	24	0.0043	1	U	L.VEVSRSL.G
441	423 - 429	395.2267	788.4388	788.4392	-0.51	0	22	0.007	1	U	L.VEVSRSL.G
442	423 - 429	395.2267	788.4389	788.4392	-0.33	0	21	0.0089	1	U	L.VEVSRSL.G
443	423 - 429	395.2268	788.4391	788.4392	-0.18	0	24	0.0041	1	U	L.VEVSRSL.G
444	423 - 429	395.2268	788.4391	788.4392	-0.10	0	24	0.0037	1	U	L.VEVSRSL.G
445	423 - 429	395.2269	788.4393	788.4392	0.13	0	20	0.0092	1	U	L.VEVSRSL.G
446	423 - 429	395.2270	788.4394	788.4392	0.28	0	21	0.0079	1	U	L.VEVSRSL.G
447	423 - 429	395.2271	788.4397	788.4392	0.66	0	24	0.0043	1	U	L.VEVSRSL.G
2775	488 - 501	769.8896	1537.7647	1537.7664	-1.09	2	37	0.00019	1	U	F.SALTPDETYVPKAF.D
2776	488 - 501	769.8897	1537.7649	1537.7664	-1.01	2	29	0.0014	1	U	F.SALTPDETYVPKAF.D
2777	488 - 501	769.8897	1537.7649	1537.7664	-1.01	2	35	0.00028	1	U	F.SALTPDETYVPKAF.D
2778	488 - 501	769.8897	1537.7649	1537.7664	-1.01	2	35	0.00033	1	U	F.SALTPDETYVPKAF.D
2779	488 - 501	769.8898	1537.7651	1537.7664	-0.85	2	29	0.0013	1	U	F.SALTPDETYVPKAF.D
2780	488 - 501	769.8898	1537.7651	1537.7664	-0.85	2	42	6.6e-005	1	U	F.SALTPDETYVPKAF.D
2781	488 - 501	769.8899	1537.7652	1537.7664	-0.77	2	28	0.0016	1	U	F.SALTPDETYVPKAF.D
2782	488 - 501	769.8899	1537.7652	1537.7664	-0.77	2	26	0.0025	1	U	F.SALTPDETYVPKAF.D
2783	488 - 501	769.8899	1537.7652	1537.7664	-0.77	2	35	0.00029	1	U	F.SALTPDETYVPKAF.D
2784	488 - 501	769.8900	1537.7653	1537.7664	-0.70	2	28	0.0015	1	U	F.SALTPDETYVPKAF.D
2785	488 - 501	769.8900	1537.7655	1537.7664	-0.62	2	38	0.00018	1	U	F.SALTPDETYVPKAF.D
2786	488 - 501	769.8900	1537.7655	1537.7664	-0.62	2	36	0.00027	1	U	F.SALTPDETYVPKAF.D
2787	488 - 501	769.8901	1537.7656	1537.7664	-0.53	2	25	0.0031	1	U	F.SALTPDETYVPKAF.D
2788	488 - 501	769.8901	1537.7656	1537.7664	-0.53	2	36	0.00027	1	U	F.SALTPDETYVPKAF.D
2789	488 - 501	769.8901	1537.7657	1537.7664	-0.45	2	32	0.0007	1	U	F.SALTPDETYVPKAF.D
2790	488 - 501	769.8902	1537.7658	1537.7664	-0.37	2	33	0.00052	1	U	F.SALTPDETYVPKAF.D
2791	488 - 501	513.5961	1537.7664	1537.7664	-0.023	2	21	0.0081	1	U	F.SALTPDETYVPKAF.D
2792	488 - 501	769.8905	1537.7664	1537.7664	0.020	2	13	0.05	1	U	F.SALTPDETYVPKAF.D
3259	488 - 501	850.9164	1699.8183	1699.8192	-0.54	2	14	0.04	1	U	F.SALTPDETYVPKAF.D + Glycation (K)
3877	488 - 505	675.3463	2023.0171	2023.0150	1.05	3	32	0.0006	1	U	F.SALTPDETYVPKAFDEKL.F
4191	488 - 506	1086.0476	2170.0807	2170.0834	-1.25	4	30	0.0011	1	U	F.SALTPDETYVPKAFDEKLF.T
4192	488 - 506	724.3679	2170.0818	2170.0834	-0.75	4	24	0.0043	1	U	F.SALTPDETYVPKAFDEKLF.T
4193	488 - 506	724.3679	2170.0819	2170.0834	-0.67	4	30	0.001	1	U	F.SALTPDETYVPKAFDEKLF.T

Query	Start - End	Observed	Mr(expt)	Mr(calc)	ppm	M	Score	Expect	Rank	U	Peptide
4194	488 - 506	724.3680	2170.0821	2170.0834	-0.59	4	20	0.011	1	U	F.SALTPDETYVPKAFDEKLF.T
4195	488 - 506	724.3680	2170.0821	2170.0834	-0.59	4	28	0.0016	1	U	F.SALTPDETYVPKAFDEKLF.T
4196	488 - 506	724.3680	2170.0821	2170.0834	-0.59	4	24	0.0043	1	U	F.SALTPDETYVPKAFDEKLF.T
4197	488 - 506	724.3680	2170.0821	2170.0834	-0.59	4	23	0.0046	1	U	F.SALTPDETYVPKAFDEKLF.T
4198	488 - 506	724.3680	2170.0823	2170.0834	-0.51	4	20	0.0096	1	U	F.SALTPDETYVPKAFDEKLF.T
4199	488 - 506	724.3680	2170.0823	2170.0834	-0.51	4	23	0.0049	1	U	F.SALTPDETYVPKAFDEKLF.T
4200	488 - 506	724.3680	2170.0823	2170.0834	-0.51	4	26	0.0025	1	U	F.SALTPDETYVPKAFDEKLF.T
4201	488 - 506	724.3681	2170.0825	2170.0834	-0.42	4	30	0.00097	1	U	F.SALTPDETYVPKAFDEKLF.T
4202	488 - 506	724.3682	2170.0827	2170.0834	-0.34	4	30	0.00099	1	U	F.SALTPDETYVPKAFDEKLF.T
4203	488 - 506	724.3682	2170.0829	2170.0834	-0.24	4	30	0.0009	1	U	F.SALTPDETYVPKAFDEKLF.T
4204	488 - 506	1086.0487	2170.0829	2170.0834	-0.24	4	27	0.002	1	U	F.SALTPDETYVPKAFDEKLF.T
4205	488 - 506	1086.0487	2170.0829	2170.0834	-0.24	4	31	0.00072	1	U	F.SALTPDETYVPKAFDEKLF.T
4206	488 - 506	1086.0487	2170.0829	2170.0834	-0.24	4	26	0.0023	1	U	F.SALTPDETYVPKAFDEKLF.T
4207	488 - 506	1086.0487	2170.0829	2170.0834	-0.24	4	25	0.0029	1	U	F.SALTPDETYVPKAFDEKLF.T
4208	488 - 506	724.3683	2170.0830	2170.0834	-0.16	4	24	0.0037	1	U	F.SALTPDETYVPKAFDEKLF.T
4209	488 - 506	1086.0488	2170.0831	2170.0834	-0.13	4	33	0.00045	1	U	F.SALTPDETYVPKAFDEKLF.T
4210	488 - 506	1086.0488	2170.0831	2170.0834	-0.13	4	31	0.00078	1	U	F.SALTPDETYVPKAFDEKLF.T
4211	488 - 506	724.3683	2170.0832	2170.0834	-0.077	4	18	0.014	1	U	F.SALTPDETYVPKAFDEKLF.T
4212	488 - 506	1086.0490	2170.0833	2170.0834	-0.019	4	29	0.0012	1	U	F.SALTPDETYVPKAFDEKLF.T
4214	488 - 506	1086.0490	2170.0835	2170.0834	0.064	4	29	0.0013	1	U	F.SALTPDETYVPKAFDEKLF.T
4215	488 - 506	724.3685	2170.0836	2170.0834	0.089	4	20	0.0093	1	U	F.SALTPDETYVPKAFDEKLF.T
4216	488 - 506	724.3685	2170.0836	2170.0834	0.089	4	30	0.00097	1	U	F.SALTPDETYVPKAFDEKLF.T
4218	488 - 506	724.3687	2170.0843	2170.0834	0.42	4	15	0.032	1	U	F.SALTPDETYVPKAFDEKLF.T
1893	491 - 501	634.3136	1266.6126	1266.6132	-0.45	1	50	1.1e-005	1	U	L.TPDETYVPKAF.D
1894	491 - 501	634.3137	1266.6129	1266.6132	-0.26	1	33	0.00047	1	U	L.TPDETYVPKAF.D
1895	491 - 501	634.3137	1266.6129	1266.6132	-0.26	1	42	6.6e-005	1	U	L.TPDETYVPKAF.D
1896	491 - 501	634.3142	1266.6137	1266.6132	0.42	1	32	0.0006	1	U	L.TPDETYVPKAF.D
3664	491 - 506	633.9838	1898.9295	1898.9302	-0.39	3	18	0.015	1	U	L.TPDETYVPKAFDEKLF.T
3665	491 - 506	950.4721	1898.9297	1898.9302	-0.28	3	25	0.0029	1	U	L.TPDETYVPKAFDEKLF.T
3666	491 - 506	633.9839	1898.9298	1898.9302	-0.18	3	19	0.012	1	U	L.TPDETYVPKAFDEKLF.T
3667	491 - 506	633.9839	1898.9298	1898.9302	-0.18	3	27	0.0019	1	U	L.TPDETYVPKAFDEKLF.T
3668	491 - 506	950.4727	1898.9309	1898.9302	0.37	3	22	0.0058	1	U	L.TPDETYVPKAFDEKLF.T
3669	491 - 506	633.9846	1898.9320	1898.9302	0.97	3	24	0.004	1	U	L.TPDETYVPKAFDEKLF.T
1626	497 - 506	597.3317	1192.6488	1192.6492	-0.35	2	29	0.0012	1	U	Y.VPKAFDEKLF.T
1627	497 - 506	597.3317	1192.6488	1192.6492	-0.35	2	34	0.00038	1	U	Y.VPKAFDEKLF.T
1628	497 - 506	597.3317	1192.6488	1192.6492	-0.35	2	28	0.0016	1	U	Y.VPKAFDEKLF.T
1629	497 - 506	597.3317	1192.6489	1192.6492	-0.24	2	31	0.00079	1	U	Y.VPKAFDEKLF.T
1630	497 - 506	398.5569	1192.6489	1192.6492	-0.22	2	19	0.014	1	U	Y.VPKAFDEKLF.T
1631	497 - 506	398.5569	1192.6489	1192.6492	-0.22	2	17	0.02	1	U	Y.VPKAFDEKLF.T
1632	497 - 506	398.5569	1192.6490	1192.6492	-0.15	2	17	0.021	1	U	Y.VPKAFDEKLF.T
1633	497 - 506	597.3318	1192.6490	1192.6492	-0.14	2	29	0.0012	1	U	Y.VPKAFDEKLF.T
1634	497 - 506	398.5570	1192.6491	1192.6492	-0.074	2	18	0.017	1	U	Y.VPKAFDEKLF.T
1635	497 - 506	398.5570	1192.6491	1192.6492	-0.074	2	20	0.0097	1	U	Y.VPKAFDEKLF.T
1636	497 - 506	398.5570	1192.6491	1192.6492	-0.074	2	20	0.011	1	U	Y.VPKAFDEKLF.T
1637	497 - 506	597.3319	1192.6491	1192.6492	-0.044	2	30	0.0009	1	U	Y.VPKAFDEKLF.T
1638	497 - 506	597.3319	1192.6491	1192.6492	-0.044	2	30	0.00092	1	U	Y.VPKAFDEKLF.T
1639	497 - 506	597.3319	1192.6491	1192.6492	-0.044	2	31	0.00079	1	U	Y.VPKAFDEKLF.T
1640	497 - 506	398.5570	1192.6492	1192.6492	0.0017	2	17	0.018	1	U	Y.VPKAFDEKLF.T
1641	497 - 506	398.5570	1192.6492	1192.6492	0.0017	2	19	0.013	1	U	Y.VPKAFDEKLF.T
1642	497 - 506	398.5570	1192.6492	1192.6492	0.0017	2	19	0.013	1	U	Y.VPKAFDEKLF.T
1643	497 - 506	597.3319	1192.6493	1192.6492	0.057	2	25	0.0032	1	U	Y.VPKAFDEKLF.T
1644	497 - 506	597.3319	1192.6493	1192.6492	0.057	2	32	0.00067	1	U	Y.VPKAFDEKLF.T
1645	497 - 506	597.3319	1192.6493	1192.6492	0.057	2	29	0.0011	1	U	Y.VPKAFDEKLF.T
1646	497 - 506	398.5570	1192.6493	1192.6492	0.077	2	15	0.03	1	U	Y.VPKAFDEKLF.T
1647	497 - 506	398.5571	1192.6494	1192.6492	0.15	2	19	0.014	1	U	Y.VPKAFDEKLF.T
1648	497 - 506	398.5571	1192.6494	1192.6492	0.15	2	19	0.013	1	U	Y.VPKAFDEKLF.T
1649	497 - 506	398.5571	1192.6495	1192.6492	0.23	2	16	0.025	1	U	Y.VPKAFDEKLF.T
1650	497 - 506	597.3320	1192.6495	1192.6492	0.26	2	36	0.00025	1	U	Y.VPKAFDEKLF.T
1651	497 - 506	398.5571	1192.6496	1192.6492	0.30	2	20	0.011	1	U	Y.VPKAFDEKLF.T
1652	497 - 506	398.5572	1192.6497	1192.6492	0.45	2	20	0.0092	1	U	Y.VPKAFDEKLF.T
2225	497 - 506	678.3578	1354.7011	1354.7020	-0.65	2	17	0.019	1	U	Y.VPKAFDEKLF.T + Glycation (K)
2226	497 - 506	678.3582	1354.7017	1354.7020	-0.21	2	19	0.013	1	U	Y.VPKAFDEKLF.T + Glycation (K)
2227	497 - 506	452.5746	1354.7019	1354.7020	-0.084	2	17	0.022	1	U	Y.VPKAFDEKLF.T + Glycation (K)
2228	497 - 506	452.5746	1354.7019	1354.7020	-0.084	2	22	0.0065	1	U	Y.VPKAFDEKLF.T + Glycation (K)
153	502 - 506	326.1709	650.3272	650.3275	-0.53	1	13	0.045	1	U	F.DEKLF.T
154	502 - 506	326.1709	650.3272	650.3275	-0.53	1	13	0.045	1	U	F.DEKLF.T
156	502 - 506	326.1709	650.3272	650.3275	-0.53	1	14	0.038	1	U	F.DEKLF.T
157	502 - 506	326.1709	650.3272	650.3275	-0.53	1	15	0.032	1	U	F.DEKLF.T
159	502 - 506	326.1709	650.3272	650.3275	-0.43	1	15	0.033	1	U	F.DEKLF.T
161	502 - 506	326.1709	650.3272	650.3275	-0.43	1	14	0.039	1	U	F.DEKLF.T
162	502 - 506	326.1709	650.3273	650.3275	-0.34	1	15	0.034	1	U	F.DEKLF.T
163	502 - 506	326.1709	650.3273	650.3275	-0.34	1	15	0.033	1	U	F.DEKLF.T
166	502 - 506	326.1710	650.3274	650.3275	-0.16	1	14	0.039	1	U	F.DEKLF.T
4787	507 - 528	680.8505	2719.3730	2719.3738	-0.32	1	15	0.03	1	U	F.TFHADICTLPDTEKQIKKQTAL.V + Glycation
5231	507 - 532	635.7374	3173.6508	3173.6530	-0.69	3	36	0.00027	1	U	F.TFHADICTLPDTEKQIKKQTALVELL.K + Glyca
5232	507 - 532	635.7376	3173.6517	3173.6530	-0.41	3	38	0.00018	1	U	F.TFHADICTLPDTEKQIKKQTALVELL.K + Glyca
5233	507 - 532	794.4203	3173.6521	3173.6530	-0.29	3	38	0.00016	1	U	F.TFHADICTLPDTEKQIKKQTALVELL.K + Glyca
5234	507 - 532	635.7378	3173.6526	3173.6530	-0.13	3	19	0.012	1	U	F.TFHADICTLPDTEKQIKKQTALVELL.K + Glyca
5235	507 - 532	635.7380	3173.6535	3173.6530	0.17	3	26	0.0026	1	U	F.TFHADICTLPDTEKQIKKQTALVELL.K + Glyca
5236	507 - 532	794.4208	3173.6540	3173.6530	0.33	3	36	0.00023	1	U	F.TFHADICTLPDTEKQIKKQTALVELL.K + Glyca
5237	507 - 532	1058.8920	3173.6541	3173.6530	0.35	3	30	0.0011	1	U	F.TFHADICTLPDTEKQIKKQTALVELL.K + Glyca
5238	507 - 532	1058.8923	3173.6552	3173.6530	0.69	3	29	0.0012	1	U	F.TFHADICTLPDTEKQIKKQTALVELL.K + Glyca
5239	507 - 532	794.4216	3173.6574	3173.6530	1.40	3	40	0.0001	1	U	F.TFHADICTLPDTEKQIKKQTALVELL.K + Glyca
5240	507 - 532	794.4230	3173.6630	3173.6530	3.17	3	29	0.0013	1	U	F.TFHADICTLPDTEKQIKKQTALVELL.K + Glyca
4440	509 - 528	770.7401	2309.1985	2309.2049	-2.77	0	55	3.3e-006	1	U	F.HADICTLPDTEKQIKKQTAL.V
4441	509 - 528	770.7411	2309.2014	2309.2049	-1.50	0	14	0.043	1	U	F.HADICTLPDTEKQIKKQTAL.V
4442	509 - 528	578.3080	2309.2028	2309.2049	-0.91	0	30	0.0009	1	U	F.HADICTLPDTEKQIKKQTAL.V
4443	509 - 528	578.3084	2309.2043	2309.2049	-0.27	0	46	2.7e-005	1	U	F.HADICTLPDTEKQIKKQTAL.V
4655	509 - 528	618.8206	2471.2534	2471.2577	-1.76	0	34	0.0004	1	U	F.HADICTLPDTEKQIKKQTAL.V + Glycation ([illegible]
4656	509 - 528	618.8207	2471.2539	2471.2577	-1.57	0	33	0.00047	1	U	F.HADICTLPDTEKQIKKQTAL.V + Glycation ([illegible]

Query	Start - End	Observed	Mr(expt)	Mr(calc)	ppm	M	Score	Expect	Rank	U	Peptide
4657	509 - 528	824.7590	2471.2553	2471.2577	-1.00	0	46	2.8e-005	1	U	F.HADICTLPDTEKQIKKQTAL.V + Glycation (K
4658	509 - 528	824.7592	2471.2557	2471.2577	-0.84	0	54	4e-006	1	U	F.HADICTLPDTEKQIKKQTAL.V + Glycation (K
4659	509 - 528	824.7593	2471.2560	2471.2577	-0.70	0	51	8.9e-006	1	U	F.HADICTLPDTEKQIKKQTAL.V + Glycation (K
4660	509 - 528	618.8214	2471.2565	2471.2577	-0.49	0	30	0.00096	1	U	F.HADICTLPDTEKQIKKQTAL.V + Glycation (K
4661	509 - 528	618.8215	2471.2570	2471.2577	-0.29	0	28	0.0017	1	U	F.HADICTLPDTEKQIKKQTAL.V + Glycation (K
4662	509 - 528	824.7597	2471.2573	2471.2577	-0.19	0	34	0.00044	1	U	F.HADICTLPDTEKQIKKQTAL.V + Glycation (K
4663	509 - 528	618.8218	2471.2580	2471.2577	0.11	0	14	0.038	1	U	F.HADICTLPDTEKQIKKQTAL.V + Glycation (K
4664	509 - 528	618.8218	2471.2580	2471.2577	0.11	0	17	0.021	1	U	F.HADICTLPDTEKQIKKQTAL.V + Glycation (K
4750	509 - 531	663.6077	2650.4018	2650.4000	0.69	1	14	0.04	1	U	F.HADICTLPDTEKQIKKQTALVEL.L
4898	509 - 531	938.4897	2812.4472	2812.4528	-1.98	1	53	5.5e-006	1	U	F.HADICTLPDTEKQIKKQTALVEL.L + Glycation
4899	509 - 531	704.1195	2812.4489	2812.4528	-1.38	1	13	0.048	1	U	F.HADICTLPDTEKQIKKQTALVEL.L + Glycation
4900	509 - 531	704.1207	2812.4536	2812.4528	0.27	1	22	0.0059	1	U	F.HADICTLPDTEKQIKKQTALVEL.L + Glycation
4813	509 - 532	922.1667	2763.4784	2763.4840	-2.03	2	38	0.00017	1	U	F.HADICTLPDTEKQIKKQTALVELL.K
4814	509 - 532	922.1669	2763.4788	2763.4840	-1.90	2	35	0.0003	1	U	F.HADICTLPDTEKQIKKQTALVELL.K
4815	509 - 532	691.8774	2763.4807	2763.4840	-1.22	2	26	0.0023	1	U	F.HADICTLPDTEKQIKKQTALVELL.K
4816	509 - 532	691.8776	2763.4811	2763.4840	-1.05	2	32	0.00067	1	U	F.HADICTLPDTEKQIKKQTALVELL.K
4817	509 - 532	922.1681	2763.4824	2763.4840	-0.58	2	39	0.00014	1	U	F.HADICTLPDTEKQIKKQTALVELL.K
4818	509 - 532	691.8779	2763.4826	2763.4840	-0.51	2	28	0.0017	1	U	F.HADICTLPDTEKQIKKQTALVELL.K
4819	509 - 532	922.1682	2763.4828	2763.4840	-0.45	2	36	0.00026	1	U	F.HADICTLPDTEKQIKKQTALVELL.K
4820	509 - 532	691.8785	2763.4851	2763.4840	0.37	2	28	0.0017	1	U	F.HADICTLPDTEKQIKKQTALVELL.K
5013	509 - 532	976.1834	2925.5282	2925.5369	-2.95	2	35	0.00033	1	U	F.HADICTLPDTEKQIKKQTALVELL.K + Glycati
5014	509 - 532	976.1848	2925.5324	2925.5369	-1.52	2	29	0.0011	1	U	F.HADICTLPDTEKQIKKQTALVELL.K + Glycati
5015	509 - 532	976.1848	2925.5326	2925.5369	-1.46	2	46	2.7e-005	1	U	F.HADICTLPDTEKQIKKQTALVELL.K + Glycati
5016	509 - 532	732.3904	2925.5327	2925.5369	-1.44	2	19	0.012	1	U	F.HADICTLPDTEKQIKKQTALVELL.K + Glycati
5017	509 - 532	976.1850	2925.5332	2925.5369	-1.26	2	51	7.1e-006	1	U	F.HADICTLPDTEKQIKKQTALVELL.K + Glycati
5018	509 - 532	976.1851	2925.5335	2925.5369	-1.14	2	49	1.2e-005	1	U	F.HADICTLPDTEKQIKKQTALVELL.K + Glycati
5019	509 - 532	732.3908	2925.5341	2925.5369	-0.93	2	22	0.0064	1	U	F.HADICTLPDTEKQIKKQTALVELL.K + Glycati
5020	509 - 532	976.1854	2925.5344	2925.5369	-0.83	2	49	1.2e-005	1	U	F.HADICTLPDTEKQIKKQTALVELL.K + Glycati
5021	509 - 532	976.1857	2925.5352	2925.5369	-0.58	2	55	3e-006	1	U	F.HADICTLPDTEKQIKKQTALVELL.K + Glycati
5022	509 - 532	976.1859	2925.5359	2925.5369	-0.33	2	52	6.4e-006	1	U	F.HADICTLPDTEKQIKKQTALVELL.K + Glycati
5023	509 - 532	732.3913	2925.5361	2925.5369	-0.26	2	51	8.1e-006	1	U	F.HADICTLPDTEKQIKKQTALVELL.K + Glycati
5024	509 - 532	732.3913	2925.5361	2925.5369	-0.26	2	49	1.4e-005	1	U	F.HADICTLPDTEKQIKKQTALVELL.K + Glycati
5025	509 - 532	732.3914	2925.5363	2925.5369	-0.18	2	41	7.3e-005	1	U	F.HADICTLPDTEKQIKKQTALVELL.K + Glycati
5026	509 - 532	732.3914	2925.5363	2925.5369	-0.18	2	46	2.4e-005	1	U	F.HADICTLPDTEKQIKKQTALVELL.K + Glycati
5027	509 - 532	976.1861	2925.5365	2925.5369	-0.13	2	49	1.2e-005	1	U	F.HADICTLPDTEKQIKKQTALVELL.K + Glycati
5028	509 - 532	732.3914	2925.5366	2925.5369	-0.099	2	48	1.5e-005	1	U	F.HADICTLPDTEKQIKKQTALVELL.K + Glycati
5029	509 - 532	732.3915	2925.5368	2925.5369	-0.017	2	51	8.1e-006	1	U	F.HADICTLPDTEKQIKKQTALVELL.K + Glycati
5030	509 - 532	732.3915	2925.5368	2925.5369	-0.017	2	48	1.5e-005	1	U	F.HADICTLPDTEKQIKKQTALVELL.K + Glycati
5031	509 - 532	732.3916	2925.5373	2925.5369	0.15	2	50	1e-005	1	U	F.HADICTLPDTEKQIKKQTALVELL.K + Glycati
5032	509 - 532	976.1866	2925.5379	2925.5369	0.36	2	55	3.2e-006	1	U	F.HADICTLPDTEKQIKKQTALVELL.K + Glycati
5033	509 - 532	732.3922	2925.5395	2925.5369	0.90	2	48	1.5e-005	1	U	F.HADICTLPDTEKQIKKQTALVELL.K + Glycati
5034	509 - 532	1463.7824	2925.5501	2925.5369	4.54	2	53	4.9e-006	1	U	F.HADICTLPDTEKQIKKQTALVELL.K + Glycati
5194	509 - 532	1030.2031	3087.5876	3087.5897	-0.69	2	32	0.00063	1	U	F.HADICTLPDTEKQIKKQTALVELL.K + 2 Glyca
675	544 - 550	434.7151	867.4156	867.4160	-0.53	0	17	0.022	1	U	L.KTVMENF.V
676	544 - 550	434.7151	867.4156	867.4160	-0.46	0	15	0.03	1	U	L.KTVMENF.V
677	544 - 550	434.7151	867.4157	867.4160	-0.40	0	14	0.037	1	U	L.KTVMENF.V
678	544 - 550	434.7151	867.4157	867.4160	-0.40	0	15	0.033	1	U	L.KTVMENF.V
680	544 - 550	434.7152	867.4158	867.4160	-0.26	0	17	0.021	1	U	L.KTVMENF.V
1576	544 - 553	593.3024	1184.5902	1184.5900	0.17	1	41	8.3e-005	1	U	L.KTVMENFVAF.V
3833	551 - 567	669.2853	2004.8340	2004.8380	-1.98	1	32	0.00064	1	U	F.VAFVDKCCAADDKEACF.A
3834	551 - 567	669.2855	2004.8347	2004.8380	-1.62	1	42	5.6e-005	1	U	F.VAFVDKCCAADDKEACF.A
3835	551 - 567	669.2856	2004.8349	2004.8380	-1.53	1	16	0.023	1	U	F.VAFVDKCCAADDKEACF.A
3836	551 - 567	669.2858	2004.8355	2004.8380	-1.25	1	35	0.0003	1	U	F.VAFVDKCCAADDKEACF.A
3837	551 - 567	669.2861	2004.8366	2004.8380	-0.71	1	36	0.00026	1	U	F.VAFVDKCCAADDKEACF.A
3838	551 - 567	1003.4256	2004.8366	2004.8380	-0.67	1	65	3.3e-007	1	U	F.VAFVDKCCAADDKEACF.A
3839	551 - 567	1003.4259	2004.8372	2004.8380	-0.37	1	50	1.1e-005	1	U	F.VAFVDKCCAADDKEACF.A
3840	551 - 567	669.2864	2004.8373	2004.8380	-0.33	1	36	0.00023	1	U	F.VAFVDKCCAADDKEACF.A
3841	551 - 567	1003.4260	2004.8374	2004.8380	-0.31	1	64	4.4e-007	1	U	F.VAFVDKCCAADDKEACF.A
3842	551 - 567	1003.4261	2004.8376	2004.8380	-0.18	1	43	5e-005	1	U	F.VAFVDKCCAADDKEACF.A
3843	551 - 567	1003.4261	2004.8376	2004.8380	-0.18	1	53	4.6e-006	1	U	F.VAFVDKCCAADDKEACF.A
3844	551 - 567	1003.4262	2004.8379	2004.8380	-0.057	1	55	3.1e-006	1	U	F.VAFVDKCCAADDKEACF.A
3845	551 - 567	1003.4263	2004.8381	2004.8380	0.063	1	72	5.8e-008	1	U	F.VAFVDKCCAADDKEACF.A
3846	551 - 567	669.2867	2004.8382	2004.8380	0.11	1	37	0.00022	1	U	F.VAFVDKCCAADDKEACF.A
3847	551 - 567	669.2867	2004.8382	2004.8380	0.11	1	46	2.7e-005	1	U	F.VAFVDKCCAADDKEACF.A
3848	551 - 567	669.2867	2004.8384	2004.8380	0.20	1	36	0.00023	1	U	F.VAFVDKCCAADDKEACF.A
3849	551 - 567	669.2868	2004.8386	2004.8380	0.29	1	33	0.0005	1	U	F.VAFVDKCCAADDKEACF.A
3850	551 - 567	669.2868	2004.8386	2004.8380	0.29	1	37	0.00022	1	U	F.VAFVDKCCAADDKEACF.A
3851	551 - 567	1003.4268	2004.8391	2004.8380	0.55	1	48	1.7e-005	1	U	F.VAFVDKCCAADDKEACF.A
3852	551 - 567	669.2873	2004.8400	2004.8380	1.03	1	36	0.00023	1	U	F.VAFVDKCCAADDKEACF.A
4188	551 - 567	723.3030	2166.8873	2166.8908	-1.62	1	49	1.3e-005	1	U	F.VAFVDKCCAADDKEACF.A + Glycation (K)
4189	551 - 567	723.3038	2166.8897	2166.8908	-0.53	1	21	0.0082	1	U	F.VAFVDKCCAADDKEACF.A + Glycation (K)
3198	554 - 567	844.8381	1687.6616	1687.6640	-1.45	0	31	0.00075	1	U	F.VDKCCAADDKEACF.A
3199	554 - 567	844.8383	1687.6621	1687.6640	-1.15	0	47	1.8e-005	1	U	F.VDKCCAADDKEACF.A
3200	554 - 567	844.8384	1687.6622	1687.6640	-1.08	0	47	1.8e-005	1	U	F.VDKCCAADDKEACF.A
3201	554 - 567	844.8384	1687.6622	1687.6640	-1.08	0	55	3.2e-006	1	U	F.VDKCCAADDKEACF.A
3202	554 - 567	844.8384	1687.6622	1687.6640	-1.08	0	53	5.2e-006	1	U	F.VDKCCAADDKEACF.A
3203	554 - 567	844.8385	1687.6624	1687.6640	-0.94	0	48	1.5e-005	1	U	F.VDKCCAADDKEACF.A
3204	554 - 567	844.8385	1687.6624	1687.6640	-0.94	0	41	8e-005	1	U	F.VDKCCAADDKEACF.A
3205	554 - 567	844.8386	1687.6626	1687.6640	-0.87	0	58	1.4e-006	1	U	F.VDKCCAADDKEACF.A
3206	554 - 567	844.8386	1687.6627	1687.6640	-0.80	0	32	0.00069	1	U	F.VDKCCAADDKEACF.A
3207	554 - 567	844.8386	1687.6627	1687.6640	-0.80	0	48	1.4e-005	1	U	F.VDKCCAADDKEACF.A
3208	554 - 567	563.5615	1687.6627	1687.6640	-0.77	0	24	0.0044	1	U	F.VDKCCAADDKEACF.A
3209	554 - 567	844.8387	1687.6628	1687.6640	-0.73	0	50	1e-005	1	U	F.VDKCCAADDKEACF.A
3210	554 - 567	844.8387	1687.6628	1687.6640	-0.73	0	47	1.9e-005	1	U	F.VDKCCAADDKEACF.A
3211	554 - 567	844.8388	1687.6629	1687.6640	-0.64	0	33	0.00054	1	U	F.VDKCCAADDKEACF.A
3212	554 - 567	563.5616	1687.6631	1687.6640	-0.54	0	14	0.038	1	U	F.VDKCCAADDKEACF.A
3213	554 - 567	563.5617	1687.6633	1687.6640	-0.43	0	36	0.00024	1	U	F.VDKCCAADDKEACF.A
3214	554 - 567	844.8389	1687.6633	1687.6640	-0.43	0	42	6.6e-005	1	U	F.VDKCCAADDKEACF.A
3215	554 - 567	844.8390	1687.6634	1687.6640	-0.36	0	28	0.0014	1	U	F.VDKCCAADDKEACF.A

Query	Start - End	Observed	Mr(expt)	Mr(calc)	ppm	M	Score	Expect	Rank	U	Peptide
3217	554 - 567	563.5618	1687.6635	1687.6640	-0.33	0	21	0.0085	1	U	F.VDKCCAADDKEACF.A
3218	554 - 567	563.5618	1687.6635	1687.6640	-0.33	0	28	0.0014	1	U	F.VDKCCAADDKEACF.A
3219	554 - 567	563.5618	1687.6637	1687.6640	-0.22	0	32	0.00061	1	U	F.VDKCCAADDKEACF.A
3220	554 - 567	563.5619	1687.6638	1687.6640	-0.11	0	26	0.0025	1	U	F.VDKCCAADDKEACF.A
3221	554 - 567	563.5619	1687.6638	1687.6640	-0.11	0	36	0.00028	1	U	F.VDKCCAADDKEACF.A
3222	554 - 567	563.5620	1687.6640	1687.6640	-0.0059	0	32	0.0006	1	U	F.VDKCCAADDKEACF.A
3223	554 - 567	563.5620	1687.6640	1687.6640	-0.0059	0	23	0.0049	1	U	F.VDKCCAADDKEACF.A
3224	554 - 567	563.5620	1687.6640	1687.6640	-0.0059	0	33	0.00045	1	U	F.VDKCCAADDKEACF.A
3225	554 - 567	563.5620	1687.6640	1687.6640	-0.0059	0	35	0.00034	1	U	F.VDKCCAADDKEACF.A
3226	554 - 567	844.8393	1687.6640	1687.6640	-0.0024	0	33	0.00049	1	U	F.VDKCCAADDKEACF.A
3228	554 - 567	563.5620	1687.6642	1687.6640	0.10	0	18	0.017	1	U	F.VDKCCAADDKEACF.A
3229	554 - 567	563.5620	1687.6642	1687.6640	0.10	0	35	0.00034	1	U	F.VDKCCAADDKEACF.A
3230	554 - 567	563.5621	1687.6644	1687.6640	0.21	0	13	0.048	1	U	F.VDKCCAADDKEACF.A
3231	554 - 567	563.5621	1687.6646	1687.6640	0.31	0	16	0.027	1	U	F.VDKCCAADDKEACF.A
3232	554 - 567	563.5621	1687.6646	1687.6640	0.31	0	28	0.0018	1	U	F.VDKCCAADDKEACF.A
3233	554 - 567	563.5622	1687.6647	1687.6640	0.42	0	38	0.00018	1	U	F.VDKCCAADDKEACF.A
3234	554 - 567	563.5622	1687.6647	1687.6640	0.42	0	33	0.00054	1	U	F.VDKCCAADDKEACF.A
3235	554 - 567	563.5623	1687.6650	1687.6640	0.55	0	17	0.021	1	U	F.VDKCCAADDKEACF.A
3236	554 - 567	563.5624	1687.6655	1687.6640	0.87	0	27	0.0019	1	U	F.VDKCCAADDKEACF.A
3237	554 - 567	563.5625	1687.6657	1687.6640	0.97	0	28	0.0015	1	U	F.VDKCCAADDKEACF.A
3569	554 - 567	617.5790	1849.7153	1849.7169	-0.85	0	22	0.0064	1	U	F.VDKCCAADDKEACF.A + Glycation (K)
3570	554 - 567	925.8650	1849.7154	1849.7169	-0.77	0	39	0.00012	1	U	F.VDKCCAADDKEACF.A + Glycation (K)
3571	554 - 567	617.5791	1849.7155	1849.7169	-0.75	0	25	0.0034	1	U	F.VDKCCAADDKEACF.A + Glycation (K)
3572	554 - 567	925.8652	1849.7159	1849.7169	-0.51	0	43	5.6e-005	1	U	F.VDKCCAADDKEACF.A + Glycation (K)
3573	554 - 567	617.5793	1849.7160	1849.7169	-0.46	0	25	0.0034	1	U	F.VDKCCAADDKEACF.A + Glycation (K)
3574	554 - 567	617.5796	1849.7169	1849.7169	0.045	0	27	0.0022	1	U	F.VDKCCAADDKEACF.A + Glycation (K)
291	568 - 574	357.2131	712.4117	712.4119	-0.31	0	20	0.0097	1	U	F.AVEGPKL.V
292	568 - 574	357.2132	712.4118	712.4119	-0.11	0	21	0.0087	1	U	F.AVEGPKL.V
2681	568 - 582	756.9345	1511.8545	1511.8559	-0.95	1	16	0.023	1	U	F.AVEGPKLVVSTQTAL.A
2682	568 - 582	756.9348	1511.8551	1511.8559	-0.56	1	25	0.0032	1	U	F.AVEGPKLVVSTQTAL.A
2683	568 - 582	756.9350	1511.8554	1511.8559	-0.31	1	33	0.00054	1	U	F.AVEGPKLVVSTQTAL.A
2953	568 - 583	792.4526	1582.8906	1582.8930	-1.52	2	51	8.7e-006	1	U	F.AVEGPKLVVSTQTALA.-
2954	568 - 583	792.4529	1582.8912	1582.8930	-1.15	2	34	0.0004	1	U	F.AVEGPKLVVSTQTALA.-
2955	568 - 583	792.4531	1582.8917	1582.8930	-0.83	2	46	2.3e-005	1	U	F.AVEGPKLVVSTQTALA.-
2956	568 - 583	792.4531	1582.8917	1582.8930	-0.83	2	39	0.00012	1	U	F.AVEGPKLVVSTQTALA.-
2957	568 - 583	792.4531	1582.8917	1582.8930	-0.83	2	45	3e-005	1	U	F.AVEGPKLVVSTQTALA.-
2958	568 - 583	792.4533	1582.8919	1582.8930	-0.68	2	47	2e-005	1	U	F.AVEGPKLVVSTQTALA.-
2959	568 - 583	792.4533	1582.8921	1582.8930	-0.60	2	44	3.6e-005	1	U	F.AVEGPKLVVSTQTALA.-
2960	568 - 583	792.4533	1582.8921	1582.8930	-0.60	2	40	0.0001	1	U	F.AVEGPKLVVSTQTALA.-
2961	568 - 583	792.4534	1582.8922	1582.8930	-0.53	2	34	0.0004	1	U	F.AVEGPKLVVSTQTALA.-
2962	568 - 583	792.4534	1582.8922	1582.8930	-0.53	2	47	2e-005	1	U	F.AVEGPKLVVSTQTALA.-
2963	568 - 583	792.4534	1582.8923	1582.8930	-0.45	2	33	0.00045	1	U	F.AVEGPKLVVSTQTALA.-
2964	568 - 583	792.4535	1582.8924	1582.8930	-0.37	2	36	0.00025	1	U	F.AVEGPKLVVSTQTALA.-
2965	568 - 583	792.4535	1582.8924	1582.8930	-0.37	2	32	0.00065	1	U	F.AVEGPKLVVSTQTALA.-
2966	568 - 583	792.4535	1582.8924	1582.8930	-0.37	2	35	0.00031	1	U	F.AVEGPKLVVSTQTALA.-
2967	568 - 583	792.4535	1582.8925	1582.8930	-0.30	2	47	2e-005	1	U	F.AVEGPKLVVSTQTALA.-
2968	568 - 583	792.4536	1582.8927	1582.8930	-0.22	2	45	3.4e-005	1	U	F.AVEGPKLVVSTQTALA.-
3346	568 - 583	873.4786	1744.9427	1744.9458	-1.79	2	19	0.012	1	U	F.AVEGPKLVVSTQTALA.- + Glycation (K)
3347	568 - 583	873.4791	1744.9437	1744.9458	-1.23	2	24	0.004	1	U	F.AVEGPKLVVSTQTALA.- + Glycation (K)
3349	568 - 583	873.4798	1744.9450	1744.9458	-0.46	2	32	0.00062	1	U	F.AVEGPKLVVSTQTALA.- + Glycation (K)

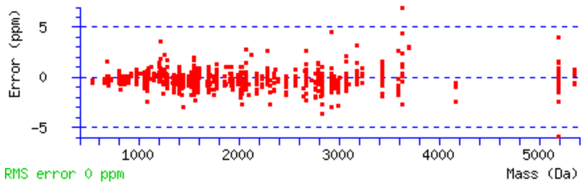

Mascot: http://www.matrixscience.com/

Appendix 3. Mascot search results of trypsin digestion for BSA-2

MATRIX SCIENCE **MASCOT Search Results**

Protein View: >gi|00000

BSA

**Database:**	BSA
**Score:**	29919
**Monoisotopic mass (M_r):**	68386
**Calculated pI:**	5.60

Sequence similarity is available as an NCBI BLAST search of >gi|00000 against nr.

Search parameters

**MS data file:**	P2019021809.mgf
**Enzyme:**	Trypsin/P: cuts C-term side of KR.
**Fixed modifications:**	Carbamidomethyl (C)
**Variable modifications:**	Glycation (K), Glycation (Protein N-term)

Protein sequence coverage: 92%

Matched peptides shown in ***bold red***.

```
  1 DTHKSEIAHR FKDLGEEHFK GLVLIAFSQY LQQCPFDEHV KLVNELTEFA
 51 KTCVADESHA GCEKSLHTLF GDELCKVASL RETYGDMADC CEKQEPERNE
101 CFLSHKDDSP DLPKLKPDPN TLCDEFKADE KKFWGKYLYE IARRHPYFYA
151 PELLYYANKY NGVFQECCQA EDKGACLLPK IETMREKVLA SSARQRLRCA
201 SIQKFGERAL KAWSVARLSQ KFPKAEFVEV TKLVTDLTKV HKECCHGDLL
251 ECADDRADLA KYICDNQDTI SSKLKECCDK PLLEKSHCIA EVEKDAIPEN
301 LPPLTADFAE DKDVCKNYQE AKDAFLGSFL YEYSRRHPEY AVSVLLRLAK
351 EYEATLEECC AKDDPHACYS TVFDKLKHLV DEPQNLIKQN CDQFEKLGEY
401 GFQNALIVRY TRKVPQVSTP TLVEVSRSLG KVGTRCCTKP ESERMPCTED
451 YLSLILNRLC VLHEKTPVSE KVTKCCTESL VNRRPCFSAL TPDETYVPKA
501 FDEKLFTFHA DICTLPDTEK QIKKQTALVE LLKHKPKATE EQLKTVMENF
551 VAFVDKCCAA DDKEACFAVE GPKLVVSTQT ALA
```

Unformatted sequence string: 583 residues (for pasting into other applications).

Sort by ◉ residue number ○ increasing mass ○ decreasing mass
Show ◉ matched peptides only ○ predicted peptides also

Query	Start – End	Observed	Mr(expt)	Mr(calc)	ppm	M	Score	Expect	Rank	U	Peptide
1047	11 – 20	417.2116	1248.6130	1248.6139	-0.70	1	26	0.0023	1	U	R.FKDLGEEHFK.G
1048	11 – 20	313.1606	1248.6133	1248.6139	-0.43	1	19	0.014	1	U	R.FKDLGEEHFK.G
1049	11 – 20	417.2118	1248.6135	1248.6139	-0.34	1	31	0.00071	1	U	R.FKDLGEEHFK.G
1050	11 – 20	625.3141	1248.6136	1248.6139	-0.20	1	49	1.2e-005	1	U	R.FKDLGEEHFK.G
1051	11 – 20	417.2119	1248.6137	1248.6139	-0.12	1	43	4.6e-005	1	U	R.FKDLGEEHFK.G
1052	11 – 20	313.1609	1248.6143	1248.6139	0.37	1	20	0.0089	1	U	R.FKDLGEEHFK.G
1053	11 – 20	625.3144	1248.6143	1248.6139	0.38	1	49	1.2e-005	1	U	R.FKDLGEEHFK.G
1054	11 – 20	417.2124	1248.6154	1248.6139	1.20	1	47	2.1e-005	1	U	R.FKDLGEEHFK.G
1371	11 – 20	353.6738	1410.6662	1410.6667	-0.34	1	46	2.7e-005	1	U	R.FKDLGEEHFK.G + Glycation (K)
1372	11 – 20	353.6739	1410.6665	1410.6667	-0.17	1	50	1.1e-005	1	U	R.FKDLGEEHFK.G + Glycation (K)
1373	11 – 20	706.3405	1410.6665	1410.6667	-0.15	1	44	4.4e-005	1	U	R.FKDLGEEHFK.G + Glycation (K)
1374	11 – 20	471.2295	1410.6666	1410.6667	-0.11	1	32	0.00069	1	U	R.FKDLGEEHFK.G + Glycation (K)
1375	11 – 20	706.3406	1410.6666	1410.6667	-0.067	1	47	2e-005	1	U	R.FKDLGEEHFK.G + Glycation (K)
1376	11 – 20	471.2295	1410.6666	1410.6667	-0.043	1	47	2.1e-005	1	U	R.FKDLGEEHFK.G + Glycation (K)
1377	11 – 20	471.2296	1410.6668	1410.6667	0.085	1	47	2.1e-005	1	U	R.FKDLGEEHFK.G + Glycation (K)
1378	11 – 20	706.3408	1410.6670	1410.6667	0.19	1	33	0.00055	1	U	R.FKDLGEEHFK.G + Glycation (K)
1379	11 – 20	353.6740	1410.6671	1410.6667	0.25	1	45	2.9e-005	1	U	R.FKDLGEEHFK.G + Glycation (K)
1380	11 – 20	706.3409	1410.6673	1410.6667	0.44	1	33	0.00055	1	U	R.FKDLGEEHFK.G + Glycation (K)
1381	11 – 20	353.6741	1410.6674	1410.6667	0.51	1	42	6.4e-005	1	U	R.FKDLGEEHFK.G + Glycation (K)
1382	11 – 20	471.2299	1410.6680	1410.6667	0.94	1	32	0.00064	1	U	R.FKDLGEEHFK.G + Glycation (K)
1383	11 – 20	471.2301	1410.6684	1410.6667	1.19	1	35	0.0003	1	U	R.FKDLGEEHFK.G + Glycation (K)
489	13 – 20	325.4906	973.4501	973.4505	-0.45	0	29	0.0013	1	U	K.DLGEEHFK.G
490	13 – 20	325.4906	973.4501	973.4505	-0.45	0	23	0.0047	1	U	K.DLGEEHFK.G
491	13 – 20	325.4907	973.4502	973.4505	-0.36	0	26	0.0028	1	U	K.DLGEEHFK.G
492	13 – 20	487.7324	973.4502	973.4505	-0.34	0	14	0.037	1	U	K.DLGEEHFK.G
493	13 – 20	487.7324	973.4503	973.4505	-0.22	0	24	0.0043	1	U	K.DLGEEHFK.G
494	13 – 20	487.7325	973.4505	973.4505	-0.033	0	22	0.0068	1	U	K.DLGEEHFK.G
495	13 – 20	325.4908	973.4505	973.4505	0.012	0	23	0.005	1	U	K.DLGEEHFK.G
496	13 – 20	487.7325	973.4505	973.4505	0.029	0	15	0.031	1	U	K.DLGEEHFK.G
497	13 – 20	487.7327	973.4509	973.4505	0.42	0	22	0.0069	1	U	K.DLGEEHFK.G
3441	21 – 41	831.4236	2491.2489	2491.2570	-3.24	0	37	0.00018	1	U	K.GLVLIAFSQYLQQCPFDEHVK.L
926	42 – 51	582.3188	1162.6230	1162.6234	-0.29	0	61	7.3e-007	1	U	K.LVNELTEFAK.T
927	42 – 51	582.3189	1162.6233	1162.6234	-0.085	0	61	7.4e-007	1	U	K.LVNELTEFAK.T
928	42 – 51	582.3190	1162.6234	1162.6234	0.018	0	61	7.1e-007	1	U	K.LVNELTEFAK.T
929	42 – 51	582.3190	1162.6235	1162.6234	0.12	0	58	1.7e-006	1	U	K.LVNELTEFAK.T
930	42 – 51	582.3192	1162.6237	1162.6234	0.33	0	62	6.9e-007	1	U	K.LVNELTEFAK.T
3616	42 – 64	693.3186	2769.2453	2769.2473	-0.73	1	92	6.2e-010	1	U	K.LVNELTEFAKTCVADESHAGCEK.S + Glycatio
3617	42 – 64	693.3189	2769.2465	2769.2473	-0.28	1	88	1.7e-009	1	U	K.LVNELTEFAKTCVADESHAGCEK.S + Glycatio
4167	42 – 76	1084.0026	4331.9811	4331.9760	1.19	2	44	3.9e-005	1	U	K.LVNELTEFAKTCVADESHAGCEKSLHTLFGDELCK
1539	52 – 64	732.2973	1462.5800	1462.5817	-1.12	0	91	8.2e-010	1	U	K.TCVADESHAGCEK.S
1540	52 – 64	732.2974	1462.5803	1462.5817	-0.95	0	91	8.4e-010	1	U	K.TCVADESHAGCEK.S
1541	52 – 64	488.5344	1462.5813	1462.5817	-0.29	0	67	2.2e-007	1	U	K.TCVADESHAGCEK.S
1542	52 – 64	488.5345	1462.5817	1462.5817	0.036	0	64	3.7e-007	1	U	K.TCVADESHAGCEK.S

Query	Start – End	Observed	Mr(expt)	Mr(calc)	ppm	M	Score	Expect	Rank	U	Peptide
1543	52 - 64	488.5347	1462.5823	1462.5817	0.40	0	76	2.4e-008	1	U	K.TCVADESHAGCEK.S
3746	52 - 76	606.0679	3025.3033	3025.3103	-2.34	1	50	1e-005	1	U	K.TCVADESHAGCEKSLHTLFGDELCK.V + Glycat
3747	52 - 76	757.3331	3025.3034	3025.3103	-2.29	1	64	4.3e-007	1	U	K.TCVADESHAGCEKSLHTLFGDELCK.V + Glycat
3748	52 - 76	1009.4431	3025.3073	3025.3103	-1.00	1	75	3.2e-008	1	U	K.TCVADESHAGCEKSLHTLFGDELCK.V + Glycat
3749	52 - 76	1009.4432	3025.3077	3025.3103	-0.87	1	74	3.8e-008	1	U	K.TCVADESHAGCEKSLHTLFGDELCK.V + Glycat
3750	52 - 76	757.3343	3025.3081	3025.3103	-0.76	1	56	2.4e-006	1	U	K.TCVADESHAGCEKSLHTLFGDELCK.V + Glycat
3751	52 - 76	757.3344	3025.3085	3025.3103	-0.60	1	56	2.5e-006	1	U	K.TCVADESHAGCEKSLHTLFGDELCK.V + Glycat
3752	52 - 76	1009.4437	3025.3094	3025.3103	-0.33	1	76	2.3e-008	1	U	K.TCVADESHAGCEKSLHTLFGDELCK.V + Glycat
3753	52 - 76	606.0692	3025.3094	3025.3103	-0.32	1	74	4.2e-008	1	U	K.TCVADESHAGCEKSLHTLFGDELCK.V + Glycat
3754	52 - 76	606.0693	3025.3100	3025.3103	-0.13	1	63	4.7e-007	1	U	K.TCVADESHAGCEKSLHTLFGDELCK.V + Glycat
3755	52 - 76	757.3348	3025.3100	3025.3103	-0.11	1	59	1.2e-006	1	U	K.TCVADESHAGCEKSLHTLFGDELCK.V + Glycat
4013	52 - 81	743.7430	3713.6785	3713.6859	-1.99	2	50	1.1e-005	1	U	K.TCVADESHAGCEKSLHTLFGDELCKVASLR.E +
4014	52 - 81	929.4269	3713.6787	3713.6859	-1.95	2	30	0.001	1	U	K.TCVADESHAGCEKSLHTLFGDELCKVASLR.E +
4015	52 - 81	743.7435	3713.6810	3713.6859	-1.33	2	14	0.037	1	U	K.TCVADESHAGCEKSLHTLFGDELCKVASLR.E +
4016	52 - 81	743.7437	3713.6822	3713.6859	-1.00	2	44	4.4e-005	1	U	K.TCVADESHAGCEKSLHTLFGDELCKVASLR.E +
4017	52 - 81	929.4286	3713.6855	3713.6859	-0.11	2	41	7.2e-005	1	U	K.TCVADESHAGCEKSLHTLFGDELCKVASLR.E +
4018	52 - 81	929.4288	3713.6860	3713.6859	0.021	2	41	7.3e-005	1	U	K.TCVADESHAGCEKSLHTLFGDELCKVASLR.E +
4019	52 - 81	619.9553	3713.6883	3713.6859	0.64	2	32	0.00066	1	U	K.TCVADESHAGCEKSLHTLFGDELCKVASLR.E +
4021	52 - 81	1238.9111	3713.7116	3713.6859	6.91	2	23	0.005	1	U	K.TCVADESHAGCEKSLHTLFGDELCKVASLR.E +
1416	65 - 76	710.3499	1418.6853	1418.6864	-0.80	0	62	5.7e-007	1	U	K.SLHTLFGDELCK.V
1417	65 - 76	710.3500	1418.6855	1418.6864	-0.62	0	64	4.3e-007	1	U	K.SLHTLFGDELCK.V
1418	65 - 76	710.3500	1418.6855	1418.6864	-0.62	0	64	3.7e-007	1	U	K.SLHTLFGDELCK.V
1419	65 - 76	473.9026	1418.6861	1418.6864	-0.20	0	43	4.5e-005	1	U	K.SLHTLFGDELCK.V
1420	65 - 76	473.9027	1418.6862	1418.6864	-0.13	0	25	0.0032	1	U	K.SLHTLFGDELCK.V
1421	65 - 76	473.9027	1418.6864	1418.6864	-0.0063	0	51	8e-006	1	U	K.SLHTLFGDELCK.V
1422	65 - 76	473.9028	1418.6865	1418.6864	0.057	0	57	1.9e-006	1	U	K.SLHTLFGDELCK.V
1423	65 - 76	473.9032	1418.6878	1418.6864	0.97	0	49	1.3e-005	1	U	K.SLHTLFGDELCK.V
3079	65 - 81	703.3605	2107.0598	2107.0620	-1.04	1	63	5.5e-007	1	U	K.SLHTLFGDELCKVASLR.E + Glycation (K)
3080	65 - 81	703.3609	2107.0609	2107.0620	-0.51	1	56	2.8e-006	1	U	K.SLHTLFGDELCKVASLR.E + Glycation (K)
3081	65 - 81	703.3609	2107.0609	2107.0620	-0.51	1	58	1.7e-006	1	U	K.SLHTLFGDELCKVASLR.E + Glycation (K)
3082	65 - 81	1054.5377	2107.0609	2107.0620	-0.50	1	30	0.001	1	U	K.SLHTLFGDELCKVASLR.E + Glycation (K)
3083	65 - 81	1054.5381	2107.0616	2107.0620	-0.15	1	37	0.0002	1	U	K.SLHTLFGDELCKVASLR.E + Glycation (K)
3084	65 - 81	527.7728	2107.0620	2107.0620	0.012	1	38	0.00018	1	U	K.SLHTLFGDELCKVASLR.E + Glycation (K)
3085	65 - 81	527.7729	2107.0625	2107.0620	0.24	1	41	8.8e-005	1	U	K.SLHTLFGDELCKVASLR.E + Glycation (K)
3086	65 - 81	527.7730	2107.0629	2107.0620	0.47	1	41	7.7e-005	1	U	K.SLHTLFGDELCKVASLR.E + Glycation (K)
3087	65 - 81	703.3616	2107.0631	2107.0620	0.53	1	17	0.019	1	U	K.SLHTLFGDELCKVASLR.E + Glycation (K)
1609	82 - 93	739.7646	1477.5146	1477.5160	-0.90	0	79	1.2e-008	1	U	R.ETYGDMADCCEK.Q
1610	82 - 93	739.7646	1477.5147	1477.5160	-0.82	0	66	2.5e-007	1	U	R.ETYGDMADCCEK.Q
1611	82 - 93	739.7646	1477.5147	1477.5160	-0.82	0	79	1.2e-008	1	U	R.ETYGDMADCCEK.Q
1612	82 - 93	739.7651	1477.5156	1477.5160	-0.24	0	66	2.5e-007	1	U	R.ETYGDMADCCEK.Q
3225	82 - 98	760.6285	2278.8638	2278.8664	-1.15	1	58	1.6e-006	1	U	R.ETYGDMADCCEKQEPER.N + Glycation (K)
3226	82 - 98	760.6290	2278.8651	2278.8664	-0.58	1	46	2.3e-005	1	U	R.ETYGDMADCCEKQEPER.N + Glycation (K)
3227	82 - 98	760.6290	2278.8651	2278.8664	-0.58	1	45	3.1e-005	1	U	R.ETYGDMADCCEKQEPER.N + Glycation (K)
628	99 - 106	517.7393	1033.4641	1033.4651	-0.98	0	34	0.00042	1	U	R.NECFLSHK.D
629	99 - 106	345.4954	1033.4643	1033.4651	-0.82	0	14	0.041	1	U	R.NECFLSHK.D
630	99 - 106	345.4955	1033.4648	1033.4651	-0.30	0	26	0.0026	1	U	R.NECFLSHK.D
631	99 - 106	345.4955	1033.4648	1033.4651	-0.30	0	30	0.0011	1	U	R.NECFLSHK.D
632	99 - 106	345.4955	1033.4648	1033.4651	-0.30	0	34	0.00038	1	U	R.NECFLSHK.D
633	99 - 106	517.7397	1033.4649	1033.4651	-0.15	0	34	0.00038	1	U	R.NECFLSHK.D
634	99 - 106	345.4956	1033.4650	1033.4651	-0.098	0	25	0.0028	1	U	R.NECFLSHK.D
635	99 - 106	517.7398	1033.4651	1033.4651	-0.034	0	34	0.00039	1	U	R.NECFLSHK.D
636	99 - 106	517.7398	1033.4651	1033.4651	-0.034	0	34	0.0004	1	U	R.NECFLSHK.D
637	99 - 106	345.4957	1033.4652	1033.4651	0.076	0	26	0.0025	1	U	R.NECFLSHK.D
638	99 - 106	345.4957	1033.4652	1033.4651	0.076	0	27	0.0022	1	U	R.NECFLSHK.D
639	99 - 106	345.4957	1033.4653	1033.4651	0.16	0	26	0.0026	1	U	R.NECFLSHK.D
640	99 - 106	517.7399	1033.4653	1033.4651	0.20	0	34	0.0004	1	U	R.NECFLSHK.D
641	99 - 106	517.7400	1033.4654	1033.4651	0.31	0	34	0.0004	1	U	R.NECFLSHK.D
642	99 - 106	345.4958	1033.4655	1033.4651	0.42	0	26	0.0024	1	U	R.NECFLSHK.D
643	99 - 106	517.7401	1033.4655	1033.4651	0.43	0	25	0.0034	1	U	R.NECFLSHK.D
644	99 - 106	517.7405	1033.4665	1033.4651	1.38	0	23	0.0046	1	U	R.NECFLSHK.D
645	99 - 106	517.7407	1033.4669	1033.4651	1.73	0	23	0.0046	1	U	R.NECFLSHK.D
2730	99 - 114	951.4366	1900.8587	1900.8625	-1.98	1	69	1.2e-007	1	U	R.NECFLSHKDDSPDLPK.L
2731	99 - 114	634.6274	1900.8605	1900.8625	-1.07	1	47	1.9e-005	1	U	R.NECFLSHKDDSPDLPK.L
2732	99 - 114	634.6277	1900.8614	1900.8625	-0.58	1	50	1e-005	1	U	R.NECFLSHKDDSPDLPK.L
2733	99 - 114	634.6278	1900.8616	1900.8625	-0.48	1	36	0.00023	1	U	R.NECFLSHKDDSPDLPK.L
2734	99 - 114	476.2228	1900.8619	1900.8625	-0.33	1	46	2.2e-005	1	U	R.NECFLSHKDDSPDLPK.L
2735	99 - 114	634.6279	1900.8620	1900.8625	-0.29	1	58	1.6e-006	1	U	R.NECFLSHKDDSPDLPK.L
2736	99 - 114	634.6279	1900.8620	1900.8625	-0.29	1	47	2e-005	1	U	R.NECFLSHKDDSPDLPK.L
2737	99 - 114	476.2228	1900.8621	1900.8625	-0.20	1	41	8.8e-005	1	U	R.NECFLSHKDDSPDLPK.L
2738	99 - 114	634.6280	1900.8621	1900.8625	-0.20	1	44	3.6e-005	1	U	R.NECFLSHKDDSPDLPK.L
2739	99 - 114	476.2228	1900.8623	1900.8625	-0.14	1	46	2.5e-005	1	U	R.NECFLSHKDDSPDLPK.L
2740	99 - 114	476.2229	1900.8626	1900.8625	0.052	1	34	0.00043	1	U	R.NECFLSHKDDSPDLPK.L
2741	99 - 114	476.2230	1900.8627	1900.8625	0.11	1	42	6.9e-005	1	U	R.NECFLSHKDDSPDLPK.L
3039	99 - 114	688.6451	2062.9134	2062.9153	-0.94	1	33	0.00051	1	U	R.NECFLSHKDDSPDLPK.L + Glycation (K)
3040	99 - 114	516.7358	2062.9143	2062.9153	-0.53	1	27	0.0018	1	U	R.NECFLSHKDDSPDLPK.L + Glycation (K)
3041	99 - 114	688.6454	2062.9143	2062.9153	-0.49	1	32	0.0007	1	U	R.NECFLSHKDDSPDLPK.L + Glycation (K)
3042	99 - 114	516.7361	2062.9152	2062.9153	-0.062	1	27	0.0018	1	U	R.NECFLSHKDDSPDLPK.L + Glycation (K)
3043	99 - 114	688.6458	2062.9156	2062.9153	0.13	1	34	0.00042	1	U	R.NECFLSHKDDSPDLPK.L + Glycation (K)
3044	99 - 114	516.7364	2062.9165	2062.9153	0.54	1	28	0.0017	1	U	R.NECFLSHKDDSPDLPK.L + Glycation (K)
335	107 - 114	443.7111	885.4077	885.4080	-0.32	0	41	8.4e-005	1	U	K.DDSPDLPK.L
336	107 - 114	443.7111	885.4077	885.4080	-0.26	0	30	0.0011	1	U	K.DDSPDLPK.L
337	107 - 114	443.7112	885.4079	885.4080	-0.12	0	43	5.2e-005	1	U	K.DDSPDLPK.L
338	107 - 114	443.7112	885.4079	885.4080	-0.12	0	38	0.00014	1	U	K.DDSPDLPK.L
339	107 - 114	443.7113	885.4081	885.4080	0.15	0	45	2.9e-005	1	U	K.DDSPDLPK.L
340	107 - 114	443.7114	885.4082	885.4080	0.31	0	42	5.7e-005	1	U	K.DDSPDLPK.L
341	107 - 114	443.7119	885.4091	885.4080	1.33	0	42	5.9e-005	1	U	K.DDSPDLPK.L
3505	107 - 127	869.4099	2605.2079	2605.2105	-1.01	2	95	3.4e-010	1	U	K.DDSPDLPKLKPDPNTLCDEFK.A + Glycation
3506	107 - 127	869.4100	2605.2081	2605.2105	-0.94	2	76	2.3e-008	1	U	K.DDSPDLPKLKPDPNTLCDEFK.A + Glycation

Query	Start - End	Observed	Mr(expt)	Mr(calc)	ppm	M	Score	Expect	Rank	U	Peptide
3507	107 - 127	869.4100	2605.2081	2605.2105	-0.94	2	72	7e-008	1	U	K.DDSPDLPKLKPDPNTLCDEFK.A + Glycation (
3508	107 - 127	652.3097	2605.2097	2605.2105	-0.33	2	88	1.6e-009	1	U	K.DDSPDLPKLKPDPNTLCDEFK.A + Glycation (
3509	107 - 127	652.3098	2605.2101	2605.2105	-0.15	2	88	1.7e-009	1	U	K.DDSPDLPKLKPDPNTLCDEFK.A + Glycation (
3510	107 - 127	652.3098	2605.2101	2605.2105	-0.15	2	93	5.2e-010	1	U	K.DDSPDLPKLKPDPNTLCDEFK.A + Glycation (
3511	107 - 127	1303.6146	2605.2147	2605.2105	1.60	2	89	1.3e-009	1	U	K.DDSPDLPKLKPDPNTLCDEFK.A + Glycation (
3611	107 - 127	923.4275	2767.2606	2767.2634	-0.98	2	53	5.5e-006	1	U	K.DDSPDLPKLKPDPNTLCDEFK.A + 2 Glycatio
3612	107 - 127	692.8230	2767.2629	2767.2634	-0.16	2	61	8.3e-007	1	U	K.DDSPDLPKLKPDPNTLCDEFK.A + 2 Glycatio
3613	107 - 127	923.4283	2767.2630	2767.2634	-0.12	2	56	2.4e-006	1	U	K.DDSPDLPKLKPDPNTLCDEFK.A + 2 Glycatio
3614	107 - 127	692.8234	2767.2646	2767.2634	0.46	2	60	1e-006	1	U	K.DDSPDLPKLKPDPNTLCDEFK.A + 2 Glycatio
3615	107 - 127	923.4291	2767.2656	2767.2634	0.81	2	56	2.4e-006	1	U	K.DDSPDLPKLKPDPNTLCDEFK.A + 2 Glycatio
1848	115 - 127	788.8864	1575.7583	1575.7603	-1.28	1	65	3e-007	1	U	K.LKPDPNTLCDEFK.A
1849	115 - 127	788.8865	1575.7585	1575.7603	-1.11	1	65	3.1e-007	1	U	K.LKPDPNTLCDEFK.A
1850	115 - 127	788.8866	1575.7586	1575.7603	-1.04	1	83	5.5e-009	1	U	K.LKPDPNTLCDEFK.A
1851	115 - 127	788.8868	1575.7590	1575.7603	-0.81	1	83	5.5e-009	1	U	K.LKPDPNTLCDEFK.A
1852	115 - 127	788.8869	1575.7592	1575.7603	-0.66	1	83	5.5e-009	1	U	K.LKPDPNTLCDEFK.A
1853	115 - 127	788.8870	1575.7594	1575.7603	-0.58	1	82	6.1e-009	1	U	K.LKPDPNTLCDEFK.A
1854	115 - 127	788.8870	1575.7595	1575.7603	-0.51	1	83	5.4e-009	1	U	K.LKPDPNTLCDEFK.A
1855	115 - 127	526.2606	1575.7600	1575.7603	-0.16	1	58	1.5e-006	1	U	K.LKPDPNTLCDEFK.A
1856	115 - 127	526.2607	1575.7602	1575.7603	-0.046	1	47	2e-005	1	U	K.LKPDPNTLCDEFK.A
1857	115 - 127	526.2608	1575.7606	1575.7603	0.18	1	49	1.3e-005	1	U	K.LKPDPNTLCDEFK.A
1858	115 - 127	526.2608	1575.7606	1575.7603	0.18	1	57	2.1e-006	1	U	K.LKPDPNTLCDEFK.A
1859	115 - 127	526.2608	1575.7606	1575.7603	0.18	1	66	2.8e-007	1	U	K.LKPDPNTLCDEFK.A
1860	115 - 127	526.2609	1575.7610	1575.7603	0.43	1	59	1.3e-006	1	U	K.LKPDPNTLCDEFK.A
1861	115 - 127	526.2610	1575.7611	1575.7603	0.54	1	54	3.7e-006	1	U	K.LKPDPNTLCDEFK.A
1862	115 - 127	526.2610	1575.7613	1575.7603	0.66	1	40	9.5e-005	1	U	K.LKPDPNTLCDEFK.A
2270	115 - 127	869.9128	1737.8110	1737.8131	-1.21	1	46	2.5e-005	1	U	K.LKPDPNTLCDEFK.A + Glycation (K)
2271	115 - 127	869.9128	1737.8110	1737.8131	-1.21	1	44	4e-005	1	U	K.LKPDPNTLCDEFK.A + Glycation (K)
2272	115 - 127	869.9129	1737.8112	1737.8131	-1.07	1	45	3.2e-005	1	U	K.LKPDPNTLCDEFK.A + Glycation (K)
2273	115 - 127	869.9131	1737.8117	1737.8131	-0.78	1	45	3.2e-005	1	U	K.LKPDPNTLCDEFK.A + Glycation (K)
2274	115 - 127	869.9134	1737.8122	1737.8131	-0.51	1	39	0.00012	1	U	K.LKPDPNTLCDEFK.A + Glycation (K)
2275	115 - 127	580.2781	1737.8126	1737.8131	-0.30	1	44	4e-005	1	U	K.LKPDPNTLCDEFK.A + Glycation (K)
2276	115 - 127	580.2782	1737.8128	1737.8131	-0.19	1	40	9.9e-005	1	U	K.LKPDPNTLCDEFK.A + Glycation (K)
2277	115 - 127	580.2782	1737.8128	1737.8131	-0.19	1	43	4.7e-005	1	U	K.LKPDPNTLCDEFK.A + Glycation (K)
2278	115 - 127	580.2783	1737.8130	1737.8131	-0.091	1	41	7.7e-005	1	U	K.LKPDPNTLCDEFK.A + Glycation (K)
2279	115 - 127	580.2784	1737.8135	1737.8131	0.22	1	37	0.00021	1	U	K.LKPDPNTLCDEFK.A + Glycation (K)
2280	115 - 127	580.2787	1737.8142	1737.8131	0.65	1	42	6.1e-005	1	U	K.LKPDPNTLCDEFK.A + Glycation (K)
3141	115 - 131	728.0110	2181.0111	2181.0147	-1.64	2	30	0.001	1	U	K.LKPDPNTLCDEFKADEK.K + Glycation (K)
3142	115 - 131	728.0116	2181.0130	2181.0147	-0.80	2	63	5.2e-007	1	U	K.LKPDPNTLCDEFKADEK.K + Glycation (K)
3144	115 - 131	728.0119	2181.0139	2181.0147	-0.38	2	66	2.6e-007	1	U	K.LKPDPNTLCDEFKADEK.K + Glycation (K)
3145	115 - 131	728.0123	2181.0152	2181.0147	0.21	2	50	9.8e-006	1	U	K.LKPDPNTLCDEFKADEK.K + Glycation (K)
3146	115 - 131	728.0124	2181.0153	2181.0147	0.29	2	26	0.0027	1	U	K.LKPDPNTLCDEFKADEK.K + Glycation (K)
3309	115 - 131	782.0294	2343.0663	2343.0675	-0.55	2	35	0.0003	1	U	K.LKPDPNTLCDEFKADEK.K + 2 Glycation (K)
3428	115 - 132	618.7971	2471.1594	2471.1625	-1.26	3	13	0.05	1	U	K.LKPDPNTLCDEFKADEKK.F + 2 Glycation (K
3429	115 - 132	618.7974	2471.1606	2471.1625	-0.77	3	15	0.032	1	U	K.LKPDPNTLCDEFKADEKK.F + 2 Glycation (K
3449	128 - 143	626.5582	2502.2038	2502.2053	-0.60	3	37	0.00018	1	U	K.ADEKKFWGKYLYEIAR.R + 3 Glycation (K)
3450	128 - 143	626.5585	2502.2050	2502.2053	-0.12	3	44	4.4e-005	1	U	K.ADEKKFWGKYLYEIAR.R + 3 Glycation (K)
3451	128 - 143	626.5585	2502.2050	2502.2053	-0.12	3	44	3.9e-005	1	U	K.ADEKKFWGKYLYEIAR.R + 3 Glycation (K)
2260	132 - 143	868.4554	1734.8963	1734.8981	-1.00	2	57	1.9e-006	1	U	K.KFWGKYLYEIAR.R + Glycation (K)
2261	132 - 143	868.4558	1734.8971	1734.8981	-0.58	2	57	1.9e-006	1	U	K.KFWGKYLYEIAR.R + Glycation (K)
2262	132 - 143	579.3064	1734.8974	1734.8981	-0.40	2	46	2.5e-005	1	U	K.KFWGKYLYEIAR.R + Glycation (K)
2263	132 - 143	434.7316	1734.8974	1734.8981	-0.38	2	20	0.0094	1	U	K.KFWGKYLYEIAR.R + Glycation (K)
2264	132 - 143	579.3065	1734.8976	1734.8981	-0.30	2	61	8.7e-007	1	U	K.KFWGKYLYEIAR.R + Glycation (K)
2265	132 - 143	434.7317	1734.8979	1734.8981	-0.10	2	23	0.0049	1	U	K.KFWGKYLYEIAR.R + Glycation (K)
2266	132 - 143	579.3066	1734.8979	1734.8981	-0.092	2	49	1.3e-005	1	U	K.KFWGKYLYEIAR.R + Glycation (K)
2267	132 - 143	434.7318	1734.8980	1734.8981	-0.032	2	23	0.0055	1	U	K.KFWGKYLYEIAR.R + Glycation (K)
2268	132 - 143	579.3067	1734.8983	1734.8981	0.12	2	73	5.5e-008	1	U	K.KFWGKYLYEIAR.R + Glycation (K)
2269	132 - 143	579.3070	1734.8990	1734.8981	0.55	2	61	8.6e-007	1	U	K.KFWGKYLYEIAR.R + Glycation (K)
2726	132 - 143	949.4803	1896.9460	1896.9509	-2.57	2	47	2e-005	1	U	K.KFWGKYLYEIAR.R + 2 Glycation (K)
2727	132 - 143	633.3239	1896.9499	1896.9509	-0.52	2	36	0.00025	1	U	K.KFWGKYLYEIAR.R + 2 Glycation (K)
2728	132 - 143	633.3241	1896.9505	1896.9509	-0.22	2	45	3.2e-005	1	U	K.KFWGKYLYEIAR.R + 2 Glycation (K)
2729	132 - 143	633.3242	1896.9507	1896.9509	-0.13	2	43	5.2e-005	1	U	K.KFWGKYLYEIAR.R + 2 Glycation (K)
1902	133 - 143	804.4083	1606.8021	1606.8031	-0.63	1	46	2.3e-005	1	U	K.FWGKYLYEIAR.R + Glycation (K)
1903	133 - 143	804.4083	1606.8021	1606.8031	-0.63	1	47	2e-005	1	U	K.FWGKYLYEIAR.R + Glycation (K)
1904	133 - 143	804.4085	1606.8025	1606.8031	-0.40	1	47	2.2e-005	1	U	K.FWGKYLYEIAR.R + Glycation (K)
1905	133 - 143	536.6082	1606.8028	1606.8031	-0.18	1	27	0.0022	1	U	K.FWGKYLYEIAR.R + Glycation (K)
1906	133 - 143	536.6082	1606.8028	1606.8031	-0.18	1	40	0.00011	1	U	K.FWGKYLYEIAR.R + Glycation (K)
1907	133 - 143	536.6083	1606.8030	1606.8031	-0.065	1	37	0.00019	1	U	K.FWGKYLYEIAR.R + Glycation (K)
1908	133 - 143	536.6086	1606.8039	1606.8031	0.50	1	26	0.0024	1	U	K.FWGKYLYEIAR.R + Glycation (K)
1910	133 - 143	804.4094	1606.8042	1606.8031	0.65	1	49	1.2e-005	1	U	K.FWGKYLYEIAR.R + Glycation (K)
1918	133 - 143	536.6101	1606.8085	1606.8031	3.35	1	40	9.8e-005	1	U	K.FWGKYLYEIAR.R + Glycation (K)
405	137 - 143	464.2502	926.4859	926.4861	-0.28	0	31	0.00084	1	U	K.YLYEIAR.R
406	137 - 143	464.2504	926.4862	926.4861	0.13	0	28	0.0016	1	U	K.YLYEIAR.R
407	137 - 143	464.2504	926.4862	926.4861	0.13	0	28	0.0015	1	U	K.YLYEIAR.R
408	137 - 143	464.2504	926.4863	926.4861	0.19	0	27	0.002	1	U	K.YLYEIAR.R
409	137 - 143	464.2505	926.4864	926.4861	0.26	0	31	0.00082	1	U	K.YLYEIAR.R
410	137 - 143	464.2505	926.4864	926.4861	0.32	0	20	0.01	1	U	K.YLYEIAR.R
3028	144 - 159	1023.0170	2044.0194	2044.0206	-0.61	1	45	3e-005	1	U	R.RHPYFYAPELLYYANK.Y
3029	144 - 159	682.3471	2044.0195	2044.0206	-0.55	1	41	8.7e-005	1	U	R.RHPYFYAPELLYYANK.Y
3030	144 - 159	682.3472	2044.0199	2044.0206	-0.38	1	48	1.6e-005	1	U	R.RHPYFYAPELLYYANK.Y
3031	144 - 159	682.3472	2044.0199	2044.0206	-0.38	1	44	4.1e-005	1	U	R.RHPYFYAPELLYYANK.Y
3032	144 - 159	512.0125	2044.0209	2044.0206	0.15	1	42	6.6e-005	1	U	R.RHPYFYAPELLYYANK.Y
3033	144 - 159	512.0127	2044.0217	2044.0206	0.52	1	37	0.00021	1	U	R.RHPYFYAPELLYYANK.Y
3034	144 - 159	512.0128	2044.0219	2044.0206	0.64	1	37	0.00021	1	U	R.RHPYFYAPELLYYANK.Y
3035	144 - 159	682.3481	2044.0226	2044.0206	0.96	1	42	6.1e-005	1	U	R.RHPYFYAPELLYYANK.Y
4091	144 - 173	787.9587	3934.7573	3934.7607	-0.85	2	38	0.00014	1	U	R.RHPYFYAPELLYYANKYNGVFQECCQAEDK.G + (
4092	144 - 173	984.6972	3934.7598	3934.7607	-0.23	2	72	6e-008	1	U	R.RHPYFYAPELLYYANKYNGVFQECCQAEDK.G + (
4093	144 - 173	787.9594	3934.7607	3934.7607	0.0010	2	66	2.3e-007	1	U	R.RHPYFYAPELLYYANKYNGVFQECCQAEDK.G + (

Query	Start – End	Observed	Mr(expt)	Mr(calc)	ppm	M	Score	Expect	Rank	U	Peptide
4094	144 – 173	984.6979	3934.7624	3934.7607	0.44	2	66	2.8e-007	1	U	R.RHPYFYAPELLYYANKYNGVFQECCQAEDK.G + G
4095	144 – 173	984.6985	3934.7649	3934.7607	1.09	2	67	2e-007	1	U	R.RHPYFYAPELLYYANKYNGVFQECCQAEDK.G + G
4096	144 – 173	787.9612	3934.7698	3934.7607	2.33	2	54	4e-006	1	U	R.RHPYFYAPELLYYANKYNGVFQECCQAEDK.G + G
4097	144 – 173	1312.5974	3934.7704	3934.7607	2.47	2	64	3.6e-007	1	U	R.RHPYFYAPELLYYANKYNGVFQECCQAEDK.G + G
4285	144 – 180	968.2476	4836.2014	4836.2186	-3.55	3	27	0.0019	1	U	R.RHPYFYAPELLYYANKYNGVFQECCQAEDKGACLL
4286	144 – 180	968.2503	4836.2152	4836.2186	-0.70	3	38	0.00017	1	U	R.RHPYFYAPELLYYANKYNGVFQECCQAEDKGACLL
4287	144 – 180	1210.0623	4836.2203	4836.2186	0.35	3	43	5.4e-005	1	U	R.RHPYFYAPELLYYANKYNGVFQECCQAEDKGACLL
2685	145 – 159	944.9662	1887.9178	1887.9195	-0.90	0	43	4.9e-005	1	U	R.HPYFYAPELLYYANK.Y
2686	145 – 159	944.9662	1887.9179	1887.9195	-0.84	0	46	2.6e-005	1	U	R.HPYFYAPELLYYANK.Y
2687	145 – 159	944.9663	1887.9181	1887.9195	-0.77	0	63	4.9e-007	1	U	R.HPYFYAPELLYYANK.Y
2688	145 – 159	630.3137	1887.9192	1887.9195	-0.20	0	41	7.4e-005	1	U	R.HPYFYAPELLYYANK.Y
2689	145 – 159	630.3137	1887.9192	1887.9195	-0.20	0	51	8e-006	1	U	R.HPYFYAPELLYYANK.Y
2690	145 – 159	630.3137	1887.9193	1887.9195	-0.10	0	48	1.5e-005	1	U	R.HPYFYAPELLYYANK.Y
2691	145 – 159	630.3138	1887.9197	1887.9195	0.088	0	46	2.3e-005	1	U	R.HPYFYAPELLYYANK.Y
4049	145 – 173	945.6708	3778.6543	3778.6596	-1.40	1	83	5e-009	1	U	R.HPYFYAPELLYYANKYNGVFQECCQAEDK.G + Gl
2294	160 – 173	874.3552	1746.6958	1746.6978	-1.15	0	64	4.3e-007	1	U	K.YNGVFQECCQAEDK.G
2295	160 – 173	874.3554	1746.6961	1746.6978	-0.93	0	67	1.8e-007	1	U	K.YNGVFQECCQAEDK.G
2296	160 – 173	874.3555	1746.6964	1746.6978	-0.79	0	64	4.2e-007	1	U	K.YNGVFQECCQAEDK.G
2297	160 – 173	874.3556	1746.6967	1746.6978	-0.58	0	44	3.9e-005	1	U	K.YNGVFQECCQAEDK.G
3531	160 – 180	883.7249	2648.1526	2648.1557	-1.05	1	18	0.016	1	U	K.YNGVFQECCQAEDKGACLLPK.I + Glycation (
3532	160 – 180	883.7264	2648.1573	2648.1557	0.62	1	24	0.0042	1	U	K.YNGVFQECCQAEDKGACLLPK.I + Glycation (
155	174 – 180	379.7149	757.4152	757.4156	-0.59	0	19	0.012	1	U	K.GACLLPK.I
156	174 – 180	379.7150	757.4154	757.4156	-0.32	0	32	0.00062	1	U	K.GACLLPK.I
157	174 – 180	379.7150	757.4154	757.4156	-0.25	0	32	0.00062	1	U	K.GACLLPK.I
158	174 – 180	379.7150	757.4154	757.4156	-0.25	0	32	0.00062	1	U	K.GACLLPK.I
159	174 – 180	379.7150	757.4155	757.4156	-0.17	0	23	0.0055	1	U	K.GACLLPK.I
160	174 – 180	379.7150	757.4155	757.4156	-0.17	0	21	0.0088	1	U	K.GACLLPK.I
161	174 – 180	379.7151	757.4157	757.4156	0.071	0	32	0.00063	1	U	K.GACLLPK.I
162	174 – 180	379.7152	757.4159	757.4156	0.31	0	21	0.0079	1	U	K.GACLLPK.I
163	174 – 180	379.7152	757.4159	757.4156	0.39	0	32	0.00063	1	U	K.GACLLPK.I
1805	174 – 185	775.8990	1549.7835	1549.7844	-0.54	1	55	3e-006	1	U	K.GACLLPKIETMR.E + Glycation (K)
1806	174 – 185	775.8992	1549.7838	1549.7844	-0.38	1	50	9.2e-006	1	U	K.GACLLPKIETMR.E + Glycation (K)
1807	174 – 185	517.6022	1549.7847	1549.7844	0.20	1	32	0.00057	1	U	K.GACLLPKIETMR.E + Glycation (K)
1808	174 – 185	517.6022	1549.7849	1549.7844	0.31	1	36	0.00022	1	U	K.GACLLPKIETMR.E + Glycation (K)
1809	174 – 185	517.6023	1549.7850	1549.7844	0.43	1	28	0.0015	1	U	K.GACLLPKIETMR.E + Glycation (K)
1258	199 – 208	679.3238	1356.6331	1356.6344	-0.89	1	34	0.00039	1	U	R.CASIQKFGER.A + Glycation (K)
1259	199 – 208	679.3239	1356.6333	1356.6344	-0.80	1	36	0.00027	1	U	R.CASIQKFGER.A + Glycation (K)
1260	199 – 208	679.3240	1356.6334	1356.6344	-0.71	1	35	0.0003	1	U	R.CASIQKFGER.A + Glycation (K)
1261	199 – 208	453.2185	1356.6336	1356.6344	-0.55	1	20	0.011	1	U	R.CASIQKFGER.A + Glycation (K)
1262	199 – 208	453.2185	1356.6337	1356.6344	-0.48	1	31	0.00078	1	U	R.CASIQKFGER.A + Glycation (K)
1263	199 – 208	453.2186	1356.6339	1356.6344	-0.35	1	31	0.00083	1	U	R.CASIQKFGER.A + Glycation (K)
1264	199 – 208	679.3243	1356.6341	1356.6344	-0.17	1	32	0.00056	1	U	R.CASIQKFGER.A + Glycation (K)
1265	199 – 208	453.2187	1356.6343	1356.6344	-0.016	1	21	0.0073	1	U	R.CASIQKFGER.A + Glycation (K)
931	209 – 217	388.5518	1162.6335	1162.6346	-0.91	1	34	0.00043	1	U	R.ALKAWSVAR.L + Glycation (K)
932	209 – 217	388.5519	1162.6337	1162.6346	-0.75	1	43	4.7e-005	1	U	R.ALKAWSVAR.L + Glycation (K)
933	209 – 217	388.5520	1162.6342	1162.6346	-0.37	1	36	0.00026	1	U	R.ALKAWSVAR.L + Glycation (K)
934	209 – 217	582.3244	1162.6342	1162.6346	-0.30	1	29	0.0013	1	U	R.ALKAWSVAR.L + Glycation (K)
93	212 – 217	345.1900	688.3654	688.3656	-0.29	0	17	0.018	1	U	K.AWSVAR.L
94	212 – 217	345.1901	688.3656	688.3656	-0.026	0	16	0.025	1	U	K.AWSVAR.L
95	212 – 217	345.1901	688.3657	688.3656	0.061	0	17	0.019	1	U	K.AWSVAR.L
96	212 – 217	345.1902	688.3658	688.3656	0.18	0	16	0.025	1	U	K.AWSVAR.L
97	212 – 217	345.1904	688.3662	688.3656	0.79	0	15	0.03	1	U	K.AWSVAR.L
560	218 – 224	505.2818	1008.5491	1008.5491	-0.041	1	20	0.0098	1	U	R.LSQKFPK.A + Glycation (K)
3344	222 – 239	598.0702	2388.2519	2388.2563	-1.86	2	24	0.0042	1	U	K.FPKAEFVEVTKLVTDLTK.V + 2 Glycation (K
3345	222 – 239	797.0917	2388.2532	2388.2563	-1.33	2	18	0.018	1	U	K.FPKAEFVEVTKLVTDLTK.V + 2 Glycation (K
3346	222 – 239	598.0712	2388.2556	2388.2563	-0.32	2	26	0.0027	1	U	K.FPKAEFVEVTKLVTDLTK.V + 2 Glycation (K
3347	222 – 239	598.0712	2388.2556	2388.2563	-0.32	2	28	0.0015	1	U	K.FPKAEFVEVTKLVTDLTK.V + 2 Glycation (K
3348	222 – 239	797.0930	2388.2572	2388.2563	0.35	2	37	0.00018	1	U	K.FPKAEFVEVTKLVTDLTK.V + 2 Glycation (K
3349	222 – 239	1195.1371	2388.2596	2388.2563	1.37	2	58	1.6e-006	1	U	K.FPKAEFVEVTKLVTDLTK.V + 2 Glycation (K
3350	222 – 239	797.0941	2388.2604	2388.2563	1.68	2	47	1.9e-005	1	U	K.FPKAEFVEVTKLVTDLTK.V + 2 Glycation (K
396	225 – 232	461.7475	921.4805	921.4807	-0.26	0	38	0.00014	1	U	K.AEFVEVTK.L
397	225 – 232	461.7476	921.4807	921.4807	-0.060	0	31	0.00084	1	U	K.AEFVEVTK.L
398	225 – 232	461.7477	921.4808	921.4807	0.071	0	38	0.00014	1	U	K.AEFVEVTK.L
2613	225 – 239	927.9998	1853.9851	1853.9874	-1.24	1	81	8.2e-009	1	U	K.AEFVEVTKLVTDLTK.V + Glycation (K)
2614	225 – 239	928.0002	1853.9858	1853.9874	-0.85	1	71	8.1e-008	1	U	K.AEFVEVTKLVTDLTK.V + Glycation (K)
2615	225 – 239	928.0005	1853.9864	1853.9874	-0.51	1	57	2.1e-006	1	U	K.AEFVEVTKLVTDLTK.V + Glycation (K)
2616	225 – 239	619.0029	1853.9870	1853.9874	-0.23	1	29	0.0013	1	U	K.AEFVEVTKLVTDLTK.V + Glycation (K)
2617	225 – 239	619.0031	1853.9873	1853.9874	-0.032	1	41	7.9e-005	1	U	K.AEFVEVTKLVTDLTK.V + Glycation (K)
2618	225 – 239	619.0032	1853.9879	1853.9874	0.26	1	41	8.9e-005	1	U	K.AEFVEVTKLVTDLTK.V + Glycation (K)
2619	225 – 239	928.0014	1853.9882	1853.9874	0.47	1	67	2.1e-007	1	U	K.AEFVEVTKLVTDLTK.V + Glycation (K)
2620	225 – 239	619.0036	1853.9890	1853.9874	0.86	1	37	0.0002	1	U	K.AEFVEVTKLVTDLTK.V + Glycation (K)
2621	225 – 239	928.0019	1853.9892	1853.9874	1.00	1	63	5e-007	1	U	K.AEFVEVTKLVTDLTK.V + Glycation (K)
2622	225 – 239	928.0020	1853.9895	1853.9874	1.13	1	68	1.5e-007	1	U	K.AEFVEVTKLVTDLTK.V + Glycation (K)
2623	225 – 239	928.0024	1853.9902	1853.9874	1.55	1	65	3.3e-007	1	U	K.AEFVEVTKLVTDLTK.V + Glycation (K)
2624	225 – 239	619.0041	1853.9904	1853.9874	1.65	1	41	8.8e-005	1	U	K.AEFVEVTKLVTDLTK.V + Glycation (K)
2625	225 – 239	619.0041	1853.9904	1853.9874	1.65	1	50	9e-006	1	U	K.AEFVEVTKLVTDLTK.V + Glycation (K)
2626	225 – 239	928.0026	1853.9907	1853.9874	1.78	1	53	5.4e-006	1	U	K.AEFVEVTKLVTDLTK.V + Glycation (K)
2627	225 – 239	619.0055	1853.9948	1853.9874	4.01	1	41	7.2e-005	1	U	K.AEFVEVTKLVTDLTK.V + Glycation (K)
2628	225 – 239	619.0057	1853.9954	1853.9874	4.32	1	48	1.4e-005	1	U	K.AEFVEVTKLVTDLTK.V + Glycation (K)
3337	225 – 242	596.0723	2380.2602	2380.2625	-0.96	2	33	0.00055	1	U	K.AEFVEVTKLVTDLTKVHK.E + 2 Glycation (K
3338	225 – 242	794.4274	2380.2603	2380.2625	-0.93	2	40	9.4e-005	1	U	K.AEFVEVTKLVTDLTKVHK.E + 2 Glycation (K
3341	225 – 242	596.0728	2380.2621	2380.2625	-0.15	2	20	0.011	1	U	K.AEFVEVTKLVTDLTKVHK.E + 2 Glycation (K
4146	225 – 256	855.5983	4272.9553	4272.9600	-1.11	3	62	6.1e-007	1	U	K.AEFVEVTKLVTDLTKVHKECCHGDLLECADDR.A
4147	225 – 256	855.5989	4272.9581	4272.9600	-0.46	3	25	0.0032	1	U	K.AEFVEVTKLVTDLTKVHKECCHGDLLECADDR.A
4148	225 – 256	1069.2468	4272.9582	4272.9600	-0.42	3	16	0.026	1	U	K.AEFVEVTKLVTDLTKVHKECCHGDLLECADDR.A
4149	225 – 256	855.5990	4272.9588	4272.9600	-0.28	3	20	0.0095	1	U	K.AEFVEVTKLVTDLTKVHKECCHGDLLECADDR.A
4150	225 – 256	713.1672	4272.9597	4272.9600	-0.070	3	62	5.6e-007	1	U	K.AEFVEVTKLVTDLTKVHKECCHGDLLECADDR.A

Query	Start - End	Observed	Mr(expt)	Mr(calc)	ppm	M	Score	Expect	Rank	U	Peptide
4151	225 - 256	1069.2474	4272.9605	4272.9600	0.11	3	39	0.00011	1	U	K.AEFVEVTKLVTDLTKVHKECCHGDLLECADDR.A
4152	225 - 256	713.1675	4272.9612	4272.9600	0.27	3	68	1.5e-007	1	U	K.AEFVEVTKLVTDLTKVHKECCHGDLLECADDR.A
4153	225 - 256	1069.2478	4272.9620	4272.9600	0.47	3	36	0.00027	1	U	K.AEFVEVTKLVTDLTKVHKECCHGDLLECADDR.A
197	233 - 239	395.2393	788.4641	788.4644	-0.36	0	35	0.00029	1	U	K.LVTDLTK.V
198	233 - 239	395.2394	788.4643	788.4644	-0.13	0	36	0.00027	1	U	K.LVTDLTK.V
199	233 - 239	395.2394	788.4643	788.4644	-0.055	0	36	0.00028	1	U	K.LVTDLTK.V
200	233 - 239	395.2395	788.4644	788.4644	0.022	0	36	0.00027	1	U	K.LVTDLTK.V
201	233 - 239	395.2395	788.4645	788.4644	0.17	0	36	0.00027	1	U	K.LVTDLTK.V
1186	233 - 242	439.2538	1314.7394	1314.7395	-0.049	1	21	0.0073	1	U	K.LVTDLTKVHK.E + Glycation (K)
3840	233 - 256	642.4942	3207.4346	3207.4370	-0.75	2	59	1.3e-006	1	U	K.LVTDLTKVHKECCHGDLLECADDR.A + 2 Glyca
3841	233 - 256	642.4943	3207.4352	3207.4370	-0.56	2	45	3e-005	1	U	K.LVTDLTKVHKECCHGDLLECADDR.A + 2 Glyca
3203	240 - 256	569.7384	2274.9245	2274.9304	-2.58	1	37	0.0002	1	U	K.VHKECCHGDLLECADDR.A + Glycation (K)
3204	240 - 256	759.3164	2274.9274	2274.9304	-1.31	1	146	2.6e-015	1	U	K.VHKECCHGDLLECADDR.A + Glycation (K)
3205	240 - 256	759.3166	2274.9279	2274.9304	-1.07	1	154	4.3e-016	1	U	K.VHKECCHGDLLECADDR.A + Glycation (K)
3206	240 - 256	759.3167	2274.9283	2274.9304	-0.91	1	146	2.6e-015	1	U	K.VHKECCHGDLLECADDR.A + Glycation (K)
3207	240 - 256	1138.4714	2274.9283	2274.9304	-0.90	1	92	6.2e-010	1	U	K.VHKECCHGDLLECADDR.A + Glycation (K)
3208	240 - 256	455.9930	2274.9284	2274.9304	-0.88	1	28	0.0016	1	U	K.VHKECCHGDLLECADDR.A + Glycation (K)
3209	240 - 256	569.7394	2274.9284	2274.9304	-0.86	1	39	0.00011	1	U	K.VHKECCHGDLLECADDR.A + Glycation (K)
3210	240 - 256	759.3168	2274.9287	2274.9304	-0.75	1	113	5.1e-012	1	U	K.VHKECCHGDLLECADDR.A + Glycation (K)
3211	240 - 256	759.3169	2274.9288	2274.9304	-0.67	1	146	2.7e-015	1	U	K.VHKECCHGDLLECADDR.A + Glycation (K)
3212	240 - 256	455.9931	2274.9290	2274.9304	-0.62	1	30	0.00091	1	U	K.VHKECCHGDLLECADDR.A + Glycation (K)
3213	240 - 256	455.9931	2274.9291	2274.9304	-0.55	1	18	0.014	1	U	K.VHKECCHGDLLECADDR.A + Glycation (K)
3214	240 - 256	569.7396	2274.9291	2274.9304	-0.54	1	36	0.00024	1	U	K.VHKECCHGDLLECADDR.A + Glycation (K)
3215	240 - 256	569.7397	2274.9299	2274.9304	-0.21	1	35	0.00031	1	U	K.VHKECCHGDLLECADDR.A + Glycation (K)
3216	240 - 256	569.7397	2274.9299	2274.9304	-0.21	1	43	5e-005	1	U	K.VHKECCHGDLLECADDR.A + Glycation (K)
3217	240 - 256	1138.4723	2274.9300	2274.9304	-0.15	1	96	2.7e-010	1	U	K.VHKECCHGDLLECADDR.A + Glycation (K)
3218	240 - 256	1138.4724	2274.9303	2274.9304	-0.047	1	100	1e-010	1	U	K.VHKECCHGDLLECADDR.A + Glycation (K)
3219	240 - 256	569.7400	2274.9309	2274.9304	0.21	1	36	0.00023	1	U	K.VHKECCHGDLLECADDR.A + Glycation (K)
3220	240 - 256	569.7403	2274.9321	2274.9304	0.76	1	43	5.2e-005	1	U	K.VHKECCHGDLLECADDR.A + Glycation (K)
2309	243 - 256	875.3340	1748.6534	1748.6553	-1.06	0	86	2.7e-009	1	U	K.ECCHGDLLECADDR.A
2310	243 - 256	875.3342	1748.6538	1748.6553	-0.84	0	115	3.5e-012	1	U	K.ECCHGDLLECADDR.A
2311	243 - 256	875.3342	1748.6539	1748.6553	-0.77	0	102	7.1e-011	1	U	K.ECCHGDLLECADDR.A
2312	243 - 256	583.8920	1748.6543	1748.6553	-0.57	0	69	1.4e-007	1	U	K.ECCHGDLLECADDR.A
2313	243 - 256	583.8921	1748.6544	1748.6553	-0.47	0	51	7.4e-006	1	U	K.ECCHGDLLECADDR.A
2314	243 - 256	583.8922	1748.6546	1748.6553	-0.36	0	81	8.1e-009	1	U	K.ECCHGDLLECADDR.A
2315	243 - 256	583.8922	1748.6548	1748.6553	-0.26	0	68	1.4e-007	1	U	K.ECCHGDLLECADDR.A
2316	243 - 256	583.8923	1748.6552	1748.6553	-0.055	0	69	1.4e-007	1	U	K.ECCHGDLLECADDR.A
2317	243 - 256	583.8923	1748.6552	1748.6553	-0.055	0	68	1.5e-007	1	U	K.ECCHGDLLECADDR.A
2318	243 - 256	583.8925	1748.6556	1748.6553	0.17	0	80	9.4e-009	1	U	K.ECCHGDLLECADDR.A
2319	243 - 256	583.8928	1748.6566	1748.6553	0.79	0	75	3.2e-008	1	U	K.ECCHGDLLECADDR.A
3067	257 - 273	1052.4901	2102.9657	2102.9678	-0.99	1	61	8e-007	1	U	R.ADLAKYICDNQDTISSK.L + Glycation (K)
3068	257 - 273	701.9960	2102.9661	2102.9678	-0.80	1	89	1.2e-009	1	U	R.ADLAKYICDNQDTISSK.L + Glycation (K)
3069	257 - 273	1052.4905	2102.9664	2102.9678	-0.64	1	77	2.1e-008	1	U	R.ADLAKYICDNQDTISSK.L + Glycation (K)
3070	257 - 273	701.9961	2102.9664	2102.9678	-0.63	1	95	3.5e-010	1	U	R.ADLAKYICDNQDTISSK.L + Glycation (K)
3071	257 - 273	701.9962	2102.9668	2102.9678	-0.44	1	95	3.3e-010	1	U	R.ADLAKYICDNQDTISSK.L + Glycation (K)
3072	257 - 273	1052.4907	2102.9669	2102.9678	-0.41	1	61	7.4e-007	1	U	R.ADLAKYICDNQDTISSK.L + Glycation (K)
3073	257 - 273	701.9964	2102.9674	2102.9678	-0.18	1	89	1.2e-009	1	U	R.ADLAKYICDNQDTISSK.L + Glycation (K)
3074	257 - 273	701.9966	2102.9681	2102.9678	0.16	1	82	6e-009	1	U	R.ADLAKYICDNQDTISSK.L + Glycation (K)
3075	257 - 273	701.9968	2102.9685	2102.9678	0.34	1	95	3.5e-010	1	U	R.ADLAKYICDNQDTISSK.L + Glycation (K)
3076	257 - 273	1052.4921	2102.9696	2102.9678	0.87	1	65	2.8e-007	1	U	R.ADLAKYICDNQDTISSK.L + Glycation (K)
3454	257 - 275	836.4057	2506.1953	2506.1996	-1.73	2	34	0.00037	1	U	R.ADLAKYICDNQDTISSKLK.E + 2 Glycation (
3455	257 - 275	836.4060	2506.1962	2506.1996	-1.36	2	39	0.00013	1	U	R.ADLAKYICDNQDTISSKLK.E + 2 Glycation (
3456	257 - 275	627.5566	2506.1975	2506.1996	-0.86	2	22	0.0069	1	U	R.ADLAKYICDNQDTISSKLK.E + 2 Glycation (
3457	257 - 275	627.5570	2506.1987	2506.1996	-0.36	2	16	0.023	1	U	R.ADLAKYICDNQDTISSKLK.E + 2 Glycation (
1458	262 - 273	722.3237	1442.6329	1442.6347	-1.28	0	72	6.4e-008	1	U	K.YICDNQDTISSK.L
1459	262 - 273	722.3238	1442.6331	1442.6347	-1.11	0	55	3.2e-006	1	U	K.YICDNQDTISSK.L
1460	262 - 273	722.3243	1442.6340	1442.6347	-0.51	0	86	2.8e-009	1	U	K.YICDNQDTISSK.L
1461	262 - 273	722.3243	1442.6340	1442.6347	-0.51	0	85	2.9e-009	1	U	K.YICDNQDTISSK.L
1462	262 - 273	722.3246	1442.6346	1442.6347	-0.098	0	85	2.9e-009	1	U	K.YICDNQDTISSK.L
1463	262 - 273	722.3247	1442.6347	1442.6347	-0.0014	0	85	3.1e-009	1	U	K.YICDNQDTISSK.L
2608	262 - 275	616.2960	1845.8661	1845.8666	-0.29	1	36	0.00024	1	U	K.YICDNQDTISSKLK.E + Glycation (K)
2609	262 - 275	616.2961	1845.8666	1845.8666		1	23	0.0054	1	U	K.YICDNQDTISSKLK.E + Glycation (K)
2610	262 - 275	616.2962	1845.8668	1845.8666	0.098	1	33	0.00049	1	U	K.YICDNQDTISSKLK.E + Glycation (K)
1756	274 - 285	766.8928	1531.7711	1531.7738	-1.77	2	66	2.7e-007	1	U	K.LKECCDKPLLEK.S
1757	274 - 285	766.8931	1531.7717	1531.7738	-1.37	2	69	1.3e-007	1	U	K.LKECCDKPLLEK.S
1758	274 - 285	511.5982	1531.7728	1531.7738	-0.65	2	51	8.5e-006	1	U	K.LKECCDKPLLEK.S
1759	274 - 285	383.9505	1531.7730	1531.7738	-0.51	2	22	0.0063	1	U	K.LKECCDKPLLEK.S
1760	274 - 285	383.9506	1531.7733	1531.7738	-0.36	2	19	0.011	1	U	K.LKECCDKPLLEK.S
1761	274 - 285	383.9506	1531.7734	1531.7738	-0.28	2	35	0.0003	1	U	K.LKECCDKPLLEK.S
1762	274 - 285	511.5985	1531.7737	1531.7738	-0.067	2	58	1.6e-006	1	U	K.LKECCDKPLLEK.S
1763	274 - 285	511.5988	1531.7745	1531.7738	0.48	2	54	3.6e-006	1	U	K.LKECCDKPLLEK.S
2143	274 - 285	847.9198	1693.8250	1693.8266	-0.93	2	45	3.1e-005	1	U	K.LKECCDKPLLEK.S + Glycation (K)
2144	274 - 285	847.9201	1693.8256	1693.8266	-0.58	2	45	3e-005	1	U	K.LKECCDKPLLEK.S + Glycation (K)
2145	274 - 285	565.6160	1693.8261	1693.8266	-0.32	2	32	0.00058	1	U	K.LKECCDKPLLEK.S + Glycation (K)
2146	274 - 285	424.4638	1693.8261	1693.8266	-0.29	2	17	0.019	1	U	K.LKECCDKPLLEK.S + Glycation (K)
2147	274 - 285	424.4639	1693.8264	1693.8266	-0.15	2	66	2.3e-007	1	U	K.LKECCDKPLLEK.S + Glycation (K)
2148	274 - 285	565.6161	1693.8264	1693.8266	-0.11	2	24	0.004	1	U	K.LKECCDKPLLEK.S + Glycation (K)
2149	274 - 285	565.6161	1693.8264	1693.8266	-0.11	2	50	9.1e-006	1	U	K.LKECCDKPLLEK.S + Glycation (K)
2150	274 - 285	424.4639	1693.8265	1693.8266	-0.079	2	39	0.00011	1	U	K.LKECCDKPLLEK.S + Glycation (K)
2151	274 - 285	424.4640	1693.8269	1693.8266	0.13	2	79	1.2e-008	1	U	K.LKECCDKPLLEK.S + Glycation (K)
2152	274 - 285	565.6163	1693.8270	1693.8266	0.21	2	50	9.6e-006	1	U	K.LKECCDKPLLEK.S + Glycation (K)
2153	274 - 285	424.4641	1693.8275	1693.8266	0.49	2	31	0.00079	1	U	K.LKECCDKPLLEK.S + Glycation (K)
2154	274 - 285	565.6165	1693.8276	1693.8266	0.54	2	46	2.6e-005	1	U	K.LKECCDKPLLEK.S + Glycation (K)
2632	274 - 285	928.9466	1855.8786	1855.8795	-0.45	2	18	0.016	1	U	K.LKECCDKPLLEK.S + 2 Glycation (K)
2635	274 - 285	464.9771	1855.8793	1855.8795	-0.065	2	16	0.023	1	U	K.LKECCDKPLLEK.S + 2 Glycation (K)
3685	274 - 294	582.8812	2909.3698	2909.3708	-0.34	3	39	0.00013	1	U	K.LKECCDKPLLEKSHCIAEVEK.D + 2 Glycatio
3686	274 - 294	582.8812	2909.3698	2909.3708	-0.34	3	42	6.1e-005	1	U	K.LKECCDKPLLEKSHCIAEVEK.D + 2 Glycatio

Query	Start – End	Observed	Mr(expt)	Mr(calc)	ppm	M	Score	Expect	Rank	U	Peptide
3687	274 – 294	728.3502	2909.3718	2909.3708	0.34	3	29	0.0013	1	U	K.LKECCDKPLLEKSHCIAEVEK.D + 2 Glycatio
1125	276 – 285	431.2053	1290.5941	1290.5948	-0.51	1	34	0.00045	1	U	K.ECCDKPLLEK.S
1126	276 – 285	646.3044	1290.5943	1290.5948	-0.36	1	45	3e-005	1	U	K.ECCDKPLLEK.S
1127	276 – 285	431.2054	1290.5945	1290.5948	-0.23	1	37	0.00021	1	U	K.ECCDKPLLEK.S
1128	276 – 285	431.2055	1290.5946	1290.5948	-0.16	1	54	4.1e-006	1	U	K.ECCDKPLLEK.S
1129	276 – 285	646.3046	1290.5947	1290.5948	-0.062	1	53	4.7e-006	1	U	K.ECCDKPLLEK.S
1130	276 – 285	646.3047	1290.5948	1290.5948	0.031	1	72	6.4e-008	1	U	K.ECCDKPLLEK.S
1131	276 – 285	431.2059	1290.5959	1290.5948	0.84	1	54	4e-006	1	U	K.ECCDKPLLEK.S
1494	276 – 285	727.3306	1452.6466	1452.6476	-0.71	1	28	0.0015	1	U	K.ECCDKPLLEK.S + Glycation (K)
1495	276 – 285	727.3309	1452.6472	1452.6476	-0.29	1	24	0.0044	1	U	K.ECCDKPLLEK.S + Glycation (K)
1497	276 – 285	727.3310	1452.6474	1452.6476	-0.13	1	25	0.0035	1	U	K.ECCDKPLLEK.S + Glycation (K)
1498	276 – 285	485.2234	1452.6483	1452.6476	0.50	1	20	0.011	1	U	K.ECCDKPLLEK.S + Glycation (K)
3452	276 – 294	627.5417	2506.1379	2506.1389	-0.41	2	39	0.00012	1	U	K.ECCDKPLLEKSHCIAEVEK.D + Glycation (K)
3453	276 – 294	627.5420	2506.1389	2506.1389	-0.030	2	46	2.5e-005	1	U	K.ECCDKPLLEKSHCIAEVEK.D + Glycation (K)
3550	276 – 294	668.0541	2668.1872	2668.1918	-1.70	2	31	0.00075	1	U	K.ECCDKPLLEKSHCIAEVEK.D + 2 Glycation
744	286 – 294	358.1744	1071.5012	1071.5019	-0.61	0	35	0.00035	1	U	K.SHCIAEVEK.D
745	286 – 294	358.1744	1071.5014	1071.5019	-0.44	0	36	0.00023	1	U	K.SHCIAEVEK.D
746	286 – 294	358.1744	1071.5014	1071.5019	-0.44	0	39	0.00012	1	U	K.SHCIAEVEK.D
747	286 – 294	358.1745	1071.5018	1071.5019	-0.11	0	39	0.00012	1	U	K.SHCIAEVEK.D
748	286 – 294	536.7582	1071.5018	1071.5019	-0.064	0	41	7.6e-005	1	U	K.SHCIAEVEK.D
749	286 – 294	536.7582	1071.5018	1071.5019	-0.064	0	50	8.9e-006	1	U	K.SHCIAEVEK.D
750	286 – 294	536.7582	1071.5018	1071.5019	-0.064	0	46	2.7e-005	1	U	K.SHCIAEVEK.D
751	286 – 294	536.7582	1071.5019	1071.5019	0.048	0	45	3.3e-005	1	U	K.SHCIAEVEK.D
752	286 – 294	358.1746	1071.5021	1071.5019	0.23	0	39	0.00012	1	U	K.SHCIAEVEK.D
3737	286 – 312	1003.8233	3008.4481	3008.4437	1.46	1	44	4.2e-005	1	U	K.SHCIAEVEKDAIPENLPPLTADFAEDK.D
3828	286 – 312	1057.8313	3170.4721	3170.4965	-7.71	1	58	1.7e-006	1	U	K.SHCIAEVEKDAIPENLPPLTADFAEDK.D + Glyc
3829	286 – 312	793.6303	3170.4919	3170.4965	-1.45	1	53	5.5e-006	1	U	K.SHCIAEVEKDAIPENLPPLTADFAEDK.D + Glyc
3830	286 – 312	793.6307	3170.4939	3170.4965	-0.84	1	56	2.6e-006	1	U	K.SHCIAEVEKDAIPENLPPLTADFAEDK.D + Glyc
3831	286 – 312	1057.8400	3170.4981	3170.4965	0.50	1	59	1.1e-006	1	U	K.SHCIAEVEKDAIPENLPPLTADFAEDK.D + Glyc
3954	286 – 316	878.6727	3510.6616	3510.6647	-0.88	2	72	6e-008	1	U	K.SHCIAEVEKDAIPENLPPLTADFAEDKDVCK.N
3955	286 – 316	1171.2293	3510.6659	3510.6647	0.36	2	40	0.0001	1	U	K.SHCIAEVEKDAIPENLPPLTADFAEDKDVCK.N
3956	286 – 316	703.1409	3510.6683	3510.6647	1.03	2	22	0.0059	1	U	K.SHCIAEVEKDAIPENLPPLTADFAEDKDVCK.N
3957	286 – 316	878.6748	3510.6700	3510.6647	1.53	2	74	3.9e-008	1	U	K.SHCIAEVEKDAIPENLPPLTADFAEDKDVCK.N
3992	286 – 316	919.1848	3672.7101	3672.7175	-2.00	2	64	3.7e-007	1	U	K.SHCIAEVEKDAIPENLPPLTADFAEDKDVCK.N +
3993	286 – 316	735.5505	3672.7163	3672.7175	-0.32	2	34	0.00039	1	U	K.SHCIAEVEKDAIPENLPPLTADFAEDKDVCK.N +
3994	286 – 316	919.1866	3672.7175	3672.7175	0.0033	2	62	6.4e-007	1	U	K.SHCIAEVEKDAIPENLPPLTADFAEDKDVCK.N +
3995	286 – 316	919.1869	3672.7185	3672.7175	0.26	2	64	3.9e-007	1	U	K.SHCIAEVEKDAIPENLPPLTADFAEDKDVCK.N +
3996	286 – 316	1225.2469	3672.7188	3672.7175	0.35	2	49	1.4e-005	1	U	K.SHCIAEVEKDAIPENLPPLTADFAEDKDVCK.N +
3997	286 – 316	1225.2474	3672.7205	3672.7175	0.82	2	53	5.2e-006	1	U	K.SHCIAEVEKDAIPENLPPLTADFAEDKDVCK.N +
3998	286 – 316	735.5516	3672.7215	3672.7175	1.09	2	60	9.1e-007	1	U	K.SHCIAEVEKDAIPENLPPLTADFAEDKDVCK.N +
3999	286 – 316	1225.2479	3672.7220	3672.7175	1.24	2	70	9.4e-008	1	U	K.SHCIAEVEKDAIPENLPPLTADFAEDKDVCK.N +
4000	286 – 316	919.1885	3672.7248	3672.7175	2.00	2	67	2e-007	1	U	K.SHCIAEVEKDAIPENLPPLTADFAEDKDVCK.N +
4058	286 – 316	767.9600	3834.7637	3834.7703	-1.72	2	21	0.0086	1	U	K.SHCIAEVEKDAIPENLPPLTADFAEDKDVCK.N +
4059	286 – 316	959.6984	3834.7643	3834.7703	-1.56	2	50	9.3e-006	1	U	K.SHCIAEVEKDAIPENLPPLTADFAEDKDVCK.N +
4060	286 – 316	959.6988	3834.7663	3834.7703	-1.05	2	23	0.0051	1	U	K.SHCIAEVEKDAIPENLPPLTADFAEDKDVCK.N +
4061	286 – 316	767.9612	3834.7695	3834.7703	-0.21	2	40	0.0001	1	U	K.SHCIAEVEKDAIPENLPPLTADFAEDKDVCK.N +
4062	286 – 316	959.6998	3834.7702	3834.7703	-0.024	2	54	3.7e-006	1	U	K.SHCIAEVEKDAIPENLPPLTADFAEDKDVCK.N +
4063	286 – 316	1279.2649	3834.7728	3834.7703	0.66	2	50	9.8e-006	1	U	K.SHCIAEVEKDAIPENLPPLTADFAEDKDVCK.N +
4181	286 – 322	1102.5250	4406.0710	4406.0570	3.18	3	45	3.2e-005	1	U	K.SHCIAEVEKDAIPENLPPLTADFAEDKDVCKNYQE
4182	286 – 322	735.3525	4406.0716	4406.0570	3.32	3	54	3.6e-006	1	U	K.SHCIAEVEKDAIPENLPPLTADFAEDKDVCKNYQE
4183	286 – 322	882.2231	4406.0790	4406.0570	5.01	3	40	9.6e-005	1	U	K.SHCIAEVEKDAIPENLPPLTADFAEDKDVCKNYQE
4198	286 – 322	914.6290	4568.1084	4568.1098	-0.30	3	19	0.013	1	U	K.SHCIAEVEKDAIPENLPPLTADFAEDKDVCKNYQE
4199	286 – 322	762.3587	4568.1085	4568.1098	-0.27	3	19	0.013	1	U	K.SHCIAEVEKDAIPENLPPLTADFAEDKDVCKNYQE
4200	286 – 322	914.6291	4568.1089	4568.1098	-0.20	3	22	0.0069	1	U	K.SHCIAEVEKDAIPENLPPLTADFAEDKDVCKNYQE
4201	286 – 322	1143.0353	4568.1120	4568.1098	0.49	3	20	0.011	1	U	K.SHCIAEVEKDAIPENLPPLTADFAEDKDVCKNYQE
4202	286 – 322	914.6307	4568.1170	4568.1098	1.58	3	53	4.8e-006	1	U	K.SHCIAEVEKDAIPENLPPLTADFAEDKDVCKNYQE
4258	286 – 322	789.3677	4730.1624	4730.1626	-0.053	3	19	0.012	1	U	K.SHCIAEVEKDAIPENLPPLTADFAEDKDVCKNYQE
4259	286 – 322	1577.7294	4730.1663	4730.1626	0.78	3	37	0.00021	1	U	K.SHCIAEVEKDAIPENLPPLTADFAEDKDVCKNYQE
4261	286 – 322	1183.5493	4730.1682	4730.1626	1.18	3	16	0.027	1	U	K.SHCIAEVEKDAIPENLPPLTADFAEDKDVCKNYQE
2867	295 – 312	978.4827	1954.9509	1954.9524	-0.74	0	50	1e-005	1	U	K.DAIPENLPPLTADFAEDK.D
2868	295 – 312	978.4827	1954.9509	1954.9524	-0.74	0	72	6.5e-008	1	U	K.DAIPENLPPLTADFAEDK.D
2869	295 – 312	978.4828	1954.9511	1954.9524	-0.62	0	69	1.3e-007	1	U	K.DAIPENLPPLTADFAEDK.D
2870	295 – 312	978.4830	1954.9514	1954.9524	-0.50	0	49	1.2e-005	1	U	K.DAIPENLPPLTADFAEDK.D
3416	295 – 316	820.0643	2457.1710	2457.1733	-0.96	1	64	4.2e-007	1	U	K.DAIPENLPPLTADFAEDKDVCK.N
3417	295 – 316	820.0644	2457.1715	2457.1733	-0.74	1	62	7e-007	1	U	K.DAIPENLPPLTADFAEDKDVCK.N
3418	295 – 316	820.0648	2457.1725	2457.1733	-0.36	1	62	5.6e-007	1	U	K.DAIPENLPPLTADFAEDKDVCK.N
3419	295 – 316	820.0648	2457.1726	2457.1733	-0.29	1	61	8.4e-007	1	U	K.DAIPENLPPLTADFAEDKDVCK.N
3420	295 – 316	1229.5944	2457.1742	2457.1733	0.34	1	82	6e-009	1	U	K.DAIPENLPPLTADFAEDKDVCK.N
3512	295 – 316	1310.6186	2619.2227	2619.2262	-1.30	1	45	3.4e-005	1	U	K.DAIPENLPPLTADFAEDKDVCK.N + Glycation
3513	295 – 316	874.0817	2619.2232	2619.2262	-1.14	1	46	2.5e-005	1	U	K.DAIPENLPPLTADFAEDKDVCK.N + Glycation
3514	295 – 316	874.0817	2619.2232	2619.2262	-1.14	1	57	2.2e-006	1	U	K.DAIPENLPPLTADFAEDKDVCK.N + Glycation
3515	295 – 316	874.0817	2619.2234	2619.2262	-1.07	1	58	1.6e-006	1	U	K.DAIPENLPPLTADFAEDKDVCK.N + Glycation
3516	295 – 316	874.0818	2619.2235	2619.2262	-1.00	1	47	2.1e-005	1	U	K.DAIPENLPPLTADFAEDKDVCK.N + Glycation
3517	295 – 316	874.0821	2619.2244	2619.2262	-0.66	1	46	2.4e-005	1	U	K.DAIPENLPPLTADFAEDKDVCK.N + Glycation
3518	295 – 316	1310.6221	2619.2296	2619.2262	1.31	1	17	0.018	1	U	K.DAIPENLPPLTADFAEDKDVCK.N + Glycation
3867	295 – 322	1118.5278	3352.5617	3352.5656	-1.19	2	25	0.0034	1	U	K.DAIPENLPPLTADFAEDKDVCKNYQEAK.D + Gly
3868	295 – 322	839.1478	3352.5622	3352.5656	-1.02	2	14	0.04	1	U	K.DAIPENLPPLTADFAEDKDVCKNYQEAK.D + Gly
3870	295 – 322	839.1484	3352.5647	3352.5656	-0.29	2	13	0.047	1	U	K.DAIPENLPPLTADFAEDKDVCKNYQEAK.D + Gly
3871	295 – 322	1118.5292	3352.5657	3352.5656	0.012	2	38	0.00016	1	U	K.DAIPENLPPLTADFAEDKDVCKNYQEAK.D + Gly
1403	313 – 322	472.8817	1415.6234	1415.6238	-0.32	1	26	0.0023	1	U	K.DVCKNYQEAK.D + Glycation (K)
1404	313 – 322	708.8190	1415.6235	1415.6238	-0.23	1	40	0.0001	1	U	K.DVCKNYQEAK.D + Glycation (K)
1405	313 – 322	472.8819	1415.6239	1415.6238	0.023	1	24	0.0044	1	U	K.DVCKNYQEAK.D + Glycation (K)
3421	317 – 335	821.7152	2462.1238	2462.1277	-1.60	1	71	8.7e-008	1	U	K.NYQEAKDAFLGSFLYEYSR.R + Glycation (K)
3422	317 – 335	821.7157	2462.1253	2462.1277	-1.01	1	83	4.9e-009	1	U	K.NYQEAKDAFLGSFLYEYSR.R + Glycation (K)
3423	317 – 335	821.7161	2462.1266	2462.1277	-0.48	1	83	4.6e-009	1	U	K.NYQEAKDAFLGSFLYEYSR.R + Glycation (K)
3424	317 – 335	821.7163	2462.1271	2462.1277	-0.26	1	73	4.8e-008	1	U	K.NYQEAKDAFLGSFLYEYSR.R + Glycation (K)
3425	317 – 335	821.7163	2462.1271	2462.1277	-0.26	1	79	1.2e-008	1	U	K.NYQEAKDAFLGSFLYEYSR.R + Glycation (K)

Query	Start - End	Observed	Mr(expt)	Mr(calc)	ppm	M	Score	Expect	Rank	U	Peptide
3426	317 - 335	1232.0710	2462.1275	2462.1277	-0.089	1	97	1.9e-010	1	U	K.NYQEAKDAFLGSFLYEYSR.R + Glycation (K)
1834	323 - 335	784.3743	1566.7340	1566.7354	-0.93	0	59	1.3e-006	1	U	K.DAFLGSFLYEYSR.R
1835	323 - 335	784.3744	1566.7342	1566.7354	-0.78	0	72	6.8e-008	1	U	K.DAFLGSFLYEYSR.R
1836	323 - 335	784.3745	1566.7343	1566.7354	-0.70	0	76	2.4e-008	1	U	K.DAFLGSFLYEYSR.R
1439	336 - 347	720.4088	1438.8031	1438.8045	-0.97	1	55	2.9e-006	1	U	R.RHPEYAVSVLLR.L
1440	336 - 347	720.4089	1438.8032	1438.8045	-0.88	1	53	5.2e-006	1	U	R.RHPEYAVSVLLR.L
1441	336 - 347	720.4089	1438.8032	1438.8045	-0.88	1	54	3.6e-006	1	U	R.RHPEYAVSVLLR.L
1442	336 - 347	480.6085	1438.8036	1438.8045	-0.57	1	49	1.2e-005	1	U	R.RHPEYAVSVLLR.L
1443	336 - 347	720.4091	1438.8037	1438.8045	-0.54	1	60	9.7e-007	1	U	R.RHPEYAVSVLLR.L
1444	336 - 347	720.4092	1438.8038	1438.8045	-0.45	1	67	2e-007	1	U	R.RHPEYAVSVLLR.L
1445	336 - 347	720.4092	1438.8038	1438.8045	-0.45	1	50	1.1e-005	1	U	R.RHPEYAVSVLLR.L
1446	336 - 347	480.6086	1438.8039	1438.8045	-0.38	1	47	2e-005	1	U	R.RHPEYAVSVLLR.L
1447	336 - 347	480.6087	1438.8042	1438.8045	-0.19	1	51	7.5e-006	1	U	R.RHPEYAVSVLLR.L
1448	336 - 347	480.6087	1438.8044	1438.8045	-0.067	1	60	9.3e-007	1	U	R.RHPEYAVSVLLR.L
1449	336 - 347	480.6088	1438.8045	1438.8045	-0.0049	1	63	4.9e-007	1	U	R.RHPEYAVSVLLR.L
1450	336 - 347	480.6089	1438.8048	1438.8045	0.20	1	63	5e-007	1	U	R.RHPEYAVSVLLR.L
1451	336 - 347	480.6089	1438.8048	1438.8045	0.27	1	63	5.6e-007	1	U	R.RHPEYAVSVLLR.L
1452	336 - 347	480.6090	1438.8052	1438.8045	0.52	1	63	5.1e-007	1	U	R.RHPEYAVSVLLR.L
1453	336 - 347	480.6092	1438.8057	1438.8045	0.89	1	63	5.1e-007	1	U	R.RHPEYAVSVLLR.L
1454	336 - 347	480.6096	1438.8069	1438.8045	1.66	1	63	5e-007	1	U	R.RHPEYAVSVLLR.L
1087	337 - 347	642.3583	1282.7021	1282.7034	-0.96	0	53	5e-006	1	U	R.HPEYAVSVLLR.L
1088	337 - 347	642.3584	1282.7022	1282.7034	-0.86	0	57	2e-006	1	U	R.HPEYAVSVLLR.L
1089	337 - 347	642.3585	1282.7024	1282.7034	-0.77	0	57	1.9e-006	1	U	R.HPEYAVSVLLR.L
1090	337 - 347	642.3586	1282.7027	1282.7034	-0.49	0	56	2.4e-006	1	U	R.HPEYAVSVLLR.L
1091	337 - 347	642.3590	1282.7033	1282.7034	-0.0047	0	63	4.8e-007	1	U	R.HPEYAVSVLLR.L
1092	337 - 347	428.5751	1282.7034	1282.7034	0.022	0	57	2.2e-006	1	U	R.HPEYAVSVLLR.L
1093	337 - 347	428.5751	1282.7034	1282.7034	0.022	0	60	9.7e-007	1	U	R.HPEYAVSVLLR.L
1094	337 - 347	642.3590	1282.7035	1282.7034	0.089	0	64	3.8e-007	1	U	R.HPEYAVSVLLR.L
1095	337 - 347	428.5751	1282.7035	1282.7034	0.092	0	50	9.3e-006	1	U	R.HPEYAVSVLLR.L
1096	337 - 347	428.5751	1282.7035	1282.7034	0.092	0	57	2.1e-006	1	U	R.HPEYAVSVLLR.L
1097	337 - 347	428.5751	1282.7036	1282.7034	0.16	0	53	4.5e-006	1	U	R.HPEYAVSVLLR.L
1098	337 - 347	642.3594	1282.7043	1282.7034	0.76	0	28	0.0017	1	U	R.HPEYAVSVLLR.L
2926	348 - 362	659.6311	1975.8715	1975.8754	-1.99	1	50	9.3e-006	1	U	R.LAKEYEATLEECCAK.D + Glycation (K)
2927	348 - 362	988.9438	1975.8730	1975.8754	-1.21	1	77	2.1e-008	1	U	R.LAKEYEATLEECCAK.D + Glycation (K)
2928	348 - 362	988.9440	1975.8734	1975.8754	-1.02	1	82	6.8e-009	1	U	R.LAKEYEATLEECCAK.D + Glycation (K)
2929	348 - 362	988.9441	1975.8736	1975.8754	-0.90	1	69	1.4e-007	1	U	R.LAKEYEATLEECCAK.D + Glycation (K)
2930	348 - 362	988.9442	1975.8739	1975.8754	-0.78	1	65	3.4e-007	1	U	R.LAKEYEATLEECCAK.D + Glycation (K)
2931	348 - 362	659.6320	1975.8742	1975.8754	-0.60	1	50	9.6e-006	1	U	R.LAKEYEATLEECCAK.D + Glycation (K)
2932	348 - 362	659.6320	1975.8742	1975.8754	-0.60	1	54	3.9e-006	1	U	R.LAKEYEATLEECCAK.D + Glycation (K)
2933	348 - 362	659.6321	1975.8746	1975.8754	-0.42	1	74	3.9e-008	1	U	R.LAKEYEATLEECCAK.D + Glycation (K)
2934	348 - 362	659.6325	1975.8757	1975.8754	0.15	1	75	2.9e-008	1	U	R.LAKEYEATLEECCAK.D + Glycation (K)
2935	348 - 362	659.6329	1975.8770	1975.8754	0.78	1	55	3.3e-006	1	U	R.LAKEYEATLEECCAK.D + Glycation (K)
4001	348 - 375	919.3954	3673.5527	3673.5634	-2.90	2	32	0.00066	1	U	R.LAKEYEATLEECCAKDDPHACYSTVFDK.L + 2
4002	348 - 375	735.7191	3673.5592	3673.5634	-1.13	2	22	0.006	1	U	R.LAKEYEATLEECCAKDDPHACYSTVFDK.L + 2
4003	348 - 375	919.3978	3673.5620	3673.5634	-0.38	2	31	0.00081	1	U	R.LAKEYEATLEECCAKDDPHACYSTVFDK.L + 2
1683	351 - 362	751.8098	1501.6049	1501.6065	-1.01	0	59	1.2e-006	1	U	K.EYEATLEECCAK.D
1684	351 - 362	751.8099	1501.6053	1501.6065	-0.75	0	59	1.2e-006	1	U	K.EYEATLEECCAK.D
1685	351 - 362	751.8101	1501.6057	1501.6065	-0.51	0	72	6.8e-008	1	U	K.EYEATLEECCAK.D
1686	351 - 362	751.8102	1501.6058	1501.6065	-0.43	0	72	6.8e-008	1	U	K.EYEATLEECCAK.D
1687	351 - 362	751.8104	1501.6062	1501.6065	-0.20	0	77	2.1e-008	1	U	K.EYEATLEECCAK.D
3837	351 - 375	800.8298	3199.2900	3199.2944	-1.38	1	28	0.0016	1	U	K.EYEATLEECCAKDDPHACYSTVFDK.L + Glycat
3838	351 - 375	1067.4374	3199.2903	3199.2944	-1.28	1	41	8.9e-005	1	U	K.EYEATLEECCAKDDPHACYSTVFDK.L + Glycat
3839	351 - 375	800.8318	3199.2981	3199.2944	1.14	1	25	0.0031	1	U	K.EYEATLEECCAKDDPHACYSTVFDK.L + Glycat
1816	363 - 375	777.8291	1553.6436	1553.6457	-1.31	0	37	0.00019	1	U	K.DDPHACYSTVFDK.L
1817	363 - 375	777.8293	1553.6440	1553.6457	-1.07	0	45	2.9e-005	1	U	K.DDPHACYSTVFDK.L
1818	363 - 375	777.8297	1553.6447	1553.6457	-0.60	0	43	4.8e-005	1	U	K.DDPHACYSTVFDK.L
1819	363 - 375	777.8297	1553.6447	1553.6457	-0.60	0	45	3e-005	1	U	K.DDPHACYSTVFDK.L
1820	363 - 375	518.8891	1553.6455	1553.6457	-0.13	0	39	0.00013	1	U	K.DDPHACYSTVFDK.L
1821	363 - 375	518.8892	1553.6457	1553.6457	-0.015	0	57	1.9e-006	1	U	K.DDPHACYSTVFDK.L
1822	363 - 375	518.8894	1553.6464	1553.6457	0.45	0	49	1.3e-005	1	U	K.DDPHACYSTVFDK.L
1823	363 - 375	518.8895	1553.6468	1553.6457	0.70	0	48	1.6e-005	1	U	K.DDPHACYSTVFDK.L
1824	363 - 375	518.8896	1553.6469	1553.6457	0.81	0	65	3.4e-007	1	U	K.DDPHACYSTVFDK.L
2871	363 - 377	979.4451	1956.8757	1956.8775	-0.92	1	20	0.0089	1	U	K.DDPHACYSTVFDKLK.H + Glycation (K)
2872	363 - 377	979.4451	1956.8757	1956.8775	-0.92	1	21	0.0076	1	U	K.DDPHACYSTVFDKLK.H + Glycation (K)
2873	363 - 377	653.2993	1956.8761	1956.8775	-0.71	1	37	0.00022	1	U	K.DDPHACYSTVFDKLK.H + Glycation (K)
2874	363 - 377	653.2994	1956.8765	1956.8775	-0.52	1	22	0.0061	1	U	K.DDPHACYSTVFDKLK.H + Glycation (K)
2875	363 - 377	653.2994	1956.8765	1956.8775	-0.52	1	31	0.00073	1	U	K.DDPHACYSTVFDKLK.H + Glycation (K)
2876	363 - 377	490.2265	1956.8768	1956.8775	-0.38	1	15	0.029	1	U	K.DDPHACYSTVFDKLK.H + Glycation (K)
2877	363 - 377	653.2996	1956.8770	1956.8775	-0.25	1	24	0.0039	1	U	K.DDPHACYSTVFDKLK.H + Glycation (K)
2878	363 - 377	653.2996	1956.8770	1956.8775	-0.25	1	26	0.0026	1	U	K.DDPHACYSTVFDKLK.H + Glycation (K)
2879	363 - 377	653.2996	1956.8770	1956.8775	-0.25	1	29	0.0013	1	U	K.DDPHACYSTVFDKLK.H + Glycation (K)
2883	363 - 377	490.2271	1956.8791	1956.8775	0.81	1	22	0.0069	1	U	K.DDPHACYSTVFDKLK.H + Glycation (K)
3897	363 - 388	852.4136	3405.6253	3405.6286	-0.97	2	31	0.00083	1	U	K.DDPHACYSTVFDKLKHLVDEPQNLIK.Q + 2 Gly
3898	363 - 388	682.1326	3405.6268	3405.6286	-0.54	2	28	0.0016	1	U	K.DDPHACYSTVFDKLKHLVDEPQNLIK.Q + 2 Gly
3899	363 - 388	1136.2172	3405.6297	3405.6286	0.30	2	24	0.004	1	U	K.DDPHACYSTVFDKLKHLVDEPQNLIK.Q + 2 Gly
3900	363 - 388	1136.2172	3405.6298	3405.6286	0.34	2	19	0.012	1	U	K.DDPHACYSTVFDKLKHLVDEPQNLIK.Q + 2 Gly
3901	363 - 388	852.4148	3405.6302	3405.6286	0.47	2	29	0.0012	1	U	K.DDPHACYSTVFDKLKHLVDEPQNLIK.Q + 2 Gly
3902	363 - 388	1136.2174	3405.6303	3405.6286	0.48	2	32	0.00065	1	U	K.DDPHACYSTVFDKLKHLVDEPQNLIK.Q + 2 Gly
3903	363 - 388	852.4150	3405.6308	3405.6286	0.65	2	16	0.027	1	U	K.DDPHACYSTVFDKLKHLVDEPQNLIK.Q + 2 Gly
3905	363 - 388	682.1357	3405.6420	3405.6286	3.94	2	36	0.00025	1	U	K.DDPHACYSTVFDKLKHLVDEPQNLIK.Q + 2 Gly
4208	363 - 396	924.4276	4617.1014	4617.1051	-0.81	3	26	0.0028	1	U	K.DDPHACYSTVFDKLKHLVDEPQNLIKQNCDQFEK.
4209	363 - 396	924.4280	4617.1034	4617.1051	-0.36	3	28	0.0016	1	U	K.DDPHACYSTVFDKLKHLVDEPQNLIKQNCDQFEK.
4210	363 - 396	1155.2840	4617.1069	4617.1051	0.38	3	24	0.0041	1	U	K.DDPHACYSTVFDKLKHLVDEPQNLIKQNCDQFEK.
2191	376 - 388	854.9766	1707.9387	1707.9407	-1.16	1	34	0.00036	1	U	K.LKHLVDEPQNLIK.Q + Glycation (K)
2192	376 - 388	854.9768	1707.9391	1707.9407	-0.94	1	38	0.00016	1	U	K.LKHLVDEPQNLIK.Q + Glycation (K)
2193	376 - 388	427.9923	1707.9403	1707.9407	-0.24	1	27	0.0021	1	U	K.LKHLVDEPQNLIK.Q + Glycation (K)
2194	376 - 388	427.9924	1707.9404	1707.9407	-0.17	1	28	0.0016	1	U	K.LKHLVDEPQNLIK.Q + Glycation (K)
2195	376 - 388	570.3207	1707.9404	1707.9407	-0.17	1	31	0.00079	1	U	K.LKHLVDEPQNLIK.Q + Glycation (K)

Query	Start – End	Observed	Mr(expt)	Mr(calc)	ppm	M	Score	Expect	Rank	U	Peptide
2196	376 – 388	570.3207	1707.9404	1707.9407	-0.17	1	30	0.001	1	U	K.LKHLVDEPQNLIK.Q + Glycation (K)
2197	376 – 388	570.3208	1707.9406	1707.9407	-0.060	1	38	0.00017	1	U	K.LKHLVDEPQNLIK.Q + Glycation (K)
2198	376 – 388	427.9924	1707.9406	1707.9407	-0.034	1	26	0.0028	1	U	K.LKHLVDEPQNLIK.Q + Glycation (K)
2199	376 – 388	570.3209	1707.9408	1707.9407	0.046	1	35	0.00029	1	U	K.LKHLVDEPQNLIK.Q + Glycation (K)
2200	376 – 388	427.9925	1707.9409	1707.9407	0.11	1	19	0.013	1	U	K.LKHLVDEPQNLIK.Q + Glycation (K)
3692	376 – 396	974.1446	2919.4121	2919.4171	-1.72	2	39	0.00011	1	U	K.LKHLVDEPQNLIKQNCDQFEK.L + 2 Glycatio
3693	376 – 396	974.1450	2919.4132	2919.4171	-1.34	2	55	3.4e-006	1	U	K.LKHLVDEPQNLIKQNCDQFEK.L + 2 Glycatio
3694	376 – 396	730.8608	2919.4140	2919.4171	-1.07	2	28	0.0017	1	U	K.LKHLVDEPQNLIKQNCDQFEK.L + 2 Glycatio
3695	376 – 396	974.1453	2919.4141	2919.4171	-1.03	2	63	5.2e-007	1	U	K.LKHLVDEPQNLIKQNCDQFEK.L + 2 Glycatio
3696	376 – 396	730.8610	2919.4147	2919.4171	-0.83	2	36	0.00028	1	U	K.LKHLVDEPQNLIKQNCDQFEK.L + 2 Glycatio
3697	376 – 396	730.8611	2919.4152	2919.4171	-0.66	2	45	3.3e-005	1	U	K.LKHLVDEPQNLIKQNCDQFEK.L + 2 Glycatio
3698	376 – 396	730.8613	2919.4162	2919.4171	-0.32	2	47	2.2e-005	1	U	K.LKHLVDEPQNLIKQNCDQFEK.L + 2 Glycatio
3699	376 – 396	584.8906	2919.4164	2919.4171	-0.25	2	35	0.00034	1	U	K.LKHLVDEPQNLIKQNCDQFEK.L + 2 Glycatio
3700	376 – 396	584.8906	2919.4168	2919.4171	-0.13	2	28	0.0016	1	U	K.LKHLVDEPQNLIKQNCDQFEK.L + 2 Glycatio
3701	376 – 396	584.8906	2919.4168	2919.4171	-0.13	2	28	0.0017	1	U	K.LKHLVDEPQNLIKQNCDQFEK.L + 2 Glycatio
4197	376 – 409	909.4576	4542.2515	4542.2476	0.87	3	93	5.1e-010	1	U	K.LKHLVDEPQNLIKQNCDQFEKLGEYGFQNALIVR.
1152	378 – 388	653.3614	1304.7082	1304.7088	-0.46	0	47	2.1e-005	1	U	K.HLVDEPQNLIK.Q
1153	378 – 388	653.3614	1304.7083	1304.7088	-0.37	0	26	0.0025	1	U	K.HLVDEPQNLIK.Q
1154	378 – 388	653.3614	1304.7083	1304.7088	-0.37	0	45	2.9e-005	1	U	K.HLVDEPQNLIK.Q
1155	378 – 388	653.3614	1304.7083	1304.7088	-0.37	0	46	2.4e-005	1	U	K.HLVDEPQNLIK.Q
1156	378 – 388	435.9101	1304.7086	1304.7088	-0.21	0	48	1.5e-005	1	U	K.HLVDEPQNLIK.Q
1157	378 – 388	653.3616	1304.7086	1304.7088	-0.19	0	46	2.4e-005	1	U	K.HLVDEPQNLIK.Q
1158	378 – 388	435.9102	1304.7087	1304.7088	-0.069	0	48	1.5e-005	1	U	K.HLVDEPQNLIK.Q
1159	378 – 388	435.9102	1304.7088	1304.7088	0	0	29	0.0014	1	U	K.HLVDEPQNLIK.Q
3464	378 – 396	839.7344	2516.1813	2516.1853	-1.59	1	73	5.1e-008	1	U	K.HLVDEPQNLIKQNCDQFEK.L + Glycation (K)
3465	378 – 396	1259.0983	2516.1820	2516.1853	-1.32	1	78	1.5e-008	1	U	K.HLVDEPQNLIKQNCDQFEK.L + Glycation (K)
3466	378 – 396	839.7350	2516.1831	2516.1853	-0.86	1	61	7.2e-007	1	U	K.HLVDEPQNLIKQNCDQFEK.L + Glycation (K)
3467	378 – 396	630.0531	2516.1833	2516.1853	-0.80	1	49	1.4e-005	1	U	K.HLVDEPQNLIKQNCDQFEK.L + Glycation (K)
3468	378 – 396	839.7351	2516.1833	2516.1853	-0.79	1	60	1e-006	1	U	K.HLVDEPQNLIKQNCDQFEK.L + Glycation (K)
3470	378 – 396	630.0535	2516.1850	2516.1853	-0.11	1	60	1.1e-006	1	U	K.HLVDEPQNLIKQNCDQFEK.L + Glycation (K)
3471	378 – 396	630.0535	2516.1850	2516.1853	-0.11	1	57	1.9e-006	1	U	K.HLVDEPQNLIKQNCDQFEK.L + Glycation (K)
3472	378 – 396	839.7357	2516.1852	2516.1853	-0.060	1	59	1.1e-006	1	U	K.HLVDEPQNLIKQNCDQFEK.L + Glycation (K)
3473	378 – 396	630.0536	2516.1855	2516.1853	0.077	1	48	1.6e-005	1	U	K.HLVDEPQNLIKQNCDQFEK.L + Glycation (K)
3474	378 – 396	1259.1001	2516.1856	2516.1853	0.14	1	71	8.9e-008	1	U	K.HLVDEPQNLIKQNCDQFEK.L + Glycation (K)
4126	378 – 409	828.8098	4139.0127	4139.0157	-0.74	2	53	4.8e-006	1	U	K.HLVDEPQNLIKQNCDQFEKLGEYGFQNALIVR.Y
4127	378 – 409	1035.7605	4139.0129	4139.0157	-0.68	2	79	1.1e-008	1	U	K.HLVDEPQNLIKQNCDQFEKLGEYGFQNALIVR.Y
4128	378 – 409	1035.7612	4139.0158	4139.0157	0.024	2	73	5.2e-008	1	U	K.HLVDEPQNLIKQNCDQFEKLGEYGFQNALIVR.Y
4129	378 – 409	828.8112	4139.0194	4139.0157	0.89	2	64	3.7e-007	1	U	K.HLVDEPQNLIKQNCDQFEKLGEYGFQNALIVR.Y
3561	389 – 409	897.7604	2690.2595	2690.2646	-1.91	1	119	1.3e-012	1	U	K.QNCDQFEKLGEYGFQNALIVR.Y + Glycation (
3562	389 – 409	897.7613	2690.2620	2690.2646	-0.96	1	96	2.4e-010	1	U	K.QNCDQFEKLGEYGFQNALIVR.Y + Glycation (
3563	389 – 409	897.7613	2690.2622	2690.2646	-0.89	1	108	1.5e-011	1	U	K.QNCDQFEKLGEYGFQNALIVR.Y + Glycation (
1614	397 – 409	740.4009	1478.7872	1478.7881	-0.63	0	97	1.9e-010	1	U	K.LGEYGFQNALIVR.Y
1615	397 – 409	740.4009	1478.7872	1478.7881	-0.63	0	97	1.9e-010	1	U	K.LGEYGFQNALIVR.Y
1616	397 – 409	740.4012	1478.7878	1478.7881	-0.23	0	97	2e-010	1	U	K.LGEYGFQNALIVR.Y
1617	397 – 409	740.4015	1478.7884	1478.7881	0.19	0	97	1.9e-010	1	U	K.LGEYGFQNALIVR.Y
1995	413 – 427	820.4716	1638.9287	1638.9305	-1.09	1	117	2.2e-012	1	U	R.KVPQVSTPTLVEVSR.S
1996	413 – 427	820.4717	1638.9289	1638.9305	-0.95	1	117	2.1e-012	1	U	R.KVPQVSTPTLVEVSR.S
1997	413 – 427	820.4718	1638.9290	1638.9305	-0.87	1	97	2e-010	1	U	R.KVPQVSTPTLVEVSR.S
1998	413 – 427	820.4718	1638.9290	1638.9305	-0.87	1	117	2.2e-012	1	U	R.KVPQVSTPTLVEVSR.S
1999	413 – 427	820.4719	1638.9292	1638.9305	-0.80	1	117	2.2e-012	1	U	R.KVPQVSTPTLVEVSR.S
2000	413 – 427	547.3171	1638.9296	1638.9305	-0.54	1	53	5.3e-006	1	U	R.KVPQVSTPTLVEVSR.S
2001	413 – 427	547.3173	1638.9300	1638.9305	-0.32	1	79	1.2e-008	1	U	R.KVPQVSTPTLVEVSR.S
2002	413 – 427	547.3173	1638.9300	1638.9305	-0.32	1	76	2.3e-008	1	U	R.KVPQVSTPTLVEVSR.S
2003	413 – 427	820.4723	1638.9300	1638.9305	-0.28	1	117	2.1e-012	1	U	R.KVPQVSTPTLVEVSR.S
2004	413 – 427	547.3174	1638.9303	1638.9305	-0.10	1	85	3.3e-009	1	U	R.KVPQVSTPTLVEVSR.S
2005	413 – 427	547.3174	1638.9305	1638.9305	0.0067	1	78	1.7e-008	1	U	R.KVPQVSTPTLVEVSR.S
2006	413 – 427	547.3178	1638.9316	1638.9305	0.68	1	72	5.7e-008	1	U	R.KVPQVSTPTLVEVSR.S
2007	413 – 427	547.3182	1638.9327	1638.9305	1.36	1	84	4.2e-009	1	U	R.KVPQVSTPTLVEVSR.S
2008	413 – 427	547.3190	1638.9351	1638.9305	2.81	1	86	2.8e-009	1	U	R.KVPQVSTPTLVEVSR.S
2009	413 – 427	547.3194	1638.9364	1638.9305	3.59	1	72	5.7e-008	1	U	R.KVPQVSTPTLVEVSR.S
2507	413 – 427	901.4981	1800.9817	1800.9833	-0.91	1	94	3.7e-010	1	U	R.KVPQVSTPTLVEVSR.S + Glycation (K)
2508	413 – 427	901.4983	1800.9821	1800.9833	-0.64	1	82	6.7e-009	1	U	R.KVPQVSTPTLVEVSR.S + Glycation (K)
2509	413 – 427	601.3347	1800.9822	1800.9833	-0.64	1	62	6.9e-007	1	U	R.KVPQVSTPTLVEVSR.S + Glycation (K)
2510	413 – 427	601.3348	1800.9825	1800.9833	-0.44	1	63	5e-007	1	U	R.KVPQVSTPTLVEVSR.S + Glycation (K)
2511	413 – 427	601.3350	1800.9832	1800.9833	-0.042	1	61	7.6e-007	1	U	R.KVPQVSTPTLVEVSR.S + Glycation (K)
2512	413 – 427	601.3351	1800.9834	1800.9833	0.058	1	60	9.1e-007	1	U	R.KVPQVSTPTLVEVSR.S + Glycation (K)
1705	414 – 427	756.4244	1510.8342	1510.8355	-0.87	0	104	3.6e-011	1	U	K.VPQVSTPTLVEVSR.S
1706	414 – 427	756.4244	1510.8343	1510.8355	-0.79	0	104	3.7e-011	1	U	K.VPQVSTPTLVEVSR.S
1707	414 – 427	756.4245	1510.8344	1510.8355	-0.71	0	119	1.3e-012	1	U	K.VPQVSTPTLVEVSR.S
1708	414 – 427	756.4246	1510.8346	1510.8355	-0.63	0	104	3.7e-011	1	U	K.VPQVSTPTLVEVSR.S
1709	414 – 427	504.6190	1510.8352	1510.8355	-0.19	0	56	2.7e-006	1	U	K.VPQVSTPTLVEVSR.S
1710	414 – 427	504.6191	1510.8356	1510.8355	0.044	0	49	1.4e-005	1	U	K.VPQVSTPTLVEVSR.S
2228	445 – 458	862.9198	1723.8250	1723.8273	-1.31	0	99	1.4e-010	1	U	R.MPCTEDYLSLILNR.L
2229	445 – 458	862.9199	1723.8253	1723.8273	-1.17	0	81	8.8e-009	1	U	R.MPCTEDYLSLILNR.L
2230	445 – 458	862.9199	1723.8253	1723.8273	-1.17	0	98	1.6e-010	1	U	R.MPCTEDYLSLILNR.L
2231	445 – 458	862.9203	1723.8260	1723.8273	-0.74	0	99	1.4e-010	1	U	R.MPCTEDYLSLILNR.L
2232	445 – 458	575.6163	1723.8270	1723.8273	-0.19	0	36	0.00028	1	U	R.MPCTEDYLSLILNR.L
2233	445 – 458	575.6163	1723.8272	1723.8273	-0.081	0	36	0.00028	1	U	R.MPCTEDYLSLILNR.L
2234	445 – 458	862.9209	1723.8272	1723.8273	-0.031	0	118	1.8e-012	1	U	R.MPCTEDYLSLILNR.L
2235	445 – 458	575.6165	1723.8277	1723.8273	0.25	0	36	0.00026	1	U	R.MPCTEDYLSLILNR.L
350	459 – 465	300.1650	897.4733	897.4742	-1.03	0	15	0.031	1	U	R.LCVLHEK.T
352	459 – 465	300.1652	897.4737	897.4742	-0.63	0	14	0.038	1	U	R.LCVLHEK.T
353	459 – 465	449.7441	897.4737	897.4742	-0.61	0	24	0.004	1	U	R.LCVLHEK.T
354	459 – 465	449.7441	897.4737	897.4742	-0.61	0	24	0.0039	1	U	R.LCVLHEK.T
356	459 – 465	300.1652	897.4737	897.4742	-0.52	0	15	0.028	1	U	R.LCVLHEK.T
357	459 – 465	449.7442	897.4738	897.4742	-0.41	0	24	0.0039	1	U	R.LCVLHEK.T
358	459 – 465	300.1653	897.4739	897.4742	-0.32	0	15	0.029	1	U	R.LCVLHEK.T
359	459 – 465	449.7443	897.4740	897.4742	-0.27	0	24	0.0039	1	U	R.LCVLHEK.T

Query	Start - End	Observed	Mr(expt)	Mr(calc)	ppm	M	Score	Expect	Rank	U	Peptide
361	459 - 465	449.7444	897.4742	897.4742	-0.0056	0	24	0.0039	1	U	R.LCVLHEK.T
362	459 - 465	449.7444	897.4742	897.4742	-0.0056	0	24	0.0039	1	U	R.LCVLHEK.T
2183	459 - 471	851.4389	1700.8632	1700.8655	-1.31	1	53	4.5e-006	1	U	R.LCVLHEKTPVSEK.V + Glycation (K)
2184	459 - 471	426.2235	1700.8647	1700.8655	-0.46	1	24	0.0041	1	U	R.LCVLHEKTPVSEK.V + Glycation (K)
2185	459 - 471	567.9623	1700.8650	1700.8655	-0.27	1	41	8.6e-005	1	U	R.LCVLHEKTPVSEK.V + Glycation (K)
2186	459 - 471	426.2235	1700.8651	1700.8655	-0.25	1	30	0.001	1	U	R.LCVLHEKTPVSEK.V + Glycation (K)
2187	459 - 471	567.9623	1700.8652	1700.8655	-0.17	1	36	0.00028	1	U	R.LCVLHEKTPVSEK.V + Glycation (K)
2188	459 - 471	426.2236	1700.8653	1700.8655	-0.11	1	24	0.0036	1	U	R.LCVLHEKTPVSEK.V + Glycation (K)
2189	459 - 471	567.9625	1700.8656	1700.8655	0.043	1	34	0.0004	1	U	R.LCVLHEKTPVSEK.V + Glycation (K)
3386	466 - 483	811.3881	2431.1424	2431.1458	-1.43	2	57	2e-006	1	U	K.TPVSEKVTKCCTESLVNR.R + 2 Glycation (K
3387	466 - 483	811.3883	2431.1431	2431.1458	-1.12	2	61	7.7e-007	1	U	K.TPVSEKVTKCCTESLVNR.R + 2 Glycation (K
3388	466 - 483	811.3884	2431.1435	2431.1458	-0.97	2	57	1.8e-006	1	U	K.TPVSEKVTKCCTESLVNR.R + 2 Glycation (K
3389	466 - 483	608.7935	2431.1447	2431.1458	-0.45	2	62	6.1e-007	1	U	K.TPVSEKVTKCCTESLVNR.R + 2 Glycation (K
3390	466 - 483	608.7936	2431.1452	2431.1458	-0.25	2	66	2.8e-007	1	U	K.TPVSEKVTKCCTESLVNR.R + 2 Glycation (K
3391	466 - 483	608.7938	2431.1459	2431.1458	0.044	2	74	3.8e-008	1	U	K.TPVSEKVTKCCTESLVNR.R + 2 Glycation (K
1958	472 - 483	814.8837	1627.7529	1627.7546	-1.02	1	69	1.3e-007	1	U	K.VTKCCTESLVNR.R + Glycation (K)
1959	472 - 483	814.8837	1627.7529	1627.7546	-1.02	1	69	1.2e-007	1	U	K.VTKCCTESLVNR.R + Glycation (K)
1960	472 - 483	543.5916	1627.7530	1627.7546	-0.96	1	76	2.3e-008	1	U	K.VTKCCTESLVNR.R + Glycation (K)
1961	472 - 483	814.8839	1627.7531	1627.7546	-0.87	1	59	1.3e-006	1	U	K.VTKCCTESLVNR.R + Glycation (K)
1962	472 - 483	814.8839	1627.7531	1627.7546	-0.87	1	54	4.4e-006	1	U	K.VTKCCTESLVNR.R + Glycation (K)
1963	472 - 483	814.8840	1627.7534	1627.7546	-0.72	1	53	5.3e-006	1	U	K.VTKCCTESLVNR.R + Glycation (K)
1964	472 - 483	543.5920	1627.7541	1627.7546	-0.28	1	71	7.7e-008	1	U	K.VTKCCTESLVNR.R + Glycation (K)
1965	472 - 483	543.5920	1627.7541	1627.7546	-0.28	1	57	2.1e-006	1	U	K.VTKCCTESLVNR.R + Glycation (K)
1966	472 - 483	543.5921	1627.7545	1627.7546	-0.055	1	59	1.3e-006	1	U	K.VTKCCTESLVNR.R + Glycation (K)
1967	472 - 483	543.5922	1627.7548	1627.7546	0.17	1	71	7.9e-008	1	U	K.VTKCCTESLVNR.R + Glycation (K)
1968	472 - 483	543.5925	1627.7556	1627.7546	0.63	1	66	2.2e-007	1	U	K.VTKCCTESLVNR.R + Glycation (K)
1969	472 - 483	543.5925	1627.7558	1627.7546	0.74	1	59	1.3e-006	1	U	K.VTKCCTESLVNR.R + Glycation (K)
868	475 - 483	569.7519	1137.4893	1137.4907	-1.17	0	75	3.4e-008	1	U	K.CCTESLVNR.R
869	475 - 483	569.7525	1137.4904	1137.4907	-0.20	0	63	5.4e-007	1	U	K.CCTESLVNR.R
870	475 - 483	569.7526	1137.4907	1137.4907	0.0097	0	63	4.6e-007	1	U	K.CCTESLVNR.R
871	475 - 483	569.7527	1137.4908	1137.4907	0.13	0	61	8.9e-007	1	U	K.CCTESLVNR.R
872	475 - 483	569.7531	1137.4915	1137.4907	0.77	0	63	4.8e-007	1	U	K.CCTESLVNR.R
873	475 - 483	569.7532	1137.4918	1137.4907	0.98	0	63	4.9e-007	1	U	K.CCTESLVNR.R
874	475 - 483	569.7543	1137.4941	1137.4907	3.02	0	69	1.4e-007	1	U	K.CCTESLVNR.R
2663	484 - 499	940.9628	1879.9110	1879.9138	-1.51	1	43	4.9e-005	1	U	R.RPCFSALTPDETYVPK.A
2664	484 - 499	940.9631	1879.9116	1879.9138	-1.19	1	57	2.2e-006	1	U	R.RPCFSALTPDETYVPK.A
2665	484 - 499	940.9631	1879.9116	1879.9138	-1.19	1	48	1.6e-005	1	U	R.RPCFSALTPDETYVPK.A
2666	484 - 499	940.9631	1879.9117	1879.9138	-1.13	1	47	2e-005	1	U	R.RPCFSALTPDETYVPK.A
2667	484 - 499	940.9632	1879.9118	1879.9138	-1.06	1	48	1.8e-005	1	U	R.RPCFSALTPDETYVPK.A
2668	484 - 499	627.6448	1879.9127	1879.9138	-0.61	1	27	0.002	1	U	R.RPCFSALTPDETYVPK.A
2669	484 - 499	627.6449	1879.9129	1879.9138	-0.51	1	28	0.0015	1	U	R.RPCFSALTPDETYVPK.A
2670	484 - 499	627.6449	1879.9129	1879.9138	-0.51	1	37	0.00022	1	U	R.RPCFSALTPDETYVPK.A
2671	484 - 499	627.6449	1879.9129	1879.9138	-0.51	1	34	0.00044	1	U	R.RPCFSALTPDETYVPK.A
2672	484 - 499	627.6450	1879.9132	1879.9138	-0.32	1	44	3.7e-005	1	U	R.RPCFSALTPDETYVPK.A
2673	484 - 499	627.6451	1879.9136	1879.9138	-0.13	1	25	0.0035	1	U	R.RPCFSALTPDETYVPK.A
2674	484 - 499	627.6452	1879.9138	1879.9138	-0.033	1	34	0.00044	1	U	R.RPCFSALTPDETYVPK.A
2675	484 - 499	627.6452	1879.9138	1879.9138	-0.033	1	37	0.00021	1	U	R.RPCFSALTPDETYVPK.A
2676	484 - 499	627.6455	1879.9147	1879.9138	0.46	1	33	0.00051	1	U	R.RPCFSALTPDETYVPK.A
2677	484 - 499	627.6461	1879.9164	1879.9138	1.34	1	35	0.0003	1	U	R.RPCFSALTPDETYVPK.A
3523	484 - 504	878.4186	2632.2341	2632.2367	-0.98	2	43	5.1e-005	1	U	R.RPCFSALTPDETYVPKAFDEK.L + Glycation (
3524	484 - 504	878.4189	2632.2348	2632.2367	-0.71	2	43	5.2e-005	1	U	R.RPCFSALTPDETYVPKAFDEK.L + Glycation (
3525	484 - 504	659.0661	2632.2353	2632.2367	-0.53	2	36	0.00027	1	U	R.RPCFSALTPDETYVPKAFDEK.L + Glycation (
3526	484 - 504	878.4191	2632.2354	2632.2367	-0.49	2	46	2.6e-005	1	U	R.RPCFSALTPDETYVPKAFDEK.L + Glycation (
3527	484 - 504	659.0662	2632.2355	2632.2367	-0.43	2	31	0.00072	1	U	R.RPCFSALTPDETYVPKAFDEK.L + Glycation (
3528	484 - 504	659.0662	2632.2358	2632.2367	-0.34	2	42	5.8e-005	1	U	R.RPCFSALTPDETYVPKAFDEK.L + Glycation (
3529	484 - 504	659.0664	2632.2365	2632.2367	-0.054	2	43	5.4e-005	1	U	R.RPCFSALTPDETYVPKAFDEK.L + Glycation (
4252	484 - 520	937.6427	4683.1771	4683.1925	-3.28	3	18	0.015	1	U	R.RPCFSALTPDETYVPKAFDEKLFTFHADICTLPDT
2227	485 - 499	862.9135	1723.8125	1723.8127	-0.15	0	30	0.001	1	U	R.PCFSALTPDETYVPK.A
3539	500 - 520	887.4185	2659.2336	2659.2364	-1.06	1	74	3.7e-008	1	U	K.AFDEKLFTFHADICTLPDTEK.Q + Glycation (
3540	500 - 520	1330.6245	2659.2345	2659.2364	-0.71	1	58	1.6e-006	1	U	K.AFDEKLFTFHADICTLPDTEK.Q + Glycation (
3541	500 - 520	887.4189	2659.2348	2659.2364	-0.58	1	63	5.1e-007	1	U	K.AFDEKLFTFHADICTLPDTEK.Q + Glycation (
3542	500 - 520	665.8161	2659.2353	2659.2364	-0.40	1	54	3.6e-006	1	U	K.AFDEKLFTFHADICTLPDTEK.Q + Glycation (
3543	500 - 520	665.8164	2659.2365	2659.2364	0.067	1	52	6.2e-006	1	U	K.AFDEKLFTFHADICTLPDTEK.Q + Glycation (
3544	500 - 520	887.4211	2659.2416	2659.2364	1.97	1	52	5.7e-006	1	U	K.AFDEKLFTFHADICTLPDTEK.Q + Glycation (
2745	505 - 520	954.4628	1906.9110	1906.9135	-1.32	0	86	2.3e-009	1	U	K.LFTFHADICTLPDTEK.Q
2746	505 - 520	954.4634	1906.9123	1906.9135	-0.62	0	78	1.6e-008	1	U	K.LFTFHADICTLPDTEK.Q
2747	505 - 520	636.6450	1906.9131	1906.9135	-0.24	0	77	1.8e-008	1	U	K.LFTFHADICTLPDTEK.Q
2748	505 - 520	636.6450	1906.9131	1906.9135	-0.24	0	74	3.7e-008	1	U	K.LFTFHADICTLPDTEK.Q
2749	505 - 520	636.6450	1906.9132	1906.9135	-0.15	0	73	4.7e-008	1	U	K.LFTFHADICTLPDTEK.Q
2750	505 - 520	954.4639	1906.9133	1906.9135	-0.11	0	59	1.3e-006	1	U	K.LFTFHADICTLPDTEK.Q
2751	505 - 520	636.6452	1906.9138	1906.9135	0.14	0	78	1.8e-008	1	U	K.LFTFHADICTLPDTEK.Q
2752	505 - 520	954.4643	1906.9140	1906.9135	0.27	0	78	1.7e-008	1	U	K.LFTFHADICTLPDTEK.Q
2753	505 - 520	636.6454	1906.9143	1906.9135	0.43	0	50	9.1e-006	1	U	K.LFTFHADICTLPDTEK.Q
3405	505 - 523	813.7404	2438.1993	2438.2039	-1.92	1	40	0.00011	1	U	K.LFTFHADICTLPDTEKQIK.K + Glycation (K)
3406	505 - 523	813.7404	2438.1993	2438.2039	-1.92	1	36	0.00026	1	U	K.LFTFHADICTLPDTEKQIK.K + Glycation (K)
3407	505 - 523	813.7415	2438.2026	2438.2039	-0.57	1	38	0.00017	1	U	K.LFTFHADICTLPDTEKQIK.K + Glycation (K)
2578	521 - 533	918.5314	1835.0483	1835.0503	-1.07	2	35	0.00032	1	U	K.QIKKQTALVELLK.H + 2 Glycation (K)
2580	521 - 533	612.6903	1835.0491	1835.0503	-0.64	2	28	0.0014	1	U	K.QIKKQTALVELLK.H + 2 Glycation (K)
2581	521 - 533	612.6905	1835.0496	1835.0503	-0.35	2	22	0.0065	1	U	K.QIKKQTALVELLK.H + 2 Glycation (K)
2582	521 - 533	612.6906	1835.0498	1835.0503	-0.25	2	28	0.0016	1	U	K.QIKKQTALVELLK.H + 2 Glycation (K)
2583	521 - 533	459.7698	1835.0500	1835.0503	-0.14	2	36	0.00023	1	U	K.QIKKQTALVELLK.H + 2 Glycation (K)
2584	521 - 533	459.7699	1835.0504	1835.0503	0.053	2	37	0.00021	1	U	K.QIKKQTALVELLK.H + 2 Glycation (K)
2585	521 - 533	459.7699	1835.0505	1835.0503	0.12	2	23	0.0055	1	U	K.QIKKQTALVELLK.H + 2 Glycation (K)
886	524 - 533	571.8605	1141.7065	1141.7070	-0.46	1	61	7.3e-007	1	U	K.KQTALVELLK.H
887	524 - 533	381.5762	1141.7067	1141.7070	-0.31	1	36	0.00023	1	U	K.KQTALVELLK.H
888	524 - 533	381.5762	1141.7067	1141.7070	-0.31	1	42	6.1e-005	1	U	K.KQTALVELLK.H
889	524 - 533	381.5762	1141.7068	1141.7070	-0.23	1	36	0.00023	1	U	K.KQTALVELLK.H
890	524 - 533	571.8607	1141.7069	1141.7070	-0.13	1	62	6.9e-007	1	U	K.KQTALVELLK.H

Query	Start - End	Observed	Mr(expt)	Mr(calc)	ppm	M	Score	Expect	Rank	U	Peptide
891	524 - 533	571.8608	1141.7070	1141.7070	-0.020	1	44	3.6e-005	1	U	K.KQTALVELLK.H
892	524 - 533	571.8608	1141.7071	1141.7070	0.085	1	52	5.8e-006	1	U	K.KQTALVELLK.H
1141	524 - 533	652.8867	1303.7589	1303.7599	-0.74	1	42	6.2e-005	1	U	K.KQTALVELLK.H + Glycation (K)
1142	524 - 533	435.5938	1303.7594	1303.7599	-0.33	1	47	2.2e-005	1	U	K.KQTALVELLK.H + Glycation (K)
1143	524 - 533	652.8870	1303.7595	1303.7599	-0.28	1	38	0.00015	1	U	K.KQTALVELLK.H + Glycation (K)
1144	524 - 533	652.8871	1303.7596	1303.7599	-0.19	1	47	2.2e-005	1	U	K.KQTALVELLK.H + Glycation (K)
1145	524 - 533	652.8871	1303.7596	1303.7599	-0.19	1	46	2.2e-005	1	U	K.KQTALVELLK.H + Glycation (K)
1146	524 - 533	652.8872	1303.7599	1303.7599	0.0084	1	32	0.0007	1	U	K.KQTALVELLK.H + Glycation (K)
1147	524 - 533	435.5939	1303.7599	1303.7599	0.012	1	52	6.2e-006	1	U	K.KQTALVELLK.H + Glycation (K)
1148	524 - 533	435.5940	1303.7601	1303.7599	0.22	1	46	2.4e-005	1	U	K.KQTALVELLK.H + Glycation (K)
1149	524 - 533	435.5941	1303.7604	1303.7599	0.45	1	47	2.1e-005	1	U	K.KQTALVELLK.H + Glycation (K)
1150	524 - 533	652.8875	1303.7605	1303.7599	0.47	1	46	2.3e-005	1	U	K.KQTALVELLK.H + Glycation (K)
1151	524 - 533	435.5942	1303.7607	1303.7599	0.66	1	47	2.1e-005	1	U	K.KQTALVELLK.H + Glycation (K)
573	525 - 533	507.8134	1013.6123	1013.6121	0.21	0	53	4.6e-006	1	U	K.QTALVELLK.H
574	525 - 533	507.8134	1013.6123	1013.6121	0.27	0	45	2.8e-005	1	U	K.QTALVELLK.H
575	525 - 533	507.8135	1013.6124	1013.6121	0.33	0	53	4.6e-006	1	U	K.QTALVELLK.H
576	525 - 533	507.8135	1013.6125	1013.6121	0.45	0	43	4.9e-005	1	U	K.QTALVELLK.H
3738	534 - 556	1005.1718	3012.4934	3012.5002	-2.23	3	69	1.3e-007	1	U	K.HKPKATEEQLKTVMENFVAFVDK.C + 2 Glycat
3739	534 - 556	754.1310	3012.4948	3012.5002	-1.77	3	74	4.1e-008	1	U	K.HKPKATEEQLKTVMENFVAFVDK.C + 2 Glycat
3740	534 - 556	1005.1725	3012.4956	3012.5002	-1.51	3	64	4.3e-007	1	U	K.HKPKATEEQLKTVMENFVAFVDK.C + 2 Glycat
3741	534 - 556	754.1323	3012.4999	3012.5002	-0.072	3	31	0.00078	1	U	K.HKPKATEEQLKTVMENFVAFVDK.C + 2 Glycat
3742	534 - 556	1005.1743	3012.5010	3012.5002	0.27	3	79	1.3e-008	1	U	K.HKPKATEEQLKTVMENFVAFVDK.C + 2 Glycat
3743	534 - 556	754.1339	3012.5063	3012.5002	2.04	3	15	0.035	1	U	K.HKPKATEEQLKTVMENFVAFVDK.C + 2 Glycat
3313	538 - 556	787.7220	2360.1443	2360.1457	-0.60	1	65	2.9e-007	1	U	K.ATEEQLKTVMENFVAFVDK.C + Glycation (K)
3314	538 - 556	787.7221	2360.1445	2360.1457	-0.52	1	86	2.8e-009	1	U	K.ATEEQLKTVMENFVAFVDK.C + Glycation (K)
3315	538 - 556	1181.0797	2360.1449	2360.1457	-0.37	1	57	2.1e-006	1	U	K.ATEEQLKTVMENFVAFVDK.C + Glycation (K)
3316	538 - 556	787.7224	2360.1452	2360.1457	-0.22	1	60	1e-006	1	U	K.ATEEQLKTVMENFVAFVDK.C + Glycation (K)
3317	538 - 556	787.7224	2360.1453	2360.1457	-0.17	1	60	1.1e-006	1	U	K.ATEEQLKTVMENFVAFVDK.C + Glycation (K)
3318	538 - 556	787.7224	2360.1454	2360.1457	-0.14	1	111	8.9e-012	1	U	K.ATEEQLKTVMENFVAFVDK.C + Glycation (K)
3319	538 - 556	1181.0812	2360.1478	2360.1457	0.88	1	81	8.3e-009	1	U	K.ATEEQLKTVMENFVAFVDK.C + Glycation (K)
1358	545 - 556	700.3494	1398.6842	1398.6853	-0.83	0	53	5.3e-006	1	U	K.TVMENFVAFVDK.C
1359	545 - 556	700.3496	1398.6845	1398.6853	-0.57	0	49	1.3e-005	1	U	K.TVMENFVAFVDK.C
1360	545 - 556	700.3496	1398.6845	1398.6853	-0.57	0	71	8.4e-008	1	U	K.TVMENFVAFVDK.C
1361	545 - 556	700.3520	1398.6895	1398.6853	3.00	0	67	2.1e-007	1	U	K.TVMENFVAFVDK.C
3343	545 - 563	794.6812	2381.0216	2381.0226	-0.39	1	53	5e-006	1	U	K.TVMENFVAFVDKCCAADDK.E + Glycation (K)
2813	557 - 573	964.4020	1926.7894	1926.7910	-0.83	1	127	2e-013	1	U	K.CCAADDKEACFAVEGPK.L
2814	557 - 573	964.4020	1926.7895	1926.7910	-0.77	1	130	9.8e-014	1	U	K.CCAADDKEACFAVEGPK.L
2815	557 - 573	643.2708	1926.7906	1926.7910	-0.21	1	109	1.2e-011	1	U	K.CCAADDKEACFAVEGPK.L
2816	557 - 573	643.2710	1926.7912	1926.7910	0.081	1	103	4.5e-011	1	U	K.CCAADDKEACFAVEGPK.L
2817	557 - 573	643.2711	1926.7915	1926.7910	0.27	1	103	4.5e-011	1	U	K.CCAADDKEACFAVEGPK.L
3055	557 - 573	697.2880	2088.8422	2088.8438	-0.77	1	56	2.3e-006	1	U	K.CCAADDKEACFAVEGPK.L + Glycation (K)
3056	557 - 573	697.2883	2088.8432	2088.8438	-0.33	1	55	3.3e-006	1	U	K.CCAADDKEACFAVEGPK.L + Glycation (K)
3768	557 - 583	1025.1429	3072.4070	3072.4090	-0.65	2	69	1.1e-007	1	U	K.CCAADDKEACFAVEGPKLVVSTQTALA.- + Glyc
3769	557 - 583	1025.1440	3072.4103	3072.4090	0.42	2	80	9.2e-009	1	U	K.CCAADDKEACFAVEGPKLVVSTQTALA.- + Glyc
3770	557 - 583	1025.1445	3072.4118	3072.4090	0.90	2	55	2.9e-006	1	U	K.CCAADDKEACFAVEGPKLVVSTQTALA.- + Glyc
3771	557 - 583	1025.1450	3072.4132	3072.4090	1.38	2	57	2.1e-006	1	U	K.CCAADDKEACFAVEGPKLVVSTQTALA.- + Glyc
3845	557 - 583	1079.1619	3234.4638	3234.4618	0.61	2	13	0.048	1	U	K.CCAADDKEACFAVEGPKLVVSTQTALA.- + 2 Gl
805	564 - 573	554.2603	1106.5061	1106.5066	-0.52	0	86	2.6e-009	1	U	K.EACFAVEGPK.L
806	564 - 573	554.2604	1106.5063	1106.5066	-0.28	0	86	2.7e-009	1	U	K.EACFAVEGPK.L
807	564 - 573	554.2605	1106.5064	1106.5066	-0.17	0	86	2.6e-009	1	U	K.EACFAVEGPK.L
808	564 - 573	554.2607	1106.5069	1106.5066	0.26	0	86	2.7e-009	1	U	K.EACFAVEGPK.L
809	564 - 573	554.2610	1106.5075	1106.5066	0.82	0	80	9.3e-009	1	U	K.EACFAVEGPK.L
3183	564 - 583	1127.0691	2252.1236	2252.1246	-0.44	1	30	0.00089	1	U	K.EACFAVEGPKLVVSTQTALA.- + Glycation (K
3184	564 - 583	751.7161	2252.1266	2252.1246	0.86	1	13	0.046	1	U	K.EACFAVEGPKLVVSTQTALA.- + Glycation (K
3185	564 - 583	1127.0712	2252.1279	2252.1246	1.45	1	25	0.0032	1	U	K.EACFAVEGPKLVVSTQTALA.- + Glycation (K
545	574 - 583	501.7950	1001.5755	1001.5757	-0.19	0	17	0.021	1	U	K.LVVSTQTALA.-
546	574 - 583	501.7950	1001.5755	1001.5757	-0.19	0	14	0.044	1	U	K.LVVSTQTALA.-
547	574 - 583	501.7951	1001.5756	1001.5757	-0.13	0	18	0.016	1	U	K.LVVSTQTALA.-
548	574 - 583	501.7951	1001.5756	1001.5757	-0.066	0	18	0.015	1	U	K.LVVSTQTALA.-
549	574 - 583	501.7951	1001.5757	1001.5757	0.014	0	13	0.049	1	U	K.LVVSTQTALA.-
550	574 - 583	501.7951	1001.5757	1001.5757	0.014	0	17	0.021	1	U	K.LVVSTQTALA.-

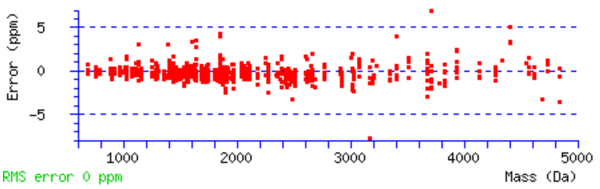

Mascot: http://www.matrixscience.com/

Appendix 4. Mascot search results of chymotrypsin digestion for BSA-2

MATRIX SCIENCE **MASCOT Search Results**

Protein View: >gi|00000

BSA

**Database:**	BSA
**Score:**	10558
**Monoisotopic mass (M_r):**	68386
**Calculated pI:**	5.60

Sequence similarity is available as **an NCBI BLAST search of >gi|00000 against nr**.

Search parameters

**MS data file:**	P2019021810.mgf
**Enzyme:**	Chymotrypsin: cuts C-term side of FLWY unless next residue is P.
**Fixed modifications:**	**Carbamidomethyl (C)**
**Variable modifications:**	**Glycation (K)**, **Glycation (Protein N-term)**

Protein sequence coverage: 71%

Matched peptides shown in ***bold red***.

1 **DTHKSEIAHR** **FKDLGEEHFK** **GLVL**IAFSQY **LQQCPFDEHV** **KLVNELTEFA**
51 **KTCVADESHA** **GCEKSLHTLF** **GDELCKVASL** **RETYGDMADC** **CEKQEPERNE**
101 **CFLSHKDDSP** **DLPKLKPDPN** **TLCDEFKADE** **KKFW**GKYLYE IARRHPY**FYA**
151 **PELL**YYANKY **NGVFQECCQA** **EDKGACL**LPK IETMREKVLA SSARQRLRCA
201 SIQKFGERAL KAWSVARL**SQ** **KFPKAEFVEV** **TKLVTDLTKV** **HKECCHGDLL**
251 **ECADDRADLA** **KYICDNQDTI** **SSKLKECCDK** **PLLEKSHCIA** **EVEKDAIPEN**
301 **LPPLTADFAE** **DKDVCKNYQE** **AKDAFLGSFL** **Y**EYSRRHPEY AVSVL**LRLAK**
351 **EYEATLEECC** **AKDDPHACYS** **TVFDKLKHLV** **DEPQNLIKQN** **CDQFEKLGEY**
401 GFQNALIVRY TRKVPQVSTP TL**VEVSRSL**G KVGTRCCTKP ESERMPCTED
451 YLSLIL**NRLC** **VL**HEKTPVSE KVTKCCTESL VNRRPCF**SAL** **TPDETYVPKA**
501 **FDEKLFTFHA** **DICTLPDTEK** **QIKKQTALVE** **LL**KHKPKATE EQL**KTVMENF**
551 **VAFVDKCCAA** **DDKEACFAVE** **GPKLVVSTQT** **ALA**

Unformatted sequence string: **583 residues** (for pasting into other applications).

Sort by ◉ residue number ○ increasing mass ○ decreasing mass
Show ◉ matched peptides only ○ predicted peptides also

Query	Start – End	Observed	Mr(expt)	Mr(calc)	ppm	M	Score	Expect	Rank	U	Peptide
1209	1 – 11	335.9228	1339.6622	1339.6633	-0.83	0	33	0.0005	1	U	-.DTHKSEIAHRF.K
1210	1 – 11	335.9229	1339.6624	1339.6633	-0.65	0	38	0.00017	1	U	-.DTHKSEIAHRF.K
1211	1 – 11	335.9229	1339.6625	1339.6633	-0.56	0	33	0.00053	1	U	-.DTHKSEIAHRF.K
1212	1 – 11	447.5615	1339.6626	1339.6633	-0.55	0	37	0.0002	1	U	-.DTHKSEIAHRF.K
1213	1 – 11	670.8386	1339.6627	1339.6633	-0.44	0	43	4.5e-005	1	U	-.DTHKSEIAHRF.K
1214	1 – 11	335.9230	1339.6628	1339.6633	-0.38	0	30	0.001	1	U	-.DTHKSEIAHRF.K
1215	1 – 11	335.9230	1339.6629	1339.6633	-0.29	0	30	0.00099	1	U	-.DTHKSEIAHRF.K
1216	1 – 11	447.5616	1339.6629	1339.6633	-0.28	0	37	0.00019	1	U	-.DTHKSEIAHRF.K
1217	1 – 11	447.5616	1339.6629	1339.6633	-0.28	0	41	8.7e-005	1	U	-.DTHKSEIAHRF.K
1218	1 – 11	447.5616	1339.6630	1339.6633	-0.21	0	39	0.00012	1	U	-.DTHKSEIAHRF.K
1219	1 – 11	335.9230	1339.6630	1339.6633	-0.20	0	36	0.00025	1	U	-.DTHKSEIAHRF.K
1220	1 – 11	335.9230	1339.6630	1339.6633	-0.20	0	44	3.9e-005	1	U	-.DTHKSEIAHRF.K
1221	1 – 11	447.5617	1339.6632	1339.6633	-0.054	0	39	0.00012	1	U	-.DTHKSEIAHRF.K
1222	1 – 11	447.5635	1339.6687	1339.6633	4.04	0	15	0.031	1	U	-.DTHKSEIAHRF.K
1576	1 – 11	376.4361	1501.7153	1501.7161	-0.54	0	41	7.2e-005	1	U	-.DTHKSEIAHRF.K + Glycation (Protein N-t
1577	1 – 11	376.4361	1501.7154	1501.7161	-0.46	0	30	0.001	1	U	-.DTHKSEIAHRF.K + Glycation (K)
1578	1 – 11	376.4361	1501.7154	1501.7161	-0.46	0	40	9.6e-005	1	U	-.DTHKSEIAHRF.K + Glycation (Protein N-t
1579	1 – 11	376.4361	1501.7154	1501.7161	-0.46	0	39	0.00014	1	U	-.DTHKSEIAHRF.K + Glycation (Protein N-t
1580	1 – 11	501.5791	1501.7155	1501.7161	-0.42	0	43	5.2e-005	1	U	-.DTHKSEIAHRF.K + Glycation (Protein N-t
1581	1 – 11	501.5791	1501.7155	1501.7161	-0.42	0	40	9.9e-005	1	U	-.DTHKSEIAHRF.K + Glycation (Protein N-t
1582	1 – 11	376.4362	1501.7155	1501.7161	-0.38	0	43	5e-005	1	U	-.DTHKSEIAHRF.K + Glycation (Protein N-t
1583	1 – 11	376.4362	1501.7155	1501.7161	-0.38	0	43	4.7e-005	1	U	-.DTHKSEIAHRF.K + Glycation (Protein N-t
1584	1 – 11	376.4362	1501.7155	1501.7161	-0.38	0	40	0.00011	1	U	-.DTHKSEIAHRF.K + Glycation (Protein N-t
1585	1 – 11	501.5791	1501.7156	1501.7161	-0.36	0	33	0.00051	1	U	-.DTHKSEIAHRF.K + Glycation (Protein N-t
1586	1 – 11	376.4362	1501.7158	1501.7161	-0.22	0	47	2.1e-005	1	U	-.DTHKSEIAHRF.K + Glycation (Protein N-t
1587	1 – 11	501.5792	1501.7158	1501.7161	-0.19	0	45	3.2e-005	1	U	-.DTHKSEIAHRF.K + Glycation (Protein N-t
1588	1 – 11	501.5792	1501.7158	1501.7161	-0.19	0	55	3.3e-006	1	U	-.DTHKSEIAHRF.K + Glycation (Protein N-t
1589	1 – 11	501.5793	1501.7161	1501.7161	0.015	0	40	0.0001	1	U	-.DTHKSEIAHRF.K + Glycation (Protein N-t
1590	1 – 11	501.5793	1501.7162	1501.7161	0.075	0	40	0.0001	1	U	-.DTHKSEIAHRF.K + Glycation (Protein N-t
1855	1 – 11	555.5968	1663.7686	1663.7689	-0.22	0	27	0.0021	1	U	-.DTHKSEIAHRF.K + Glycation (K); Glycat:
1856	1 – 11	416.9494	1663.7686	1663.7689	-0.19	0	43	4.5e-005	1	U	-.DTHKSEIAHRF.K + Glycation (K); Glycat:
1857	1 – 11	416.9495	1663.7687	1663.7689	-0.12	0	30	0.001	1	U	-.DTHKSEIAHRF.K + Glycation (K); Glycat:
1858	1 – 11	416.9495	1663.7690	1663.7689	0.024	0	28	0.0014	1	U	-.DTHKSEIAHRF.K + Glycation (K); Glycat:
1859	1 – 11	416.9495	1663.7690	1663.7689	0.024	0	33	0.0005	1	U	-.DTHKSEIAHRF.K + Glycation (K); Glycat:
1860	1 – 11	416.9495	1663.7691	1663.7689	0.096	0	22	0.0067	1	U	-.DTHKSEIAHRF.K + Glycation (K); Glycat:
1861	1 – 11	416.9497	1663.7696	1663.7689	0.41	0	33	0.00052	1	U	-.DTHKSEIAHRF.K + Glycation (K); Glycat:
1862	1 – 11	555.5973	1663.7700	1663.7689	0.66	0	41	8.1e-005	1	U	-.DTHKSEIAHRF.K + Glycation (K); Glycat:
553	12 – 19	325.4906	973.4500	973.4505	-0.54	1	23	0.0051	1	U	F.KDLGEEHF.K
554	12 – 19	325.4907	973.4502	973.4505	-0.36	1	17	0.018	1	U	F.KDLGEEHF.K
555	12 – 19	487.7324	973.4502	973.4505	-0.28	1	24	0.0041	1	U	F.KDLGEEHF.K
556	12 – 19	325.4907	973.4502	973.4505	-0.27	1	21	0.0089	1	U	F.KDLGEEHF.K
557	12 – 19	487.7326	973.4506	973.4505	0.090	1	23	0.0047	1	U	F.KDLGEEHF.K

Query	Start - End	Observed	Mr(expt)	Mr(calc)	ppm	M	Score	Expect	Rank	U	Peptide
558	12 - 19	487.7326	973.4507	973.4505	0.23	1	31	0.00079	1	U	F.KDLGEEHF.K
559	12 - 19	487.7327	973.4508	973.4505	0.30	1	30	0.0011	1	U	F.KDLGEEHF.K
839	12 - 19	568.7584	1135.5023	1135.5033	-0.91	1	21	0.0086	1	U	F.KDLGEEHF.K + Glycation (K)
840	12 - 19	379.5082	1135.5029	1135.5033	-0.38	1	14	0.043	1	U	F.KDLGEEHF.K + Glycation (K)
841	12 - 19	379.5083	1135.5030	1135.5033	-0.30	1	13	0.045	1	U	F.KDLGEEHF.K + Glycation (K)
842	12 - 19	379.5083	1135.5030	1135.5033	-0.30	1	15	0.031	1	U	F.KDLGEEHF.K + Glycation (K)
845	12 - 19	568.7589	1135.5031	1135.5033	-0.16	1	25	0.0032	1	U	F.KDLGEEHF.K + Glycation (K)
846	12 - 19	568.7589	1135.5031	1135.5033	-0.16	1	33	0.00048	1	U	F.KDLGEEHF.K + Glycation (K)
847	12 - 19	568.7589	1135.5031	1135.5033	-0.16	1	33	0.00047	1	U	F.KDLGEEHF.K + Glycation (K)
848	12 - 19	568.7589	1135.5033	1135.5033	-0.051	1	26	0.0026	1	U	F.KDLGEEHF.K + Glycation (K)
849	12 - 19	568.7592	1135.5039	1135.5033	0.49	1	27	0.0019	1	U	F.KDLGEEHF.K + Glycation (K)
1085	12 - 22	636.8324	1271.6502	1271.6510	-0.58	2	30	0.0011	1	U	F.KDLGEEHFKGL.V
1086	12 - 22	424.8907	1271.6503	1271.6510	-0.51	2	17	0.02	1	U	F.KDLGEEHFKGL.V
1087	12 - 22	636.8325	1271.6505	1271.6510	-0.39	2	29	0.0014	1	U	F.KDLGEEHFKGL.V
1088	12 - 22	636.8325	1271.6505	1271.6510	-0.39	2	27	0.002	1	U	F.KDLGEEHFKGL.V
1089	12 - 22	424.8908	1271.6505	1271.6510	-0.37	2	22	0.0059	1	U	F.KDLGEEHFKGL.V
1090	12 - 22	424.8908	1271.6505	1271.6510	-0.37	2	19	0.014	1	U	F.KDLGEEHFKGL.V
1091	12 - 22	424.8910	1271.6511	1271.6510	0.052	2	22	0.007	1	U	F.KDLGEEHFKGL.V
1092	12 - 22	424.8912	1271.6517	1271.6510	0.55	2	19	0.013	1	U	F.KDLGEEHFKGL.V
1093	12 - 22	636.8336	1271.6527	1271.6510	1.34	2	28	0.0017	1	U	F.KDLGEEHFKGL.V
1443	12 - 22	717.8585	1433.7024	1433.7038	-1.01	2	22	0.0065	1	U	F.KDLGEEHFKGL.V + Glycation (K)
1444	12 - 22	717.8585	1433.7025	1433.7038	-0.92	2	26	0.0023	1	U	F.KDLGEEHFKGL.V + Glycation (K)
1445	12 - 22	717.8586	1433.7027	1433.7038	-0.76	2	22	0.0057	1	U	F.KDLGEEHFKGL.V + Glycation (K)
1446	12 - 22	478.9083	1433.7030	1433.7038	-0.54	2	34	0.00037	1	U	F.KDLGEEHFKGL.V + Glycation (K)
1447	12 - 22	717.8589	1433.7032	1433.7038	-0.41	2	26	0.0024	1	U	F.KDLGEEHFKGL.V + Glycation (K)
1448	12 - 22	478.9084	1433.7034	1433.7038	-0.27	2	39	0.00011	1	U	F.KDLGEEHFKGL.V + Glycation (K)
1449	12 - 22	717.8590	1433.7035	1433.7038	-0.24	2	25	0.0029	1	U	F.KDLGEEHFKGL.V + Glycation (K)
1450	12 - 22	478.9085	1433.7036	1433.7038	-0.14	2	31	0.00086	1	U	F.KDLGEEHFKGL.V + Glycation (K)
1451	12 - 22	478.9085	1433.7038	1433.7038	-0.014	2	34	0.0004	1	U	F.KDLGEEHFKGL.V + Glycation (K)
1452	12 - 22	478.9086	1433.7039	1433.7038	0.049	2	33	0.0005	1	U	F.KDLGEEHFKGL.V + Glycation (K)
1455	12 - 22	478.9087	1433.7042	1433.7038	0.24	2	37	0.00021	1	U	F.KDLGEEHFKGL.V + Glycation (K)
1456	12 - 22	478.9087	1433.7042	1433.7038	0.24	2	40	0.0001	1	U	F.KDLGEEHFKGL.V + Glycation (K)
1781	12 - 22	532.9263	1595.7570	1595.7566	0.22	2	17	0.02	1	U	F.KDLGEEHFKGL.V + 2 Glycation (K)
1782	12 - 22	532.9263	1595.7570	1595.7566	0.22	2	27	0.002	1	U	F.KDLGEEHFKGL.V + 2 Glycation (K)
1783	12 - 22	532.9263	1595.7572	1595.7566	0.33	2	24	0.004	1	U	F.KDLGEEHFKGL.V + 2 Glycation (K)
1784	12 - 22	532.9264	1595.7573	1595.7566	0.44	2	25	0.0032	1	U	F.KDLGEEHFKGL.V + 2 Glycation (K)
1785	12 - 22	532.9265	1595.7575	1595.7566	0.55	2	23	0.0046	1	U	F.KDLGEEHFKGL.V + 2 Glycation (K)
1544	12 - 24	495.6084	1483.8033	1483.8035	-0.12	3	17	0.021	1	U	F.KDLGEEHFKGLVL.I
14	20 - 24	265.1890	528.3634	528.3635	-0.30	1	13	0.048	1	U	F.KGLVL.I
15	20 - 24	265.1890	528.3634	528.3635	-0.30	1	13	0.049	1	U	F.KGLVL.I
16	20 - 24	265.1890	528.3635	528.3635	-0.032	1	15	0.034	1	U	F.KGLVL.I
252	31 - 36	396.6890	791.3634	791.3636	-0.31	1	35	0.00033	1	U	Y.LQQCPF.D
253	31 - 36	396.6891	791.3636	791.3636	0.014	1	30	0.001	1	U	Y.LQQCPF.D
254	31 - 36	396.6895	791.3643	791.3636	0.92	1	19	0.012	1	U	Y.LQQCPF.D
1614	31 - 42	757.3762	1512.7378	1512.7395	-1.15	2	24	0.0039	1	U	Y.LQQCPFDEHVKL.V
1615	31 - 42	757.3765	1512.7384	1512.7395	-0.75	2	29	0.0013	1	U	Y.LQQCPFDEHVKL.V
1616	31 - 42	505.2534	1512.7385	1512.7395	-0.65	2	13	0.049	1	U	Y.LQQCPFDEHVKL.V
1617	31 - 42	505.2536	1512.7390	1512.7395	-0.35	2	23	0.005	1	U	Y.LQQCPFDEHVKL.V
1618	31 - 42	505.2538	1512.7395	1512.7395	0.0066	2	20	0.01	1	U	Y.LQQCPFDEHVKL.V
1872	31 - 42	838.4025	1674.7904	1674.7923	-1.16	2	25	0.003	1	U	Y.LQQCPFDEHVKL.V + Glycation (K)
1873	31 - 42	838.4026	1674.7906	1674.7923	-1.02	2	27	0.0021	1	U	Y.LQQCPFDEHVKL.V + Glycation (K)
1875	31 - 42	559.2714	1674.7924	1674.7923	0.062	2	14	0.042	1	U	Y.LQQCPFDEHVKL.V + Glycation (K)
2719	31 - 49	1173.5767	2345.1388	2345.1362	1.10	4	14	0.038	1	U	Y.LQQCPFDEHVKLVNELTEF.A
2908	31 - 49	1254.6023	2507.1900	2507.1890	0.41	4	22	0.0057	1	U	Y.LQQCPFDEHVKLVNELTEF.A + Glycation (K)
1732	37 - 49	786.8981	1571.7817	1571.7831	-0.90	2	47	2e-005	1	U	F.DEHVKLVNELTEF.A
1733	37 - 49	786.8987	1571.7829	1571.7831	-0.13	2	47	2e-005	1	U	F.DEHVKLVNELTEF.A
1736	37 - 49	524.9351	1571.7835	1571.7831	0.26	2	64	4e-007	1	U	F.DEHVKLVNELTEF.A
1737	37 - 49	524.9351	1571.7835	1571.7831	0.26	2	83	5.5e-009	1	U	F.DEHVKLVNELTEF.A
1739	37 - 49	524.9352	1571.7839	1571.7831	0.49	2	41	7.9e-005	1	U	F.DEHVKLVNELTEF.A
1958	37 - 49	867.9242	1733.8338	1733.8360	-1.23	2	64	4.4e-007	1	U	F.DEHVKLVNELTEF.A + Glycation (K)
1959	37 - 49	867.9244	1733.8343	1733.8360	-0.94	2	52	5.6e-006	1	U	F.DEHVKLVNELTEF.A + Glycation (K)
1960	37 - 49	867.9246	1733.8346	1733.8360	-0.80	2	45	3.1e-005	1	U	F.DEHVKLVNELTEF.A + Glycation (K)
1961	37 - 49	578.9524	1733.8353	1733.8360	-0.35	2	62	6.4e-007	1	U	F.DEHVKLVNELTEF.A + Glycation (K)
1962	37 - 49	578.9525	1733.8357	1733.8360	-0.14	2	74	3.8e-008	1	U	F.DEHVKLVNELTEF.A + Glycation (K)
2292	50 - 66	675.6343	2023.8810	2023.8827	-0.81	0	35	0.0003	1	U	F.AKTCVADESHAGCEKSL.H + Glycation (K)
2990	50 - 70	672.0557	2684.1935	2684.1946	-0.38	2	18	0.016	1	U	F.AKTCVADESHAGCEKSLHTLF.G + 2 Glycatio
2991	50 - 70	672.0560	2684.1948	2684.1946	0.079	2	14	0.043	1	U	F.AKTCVADESHAGCEKSLHTLF.G + 2 Glycatio
779	71 - 80	546.2737	1090.5328	1090.5329	-0.040	1	47	1.9e-005	1	U	F.GDELCKVASL.R
780	71 - 80	546.2739	1090.5332	1090.5329	0.29	1	45	3.1e-005	1	U	F.GDELCKVASL.R
1050	71 - 80	627.2998	1252.5850	1252.5857	-0.50	1	20	0.0095	1	U	F.GDELCKVASL.R + Glycation (K)
98	75 - 80	339.1863	676.3580	676.3578	0.33	0	24	0.0042	1	U	L.CKVASL.R
3064	81 - 102	706.7819	2823.0986	2823.0993	-0.25	1	62	6.4e-007	1	U	L.RETYGDMADCCEKQEPERNECF.L
2605	85 - 102	758.9547	2273.8423	2273.8446	-1.00	0	55	3.3e-006	1	U	Y.GDMADCCEKQEPERNECF.L
2606	85 - 102	758.9551	2273.8436	2273.8446	-0.43	0	59	1.4e-006	1	U	Y.GDMADCCEKQEPERNECF.L
2607	85 - 102	1137.9293	2273.8441	2273.8446	-0.21	0	64	4.2e-007	1	U	Y.GDMADCCEKQEPERNECF.L
2608	85 - 102	1137.9293	2273.8441	2273.8446	-0.21	0	79	1.3e-008	1	U	Y.GDMADCCEKQEPERNECF.L
2609	85 - 102	758.9553	2273.8441	2273.8446	-0.19	0	59	1.4e-006	1	U	Y.GDMADCCEKQEPERNECF.L
2610	85 - 102	758.9567	2273.8484	2273.8446	1.67	0	46	2.7e-005	1	U	Y.GDMADCCEKQEPERNECF.L
2843	85 - 102	812.9724	2435.8954	2435.8974	-0.82	0	40	0.00011	1	U	Y.GDMADCCEKQEPERNECF.L + Glycation (K)
2844	85 - 102	812.9725	2435.8958	2435.8974	-0.67	0	32	0.00065	1	U	Y.GDMADCCEKQEPERNECF.L + Glycation (K)
2845	85 - 102	812.9727	2435.8962	2435.8974	-0.51	0	39	0.00012	1	U	Y.GDMADCCEKQEPERNECF.L + Glycation (K)
2782	85 - 103	796.6494	2386.9264	2386.9286	-0.94	1	21	0.0087	1	U	Y.GDMADCCEKQEPERNECFL.S
2546	103 - 122	744.0600	2229.1582	2229.1641	-2.66	2	35	0.00035	1	U	F.LSHKDDSPDLPKLKPDPNTL.C
2547	103 - 122	744.0617	2229.1633	2229.1641	-0.35	2	35	0.00029	1	U	F.LSHKDDSPDLPKLKPDPNTL.C
2548	103 - 122	558.2983	2229.1643	2229.1641	0.073	2	18	0.015	1	U	F.LSHKDDSPDLPKLKPDPNTL.C
2549	103 - 122	558.2984	2229.1645	2229.1641	0.18	2	19	0.014	1	U	F.LSHKDDSPDLPKLKPDPNTL.C
2550	103 - 122	558.2987	2229.1657	2229.1641	0.74	2	19	0.012	1	U	F.LSHKDDSPDLPKLKPDPNTL.C
3025	103 - 126	927.7832	2780.3278	2780.3327	-1.77	3	45	3.3e-005	1	U	F.LSHKDDSPDLPKLKPDPNTLCDEF.K

Query	Start – End	Observed	Mr(expt)	Mr(calc)	ppm	M	Score	Expect	Rank	U	Peptide
3026	103 – 126	927.7839	2780.3300	2780.3327	-0.98	3	34	0.00044	1	U	F.LSHKDDSPDLPKLKPDPNTLCDEF.K
3027	103 – 126	557.0733	2780.3301	2780.3327	-0.93	3	20	0.011	1	U	F.LSHKDDSPDLPKLKPDPNTLCDEF.K
3029	103 – 126	696.0899	2780.3305	2780.3327	-0.79	3	21	0.0076	1	U	F.LSHKDDSPDLPKLKPDPNTLCDEF.K
3030	103 – 126	696.0900	2780.3308	2780.3327	-0.69	3	17	0.019	1	U	F.LSHKDDSPDLPKLKPDPNTLCDEF.K
3031	103 – 126	927.7842	2780.3309	2780.3327	-0.65	3	28	0.0015	1	U	F.LSHKDDSPDLPKLKPDPNTLCDEF.K
3034	103 – 126	696.0901	2780.3315	2780.3327	-0.43	3	23	0.0046	1	U	F.LSHKDDSPDLPKLKPDPNTLCDEF.K
3036	103 – 126	696.0905	2780.3327	2780.3327	-0.0025	3	23	0.0045	1	U	F.LSHKDDSPDLPKLKPDPNTLCDEF.K
3037	103 – 126	927.7849	2780.3327	2780.3327	0.0068	3	34	0.00044	1	U	F.LSHKDDSPDLPKLKPDPNTLCDEF.K
3038	103 – 126	557.0739	2780.3332	2780.3327	0.17	3	24	0.0037	1	U	F.LSHKDDSPDLPKLKPDPNTLCDEF.K
3039	103 – 126	927.7850	2780.3333	2780.3327	0.20	3	36	0.00027	1	U	F.LSHKDDSPDLPKLKPDPNTLCDEF.K
3142	103 – 126	981.8004	2942.3794	2942.3855	-2.07	3	31	0.00075	1	U	F.LSHKDDSPDLPKLKPDPNTLCDEF.K + Glycati
3143	103 – 126	589.4836	2942.3818	2942.3855	-1.26	3	18	0.016	1	U	F.LSHKDDSPDLPKLKPDPNTLCDEF.K + Glycati
3144	103 – 126	981.8015	2942.3825	2942.3855	-1.02	3	21	0.008	1	U	F.LSHKDDSPDLPKLKPDPNTLCDEF.K + Glycati
3145	103 – 126	736.6030	2942.3828	2942.3855	-0.94	3	32	0.00062	1	U	F.LSHKDDSPDLPKLKPDPNTLCDEF.K + Glycati
3146	103 – 126	981.8016	2942.3831	2942.3855	-0.83	3	33	0.00048	1	U	F.LSHKDDSPDLPKLKPDPNTLCDEF.K + Glycati
3147	103 – 126	736.6031	2942.3833	2942.3855	-0.77	3	24	0.0044	1	U	F.LSHKDDSPDLPKLKPDPNTLCDEF.K + Glycati
3148	103 – 126	981.8017	2942.3833	2942.3855	-0.77	3	33	0.00045	1	U	F.LSHKDDSPDLPKLKPDPNTLCDEF.K + Glycati
3149	103 – 126	981.8018	2942.3835	2942.3855	-0.71	3	46	2.4e-005	1	U	F.LSHKDDSPDLPKLKPDPNTLCDEF.K + Glycati
3150	103 – 126	736.6035	2942.3850	2942.3855	-0.19	3	21	0.0074	1	U	F.LSHKDDSPDLPKLKPDPNTLCDEF.K + Glycati
3151	103 – 126	736.6036	2942.3855	2942.3855	-0.025	3	38	0.00017	1	U	F.LSHKDDSPDLPKLKPDPNTLCDEF.K + Glycati
3152	103 – 126	589.4844	2942.3855	2942.3855	-0.0031	3	20	0.011	1	U	F.LSHKDDSPDLPKLKPDPNTLCDEF.K + Glycati
3153	103 – 126	589.4844	2942.3858	2942.3855	0.099	3	25	0.0035	1	U	F.LSHKDDSPDLPKLKPDPNTLCDEF.K + Glycati
3154	103 – 126	736.6038	2942.3862	2942.3855	0.22	3	29	0.0013	1	U	F.LSHKDDSPDLPKLKPDPNTLCDEF.K + Glycati
3155	103 – 126	736.6039	2942.3864	2942.3855	0.30	3	29	0.0013	1	U	F.LSHKDDSPDLPKLKPDPNTLCDEF.K + Glycati
3156	103 – 126	736.6049	2942.3903	2942.3855	1.63	3	21	0.0081	1	U	F.LSHKDDSPDLPKLKPDPNTLCDEF.K + Glycati
3157	103 – 126	1472.2029	2942.3912	2942.3855	1.93	3	29	0.0013	1	U	F.LSHKDDSPDLPKLKPDPNTLCDEF.K + Glycati
3289	103 – 126	777.1160	3104.4350	3104.4384	-1.08	3	19	0.011	1	U	F.LSHKDDSPDLPKLKPDPNTLCDEF.K + 2 Glyca
3290	103 – 126	777.1161	3104.4355	3104.4384	-0.92	3	30	0.001	1	U	F.LSHKDDSPDLPKLKPDPNTLCDEF.K + 2 Glyca
3291	103 – 126	1035.8195	3104.4366	3104.4384	-0.58	3	29	0.0013	1	U	F.LSHKDDSPDLPKLKPDPNTLCDEF.K + 2 Glyca
3292	103 – 126	1035.8197	3104.4373	3104.4384	-0.35	3	22	0.0068	1	U	F.LSHKDDSPDLPKLKPDPNTLCDEF.K + 2 Glyca
3293	103 – 126	621.8948	3104.4375	3104.4384	-0.27	3	31	0.00087	1	U	F.LSHKDDSPDLPKLKPDPNTLCDEF.K + 2 Glyca
3294	103 – 126	621.8950	3104.4387	3104.4384	0.12	3	26	0.0026	1	U	F.LSHKDDSPDLPKLKPDPNTLCDEF.K + 2 Glyca
3295	103 – 126	777.1177	3104.4419	3104.4384	1.13	3	27	0.0021	1	U	F.LSHKDDSPDLPKLKPDPNTLCDEF.K + 2 Glyca
1232	104 – 115	451.2331	1350.6774	1350.6779	-0.43	0	17	0.021	1	U	L.SHKDDSPDLPKL.K
1233	104 – 115	451.2331	1350.6774	1350.6779	-0.36	0	15	0.033	1	U	L.SHKDDSPDLPKL.K
1234	104 – 115	338.6766	1350.6775	1350.6779	-0.35	0	14	0.039	1	U	L.SHKDDSPDLPKL.K
1235	104 – 115	451.2332	1350.6776	1350.6779	-0.23	0	15	0.033	1	U	L.SHKDDSPDLPKL.K
2382	104 – 122	706.3664	2116.0773	2116.0800	-1.27	1	30	0.001	1	U	L.SHKDDSPDLPKLKPDPNTL.C
2383	104 – 122	530.0274	2116.0805	2116.0800	0.22	1	38	0.00018	1	U	L.SHKDDSPDLPKLKPDPNTL.C
2980	104 – 126	890.0883	2667.2430	2667.2486	-2.13	2	34	0.0004	1	U	L.SHKDDSPDLPKLKPDPNTLCDEF.K
2981	104 – 126	667.8184	2667.2446	2667.2486	-1.52	2	28	0.0016	1	U	L.SHKDDSPDLPKLKPDPNTLCDEF.K
2982	104 – 126	890.0895	2667.2468	2667.2486	-0.69	2	40	9e-005	1	U	L.SHKDDSPDLPKLKPDPNTLCDEF.K
2983	104 – 126	667.8191	2667.2473	2667.2486	-0.52	2	32	0.00069	1	U	L.SHKDDSPDLPKLKPDPNTLCDEF.K
2984	104 – 126	890.0898	2667.2477	2667.2486	-0.36	2	30	0.00096	1	U	L.SHKDDSPDLPKLKPDPNTLCDEF.K
2985	104 – 126	667.8195	2667.2490	2667.2486	0.13	2	37	0.00018	1	U	L.SHKDDSPDLPKLKPDPNTLCDEF.K
2986	104 – 126	667.8196	2667.2495	2667.2486	0.31	2	37	0.00019	1	U	L.SHKDDSPDLPKLKPDPNTLCDEF.K
2987	104 – 126	890.0906	2667.2501	2667.2486	0.54	2	37	0.00021	1	U	L.SHKDDSPDLPKLKPDPNTLCDEF.K
3066	104 – 126	944.1069	2829.2990	2829.3015	-0.89	2	26	0.0022	1	U	L.SHKDDSPDLPKLKPDPNTLCDEF.K + Glycatio
3067	104 – 126	708.3321	2829.2993	2829.3015	-0.78	2	31	0.00077	1	U	L.SHKDDSPDLPKLKPDPNTLCDEF.K + Glycatio
3068	104 – 126	944.1071	2829.2995	2829.3015	-0.68	2	30	0.00093	1	U	L.SHKDDSPDLPKLKPDPNTLCDEF.K + Glycatio
3069	104 – 126	708.3323	2829.3000	2829.3015	-0.51	2	33	0.00046	1	U	L.SHKDDSPDLPKLKPDPNTLCDEF.K + Glycatio
3070	104 – 126	708.3323	2829.3003	2829.3015	-0.43	2	25	0.0035	1	U	L.SHKDDSPDLPKLKPDPNTLCDEF.K + Glycatio
3071	104 – 126	944.1076	2829.3010	2829.3015	-0.18	2	28	0.0016	1	U	L.SHKDDSPDLPKLKPDPNTLCDEF.K + Glycatio
3072	104 – 126	708.3328	2829.3019	2829.3015	0.17	2	24	0.0044	1	U	L.SHKDDSPDLPKLKPDPNTLCDEF.K + Glycatio
3204	104 – 126	748.8452	2991.3517	2991.3543	-0.86	2	27	0.0022	1	U	L.SHKDDSPDLPKLKPDPNTLCDEF.K + 2 Glycat
3205	104 – 126	998.1246	2991.3519	2991.3543	-0.81	2	19	0.014	1	U	L.SHKDDSPDLPKLKPDPNTLCDEF.K + 2 Glycat
3206	104 – 126	748.8456	2991.3535	2991.3543	-0.28	2	14	0.037	1	U	L.SHKDDSPDLPKLKPDPNTLCDEF.K + 2 Glycat
680	127 – 134	351.1903	1050.5492	1050.5498	-0.58	1	24	0.0039	1	U	F.KADEKKFW.G
681	127 – 134	351.1905	1050.5496	1050.5498	-0.16	1	15	0.032	1	U	F.KADEKKFW.G
682	127 – 134	351.1905	1050.5497	1050.5498	-0.069	1	15	0.035	1	U	F.KADEKKFW.G
683	127 – 134	351.1905	1050.5497	1050.5498	-0.069	1	23	0.0051	1	U	F.KADEKKFW.G
684	127 – 134	526.2823	1050.5500	1050.5498	0.21	1	38	0.00014	1	U	F.KADEKKFW.G
685	127 – 134	526.2823	1050.5501	1050.5498	0.33	1	37	0.00019	1	U	F.KADEKKFW.G
686	127 – 134	526.2824	1050.5503	1050.5498	0.44	1	39	0.00013	1	U	F.KADEKKFW.G
957	127 – 134	607.3084	1212.6021	1212.6026	-0.40	1	34	0.00039	1	U	F.KADEKKFW.G + Glycation (K)
958	127 – 134	607.3084	1212.6023	1212.6026	-0.30	1	20	0.011	1	U	F.KADEKKFW.G + Glycation (K)
959	127 – 134	607.3085	1212.6024	1212.6026	-0.20	1	29	0.0012	1	U	F.KADEKKFW.G + Glycation (K)
960	127 – 134	405.2081	1212.6025	1212.6026	-0.13	1	19	0.012	1	U	F.KADEKKFW.G + Glycation (K)
961	127 – 134	607.3085	1212.6025	1212.6026	-0.10	1	26	0.0026	1	U	F.KADEKKFW.G + Glycation (K)
962	127 – 134	607.3085	1212.6025	1212.6026	-0.10	1	18	0.014	1	U	F.KADEKKFW.G + Glycation (K)
963	127 – 134	405.2081	1212.6026	1212.6026	-0.057	1	26	0.0023	1	U	F.KADEKKFW.G + Glycation (K)
964	127 – 134	607.3086	1212.6026	1212.6026	-0.0025	1	28	0.0015	1	U	F.KADEKKFW.G + Glycation (K)
965	127 – 134	405.2082	1212.6027	1212.6026	0.017	1	45	3.1e-005	1	U	F.KADEKKFW.G + Glycation (K)
967	127 – 134	405.2082	1212.6027	1212.6026	0.092	1	21	0.0086	1	U	F.KADEKKFW.G + Glycation (K)
968	127 – 134	405.2082	1212.6027	1212.6026	0.092	1	16	0.025	1	U	F.KADEKKFW.G + Glycation (K)
969	127 – 134	405.2082	1212.6028	1212.6026	0.17	1	32	0.00059	1	U	F.KADEKKFW.G + Glycation (K)
1298	127 – 134	688.3345	1374.6545	1374.6555	-0.69	1	20	0.0096	1	U	F.KADEKKFW.G + 2 Glycation (K)
351	148 – 154	426.7286	851.4427	851.4429	-0.26	3	21	0.0082	1	U	Y.FYAPELL.Y
352	148 – 154	426.7287	851.4428	851.4429	-0.045	3	21	0.0083	1	U	Y.FYAPELL.Y
353	148 – 154	426.7288	851.4430	851.4429	0.096	3	21	0.0082	1	U	Y.FYAPELL.Y
354	148 – 154	426.7288	851.4430	851.4429	0.17	3	19	0.012	1	U	Y.FYAPELL.Y
355	148 – 154	426.7288	851.4430	851.4429	0.17	3	21	0.0082	1	U	Y.FYAPELL.Y
2236	161 – 177	993.4102	1984.8058	1984.8077	-0.99	1	63	5.3e-007	1	U	Y.NGVFQECCQAEDKGACL.L
2237	161 – 177	662.6093	1984.8061	1984.8077	-0.82	1	30	0.00089	1	U	Y.NGVFQECCQAEDKGACL.L
2238	161 – 177	993.4103	1984.8061	1984.8077	-0.81	1	59	1.2e-006	1	U	Y.NGVFQECCQAEDKGACL.L
2410	161 – 177	1074.4364	2146.8582	2146.8606	-1.08	1	49	1.3e-005	1	U	Y.NGVFQECCQAEDKGACL.L + Glycation (K)
2411	161 – 177	716.6270	2146.8592	2146.8606	-0.63	1	28	0.0017	1	U	Y.NGVFQECCQAEDKGACL.L + Glycation (K)
2412	161 – 177	1074.4370	2146.8595	2146.8606	-0.51	1	59	1.2e-006	1	U	Y.NGVFQECCQAEDKGACL.L + Glycation (K)

Query	Start - End	Observed	Mr(expt)	Mr(calc)	ppm	M	Score	Expect	Rank	U	Peptide
1719	165 - 177	784.8098	1567.6051	1567.6065	-0.92	0	62	5.9e-007	1	U	F.QECCQAEDKGACL.L
1720	165 - 177	784.8101	1567.6057	1567.6065	-0.52	0	60	1e-006	1	U	F.QECCQAEDKGACL.L
1952	165 - 177	865.8348	1729.6551	1729.6593	-2.43	0	39	0.00013	1	U	F.QECCQAEDKGACL.L + Glycation (K)
759	219 - 227	541.2873	1080.5601	1080.5604	-0.21	0	23	0.0046	1	U	L.SQKFPKAEF.V
760	219 - 227	361.1940	1080.5602	1080.5604	-0.19	0	14	0.045	1	U	L.SQKFPKAEF.V
761	219 - 227	541.2874	1080.5603	1080.5604	-0.10	0	23	0.005	1	U	L.SQKFPKAEF.V
762	219 - 227	541.2874	1080.5603	1080.5604	-0.10	0	22	0.006	1	U	L.SQKFPKAEF.V
763	219 - 227	541.2875	1080.5605	1080.5604	0.14	0	23	0.0047	1	U	L.SQKFPKAEF.V
765	219 - 227	541.2876	1080.5606	1080.5604	0.25	0	22	0.0067	1	U	L.SQKFPKAEF.V
766	219 - 227	541.2877	1080.5608	1080.5604	0.36	0	22	0.006	1	U	L.SQKFPKAEF.V
1008	219 - 227	622.3135	1242.6124	1242.6132	-0.64	0	15	0.033	1	U	L.SQKFPKAEF.V + Glycation (K)
1009	219 - 227	622.3135	1242.6125	1242.6132	-0.54	0	19	0.012	1	U	L.SQKFPKAEF.V + Glycation (K)
1010	219 - 227	622.3137	1242.6128	1242.6132	-0.35	0	23	0.0052	1	U	L.SQKFPKAEF.V + Glycation (K)
1011	219 - 227	415.2116	1242.6130	1242.6132	-0.16	0	14	0.039	1	U	L.SQKFPKAEF.V + Glycation (K)
1012	219 - 227	415.2117	1242.6134	1242.6132	0.13	0	14	0.037	1	U	L.SQKFPKAEF.V + Glycation (K)
1013	219 - 227	415.2118	1242.6135	1242.6132	0.27	0	13	0.049	1	U	L.SQKFPKAEF.V + Glycation (K)
1014	219 - 227	622.3142	1242.6137	1242.6132	0.44	0	17	0.021	1	U	L.SQKFPKAEF.V + Glycation (K)
112	228 - 233	344.7154	687.4162	687.4167	-0.67	0	19	0.012	1	U	F.VEVTKL.V
114	228 - 233	344.7154	687.4163	687.4167	-0.49	0	19	0.014	1	U	F.VEVTKL.V
116	228 - 233	344.7155	687.4165	687.4167	-0.29	0	21	0.0088	1	U	F.VEVTKL.V
117	228 - 233	344.7157	687.4168	687.4167	0.15	0	19	0.014	1	U	F.VEVTKL.V
338	228 - 233	425.7418	849.4690	849.4695	-0.57	0	26	0.0024	1	U	F.VEVTKL.V + Glycation (K)
340	228 - 233	425.7419	849.4692	849.4695	-0.36	0	26	0.0026	1	U	F.VEVTKL.V + Glycation (K)
341	228 - 233	425.7419	849.4692	849.4695	-0.36	0	26	0.0024	1	U	F.VEVTKL.V + Glycation (K)
343	228 - 233	425.7420	849.4694	849.4695	-0.15	0	26	0.0027	1	U	F.VEVTKL.V + Glycation (K)
345	228 - 233	425.7420	849.4694	849.4695	-0.077	0	26	0.0023	1	U	F.VEVTKL.V + Glycation (K)
346	228 - 233	425.7421	849.4697	849.4695	0.23	0	26	0.0023	1	U	F.VEVTKL.V + Glycation (K)
1105	228 - 237	639.8534	1277.6922	1277.6966	-3.44	1	40	0.00011	1	U	F.VEVTKLVTDL.T + Glycation (K)
2294	234 - 250	675.6677	2023.9813	2023.9819	-0.30	2	17	0.02	1	U	L.VTDLTKVHKECCHGDLL.E
2469	234 - 250	729.6854	2186.0343	2186.0348	-0.24	2	18	0.015	1	U	L.VTDLTKVHKECCHGDLL.E + Glycation (K)
3260	234 - 259	1024.1377	3069.3913	3069.3954	-1.35	3	53	5.5e-006	1	U	L.VTDLTKVHKECCHGDLLECADDRADL.A
3261	234 - 259	768.3552	3069.3915	3069.3954	-1.26	3	65	3e-007	1	U	L.VTDLTKVHKECCHGDLLECADDRADL.A
3262	234 - 259	1024.1381	3069.3924	3069.3954	-1.00	3	82	6.2e-009	1	U	L.VTDLTKVHKECCHGDLLECADDRADL.A
3263	234 - 259	768.3556	3069.3935	3069.3954	-0.63	3	76	2.6e-008	1	U	L.VTDLTKVHKECCHGDLLECADDRADL.A
3264	234 - 259	614.8862	3069.3945	3069.3954	-0.31	3	19	0.013	1	U	L.VTDLTKVHKECCHGDLLECADDRADL.A
3265	234 - 259	614.8862	3069.3948	3069.3954	-0.21	3	24	0.004	1	U	L.VTDLTKVHKECCHGDLLECADDRADL.A
3350	234 - 259	1078.1541	3231.4403	3231.4482	-2.45	3	96	2.6e-010	1	U	L.VTDLTKVHKECCHGDLLECADDRADL.A + Glyca
3351	234 - 259	808.8683	3231.4441	3231.4482	-1.30	3	85	3.4e-009	1	U	L.VTDLTKVHKECCHGDLLECADDRADL.A + Glyca
3352	234 - 259	808.8686	3231.4453	3231.4482	-0.92	3	96	2.8e-010	1	U	L.VTDLTKVHKECCHGDLLECADDRADL.A + Glyca
3353	234 - 259	1078.1560	3231.4462	3231.4482	-0.63	3	112	5.7e-012	1	U	L.VTDLTKVHKECCHGDLLECADDRADL.A + Glyca
3354	234 - 259	647.2967	3231.4471	3231.4482	-0.36	3	38	0.00016	1	U	L.VTDLTKVHKECCHGDLLECADDRADL.A + Glyca
3355	234 - 259	647.2969	3231.4480	3231.4482	-0.069	3	35	0.0003	1	U	L.VTDLTKVHKECCHGDLLECADDRADL.A + Glyca
3356	234 - 259	808.8693	3231.4482	3231.4482	-0.020	3	74	3.6e-008	1	U	L.VTDLTKVHKECCHGDLLECADDRADL.A + Glyca
3409	234 - 259	849.3810	3393.4951	3393.5011	-1.77	3	74	4.2e-008	1	U	L.VTDLTKVHKECCHGDLLECADDRADL.A + 2 Gly
3450	234 - 262	899.4177	3593.6418	3593.6436	-0.52	4	18	0.016	1	U	L.VTDLTKVHKECCHGDLLECADDRADLAKY.I + Gl
3452	234 - 262	899.4180	3593.6428	3593.6436	-0.24	4	19	0.013	1	U	L.VTDLTKVHKECCHGDLLECADDRADLAKY.I + Gl
3496	234 - 262	1252.9009	3755.6808	3755.6965	-4.17	4	25	0.003	1	U	L.VTDLTKVHKECCHGDLLECADDRADLAKY.I + 2
722	251 - 259	532.7192	1063.4238	1063.4240	-0.20	0	24	0.0042	1	U	L.ECADDRADL.A
723	251 - 259	532.7192	1063.4238	1063.4240	-0.20	0	24	0.004	1	U	L.ECADDRADL.A
724	251 - 259	532.7196	1063.4246	1063.4240	0.58	0	24	0.0041	1	U	L.ECADDRADL.A
1422	251 - 262	713.8166	1425.6186	1425.6194	-0.55	1	30	0.001	1	U	L.ECADDRADLAKY.I
1423	251 - 262	713.8166	1425.6187	1425.6194	-0.47	1	33	0.00053	1	U	L.ECADDRADLAKY.I
1762	251 - 262	794.8425	1587.6705	1587.6722	-1.09	1	15	0.029	1	U	L.ECADDRADLAKY.I + Glycation (K)
1765	251 - 262	530.2316	1587.6729	1587.6722	0.40	1	18	0.016	1	U	L.ECADDRADLAKY.I + Glycation (K)
1323	263 - 274	697.3328	1392.6511	1392.6555	-3.16	0	53	5.5e-006	1	U	Y.ICDNQDTISSKL.K
1324	263 - 274	697.3343	1392.6541	1392.6555	-0.96	0	65	2.9e-007	1	U	Y.ICDNQDTISSKL.K
1325	263 - 274	697.3343	1392.6541	1392.6555	-0.96	0	24	0.0042	1	U	Y.ICDNQDTISSKL.K
1326	263 - 274	697.3344	1392.6543	1392.6555	-0.88	0	42	6.2e-005	1	U	Y.ICDNQDTISSKL.K
1327	263 - 274	697.3347	1392.6548	1392.6555	-0.52	0	27	0.0021	1	U	Y.ICDNQDTISSKL.K
1328	263 - 274	697.3347	1392.6549	1392.6555	-0.43	0	53	5.1e-006	1	U	Y.ICDNQDTISSKL.K
1329	263 - 274	697.3347	1392.6549	1392.6555	-0.43	0	64	3.5e-007	1	U	Y.ICDNQDTISSKL.K
1330	263 - 274	697.3347	1392.6549	1392.6555	-0.43	0	67	1.9e-007	1	U	Y.ICDNQDTISSKL.K
1331	263 - 274	697.3349	1392.6552	1392.6555	-0.17	0	48	1.7e-005	1	U	Y.ICDNQDTISSKL.K
1332	263 - 274	697.3349	1392.6552	1392.6555	-0.17	0	67	2e-007	1	U	Y.ICDNQDTISSKL.K
1333	263 - 274	697.3350	1392.6554	1392.6555	-0.085	0	65	3.5e-007	1	U	Y.ICDNQDTISSKL.K
1334	263 - 274	697.3356	1392.6567	1392.6555	0.88	0	67	2e-007	1	U	Y.ICDNQDTISSKL.K
1693	263 - 274	778.3606	1554.7066	1554.7083	-1.07	0	45	3.1e-005	1	U	Y.ICDNQDTISSKL.K + Glycation (K)
1694	263 - 274	778.3607	1554.7068	1554.7083	-0.99	0	52	5.7e-006	1	U	Y.ICDNQDTISSKL.K + Glycation (K)
1695	263 - 274	778.3607	1554.7069	1554.7083	-0.92	0	45	3.2e-005	1	U	Y.ICDNQDTISSKL.K + Glycation (K)
1696	263 - 274	778.3607	1554.7069	1554.7083	-0.92	0	45	3.3e-005	1	U	Y.ICDNQDTISSKL.K + Glycation (K)
1697	263 - 274	778.3608	1554.7070	1554.7083	-0.84	0	50	9e-006	1	U	Y.ICDNQDTISSKL.K + Glycation (K)
1698	263 - 274	778.3614	1554.7083	1554.7083	0.023	0	48	1.6e-005	1	U	Y.ICDNQDTISSKL.K + Glycation (K)
1699	263 - 274	778.3618	1554.7090	1554.7083	0.42	0	36	0.00028	1	U	Y.ICDNQDTISSKL.K + Glycation (K)
3615	275 - 318	1071.1100	5350.5136	5350.5029	1.99	4	24	0.0036	1	U	L.KECCDKPLLEKSHCIAEVEKDAIPENLPPLTADFA
3568	283 - 318	865.0169	4320.0482	4320.0453	0.66	3	19	0.012	1	U	L.LEKSHCIAEVEKDAIPENLPPLTADFAEDKDVCKN
3576	283 - 318	1121.5337	4482.1057	4482.0982	1.67	3	16	0.026	1	U	L.LEKSHCIAEVEKDAIPENLPPLTADFAEDKDVCKN
950	319 - 329	606.7983	1211.5820	1211.5822	-0.20	2	26	0.0027	1	U	Y.QEAKDAFLGSF.L
951	319 - 329	606.7983	1211.5820	1211.5822	-0.20	2	21	0.0075	1	U	Y.QEAKDAFLGSF.L
952	319 - 329	606.7983	1211.5820	1211.5822	-0.20	2	24	0.004	1	U	Y.QEAKDAFLGSF.L
953	319 - 329	606.7984	1211.5822	1211.5822	0.0025	2	21	0.0075	1	U	Y.QEAKDAFLGSF.L
955	319 - 329	606.7985	1211.5824	1211.5822	0.10	2	13	0.046	1	U	Y.QEAKDAFLGSF.L
1286	319 - 329	687.8243	1373.6340	1373.6351	-0.77	2	20	0.0097	1	U	Y.QEAKDAFLGSF.L + Glycation (K)
1287	319 - 329	687.8243	1373.6341	1373.6351	-0.69	2	26	0.0024	1	U	Y.QEAKDAFLGSF.L + Glycation (K)
1288	319 - 329	687.8243	1373.6341	1373.6351	-0.69	2	25	0.0032	1	U	Y.QEAKDAFLGSF.L + Glycation (K)
1290	319 - 329	687.8249	1373.6352	1373.6351	0.11	2	19	0.012	1	U	Y.QEAKDAFLGSF.L + Glycation (K)
1291	319 - 329	687.8252	1373.6358	1373.6351	0.57	2	26	0.0025	1	U	Y.QEAKDAFLGSF.L + Glycation (K)
1188	319 - 330	663.3403	1324.6661	1324.6663	-0.15	3	26	0.0025	1	U	Y.QEAKDAFLGSFL.Y
1546	319 - 331	744.8714	1487.7282	1487.7296	-0.93	4	26	0.0025	1	U	Y.QEAKDAFLGSFLY.E

Query	Start - End	Observed	Mr(expt)	Mr(calc)	ppm	M	Score	Expect	Rank	U	Peptide
1547	319 - 331	744.8716	1487.7287	1487.7296	-0.61	4	28	0.0014	1	U	Y.QEAKDAFLGSFLY.E
1550	319 - 331	744.8723	1487.7301	1487.7296	0.29	4	28	0.0015	1	U	Y.QEAKDAFLGSFLY.E
1845	319 - 331	825.8975	1649.7804	1649.7825	-1.27	4	25	0.0033	1	U	Y.QEAKDAFLGSFLY.E + Glycation (K)
1846	319 - 331	825.8976	1649.7807	1649.7825	-1.05	4	30	0.0011	1	U	Y.QEAKDAFLGSFLY.E + Glycation (K)
1847	319 - 331	825.8978	1649.7811	1649.7825	-0.82	4	20	0.011	1	U	Y.QEAKDAFLGSFLY.E + Glycation (K)
1848	319 - 331	825.8978	1649.7811	1649.7825	-0.82	4	18	0.016	1	U	Y.QEAKDAFLGSFLY.E + Glycation (K)
418	346 - 352	298.1798	891.5174	891.5178	-0.38	2	17	0.02	1	U	L.LRLAKEY.E
419	346 - 352	446.7662	891.5178	891.5178	0.030	2	26	0.0028	1	U	L.LRLAKEY.E
420	346 - 352	298.1800	891.5181	891.5178	0.43	2	14	0.044	1	U	L.LRLAKEY.E
702	346 - 352	352.1973	1053.5701	1053.5706	-0.49	2	14	0.038	1	U	L.LRLAKEY.E + Glycation (K)
703	346 - 352	352.1973	1053.5702	1053.5706	-0.40	2	18	0.017	1	U	L.LRLAKEY.E + Glycation (K)
704	346 - 352	527.7924	1053.5702	1053.5706	-0.40	2	14	0.042	1	U	L.LRLAKEY.E + Glycation (K)
705	346 - 352	352.1974	1053.5703	1053.5706	-0.32	2	18	0.015	1	U	L.LRLAKEY.E + Glycation (K)
706	346 - 352	352.1975	1053.5705	1053.5706	-0.062	2	13	0.046	1	U	L.LRLAKEY.E + Glycation (K)
709	346 - 352	352.1975	1053.5707	1053.5706	0.11	2	14	0.036	1	U	L.LRLAKEY.E + Glycation (K)
3201	347 - 369	748.5747	2990.2697	2990.2732	-1.15	3	41	8.2e-005	1	U	L.RLAKEYEATLEECCAKDDPHACY.S + Glycatio
3202	347 - 369	748.5754	2990.2724	2990.2732	-0.25	3	46	2.8e-005	1	U	L.RLAKEYEATLEECCAKDDPHACY.S + Glycatio
3203	347 - 369	748.5756	2990.2731	2990.2732	-0.014	3	47	2e-005	1	U	L.RLAKEYEATLEECCAKDDPHACY.S + Glycatio
2948	349 - 369	854.0185	2559.0336	2559.0352	-0.60	2	46	2.8e-005	1	U	L.AKEYEATLEECCAKDDPHACY.S
2949	349 - 369	640.7663	2559.0361	2559.0352	0.36	2	26	0.0023	1	U	L.AKEYEATLEECCAKDDPHACY.S
3003	349 - 369	908.0373	2721.0840	2721.0880	-1.47	2	17	0.02	1	U	L.AKEYEATLEECCAKDDPHACY.S + Glycation
3004	349 - 369	681.2790	2721.0869	2721.0880	-0.43	2	23	0.0053	1	U	L.AKEYEATLEECCAKDDPHACY.S + Glycation
2335	353 - 369	1034.9047	2067.7948	2067.7972	-1.18	1	52	6e-006	1	U	Y.EATLEECCAKDDPHACY.S
2336	353 - 369	1034.9050	2067.7955	2067.7972	-0.82	1	62	6.4e-007	1	U	Y.EATLEECCAKDDPHACY.S
2337	353 - 369	1034.9052	2067.7957	2067.7972	-0.71	1	64	3.9e-007	1	U	Y.EATLEECCAKDDPHACY.S
2338	353 - 369	690.2726	2067.7961	2067.7972	-0.54	1	23	0.0056	1	U	Y.EATLEECCAKDDPHACY.S
2339	353 - 369	690.2728	2067.7965	2067.7972	-0.35	1	26	0.0024	1	U	Y.EATLEECCAKDDPHACY.S
2340	353 - 369	690.2728	2067.7967	2067.7972	-0.27	1	30	0.001	1	U	Y.EATLEECCAKDDPHACY.S
2341	353 - 369	690.2731	2067.7976	2067.7972	0.17	1	31	0.00077	1	U	Y.EATLEECCAKDDPHACY.S
2342	353 - 369	690.2737	2067.7994	2067.7972	1.05	1	22	0.006	1	U	Y.EATLEECCAKDDPHACY.S
2554	353 - 369	1115.9316	2229.8487	2229.8500	-0.59	1	35	0.00033	1	U	Y.EATLEECCAKDDPHACY.S + Glycation (K)
2555	353 - 369	1115.9320	2229.8495	2229.8500	-0.26	1	36	0.00025	1	U	Y.EATLEECCAKDDPHACY.S + Glycation (K)
280	370 - 376	405.2237	808.4328	808.4331	-0.38	1	31	0.00072	1	U	Y.STVFDKL.K
281	370 - 376	405.2238	808.4329	808.4331	-0.16	1	21	0.0088	1	U	Y.STVFDKL.K
282	370 - 376	405.2238	808.4330	808.4331	-0.058	1	37	0.00019	1	U	Y.STVFDKL.K
283	370 - 376	405.2239	808.4332	808.4331	0.16	1	40	9.5e-005	1	U	Y.STVFDKL.K
542	370 - 376	486.2501	970.4856	970.4859	-0.28	1	18	0.015	1	U	Y.STVFDKL.K + Glycation (K)
543	370 - 376	486.2502	970.4857	970.4859	-0.16	1	26	0.0024	1	U	Y.STVFDKL.K + Glycation (K)
544	370 - 376	486.2502	970.4858	970.4859	-0.096	1	22	0.0065	1	U	Y.STVFDKL.K + Glycation (K)
545	370 - 376	486.2502	970.4859	970.4859	-0.034	1	26	0.0022	1	U	Y.STVFDKL.K + Glycation (K)
546	370 - 376	486.2502	970.4859	970.4859	-0.034	1	18	0.015	1	U	Y.STVFDKL.K + Glycation (K)
547	370 - 376	486.2502	970.4859	970.4859	0.028	1	30	0.001	1	U	Y.STVFDKL.K + Glycation (K)
548	370 - 376	486.2502	970.4859	970.4859	0.028	1	21	0.0087	1	U	Y.STVFDKL.K + Glycation (K)
1683	374 - 386	774.9215	1547.8285	1547.8307	-1.46	2	52	6.5e-006	1	U	F.DKLKHLVDEPQNL.I
1685	374 - 386	774.9219	1547.8292	1547.8307	-0.99	2	61	8.1e-007	1	U	F.DKLKHLVDEPQNL.I
1686	374 - 386	774.9222	1547.8299	1547.8307	-0.52	2	53	5.4e-006	1	U	F.DKLKHLVDEPQNL.I
1688	374 - 386	516.9509	1547.8308	1547.8307	0.031	2	30	0.00099	1	U	F.DKLKHLVDEPQNL.I
1689	374 - 386	516.9511	1547.8315	1547.8307	0.50	2	17	0.022	1	U	F.DKLKHLVDEPQNL.I
1934	374 - 386	855.9475	1709.8805	1709.8836	-1.81	2	19	0.011	1	U	F.DKLKHLVDEPQNL.I + Glycation (K)
1935	374 - 386	855.9480	1709.8814	1709.8836	-1.24	2	27	0.0018	1	U	F.DKLKHLVDEPQNL.I + Glycation (K)
1937	374 - 386	855.9483	1709.8820	1709.8836	-0.88	2	25	0.0031	1	U	F.DKLKHLVDEPQNL.I + Glycation (K)
1938	374 - 386	855.9484	1709.8823	1709.8836	-0.73	2	37	0.00021	1	U	F.DKLKHLVDEPQNL.I + Glycation (K)
1939	374 - 386	855.9484	1709.8823	1709.8836	-0.73	2	24	0.0037	1	U	F.DKLKHLVDEPQNL.I + Glycation (K)
1940	374 - 386	570.9683	1709.8831	1709.8836	-0.25	2	16	0.027	1	U	F.DKLKHLVDEPQNL.I + Glycation (K)
1941	374 - 386	570.9683	1709.8831	1709.8836	-0.25	2	19	0.012	1	U	F.DKLKHLVDEPQNL.I + Glycation (K)
1942	374 - 386	570.9684	1709.8835	1709.8836	-0.040	2	14	0.041	1	U	F.DKLKHLVDEPQNL.I + Glycation (K)
1943	374 - 386	570.9684	1709.8835	1709.8836	-0.040	2	18	0.017	1	U	F.DKLKHLVDEPQNL.I + Glycation (K)
2132	374 - 386	936.9747	1871.9349	1871.9364	-0.79	2	22	0.0064	1	U	F.DKLKHLVDEPQNL.I + 2 Glycation (K)
2134	374 - 386	624.9857	1871.9353	1871.9364	-0.56	2	14	0.041	1	U	F.DKLKHLVDEPQNL.I + 2 Glycation (K)
2136	374 - 386	624.9859	1871.9359	1871.9364	-0.27	2	17	0.02	1	U	F.DKLKHLVDEPQNL.I + 2 Glycation (K)
2137	374 - 386	936.9753	1871.9360	1871.9364	-0.20	2	27	0.0022	1	U	F.DKLKHLVDEPQNL.I + 2 Glycation (K)
915	377 - 386	596.8193	1191.6241	1191.6248	-0.54	1	54	3.7e-006	1	U	L.KHLVDEPQNL.I
916	377 - 386	398.2153	1191.6242	1191.6248	-0.51	1	73	4.8e-008	1	U	L.KHLVDEPQNL.I
917	377 - 386	398.2154	1191.6243	1191.6248	-0.36	1	73	4.7e-008	1	U	L.KHLVDEPQNL.I
918	377 - 386	398.2154	1191.6243	1191.6248	-0.36	1	73	4.7e-008	1	U	L.KHLVDEPQNL.I
919	377 - 386	596.8196	1191.6246	1191.6248	-0.14	1	47	2.1e-005	1	U	L.KHLVDEPQNL.I
920	377 - 386	596.8196	1191.6247	1191.6248	-0.036	1	54	3.7e-006	1	U	L.KHLVDEPQNL.I
921	377 - 386	596.8197	1191.6248	1191.6248	0.065	1	62	5.8e-007	1	U	L.KHLVDEPQNL.I
922	377 - 386	398.2156	1191.6249	1191.6248	0.12	1	67	2e-007	1	U	L.KHLVDEPQNL.I
1242	377 - 386	677.8453	1353.6760	1353.6776	-1.17	1	19	0.012	1	U	L.KHLVDEPQNL.I + Glycation (K)
1243	377 - 386	677.8454	1353.6762	1353.6776	-1.00	1	21	0.0075	1	U	L.KHLVDEPQNL.I + Glycation (K)
1244	377 - 386	677.8456	1353.6766	1353.6776	-0.73	1	21	0.0076	1	U	L.KHLVDEPQNL.I + Glycation (K)
1245	377 - 386	677.8456	1353.6767	1353.6776	-0.64	1	18	0.015	1	U	L.KHLVDEPQNL.I + Glycation (K)
1246	377 - 386	452.2331	1353.6774	1353.6776	-0.18	1	26	0.0024	1	U	L.KHLVDEPQNL.I + Glycation (K)
1247	377 - 386	677.8460	1353.6775	1353.6776	-0.095	1	14	0.038	1	U	L.KHLVDEPQNL.I + Glycation (K)
1248	377 - 386	452.2331	1353.6775	1353.6776	-0.048	1	14	0.042	1	U	L.KHLVDEPQNL.I + Glycation (K)
1249	377 - 386	452.2332	1353.6778	1353.6776	0.17	1	31	0.00073	1	U	L.KHLVDEPQNL.I + Glycation (K)
687	387 - 394	526.7451	1051.4757	1051.4757	0.010	0	19	0.012	1	U	L.IKQNCDQF.E
688	387 - 394	526.7451	1051.4757	1051.4757	0.010	0	25	0.0034	1	U	L.IKQNCDQF.E
689	387 - 394	526.7452	1051.4758	1051.4757	0.12	0	21	0.0076	1	U	L.IKQNCDQF.E
690	387 - 394	526.7452	1051.4759	1051.4757	0.24	0	25	0.0035	1	U	L.IKQNCDQF.E
971	387 - 394	607.7713	1213.5280	1213.5285	-0.37	0	16	0.025	1	U	L.IKQNCDQF.E + Glycation (K)
972	387 - 394	607.7713	1213.5280	1213.5285	-0.37	0	20	0.011	1	U	L.IKQNCDQF.E + Glycation (K)
973	387 - 394	607.7714	1213.5283	1213.5285	-0.18	0	16	0.026	1	U	L.IKQNCDQF.E + Glycation (K)
974	387 - 394	607.7714	1213.5283	1213.5285	-0.18	0	26	0.0024	1	U	L.IKQNCDQF.E + Glycation (K)
2038	387 - 400	886.4157	1770.8169	1770.8247	-4.40	2	34	0.0004	1	U	L.IKQNCDQFEKLGEY.G
2039	387 - 400	886.4179	1770.8211	1770.8247	-1.98	2	30	0.0011	1	U	L.IKQNCDQFEKLGEY.G
2040	387 - 400	886.4186	1770.8226	1770.8247	-1.16	2	43	4.9e-005	1	U	L.IKQNCDQFEKLGEY.G

Query	Start – End	Observed	Mr(expt)	Mr(calc)	ppm	M	Score	Expect	Rank	U	Peptide
2041	387 – 400	886.4187	1770.8228	1770.8247	-1.02	2	41	7.2e-005	1	U	L.IKQNCDQFEKLGEY.G
2042	387 – 400	886.4191	1770.8237	1770.8247	-0.54	2	32	0.00068	1	U	L.IKQNCDQFEKLGEY.G
2043	387 – 400	591.2819	1770.8239	1770.8247	-0.41	2	40	0.0001	1	U	L.IKQNCDQFEKLGEY.G
2044	387 – 400	591.2819	1770.8239	1770.8247	-0.41	2	27	0.0021	1	U	L.IKQNCDQFEKLGEY.G
2045	387 – 400	591.2821	1770.8245	1770.8247	-0.11	2	43	4.6e-005	1	U	L.IKQNCDQFEKLGEY.G
2046	387 – 400	591.2823	1770.8250	1770.8247	0.22	2	33	0.0005	1	U	L.IKQNCDQFEKLGEY.G
2192	387 – 400	967.4451	1932.8756	1932.8775	-0.98	2	28	0.0017	1	U	L.IKQNCDQFEKLGEY.G + Glycation (K)
2193	387 – 400	967.4454	1932.8762	1932.8775	-0.67	2	27	0.0019	1	U	L.IKQNCDQFEKLGEY.G + Glycation (K)
2194	387 – 400	967.4454	1932.8763	1932.8775	-0.61	2	26	0.0028	1	U	L.IKQNCDQFEKLGEY.G + Glycation (K)
2195	387 – 400	645.2995	1932.8767	1932.8775	-0.42	2	30	0.0011	1	U	L.IKQNCDQFEKLGEY.G + Glycation (K)
2196	387 – 400	645.2996	1932.8769	1932.8775	-0.33	2	32	0.00064	1	U	L.IKQNCDQFEKLGEY.G + Glycation (K)
2197	387 – 400	645.2997	1932.8772	1932.8775	-0.14	2	32	0.00063	1	U	L.IKQNCDQFEKLGEY.G + Glycation (K)
2198	387 – 400	645.3000	1932.8781	1932.8775	0.34	2	32	0.00063	1	U	L.IKQNCDQFEKLGEY.G + Glycation (K)
2199	387 – 400	967.4466	1932.8786	1932.8775	0.59	2	20	0.009	1	U	L.IKQNCDQFEKLGEY.G + Glycation (K)
2200	387 – 400	645.3002	1932.8787	1932.8775	0.62	2	27	0.0021	1	U	L.IKQNCDQFEKLGEY.G + Glycation (K)
2357	387 – 400	699.3164	2094.9272	2094.9303	-1.48	2	27	0.002	1	U	L.IKQNCDQFEKLGEY.G + 2 Glycation (K)
2358	387 – 400	699.3165	2094.9276	2094.9303	-1.30	2	17	0.019	1	U	L.IKQNCDQFEKLGEY.G + 2 Glycation (K)
2361	387 – 400	699.3170	2094.9291	2094.9303	-0.60	2	20	0.01	1	U	L.IKQNCDQFEKLGEY.G + 2 Glycation (K)
2362	387 – 400	699.3171	2094.9294	2094.9303	-0.43	2	31	0.00082	1	U	L.IKQNCDQFEKLGEY.G + 2 Glycation (K)
245	423 – 429	395.2268	788.4391	788.4392	-0.18	0	24	0.0036	1	U	L.VEVSRSL.G
246	423 – 429	395.2268	788.4391	788.4392	-0.10	0	29	0.0014	1	U	L.VEVSRSL.G
247	423 – 429	395.2271	788.4396	788.4392	0.51	0	24	0.0042	1	U	L.VEVSRSL.G
228	457 – 462	387.7166	773.4187	773.4218	-3.95	1	16	0.026	1	U	L.NRLCVL.H
229	457 – 462	387.7179	773.4212	773.4218	-0.69	1	16	0.024	1	U	L.NRLCVL.H
230	457 – 462	387.7179	773.4213	773.4218	-0.61	1	16	0.022	1	U	L.NRLCVL.H
1662	488 – 501	769.8881	1537.7616	1537.7664	-3.15	2	34	0.00036	1	U	F.SALTPDETYVPKAF.D
1663	488 – 501	769.8897	1537.7649	1537.7664	-1.01	2	32	0.00066	1	U	F.SALTPDETYVPKAF.D
1664	488 – 501	769.8899	1537.7652	1537.7664	-0.77	2	39	0.00013	1	U	F.SALTPDETYVPKAF.D
1665	488 – 501	769.8900	1537.7653	1537.7664	-0.70	2	33	0.00055	1	U	F.SALTPDETYVPKAF.D
1666	488 – 501	769.8900	1537.7653	1537.7664	-0.70	2	38	0.00015	1	U	F.SALTPDETYVPKAF.D
1913	488 – 501	850.9159	1699.8173	1699.8192	-1.12	2	25	0.0031	1	U	F.SALTPDETYVPKAF.D + Glycation (K)
1914	488 – 501	850.9160	1699.8175	1699.8192	-1.03	2	22	0.006	1	U	F.SALTPDETYVPKAF.D + Glycation (K)
1915	488 – 501	850.9163	1699.8180	1699.8192	-0.75	2	18	0.015	1	U	F.SALTPDETYVPKAF.D + Glycation (K)
2449	488 – 506	724.3679	2170.0819	2170.0834	-0.67	4	26	0.0024	1	U	F.SALTPDETYVPKAFDEKLF.T
2450	488 – 506	724.3679	2170.0819	2170.0834	-0.67	4	30	0.0011	1	U	F.SALTPDETYVPKAFDEKLF.T
2452	488 – 506	1086.0483	2170.0821	2170.0834	-0.58	4	25	0.0031	1	U	F.SALTPDETYVPKAFDEKLF.T
2453	488 – 506	1086.0483	2170.0821	2170.0834	-0.58	4	27	0.0018	1	U	F.SALTPDETYVPKAFDEKLF.T
2454	488 – 506	1086.0493	2170.0841	2170.0834	0.32	4	25	0.0031	1	U	F.SALTPDETYVPKAFDEKLF.T
2455	488 – 506	724.3728	2170.0966	2170.0834	6.07	4	29	0.0013	1	U	F.SALTPDETYVPKAFDEKLF.T
2693	488 – 506	1167.0752	2332.1358	2332.1362	-0.16	4	16	0.023	1	U	F.SALTPDETYVPKAFDEKLF.T + Glycation (K)
2694	488 – 506	1167.0754	2332.1363	2332.1362	0.048	4	13	0.047	1	U	F.SALTPDETYVPKAFDEKLF.T + Glycation (K)
2695	488 – 506	1167.0754	2332.1363	2332.1362	0.048	4	23	0.0054	1	U	F.SALTPDETYVPKAFDEKLF.T + Glycation (K)
1069	491 – 501	634.3135	1266.6125	1266.6132	-0.55	1	47	1.8e-005	1	U	L.TPDETYVPKAF.D
1070	491 – 501	634.3137	1266.6129	1266.6132	-0.26	1	44	3.7e-005	1	U	L.TPDETYVPKAF.D
2168	491 – 506	633.9839	1898.9298	1898.9302	-0.18	3	30	0.0011	1	U	L.TPDETYVPKAFDEKLF.T
2169	491 – 506	633.9846	1898.9319	1898.9302	0.87	3	31	0.00072	1	U	L.TPDETYVPKAFDEKLF.T
2170	491 – 506	950.4768	1898.9389	1898.9302	4.61	3	19	0.014	1	U	L.TPDETYVPKAFDEKLF.T
2332	491 – 506	688.0013	2060.9820	2060.9830	-0.49	3	21	0.0077	1	U	L.TPDETYVPKAFDEKLF.T + Glycation (K)
172	497 – 501	362.1997	722.3848	722.3851	-0.34	0	19	0.012	1	U	Y.VPKAF.D + Glycation (K)
173	497 – 501	362.1997	722.3849	722.3851	-0.18	0	19	0.012	1	U	Y.VPKAF.D + Glycation (K)
174	497 – 501	362.1998	722.3851	722.3851	0.072	0	17	0.018	1	U	Y.VPKAF.D + Glycation (K)
925	497 – 506	597.3315	1192.6485	1192.6492	-0.56	2	31	0.00076	1	U	Y.VPKAFDEKLF.T
926	497 – 506	398.5568	1192.6486	1192.6492	-0.48	2	19	0.012	1	U	Y.VPKAFDEKLF.T
927	497 – 506	597.3316	1192.6486	1192.6492	-0.46	2	30	0.001	1	U	Y.VPKAFDEKLF.T
928	497 – 506	597.3317	1192.6488	1192.6492	-0.35	2	40	9e-005	1	U	Y.VPKAFDEKLF.T
929	497 – 506	398.5569	1192.6489	1192.6492	-0.22	2	18	0.017	1	U	Y.VPKAFDEKLF.T
930	497 – 506	597.3318	1192.6490	1192.6492	-0.14	2	33	0.00047	1	U	Y.VPKAFDEKLF.T
931	497 – 506	398.5570	1192.6491	1192.6492	-0.074	2	18	0.014	1	U	Y.VPKAFDEKLF.T
932	497 – 506	398.5571	1192.6495	1192.6492	0.23	2	22	0.0065	1	U	Y.VPKAFDEKLF.T
933	497 – 506	398.5571	1192.6496	1192.6492	0.30	2	18	0.015	1	U	Y.VPKAFDEKLF.T
1252	497 – 506	678.3575	1354.7005	1354.7020	-1.11	2	22	0.006	1	U	Y.VPKAFDEKLF.T + Glycation (K)
1253	497 – 506	678.3576	1354.7006	1354.7020	-1.02	2	13	0.046	1	U	Y.VPKAFDEKLF.T + Glycation (K)
1254	497 – 506	678.3579	1354.7013	1354.7020	-0.56	2	22	0.0063	1	U	Y.VPKAFDEKLF.T + Glycation (K)
1255	497 – 506	678.3579	1354.7013	1354.7020	-0.56	2	24	0.0044	1	U	Y.VPKAFDEKLF.T + Glycation (K)
1256	497 – 506	452.5745	1354.7016	1354.7020	-0.35	2	26	0.0024	1	U	Y.VPKAFDEKLF.T + Glycation (K)
1257	497 – 506	452.5745	1354.7016	1354.7020	-0.35	2	23	0.0048	1	U	Y.VPKAFDEKLF.T + Glycation (K)
1258	497 – 506	452.5745	1354.7017	1354.7020	-0.22	2	23	0.0051	1	U	Y.VPKAFDEKLF.T + Glycation (K)
1259	497 – 506	452.5745	1354.7018	1354.7020	-0.15	2	14	0.036	1	U	Y.VPKAFDEKLF.T + Glycation (K)
1622	497 – 506	506.5921	1516.7546	1516.7549	-0.19	2	21	0.0076	1	U	Y.VPKAFDEKLF.T + 2 Glycation (K)
67	502 – 506	326.1707	650.3269	650.3275	-0.99	1	15	0.035	1	U	F.DEKLF.T
68	502 – 506	326.1709	650.3272	650.3275	-0.43	1	14	0.04	1	U	F.DEKLF.T
71	502 – 506	326.1710	650.3274	650.3275	-0.25	1	15	0.035	1	U	F.DEKLF.T
72	502 – 506	326.1710	650.3275	650.3275	-0.065	1	14	0.036	1	U	F.DEKLF.T
73	502 – 506	326.1710	650.3275	650.3275	-0.065	1	14	0.039	1	U	F.DEKLF.T
289	502 – 506	407.1975	812.3804	812.3804	0.064	1	15	0.035	1	U	F.DEKLF.T + Glycation (K)
3331	507 – 532	794.4200	3173.6510	3173.6530	-0.62	3	30	0.00091	1	U	F.TFHADICTLPDTEKQIKKQTALVELL.K + Glyca
3332	507 – 532	1058.8910	3173.6511	3173.6530	-0.58	3	25	0.0034	1	U	F.TFHADICTLPDTEKQIKKQTALVELL.K + Glyca
3334	507 – 532	1058.8922	3173.6548	3173.6530	0.58	3	32	0.00067	1	U	F.TFHADICTLPDTEKQIKKQTALVELL.K + Glyca
3335	507 – 532	794.4217	3173.6579	3173.6530	1.55	3	20	0.011	1	U	F.TFHADICTLPDTEKQIKKQTALVELL.K + Glyca
3387	507 – 532	834.9316	3335.6975	3335.7058	-2.50	3	34	0.00037	1	U	F.TFHADICTLPDTEKQIKKQTALVELL.K + 2 Gly
3388	507 – 532	834.9325	3335.7009	3335.7058	-1.47	3	43	5.3e-005	1	U	F.TFHADICTLPDTEKQIKKQTALVELL.K + 2 Gly
3392	507 – 532	834.9343	3335.7080	3335.7058	0.67	3	30	0.00099	1	U	F.TFHADICTLPDTEKQIKKQTALVELL.K + 2 Gly
3393	507 – 532	1112.9103	3335.7090	3335.7058	0.96	3	30	0.0011	1	U	F.TFHADICTLPDTEKQIKKQTALVELL.K + 2 Gly
3394	507 – 532	668.1492	3335.7095	3335.7058	1.10	3	33	0.0005	1	U	F.TFHADICTLPDTEKQIKKQTALVELL.K + 2 Gly
3396	507 – 532	834.9351	3335.7111	3335.7058	1.60	3	29	0.0013	1	U	F.TFHADICTLPDTEKQIKKQTALVELL.K + 2 Gly
3397	507 – 532	834.9362	3335.7159	3335.7058	3.02	3	32	0.00058	1	U	F.TFHADICTLPDTEKQIKKQTALVELL.K + 2 Gly
3427	507 – 532	875.4481	3497.7633	3497.7586	1.34	3	18	0.017	1	U	F.TFHADICTLPDTEKQIKKQTALVELL.K + 3 Gl
3428	507 – 532	875.4481	3497.7633	3497.7586	1.34	3	56	2.5e-006	1	U	F.TFHADICTLPDTEKQIKKQTALVELL.K + 3 Gl

Query	Start - End	Observed	Mr(expt)	Mr(calc)	ppm	M	Score	Expect	Rank	U	Peptide
2868	509 - 528	824.7586	2471.2540	2471.2577	-1.51	0	50	9.2e-006	1	U	F.HADICTLPDTEKQIKKQTAL.V + Glycation (K
2869	509 - 528	618.8211	2471.2553	2471.2577	-0.97	0	28	0.0017	1	U	F.HADICTLPDTEKQIKKQTAL.V + Glycation (K
2870	509 - 528	618.8212	2471.2556	2471.2577	-0.87	0	28	0.0015	1	U	F.HADICTLPDTEKQIKKQTAL.V + Glycation (K
2969	509 - 528	878.7764	2633.3073	2633.3106	-1.25	0	49	1.2e-005	1	U	F.HADICTLPDTEKQIKKQTAL.V + 2 Glycation
2970	509 - 528	659.3343	2633.3083	2633.3106	-0.86	0	24	0.0041	1	U	F.HADICTLPDTEKQIKKQTAL.V + 2 Glycation
2971	509 - 528	659.3347	2633.3098	2633.3106	-0.30	0	26	0.0024	1	U	F.HADICTLPDTEKQIKKQTAL.V + 2 Glycation
2972	509 - 528	659.3350	2633.3110	2633.3106	0.16	0	21	0.0078	1	U	F.HADICTLPDTEKQIKKQTAL.V + 2 Glycation
2974	509 - 528	878.7794	2633.3164	2633.3106	2.23	0	40	8.9e-005	1	U	F.HADICTLPDTEKQIKKQTAL.V + 2 Glycation
3048	509 - 528	699.8492	2795.3676	2795.3634	1.51	0	13	0.046	1	U	F.HADICTLPDTEKQIKKQTAL.V + 3 Glycation
3056	509 - 531	938.4913	2812.4522	2812.4528	-0.23	1	42	6.4e-005	1	U	F.HADICTLPDTEKQIKKQTALVEL.L + Glycation
3057	509 - 531	704.1207	2812.4536	2812.4528	0.27	1	22	0.0066	1	U	F.HADICTLPDTEKQIKKQTALVEL.L + Glycation
3174	509 - 531	744.6326	2974.5012	2974.5056	-1.50	1	28	0.0017	1	U	F.HADICTLPDTEKQIKKQTALVEL.L + 2 Glycat
3175	509 - 531	992.5085	2974.5038	2974.5056	-0.62	1	32	0.00064	1	U	F.HADICTLPDTEKQIKKQTALVEL.L + 2 Glycat
3303	509 - 531	785.1450	3136.5510	3136.5585	-2.38	1	15	0.03	1	U	F.HADICTLPDTEKQIKKQTALVEL.L + 3 Glycat
3020	509 - 532	691.8790	2763.4868	2763.4840	0.98	2	31	0.00077	1	U	F.HADICTLPDTEKQIKKQTALVELL.K
3130	509 - 532	976.1848	2925.5324	2925.5369	-1.52	2	55	3.3e-006	1	U	F.HADICTLPDTEKQIKKQTALVELL.K + Glycati
3131	509 - 532	732.3907	2925.5337	2925.5369	-1.10	2	49	1.3e-005	1	U	F.HADICTLPDTEKQIKKQTALVELL.K + Glycati
3132	509 - 532	976.1854	2925.5343	2925.5369	-0.89	2	19	0.014	1	U	F.HADICTLPDTEKQIKKQTALVELL.K + Glycati
3134	509 - 532	976.1865	2925.5377	2925.5369	0.30	2	75	3e-008	1	U	F.HADICTLPDTEKQIKKQTALVELL.K + Glycati
3135	509 - 532	732.3918	2925.5380	2925.5369	0.39	2	39	0.00011	1	U	F.HADICTLPDTEKQIKKQTALVELL.K + Glycati
3136	509 - 532	976.1875	2925.5407	2925.5369	1.30	2	53	4.8e-006	1	U	F.HADICTLPDTEKQIKKQTALVELL.K + Glycati
3137	509 - 532	732.3926	2925.5412	2925.5369	1.49	2	44	4e-005	1	U	F.HADICTLPDTEKQIKKQTALVELL.K + Glycati
3272	509 - 532	772.9035	3087.5849	3087.5897	-1.55	2	74	3.9e-008	1	U	F.HADICTLPDTEKQIKKQTALVELL.K + 2 Glyca
3273	509 - 532	1030.2029	3087.5868	3087.5897	-0.93	2	60	9.2e-007	1	U	F.HADICTLPDTEKQIKKQTALVELL.K + 2 Glyca
3274	509 - 532	1030.2029	3087.5868	3087.5897	-0.93	2	60	9.5e-007	1	U	F.HADICTLPDTEKQIKKQTALVELL.K + 2 Glyca
3275	509 - 532	772.9040	3087.5871	3087.5897	-0.84	2	60	1e-006	1	U	F.HADICTLPDTEKQIKKQTALVELL.K + 2 Glyca
3276	509 - 532	1030.2036	3087.5890	3087.5897	-0.22	2	54	3.9e-006	1	U	F.HADICTLPDTEKQIKKQTALVELL.K + 2 Glyca
3277	509 - 532	1030.2037	3087.5893	3087.5897	-0.14	2	58	1.5e-006	1	U	F.HADICTLPDTEKQIKKQTALVELL.K + 2 Glyca
3278	509 - 532	1030.2042	3087.5909	3087.5897	0.38	2	18	0.016	1	U	F.HADICTLPDTEKQIKKQTALVELL.K + 2 Glyca
3281	509 - 532	772.9053	3087.5922	3087.5897	0.82	2	34	0.00043	1	U	F.HADICTLPDTEKQIKKQTALVELL.K + 2 Glyca
3282	509 - 532	772.9054	3087.5925	3087.5897	0.90	2	23	0.0056	1	U	F.HADICTLPDTEKQIKKQTALVELL.K + 2 Glyca
3283	509 - 532	772.9062	3087.5957	3087.5897	1.93	2	47	1.8e-005	1	U	F.HADICTLPDTEKQIKKQTALVELL.K + 2 Glyca
3365	509 - 532	813.4174	3249.6403	3249.6425	-0.67	2	53	5.2e-006	1	U	F.HADICTLPDTEKQIKKQTALVELL.K + 3 Glyca
3366	509 - 532	813.4178	3249.6421	3249.6425	-0.14	2	49	1.2e-005	1	U	F.HADICTLPDTEKQIKKQTALVELL.K + 3 Glyca
3367	509 - 532	813.4179	3249.6423	3249.6425	-0.068	2	18	0.014	1	U	F.HADICTLPDTEKQIKKQTALVELL.K + 3 Glyca
3368	509 - 532	1084.2218	3249.6436	3249.6425	0.32	2	40	0.0001	1	U	F.HADICTLPDTEKQIKKQTALVELL.K + 3 Glyca
3369	509 - 532	1084.2222	3249.6448	3249.6425	0.70	2	29	0.0013	1	U	F.HADICTLPDTEKQIKKQTALVELL.K + 3 Glyca
376	544 - 550	434.7150	867.4155	867.4160	-0.60	0	15	0.029	1	U	L.KTVMENF.V
377	544 - 550	434.7151	867.4156	867.4160	-0.46	0	17	0.022	1	U	L.KTVMENF.V
2270	551 - 567	1003.4246	2004.8346	2004.8380	-1.70	1	60	1e-006	1	U	F.VAFVDKCCAADDKEACF.A
2271	551 - 567	1003.4254	2004.8363	2004.8380	-0.84	1	67	2.1e-007	1	U	F.VAFVDKCCAADDKEACF.A
2272	551 - 567	669.2863	2004.8371	2004.8380	-0.42	1	37	0.00022	1	U	F.VAFVDKCCAADDKEACF.A
2273	551 - 567	669.2864	2004.8373	2004.8380	-0.33	1	33	0.00047	1	U	F.VAFVDKCCAADDKEACF.A
2274	551 - 567	669.2865	2004.8377	2004.8380	-0.15	1	46	2.8e-005	1	U	F.VAFVDKCCAADDKEACF.A
2441	551 - 567	723.3035	2166.8888	2166.8908	-0.94	1	32	0.00057	1	U	F.VAFVDKCCAADDKEACF.A + Glycation (K)
2442	551 - 567	723.3039	2166.8898	2166.8908	-0.45	1	34	0.00042	1	U	F.VAFVDKCCAADDKEACF.A + Glycation (K)
2443	551 - 567	1084.4523	2166.8900	2166.8908	-0.38	1	37	0.00022	1	U	F.VAFVDKCCAADDKEACF.A + Glycation (K)
2444	551 - 567	1084.4524	2166.8902	2166.8908	-0.27	1	38	0.00016	1	U	F.VAFVDKCCAADDKEACF.A + Glycation (K)
2445	551 - 567	723.3041	2166.8906	2166.8908	-0.100	1	28	0.0018	1	U	F.VAFVDKCCAADDKEACF.A + Glycation (K)
1889	554 - 567	844.8379	1687.6613	1687.6640	-1.59	0	40	0.0001	1	U	F.VDKCCAADDKEACF.A
1890	554 - 567	844.8380	1687.6615	1687.6640	-1.52	0	47	1.9e-005	1	U	F.VDKCCAADDKEACF.A
1891	554 - 567	844.8381	1687.6617	1687.6640	-1.38	0	47	1.9e-005	1	U	F.VDKCCAADDKEACF.A
1892	554 - 567	844.8385	1687.6624	1687.6640	-0.94	0	53	4.9e-006	1	U	F.VDKCCAADDKEACF.A
1894	554 - 567	563.5617	1687.6633	1687.6640	-0.43	0	27	0.0022	1	U	F.VDKCCAADDKEACF.A
1895	554 - 567	563.5618	1687.6635	1687.6640	-0.33	0	30	0.00096	1	U	F.VDKCCAADDKEACF.A
1896	554 - 567	563.5619	1687.6638	1687.6640	-0.11	0	25	0.0032	1	U	F.VDKCCAADDKEACF.A
1897	554 - 567	563.5620	1687.6640	1687.6640	-0.0059	0	18	0.017	1	U	F.VDKCCAADDKEACF.A
1898	554 - 567	563.5621	1687.6646	1687.6640	0.31	0	25	0.0029	1	U	F.VDKCCAADDKEACF.A
1899	554 - 567	563.5621	1687.6646	1687.6640	0.31	0	33	0.00047	1	U	F.VDKCCAADDKEACF.A
2103	554 - 567	925.8644	1849.7143	1849.7169	-1.37	0	36	0.00025	1	U	F.VDKCCAADDKEACF.A + Glycation (K)
2104	554 - 567	925.8648	1849.7151	1849.7169	-0.97	0	42	5.8e-005	1	U	F.VDKCCAADDKEACF.A + Glycation (K)
2105	554 - 567	925.8648	1849.7151	1849.7169	-0.97	0	39	0.00011	1	U	F.VDKCCAADDKEACF.A + Glycation (K)
2106	554 - 567	617.5790	1849.7151	1849.7169	-0.94	0	33	0.00052	1	U	F.VDKCCAADDKEACF.A + Glycation (K)
2107	554 - 567	617.5790	1849.7151	1849.7169	-0.94	0	43	4.6e-005	1	U	F.VDKCCAADDKEACF.A + Glycation (K)
2108	554 - 567	617.5792	1849.7157	1849.7169	-0.65	0	33	0.00051	1	U	F.VDKCCAADDKEACF.A + Glycation (K)
2109	554 - 567	617.5797	1849.7171	1849.7169	0.14	0	28	0.0015	1	U	F.VDKCCAADDKEACF.A + Glycation (K)
2110	554 - 567	617.5797	1849.7173	1849.7169	0.24	0	46	2.5e-005	1	U	F.VDKCCAADDKEACF.A + Glycation (K)
2277	554 - 567	671.5970	2011.7693	2011.7697	-0.18	0	20	0.0089	1	U	F.VDKCCAADDKEACF.A + 2 Glycation (K)
153	568 - 574	357.2131	712.4117	712.4119	-0.31	0	15	0.034	1	U	F.AVEGPKL.V
1751	568 - 583	792.4529	1582.8912	1582.8930	-1.15	2	33	0.00048	1	U	F.AVEGPKLVVSTQTALA.-
1752	568 - 583	792.4531	1582.8916	1582.8930	-0.92	2	47	2.1e-005	1	U	F.AVEGPKLVVSTQTALA.-
1753	568 - 583	792.4532	1582.8918	1582.8930	-0.75	2	33	0.00055	1	U	F.AVEGPKLVVSTQTALA.-
1990	568 - 583	873.4794	1744.9443	1744.9458	-0.88	2	51	8.4e-006	1	U	F.AVEGPKLVVSTQTALA.- + Glycation (K)
1991	568 - 583	873.4794	1744.9443	1744.9458	-0.88	2	64	4.1e-007	1	U	F.AVEGPKLVVSTQTALA.- + Glycation (K)
1992	568 - 583	873.4797	1744.9448	1744.9458	-0.60	2	25	0.003	1	U	F.AVEGPKLVVSTQTALA.- + Glycation (K)
1993	568 - 583	873.4804	1744.9461	1744.9458	0.17	2	22	0.0061	1	U	F.AVEGPKLVVSTQTALA.- + Glycation (K)
1994	568 - 583	873.4810	1744.9474	1744.9458	0.87	2	30	0.0011	1	U	F.AVEGPKLVVSTQTALA.- + Glycation (K)

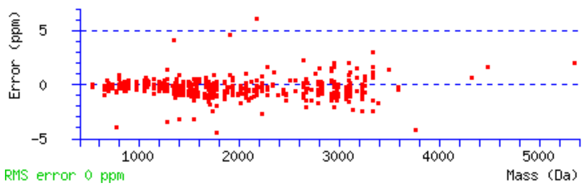

Mascot: http://www.matrixscience.com/

Appendix 5. Mascot search results of trypsin digestion for BSA-3

MATRIX SCIENCE MASCOT Search Results

Protein View: >gi|00000

BSA

**Database:**	BSA
**Score:**	35603
**Monoisotopic mass (M_r):**	68386
**Calculated pI:**	5.60

Sequence similarity is available as **an NCBI BLAST search of >gi|00000 against nr**.

Search parameters

**MS data file:**	P2019021811.mgf
**Enzyme:**	Trypsin/P: cuts C-term side of KR.
**Fixed modifications:**	**Carbamidomethyl (C)**
**Variable modifications:**	**Glycation (K)**, **Glycation (Protein N-term)**

Protein sequence coverage: 92%

Matched peptides shown in ***bold red***.

1	DTHKSEIAHR	**FKDLGEEHFK**	**GLVLIAFSQY**	**LQQCPFDEHV**	**KLVNELTEFA**
51	**KTCVADESHA**	**GCEKSLHTLF**	**GDELCKVASL**	**RETYGDMADC**	**CEKQEPERNE**
101	**CFLSHKDDSP**	**DLPKLKPDPN**	**TLCDEFKADE**	**KKFWGKYLYE**	**IARRHPYFYA**
151	**PELLYYANKY**	**NGVFQECCQA**	**EDKGACLLPK**	**IETMR**EKVLA	SSARQRLR**CA**
201	**SIQKFGERAL**	**KAWSVARLSQ**	**KFPKAEFVEV**	**TKLVTDLTKV**	**HKECCHGDLL**
251	**ECADDRADLA**	**KYICDNQDTI**	**SSKLKECCDK**	**PLLEKSHCIA**	**EVEKDAIPEN**
301	**LPPLTADFAE**	**DKDVCKNYQE**	**AKDAFLGSFL**	**YEYSRRHPEY**	**AVSVLLRLAK**
351	**EYEATLEECC**	**AKDDPHACYS**	**TVFDKLKHLV**	**DEPQNLIKQN**	**CDQFEKLGEY**
401	**GFQNALIVR**Y	TR**KVPQVSTP**	**TLVEVSR**SLG	KVGTRCCTKP	ESER**MPCTED**
451	**YLSLILNRLC**	**VLHEKTPVSE**	**KVTKCCTESL**	**VNRRPCFSAL**	**TPDETYVPKA**
501	**FDEKLFTFHA**	**DICTLPDTEK**	**QIKKQTALVE**	**LLKHKPKATE**	**EQLKTVMENF**
551	**VAFVDKCCAA**	**DDKEACFAVE**	**GPKLVVSTQT**	**ALA**	

Unformatted sequence string: **583 residues** (for pasting into other applications).

Sort by ◉ residue number ○ increasing mass ○ decreasing mass
Show ◉ matched peptides only ○ predicted peptides also

Query	Start – End	Observed	Mr(expt)	Mr(calc)	ppm	M	Score	Expect	Rank	U	Peptide
1169	11 – 20	417.2114	1248.6124	1248.6139	-1.20	1	25	0.0031	1	U	R.FKDLGEEHFK.G
1171	11 – 20	313.1606	1248.6131	1248.6139	-0.62	1	19	0.012	1	U	R.FKDLGEEHFK.G
1172	11 – 20	417.2118	1248.6135	1248.6139	-0.27	1	47	2.1e-005	1	U	R.FKDLGEEHFK.G
1173	11 – 20	313.1607	1248.6139	1248.6139	-0.016	1	18	0.015	1	U	R.FKDLGEEHFK.G
1174	11 – 20	625.3144	1248.6143	1248.6139	0.38	1	44	4.3e-005	1	U	R.FKDLGEEHFK.G
1175	11 – 20	417.2121	1248.6145	1248.6139	0.48	1	47	2.1e-005	1	U	R.FKDLGEEHFK.G
1176	11 – 20	417.2121	1248.6146	1248.6139	0.55	1	32	0.0006	1	U	R.FKDLGEEHFK.G
1177	11 – 20	625.3150	1248.6154	1248.6139	1.26	1	56	2.3e-006	1	U	R.FKDLGEEHFK.G
1559	11 – 20	706.3399	1410.6652	1410.6667	-1.03	1	47	2.1e-005	1	U	R.FKDLGEEHFK.G + Glycation (K)
1560	11 – 20	706.3401	1410.6657	1410.6667	-0.68	1	33	0.00055	1	U	R.FKDLGEEHFK.G + Glycation (K)
1561	11 – 20	353.6738	1410.6660	1410.6667	-0.51	1	18	0.015	1	U	R.FKDLGEEHFK.G + Glycation (K)
1562	11 – 20	706.3403	1410.6661	1410.6667	-0.42	1	43	4.6e-005	1	U	R.FKDLGEEHFK.G + Glycation (K)
1563	11 – 20	353.6738	1410.6662	1410.6667	-0.34	1	34	0.00039	1	U	R.FKDLGEEHFK.G + Glycation (K)
1564	11 – 20	353.6739	1410.6665	1410.6667	-0.17	1	47	1.9e-005	1	U	R.FKDLGEEHFK.G + Glycation (K)
1565	11 – 20	471.2294	1410.6665	1410.6667	-0.17	1	47	2.1e-005	1	U	R.FKDLGEEHFK.G + Glycation (K)
1566	11 – 20	353.6739	1410.6666	1410.6667	-0.089	1	42	6.4e-005	1	U	R.FKDLGEEHFK.G + Glycation (K)
1567	11 – 20	706.3406	1410.6666	1410.6667	-0.067	1	44	4.4e-005	1	U	R.FKDLGEEHFK.G + Glycation (K)
1568	11 – 20	471.2295	1410.6667	1410.6667	0.021	1	35	0.00029	1	U	R.FKDLGEEHFK.G + Glycation (K)
1569	11 – 20	471.2296	1410.6670	1410.6667	0.21	1	32	0.00064	1	U	R.FKDLGEEHFK.G + Glycation (K)
1570	11 – 20	353.6740	1410.6671	1410.6667	0.25	1	45	2.9e-005	1	U	R.FKDLGEEHFK.G + Glycation (K)
1571	11 – 20	471.2296	1410.6671	1410.6667	0.28	1	39	0.00013	1	U	R.FKDLGEEHFK.G + Glycation (K)
1572	11 – 20	471.2299	1410.6678	1410.6667	0.74	1	35	0.0003	1	U	R.FKDLGEEHFK.G + Glycation (K)
522	13 – 20	487.7323	973.4500	973.4505	-0.46	0	23	0.0047	1	U	K.DLGEEHFK.G
523	13 – 20	325.4907	973.4502	973.4505	-0.27	0	22	0.006	1	U	K.DLGEEHFK.G
524	13 – 20	487.7324	973.4503	973.4505	-0.16	0	25	0.0032	1	U	K.DLGEEHFK.G
525	13 – 20	487.7325	973.4504	973.4505	-0.095	0	28	0.0017	1	U	K.DLGEEHFK.G
526	13 – 20	487.7326	973.4506	973.4505	0.090	0	14	0.043	1	U	K.DLGEEHFK.G
527	13 – 20	325.4908	973.4506	973.4505	0.10	0	21	0.0082	1	U	K.DLGEEHFK.G
528	13 – 20	325.4908	973.4506	973.4505	0.10	0	25	0.0029	1	U	K.DLGEEHFK.G
4580	21 – 41	831.4260	2491.2563	2491.2570	-0.29	0	45	3.4e-005	1	U	K.GLVLIAFSQYLQQCPFDEHVK.L
4581	21 – 41	831.4261	2491.2564	2491.2570	-0.21	0	53	5e-006	1	U	K.GLVLIAFSQYLQQCPFDEHVK.L
1024	42 – 51	582.3187	1162.6228	1162.6234	-0.52	0	62	7e-007	1	U	K.LVNELTEFAK.T
1025	42 – 51	582.3188	1162.6230	1162.6234	-0.29	0	58	1.7e-006	1	U	K.LVNELTEFAK.T
1026	42 – 51	582.3190	1162.6235	1162.6234	0.12	0	61	7.4e-007	1	U	K.LVNELTEFAK.T
1027	42 – 51	582.3190	1162.6235	1162.6234	0.12	0	61	7.3e-007	1	U	K.LVNELTEFAK.T
4946	42 – 64	924.0871	2769.2395	2769.2473	-2.83	1	88	1.6e-009	1	U	K.LVNELTEFAKTCVADESHAGCEK.S + Glycatio
4947	42 – 64	924.0885	2769.2437	2769.2473	-1.32	1	84	4.1e-009	1	U	K.LVNELTEFAKTCVADESHAGCEK.S + Glycatio
4948	42 – 64	924.0888	2769.2444	2769.2473	-1.04	1	80	9.9e-009	1	U	K.LVNELTEFAKTCVADESHAGCEK.S + Glycatio
4949	42 – 64	693.3185	2769.2451	2769.2473	-0.82	1	87	1.9e-009	1	U	K.LVNELTEFAKTCVADESHAGCEK.S + Glycatio
4950	42 – 64	693.3192	2769.2477	2769.2473	0.15	1	68	1.5e-007	1	U	K.LVNELTEFAKTCVADESHAGCEK.S + Glycatio
5970	42 – 76	723.0024	4331.9706	4331.9760	-1.24	2	46	2.7e-005	1	U	K.LVNELTEFAKTCVADESHAGCEKSLHTLFGDELCK
5971	42 – 76	1084.0006	4331.9733	4331.9760	-0.61	2	52	6.7e-006	1	U	K.LVNELTEFAKTCVADESHAGCEKSLHTLFGDELCK

Query	Start – End	Observed	Mr(expt)	Mr(calc)	ppm	M	Score	Expect	Rank	U	Peptide
5972	42 – 76	867.4021	4331.9741	4331.9760	-0.43	2	51	8.9e-006	1	U	K.LVNELTEFAKTCVADESHAGCEKSLHTLFGDELCK
6215	42 – 81	837.7302	5020.3377	5020.3515	-2.76	3	34	0.00041	1	U	K.LVNELTEFAKTCVADESHAGCEKSLHTLFGDELCK
1747	52 – 64	732.2976	1462.5807	1462.5817	-0.69	0	91	8.3e-010	1	U	K.TCVADESHAGCEK.S
1748	52 – 64	732.2977	1462.5809	1462.5817	-0.53	0	91	8.2e-010	1	U	K.TCVADESHAGCEK.S
1749	52 – 64	488.5345	1462.5817	1462.5817	0.036	0	66	2.3e-007	1	U	K.TCVADESHAGCEK.S
1750	52 – 64	488.5345	1462.5817	1462.5817	0.036	0	50	1.1e-005	1	U	K.TCVADESHAGCEK.S
1751	52 – 64	488.5346	1462.5818	1462.5817	0.097	0	76	2.4e-008	1	U	K.TCVADESHAGCEK.S
5233	52 – 76	1009.4417	3025.3031	3025.3103	-2.39	1	73	5.4e-008	1	U	K.TCVADESHAGCEKSLHTLFGDELCK.V + Glycat
5234	52 – 76	757.3335	3025.3049	3025.3103	-1.80	1	48	1.5e-005	1	U	K.TCVADESHAGCEKSLHTLFGDELCK.V + Glycat
5235	52 – 76	606.0687	3025.3069	3025.3103	-1.13	1	32	0.00057	1	U	K.TCVADESHAGCEKSLHTLFGDELCK.V + Glycat
5236	52 – 76	757.3341	3025.3073	3025.3103	-1.00	1	59	1.1e-006	1	U	K.TCVADESHAGCEKSLHTLFGDELCK.V + Glycat
5237	52 – 76	1009.4434	3025.3083	3025.3103	-0.69	1	77	2e-008	1	U	K.TCVADESHAGCEKSLHTLFGDELCK.V + Glycat
5238	52 – 76	1009.4434	3025.3084	3025.3103	-0.63	1	70	8.9e-008	1	U	K.TCVADESHAGCEKSLHTLFGDELCK.V + Glycat
5239	52 – 76	606.0691	3025.3091	3025.3103	-0.42	1	59	1.1e-006	1	U	K.TCVADESHAGCEKSLHTLFGDELCK.V + Glycat
5240	52 – 76	757.3347	3025.3098	3025.3103	-0.19	1	56	2.5e-006	1	U	K.TCVADESHAGCEKSLHTLFGDELCK.V + Glycat
5674	52 – 81	743.7438	3713.6825	3713.6859	-0.92	2	41	8.3e-005	1	U	K.TCVADESHAGCEKSLHTLFGDELCKVASLR.E + :
5675	52 – 81	929.4284	3713.6845	3713.6859	-0.37	2	51	8.6e-006	1	U	K.TCVADESHAGCEKSLHTLFGDELCKVASLR.E + :
5676	52 – 81	619.9547	3713.6846	3713.6859	-0.35	2	29	0.0012	1	U	K.TCVADESHAGCEKSLHTLFGDELCKVASLR.E + :
5677	52 – 81	743.7442	3713.6846	3713.6859	-0.34	2	23	0.0046	1	U	K.TCVADESHAGCEKSLHTLFGDELCKVASLR.E + :
5678	52 – 81	743.7444	3713.6855	3713.6859	-0.10	2	15	0.029	1	U	K.TCVADESHAGCEKSLHTLFGDELCKVASLR.E + :
5679	52 – 81	1238.9032	3713.6878	3713.6859	0.50	2	71	7.9e-008	1	U	K.TCVADESHAGCEKSLHTLFGDELCKVASLR.E + :
5680	52 – 81	929.4296	3713.6892	3713.6859	0.88	2	27	0.0019	1	U	K.TCVADESHAGCEKSLHTLFGDELCKVASLR.E + :
5681	52 – 81	1238.9052	3713.6936	3713.6859	2.08	2	33	0.00045	1	U	K.TCVADESHAGCEKSLHTLFGDELCKVASLR.E + :
5682	52 – 81	929.4315	3713.6967	3713.6859	2.92	2	32	0.0007	1	U	K.TCVADESHAGCEKSLHTLFGDELCKVASLR.E + :
5683	52 – 81	619.9577	3713.7025	3713.6859	4.48	2	17	0.018	1	U	K.TCVADESHAGCEKSLHTLFGDELCKVASLR.E + :
1604	65 – 76	710.3497	1418.6849	1418.6864	-1.05	0	64	4.1e-007	1	U	K.SLHTLFGDELCK.V
1605	65 – 76	710.3499	1418.6853	1418.6864	-0.80	0	59	1.2e-006	1	U	K.SLHTLFGDELCK.V
1606	65 – 76	473.9026	1418.6860	1418.6864	-0.26	0	51	8.3e-006	1	U	K.SLHTLFGDELCK.V
1607	65 – 76	473.9027	1418.6863	1418.6864	-0.070	0	21	0.0071	1	U	K.SLHTLFGDELCK.V
1608	65 – 76	473.9028	1418.6865	1418.6864	0.057	0	48	1.5e-005	1	U	K.SLHTLFGDELCK.V
1609	65 – 76	710.3511	1418.6876	1418.6864	0.84	0	65	3.4e-007	1	U	K.SLHTLFGDELCK.V
1610	65 – 76	473.9045	1418.6915	1418.6864	3.61	0	50	9.1e-006	1	U	K.SLHTLFGDELCK.V
3952	65 – 81	703.3605	2107.0596	2107.0620	-1.12	1	52	5.8e-006	1	U	K.SLHTLFGDELCKVASLR.E + Glycation (K)
3953	65 – 81	1054.5374	2107.0601	2107.0620	-0.86	1	40	0.0001	1	U	K.SLHTLFGDELCKVASLR.E + Glycation (K)
3954	65 – 81	703.3610	2107.0612	2107.0620	-0.34	1	53	5.4e-006	1	U	K.SLHTLFGDELCKVASLR.E + Glycation (K)
3955	65 – 81	703.3611	2107.0616	2107.0620	-0.16	1	56	2.4e-006	1	U	K.SLHTLFGDELCKVASLR.E + Glycation (K)
3956	65 – 81	1054.5385	2107.0623	2107.0620	0.19	1	39	0.00013	1	U	K.SLHTLFGDELCKVASLR.E + Glycation (K)
3957	65 – 81	527.7730	2107.0627	2107.0620	0.35	1	36	0.00027	1	U	K.SLHTLFGDELCKVASLR.E + Glycation (K)
3958	65 – 81	703.3616	2107.0629	2107.0620	0.44	1	52	6.8e-006	1	U	K.SLHTLFGDELCKVASLR.E + Glycation (K)
3959	65 – 81	527.7731	2107.0632	2107.0620	0.58	1	39	0.00014	1	U	K.SLHTLFGDELCKVASLR.E + Glycation (K)
3960	65 – 81	527.7731	2107.0634	2107.0620	0.70	1	51	8.8e-006	1	U	K.SLHTLFGDELCKVASLR.E + Glycation (K)
1829	82 – 93	739.7645	1477.5145	1477.5160	-0.98	0	66	2.5e-007	1	U	R.ETYGDMADCCEK.Q
1830	82 – 93	739.7648	1477.5151	1477.5160	-0.58	0	79	1.2e-008	1	U	R.ETYGDMADCCEK.Q
1831	82 – 93	739.7648	1477.5151	1477.5160	-0.58	0	79	1.2e-008	1	U	R.ETYGDMADCCEK.Q
4195	82 – 98	760.6281	2278.8623	2278.8664	-1.79	1	59	1.2e-006	1	U	R.ETYGDMADCCEKQEPER.N + Glycation (K)
4196	82 – 98	760.6283	2278.8631	2278.8664	-1.46	1	48	1.7e-005	1	U	R.ETYGDMADCCEKQEPER.N + Glycation (K)
4197	82 – 98	760.6289	2278.8649	2278.8664	-0.66	1	48	1.5e-005	1	U	R.ETYGDMADCCEKQEPER.N + Glycation (K)
4198	82 – 98	570.7285	2278.8850	2278.8664	8.15	1	26	0.0025	1	U	R.ETYGDMADCCEKQEPER.N + Glycation (K)
680	99 – 106	517.7392	1033.4638	1033.4651	-1.21	0	34	0.00041	1	U	R.NECFLSHK.D
681	99 – 106	517.7394	1033.4642	1033.4651	-0.87	0	26	0.0027	1	U	R.NECFLSHK.D
682	99 – 106	345.4954	1033.4644	1033.4651	-0.65	0	38	0.00018	1	U	R.NECFLSHK.D
683	99 – 106	517.7395	1033.4644	1033.4651	-0.63	0	35	0.00029	1	U	R.NECFLSHK.D
684	99 – 106	517.7396	1033.4647	1033.4651	-0.40	0	34	0.00042	1	U	R.NECFLSHK.D
685	99 – 106	345.4955	1033.4647	1033.4651	-0.39	0	30	0.0011	1	U	R.NECFLSHK.D
686	99 – 106	517.7397	1033.4648	1033.4651	-0.27	0	34	0.00041	1	U	R.NECFLSHK.D
687	99 – 106	517.7397	1033.4648	1033.4651	-0.27	0	34	0.00039	1	U	R.NECFLSHK.D
688	99 – 106	345.4956	1033.4649	1033.4651	-0.21	0	29	0.0011	1	U	R.NECFLSHK.D
689	99 – 106	517.7398	1033.4651	1033.4651	-0.034	0	34	0.0004	1	U	R.NECFLSHK.D
690	99 – 106	345.4956	1033.4651	1033.4651	-0.011	0	14	0.041	1	U	R.NECFLSHK.D
691	99 – 106	345.4957	1033.4653	1033.4651	0.16	0	34	0.00041	1	U	R.NECFLSHK.D
692	99 – 106	345.4958	1033.4655	1033.4651	0.34	0	26	0.0026	1	U	R.NECFLSHK.D
693	99 – 106	345.4958	1033.4655	1033.4651	0.34	0	27	0.002	1	U	R.NECFLSHK.D
694	99 – 106	517.7401	1033.4657	1033.4651	0.55	0	34	0.0004	1	U	R.NECFLSHK.D
695	99 – 106	345.4959	1033.4657	1033.4651	0.60	0	26	0.0023	1	U	R.NECFLSHK.D
696	99 – 106	345.4960	1033.4663	1033.4651	1.12	0	29	0.0012	1	U	R.NECFLSHK.D
697	99 – 106	517.7404	1033.4663	1033.4651	1.15	0	26	0.0026	1	U	R.NECFLSHK.D
3459	99 – 114	634.6276	1900.8610	1900.8625	-0.78	1	58	1.5e-006	1	U	R.NECFLSHKDDSPDLPK.L
3460	99 – 114	634.6277	1900.8614	1900.8625	-0.58	1	37	0.00022	1	U	R.NECFLSHKDDSPDLPK.L
3461	99 – 114	634.6278	1900.8616	1900.8625	-0.48	1	47	2.1e-005	1	U	R.NECFLSHKDDSPDLPK.L
3462	99 – 114	476.2227	1900.8618	1900.8625	-0.39	1	33	0.00046	1	U	R.NECFLSHKDDSPDLPK.L
3463	99 – 114	476.2228	1900.8620	1900.8625	-0.26	1	46	2.3e-005	1	U	R.NECFLSHKDDSPDLPK.L
3464	99 – 114	476.2228	1900.8620	1900.8625	-0.26	1	43	4.6e-005	1	U	R.NECFLSHKDDSPDLPK.L
3465	99 – 114	476.2228	1900.8621	1900.8625	-0.20	1	38	0.00015	1	U	R.NECFLSHKDDSPDLPK.L
3466	99 – 114	634.6280	1900.8621	1900.8625	-0.20	1	50	1e-005	1	U	R.NECFLSHKDDSPDLPK.L
3467	99 – 114	476.2229	1900.8625	1900.8625	-0.012	1	50	9.8e-006	1	U	R.NECFLSHKDDSPDLPK.L
3468	99 – 114	951.4388	1900.8631	1900.8625	0.32	1	74	4.2e-008	1	U	R.NECFLSHKDDSPDLPK.L
3469	99 – 114	476.2231	1900.8632	1900.8625	0.37	1	35	0.00031	1	U	R.NECFLSHKDDSPDLPK.L
3885	99 – 114	1032.4630	2062.9115	2062.9153	-1.88	1	52	5.7e-006	1	U	R.NECFLSHKDDSPDLPK.L + Glycation (K)
3886	99 – 114	688.6448	2062.9125	2062.9153	-1.37	1	39	0.00012	1	U	R.NECFLSHKDDSPDLPK.L + Glycation (K)
3887	99 – 114	688.6450	2062.9131	2062.9153	-1.11	1	28	0.0017	1	U	R.NECFLSHKDDSPDLPK.L + Glycation (K)
3888	99 – 114	688.6451	2062.9136	2062.9153	-0.85	1	32	0.00066	1	U	R.NECFLSHKDDSPDLPK.L + Glycation (K)
3889	99 – 114	688.6452	2062.9138	2062.9153	-0.76	1	23	0.0052	1	U	R.NECFLSHKDDSPDLPK.L + Glycation (K)
3890	99 – 114	688.6452	2062.9138	2062.9153	-0.76	1	43	5.5e-005	1	U	R.NECFLSHKDDSPDLPK.L + Glycation (K)
3891	99 – 114	516.7360	2062.9147	2062.9153	-0.29	1	31	0.00089	1	U	R.NECFLSHKDDSPDLPK.L + Glycation (K)
3892	99 – 114	688.6456	2062.9149	2062.9153	-0.22	1	37	0.00021	1	U	R.NECFLSHKDDSPDLPK.L + Glycation (K)
3893	99 – 114	516.7360	2062.9150	2062.9153	-0.18	1	27	0.0022	1	U	R.NECFLSHKDDSPDLPK.L + Glycation (K)
3894	99 – 114	516.7361	2062.9152	2062.9153	-0.062	1	27	0.0018	1	U	R.NECFLSHKDDSPDLPK.L + Glycation (K)
3895	99 – 114	516.7361	2062.9155	2062.9153	0.074	1	32	0.0007	1	U	R.NECFLSHKDDSPDLPK.L + Glycation (K)

Query	Start – End	Observed	Mr(expt)	Mr(calc)	ppm	M	Score	Expect	Rank	U	Peptide
3896	99 – 114	516.7361	2062.9155	2062.9153	0.074	1	36	0.00024	1	U	R.NECFLSHKDDSPDLPK.L + Glycation (K)
3897	99 – 114	516.7362	2062.9157	2062.9153	0.19	1	39	0.00012	1	U	R.NECFLSHKDDSPDLPK.L + Glycation (K)
5643	99 – 127	725.1401	3620.6643	3620.6651	-0.21	3	50	9.8e-006	1	U	R.NECFLSHKDDSPDLPKLKPDPNTLCDEFK.A + Gl
5644	99 – 127	906.1737	3620.6657	3620.6651	0.18	3	57	2.2e-006	1	U	R.NECFLSHKDDSPDLPKLKPDPNTLCDEFK.A + Gl
5727	99 – 127	757.5498	3782.7128	3782.7179	-1.36	3	36	0.00025	1	U	R.NECFLSHKDDSPDLPKLKPDPNTLCDEFK.A + 2
5728	99 – 127	946.6858	3782.7143	3782.7179	-0.95	3	52	5.9e-006	1	U	R.NECFLSHKDDSPDLPKLKPDPNTLCDEFK.A + 2
341	107 – 114	443.7111	885.4076	885.4080	-0.39	0	37	0.00018	1	U	K.DDSPDLPK.L
342	107 – 114	443.7111	885.4077	885.4080	-0.26	0	38	0.00014	1	U	K.DDSPDLPK.L
343	107 – 114	443.7112	885.4079	885.4080	-0.12	0	40	9e-005	1	U	K.DDSPDLPK.L
344	107 – 114	443.7112	885.4079	885.4080	-0.052	0	41	8.5e-005	1	U	K.DDSPDLPK.L
345	107 – 114	443.7113	885.4080	885.4080	0.084	0	43	4.8e-005	1	U	K.DDSPDLPK.L
346	107 – 114	443.7114	885.4082	885.4080	0.22	0	36	0.00023	1	U	K.DDSPDLPK.L
347	107 – 114	443.7114	885.4082	885.4080	0.22	0	42	5.7e-005	1	U	K.DDSPDLPK.L
348	107 – 114	443.7117	885.4088	885.4080	0.92	0	42	5.7e-005	1	U	K.DDSPDLPK.L
4684	107 – 127	869.4086	2605.2041	2605.2105	-2.48	2	83	5.5e-009	1	U	K.DDSPDLPKLKPDPNTLCDEFK.A + Glycation (
4685	107 – 127	869.4101	2605.2085	2605.2105	-0.79	2	83	4.6e-009	1	U	K.DDSPDLPKLKPDPNTLCDEFK.A + Glycation (
4686	107 – 127	1303.6116	2605.2086	2605.2105	-0.74	2	68	1.7e-007	1	U	K.DDSPDLPKLKPDPNTLCDEFK.A + Glycation (
4687	107 – 127	869.4102	2605.2087	2605.2105	-0.72	2	73	4.8e-008	1	U	K.DDSPDLPKLKPDPNTLCDEFK.A + Glycation (
4688	107 – 127	652.3095	2605.2089	2605.2105	-0.61	2	93	5.4e-010	1	U	K.DDSPDLPKLKPDPNTLCDEFK.A + Glycation (
4689	107 – 127	869.4104	2605.2094	2605.2105	-0.44	2	95	3.3e-010	1	U	K.DDSPDLPKLKPDPNTLCDEFK.A + Glycation (
4690	107 – 127	652.3100	2605.2109	2605.2105	0.14	2	65	3.3e-007	1	U	K.DDSPDLPKLKPDPNTLCDEFK.A + Glycation (
4691	107 – 127	652.3104	2605.2123	2605.2105	0.69	2	54	3.7e-006	1	U	K.DDSPDLPKLKPDPNTLCDEFK.A + Glycation (
4939	107 – 127	692.8221	2767.2592	2767.2634	-1.49	2	61	8.6e-007	1	U	K.DDSPDLPKLKPDPNTLCDEFK.A + 2 Glycatio
4940	107 – 127	923.4276	2767.2608	2767.2634	-0.91	2	54	4e-006	1	U	K.DDSPDLPKLKPDPNTLCDEFK.A + 2 Glycatio
4941	107 – 127	923.4276	2767.2610	2767.2634	-0.85	2	63	4.9e-007	1	U	K.DDSPDLPKLKPDPNTLCDEFK.A + 2 Glycatio
4942	107 – 127	692.8227	2767.2617	2767.2634	-0.61	2	69	1.3e-007	1	U	K.DDSPDLPKLKPDPNTLCDEFK.A + 2 Glycatio
4943	107 – 127	923.4282	2767.2627	2767.2634	-0.25	2	38	0.00015	1	U	K.DDSPDLPKLKPDPNTLCDEFK.A + 2 Glycatio
4944	107 – 127	692.8231	2767.2631	2767.2634	-0.078	2	64	4.2e-007	1	U	K.DDSPDLPKLKPDPNTLCDEFK.A + 2 Glycatio
4945	107 – 127	923.4323	2767.2751	2767.2634	4.25	2	59	1.2e-006	1	U	K.DDSPDLPKLKPDPNTLCDEFK.A + 2 Glycatio
5397	107 – 131	803.6217	3210.4577	3210.4650	-2.26	3	28	0.0017	1	U	K.DDSPDLPKLKPDPNTLCDEFKADEK.K + 2 Glyc
5463	107 – 131	844.1366	3372.5173	3372.5178	-0.14	3	24	0.0038	1	U	K.DDSPDLPKLKPDPNTLCDEFKADEK.K + 3 Glyc
2164	115 – 127	788.8866	1575.7586	1575.7603	-1.04	1	62	7e-007	1	U	K.LKPDPNTLCDEFK.A
2165	115 – 127	788.8867	1575.7589	1575.7603	-0.89	1	78	1.6e-008	1	U	K.LKPDPNTLCDEFK.A
2166	115 – 127	788.8868	1575.7590	1575.7603	-0.81	1	83	5.4e-009	1	U	K.LKPDPNTLCDEFK.A
2167	115 – 127	788.8868	1575.7591	1575.7603	-0.73	1	78	1.6e-008	1	U	K.LKPDPNTLCDEFK.A
2168	115 – 127	788.8869	1575.7592	1575.7603	-0.66	1	83	5.5e-009	1	U	K.LKPDPNTLCDEFK.A
2169	115 – 127	526.2606	1575.7599	1575.7603	-0.27	1	57	1.8e-006	1	U	K.LKPDPNTLCDEFK.A
2170	115 – 127	526.2607	1575.7602	1575.7603	-0.046	1	45	3.2e-005	1	U	K.LKPDPNTLCDEFK.A
2171	115 – 127	526.2607	1575.7602	1575.7603	-0.046	1	55	2.9e-006	1	U	K.LKPDPNTLCDEFK.A
2172	115 – 127	526.2607	1575.7602	1575.7603	-0.046	1	54	3.6e-006	1	U	K.LKPDPNTLCDEFK.A
2173	115 – 127	526.2607	1575.7604	1575.7603	0.069	1	45	3.2e-005	1	U	K.LKPDPNTLCDEFK.A
2174	115 – 127	526.2608	1575.7606	1575.7603	0.18	1	56	2.4e-006	1	U	K.LKPDPNTLCDEFK.A
2175	115 – 127	526.2609	1575.7610	1575.7603	0.43	1	56	2.3e-006	1	U	K.LKPDPNTLCDEFK.A
2176	115 – 127	788.8878	1575.7611	1575.7603	0.51	1	65	3.2e-007	1	U	K.LKPDPNTLCDEFK.A
2720	115 – 127	869.9125	1737.8105	1737.8131	-1.49	1	41	7.3e-005	1	U	K.LKPDPNTLCDEFK.A + Glycation (K)
2721	115 – 127	869.9128	1737.8110	1737.8131	-1.21	1	39	0.00012	1	U	K.LKPDPNTLCDEFK.A + Glycation (K)
2722	115 – 127	869.9129	1737.8112	1737.8131	-1.07	1	50	1.1e-005	1	U	K.LKPDPNTLCDEFK.A + Glycation (K)
2723	115 – 127	869.9130	1737.8114	1737.8131	-1.00	1	38	0.00016	1	U	K.LKPDPNTLCDEFK.A + Glycation (K)
2724	115 – 127	869.9130	1737.8115	1737.8131	-0.93	1	46	2.7e-005	1	U	K.LKPDPNTLCDEFK.A + Glycation (K)
2725	115 – 127	580.2779	1737.8118	1737.8131	-0.73	1	42	6.5e-005	1	U	K.LKPDPNTLCDEFK.A + Glycation (K)
2726	115 – 127	580.2783	1737.8130	1737.8131	-0.091	1	42	6.1e-005	1	U	K.LKPDPNTLCDEFK.A + Glycation (K)
2727	115 – 127	580.2783	1737.8130	1737.8131	-0.091	1	42	5.9e-005	1	U	K.LKPDPNTLCDEFK.A + Glycation (K)
2728	115 – 127	580.2783	1737.8130	1737.8131	-0.091	1	41	7.2e-005	1	U	K.LKPDPNTLCDEFK.A + Glycation (K)
2729	115 – 127	580.2784	1737.8133	1737.8131	0.12	1	39	0.00014	1	U	K.LKPDPNTLCDEFK.A + Glycation (K)
2730	115 – 127	580.2784	1737.8133	1737.8131	0.12	1	43	5e-005	1	U	K.LKPDPNTLCDEFK.A + Glycation (K)
4040	115 – 131	728.0103	2181.0089	2181.0147	-2.65	2	43	5.1e-005	1	U	K.LKPDPNTLCDEFKADEK.K + Glycation (K)
4041	115 – 131	728.0113	2181.0122	2181.0147	-1.14	2	50	1.1e-005	1	U	K.LKPDPNTLCDEFKADEK.K + Glycation (K)
4042	115 – 131	728.0115	2181.0128	2181.0147	-0.88	2	32	0.00064	1	U	K.LKPDPNTLCDEFKADEK.K + Glycation (K)
4043	115 – 131	728.0122	2181.0146	2181.0147	-0.040	2	45	3.1e-005	1	U	K.LKPDPNTLCDEFKADEK.K + Glycation (K)
4343	115 – 131	782.0302	2343.0686	2343.0675	0.46	2	42	6.2e-005	1	U	K.LKPDPNTLCDEFKADEK.K + 2 Glycation (K)
4589	128 – 143	835.0748	2502.2027	2502.2053	-1.06	3	38	0.00017	1	U	K.ADEKKFWGKYLYEIAR.R + 3 Glycation (K)
4590	128 – 143	835.0753	2502.2041	2502.2053	-0.48	3	35	0.00029	1	U	K.ADEKKFWGKYLYEIAR.R + 3 Glycation (K)
4592	128 – 143	626.5584	2502.2045	2502.2053	-0.31	3	34	0.0004	1	U	K.ADEKKFWGKYLYEIAR.R + 3 Glycation (K)
4593	128 – 143	626.5585	2502.2048	2502.2053	-0.22	3	47	2e-005	1	U	K.ADEKKFWGKYLYEIAR.R + 3 Glycation (K)
4594	128 – 143	626.5585	2502.2050	2502.2053	-0.12	3	47	1.9e-005	1	U	K.ADEKKFWGKYLYEIAR.R + 3 Glycation (K)
4595	128 – 143	626.5586	2502.2055	2502.2053	0.070	3	44	4.3e-005	1	U	K.ADEKKFWGKYLYEIAR.R + 3 Glycation (K)
2710	132 – 143	868.4556	1734.8966	1734.8981	-0.85	2	69	1.3e-007	1	U	K.KFWGKYLYEIAR.R + Glycation (K)
2711	132 – 143	434.7315	1734.8967	1734.8981	-0.79	2	17	0.02	1	U	K.KFWGKYLYEIAR.R + Glycation (K)
2712	132 – 143	868.4556	1734.8967	1734.8981	-0.79	2	56	2.2e-006	1	U	K.KFWGKYLYEIAR.R + Glycation (K)
2713	132 – 143	579.3063	1734.8970	1734.8981	-0.63	2	46	2.4e-005	1	U	K.KFWGKYLYEIAR.R + Glycation (K)
2714	132 – 143	579.3065	1734.8976	1734.8981	-0.30	2	53	5.1e-006	1	U	K.KFWGKYLYEIAR.R + Glycation (K)
2715	132 – 143	579.3065	1734.8977	1734.8981	-0.20	2	61	8e-007	1	U	K.KFWGKYLYEIAR.R + Glycation (K)
2716	132 – 143	579.3066	1734.8979	1734.8981	-0.092	2	53	4.9e-006	1	U	K.KFWGKYLYEIAR.R + Glycation (K)
2717	132 – 143	434.7319	1734.8984	1734.8981	0.18	2	22	0.0065	1	U	K.KFWGKYLYEIAR.R + Glycation (K)
2718	132 – 143	434.7321	1734.8991	1734.8981	0.61	2	23	0.0054	1	U	K.KFWGKYLYEIAR.R + Glycation (K)
3450	132 – 143	949.4809	1896.9473	1896.9509	-1.89	2	44	4.3e-005	1	U	K.KFWGKYLYEIAR.R + 2 Glycation (K)
3451	132 – 143	949.4818	1896.9489	1896.9509	-1.03	2	47	1.9e-005	1	U	K.KFWGKYLYEIAR.R + 2 Glycation (K)
3452	132 – 143	633.3242	1896.9507	1896.9509	-0.13	2	38	0.00015	1	U	K.KFWGKYLYEIAR.R + 2 Glycation (K)
3453	132 – 143	633.3242	1896.9507	1896.9509	-0.13	2	37	0.00018	1	U	K.KFWGKYLYEIAR.R + 2 Glycation (K)
3454	132 – 143	633.3242	1896.9507	1896.9509	-0.13	2	36	0.00028	1	U	K.KFWGKYLYEIAR.R + 2 Glycation (K)
3455	132 – 143	633.3243	1896.9510	1896.9509	0.061	2	37	0.00018	1	U	K.KFWGKYLYEIAR.R + 2 Glycation (K)
3456	132 – 143	475.2451	1896.9511	1896.9509	0.13	2	27	0.0019	1	U	K.KFWGKYLYEIAR.R + 2 Glycation (K)
3457	132 – 143	949.4847	1896.9549	1896.9509	2.12	2	30	0.0009	1	U	K.KFWGKYLYEIAR.R + 2 Glycation (K)
2243	133 – 143	804.4078	1606.8010	1606.8031	-1.31	1	43	5.1e-005	1	U	K.FWGKYLYEIAR.R + Glycation (K)
2244	133 – 143	804.4079	1606.8012	1606.8031	-1.16	1	46	2.4e-005	1	U	K.FWGKYLYEIAR.R + Glycation (K)
2245	133 – 143	804.4083	1606.8020	1606.8031	-0.71	1	47	2e-005	1	U	K.FWGKYLYEIAR.R + Glycation (K)
2246	133 – 143	804.4083	1606.8020	1606.8031	-0.71	1	47	2e-005	1	U	K.FWGKYLYEIAR.R + Glycation (K)

Query	Start - End	Observed	Mr(expt)	Mr(calc)	ppm	M	Score	Expect	Rank	U	Peptide
2247	133 - 143	536.6082	1606.8028	1606.8031	-0.18	1	29	0.0014	1	U	K.FWGKYLYEIAR.R + Glycation (K)
2248	133 - 143	536.6082	1606.8028	1606.8031	-0.18	1	40	0.00011	1	U	K.FWGKYLYEIAR.R + Glycation (K)
2249	133 - 143	536.6085	1606.8036	1606.8031	0.27	1	37	0.00018	1	U	K.FWGKYLYEIAR.R + Glycation (K)
2250	133 - 143	536.6085	1606.8037	1606.8031	0.38	1	26	0.0028	1	U	K.FWGKYLYEIAR.R + Glycation (K)
2251	133 - 143	536.6086	1606.8039	1606.8031	0.50	1	39	0.00011	1	U	K.FWGKYLYEIAR.R + Glycation (K)
422	137 - 143	464.2501	926.4857	926.4861	-0.48	0	30	0.00093	1	U	K.YLYEIAR.R
423	137 - 143	464.2502	926.4858	926.4861	-0.35	0	28	0.0016	1	U	K.YLYEIAR.R
424	137 - 143	464.2503	926.4861	926.4861	-0.0022	0	31	0.00082	1	U	K.YLYEIAR.R
425	137 - 143	464.2504	926.4862	926.4861	0.063	0	29	0.0013	1	U	K.YLYEIAR.R
426	137 - 143	464.2505	926.4864	926.4861	0.26	0	28	0.0015	1	U	K.YLYEIAR.R
427	137 - 143	464.2505	926.4865	926.4861	0.45	0	26	0.0026	1	U	K.YLYEIAR.R
428	137 - 143	464.2507	926.4868	926.4861	0.71	0	20	0.0095	1	U	K.YLYEIAR.R
3866	144 - 159	1023.0168	2044.0190	2044.0206	-0.79	1	38	0.00017	1	U	R.RHPYFYAPELLYYANK.Y
3867	144 - 159	1023.0171	2044.0197	2044.0206	-0.47	1	45	3e-005	1	U	R.RHPYFYAPELLYYANK.Y
3868	144 - 159	682.3472	2044.0197	2044.0206	-0.46	1	49	1.4e-005	1	U	R.RHPYFYAPELLYYANK.Y
3869	144 - 159	682.3472	2044.0199	2044.0206	-0.38	1	36	0.00027	1	U	R.RHPYFYAPELLYYANK.Y
3870	144 - 159	512.0124	2044.0207	2044.0206	0.032	1	38	0.00016	1	U	R.RHPYFYAPELLYYANK.Y
3871	144 - 159	682.3477	2044.0212	2044.0206	0.26	1	43	4.8e-005	1	U	R.RHPYFYAPELLYYANK.Y
3872	144 - 159	512.0126	2044.0214	2044.0206	0.38	1	47	2.1e-005	1	U	R.RHPYFYAPELLYYANK.Y
3873	144 - 159	512.0126	2044.0214	2044.0206	0.38	1	47	1.9e-005	1	U	R.RHPYFYAPELLYYANK.Y
3874	144 - 159	682.3484	2044.0235	2044.0206	1.41	1	40	0.00011	1	U	R.RHPYFYAPELLYYANK.Y
3875	144 - 159	1023.0191	2044.0237	2044.0206	1.50	1	33	0.0005	1	U	R.RHPYFYAPELLYYANK.Y
5764	144 - 173	984.6960	3934.7548	3934.7607	-1.49	2	74	3.6e-008	1	U	R.RHPYFYAPELLYYANKYNGVFQECCQAEDK.G + G
5765	144 - 173	984.6967	3934.7578	3934.7607	-0.73	2	73	4.6e-008	1	U	R.RHPYFYAPELLYYANKYNGVFQECCQAEDK.G + G
5766	144 - 173	787.9589	3934.7580	3934.7607	-0.67	2	68	1.6e-007	1	U	R.RHPYFYAPELLYYANKYNGVFQECCQAEDK.G + G
5767	144 - 173	787.9591	3934.7590	3934.7607	-0.43	2	67	2.1e-007	1	U	R.RHPYFYAPELLYYANKYNGVFQECCQAEDK.G + G
5768	144 - 173	1312.5941	3934.7605	3934.7607	-0.034	2	87	1.9e-009	1	U	R.RHPYFYAPELLYYANKYNGVFQECCQAEDK.G + G
5769	144 - 173	984.6980	3934.7629	3934.7607	0.57	2	67	1.9e-007	1	U	R.RHPYFYAPELLYYANKYNGVFQECCQAEDK.G + G
5770	144 - 173	984.6987	3934.7656	3934.7607	1.25	2	59	1.2e-006	1	U	R.RHPYFYAPELLYYANKYNGVFQECCQAEDK.G + G
5771	144 - 173	787.9606	3934.7668	3934.7607	1.55	2	70	1e-007	1	U	R.RHPYFYAPELLYYANKYNGVFQECCQAEDK.G + G
5772	144 - 173	1312.5969	3934.7689	3934.7607	2.10	2	78	1.7e-008	1	U	R.RHPYFYAPELLYYANKYNGVFQECCQAEDK.G + G
6198	144 - 180	1210.0590	4836.2067	4836.2186	-2.45	3	21	0.0074	1	U	R.RHPYFYAPELLYYANKYNGVFQECCQAEDKGACLL
6199	144 - 180	1210.0593	4836.2082	4836.2186	-2.14	3	47	2.2e-005	1	U	R.RHPYFYAPELLYYANKYNGVFQECCQAEDKGACLL
6200	144 - 180	1210.0621	4836.2194	4836.2186	0.18	3	42	6.9e-005	1	U	R.RHPYFYAPELLYYANKYNGVFQECCQAEDKGACLL
6201	144 - 180	968.2519	4836.2234	4836.2186	0.99	3	40	0.00011	1	U	R.RHPYFYAPELLYYANKYNGVFQECCQAEDKGACLL
6202	144 - 180	968.2523	4836.2252	4836.2186	1.38	3	32	0.00063	1	U	R.RHPYFYAPELLYYANKYNGVFQECCQAEDKGACLL
6203	144 - 180	968.2537	4836.2319	4836.2186	2.76	3	29	0.0012	1	U	R.RHPYFYAPELLYYANKYNGVFQECCQAEDKGACLL
6204	144 - 180	807.0483	4836.2460	4836.2186	5.68	3	26	0.0024	1	U	R.RHPYFYAPELLYYANKYNGVFQECCQAEDKGACLL
3383	145 - 159	944.9657	1887.9168	1887.9195	-1.42	0	55	3.4e-006	1	U	R.HPYFYAPELLYYANK.Y
3384	145 - 159	944.9658	1887.9170	1887.9195	-1.35	0	53	5.1e-006	1	U	R.HPYFYAPELLYYANK.Y
3385	145 - 159	944.9658	1887.9171	1887.9195	-1.29	0	46	2.3e-005	1	U	R.HPYFYAPELLYYANK.Y
3386	145 - 159	630.3135	1887.9186	1887.9195	-0.48	0	46	2.3e-005	1	U	R.HPYFYAPELLYYANK.Y
3387	145 - 159	630.3135	1887.9186	1887.9195	-0.48	0	48	1.5e-005	1	U	R.HPYFYAPELLYYANK.Y
3388	145 - 159	630.3137	1887.9193	1887.9195	-0.10	0	46	2.3e-005	1	U	R.HPYFYAPELLYYANK.Y
3389	145 - 159	944.9676	1887.9206	1887.9195	0.58	0	29	0.0012	1	U	R.HPYFYAPELLYYANK.Y
2756	160 - 173	874.3552	1746.6958	1746.6978	-1.15	0	66	2.8e-007	1	U	K.YNGVFQECCQAEDK.G
2757	160 - 173	874.3552	1746.6959	1746.6978	-1.08	0	73	5e-008	1	U	K.YNGVFQECCQAEDK.G
2758	160 - 173	874.3554	1746.6963	1746.6978	-0.86	0	47	1.8e-005	1	U	K.YNGVFQECCQAEDK.G
2759	160 - 173	874.3556	1746.6966	1746.6978	-0.65	0	61	8.2e-007	1	U	K.YNGVFQECCQAEDK.G
2760	160 - 173	874.3569	1746.6992	1746.6978	0.81	0	33	0.00049	1	U	K.YNGVFQECCQAEDK.G
4735	160 - 180	883.7219	2648.1439	2648.1557	-4.43	1	22	0.0067	1	U	K.YNGVFQECCQAEDKGACLLPK.I + Glycation (
4736	160 - 180	883.7240	2648.1502	2648.1557	-2.08	1	18	0.016	1	U	K.YNGVFQECCQAEDKGACLLPK.I + Glycation (
4738	160 - 180	883.7253	2648.1542	2648.1557	-0.56	1	24	0.0036	1	U	K.YNGVFQECCQAEDKGACLLPK.I + Glycation (
144	174 - 180	379.7147	757.4149	757.4156	-0.98	0	22	0.006	1	U	K.GACLLPK.I
145	174 - 180	379.7148	757.4150	757.4156	-0.83	0	32	0.00063	1	U	K.GACLLPK.I
146	174 - 180	379.7150	757.4154	757.4156	-0.32	0	32	0.00062	1	U	K.GACLLPK.I
147	174 - 180	379.7150	757.4154	757.4156	-0.25	0	23	0.0055	1	U	K.GACLLPK.I
148	174 - 180	379.7150	757.4154	757.4156	-0.25	0	19	0.012	1	U	K.GACLLPK.I
149	174 - 180	379.7150	757.4154	757.4156	-0.25	0	32	0.00063	1	U	K.GACLLPK.I
150	174 - 180	379.7151	757.4156	757.4156	-0.087	0	23	0.0054	1	U	K.GACLLPK.I
151	174 - 180	379.7151	757.4157	757.4156	0.071	0	23	0.0056	1	U	K.GACLLPK.I
2078	174 - 185	775.8984	1549.7822	1549.7844	-1.40	1	40	9.9e-005	1	U	K.GACLLPKIETMR.E + Glycation (K)
2079	174 - 185	775.8986	1549.7827	1549.7844	-1.09	1	53	5.2e-006	1	U	K.GACLLPKIETMR.E + Glycation (K)
2080	174 - 185	775.8986	1549.7827	1549.7844	-1.09	1	35	0.0003	1	U	K.GACLLPKIETMR.E + Glycation (K)
2081	174 - 185	517.6021	1549.7845	1549.7844	0.080	1	26	0.0025	1	U	K.GACLLPKIETMR.E + Glycation (K)
2082	174 - 185	517.6022	1549.7847	1549.7844	0.20	1	34	0.00043	1	U	K.GACLLPKIETMR.E + Glycation (K)
2083	174 - 185	517.6022	1549.7849	1549.7844	0.31	1	36	0.00023	1	U	K.GACLLPKIETMR.E + Glycation (K)
1433	199 - 208	453.2182	1356.6327	1356.6344	-1.23	1	21	0.0087	1	U	R.CASIQKFGER.A + Glycation (K)
1434	199 - 208	453.2182	1356.6329	1356.6344	-1.10	1	23	0.0048	1	U	R.CASIQKFGER.A + Glycation (K)
1435	199 - 208	679.3237	1356.6329	1356.6344	-1.07	1	34	0.00039	1	U	R.CASIQKFGER.A + Glycation (K)
1436	199 - 208	679.3238	1356.6331	1356.6344	-0.89	1	39	0.00012	1	U	R.CASIQKFGER.A + Glycation (K)
1437	199 - 208	453.2184	1356.6333	1356.6344	-0.77	1	22	0.0057	1	U	R.CASIQKFGER.A + Glycation (K)
1438	199 - 208	453.2184	1356.6334	1356.6344	-0.70	1	22	0.0058	1	U	R.CASIQKFGER.A + Glycation (K)
1439	199 - 208	679.3242	1356.6338	1356.6344	-0.43	1	36	0.00028	1	U	R.CASIQKFGER.A + Glycation (K)
1442	199 - 208	679.3245	1356.6344	1356.6344	0.010	1	37	0.00018	1	U	R.CASIQKFGER.A + Glycation (K)
1444	199 - 208	679.3247	1356.6349	1356.6344	0.38	1	37	0.00019	1	U	R.CASIQKFGER.A + Glycation (K)
1028	209 - 217	388.5515	1162.6328	1162.6346	-1.55	1	36	0.00027	1	U	R.ALKAWSVAR.L + Glycation (K)
1029	209 - 217	388.5519	1162.6340	1162.6346	-0.52	1	43	5e-005	1	U	R.ALKAWSVAR.L + Glycation (K)
1030	209 - 217	388.5520	1162.6341	1162.6346	-0.44	1	38	0.00017	1	U	R.ALKAWSVAR.L + Glycation (K)
1031	209 - 217	582.3245	1162.6344	1162.6346	-0.20	1	35	0.00029	1	U	R.ALKAWSVAR.L + Glycation (K)
81	212 - 217	345.1900	688.3654	688.3656	-0.37	0	16	0.026	1	U	K.AWSVAR.L
82	212 - 217	345.1900	688.3655	688.3656	-0.20	0	17	0.018	1	U	K.AWSVAR.L
83	212 - 217	345.1901	688.3656	688.3656	-0.026	0	16	0.026	1	U	K.AWSVAR.L
84	212 - 217	345.1902	688.3658	688.3656	0.26	0	16	0.025	1	U	K.AWSVAR.L
284	218 - 224	283.1726	846.4959	846.4963	-0.49	1	22	0.0063	1	U	R.LSQKFPK.A
285	218 - 224	283.1726	846.4961	846.4963	-0.27	1	18	0.016	1	U	R.LSQKFPK.A
599	218 - 224	505.2819	1008.5492	1008.5491	0.019	1	20	0.01	1	U	R.LSQKFPK.A + Glycation (K)
5218	218 - 239	1003.1995	3006.5767	3006.5788	-0.69	3	22	0.0064	1	U	R.LSQKFPKAEFVEVTKLVTDLTK.V + 3 Glycati

Query	Start - End	Observed	Mr(expt)	Mr(calc)	ppm	M	Score	Expect	Rank	U	Peptide
1740	222 - 232	486.2571	1455.7494	1455.7497	-0.20	1	32	0.00066	1	U	K.FPKAEFVEVTK.L + Glycation (K)
4399	222 - 239	797.0910	2388.2512	2388.2563	-2.17	2	78	1.4e-008	1	U	K.FPKAEFVEVTKLVTDLTK.V + 2 Glycation (K
4400	222 - 239	598.0709	2388.2543	2388.2563	-0.84	2	42	6.6e-005	1	U	K.FPKAEFVEVTKLVTDLTK.V + 2 Glycation (K
4401	222 - 239	797.0921	2388.2545	2388.2563	-0.79	2	78	1.5e-008	1	U	K.FPKAEFVEVTKLVTDLTK.V + 2 Glycation (K
4402	222 - 239	598.0712	2388.2556	2388.2563	-0.32	2	28	0.0018	1	U	K.FPKAEFVEVTKLVTDLTK.V + 2 Glycation (K
4403	222 - 239	598.0712	2388.2558	2388.2563	-0.22	2	40	0.00011	1	U	K.FPKAEFVEVTKLVTDLTK.V + 2 Glycation (K
4404	222 - 239	598.0713	2388.2561	2388.2563	-0.12	2	25	0.0031	1	U	K.FPKAEFVEVTKLVTDLTK.V + 2 Glycation (K
4405	222 - 239	598.0715	2388.2570	2388.2563	0.28	2	23	0.0049	1	U	K.FPKAEFVEVTKLVTDLTK.V + 2 Glycation (K
4406	222 - 239	797.0930	2388.2571	2388.2563	0.29	2	48	1.5e-005	1	U	K.FPKAEFVEVTKLVTDLTK.V + 2 Glycation (K
4407	222 - 239	1195.1361	2388.2577	2388.2563	0.57	2	48	1.6e-005	1	U	K.FPKAEFVEVTKLVTDLTK.V + 2 Glycation (K
4408	222 - 239	797.0934	2388.2583	2388.2563	0.82	2	51	7.6e-006	1	U	K.FPKAEFVEVTKLVTDLTK.V + 2 Glycation (K
4409	222 - 239	797.0938	2388.2594	2388.2563	1.29	2	54	4.2e-006	1	U	K.FPKAEFVEVTKLVTDLTK.V + 2 Glycation (K
4410	222 - 239	797.0939	2388.2600	2388.2563	1.51	2	22	0.0069	1	U	K.FPKAEFVEVTKLVTDLTK.V + 2 Glycation (K
412	225 - 232	461.7476	921.4806	921.4807	-0.19	0	31	0.00083	1	U	K.AEFVEVTK.L
413	225 - 232	461.7476	921.4806	921.4807	-0.19	0	39	0.00014	1	U	K.AEFVEVTK.L
414	225 - 232	461.7476	921.4806	921.4807	-0.12	0	38	0.00015	1	U	K.AEFVEVTK.L
415	225 - 232	461.7477	921.4808	921.4807	0.071	0	32	0.00062	1	U	K.AEFVEVTK.L
3240	225 - 239	927.9978	1853.9810	1853.9874	-3.42	1	37	0.00021	1	U	K.AEFVEVTKLVTDLTK.V + Glycation (K)
3241	225 - 239	927.9986	1853.9826	1853.9874	-2.55	1	36	0.00027	1	U	K.AEFVEVTKLVTDLTK.V + Glycation (K)
3242	225 - 239	927.9991	1853.9837	1853.9874	-1.96	1	55	3.5e-006	1	U	K.AEFVEVTKLVTDLTK.V + Glycation (K)
3243	225 - 239	927.9996	1853.9847	1853.9874	-1.44	1	40	9e-005	1	U	K.AEFVEVTKLVTDLTK.V + Glycation (K)
3244	225 - 239	619.0022	1853.9848	1853.9874	-1.41	1	41	7.5e-005	1	U	K.AEFVEVTKLVTDLTK.V + Glycation (K)
3245	225 - 239	927.9998	1853.9850	1853.9874	-1.30	1	68	1.4e-007	1	U	K.AEFVEVTKLVTDLTK.V + Glycation (K)
3246	225 - 239	928.0000	1853.9854	1853.9874	-1.04	1	68	1.7e-007	1	U	K.AEFVEVTKLVTDLTK.V + Glycation (K)
3248	225 - 239	928.0001	1853.9857	1853.9874	-0.91	1	85	3.3e-009	1	U	K.AEFVEVTKLVTDLTK.V + Glycation (K)
3249	225 - 239	928.0002	1853.9858	1853.9874	-0.85	1	62	5.8e-007	1	U	K.AEFVEVTKLVTDLTK.V + Glycation (K)
3250	225 - 239	928.0002	1853.9859	1853.9874	-0.78	1	23	0.0052	1	U	K.AEFVEVTKLVTDLTK.V + Glycation (K)
3251	225 - 239	619.0026	1853.9860	1853.9874	-0.73	1	26	0.0023	1	U	K.AEFVEVTKLVTDLTK.V + Glycation (K)
3253	225 - 239	928.0004	1853.9862	1853.9874	-0.64	1	16	0.028	1	U	K.AEFVEVTKLVTDLTK.V + Glycation (K)
3254	225 - 239	928.0004	1853.9863	1853.9874	-0.58	1	70	9.8e-008	1	U	K.AEFVEVTKLVTDLTK.V + Glycation (K)
3255	225 - 239	619.0027	1853.9864	1853.9874	-0.52	1	47	2.2e-005	1	U	K.AEFVEVTKLVTDLTK.V + Glycation (K)
3256	225 - 239	619.0029	1853.9868	1853.9874	-0.32	1	39	0.00013	1	U	K.AEFVEVTKLVTDLTK.V + Glycation (K)
3257	225 - 239	619.0029	1853.9870	1853.9874	-0.23	1	44	3.8e-005	1	U	K.AEFVEVTKLVTDLTK.V + Glycation (K)
3258	225 - 239	928.0009	1853.9871	1853.9874	-0.13	1	70	1.1e-007	1	U	K.AEFVEVTKLVTDLTK.V + Glycation (K)
3259	225 - 239	619.0031	1853.9873	1853.9874	-0.032	1	33	0.00046	1	U	K.AEFVEVTKLVTDLTK.V + Glycation (K)
3260	225 - 239	619.0031	1853.9873	1853.9874	-0.032	1	25	0.0033	1	U	K.AEFVEVTKLVTDLTK.V + Glycation (K)
3261	225 - 239	619.0032	1853.9877	1853.9874	0.16	1	38	0.00015	1	U	K.AEFVEVTKLVTDLTK.V + Glycation (K)
3262	225 - 239	619.0032	1853.9877	1853.9874	0.16	1	51	8e-006	1	U	K.AEFVEVTKLVTDLTK.V + Glycation (K)
3263	225 - 239	928.0013	1853.9880	1853.9874	0.34	1	78	1.5e-008	1	U	K.AEFVEVTKLVTDLTK.V + Glycation (K)
3264	225 - 239	928.0014	1853.9882	1853.9874	0.47	1	55	3.4e-006	1	U	K.AEFVEVTKLVTDLTK.V + Glycation (K)
3265	225 - 239	619.0034	1853.9883	1853.9874	0.47	1	51	7.9e-006	1	U	K.AEFVEVTKLVTDLTK.V + Glycation (K)
3266	225 - 239	619.0034	1853.9884	1853.9874	0.57	1	27	0.0018	1	U	K.AEFVEVTKLVTDLTK.V + Glycation (K)
3267	225 - 239	928.0016	1853.9886	1853.9874	0.67	1	53	4.7e-006	1	U	K.AEFVEVTKLVTDLTK.V + Glycation (K)
3268	225 - 239	928.0018	1853.9891	1853.9874	0.93	1	43	4.8e-005	1	U	K.AEFVEVTKLVTDLTK.V + Glycation (K)
3269	225 - 239	928.0018	1853.9891	1853.9874	0.93	1	62	6e-007	1	U	K.AEFVEVTKLVTDLTK.V + Glycation (K)
3270	225 - 239	619.0037	1853.9892	1853.9874	0.96	1	35	0.00035	1	U	K.AEFVEVTKLVTDLTK.V + Glycation (K)
3271	225 - 239	619.0039	1853.9899	1853.9874	1.36	1	41	8.3e-005	1	U	K.AEFVEVTKLVTDLTK.V + Glycation (K)
3272	225 - 239	619.0039	1853.9899	1853.9874	1.36	1	41	7.7e-005	1	U	K.AEFVEVTKLVTDLTK.V + Glycation (K)
3273	225 - 239	928.0023	1853.9900	1853.9874	1.40	1	47	2.2e-005	1	U	K.AEFVEVTKLVTDLTK.V + Glycation (K)
3274	225 - 239	619.0090	1854.0051	1853.9874	9.55	1	38	0.00016	1	U	K.AEFVEVTKLVTDLTK.V + Glycation (K)
4388	225 - 242	794.4279	2380.2618	2380.2625	-0.31	2	38	0.00016	1	U	K.AEFVEVTKLVTDLTKVHK.E + 2 Glycation (K
4389	225 - 242	477.0597	2380.2619	2380.2625	-0.24	2	20	0.01	1	U	K.AEFVEVTKLVTDLTKVHK.E + 2 Glycation (K
4391	225 - 242	596.0731	2380.2631	2380.2625	0.27	2	46	2.6e-005	1	U	K.AEFVEVTKLVTDLTKVHK.E + 2 Glycation (K
5914	225 - 256	1069.2459	4272.9543	4272.9600	-1.34	3	55	3.3e-006	1	U	K.AEFVEVTKLVTDLTKVHKECCHGDLLECADDR.A
5915	225 - 256	1069.2463	4272.9561	4272.9600	-0.93	3	56	2.6e-006	1	U	K.AEFVEVTKLVTDLTKVHKECCHGDLLECADDR.A
5916	225 - 256	855.5990	4272.9586	4272.9600	-0.33	3	71	7.4e-008	1	U	K.AEFVEVTKLVTDLTKVHKECCHGDLLECADDR.A
5917	225 - 256	1069.2470	4272.9587	4272.9600	-0.31	3	73	4.5e-008	1	U	K.AEFVEVTKLVTDLTKVHKECCHGDLLECADDR.A
5918	225 - 256	855.5992	4272.9598	4272.9600	-0.047	3	43	5.1e-005	1	U	K.AEFVEVTKLVTDLTKVHKECCHGDLLECADDR.A
5919	225 - 256	713.1673	4272.9601	4272.9600	0.029	3	68	1.5e-007	1	U	K.AEFVEVTKLVTDLTKVHKECCHGDLLECADDR.A
5920	225 - 256	713.1679	4272.9638	4272.9600	0.89	3	78	1.5e-008	1	U	K.AEFVEVTKLVTDLTKVHKECCHGDLLECADDR.A
5921	225 - 256	1069.2486	4272.9651	4272.9600	1.19	3	75	3.3e-008	1	U	K.AEFVEVTKLVTDLTKVHKECCHGDLLECADDR.A
5922	225 - 256	855.6006	4272.9666	4272.9600	1.53	3	34	0.0004	1	U	K.AEFVEVTKLVTDLTKVHKECCHGDLLECADDR.A
5923	225 - 256	1425.3336	4272.9790	4272.9600	4.45	3	51	7.1e-006	1	U	K.AEFVEVTKLVTDLTKVHKECCHGDLLECADDR.A
188	233 - 239	395.2391	788.4637	788.4644	-0.89	0	36	0.00027	1	U	K.LVTDLTK.V
189	233 - 239	395.2394	788.4642	788.4644	-0.21	0	35	0.00029	1	U	K.LVTDLTK.V
190	233 - 239	395.2394	788.4642	788.4644	-0.21	0	36	0.00027	1	U	K.LVTDLTK.V
191	233 - 239	395.2395	788.4644	788.4644	0.022	0	36	0.00027	1	U	K.LVTDLTK.V
192	233 - 239	395.2395	788.4644	788.4644	0.022	0	36	0.00027	1	U	K.LVTDLTK.V
5394	233 - 256	802.8663	3207.4360	3207.4370	-0.32	2	93	5.2e-010	1	U	K.LVTDLTKVHKECCHGDLLECADDR.A + 2 Glyca
5395	233 - 256	642.4947	3207.4374	3207.4370	0.11	2	43	4.6e-005	1	U	K.LVTDLTKVHKECCHGDLLECADDR.A + 2 Glyca
4164	240 - 256	455.9924	2274.9256	2274.9304	-2.09	1	30	0.00093	1	U	K.VHKECCHGDLLECADDR.A + Glycation (K)
4165	240 - 256	569.7388	2274.9262	2274.9304	-1.83	1	33	0.00055	1	U	K.VHKECCHGDLLECADDR.A + Glycation (K)
4166	240 - 256	759.3163	2274.9270	2274.9304	-1.48	1	140	1.1e-014	1	U	K.VHKECCHGDLLECADDR.A + Glycation (K)
4167	240 - 256	759.3164	2274.9272	2274.9304	-1.39	1	100	9.9e-011	1	U	K.VHKECCHGDLLECADDR.A + Glycation (K)
4168	240 - 256	759.3166	2274.9279	2274.9304	-1.07	1	154	4.4e-016	1	U	K.VHKECCHGDLLECADDR.A + Glycation (K)
4169	240 - 256	759.3168	2274.9285	2274.9304	-0.83	1	154	4.3e-016	1	U	K.VHKECCHGDLLECADDR.A + Glycation (K)
4170	240 - 256	569.7395	2274.9289	2274.9304	-0.65	1	40	0.00011	1	U	K.VHKECCHGDLLECADDR.A + Glycation (K)
4171	240 - 256	455.9931	2274.9291	2274.9304	-0.55	1	20	0.0099	1	U	K.VHKECCHGDLLECADDR.A + Glycation (K)
4172	240 - 256	569.7396	2274.9294	2274.9304	-0.44	1	43	5e-005	1	U	K.VHKECCHGDLLECADDR.A + Glycation (K)
4173	240 - 256	759.3171	2274.9294	2274.9304	-0.42	1	154	4.3e-016	1	U	K.VHKECCHGDLLECADDR.A + Glycation (K)
4174	240 - 256	569.7397	2274.9299	2274.9304	-0.21	1	35	0.00033	1	U	K.VHKECCHGDLLECADDR.A + Glycation (K)
4175	240 - 256	569.7397	2274.9299	2274.9304	-0.21	1	36	0.00023	1	U	K.VHKECCHGDLLECADDR.A + Glycation (K)
4176	240 - 256	455.9933	2274.9301	2274.9304	-0.13	1	28	0.0017	1	U	K.VHKECCHGDLLECADDR.A + Glycation (K)
4177	240 - 256	569.7398	2274.9301	2274.9304	-0.10	1	40	0.0001	1	U	K.VHKECCHGDLLECADDR.A + Glycation (K)
4178	240 - 256	1138.4724	2274.9303	2274.9304	-0.047	1	112	5.9e-012	1	U	K.VHKECCHGDLLECADDR.A + Glycation (K)
4179	240 - 256	455.9934	2274.9308	2274.9304	0.20	1	33	0.00048	1	U	K.VHKECCHGDLLECADDR.A + Glycation (K)

Query	Start – End	Observed	Mr(expt)	Mr(calc)	ppm	M	Score	Expect	Rank	U	Peptide
4180	240 – 256	1138.4728	2274.9310	2274.9304	0.28	1	117	2.2e-012	1	U	K.VHKECCHGDLLECADDR.A + Glycation (K)
4181	240 – 256	569.7402	2274.9316	2274.9304	0.53	1	39	0.00012	1	U	K.VHKECCHGDLLECADDR.A + Glycation (K)
2776	243 – 256	875.3339	1748.6532	1748.6553	-1.20	0	115	3.5e-012	1	U	K.ECCHGDLLECADDR.A
2777	243 – 256	583.8920	1748.6543	1748.6553	-0.57	0	68	1.6e-007	1	U	K.ECCHGDLLECADDR.A
2778	243 – 256	875.3345	1748.6544	1748.6553	-0.50	0	109	1.3e-011	1	U	K.ECCHGDLLECADDR.A
2779	243 – 256	875.3345	1748.6544	1748.6553	-0.50	0	101	8.6e-011	1	U	K.ECCHGDLLECADDR.A
2780	243 – 256	583.8921	1748.6544	1748.6553	-0.47	0	81	8e-009	1	U	K.ECCHGDLLECADDR.A
2781	243 – 256	583.8922	1748.6548	1748.6553	-0.26	0	68	1.4e-007	1	U	K.ECCHGDLLECADDR.A
2782	243 – 256	583.8925	1748.6556	1748.6553	0.17	0	68	1.5e-007	1	U	K.ECCHGDLLECADDR.A
2783	243 – 256	583.8926	1748.6561	1748.6553	0.48	0	81	8.1e-009	1	U	K.ECCHGDLLECADDR.A
3932	257 – 273	1052.4901	2102.9657	2102.9678	-0.99	1	65	3.5e-007	1	U	R.ADLAKYICDNQDTISSK.L + Glycation (K)
3933	257 – 273	701.9959	2102.9659	2102.9678	-0.88	1	76	2.4e-008	1	U	R.ADLAKYICDNQDTISSK.L + Glycation (K)
3934	257 – 273	701.9960	2102.9661	2102.9678	-0.80	1	95	3.5e-010	1	U	R.ADLAKYICDNQDTISSK.L + Glycation (K)
3935	257 – 273	701.9961	2102.9664	2102.9678	-0.63	1	95	3.5e-010	1	U	R.ADLAKYICDNQDTISSK.L + Glycation (K)
3936	257 – 273	701.9961	2102.9666	2102.9678	-0.54	1	81	8e-009	1	U	R.ADLAKYICDNQDTISSK.L + Glycation (K)
3937	257 – 273	701.9962	2102.9668	2102.9678	-0.44	1	89	1.1e-009	1	U	R.ADLAKYICDNQDTISSK.L + Glycation (K)
3938	257 – 273	1052.4907	2102.9669	2102.9678	-0.41	1	50	9e-006	1	U	R.ADLAKYICDNQDTISSK.L + Glycation (K)
3939	257 – 273	701.9964	2102.9674	2102.9678	-0.18	1	89	1.2e-009	1	U	R.ADLAKYICDNQDTISSK.L + Glycation (K)
3940	257 – 273	1052.4910	2102.9674	2102.9678	-0.18	1	58	1.6e-006	1	U	R.ADLAKYICDNQDTISSK.L + Glycation (K)
3941	257 – 273	1052.4910	2102.9674	2102.9678	-0.18	1	61	7.4e-007	1	U	R.ADLAKYICDNQDTISSK.L + Glycation (K)
3942	257 – 273	701.9966	2102.9679	2102.9678	0.073	1	75	3.2e-008	1	U	R.ADLAKYICDNQDTISSK.L + Glycation (K)
3943	257 – 273	701.9966	2102.9679	2102.9678	0.073	1	46	2.4e-005	1	U	R.ADLAKYICDNQDTISSK.L + Glycation (K)
3944	257 – 273	701.9971	2102.9696	2102.9678	0.86	1	95	3.4e-010	1	U	R.ADLAKYICDNQDTISSK.L + Glycation (K)
4599	257 – 275	836.4060	2506.1962	2506.1996	-1.36	2	36	0.00025	1	U	R.ADLAKYICDNQDTISSKLK.E + 2 Glycation
4600	257 – 275	836.4060	2506.1962	2506.1996	-1.36	2	40	0.0001	1	U	R.ADLAKYICDNQDTISSKLK.E + 2 Glycation
4601	257 – 275	627.5566	2506.1975	2506.1996	-0.86	2	17	0.022	1	U	R.ADLAKYICDNQDTISSKLK.E + 2 Glycation
4602	257 – 275	836.4068	2506.1986	2506.1996	-0.41	2	35	0.0003	1	U	R.ADLAKYICDNQDTISSKLK.E + 2 Glycation
4603	257 – 275	836.4071	2506.1995	2506.1996	-0.051	2	28	0.0016	1	U	R.ADLAKYICDNQDTISSKLK.E + 2 Glycation
4604	257 – 275	627.5572	2506.1999	2506.1996	0.12	2	18	0.014	1	U	R.ADLAKYICDNQDTISSKLK.E + 2 Glycation
5775	257 – 285	986.2151	3940.8313	3940.8367	-1.37	4	40	9.2e-005	1	U	R.ADLAKYICDNQDTISSKLKECCDKPLLEK.S + 3
5776	257 – 285	789.1736	3940.8315	3940.8367	-1.30	4	22	0.0065	1	U	R.ADLAKYICDNQDTISSKLKECCDKPLLEK.S + 3
5777	257 – 285	986.2168	3940.8380	3940.8367	0.34	4	29	0.0013	1	U	R.ADLAKYICDNQDTISSKLKECCDKPLLEK.S + 3
5778	257 – 285	789.1754	3940.8405	3940.8367	0.97	4	14	0.039	1	U	R.ADLAKYICDNQDTISSKLKECCDKPLLEK.S + 3
5779	257 – 285	1314.6213	3940.8422	3940.8367	1.41	4	17	0.018	1	U	R.ADLAKYICDNQDTISSKLKECCDKPLLEK.S + 3
5832	257 – 285	821.5836	4102.8814	4102.8895	-1.96	4	24	0.0037	1	U	R.ADLAKYICDNQDTISSKLKECCDKPLLEK.S + 4
1659	262 – 273	722.3240	1442.6334	1442.6347	-0.94	0	59	1.3e-006	1	U	K.YICDNQDTISSK.L
1660	262 – 273	722.3243	1442.6340	1442.6347	-0.51	0	85	2.9e-009	1	U	K.YICDNQDTISSK.L
1661	262 – 273	722.3245	1442.6344	1442.6347	-0.26	0	85	2.9e-009	1	U	K.YICDNQDTISSK.L
1662	262 – 273	722.3245	1442.6344	1442.6347	-0.26	0	85	3e-009	1	U	K.YICDNQDTISSK.L
1663	262 – 273	722.3247	1442.6349	1442.6347	0.082	0	75	2.8e-008	1	U	K.YICDNQDTISSK.L
1664	262 – 273	722.3248	1442.6351	1442.6347	0.25	0	65	3.2e-007	1	U	K.YICDNQDTISSK.L
3230	262 – 275	616.2955	1845.8646	1845.8666	-1.09	1	16	0.026	1	U	K.YICDNQDTISSKLK.E + Glycation (K)
3231	262 – 275	616.2961	1845.8664	1845.8666	-0.098	1	36	0.00028	1	U	K.YICDNQDTISSKLK.E + Glycation (K)
3232	262 – 275	616.2961	1845.8666	1845.8666		1	27	0.0022	1	U	K.YICDNQDTISSKLK.E + Glycation (K)
5428	262 – 285	657.1073	3280.5001	3280.5036	-1.08	3	26	0.0026	1	U	K.YICDNQDTISSKLKECCDKPLLEK.S + 2 Glyca
2016	274 – 285	511.5983	1531.7731	1531.7738	-0.48	2	58	1.6e-006	1	U	K.LKECCDKPLLEK.S
2017	274 – 285	383.9506	1531.7734	1531.7738	-0.28	2	35	0.00033	1	U	K.LKECCDKPLLEK.S
2018	274 – 285	766.8940	1531.7735	1531.7738	-0.18	2	49	1.3e-005	1	U	K.LKECCDKPLLEK.S
2019	274 – 285	383.9508	1531.7740	1531.7738	0.14	2	35	0.0003	1	U	K.LKECCDKPLLEK.S
2020	274 – 285	511.5987	1531.7742	1531.7738	0.23	2	54	3.9e-006	1	U	K.LKECCDKPLLEK.S
2557	274 – 285	565.6157	1693.8253	1693.8266	-0.77	2	63	4.6e-007	1	U	K.LKECCDKPLLEK.S + Glycation (K)
2558	274 – 285	424.4636	1693.8254	1693.8266	-0.74	2	68	1.6e-007	1	U	K.LKECCDKPLLEK.S + Glycation (K)
2559	274 – 285	847.9202	1693.8258	1693.8266	-0.50	2	45	3.2e-005	1	U	K.LKECCDKPLLEK.S + Glycation (K)
2560	274 – 285	847.9202	1693.8258	1693.8266	-0.50	2	45	3.1e-005	1	U	K.LKECCDKPLLEK.S + Glycation (K)
2561	274 – 285	424.4638	1693.8260	1693.8266	-0.36	2	73	5.1e-008	1	U	K.LKECCDKPLLEK.S + Glycation (K)
2562	274 – 285	565.6160	1693.8263	1693.8266	-0.22	2	50	9.7e-006	1	U	K.LKECCDKPLLEK.S + Glycation (K)
2563	274 – 285	565.6162	1693.8268	1693.8266	0.10	2	16	0.028	1	U	K.LKECCDKPLLEK.S + Glycation (K)
2564	274 – 285	565.6162	1693.8268	1693.8266	0.10	2	45	3.2e-005	1	U	K.LKECCDKPLLEK.S + Glycation (K)
2565	274 – 285	424.4640	1693.8269	1693.8266	0.13	2	53	5.1e-006	1	U	K.LKECCDKPLLEK.S + Glycation (K)
2566	274 – 285	424.4640	1693.8271	1693.8266	0.28	2	49	1.2e-005	1	U	K.LKECCDKPLLEK.S + Glycation (K)
2567	274 – 285	424.4640	1693.8271	1693.8266	0.28	2	28	0.0015	1	U	K.LKECCDKPLLEK.S + Glycation (K)
2568	274 – 285	565.6164	1693.8273	1693.8266	0.42	2	31	0.00074	1	U	K.LKECCDKPLLEK.S + Glycation (K)
3276	274 – 285	928.9459	1855.8772	1855.8795	-1.23	2	13	0.046	1	U	K.LKECCDKPLLEK.S + 2 Glycation (K)
3277	274 – 285	928.9459	1855.8772	1855.8795	-1.23	2	19	0.013	1	U	K.LKECCDKPLLEK.S + 2 Glycation (K)
3279	274 – 285	464.9768	1855.8783	1855.8795	-0.65	2	14	0.036	1	U	K.LKECCDKPLLEK.S + 2 Glycation (K)
3283	274 – 285	464.9771	1855.8795	1855.8795		2	16	0.023	1	U	K.LKECCDKPLLEK.S + 2 Glycation (K)
5133	274 – 294	728.3476	2909.3613	2909.3708	-3.26	3	31	0.00073	1	U	K.LKECCDKPLLEKSHCIAEVEK.D + 2 Glycatio
5134	274 – 294	582.8809	2909.3682	2909.3708	-0.88	3	15	0.03	1	U	K.LKECCDKPLLEKSHCIAEVEK.D + 2 Glycatio
5135	274 – 294	582.8809	2909.3682	2909.3708	-0.88	3	49	1.2e-005	1	U	K.LKECCDKPLLEKSHCIAEVEK.D + 2 Glycatio
5271	274 – 294	768.8621	3071.4194	3071.4236	-1.37	3	21	0.0078	1	U	K.LKECCDKPLLEKSHCIAEVEK.D + 3 Glycatio
5272	274 – 294	615.2923	3071.4251	3071.4236	0.50	3	49	1.4e-005	1	U	K.LKECCDKPLLEKSHCIAEVEK.D + 3 Glycatio
1270	276 – 285	431.2050	1290.5932	1290.5948	-1.21	1	37	0.00022	1	U	K.ECCDKPLLEK.S
1271	276 – 285	646.3043	1290.5941	1290.5948	-0.54	1	55	3.1e-006	1	U	K.ECCDKPLLEK.S
1272	276 – 285	646.3044	1290.5943	1290.5948	-0.36	1	61	7.4e-007	1	U	K.ECCDKPLLEK.S
1273	276 – 285	431.2054	1290.5944	1290.5948	-0.30	1	66	2.7e-007	1	U	K.ECCDKPLLEK.S
1274	276 – 285	431.2054	1290.5944	1290.5948	-0.30	1	54	4e-006	1	U	K.ECCDKPLLEK.S
1701	276 – 285	727.3297	1452.6449	1452.6476	-1.89	1	27	0.0019	1	U	K.ECCDKPLLEK.S + Glycation (K)
1703	276 – 285	727.3303	1452.6461	1452.6476	-1.05	1	27	0.0022	1	U	K.ECCDKPLLEK.S + Glycation (K)
1704	276 – 285	727.3307	1452.6469	1452.6476	-0.46	1	28	0.0017	1	U	K.ECCDKPLLEK.S + Glycation (K)
1706	276 – 285	485.2232	1452.6478	1452.6476	0.13	1	14	0.041	1	U	K.ECCDKPLLEK.S + Glycation (K)
1708	276 – 285	485.2233	1452.6480	1452.6476	0.25	1	20	0.011	1	U	K.ECCDKPLLEK.S + Glycation (K)
4597	276 – 294	627.5419	2506.1384	2506.1389	-0.22	2	49	1.2e-005	1	U	K.ECCDKPLLEKSHCIAEVEK.D + Glycation (K)
817	286 – 294	358.1743	1071.5011	1071.5019	-0.70	0	36	0.00025	1	U	K.SHCIAEVEK.D
818	286 – 294	358.1744	1071.5013	1071.5019	-0.53	0	39	0.00012	1	U	K.SHCIAEVEK.D
819	286 – 294	358.1744	1071.5014	1071.5019	-0.44	0	42	6.3e-005	1	U	K.SHCIAEVEK.D
820	286 – 294	358.1745	1071.5017	1071.5019	-0.19	0	39	0.00012	1	U	K.SHCIAEVEK.D
821	286 – 294	536.7581	1071.5017	1071.5019	-0.18	0	32	0.00061	1	U	K.SHCIAEVEK.D

Query	Start - End	Observed	Mr(expt)	Mr(calc)	ppm	M	Score	Expect	Rank	U	Peptide
822	286 - 294	536.7582	1071.5018	1071.5019	-0.064	0	45	3.2e-005	1	U	K.SHCIAEVEK.D
823	286 - 294	536.7582	1071.5018	1071.5019	-0.064	0	41	7.5e-005	1	U	K.SHCIAEVEK.D
824	286 - 294	536.7582	1071.5019	1071.5019	0.048	0	48	1.6e-005	1	U	K.SHCIAEVEK.D
5365	286 - 312	793.6297	3170.4897	3170.4965	-2.15	1	59	1.1e-006	1	U	K.SHCIAEVEKDAIPENLPPLTADFAEDK.D + Glyc
5366	286 - 312	1057.8378	3170.4917	3170.4965	-1.53	1	62	7e-007	1	U	K.SHCIAEVEKDAIPENLPPLTADFAEDK.D + Glyc
5367	286 - 312	1057.8395	3170.4966	3170.4965	0.033	1	14	0.04	1	U	K.SHCIAEVEKDAIPENLPPLTADFAEDK.D + Glyc
5368	286 - 312	793.6317	3170.4977	3170.4965	0.39	1	62	6.7e-007	1	U	K.SHCIAEVEKDAIPENLPPLTADFAEDK.D + Glyc
5369	286 - 312	1057.8401	3170.4984	3170.4965	0.61	1	46	2.3e-005	1	U	K.SHCIAEVEKDAIPENLPPLTADFAEDK.D + Glyc
5565	286 - 316	878.6730	3510.6630	3510.6647	-0.47	2	82	6e-009	1	U	K.SHCIAEVEKDAIPENLPPLTADFAEDKDVCK.N
5654	286 - 316	919.1860	3672.7151	3672.7175	-0.66	2	60	9.4e-007	1	U	K.SHCIAEVEKDAIPENLPPLTADFAEDKDVCK.N +
5655	286 - 316	735.5503	3672.7154	3672.7175	-0.58	2	49	1.3e-005	1	U	K.SHCIAEVEKDAIPENLPPLTADFAEDKDVCK.N +
5656	286 - 316	919.1864	3672.7165	3672.7175	-0.27	2	73	5.4e-008	1	U	K.SHCIAEVEKDAIPENLPPLTADFAEDKDVCK.N +
5657	286 - 316	735.5507	3672.7172	3672.7175	-0.072	2	39	0.00011	1	U	K.SHCIAEVEKDAIPENLPPLTADFAEDKDVCK.N +
5658	286 - 316	1225.2471	3672.7194	3672.7175	0.52	2	53	4.7e-006	1	U	K.SHCIAEVEKDAIPENLPPLTADFAEDKDVCK.N +
5659	286 - 316	1225.2476	3672.7211	3672.7175	0.98	2	65	2.8e-007	1	U	K.SHCIAEVEKDAIPENLPPLTADFAEDKDVCK.N +
5660	286 - 316	1225.2490	3672.7252	3672.7175	2.11	2	55	3.3e-006	1	U	K.SHCIAEVEKDAIPENLPPLTADFAEDKDVCK.N +
5735	286 - 316	767.9593	3834.7601	3834.7703	-2.67	2	31	0.00085	1	U	K.SHCIAEVEKDAIPENLPPLTADFAEDKDVCK.N +
5736	286 - 316	959.6979	3834.7627	3834.7703	-2.00	2	57	2e-006	1	U	K.SHCIAEVEKDAIPENLPPLTADFAEDKDVCK.N +
5737	286 - 316	1279.2626	3834.7659	3834.7703	-1.15	2	57	2.1e-006	1	U	K.SHCIAEVEKDAIPENLPPLTADFAEDKDVCK.N +
5738	286 - 316	959.6988	3834.7661	3834.7703	-1.11	2	57	2.1e-006	1	U	K.SHCIAEVEKDAIPENLPPLTADFAEDKDVCK.N +
5739	286 - 316	959.6992	3834.7678	3834.7703	-0.66	2	52	5.7e-006	1	U	K.SHCIAEVEKDAIPENLPPLTADFAEDKDVCK.N +
5740	286 - 316	959.6995	3834.7687	3834.7703	-0.41	2	25	0.0034	1	U	K.SHCIAEVEKDAIPENLPPLTADFAEDKDVCK.N +
6077	286 - 322	762.3588	4568.1093	4568.1098	-0.10	3	14	0.039	1	U	K.SHCIAEVEKDAIPENLPPLTADFAEDKDVCKNYQE
6078	286 - 322	1143.0346	4568.1094	4568.1098	-0.081	3	20	0.0094	1	U	K.SHCIAEVEKDAIPENLPPLTADFAEDKDVCKNYQE
6154	286 - 322	789.3666	4730.1558	4730.1626	-1.44	3	19	0.013	1	U	K.SHCIAEVEKDAIPENLPPLTADFAEDKDVCKNYQE
6157	286 - 322	1183.5478	4730.1623	4730.1626	-0.067	3	17	0.02	1	U	K.SHCIAEVEKDAIPENLPPLTADFAEDKDVCKNYQE
6158	286 - 322	1183.5482	4730.1638	4730.1626	0.25	3	21	0.0088	1	U	K.SHCIAEVEKDAIPENLPPLTADFAEDKDVCKNYQE
6323	286 - 335	1047.4845	6278.8633	6278.8875	-3.85	4	42	6.1e-005	1	U	K.SHCIAEVEKDAIPENLPPLTADFAEDKDVCKNYQE
6324	286 - 335	1047.4899	6278.8956	6278.8875	1.28	4	46	2.6e-005	1	U	K.SHCIAEVEKDAIPENLPPLTADFAEDKDVCKNYQE
6325	286 - 335	1256.7865	6278.8963	6278.8875	1.41	4	31	0.00083	1	U	K.SHCIAEVEKDAIPENLPPLTADFAEDKDVCKNYQE
6326	286 - 335	1256.7866	6278.8966	6278.8875	1.45	4	24	0.0039	1	U	K.SHCIAEVEKDAIPENLPPLTADFAEDKDVCKNYQE
6336	286 - 335	1289.1879	6440.9030	6440.9403	-5.80	4	27	0.0019	1	U	K.SHCIAEVEKDAIPENLPPLTADFAEDKDVCKNYQE
6337	286 - 335	1074.4966	6440.9358	6440.9403	-0.70	4	30	0.001	1	U	K.SHCIAEVEKDAIPENLPPLTADFAEDKDVCKNYQE
6338	286 - 335	1074.4980	6440.9446	6440.9403	0.66	4	47	1.9e-005	1	U	K.SHCIAEVEKDAIPENLPPLTADFAEDKDVCKNYQE
6339	286 - 335	1289.1968	6440.9475	6440.9403	1.12	4	34	0.00042	1	U	K.SHCIAEVEKDAIPENLPPLTADFAEDKDVCKNYQE
6340	286 - 335	1289.1993	6440.9603	6440.9403	3.10	4	26	0.0027	1	U	K.SHCIAEVEKDAIPENLPPLTADFAEDKDVCKNYQE
3639	295 - 312	978.4831	1954.9516	1954.9524	-0.38	0	71	7.1e-008	1	U	K.DAIPENLPPLTADFAEDK.D
3640	295 - 312	978.4838	1954.9531	1954.9524	0.38	0	71	7.5e-008	1	U	K.DAIPENLPPLTADFAEDK.D
3641	295 - 312	978.4841	1954.9537	1954.9524	0.69	0	59	1.1e-006	1	U	K.DAIPENLPPLTADFAEDK.D
4526	295 - 316	820.0642	2457.1708	2457.1733	-1.03	1	73	5.2e-008	1	U	K.DAIPENLPPLTADFAEDKDVCK.N
4527	295 - 316	820.0644	2457.1715	2457.1733	-0.74	1	61	8.4e-007	1	U	K.DAIPENLPPLTADFAEDKDVCK.N
4528	295 - 316	1229.5931	2457.1717	2457.1733	-0.66	1	74	3.6e-008	1	U	K.DAIPENLPPLTADFAEDKDVCK.N
4529	295 - 316	820.0657	2457.1754	2457.1733	0.82	1	61	8.2e-007	1	U	K.DAIPENLPPLTADFAEDKDVCK.N
4530	295 - 316	1229.5959	2457.1773	2457.1733	1.63	1	79	1.4e-008	1	U	K.DAIPENLPPLTADFAEDKDVCK.N
4701	295 - 316	874.0821	2619.2244	2619.2262	-0.66	1	56	2.5e-006	1	U	K.DAIPENLPPLTADFAEDKDVCK.N + Glycation
4702	295 - 316	874.0821	2619.2244	2619.2262	-0.66	1	58	1.7e-006	1	U	K.DAIPENLPPLTADFAEDKDVCK.N + Glycation
4703	295 - 316	874.0821	2619.2246	2619.2262	-0.59	1	56	2.5e-006	1	U	K.DAIPENLPPLTADFAEDKDVCK.N + Glycation
4704	295 - 316	1310.6198	2619.2249	2619.2262	-0.47	1	40	0.00011	1	U	K.DAIPENLPPLTADFAEDKDVCK.N + Glycation
4705	295 - 316	874.0836	2619.2290	2619.2262	1.09	1	21	0.0075	1	U	K.DAIPENLPPLTADFAEDKDVCK.N + Glycation
4706	295 - 316	874.0840	2619.2301	2619.2262	1.51	1	40	9.8e-005	1	U	K.DAIPENLPPLTADFAEDKDVCK.N + Glycation
4707	295 - 316	1310.6226	2619.2306	2619.2262	1.68	1	36	0.00027	1	U	K.DAIPENLPPLTADFAEDKDVCK.N + Glycation
5450	295 - 322	839.1470	3352.5590	3352.5656	-1.98	2	24	0.0041	1	U	K.DAIPENLPPLTADFAEDKDVCKNYQEAK.D + Gly
5451	295 - 322	839.1478	3352.5620	3352.5656	-1.09	2	18	0.017	1	U	K.DAIPENLPPLTADFAEDKDVCKNYQEAK.D + Gly
5452	295 - 322	1118.5297	3352.5672	3352.5656	0.45	2	19	0.014	1	U	K.DAIPENLPPLTADFAEDKDVCKNYQEAK.D + Gly
5453	295 - 322	1118.5299	3352.5679	3352.5656	0.67	2	20	0.011	1	U	K.DAIPENLPPLTADFAEDKDVCKNYQEAK.D + Gly
6217	295 - 335	1013.6722	5063.3248	5063.3434	-3.66	3	34	0.00038	1	U	K.DAIPENLPPLTADFAEDKDVCKNYQEAKDAFLGSF
6218	295 - 335	1266.8419	5063.3386	5063.3434	-0.94	3	32	0.00056	1	U	K.DAIPENLPPLTADFAEDKDVCKNYQEAKDAFLGSF
6224	295 - 335	1046.0883	5225.4049	5225.3962	1.67	3	40	0.0001	1	U	K.DAIPENLPPLTADFAEDKDVCKNYQEAKDAFLGSF
6225	295 - 335	1307.3591	5225.4074	5225.3962	2.15	3	31	0.00083	1	U	K.DAIPENLPPLTADFAEDKDVCKNYQEAKDAFLGSF
6226	295 - 335	1046.0889	5225.4080	5225.3962	2.26	3	40	0.0001	1	U	K.DAIPENLPPLTADFAEDKDVCKNYQEAKDAFLGSF
6227	295 - 335	1046.0894	5225.4104	5225.3962	2.73	3	37	0.00019	1	U	K.DAIPENLPPLTADFAEDKDVCKNYQEAKDAFLGSF
1590	313 - 322	472.8818	1415.6237	1415.6238	-0.10	1	23	0.0052	1	U	K.DVCKNYQEAK.D + Glycation (K)
5333	313 - 335	1043.1437	3126.4092	3126.4016	2.45	2	62	6.4e-007	1	U	K.DVCKNYQEAKDAFLGSFLYEYSR.R + 2 Glycat
4531	317 - 335	821.7137	2462.1192	2462.1277	-3.47	1	75	3e-008	1	U	K.NYQEAKDAFLGSFLYEYSR.R + Glycation (K)
4532	317 - 335	1232.0692	2462.1239	2462.1277	-1.58	1	107	2.2e-011	1	U	K.NYQEAKDAFLGSFLYEYSR.R + Glycation (K)
4533	317 - 335	821.7153	2462.1242	2462.1277	-1.46	1	58	1.8e-006	1	U	K.NYQEAKDAFLGSFLYEYSR.R + Glycation (K)
4534	317 - 335	1232.0697	2462.1248	2462.1277	-1.18	1	108	1.6e-011	1	U	K.NYQEAKDAFLGSFLYEYSR.R + Glycation (K)
4535	317 - 335	821.7158	2462.1255	2462.1277	-0.93	1	80	9.3e-009	1	U	K.NYQEAKDAFLGSFLYEYSR.R + Glycation (K)
4536	317 - 335	1232.0701	2462.1256	2462.1277	-0.88	1	109	1.2e-011	1	U	K.NYQEAKDAFLGSFLYEYSR.R + Glycation (K)
4537	317 - 335	821.7161	2462.1264	2462.1277	-0.57	1	75	3e-008	1	U	K.NYQEAKDAFLGSFLYEYSR.R + Glycation (K)
4538	317 - 335	821.7161	2462.1266	2462.1277	-0.48	1	78	1.6e-008	1	U	K.NYQEAKDAFLGSFLYEYSR.R + Glycation (K)
4539	317 - 335	821.7161	2462.1266	2462.1277	-0.48	1	87	1.9e-009	1	U	K.NYQEAKDAFLGSFLYEYSR.R + Glycation (K)
4540	317 - 335	1232.0714	2462.1283	2462.1277	0.21	1	105	3.4e-011	1	U	K.NYQEAKDAFLGSFLYEYSR.R + Glycation (K)
4541	317 - 335	821.7175	2462.1306	2462.1277	1.15	1	75	2.9e-008	1	U	K.NYQEAKDAFLGSFLYEYSR.R + Glycation (K)
2126	323 - 335	784.3740	1566.7335	1566.7354	-1.25	0	68	1.6e-007	1	U	K.DAFLGSFLYEYSR.R
2127	323 - 335	784.3740	1566.7335	1566.7354	-1.25	0	76	2.5e-008	1	U	K.DAFLGSFLYEYSR.R
2128	323 - 335	784.3741	1566.7337	1566.7354	-1.08	0	82	6.7e-009	1	U	K.DAFLGSFLYEYSR.R
2129	323 - 335	784.3743	1566.7340	1566.7354	-0.93	0	65	3.2e-007	1	U	K.DAFLGSFLYEYSR.R
2130	323 - 335	784.3745	1566.7345	1566.7354	-0.62	0	72	6.8e-008	1	U	K.DAFLGSFLYEYSR.R
2131	323 - 335	784.3747	1566.7348	1566.7354	-0.39	0	72	6.9e-008	1	U	K.DAFLGSFLYEYSR.R
2132	323 - 335	784.3753	1566.7361	1566.7354	0.40	0	82	5.9e-009	1	U	K.DAFLGSFLYEYSR.R
2133	323 - 335	523.2527	1566.7362	1566.7354	0.51	0	25	0.0032	1	U	K.DAFLGSFLYEYSR.R
2134	323 - 335	784.3761	1566.7376	1566.7354	1.41	0	67	1.8e-007	1	U	K.DAFLGSFLYEYSR.R
1632	336 - 347	720.4079	1438.8012	1438.8045	-2.23	1	54	3.7e-006	1	U	R.RHPEYAVSVLLR.L
1633	336 - 347	720.4083	1438.8021	1438.8045	-1.63	1	54	3.7e-006	1	U	R.RHPEYAVSVLLR.L
1634	336 - 347	720.4087	1438.8028	1438.8045	-1.13	1	51	8.3e-006	1	U	R.RHPEYAVSVLLR.L
1635	336 - 347	720.4089	1438.8033	1438.8045	-0.79	1	67	2.2e-007	1	U	R.RHPEYAVSVLLR.L

Query	Start - End	Observed	Mr(expt)	Mr(calc)	ppm	M	Score	Expect	Rank	U	Peptide
1636	336 - 347	720.4089	1438.8033	1438.8045	-0.79	1	55	3.3e-006	1	U	R.RHPEYAVSVLLR.L
1637	336 - 347	720.4089	1438.8033	1438.8045	-0.79	1	55	3.1e-006	1	U	R.RHPEYAVSVLLR.L
1638	336 - 347	720.4089	1438.8033	1438.8045	-0.79	1	54	3.7e-006	1	U	R.RHPEYAVSVLLR.L
1639	336 - 347	720.4091	1438.8037	1438.8045	-0.54	1	54	3.8e-006	1	U	R.RHPEYAVSVLLR.L
1640	336 - 347	480.6085	1438.8038	1438.8045	-0.44	1	63	5.3e-007	1	U	R.RHPEYAVSVLLR.L
1641	336 - 347	480.6086	1438.8040	1438.8045	-0.32	1	54	4.4e-006	1	U	R.RHPEYAVSVLLR.L
1642	336 - 347	480.6086	1438.8041	1438.8045	-0.26	1	53	5.3e-006	1	U	R.RHPEYAVSVLLR.L
1643	336 - 347	480.6087	1438.8043	1438.8045	-0.13	1	48	1.6e-005	1	U	R.RHPEYAVSVLLR.L
1644	336 - 347	480.6087	1438.8043	1438.8045	-0.13	1	51	8.5e-006	1	U	R.RHPEYAVSVLLR.L
1645	336 - 347	480.6087	1438.8044	1438.8045	-0.067	1	63	5e-007	1	U	R.RHPEYAVSVLLR.L
1646	336 - 347	480.6088	1438.8045	1438.8045	-0.0049	1	63	5.1e-007	1	U	R.RHPEYAVSVLLR.L
1647	336 - 347	480.6088	1438.8046	1438.8045	0.079	1	46	2.5e-005	1	U	R.RHPEYAVSVLLR.L
1648	336 - 347	480.6088	1438.8047	1438.8045	0.14	1	50	1.1e-005	1	U	R.RHPEYAVSVLLR.L
1649	336 - 347	480.6089	1438.8048	1438.8045	0.20	1	57	2e-006	1	U	R.RHPEYAVSVLLR.L
1650	336 - 347	480.6089	1438.8048	1438.8045	0.20	1	63	4.9e-007	1	U	R.RHPEYAVSVLLR.L
1651	336 - 347	480.6089	1438.8048	1438.8045	0.27	1	54	4.2e-006	1	U	R.RHPEYAVSVLLR.L
1652	336 - 347	360.7085	1438.8049	1438.8045	0.30	1	45	3.3e-005	1	U	R.RHPEYAVSVLLR.L
1653	336 - 347	480.6091	1438.8056	1438.8045	0.77	1	60	9.8e-007	1	U	R.RHPEYAVSVLLR.L
1654	336 - 347	480.6092	1438.8058	1438.8045	0.95	1	63	5.1e-007	1	U	R.RHPEYAVSVLLR.L + Glycation (R)
1655	336 - 347	480.6094	1438.8065	1438.8045	1.41	1	63	5.1e-007	1	U	R.RHPEYAVSVLLR.L + Glycation (R)
1228	337 - 347	642.3580	1282.7014	1282.7034	-1.53	0	63	5.5e-007	1	U	R.HPEYAVSVLLR.L
1229	337 - 347	642.3584	1282.7022	1282.7034	-0.86	0	59	1.3e-006	1	U	R.HPEYAVSVLLR.L
1230	337 - 347	642.3588	1282.7030	1282.7034	-0.30	0	51	7.5e-006	1	U	R.HPEYAVSVLLR.L
1231	337 - 347	642.3588	1282.7030	1282.7034	-0.30	0	62	6.8e-007	1	U	R.HPEYAVSVLLR.L
1232	337 - 347	642.3588	1282.7031	1282.7034	-0.19	0	52	6.1e-006	1	U	R.HPEYAVSVLLR.L
1233	337 - 347	642.3588	1282.7031	1282.7034	-0.19	0	63	4.9e-007	1	U	R.HPEYAVSVLLR.L
1234	337 - 347	428.5750	1282.7031	1282.7034	-0.19	0	57	2.2e-006	1	U	R.HPEYAVSVLLR.L
1235	337 - 347	428.5750	1282.7032	1282.7034	-0.12	0	51	8.7e-006	1	U	R.HPEYAVSVLLR.L
1236	337 - 347	428.5750	1282.7033	1282.7034	-0.048	0	52	5.7e-006	1	U	R.HPEYAVSVLLR.L
1237	337 - 347	428.5751	1282.7034	1282.7034	0.022	0	59	1.2e-006	1	U	R.HPEYAVSVLLR.L
1238	337 - 347	428.5751	1282.7035	1282.7034	0.092	0	57	2.1e-006	1	U	R.HPEYAVSVLLR.L
1239	337 - 347	642.3591	1282.7036	1282.7034	0.18	0	61	8.3e-007	1	U	R.HPEYAVSVLLR.L
1240	337 - 347	642.3599	1282.7052	1282.7034	1.41	0	63	5.6e-007	1	U	R.HPEYAVSVLLR.L
1241	337 - 347	428.5759	1282.7059	1282.7034	1.96	0	57	2.2e-006	1	U	R.HPEYAVSVLLR.L
3715	348 - 362	659.6312	1975.8717	1975.8754	-1.90	1	54	3.7e-006	1	U	R.LAKEYEATLEECCAK.D + Glycation (K)
3716	348 - 362	659.6315	1975.8728	1975.8754	-1.34	1	46	2.5e-005	1	U	R.LAKEYEATLEECCAK.D + Glycation (K)
3717	348 - 362	988.9438	1975.8730	1975.8754	-1.21	1	70	9.3e-008	1	U	R.LAKEYEATLEECCAK.D + Glycation (K)
3718	348 - 362	988.9439	1975.8731	1975.8754	-1.15	1	82	7.1e-009	1	U	R.LAKEYEATLEECCAK.D + Glycation (K)
3719	348 - 362	988.9441	1975.8736	1975.8754	-0.90	1	70	9.1e-008	1	U	R.LAKEYEATLEECCAK.D + Glycation (K)
3720	348 - 362	659.6321	1975.8744	1975.8754	-0.51	1	55	3.5e-006	1	U	R.LAKEYEATLEECCAK.D + Glycation (K)
3721	348 - 362	659.6324	1975.8753	1975.8754	-0.035	1	66	2.3e-007	1	U	R.LAKEYEATLEECCAK.D + Glycation (K)
3722	348 - 362	659.6324	1975.8755	1975.8754	0.056	1	66	2.3e-007	1	U	R.LAKEYEATLEECCAK.D + Glycation (K)
3723	348 - 362	988.9451	1975.8756	1975.8754	0.089	1	69	1.4e-007	1	U	R.LAKEYEATLEECCAK.D + Glycation (K)
3724	348 - 362	659.6325	1975.8757	1975.8754	0.15	1	55	3.5e-006	1	U	R.LAKEYEATLEECCAK.D + Glycation (K)
3725	348 - 362	659.6326	1975.8759	1975.8754	0.24	1	51	8.7e-006	1	U	R.LAKEYEATLEECCAK.D + Glycation (K)
5661	348 - 375	735.7183	3673.5549	3673.5634	-2.30	2	23	0.0054	1	U	R.LAKEYEATLEECCAKDDPHACYSTVFDK.L + 2
5662	348 - 375	919.3971	3673.5593	3673.5634	-1.12	2	36	0.00024	1	U	R.LAKEYEATLEECCAKDDPHACYSTVFDK.L + 2
5663	348 - 375	735.7192	3673.5595	3673.5634	-1.04	2	30	0.00096	1	U	R.LAKEYEATLEECCAKDDPHACYSTVFDK.L + 2
5664	348 - 375	919.3972	3673.5598	3673.5634	-0.97	2	30	0.0009	1	U	R.LAKEYEATLEECCAKDDPHACYSTVFDK.L + 2
5665	348 - 375	919.3976	3673.5615	3673.5634	-0.52	2	23	0.0048	1	U	R.LAKEYEATLEECCAKDDPHACYSTVFDK.L + 2
5666	348 - 375	1225.5279	3673.5618	3673.5634	-0.43	2	41	8.1e-005	1	U	R.LAKEYEATLEECCAKDDPHACYSTVFDK.L + 2
5830	348 - 377	1020.2044	4076.7883	4076.7952	-1.69	3	18	0.017	1	U	R.LAKEYEATLEECCAKDDPHACYSTVFDKLK.H +
1923	351 - 362	751.8092	1501.6038	1501.6065	-1.74	0	55	2.9e-006	1	U	K.EYEATLEECCAK.D
1924	351 - 362	751.8099	1501.6052	1501.6065	-0.83	0	59	1.2e-006	1	U	K.EYEATLEECCAK.D
1925	351 - 362	751.8099	1501.6052	1501.6065	-0.83	0	72	6.7e-008	1	U	K.EYEATLEECCAK.D
1926	351 - 362	751.8101	1501.6056	1501.6065	-0.59	0	56	2.6e-006	1	U	K.EYEATLEECCAK.D
1927	351 - 362	751.8112	1501.6079	1501.6065	0.95	0	72	7e-008	1	U	K.EYEATLEECCAK.D
5390	351 - 375	800.8286	3199.2853	3199.2944	-2.83	1	24	0.0036	1	U	K.EYEATLEECCAKDDPHACYSTVFDK.L + Glycat
5391	351 - 375	1067.4360	3199.2863	3199.2944	-2.54	1	34	0.00043	1	U	K.EYEATLEECCAKDDPHACYSTVFDK.L + Glycat
5392	351 - 375	800.8293	3199.2883	3199.2944	-1.91	1	18	0.016	1	U	K.EYEATLEECCAKDDPHACYSTVFDK.L + Glycat
2096	363 - 375	777.8283	1553.6419	1553.6457	-2.40	0	32	0.00059	1	U	K.DDPHACYSTVFDK.L
2097	363 - 375	777.8295	1553.6444	1553.6457	-0.83	0	36	0.00024	1	U	K.DDPHACYSTVFDK.L
2098	363 - 375	777.8295	1553.6444	1553.6457	-0.83	0	43	4.9e-005	1	U	K.DDPHACYSTVFDK.L
2099	363 - 375	777.8296	1553.6446	1553.6457	-0.67	0	30	0.0011	1	U	K.DDPHACYSTVFDK.L
2100	363 - 375	777.8297	1553.6447	1553.6457	-0.60	0	45	3e-005	1	U	K.DDPHACYSTVFDK.L
2101	363 - 375	518.8893	1553.6460	1553.6457	0.22	0	45	3.3e-005	1	U	K.DDPHACYSTVFDK.L
2102	363 - 375	518.8893	1553.6460	1553.6457	0.22	0	54	3.7e-006	1	U	K.DDPHACYSTVFDK.L
2103	363 - 375	518.8895	1553.6468	1553.6457	0.70	0	65	3.4e-007	1	U	K.DDPHACYSTVFDK.L
3643	363 - 377	979.4445	1956.8744	1956.8775	-1.61	1	25	0.0031	1	U	K.DDPHACYSTVFDKLK.H + Glycation (K)
3644	363 - 377	979.4449	1956.8752	1956.8775	-1.17	1	20	0.01	1	U	K.DDPHACYSTVFDKLK.H + Glycation (K)
3645	363 - 377	979.4452	1956.8758	1956.8775	-0.86	1	26	0.0023	1	U	K.DDPHACYSTVFDKLK.H + Glycation (K)
3647	363 - 377	653.2994	1956.8765	1956.8775	-0.52	1	31	0.00085	1	U	K.DDPHACYSTVFDKLK.H + Glycation (K)
3648	363 - 377	653.2995	1956.8767	1956.8775	-0.43	1	30	0.001	1	U	K.DDPHACYSTVFDKLK.H + Glycation (K)
3649	363 - 377	653.2995	1956.8767	1956.8775	-0.43	1	31	0.00083	1	U	K.DDPHACYSTVFDKLK.H + Glycation (K)
3650	363 - 377	490.2266	1956.8771	1956.8775	-0.20	1	16	0.026	1	U	K.DDPHACYSTVFDKLK.H + Glycation (K)
3651	363 - 377	653.2997	1956.8772	1956.8775	-0.16	1	24	0.0039	1	U	K.DDPHACYSTVFDKLK.H + Glycation (K)
3653	363 - 377	653.2997	1956.8774	1956.8775	-0.064	1	33	0.00053	1	U	K.DDPHACYSTVFDKLK.H + Glycation (K)
3654	363 - 377	490.2267	1956.8776	1956.8775	0.050	1	18	0.017	1	U	K.DDPHACYSTVFDKLK.H + Glycation (K)
5491	363 - 388	1136.2137	3405.6194	3405.6286	-2.70	2	24	0.0038	1	U	K.DDPHACYSTVFDKLKHLVDEPQNLIK.Q + 2 Gly
5492	363 - 388	852.4131	3405.6235	3405.6286	-1.50	2	25	0.0035	1	U	K.DDPHACYSTVFDKLKHLVDEPQNLIK.Q + 2 Gly
5493	363 - 388	852.4131	3405.6235	3405.6286	-1.50	2	28	0.0014	1	U	K.DDPHACYSTVFDKLKHLVDEPQNLIK.Q + 2 Gly
5494	363 - 388	852.4139	3405.6263	3405.6286	-0.67	2	32	0.00063	1	U	K.DDPHACYSTVFDKLKHLVDEPQNLIK.Q + 2 Gly
5495	363 - 388	682.1326	3405.6265	3405.6286	-0.63	2	37	0.0002	1	U	K.DDPHACYSTVFDKLKHLVDEPQNLIK.Q + 2 Gly
5497	363 - 388	682.1331	3405.6289	3405.6286	0.089	2	29	0.0013	1	U	K.DDPHACYSTVFDKLKHLVDEPQNLIK.Q + 2 Gly
5498	363 - 388	1136.2172	3405.6297	3405.6286	0.30	2	14	0.042	1	U	K.DDPHACYSTVFDKLKHLVDEPQNLIK.Q + 2 Gly
5499	363 - 388	1136.2172	3405.6297	3405.6286	0.30	2	24	0.0041	1	U	K.DDPHACYSTVFDKLKHLVDEPQNLIK.Q + 2 Gly
6098	363 - 396	1155.2819	4617.0983	4617.1051	-1.46	3	37	0.0002	1	U	K.DDPHACYSTVFDKLKHLVDEPQNLIKQNCDQFEK.
6099	363 - 396	924.4271	4617.0992	4617.1051	-1.27	3	26	0.0028	1	U	K.DDPHACYSTVFDKLKHLVDEPQNLIKQNCDQFEK.

Query	Start - End	Observed	Mr(expt)	Mr(calc)	ppm	M	Score	Expect	Rank	U	Peptide
6100	363 - 396	924.4273	4617.1002	4617.1051	-1.06	3	32	0.0007	1	U	K.DDPHACYSTVFDKLKHLVDEPQNLIKQNCDQFEK.
6102	363 - 396	1155.2840	4617.1071	4617.1051	0.43	3	24	0.0044	1	U	K.DDPHACYSTVFDKLKHLVDEPQNLIKQNCDQFEK.
6103	363 - 396	1155.2845	4617.1089	4617.1051	0.83	3	24	0.0036	1	U	K.DDPHACYSTVFDKLKHLVDEPQNLIKQNCDQFEK.
6104	363 - 396	924.4306	4617.1166	4617.1051	2.50	3	29	0.0011	1	U	K.DDPHACYSTVFDKLKHLVDEPQNLIKQNCDQFEK.
6313	363 - 409	1248.9899	6239.9130	6239.9355	-3.61	4	35	0.00031	1	U	K.DDPHACYSTVFDKLKHLVDEPQNLIKQNCDQFEKI
6314	363 - 409	1040.9976	6239.9418	6239.9355	1.01	4	70	9.2e-008	1	U	K.DDPHACYSTVFDKLKHLVDEPQNLIKQNCDQFEKI
2616	376 - 388	854.9766	1707.9386	1707.9407	-1.23	1	37	0.0002	1	U	K.LKHLVDEPQNLIK.Q + Glycation (K)
2617	376 - 388	854.9770	1707.9394	1707.9407	-0.73	1	38	0.00018	1	U	K.LKHLVDEPQNLIK.Q + Glycation (K)
2618	376 - 388	570.3207	1707.9402	1707.9407	-0.27	1	37	0.00019	1	U	K.LKHLVDEPQNLIK.Q + Glycation (K)
2619	376 - 388	427.9923	1707.9403	1707.9407	-0.24	1	25	0.0031	1	U	K.LKHLVDEPQNLIK.Q + Glycation (K)
2620	376 - 388	570.3207	1707.9404	1707.9407	-0.17	1	26	0.0026	1	U	K.LKHLVDEPQNLIK.Q + Glycation (K)
2621	376 - 388	570.3207	1707.9404	1707.9407	-0.17	1	28	0.0017	1	U	K.LKHLVDEPQNLIK.Q + Glycation (K)
2622	376 - 388	427.9925	1707.9409	1707.9407	0.11	1	16	0.026	1	U	K.LKHLVDEPQNLIK.Q + Glycation (K)
2623	376 - 388	570.3210	1707.9411	1707.9407	0.26	1	37	0.0002	1	U	K.LKHLVDEPQNLIK.Q + Glycation (K)
2624	376 - 388	427.9927	1707.9415	1707.9407	0.48	1	20	0.011	1	U	K.LKHLVDEPQNLIK.Q + Glycation (K)
5147	376 - 396	730.8605	2919.4130	2919.4171	-1.41	2	46	2.3e-005	1	U	K.LKHLVDEPQNLIKQNCDQFEK.L + 2 Glycatio
5148	376 - 396	974.1450	2919.4131	2919.4171	-1.40	2	48	1.7e-005	1	U	K.LKHLVDEPQNLIKQNCDQFEK.L + 2 Glycatio
5149	376 - 396	974.1453	2919.4140	2919.4171	-1.09	2	52	5.8e-006	1	U	K.LKHLVDEPQNLIKQNCDQFEK.L + 2 Glycatio
5150	376 - 396	974.1454	2919.4143	2919.4171	-0.96	2	46	2.2e-005	1	U	K.LKHLVDEPQNLIKQNCDQFEK.L + 2 Glycatio
5151	376 - 396	730.8610	2919.4147	2919.4171	-0.83	2	40	9.3e-005	1	U	K.LKHLVDEPQNLIKQNCDQFEK.L + 2 Glycatio
5152	376 - 396	584.8903	2919.4152	2919.4171	-0.66	2	29	0.0013	1	U	K.LKHLVDEPQNLIKQNCDQFEK.L + 2 Glycatio
5153	376 - 396	584.8903	2919.4152	2919.4171	-0.66	2	29	0.0013	1	U	K.LKHLVDEPQNLIKQNCDQFEK.L + 2 Glycatio
5154	376 - 396	730.8611	2919.4155	2919.4171	-0.56	2	23	0.0045	1	U	K.LKHLVDEPQNLIKQNCDQFEK.L + 2 Glycatio
5155	376 - 396	730.8613	2919.4160	2919.4171	-0.40	2	48	1.6e-005	1	U	K.LKHLVDEPQNLIKQNCDQFEK.L + 2 Glycatio
6059	376 - 409	909.4540	4542.2338	4542.2476	-3.02	3	88	1.6e-009	1	U	K.LKHLVDEPQNLIKQNCDQFEKLGEYGFQNALIVR.
6060	376 - 409	758.0483	4542.2460	4542.2476	-0.34	3	33	0.00052	1	U	K.LKHLVDEPQNLIKQNCDQFEKLGEYGFQNALIVR.
6061	376 - 409	909.4581	4542.2543	4542.2476	1.48	3	88	1.7e-009	1	U	K.LKHLVDEPQNLIKQNCDQFEKLGEYGFQNALIVR.
6062	376 - 409	1136.5712	4542.2556	4542.2476	1.76	3	54	4.2e-006	1	U	K.LKHLVDEPQNLIKQNCDQFEKLGEYGFQNALIVR.
6063	376 - 409	1136.5721	4542.2595	4542.2476	2.62	3	61	7.5e-007	1	U	K.LKHLVDEPQNLIKQNCDQFEKLGEYGFQNALIVR.
1300	378 - 388	653.3612	1304.7079	1304.7088	-0.74	0	48	1.4e-005	1	U	K.HLVDEPQNLIK.Q
1301	378 - 388	653.3614	1304.7083	1304.7088	-0.37	0	47	2.1e-005	1	U	K.HLVDEPQNLIK.Q
1302	378 - 388	653.3614	1304.7083	1304.7088	-0.37	0	46	2.4e-005	1	U	K.HLVDEPQNLIK.Q
1303	378 - 388	435.9103	1304.7090	1304.7088	0.14	0	17	0.021	1	U	K.HLVDEPQNLIK.Q
1304	378 - 388	435.9103	1304.7090	1304.7088	0.14	0	48	1.6e-005	1	U	K.HLVDEPQNLIK.Q
1305	378 - 388	435.9103	1304.7091	1304.7088	0.21	0	36	0.00023	1	U	K.HLVDEPQNLIK.Q
4613	378 - 396	839.7344	2516.1815	2516.1853	-1.51	1	59	1.3e-006	1	U	K.HLVDEPQNLIKQNCDQFEK.L + Glycation (K)
4614	378 - 396	839.7347	2516.1824	2516.1853	-1.16	1	60	1.1e-006	1	U	K.HLVDEPQNLIKQNCDQFEK.L + Glycation (K)
4615	378 - 396	839.7351	2516.1833	2516.1853	-0.79	1	56	2.8e-006	1	U	K.HLVDEPQNLIKQNCDQFEK.L + Glycation (K)
4616	378 - 396	839.7351	2516.1833	2516.1853	-0.79	1	62	6.3e-007	1	U	K.HLVDEPQNLIKQNCDQFEK.L + Glycation (K)
4617	378 - 396	630.0532	2516.1838	2516.1853	-0.61	1	47	2e-005	1	U	K.HLVDEPQNLIKQNCDQFEK.L + Glycation (K)
4618	378 - 396	1259.0994	2516.1842	2516.1853	-0.44	1	70	1.1e-007	1	U	K.HLVDEPQNLIKQNCDQFEK.L + Glycation (K)
4619	378 - 396	630.0533	2516.1843	2516.1853	-0.42	1	58	1.6e-006	1	U	K.HLVDEPQNLIKQNCDQFEK.L + Glycation (K)
4620	378 - 396	630.0535	2516.1850	2516.1853	-0.11	1	58	1.7e-006	1	U	K.HLVDEPQNLIKQNCDQFEK.L + Glycation (K)
4621	378 - 396	1259.1000	2516.1854	2516.1853	0.042	1	55	3.5e-006	1	U	K.HLVDEPQNLIKQNCDQFEK.L + Glycation (K)
5858	378 - 409	828.8096	4139.0118	4139.0157	-0.95	2	76	2.7e-008	1	U	K.HLVDEPQNLIKQNCDQFEKLGEYGFQNALIVR.Y
5859	378 - 409	1035.7610	4139.0149	4139.0157	-0.21	2	79	1.2e-008	1	U	K.HLVDEPQNLIKQNCDQFEKLGEYGFQNALIVR.Y
5860	378 - 409	828.8104	4139.0157	4139.0157	0.00072	2	63	4.6e-007	1	U	K.HLVDEPQNLIKQNCDQFEKLGEYGFQNALIVR.Y
5861	378 - 409	1035.7612	4139.0158	4139.0157	0.024	2	71	7.7e-008	1	U	K.HLVDEPQNLIKQNCDQFEKLGEYGFQNALIVR.Y
5862	378 - 409	1035.7615	4139.0168	4139.0157	0.26	2	76	2.3e-008	1	U	K.HLVDEPQNLIKQNCDQFEKLGEYGFQNALIVR.Y
5863	378 - 409	1380.6802	4139.0187	4139.0157	0.72	2	47	2.1e-005	1	U	K.HLVDEPQNLIKQNCDQFEKLGEYGFQNALIVR.Y
5864	378 - 409	1035.7637	4139.0256	4139.0157	2.38	2	83	5.4e-009	1	U	K.HLVDEPQNLIKQNCDQFEKLGEYGFQNALIVR.Y
5865	378 - 409	828.8132	4139.0295	4139.0157	3.32	2	18	0.015	1	U	K.HLVDEPQNLIKQNCDQFEKLGEYGFQNALIVR.Y
4805	389 - 409	897.7592	2690.2558	2690.2646	-3.27	1	97	2.1e-010	1	U	K.QNCDQFEKLGEYGFQNALIVR.Y + Glycation (
4806	389 - 409	897.7612	2690.2617	2690.2646	-1.09	1	108	1.5e-011	1	U	K.QNCDQFEKLGEYGFQNALIVR.Y + Glycation (
4807	389 - 409	897.7613	2690.2620	2690.2646	-0.96	1	119	1.4e-012	1	U	K.QNCDQFEKLGEYGFQNALIVR.Y + Glycation (
4808	389 - 409	1346.1383	2690.2621	2690.2646	-0.95	1	88	1.7e-009	1	U	K.QNCDQFEKLGEYGFQNALIVR.Y + Glycation (
4809	389 - 409	897.7618	2690.2635	2690.2646	-0.41	1	89	1.3e-009	1	U	K.QNCDQFEKLGEYGFQNALIVR.Y + Glycation (
1834	397 - 409	493.9354	1478.7843	1478.7881	-2.63	0	67	2.2e-007	1	U	K.LGEYGFQNALIVR.Y
1835	397 - 409	740.4004	1478.7862	1478.7881	-1.30	0	88	1.7e-009	1	U	K.LGEYGFQNALIVR.Y
1836	397 - 409	740.4006	1478.7866	1478.7881	-1.05	0	97	1.9e-010	1	U	K.LGEYGFQNALIVR.Y
1837	397 - 409	740.4009	1478.7872	1478.7881	-0.63	0	97	1.9e-010	1	U	K.LGEYGFQNALIVR.Y
1838	397 - 409	740.4010	1478.7874	1478.7881	-0.47	0	97	1.9e-010	1	U	K.LGEYGFQNALIVR.Y
1839	397 - 409	740.4023	1478.7901	1478.7881	1.34	0	97	2e-010	1	U	K.LGEYGFQNALIVR.Y
2366	413 - 427	820.4716	1638.9286	1638.9305	-1.17	1	117	2.1e-012	1	U	R.KVPQVSTPTLVEVSR.S
2367	413 - 427	820.4716	1638.9286	1638.9305	-1.17	1	117	2.2e-012	1	U	R.KVPQVSTPTLVEVSR.S
2368	413 - 427	547.3170	1638.9291	1638.9305	-0.87	1	65	3.4e-007	1	U	R.KVPQVSTPTLVEVSR.S
2369	413 - 427	820.4719	1638.9293	1638.9305	-0.73	1	97	2.1e-010	1	U	R.KVPQVSTPTLVEVSR.S
2370	413 - 427	820.4720	1638.9295	1638.9305	-0.57	1	117	2.1e-012	1	U	R.KVPQVSTPTLVEVSR.S
2371	413 - 427	820.4721	1638.9297	1638.9305	-0.50	1	79	1.4e-008	1	U	R.KVPQVSTPTLVEVSR.S
2372	413 - 427	820.4724	1638.9303	1638.9305	-0.13	1	117	2.1e-012	1	U	R.KVPQVSTPTLVEVSR.S
2373	413 - 427	547.3174	1638.9303	1638.9305	-0.10	1	51	7.9e-006	1	U	R.KVPQVSTPTLVEVSR.S
2374	413 - 427	547.3174	1638.9303	1638.9305	-0.10	1	62	7e-007	1	U	R.KVPQVSTPTLVEVSR.S
2375	413 - 427	547.3174	1638.9303	1638.9305	-0.10	1	71	7.9e-008	1	U	R.KVPQVSTPTLVEVSR.S
2376	413 - 427	820.4725	1638.9304	1638.9305	-0.057	1	117	2.1e-012	1	U	R.KVPQVSTPTLVEVSR.S
2377	413 - 427	547.3175	1638.9307	1638.9305	0.12	1	81	8.4e-009	1	U	R.KVPQVSTPTLVEVSR.S
2378	413 - 427	547.3176	1638.9309	1638.9305	0.24	1	86	2.5e-009	1	U	R.KVPQVSTPTLVEVSR.S
2379	413 - 427	820.4727	1638.9309	1638.9305	0.25	1	117	2.2e-012	1	U	R.KVPQVSTPTLVEVSR.S
2380	413 - 427	547.3176	1638.9311	1638.9305	0.35	1	69	1.3e-007	1	U	R.KVPQVSTPTLVEVSR.S
2381	413 - 427	547.3176	1638.9311	1638.9305	0.35	1	87	1.8e-009	1	U	R.KVPQVSTPTLVEVSR.S
2382	413 - 427	547.3184	1638.9334	1638.9305	1.80	1	84	4.3e-009	1	U	R.KVPQVSTPTLVEVSR.S
2383	413 - 427	547.3199	1638.9380	1638.9305	4.60	1	69	1.1e-007	1	U	R.KVPQVSTPTLVEVSR.S
3051	413 - 427	601.3336	1800.9789	1800.9833	-2.47	1	62	6.9e-007	1	U	R.KVPQVSTPTLVEVSR.S + Glycation (K)
3052	413 - 427	901.4977	1800.9809	1800.9833	-1.32	1	82	7e-009	1	U	R.KVPQVSTPTLVEVSR.S + Glycation (K)
3053	413 - 427	601.3345	1800.9816	1800.9833	-0.96	1	58	1.6e-006	1	U	R.KVPQVSTPTLVEVSR.S + Glycation (K)
3054	413 - 427	901.4981	1800.9817	1800.9833	-0.91	1	45	3e-005	1	U	R.KVPQVSTPTLVEVSR.S + Glycation (K)
3055	413 - 427	901.4984	1800.9823	1800.9833	-0.58	1	82	6.6e-009	1	U	R.KVPQVSTPTLVEVSR.S + Glycation (K)
3056	413 - 427	601.3350	1800.9831	1800.9833	-0.14	1	68	1.5e-007	1	U	R.KVPQVSTPTLVEVSR.S + Glycation (K)

Query	Start - End	Observed	Mr(expt)	Mr(calc)	ppm	M	Score	Expect	Rank	U	Peptide
3057	413 - 427	901.4994	1800.9842	1800.9833	0.51	1	94	3.9e-010	1	U	R.KVPQVSTPTLVEVSR.S + Glycation (K)
1951	414 - 427	756.4232	1510.8319	1510.8355	-2.41	0	34	0.00038	1	U	K.VPQVSTPTLVEVSR.S
1952	414 - 427	756.4237	1510.8329	1510.8355	-1.76	0	90	9e-010	1	U	K.VPQVSTPTLVEVSR.S
1953	414 - 427	756.4241	1510.8337	1510.8355	-1.20	0	119	1.3e-012	1	U	K.VPQVSTPTLVEVSR.S
1954	414 - 427	756.4241	1510.8337	1510.8355	-1.20	0	119	1.4e-012	1	U	K.VPQVSTPTLVEVSR.S
1955	414 - 427	756.4243	1510.8341	1510.8355	-0.95	0	104	3.7e-011	1	U	K.VPQVSTPTLVEVSR.S
1956	414 - 427	756.4244	1510.8343	1510.8355	-0.79	0	104	3.6e-011	1	U	K.VPQVSTPTLVEVSR.S
1957	414 - 427	504.6189	1510.8348	1510.8355	-0.49	0	45	3.1e-005	1	U	K.VPQVSTPTLVEVSR.S
1958	414 - 427	756.4247	1510.8348	1510.8355	-0.48	0	104	3.7e-011	1	U	K.VPQVSTPTLVEVSR.S
1959	414 - 427	504.6191	1510.8355	1510.8355	-0.016	0	54	4.4e-006	1	U	K.VPQVSTPTLVEVSR.S
1960	414 - 427	504.6191	1510.8355	1510.8355	-0.016	0	60	1e-006	1	U	K.VPQVSTPTLVEVSR.S
1961	414 - 427	756.4251	1510.8355	1510.8355	0.015	0	84	3.6e-009	1	U	K.VPQVSTPTLVEVSR.S
2664	445 - 458	862.9197	1723.8248	1723.8273	-1.45	0	98	1.4e-010	1	U	R.MPCTEDYLSLILNR.L
2665	445 - 458	862.9200	1723.8255	1723.8273	-1.03	0	98	1.5e-010	1	U	R.MPCTEDYLSLILNR.L
2666	445 - 458	862.9202	1723.8258	1723.8273	-0.88	0	98	1.4e-010	1	U	R.MPCTEDYLSLILNR.L
2667	445 - 458	862.9202	1723.8259	1723.8273	-0.81	0	64	3.8e-007	1	U	R.MPCTEDYLSLILNR.L
2668	445 - 458	862.9207	1723.8267	1723.8273	-0.32	0	98	1.5e-010	1	U	R.MPCTEDYLSLILNR.L
2669	445 - 458	575.6163	1723.8272	1723.8273	-0.081	0	34	0.00044	1	U	R.MPCTEDYLSLILNR.L
2670	445 - 458	575.6163	1723.8272	1723.8273	-0.081	0	36	0.00028	1	U	R.MPCTEDYLSLILNR.L
2671	445 - 458	575.6165	1723.8277	1723.8273	0.25	0	36	0.00027	1	U	R.MPCTEDYLSLILNR.L
2672	445 - 458	862.9231	1723.8316	1723.8273	2.52	0	83	5.4e-009	1	U	R.MPCTEDYLSLILNR.L
2673	445 - 458	862.9251	1723.8355	1723.8273	4.78	0	98	1.4e-010	1	U	R.MPCTEDYLSLILNR.L
358	459 - 465	449.7439	897.4732	897.4742	-1.14	0	24	0.0039	1	U	R.LCVLHEK.T
360	459 - 465	449.7440	897.4735	897.4742	-0.81	0	24	0.0038	1	U	R.LCVLHEK.T
361	459 - 465	449.7441	897.4735	897.4742	-0.74	0	27	0.0019	1	U	R.LCVLHEK.T
363	459 - 465	300.1652	897.4737	897.4742	-0.52	0	14	0.045	1	U	R.LCVLHEK.T
365	459 - 465	300.1652	897.4737	897.4742	-0.52	0	16	0.028	1	U	R.LCVLHEK.T
366	459 - 465	449.7442	897.4738	897.4742	-0.47	0	24	0.0039	1	U	R.LCVLHEK.T
367	459 - 465	300.1653	897.4739	897.4742	-0.32	0	15	0.029	1	U	R.LCVLHEK.T
368	459 - 465	300.1653	897.4741	897.4742	-0.12	0	14	0.039	1	U	R.LCVLHEK.T
369	459 - 465	300.1653	897.4741	897.4742	-0.12	0	15	0.032	1	U	R.LCVLHEK.T
370	459 - 465	449.7444	897.4741	897.4742	-0.072	0	24	0.0039	1	U	R.LCVLHEK.T
372	459 - 465	449.7445	897.4744	897.4742	0.22	0	24	0.004	1	U	R.LCVLHEK.T
374	459 - 465	449.7448	897.4749	897.4742	0.82	0	28	0.0017	1	U	R.LCVLHEK.T
2603	459 - 471	426.2236	1700.8652	1700.8655	-0.18	1	28	0.0015	1	U	R.LCVLHEKTPVSEK.V + Glycation (K)
2604	459 - 471	567.9623	1700.8652	1700.8655	-0.17	1	38	0.00015	1	U	R.LCVLHEKTPVSEK.V + Glycation (K)
2605	459 - 471	426.2237	1700.8655	1700.8655	0.034	1	18	0.015	1	U	R.LCVLHEKTPVSEK.V + Glycation (K)
2606	459 - 471	426.2237	1700.8655	1700.8655	0.034	1	22	0.0067	1	U	R.LCVLHEKTPVSEK.V + Glycation (K)
2607	459 - 471	567.9625	1700.8657	1700.8655	0.15	1	32	0.00062	1	U	R.LCVLHEKTPVSEK.V + Glycation (K)
2608	459 - 471	567.9626	1700.8659	1700.8655	0.27	1	49	1.2e-005	1	U	R.LCVLHEKTPVSEK.V + Glycation (K)
2609	459 - 471	851.4410	1700.8674	1700.8655	1.13	1	56	2.5e-006	1	U	R.LCVLHEKTPVSEK.V + Glycation (K)
2610	459 - 471	567.9631	1700.8676	1700.8655	1.22	1	23	0.0054	1	U	R.LCVLHEKTPVSEK.V + Glycation (K)
4055	459 - 474	548.7908	2191.1342	2191.1294	2.21	2	17	0.02	1	U	R.LCVLHEKTPVSEKVTK.C + 2 Glycation (K)
1014	466 - 474	384.2114	1149.6125	1149.6129	-0.37	1	18	0.016	1	U	K.TPVSEKVTK.C + Glycation (K)
1015	466 - 474	384.2115	1149.6126	1149.6129	-0.29	1	18	0.017	1	U	K.TPVSEKVTK.C + Glycation (K)
1016	466 - 474	384.2115	1149.6126	1149.6129	-0.29	1	28	0.0015	1	U	K.TPVSEKVTK.C + Glycation (K)
1017	466 - 474	575.8136	1149.6126	1149.6129	-0.21	1	39	0.00011	1	U	K.TPVSEKVTK.C + Glycation (K)
4460	466 - 483	608.7928	2431.1421	2431.1458	-1.55	2	58	1.6e-006	1	U	K.TPVSEKVTKCCTESLVNR.R + 2 Glycation (K
4461	466 - 483	811.3885	2431.1436	2431.1458	-0.90	2	72	7e-008	1	U	K.TPVSEKVTKCCTESLVNR.R + 2 Glycation (K
4462	466 - 483	811.3885	2431.1436	2431.1458	-0.90	2	71	7.1e-008	1	U	K.TPVSEKVTKCCTESLVNR.R + 2 Glycation (K
4463	466 - 483	608.7933	2431.1440	2431.1458	-0.76	2	66	2.4e-007	1	U	K.TPVSEKVTKCCTESLVNR.R + 2 Glycation (K
4464	466 - 483	811.3888	2431.1445	2431.1458	-0.53	2	71	7.3e-008	1	U	K.TPVSEKVTKCCTESLVNR.R + 2 Glycation (K
4465	466 - 483	608.7935	2431.1447	2431.1458	-0.45	2	69	1.2e-007	1	U	K.TPVSEKVTKCCTESLVNR.R + 2 Glycation (K
4466	466 - 483	608.7936	2431.1455	2431.1458	-0.15	2	81	7.5e-009	1	U	K.TPVSEKVTKCCTESLVNR.R + 2 Glycation (K
2322	472 - 483	814.8835	1627.7524	1627.7546	-1.32	1	47	2.1e-005	1	U	K.VTKCCTESLVNR.R + Glycation (K)
2323	472 - 483	814.8836	1627.7527	1627.7546	-1.16	1	54	4.3e-006	1	U	K.VTKCCTESLVNR.R + Glycation (K)
2324	472 - 483	814.8837	1627.7529	1627.7546	-1.02	1	54	4.3e-006	1	U	K.VTKCCTESLVNR.R + Glycation (K)
2325	472 - 483	814.8838	1627.7530	1627.7546	-0.94	1	54	4.2e-006	1	U	K.VTKCCTESLVNR.R + Glycation (K)
2326	472 - 483	814.8839	1627.7531	1627.7546	-0.87	1	69	1.2e-007	1	U	K.VTKCCTESLVNR.R + Glycation (K)
2327	472 - 483	543.5919	1627.7539	1627.7546	-0.39	1	76	2.5e-008	1	U	K.VTKCCTESLVNR.R + Glycation (K)
2328	472 - 483	543.5921	1627.7545	1627.7546	-0.055	1	71	8.3e-008	1	U	K.VTKCCTESLVNR.R + Glycation (K)
2329	472 - 483	543.5921	1627.7545	1627.7546	-0.055	1	62	7e-007	1	U	K.VTKCCTESLVNR.R + Glycation (K)
2330	472 - 483	543.5922	1627.7547	1627.7546	0.055	1	76	2.5e-008	1	U	K.VTKCCTESLVNR.R + Glycation (K)
2331	472 - 483	543.5922	1627.7547	1627.7546	0.055	1	60	9.7e-007	1	U	K.VTKCCTESLVNR.R + Glycation (K)
2332	472 - 483	543.5922	1627.7547	1627.7546	0.055	1	59	1.3e-006	1	U	K.VTKCCTESLVNR.R + Glycation (K)
2333	472 - 483	543.5922	1627.7548	1627.7546	0.17	1	64	4.3e-007	1	U	K.VTKCCTESLVNR.R + Glycation (K)
2334	472 - 483	814.8848	1627.7550	1627.7546	0.26	1	43	4.7e-005	1	U	K.VTKCCTESLVNR.R + Glycation (K)
2335	472 - 483	543.5928	1627.7565	1627.7546	1.18	1	89	1.4e-009	1	U	K.VTKCCTESLVNR.R + Glycation (K)
962	475 - 483	569.7526	1137.4906	1137.4907	-0.096	0	63	4.6e-007	1	U	K.CCTESLVNR.R
963	475 - 483	569.7527	1137.4908	1137.4907	0.13	0	63	4.8e-007	1	U	K.CCTESLVNR.R
964	475 - 483	569.7527	1137.4909	1137.4907	0.24	0	63	5.1e-007	1	U	K.CCTESLVNR.R
965	475 - 483	569.7529	1137.4912	1137.4907	0.45	0	63	5e-007	1	U	K.CCTESLVNR.R
966	475 - 483	569.7529	1137.4912	1137.4907	0.45	0	55	3.4e-006	1	U	K.CCTESLVNR.R
967	475 - 483	569.7533	1137.4920	1137.4907	1.21	0	50	9.3e-006	1	U	K.CCTESLVNR.R
3352	484 - 499	940.9623	1879.9100	1879.9138	-2.04	1	37	0.00019	1	U	R.RPCFSALTPDETYVPK.A
3353	484 - 499	940.9625	1879.9104	1879.9138	-1.84	1	41	7.5e-005	1	U	R.RPCFSALTPDETYVPK.A
3354	484 - 499	940.9626	1879.9106	1879.9138	-1.71	1	41	8.4e-005	1	U	R.RPCFSALTPDETYVPK.A
3355	484 - 499	940.9626	1879.9106	1879.9138	-1.71	1	46	2.4e-005	1	U	R.RPCFSALTPDETYVPK.A
3356	484 - 499	940.9631	1879.9117	1879.9138	-1.13	1	50	1e-005	1	U	R.RPCFSALTPDETYVPK.A
3357	484 - 499	940.9633	1879.9120	1879.9138	-0.99	1	49	1.4e-005	1	U	R.RPCFSALTPDETYVPK.A
3358	484 - 499	940.9634	1879.9123	1879.9138	-0.80	1	48	1.5e-005	1	U	R.RPCFSALTPDETYVPK.A
3359	484 - 499	627.6448	1879.9125	1879.9138	-0.70	1	27	0.0019	1	U	R.RPCFSALTPDETYVPK.A
3360	484 - 499	627.6450	1879.9131	1879.9138	-0.42	1	33	0.00052	1	U	R.RPCFSALTPDETYVPK.A
3361	484 - 499	627.6450	1879.9132	1879.9138	-0.32	1	28	0.0016	1	U	R.RPCFSALTPDETYVPK.A
3362	484 - 499	627.6450	1879.9132	1879.9138	-0.32	1	31	0.00081	1	U	R.RPCFSALTPDETYVPK.A
3363	484 - 499	627.6450	1879.9132	1879.9138	-0.32	1	26	0.0027	1	U	R.RPCFSALTPDETYVPK.A
3364	484 - 499	627.6451	1879.9134	1879.9138	-0.22	1	28	0.0017	1	U	R.RPCFSALTPDETYVPK.A
3365	484 - 499	627.6451	1879.9134	1879.9138	-0.22	1	35	0.0003	1	U	R.RPCFSALTPDETYVPK.A
3366	484 - 499	627.6451	1879.9136	1879.9138	-0.13	1	31	0.00083	1	U	R.RPCFSALTPDETYVPK.A

Query	Start - End	Observed	Mr(expt)	Mr(calc)	ppm	M	Score	Expect	Rank	U	Peptide
3367	484 - 499	627.6452	1879.9138	1879.9138	-0.033	1	29	0.0012	1	U	R.RPCFSALTPDETYVPK.A
3368	484 - 499	627.6452	1879.9138	1879.9138	-0.033	1	44	4.2e-005	1	U	R.RPCFSALTPDETYVPK.A
3369	484 - 499	627.6456	1879.9151	1879.9138	0.65	1	38	0.00015	1	U	R.RPCFSALTPDETYVPK.A
4717	484 - 504	878.4127	2632.2163	2632.2367	-7.73	2	25	0.0032	1	U	R.RPCFSALTPDETYVPKAFDEK.L + Glycation (
4719	484 - 504	878.4181	2632.2324	2632.2367	-1.61	2	46	2.7e-005	1	U	R.RPCFSALTPDETYVPKAFDEK.L + Glycation (
4720	484 - 504	878.4181	2632.2326	2632.2367	-1.54	2	46	2.7e-005	1	U	R.RPCFSALTPDETYVPKAFDEK.L + Glycation (
4721	484 - 504	1317.1238	2632.2330	2632.2367	-1.39	2	50	1e-005	1	U	R.RPCFSALTPDETYVPKAFDEK.L + Glycation (
4722	484 - 504	1317.1243	2632.2340	2632.2367	-1.02	2	41	7.3e-005	1	U	R.RPCFSALTPDETYVPKAFDEK.L + Glycation (
4723	484 - 504	878.4187	2632.2343	2632.2367	-0.91	2	43	5.3e-005	1	U	R.RPCFSALTPDETYVPKAFDEK.L + Glycation (
4724	484 - 504	659.0659	2632.2343	2632.2367	-0.89	2	32	0.00063	1	U	R.RPCFSALTPDETYVPKAFDEK.L + Glycation (
4725	484 - 504	878.4188	2632.2345	2632.2367	-0.85	2	41	7.9e-005	1	U	R.RPCFSALTPDETYVPKAFDEK.L + Glycation (
4726	484 - 504	659.0660	2632.2348	2632.2367	-0.71	2	43	4.5e-005	1	U	R.RPCFSALTPDETYVPKAFDEK.L + Glycation (
4727	484 - 504	878.4190	2632.2352	2632.2367	-0.56	2	45	2.9e-005	1	U	R.RPCFSALTPDETYVPKAFDEK.L + Glycation (
4728	484 - 504	659.0664	2632.2365	2632.2367	-0.054	2	36	0.00026	1	U	R.RPCFSALTPDETYVPKAFDEK.L + Glycation (
4729	484 - 504	659.0665	2632.2368	2632.2367	0.037	2	41	7.8e-005	1	U	R.RPCFSALTPDETYVPKAFDEK.L + Glycation (
6145	484 - 520	1171.8014	4683.1765	4683.1925	-3.42	3	52	7e-006	1	U	R.RPCFSALTPDETYVPKAFDEKLFTFHADICTLPDT
6146	484 - 520	937.6434	4683.1805	4683.1925	-2.56	3	42	6.4e-005	1	U	R.RPCFSALTPDETYVPKAFDEKLFTFHADICTLPDT
6147	484 - 520	937.6483	4683.2052	4683.1925	2.72	3	51	8.8e-006	1	U	R.RPCFSALTPDETYVPKAFDEKLFTFHADICTLPDT
6148	484 - 520	1171.8095	4683.2087	4683.1925	3.47	3	40	9.8e-005	1	U	R.RPCFSALTPDETYVPKAFDEKLFTFHADICTLPDT
2662	485 - 499	862.9129	1723.8112	1723.8127	-0.86	0	30	0.00094	1	U	R.PCFSALTPDETYVPK.A
2663	485 - 499	862.9131	1723.8116	1723.8127	-0.64	0	29	0.0014	1	U	R.PCFSALTPDETYVPK.A
4750	500 - 520	887.4138	2659.2195	2659.2364	-6.36	1	49	1.1e-005	1	U	K.AFDEKLFTFHADICTLPDTEK.Q + Glycation (
4751	500 - 520	887.4179	2659.2319	2659.2364	-1.68	1	67	2.2e-007	1	U	K.AFDEKLFTFHADICTLPDTEK.Q + Glycation (
4752	500 - 520	887.4185	2659.2337	2659.2364	-0.99	1	82	6.5e-009	1	U	K.AFDEKLFTFHADICTLPDTEK.Q + Glycation (
4753	500 - 520	665.8160	2659.2348	2659.2364	-0.58	1	46	2.8e-005	1	U	K.AFDEKLFTFHADICTLPDTEK.Q + Glycation (
4754	500 - 520	665.8162	2659.2355	2659.2364	-0.31	1	53	4.5e-006	1	U	K.AFDEKLFTFHADICTLPDTEK.Q + Glycation (
4756	500 - 520	665.8174	2659.2404	2659.2364	1.53	1	42	5.8e-005	1	U	K.AFDEKLFTFHADICTLPDTEK.Q + Glycation (
4757	500 - 520	887.4209	2659.2409	2659.2364	1.70	1	52	6.4e-006	1	U	K.AFDEKLFTFHADICTLPDTEK.Q + Glycation (
5382	500 - 523	1064.5165	3190.5276	3190.5268	0.26	2	25	0.0029	1	U	K.AFDEKLFTFHADICTLPDTEKQIK.K + 2 Glyca
5383	500 - 523	798.6395	3190.5290	3190.5268	0.70	2	26	0.0024	1	U	K.AFDEKLFTFHADICTLPDTEKQIK.K + 2 Glyca
5385	500 - 523	639.1133	3190.5300	3190.5268	1.02	2	14	0.039	1	U	K.AFDEKLFTFHADICTLPDTEKQIK.K + 2 Glyca
3475	505 - 520	954.4625	1906.9104	1906.9135	-1.65	0	74	3.7e-008	1	U	K.LFTFHADICTLPDTEK.Q
3476	505 - 520	954.4632	1906.9118	1906.9135	-0.87	0	78	1.5e-008	1	U	K.LFTFHADICTLPDTEK.Q
3477	505 - 520	636.6446	1906.9119	1906.9135	-0.82	0	73	4.7e-008	1	U	K.LFTFHADICTLPDTEK.Q
3478	505 - 520	954.4633	1906.9121	1906.9135	-0.75	0	96	2.7e-010	1	U	K.LFTFHADICTLPDTEK.Q
3479	505 - 520	954.4634	1906.9122	1906.9135	-0.68	0	59	1.4e-006	1	U	K.LFTFHADICTLPDTEK.Q
3480	505 - 520	636.6448	1906.9125	1906.9135	-0.52	0	66	2.7e-007	1	U	K.LFTFHADICTLPDTEK.Q
3481	505 - 520	636.6448	1906.9127	1906.9135	-0.43	0	73	4.8e-008	1	U	K.LFTFHADICTLPDTEK.Q
3482	505 - 520	636.6449	1906.9129	1906.9135	-0.34	0	51	8.8e-006	1	U	K.LFTFHADICTLPDTEK.Q
3483	505 - 520	636.6451	1906.9136	1906.9135	0.041	0	66	2.8e-007	1	U	K.LFTFHADICTLPDTEK.Q
3484	505 - 520	636.6455	1906.9147	1906.9135	0.62	0	65	3e-007	1	U	K.LFTFHADICTLPDTEK.Q
3485	505 - 520	954.4669	1906.9192	1906.9135	2.97	0	59	1.2e-006	1	U	K.LFTFHADICTLPDTEK.Q
4500	505 - 523	813.7411	2438.2014	2438.2039	-1.02	1	28	0.0014	1	U	K.LFTFHADICTLPDTEKQIK.K + Glycation (K)
4501	505 - 523	813.7416	2438.2031	2438.2039	-0.35	1	37	0.00019	1	U	K.LFTFHADICTLPDTEKQIK.K + Glycation (K)
4504	505 - 523	1220.1101	2438.2057	2438.2039	0.71	1	26	0.0024	1	U	K.LFTFHADICTLPDTEKQIK.K + Glycation (K)
5748	505 - 533	972.5084	3886.0046	3886.0061	-0.38	3	45	3.2e-005	1	U	K.LFTFHADICTLPDTEKQIKKQTALVELLK.H + 3
5749	505 - 533	972.5086	3886.0053	3886.0061	-0.19	3	53	5.4e-006	1	U	K.LFTFHADICTLPDTEKQIKKQTALVELLK.H + 3
5750	505 - 533	778.2086	3886.0064	3886.0061	0.093	3	17	0.02	1	U	K.LFTFHADICTLPDTEKQIKKQTALVELLK.H + 3
3183	521 - 533	918.5310	1835.0475	1835.0503	-1.53	2	23	0.0049	1	U	K.QIKKQTALVELLK.H + 2 Glycation (K)
3184	521 - 533	918.5316	1835.0487	1835.0503	-0.87	2	34	0.00036	1	U	K.QIKKQTALVELLK.H + 2 Glycation (K)
3185	521 - 533	612.6904	1835.0495	1835.0503	-0.44	2	24	0.0043	1	U	K.QIKKQTALVELLK.H + 2 Glycation (K)
3186	521 - 533	612.6904	1835.0495	1835.0503	-0.44	2	32	0.0006	1	U	K.QIKKQTALVELLK.H + 2 Glycation (K)
3187	521 - 533	612.6904	1835.0495	1835.0503	-0.44	2	42	6.4e-005	1	U	K.QIKKQTALVELLK.H + 2 Glycation (K)
3188	521 - 533	918.5320	1835.0495	1835.0503	-0.41	2	29	0.0013	1	U	K.QIKKQTALVELLK.H + 2 Glycation (K)
3189	521 - 533	612.6906	1835.0500	1835.0503	-0.15	2	24	0.0043	1	U	K.QIKKQTALVELLK.H + 2 Glycation (K)
3190	521 - 533	459.7698	1835.0501	1835.0503	-0.077	2	37	0.00022	1	U	K.QIKKQTALVELLK.H + 2 Glycation (K)
3191	521 - 533	459.7698	1835.0503	1835.0503	-0.012	2	26	0.0026	1	U	K.QIKKQTALVELLK.H + 2 Glycation (K)
3192	521 - 533	459.7699	1835.0504	1835.0503	0.053	2	28	0.0017	1	U	K.QIKKQTALVELLK.H + 2 Glycation (K)
3193	521 - 533	459.7699	1835.0505	1835.0503	0.12	2	24	0.0037	1	U	K.QIKKQTALVELLK.H + 2 Glycation (K)
977	524 - 533	571.8602	1141.7059	1141.7070	-0.98	1	66	2.6e-007	1	U	K.KQTALVELLK.H
978	524 - 533	381.5760	1141.7061	1141.7070	-0.78	1	38	0.00018	1	U	K.KQTALVELLK.H
979	524 - 533	571.8606	1141.7066	1141.7070	-0.34	1	61	7.4e-007	1	U	K.KQTALVELLK.H
980	524 - 533	571.8606	1141.7066	1141.7070	-0.34	1	62	6.8e-007	1	U	K.KQTALVELLK.H
981	524 - 533	571.8607	1141.7068	1141.7070	-0.23	1	62	6.9e-007	1	U	K.KQTALVELLK.H
982	524 - 533	381.5762	1141.7069	1141.7070	-0.15	1	37	0.0002	1	U	K.KQTALVELLK.H
983	524 - 533	381.5763	1141.7071	1141.7070	0.088	1	39	0.00013	1	U	K.KQTALVELLK.H
1290	524 - 533	652.8867	1303.7588	1303.7599	-0.84	1	36	0.00025	1	U	K.KQTALVELLK.H + Glycation (K)
1291	524 - 533	652.8867	1303.7589	1303.7599	-0.74	1	44	4.3e-005	1	U	K.KQTALVELLK.H + Glycation (K)
1292	524 - 533	652.8867	1303.7589	1303.7599	-0.74	1	44	4.1e-005	1	U	K.KQTALVELLK.H + Glycation (K)
1293	524 - 533	652.8870	1303.7594	1303.7599	-0.38	1	45	3e-005	1	U	K.KQTALVELLK.H + Glycation (K)
1294	524 - 533	435.5938	1303.7594	1303.7599	-0.33	1	44	3.8e-005	1	U	K.KQTALVELLK.H + Glycation (K)
1295	524 - 533	435.5938	1303.7597	1303.7599	-0.13	1	47	2.2e-005	1	U	K.KQTALVELLK.H + Glycation (K)
1296	524 - 533	652.8872	1303.7597	1303.7599	-0.084	1	46	2.3e-005	1	U	K.KQTALVELLK.H + Glycation (K)
1297	524 - 533	435.5940	1303.7601	1303.7599	0.22	1	47	2.1e-005	1	U	K.KQTALVELLK.H + Glycation (K)
1298	524 - 533	652.8878	1303.7610	1303.7599	0.85	1	44	4.3e-005	1	U	K.KQTALVELLK.H + Glycation (K)
1299	524 - 533	435.5948	1303.7626	1303.7599	2.13	1	46	2.4e-005	1	U	K.KQTALVELLK.H + Glycation (K)
619	525 - 533	507.8133	1013.6120	1013.6121	-0.047	0	54	4.3e-006	1	U	K.QTALVELLK.H
620	525 - 533	507.8134	1013.6121	1013.6121	0.071	0	46	2.7e-005	1	U	K.QTALVELLK.H
621	525 - 533	507.8134	1013.6122	1013.6121	0.15	0	53	4.7e-006	1	U	K.QTALVELLK.H
622	525 - 533	507.8134	1013.6123	1013.6121	0.27	0	33	0.00049	1	U	K.QTALVELLK.H
5221	534 - 556	754.1309	3012.4943	3012.5002	-1.93	3	79	1.2e-008	1	U	K.HKPKATEEQLKTVMENFVAFVDK.C + 2 Glycat
5222	534 - 556	1005.1730	3012.4971	3012.5002	-1.02	3	56	2.5e-006	1	U	K.HKPKATEEQLKTVMENFVAFVDK.C + 2 Glycat
5223	534 - 556	754.1321	3012.4992	3012.5002	-0.31	3	91	8.5e-010	1	U	K.HKPKATEEQLKTVMENFVAFVDK.C + 2 Glycat
5224	534 - 556	754.1321	3012.4995	3012.5002	-0.23	3	56	2.5e-006	1	U	K.HKPKATEEQLKTVMENFVAFVDK.C + 2 Glycat
5225	534 - 556	603.5074	3012.5006	3012.5002	0.14	3	22	0.0062	1	U	K.HKPKATEEQLKTVMENFVAFVDK.C + 2 Glycat
5226	534 - 556	1005.1742	3012.5007	3012.5002	0.20	3	50	9.1e-006	1	U	K.HKPKATEEQLKTVMENFVAFVDK.C + 2 Glycat
5227	534 - 556	603.5081	3012.5040	3012.5002	1.28	3	24	0.004	1	U	K.HKPKATEEQLKTVMENFVAFVDK.C + 2 Glycat

Query	Start - End	Observed	Mr(expt)	Mr(calc)	ppm	M	Score	Expect	Rank	U	Peptide
5370	534 - 556	1059.1887	3174.5443	3174.5530	-2.72	3	77	2e-008	1	U	K.HKPKATEEQLKTVMENFVAFVDK.C + 3 Glycat
5371	534 - 556	635.9174	3174.5504	3174.5530	-0.81	3	35	0.00031	1	U	K.HKPKATEEQLKTVMENFVAFVDK.C + 3 Glycat
5372	534 - 556	794.6456	3174.5532	3174.5530	0.062	3	85	3e-009	1	U	K.HKPKATEEQLKTVMENFVAFVDK.C + 3 Glycat
5373	534 - 556	1059.1924	3174.5553	3174.5530	0.73	3	69	1.3e-007	1	U	K.HKPKATEEQLKTVMENFVAFVDK.C + 3 Glycat
5374	534 - 556	794.6469	3174.5585	3174.5530	1.75	3	79	1.2e-008	1	U	K.HKPKATEEQLKTVMENFVAFVDK.C + 3 Glycat
5375	534 - 556	794.6486	3174.5651	3174.5530	3.83	3	47	2.2e-005	1	U	K.HKPKATEEQLKTVMENFVAFVDK.C + 3 Glycat
4352	538 - 556	787.7217	2360.1434	2360.1457	-0.99	1	91	8.2e-010	1	U	K.ATEEQLKTVMENFVAFVDK.C + Glycation (K)
4353	538 - 556	787.7219	2360.1439	2360.1457	-0.76	1	91	8.5e-010	1	U	K.ATEEQLKTVMENFVAFVDK.C + Glycation (K)
4354	538 - 556	787.7219	2360.1439	2360.1457	-0.76	1	111	8.9e-012	1	U	K.ATEEQLKTVMENFVAFVDK.C + Glycation (K)
4355	538 - 556	1181.0798	2360.1451	2360.1457	-0.27	1	62	6.4e-007	1	U	K.ATEEQLKTVMENFVAFVDK.C + Glycation (K)
4356	538 - 556	1181.0808	2360.1471	2360.1457	0.56	1	59	1.2e-006	1	U	K.ATEEQLKTVMENFVAFVDK.C + Glycation (K)
5446	538 - 563	836.6282	3342.4838	3342.4829	0.26	2	52	6e-006	1	U	K.ATEEQLKTVMENFVAFVDKCCAADDK.E + 2 Gly
6020	538 - 573	1108.7512	4430.9756	4430.9790	-0.78	3	109	1.4e-011	1	U	K.ATEEQLKTVMENFVAFVDKCCAADDKEACFAVEGP
6021	538 - 573	1108.7544	4430.9884	4430.9790	2.11	3	56	2.8e-006	1	U	K.ATEEQLKTVMENFVAFVDKCCAADDKEACFAVEGP
1545	545 - 556	700.3493	1398.6840	1398.6853	-0.93	0	52	5.9e-006	1	U	K.TVMENFVAFVDK.C
1546	545 - 556	700.3495	1398.6844	1398.6853	-0.66	0	71	8.5e-008	1	U	K.TVMENFVAFVDK.C
1547	545 - 556	700.3495	1398.6844	1398.6853	-0.66	0	71	8.1e-008	1	U	K.TVMENFVAFVDK.C
4394	545 - 563	794.6805	2381.0198	2381.0226	-1.16	1	55	3.3e-006	1	U	K.TVMENFVAFVDKCCAADDK.E + Glycation (K)
4395	545 - 563	794.6813	2381.0222	2381.0226	-0.15	1	53	4.9e-006	1	U	K.TVMENFVAFVDKCCAADDK.E + Glycation (K)
5553	545 - 573	868.3849	3469.5105	3469.5186	-2.36	2	48	1.4e-005	1	U	K.TVMENFVAFVDKCCAADDKEACFAVEGPK.L + Gl
5554	545 - 573	1157.5129	3469.5170	3469.5186	-0.47	2	77	2.2e-008	1	U	K.TVMENFVAFVDKCCAADDKEACFAVEGPK.L + Gl
5555	545 - 573	868.3877	3469.5217	3469.5186	0.88	2	82	7e-009	1	U	K.TVMENFVAFVDKCCAADDKEACFAVEGPK.L + Gl
3562	557 - 573	964.3994	1926.7843	1926.7910	-3.49	1	106	2.3e-011	1	U	K.CCAADDKEACFAVEGPK.L
3563	557 - 573	964.4009	1926.7872	1926.7910	-1.98	1	124	4.1e-013	1	U	K.CCAADDKEACFAVEGPK.L
3564	557 - 573	964.4017	1926.7888	1926.7910	-1.16	1	107	1.9e-011	1	U	K.CCAADDKEACFAVEGPK.L
3565	557 - 573	643.2706	1926.7899	1926.7910	-0.59	1	90	9.9e-010	1	U	K.CCAADDKEACFAVEGPK.L
3566	557 - 573	643.2709	1926.7908	1926.7910	-0.12	1	109	1.3e-011	1	U	K.CCAADDKEACFAVEGPK.L
3567	557 - 573	643.2709	1926.7908	1926.7910	-0.12	1	103	4.7e-011	1	U	K.CCAADDKEACFAVEGPK.L
3917	557 - 573	697.2879	2088.8419	2088.8438	-0.94	1	67	2e-007	1	U	K.CCAADDKEACFAVEGPK.L + Glycation (K)
3918	557 - 573	697.2881	2088.8426	2088.8438	-0.58	1	60	1.1e-006	1	U	K.CCAADDKEACFAVEGPK.L + Glycation (K)
5276	557 - 583	1025.1426	3072.4059	3072.4090	-1.00	2	77	1.9e-008	1	U	K.CCAADDKEACFAVEGPKLVVSTQTALA.- + Glyc
5277	557 - 583	1025.1428	3072.4066	3072.4090	-0.77	2	69	1.3e-007	1	U	K.CCAADDKEACFAVEGPKLVVSTQTALA.- + Glyc
5278	557 - 583	1025.1431	3072.4074	3072.4090	-0.53	2	84	3.7e-009	1	U	K.CCAADDKEACFAVEGPKLVVSTQTALA.- + Glyc
5279	557 - 583	1025.1431	3072.4074	3072.4090	-0.53	2	69	1.2e-007	1	U	K.CCAADDKEACFAVEGPKLVVSTQTALA.- + Glyc
5280	557 - 583	1025.1434	3072.4085	3072.4090	-0.17	2	42	7e-005	1	U	K.CCAADDKEACFAVEGPKLVVSTQTALA.- + Glyc
5282	557 - 583	1025.1439	3072.4099	3072.4090	0.30	2	80	1.1e-008	1	U	K.CCAADDKEACFAVEGPKLVVSTQTALA.- + Glyc
5407	557 - 583	1079.1614	3234.4623	3234.4618	0.15	2	24	0.0043	1	U	K.CCAADDKEACFAVEGPKLVVSTQTALA.- + 2 Gl
884	564 - 573	554.2601	1106.5056	1106.5066	-0.95	0	86	2.6e-009	1	U	K.EACFAVEGPK.L
885	564 - 573	554.2606	1106.5067	1106.5066	0.045	0	86	2.6e-009	1	U	K.EACFAVEGPK.L
886	564 - 573	554.2607	1106.5069	1106.5066	0.26	0	81	8.7e-009	1	U	K.EACFAVEGPK.L
887	564 - 573	554.2612	1106.5078	1106.5066	1.04	0	80	9.1e-009	1	U	K.EACFAVEGPK.L
4126	564 - 583	1127.0691	2252.1236	2252.1246	-0.44	1	35	0.00032	1	U	K.EACFAVEGPKLVVSTQTALA.- + Glycation (K
4127	564 - 583	1127.0692	2252.1239	2252.1246	-0.33	1	38	0.00017	1	U	K.EACFAVEGPKLVVSTQTALA.- + Glycation (K
4129	564 - 583	1127.0696	2252.1246	2252.1246	-0.0049	1	31	0.00083	1	U	K.EACFAVEGPKLVVSTQTALA.- + Glycation (K
4130	564 - 583	1127.0700	2252.1253	2252.1246	0.32	1	32	0.0007	1	U	K.EACFAVEGPKLVVSTQTALA.- + Glycation (K
4131	564 - 583	1127.0706	2252.1266	2252.1246	0.87	1	13	0.047	1	U	K.EACFAVEGPKLVVSTQTALA.- + Glycation (K
4133	564 - 583	1127.0731	2252.1317	2252.1246	3.14	1	29	0.0014	1	U	K.EACFAVEGPKLVVSTQTALA.- + Glycation (K
586	574 - 583	501.7950	1001.5754	1001.5757	-0.31	0	16	0.024	1	U	K.LVVSTQTALA.-
587	574 - 583	501.7950	1001.5754	1001.5757	-0.31	0	18	0.015	1	U	K.LVVSTQTALA.-
588	574 - 583	501.7951	1001.5757	1001.5757	0.014	0	21	0.0073	1	U	K.LVVSTQTALA.-
589	574 - 583	501.7951	1001.5757	1001.5757	0.014	0	18	0.016	1	U	K.LVVSTQTALA.-
592	574 - 583	501.7964	1001.5783	1001.5757	2.63	0	17	0.021	1	U	K.LVVSTQTALA.-

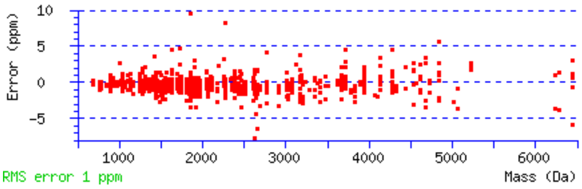

Mascot: http://www.matrixscience.com/

Appendix 6. Mascot search results of chymotrypsin digestion for BSA-3

MATRIX SCIENCE MASCOT Search Results

Protein View: >gi|00000

BSA

**Database:**	BSA
**Score:**	10362
**Monoisotopic mass (M_r):**	68386
**Calculated pI:**	5.60

Sequence similarity is available as an NCBI BLAST search of >gi|00000 against nr.

Search parameters

**MS data file:**	P2019021812.mgf
**Enzyme:**	Chymotrypsin: cuts C-term side of FLWY unless next residue is P.
**Fixed modifications:**	Carbamidomethyl (C)
**Variable modifications:**	Glycation (K), Glycation (Protein N-term)

Protein sequence coverage: 65%

Matched peptides shown in ***bold red***.

```
  1 DTHKSEIAHR FKDLGEEHFK GLVLIAFSQY LQQCPFDEHV KLVNELTEFA
 51 KTCVADESHA GCEKSLHTLF GDELCKVASL RETYGDMADC CEKQEPERNE
101 CFLSHKDDSP DLPKLKPDPN TLCDEFKADE KKFWGKYLYE IARRHPYFYA
151 PELLYYANKY NGVFQECCQA EDKGACLLPK IETMREKVLA SSARQRLRCA
201 SIQKFGERAL KAWSVARLSQ KFPKAEFVEV TKLVTDLTKV HKECCHGDLL
251 ECADDRADLA KYICDNQDTI SSKLKECCDK PLLEKSHCIA EVEKDAIPEN
301 LPPLTADFAE DKDVCKNYQE AKDAFLGSFL YEYSRRHPEY AVSVLLRLAK
351 EYEATLEECC AKDDPHACYS TVFDKLKHLV DEPQNLIKQN CDQFEKLGEY
401 GFQNALIVRY TRKVPQVSTP TLVEVSRSLG KVGTRCCTKP ESERMPCTED
451 YLSLILNRLC VLHEKTPVSE KVTKCCTESL VNRRPCFSAL TPDETYVPKA
501 FDEKLFTFHA DICTLPDTEK QIKKQTALVE LLKHKPKATE EQLKTVMENF
551 VAFVDKCCAA DDKEACFAVE GPKLVVSTQT ALA
```

Unformatted sequence string: 583 residues (for pasting into other applications).

Sort by ◉ residue number ○ increasing mass ○ decreasing mass
Show ◉ matched peptides only ○ predicted peptides also

Query	Start – End	Observed	Mr(expt)	Mr(calc)	ppm	M	Score	Expect	Rank	U	Peptide
1564	1 – 11	335.9228	1339.6623	1339.6633	-0.74	0	33	0.00055	1	U	-.DTHKSEIAHRF.K
1565	1 – 11	670.8384	1339.6623	1339.6633	-0.71	0	36	0.00028	1	U	-.DTHKSEIAHRF.K
1566	1 – 11	335.9229	1339.6624	1339.6633	-0.65	0	40	9.5e-005	1	U	-.DTHKSEIAHRF.K
1567	1 – 11	335.9229	1339.6627	1339.6633	-0.47	0	28	0.0017	1	U	-.DTHKSEIAHRF.K
1568	1 – 11	447.5615	1339.6627	1339.6633	-0.41	0	35	0.0003	1	U	-.DTHKSEIAHRF.K
1569	1 – 11	335.9230	1339.6628	1339.6633	-0.38	0	33	0.0005	1	U	-.DTHKSEIAHRF.K
1570	1 – 11	447.5616	1339.6630	1339.6633	-0.21	0	32	0.00065	1	U	-.DTHKSEIAHRF.K
1571	1 – 11	447.5616	1339.6630	1339.6633	-0.21	0	44	4.3e-005	1	U	-.DTHKSEIAHRF.K
1572	1 – 11	335.9230	1339.6630	1339.6633	-0.20	0	44	3.9e-005	1	U	-.DTHKSEIAHRF.K
1573	1 – 11	447.5616	1339.6631	1339.6633	-0.12	0	31	0.00071	1	U	-.DTHKSEIAHRF.K
1574	1 – 11	447.5619	1339.6638	1339.6633	0.42	0	32	0.00061	1	U	-.DTHKSEIAHRF.K
2006	1 – 11	376.4361	1501.7153	1501.7161	-0.54	0	43	4.6e-005	1	U	-.DTHKSEIAHRF.K + Glycation (Protein N-t
2007	1 – 11	376.4361	1501.7154	1501.7161	-0.46	0	43	4.7e-005	1	U	-.DTHKSEIAHRF.K + Glycation (Protein N-t
2008	1 – 11	501.5792	1501.7157	1501.7161	-0.30	0	43	5.4e-005	1	U	-.DTHKSEIAHRF.K + Glycation (Protein N-t
2009	1 – 11	501.5792	1501.7157	1501.7161	-0.30	0	55	3.4e-006	1	U	-.DTHKSEIAHRF.K + Glycation (Protein N-t
2010	1 – 11	376.4362	1501.7157	1501.7161	-0.30	0	40	0.00011	1	U	-.DTHKSEIAHRF.K + Glycation (Protein N-t
2011	1 – 11	501.5792	1501.7157	1501.7161	-0.25	0	40	0.0001	1	U	-.DTHKSEIAHRF.K + Glycation (Protein N-t
2012	1 – 11	376.4362	1501.7158	1501.7161	-0.22	0	43	5.1e-005	1	U	-.DTHKSEIAHRF.K + Glycation (Protein N-t
2013	1 – 11	376.4362	1501.7158	1501.7161	-0.22	0	46	2.4e-005	1	U	-.DTHKSEIAHRF.K + Glycation (Protein N-t
2014	1 – 11	501.5792	1501.7158	1501.7161	-0.19	0	40	0.0001	1	U	-.DTHKSEIAHRF.K + Glycation (Protein N-t
2015	1 – 11	376.4362	1501.7159	1501.7161	-0.14	0	32	0.00058	1	U	-.DTHKSEIAHRF.K + Glycation (K)
2016	1 – 11	501.5793	1501.7160	1501.7161	-0.065	0	49	1.3e-005	1	U	-.DTHKSEIAHRF.K + Glycation (Protein N-t
2017	1 – 11	501.5793	1501.7160	1501.7161	-0.065	0	43	4.7e-005	1	U	-.DTHKSEIAHRF.K + Glycation (Protein N-t
2018	1 – 11	376.4363	1501.7160	1501.7161	-0.063	0	40	9.4e-005	1	U	-.DTHKSEIAHRF.K + Glycation (Protein N-t
2019	1 – 11	376.4363	1501.7163	1501.7161	0.097	0	43	4.9e-005	1	U	-.DTHKSEIAHRF.K + Glycation (Protein N-t
2020	1 – 11	501.5795	1501.7168	1501.7161	0.43	0	42	5.8e-005	1	U	-.DTHKSEIAHRF.K + Glycation (Protein N-t
2341	1 – 11	416.9493	1663.7683	1663.7689	-0.41	0	33	0.00048	1	U	-.DTHKSEIAHRF.K + Glycation (K); Glycat:
2342	1 – 11	555.5967	1663.7684	1663.7689	-0.33	0	38	0.00015	1	U	-.DTHKSEIAHRF.K + Glycation (K); Glycat:
2343	1 – 11	416.9494	1663.7685	1663.7689	-0.26	0	30	0.00093	1	U	-.DTHKSEIAHRF.K + Glycation (K); Glycat:
2344	1 – 11	555.5968	1663.7686	1663.7689	-0.22	0	29	0.0013	1	U	-.DTHKSEIAHRF.K + Glycation (K); Glycat:
2345	1 – 11	416.9495	1663.7687	1663.7689	-0.12	0	40	9.7e-005	1	U	-.DTHKSEIAHRF.K + Glycation (K); Glycat:
2346	1 – 11	555.5969	1663.7689	1663.7689	-0.0024	0	39	0.00012	1	U	-.DTHKSEIAHRF.K + Glycation (K); Glycat:
2347	1 – 11	416.9495	1663.7690	1663.7689	0.024	0	31	0.00081	1	U	-.DTHKSEIAHRF.K + Glycation (K); Glycat:
2348	1 – 11	416.9497	1663.7696	1663.7689	0.41	0	29	0.0012	1	U	-.DTHKSEIAHRF.K + Glycation (K); Glycat:
764	12 – 19	487.7321	973.4497	973.4505	-0.77	1	31	0.00072	1	U	F.KDLGEEHF.K
765	12 – 19	325.4905	973.4498	973.4505	-0.73	1	19	0.013	1	U	F.KDLGEEHF.K
766	12 – 19	325.4907	973.4502	973.4505	-0.36	1	17	0.02	1	U	F.KDLGEEHF.K
767	12 – 19	325.4907	973.4502	973.4505	-0.36	1	15	0.029	1	U	F.KDLGEEHF.K
768	12 – 19	487.7324	973.4503	973.4505	-0.16	1	23	0.0047	1	U	F.KDLGEEHF.K
769	12 – 19	487.7324	973.4503	973.4505	-0.16	1	24	0.0042	1	U	F.KDLGEEHF.K
770	12 – 19	487.7325	973.4504	973.4505	-0.095	1	23	0.0053	1	U	F.KDLGEEHF.K
1107	12 – 19	568.7587	1135.5028	1135.5033	-0.47	1	24	0.0036	1	U	F.KDLGEEHF.K + Glycation (K)

Query	Start - End	Observed	Mr(expt)	Mr(calc)	ppm	M	Score	Expect	Rank	U	Peptide
1109	12 - 19	379.5082	1135.5029	1135.5033	-0.38	1	21	0.0076	1	U	F.KDLGEEHF.K + Glycation (K)
1110	12 - 19	568.7587	1135.5029	1135.5033	-0.37	1	33	0.00047	1	U	F.KDLGEEHF.K + Glycation (K)
1111	12 - 19	568.7588	1135.5030	1135.5033	-0.26	1	26	0.0024	1	U	F.KDLGEEHF.K + Glycation (K)
1112	12 - 19	568.7589	1135.5031	1135.5033	-0.16	1	26	0.0026	1	U	F.KDLGEEHF.K + Glycation (K)
1113	12 - 19	379.5083	1135.5032	1135.5033	-0.14	1	14	0.039	1	U	F.KDLGEEHF.K + Glycation (K)
1116	12 - 19	568.7589	1135.5033	1135.5033	-0.051	1	25	0.0034	1	U	F.KDLGEEHF.K + Glycation (K)
1117	12 - 19	568.7590	1135.5034	1135.5033	0.055	1	25	0.0032	1	U	F.KDLGEEHF.K + Glycation (K)
1420	12 - 22	424.8906	1271.6499	1271.6510	-0.82	2	14	0.038	1	U	F.KDLGEEHFKGL.V
1421	12 - 22	424.8908	1271.6506	1271.6510	-0.30	2	23	0.0053	1	U	F.KDLGEEHFKGL.V
1422	12 - 22	636.8326	1271.6507	1271.6510	-0.20	2	30	0.0011	1	U	F.KDLGEEHFKGL.V
1423	12 - 22	636.8326	1271.6507	1271.6510	-0.20	2	28	0.0015	1	U	F.KDLGEEHFKGL.V
1424	12 - 22	424.8909	1271.6509	1271.6510	-0.090	2	22	0.0057	1	U	F.KDLGEEHFKGL.V
1425	12 - 22	636.8328	1271.6510	1271.6510	-0.014	2	31	0.00076	1	U	F.KDLGEEHFKGL.V
1427	12 - 22	424.8910	1271.6511	1271.6510	0.12	2	20	0.0091	1	U	F.KDLGEEHFKGL.V
1863	12 - 22	717.8587	1433.7028	1433.7038	-0.67	2	26	0.0025	1	U	F.KDLGEEHFKGL.V + Glycation (K)
1864	12 - 22	717.8590	1433.7033	1433.7038	-0.32	2	16	0.023	1	U	F.KDLGEEHFKGL.V + Glycation (K)
1865	12 - 22	478.9085	1433.7035	1433.7038	-0.20	2	30	0.00094	1	U	F.KDLGEEHFKGL.V + Glycation (K)
1866	12 - 22	717.8591	1433.7036	1433.7038	-0.16	2	26	0.0024	1	U	F.KDLGEEHFKGL.V + Glycation (K)
1868	12 - 22	478.9085	1433.7037	1433.7038	-0.077	2	35	0.00032	1	U	F.KDLGEEHFKGL.V + Glycation (K)
1869	12 - 22	717.8591	1433.7037	1433.7038	-0.073	2	27	0.0019	1	U	F.KDLGEEHFKGL.V + Glycation (K)
1870	12 - 22	478.9086	1433.7039	1433.7038	0.049	2	23	0.0053	1	U	F.KDLGEEHFKGL.V + Glycation (K)
1871	12 - 22	478.9086	1433.7039	1433.7038	0.049	2	36	0.00026	1	U	F.KDLGEEHFKGL.V + Glycation (K)
1872	12 - 22	478.9086	1433.7040	1433.7038	0.11	2	33	0.00047	1	U	F.KDLGEEHFKGL.V + Glycation (K)
1873	12 - 22	478.9088	1433.7044	1433.7038	0.43	2	33	0.00052	1	U	F.KDLGEEHFKGL.V + Glycation (K)
2230	12 - 22	798.8845	1595.7545	1595.7566	-1.35	2	14	0.036	1	U	F.KDLGEEHFKGL.V + 2 Glycation (K)
2232	12 - 22	532.9262	1595.7566	1595.7566	-0.010	2	30	0.00093	1	U	F.KDLGEEHFKGL.V + 2 Glycation (K)
2236	12 - 22	532.9263	1595.7572	1595.7566	0.33	2	23	0.0054	1	U	F.KDLGEEHFKGL.V + 2 Glycation (K)
2237	12 - 22	532.9263	1595.7572	1595.7566	0.33	2	31	0.00086	1	U	F.KDLGEEHFKGL.V + 2 Glycation (K)
2239	12 - 22	532.9265	1595.7577	1595.7566	0.67	2	28	0.0016	1	U	F.KDLGEEHFKGL.V + 2 Glycation (K)
23	20 - 24	265.1890	528.3634	528.3635	-0.15	1	15	0.034	1	U	F.KGLVL.I
25	20 - 24	265.1890	528.3634	528.3635	-0.15	1	13	0.049	1	U	F.KGLVL.I
346	31 - 36	396.6890	791.3635	791.3636	-0.14	1	23	0.005	1	U	Y.LQQCPF.D
347	31 - 36	396.6890	791.3635	791.3636	-0.14	1	30	0.00095	1	U	Y.LQQCPF.D
348	31 - 36	396.6894	791.3642	791.3636	0.70	1	23	0.0051	1	U	Y.LQQCPF.D
2047	31 - 42	505.2525	1512.7358	1512.7395	-2.47	2	26	0.0025	1	U	Y.LQQCPFDEHVKL.V
2048	31 - 42	757.3757	1512.7368	1512.7395	-1.79	2	22	0.0058	1	U	Y.LQQCPFDEHVKL.V
2050	31 - 42	505.2533	1512.7381	1512.7395	-0.95	2	24	0.0037	1	U	Y.LQQCPFDEHVKL.V
2051	31 - 42	757.3766	1512.7386	1512.7395	-0.58	2	25	0.003	1	U	Y.LQQCPFDEHVKL.V
2052	31 - 42	505.2538	1512.7396	1512.7395	0.066	2	20	0.01	1	U	Y.LQQCPFDEHVKL.V
2368	31 - 42	838.4028	1674.7910	1674.7923	-0.80	2	23	0.0054	1	U	Y.LQQCPFDEHVKL.V + Glycation (K)
2369	31 - 42	559.2714	1674.7923	1674.7923	-0.045	2	14	0.037	1	U	Y.LQQCPFDEHVKL.V + Glycation (K)
2371	31 - 42	838.4036	1674.7926	1674.7923	0.14	2	18	0.014	1	U	Y.LQQCPFDEHVKL.V + Glycation (K)
3449	31 - 49	1173.5765	2345.1384	2345.1362	0.93	4	35	0.0003	1	U	Y.LQQCPFDEHVKLVNELTEF.A
3450	31 - 49	1173.5781	2345.1417	2345.1362	2.36	4	23	0.0048	1	U	Y.LQQCPFDEHVKLVNELTEF.A
3721	31 - 49	1254.6023	2507.1900	2507.1890	0.41	4	30	0.00096	1	U	Y.LQQCPFDEHVKLVNELTEF.A + Glycation (K)
3723	31 - 49	1254.6026	2507.1907	2507.1890	0.67	4	13	0.047	1	U	Y.LQQCPFDEHVKLVNELTEF.A + Glycation (K)
3724	31 - 49	1254.6032	2507.1918	2507.1890	1.12	4	28	0.0015	1	U	Y.LQQCPFDEHVKLVNELTEF.A + Glycation (K)
587	37 - 42	451.7268	901.4391	901.4393	-0.22	0	15	0.03	1	U	F.DEHVKL.V + Glycation (K)
590	37 - 42	451.7270	901.4394	901.4393	0.11	0	14	0.039	1	U	F.DEHVKL.V + Glycation (K)
2174	37 - 49	786.8984	1571.7822	1571.7831	-0.58	2	47	2e-005	1	U	F.DEHVKLVNELTEF.A
2177	37 - 49	524.9351	1571.7834	1571.7831	0.14	2	58	1.8e-006	1	U	F.DEHVKLVNELTEF.A
2179	37 - 49	524.9352	1571.7837	1571.7831	0.37	2	60	1.1e-006	1	U	F.DEHVKLVNELTEF.A
2181	37 - 49	786.8999	1571.7852	1571.7831	1.35	2	50	1.1e-005	1	U	F.DEHVKLVNELTEF.A
2479	37 - 49	867.9246	1733.8347	1733.8360	-0.73	2	64	4.3e-007	1	U	F.DEHVKLVNELTEF.A + Glycation (K)
2480	37 - 49	867.9249	1733.8353	1733.8360	-0.37	2	64	4.4e-007	1	U	F.DEHVKLVNELTEF.A + Glycation (K)
2481	37 - 49	578.9526	1733.8359	1733.8360	-0.024	2	62	6e-007	1	U	F.DEHVKLVNELTEF.A + Glycation (K)
2482	37 - 49	578.9527	1733.8363	1733.8360	0.18	2	74	4e-008	1	U	F.DEHVKLVNELTEF.A + Glycation (K)
2483	37 - 49	578.9528	1733.8365	1733.8360	0.29	2	68	1.6e-007	1	U	F.DEHVKLVNELTEF.A + Glycation (K)
2484	37 - 49	867.9258	1733.8371	1733.8360	0.68	2	49	1.4e-005	1	U	F.DEHVKLVNELTEF.A + Glycation (K)
477	43 - 49	426.2108	850.4069	850.4072	-0.35	1	17	0.018	1	U	L.VNELTEF.A
2908	50 - 66	675.6347	2023.8823	2023.8827	-0.19	0	24	0.0042	1	U	F.AKTCVADESHAGCEKSL.H + Glycation (K)
3139	50 - 66	729.6522	2185.9347	2185.9355	-0.38	0	30	0.0011	1	U	F.AKTCVADESHAGCEKSL.H + 2 Glycation (K)
3860	50 - 70	672.0554	2684.1923	2684.1946	-0.83	2	15	0.033	1	U	F.AKTCVADESHAGCEKSLHTLF.G + 2 Glycatio
3861	50 - 70	672.0557	2684.1935	2684.1946	-0.38	2	22	0.0058	1	U	F.AKTCVADESHAGCEKSLHTLF.G + 2 Glycatio
1028	71 - 80	546.2737	1090.5328	1090.5329	-0.040	1	44	4e-005	1	U	F.GDELCKVASL.R
1029	71 - 80	546.2737	1090.5328	1090.5329	-0.040	1	45	3.3e-005	1	U	F.GDELCKVASL.R
1377	71 - 80	627.2999	1252.5852	1252.5857	-0.39	1	26	0.0027	1	U	F.GDELCKVASL.R + Glycation (K)
1378	71 - 80	627.3002	1252.5858	1252.5857	0.088	1	21	0.0083	1	U	F.GDELCKVASL.R + Glycation (K)
147	75 - 80	339.1862	676.3579	676.3578	0.24	0	22	0.006	1	U	L.CKVASL.R
441	75 - 80	420.2125	838.4104	838.4106	-0.22	0	15	0.029	1	U	L.CKVASL.R + Glycation (K)
442	75 - 80	420.2128	838.4110	838.4106	0.45	0	16	0.026	1	U	L.CKVASL.R + Glycation (K)
3308	85 - 102	1137.9285	2273.8424	2273.8446	-0.96	0	64	3.9e-007	1	U	Y.GDMADCCEKQEPERNECF.L
3309	85 - 102	758.9553	2273.8440	2273.8446	-0.27	0	59	1.4e-006	1	U	Y.GDMADCCEKQEPERNECF.L
3310	85 - 102	758.9553	2273.8441	2273.8446	-0.19	0	55	3.4e-006	1	U	Y.GDMADCCEKQEPERNECF.L
3311	85 - 102	758.9555	2273.8447	2273.8446	0.056	0	29	0.0014	1	U	Y.GDMADCCEKQEPERNECF.L
3312	85 - 102	758.9610	2273.8612	2273.8446	7.30	0	25	0.0033	1	U	Y.GDMADCCEKQEPERNECF.L
3613	85 - 102	812.9724	2435.8952	2435.8974	-0.89	0	39	0.00013	1	U	Y.GDMADCCEKQEPERNECF.L + Glycation (K)
3614	85 - 102	812.9727	2435.8963	2435.8974	-0.44	0	42	7e-005	1	U	Y.GDMADCCEKQEPERNECF.L + Glycation (K)
3615	85 - 102	1218.9565	2435.8985	2435.8974	0.46	0	29	0.0012	1	U	Y.GDMADCCEKQEPERNECF.L + Glycation (K)
3227	103 - 122	744.0616	2229.1629	2229.1641	-0.53	2	30	0.00096	1	U	F.LSHKDDSPDLPKLKPDPNTL.C
3228	103 - 122	558.2982	2229.1638	2229.1641	-0.14	2	26	0.0024	1	U	F.LSHKDDSPDLPKLKPDPNTL.C
3229	103 - 122	558.2988	2229.1660	2229.1641	0.84	2	17	0.019	1	U	F.LSHKDDSPDLPKLKPDPNTL.C
3557	103 - 122	798.0797	2391.2173	2391.2169	0.16	2	23	0.0056	1	U	F.LSHKDDSPDLPKLKPDPNTL.C + Glycation (K
3558	103 - 122	598.8121	2391.2192	2391.2169	0.94	2	16	0.026	1	U	F.LSHKDDSPDLPKLKPDPNTL.C + Glycation (K
3781	103 - 122	639.3251	2553.2714	2553.2697	0.65	2	20	0.01	1	U	F.LSHKDDSPDLPKLKPDPNTL.C + 2 Glycation
3894	103 - 126	927.7836	2780.3291	2780.3327	-1.31	3	42	6.2e-005	1	U	F.LSHKDDSPDLPKLKPDPNTLCDEF.K
3895	103 - 126	557.0732	2780.3298	2780.3327	-1.04	3	18	0.014	1	U	F.LSHKDDSPDLPKLKPDPNTLCDEF.K
3896	103 - 126	696.0900	2780.3308	2780.3327	-0.69	3	18	0.018	1	U	F.LSHKDDSPDLPKLKPDPNTLCDEF.K

Query	Start – End	Observed	Mr(expt)	Mr(calc)	ppm	M	Score	Expect	Rank	U	Peptide
3897	103 – 126	927.7843	2780.3311	2780.3327	-0.59	3	41	8e-005	1	U	F.LSHKDDSPDLPKLKPDPNTLCDEF.K
3898	103 – 126	927.7844	2780.3313	2780.3327	-0.52	3	22	0.0066	1	U	F.LSHKDDSPDLPKLKPDPNTLCDEF.K
3900	103 – 126	696.0901	2780.3315	2780.3327	-0.43	3	16	0.024	1	U	F.LSHKDDSPDLPKLKPDPNTLCDEF.K
3901	103 – 126	696.0903	2780.3320	2780.3327	-0.26	3	16	0.024	1	U	F.LSHKDDSPDLPKLKPDPNTLCDEF.K
3902	103 – 126	927.7847	2780.3324	2780.3327	-0.12	3	28	0.0017	1	U	F.LSHKDDSPDLPKLKPDPNTLCDEF.K
3905	103 – 126	696.0906	2780.3335	2780.3327	0.27	3	20	0.011	1	U	F.LSHKDDSPDLPKLKPDPNTLCDEF.K
3906	103 – 126	927.7853	2780.3340	2780.3327	0.47	3	23	0.0054	1	U	F.LSHKDDSPDLPKLKPDPNTLCDEF.K
3907	103 – 126	557.0741	2780.3341	2780.3327	0.51	3	14	0.036	1	U	F.LSHKDDSPDLPKLKPDPNTLCDEF.K
4020	103 – 126	981.8009	2942.3809	2942.3855	-1.58	3	23	0.0051	1	U	F.LSHKDDSPDLPKLKPDPNTLCDEF.K + Glycati
4021	103 – 126	981.8012	2942.3818	2942.3855	-1.27	3	32	0.00061	1	U	F.LSHKDDSPDLPKLKPDPNTLCDEF.K + Glycati
4022	103 – 126	981.8013	2942.3822	2942.3855	-1.14	3	30	0.00096	1	U	F.LSHKDDSPDLPKLKPDPNTLCDEF.K + Glycati
4023	103 – 126	589.4838	2942.3824	2942.3855	-1.06	3	20	0.009	1	U	F.LSHKDDSPDLPKLKPDPNTLCDEF.K + Glycati
4024	103 – 126	736.6030	2942.3828	2942.3855	-0.94	3	42	6.5e-005	1	U	F.LSHKDDSPDLPKLKPDPNTLCDEF.K + Glycati
4025	103 – 126	981.8016	2942.3829	2942.3855	-0.90	3	22	0.0061	1	U	F.LSHKDDSPDLPKLKPDPNTLCDEF.K + Glycati
4026	103 – 126	736.6031	2942.3833	2942.3855	-0.77	3	41	8.2e-005	1	U	F.LSHKDDSPDLPKLKPDPNTLCDEF.K + Glycati
4027	103 – 126	981.8017	2942.3833	2942.3855	-0.77	3	40	0.0001	1	U	F.LSHKDDSPDLPKLKPDPNTLCDEF.K + Glycati
4028	103 – 126	589.4841	2942.3840	2942.3855	-0.53	3	31	0.00076	1	U	F.LSHKDDSPDLPKLKPDPNTLCDEF.K + Glycati
4029	103 – 126	981.8020	2942.3842	2942.3855	-0.46	3	33	0.00046	1	U	F.LSHKDDSPDLPKLKPDPNTLCDEF.K + Glycati
4030	103 – 126	736.6033	2942.3842	2942.3855	-0.45	3	29	0.0013	1	U	F.LSHKDDSPDLPKLKPDPNTLCDEF.K + Glycati
4031	103 – 126	736.6034	2942.3847	2942.3855	-0.28	3	28	0.0018	1	U	F.LSHKDDSPDLPKLKPDPNTLCDEF.K + Glycati
4032	103 – 126	589.4844	2942.3855	2942.3855	-0.0031	3	20	0.01	1	U	F.LSHKDDSPDLPKLKPDPNTLCDEF.K + Glycati
4033	103 – 126	736.6038	2942.3859	2942.3855	0.14	3	37	0.0002	1	U	F.LSHKDDSPDLPKLKPDPNTLCDEF.K + Glycati
4034	103 – 126	736.6042	2942.3879	2942.3855	0.80	3	24	0.0042	1	U	F.LSHKDDSPDLPKLKPDPNTLCDEF.K + Glycati
4035	103 – 126	736.6046	2942.3893	2942.3855	1.29	3	31	0.00089	1	U	F.LSHKDDSPDLPKLKPDPNTLCDEF.K + Glycati
4182	103 – 126	1035.8177	3104.4314	3104.4384	-2.23	3	28	0.0016	1	U	F.LSHKDDSPDLPKLKPDPNTLCDEF.K + 2 Glyca
4183	103 – 126	1035.8179	3104.4318	3104.4384	-2.12	3	18	0.016	1	U	F.LSHKDDSPDLPKLKPDPNTLCDEF.K + 2 Glyca
4184	103 – 126	1035.8181	3104.4325	3104.4384	-1.88	3	29	0.0014	1	U	F.LSHKDDSPDLPKLKPDPNTLCDEF.K + 2 Glyca
4185	103 – 126	777.1157	3104.4335	3104.4384	-1.55	3	19	0.012	1	U	F.LSHKDDSPDLPKLKPDPNTLCDEF.K + 2 Glyca
4186	103 – 126	777.1160	3104.4348	3104.4384	-1.15	3	15	0.034	1	U	F.LSHKDDSPDLPKLKPDPNTLCDEF.K + 2 Glyca
4189	103 – 126	1035.8195	3104.4366	3104.4384	-0.58	3	25	0.0028	1	U	F.LSHKDDSPDLPKLKPDPNTLCDEF.K + 2 Glyca
4190	103 – 126	777.1165	3104.4367	3104.4384	-0.52	3	29	0.0011	1	U	F.LSHKDDSPDLPKLKPDPNTLCDEF.K + 2 Glyca
4191	103 – 126	621.8947	3104.4372	3104.4384	-0.38	3	24	0.004	1	U	F.LSHKDDSPDLPKLKPDPNTLCDEF.K + 2 Glyca
4192	103 – 126	777.1168	3104.4379	3104.4384	-0.13	3	20	0.011	1	U	F.LSHKDDSPDLPKLKPDPNTLCDEF.K + 2 Glyca
4193	103 – 126	621.8950	3104.4384	3104.4384	0.021	3	30	0.0009	1	U	F.LSHKDDSPDLPKLKPDPNTLCDEF.K + 2 Glyca
4194	103 – 126	1035.8209	3104.4409	3104.4384	0.83	3	21	0.0084	1	U	F.LSHKDDSPDLPKLKPDPNTLCDEF.K + 2 Glyca
1587	104 – 115	338.6767	1350.6776	1350.6779	-0.26	0	15	0.029	1	U	L.SHKDDSPDLPKL.K
1588	104 – 115	451.2332	1350.6776	1350.6779	-0.23	0	19	0.013	1	U	L.SHKDDSPDLPKL.K
1589	104 – 115	451.2332	1350.6777	1350.6779	-0.16	0	17	0.02	1	U	L.SHKDDSPDLPKL.K
1590	104 – 115	451.2333	1350.6782	1350.6779	0.19	0	20	0.01	1	U	L.SHKDDSPDLPKL.K
3848	104 – 126	890.0889	2667.2448	2667.2486	-1.45	2	34	0.00037	1	U	L.SHKDDSPDLPKLKPDPNTLCDEF.K
3849	104 – 126	890.0893	2667.2460	2667.2486	-0.97	2	52	5.8e-006	1	U	L.SHKDDSPDLPKLKPDPNTLCDEF.K
3850	104 – 126	667.8190	2667.2468	2667.2486	-0.70	2	25	0.0029	1	U	L.SHKDDSPDLPKLKPDPNTLCDEF.K
3851	104 – 126	667.8190	2667.2470	2667.2486	-0.61	2	35	0.00028	1	U	L.SHKDDSPDLPKLKPDPNTLCDEF.K
3852	104 – 126	667.8192	2667.2475	2667.2486	-0.43	2	27	0.0022	1	U	L.SHKDDSPDLPKLKPDPNTLCDEF.K
3853	104 – 126	890.0902	2667.2488	2667.2486	0.060	2	47	2.1e-005	1	U	L.SHKDDSPDLPKLKPDPNTLCDEF.K
3927	104 – 126	944.1060	2829.2961	2829.3015	-1.91	2	30	0.00091	1	U	L.SHKDDSPDLPKLKPDPNTLCDEF.K + Glycatio
3928	104 – 126	944.1063	2829.2970	2829.3015	-1.60	2	36	0.00023	1	U	L.SHKDDSPDLPKLKPDPNTLCDEF.K + Glycatio
3929	104 – 126	944.1068	2829.2984	2829.3015	-1.08	2	19	0.011	1	U	L.SHKDDSPDLPKLKPDPNTLCDEF.K + Glycatio
3930	104 – 126	944.1071	2829.2995	2829.3015	-0.68	2	33	0.00049	1	U	L.SHKDDSPDLPKLKPDPNTLCDEF.K + Glycatio
3931	104 – 126	708.3322	2829.2997	2829.3015	-0.61	2	45	3e-005	1	U	L.SHKDDSPDLPKLKPDPNTLCDEF.K + Glycatio
3932	104 – 126	708.3323	2829.3003	2829.3015	-0.43	2	30	0.00098	1	U	L.SHKDDSPDLPKLKPDPNTLCDEF.K + Glycatio
3933	104 – 126	708.3325	2829.3007	2829.3015	-0.26	2	24	0.0043	1	U	L.SHKDDSPDLPKLKPDPNTLCDEF.K + Glycatio
3934	104 – 126	708.3326	2829.3015	2829.3015	-0.0042	2	25	0.0032	1	U	L.SHKDDSPDLPKLKPDPNTLCDEF.K + Glycatio
4072	104 – 126	748.8450	2991.3510	2991.3543	-1.10	2	19	0.014	1	U	L.SHKDDSPDLPKLKPDPNTLCDEF.K + 2 Glycat
4073	104 – 126	748.8455	2991.3530	2991.3543	-0.44	2	17	0.02	1	U	L.SHKDDSPDLPKLKPDPNTLCDEF.K + 2 Glycat
4074	104 – 126	998.1249	2991.3530	2991.3543	-0.44	2	20	0.01	1	U	L.SHKDDSPDLPKLKPDPNTLCDEF.K + 2 Glycat
4075	104 – 126	998.1255	2991.3546	2991.3543	0.12	2	21	0.0089	1	U	L.SHKDDSPDLPKLKPDPNTLCDEF.K + 2 Glycat
918	127 – 134	351.1904	1050.5494	1050.5498	-0.41	1	25	0.003	1	U	F.KADEKKFW.G
919	127 – 134	526.2822	1050.5498	1050.5498	-0.016	1	37	0.00018	1	U	F.KADEKKFW.G
920	127 – 134	351.1906	1050.5499	1050.5498	0.10	1	25	0.0029	1	U	F.KADEKKFW.G
921	127 – 134	526.2823	1050.5500	1050.5498	0.21	1	39	0.00013	1	U	F.KADEKKFW.G
1271	127 – 134	405.2079	1212.6018	1212.6026	-0.72	1	26	0.0026	1	U	F.KADEKKFW.G + Glycation (K)
1272	127 – 134	607.3084	1212.6021	1212.6026	-0.40	1	26	0.0023	1	U	F.KADEKKFW.G + Glycation (K)
1273	127 – 134	405.2080	1212.6022	1212.6026	-0.35	1	26	0.0026	1	U	F.KADEKKFW.G + Glycation (K)
1274	127 – 134	607.3085	1212.6024	1212.6026	-0.20	1	19	0.013	1	U	F.KADEKKFW.G + Glycation (K)
1275	127 – 134	607.3085	1212.6025	1212.6026	-0.10	1	24	0.0037	1	U	F.KADEKKFW.G + Glycation (K)
1276	127 – 134	607.3085	1212.6025	1212.6026	-0.10	1	29	0.0012	1	U	F.KADEKKFW.G + Glycation (K)
1277	127 – 134	405.2081	1212.6026	1212.6026	-0.057	1	34	0.00039	1	U	F.KADEKKFW.G + Glycation (K)
1280	127 – 134	405.2082	1212.6027	1212.6026	0.092	1	23	0.005	1	U	F.KADEKKFW.G + Glycation (K)
1668	127 – 134	688.3345	1374.6544	1374.6555	-0.78	1	19	0.014	1	U	F.KADEKKFW.G + 2 Glycation (K)
1669	127 – 134	688.3347	1374.6548	1374.6555	-0.50	1	21	0.0079	1	U	F.KADEKKFW.G + 2 Glycation (K)
1672	127 – 134	459.2256	1374.6551	1374.6555	-0.24	1	20	0.011	1	U	F.KADEKKFW.G + 2 Glycation (K)
482	148 – 154	426.7287	851.4428	851.4429	-0.045	3	21	0.0083	1	U	Y.FYAPELL.Y
483	148 – 154	426.7287	851.4429	851.4429	0.026	3	15	0.029	1	U	Y.FYAPELL.Y
484	148 – 154	426.7288	851.4430	851.4429	0.096	3	21	0.0081	1	U	Y.FYAPELL.Y
485	148 – 154	426.7288	851.4430	851.4429	0.17	3	21	0.0081	1	U	Y.FYAPELL.Y
486	148 – 154	426.7288	851.4430	851.4429	0.17	3	21	0.0082	1	U	Y.FYAPELL.Y
835	148 – 155	508.2605	1014.5064	1014.5062	0.18	4	14	0.036	1	U	Y.FYAPELLY.Y
836	148 – 155	508.2605	1014.5064	1014.5062	0.18	4	14	0.037	1	U	Y.FYAPELLY.Y
2848	161 – 177	993.4102	1984.8059	1984.8077	-0.93	1	59	1.2e-006	1	U	Y.NGVFQECCQAEDKGACL.L
2849	161 – 177	993.4108	1984.8071	1984.8077	-0.32	1	75	3.5e-008	1	U	Y.NGVFQECCQAEDKGACL.L
2850	161 – 177	662.6099	1984.8078	1984.8077	0.0065	1	34	0.0004	1	U	Y.NGVFQECCQAEDKGACL.L
3070	161 – 177	716.6267	2146.8583	2146.8606	-1.05	1	28	0.0015	1	U	Y.NGVFQECCQAEDKGACL.L + Glycation (K)
3071	161 – 177	716.6268	2146.8587	2146.8606	-0.89	1	28	0.0017	1	U	Y.NGVFQECCQAEDKGACL.L + Glycation (K)
3072	161 – 177	1074.4375	2146.8604	2146.8606	-0.054	1	58	1.7e-006	1	U	Y.NGVFQECCQAEDKGACL.L + Glycation (K)
3073	161 – 177	1074.4383	2146.8621	2146.8606	0.74	1	47	2.2e-005	1	U	Y.NGVFQECCQAEDKGACL.L + Glycation (K)
3074	161 – 177	1074.4383	2146.8621	2146.8606	0.74	1	53	5.1e-006	1	U	Y.NGVFQECCQAEDKGACL.L + Glycation (K)

Query	Start - End	Observed	Mr(expt)	Mr(calc)	ppm	M	Score	Expect	Rank	U	Peptide
2164	165 - 177	784.8098	1567.6051	1567.6065	-0.92	0	48	1.5e-005	1	U	F.QECCQAEDKGACL.L
2470	165 - 177	865.8361	1729.6577	1729.6593	-0.95	0	34	0.00038	1	U	F.QECCQAEDKGACL.L + Glycation (K)
1003	219 - 227	541.2874	1080.5603	1080.5604	-0.10	0	22	0.0059	1	U	L.SQKFPKAEF.V
1004	219 - 227	541.2875	1080.5604	1080.5604	0.027	0	23	0.0048	1	U	L.SQKFPKAEF.V
1006	219 - 227	541.2875	1080.5605	1080.5604	0.14	0	22	0.0061	1	U	L.SQKFPKAEF.V
1007	219 - 227	541.2875	1080.5605	1080.5604	0.14	0	23	0.0051	1	U	L.SQKFPKAEF.V
1009	219 - 227	541.2876	1080.5606	1080.5604	0.25	0	23	0.0051	1	U	L.SQKFPKAEF.V
1010	219 - 227	541.2878	1080.5611	1080.5604	0.69	0	20	0.011	1	U	L.SQKFPKAEF.V
1326	219 - 227	622.3134	1242.6123	1242.6132	-0.74	0	26	0.0026	1	U	L.SQKFPKAEF.V + Glycation (K)
1327	219 - 227	622.3135	1242.6124	1242.6132	-0.64	0	15	0.03	1	U	L.SQKFPKAEF.V + Glycation (K)
1329	219 - 227	415.2115	1242.6126	1242.6132	-0.53	0	15	0.032	1	U	L.SQKFPKAEF.V + Glycation (K)
1330	219 - 227	415.2116	1242.6131	1242.6132	-0.091	0	15	0.03	1	U	L.SQKFPKAEF.V + Glycation (K)
1331	219 - 227	622.3138	1242.6131	1242.6132	-0.062	0	19	0.012	1	U	L.SQKFPKAEF.V + Glycation (K)
1332	219 - 227	415.2117	1242.6132	1242.6132	-0.019	0	15	0.031	1	U	L.SQKFPKAEF.V + Glycation (K)
1333	219 - 227	415.2117	1242.6133	1242.6132	0.054	0	21	0.0072	1	U	L.SQKFPKAEF.V + Glycation (K)
1335	219 - 227	415.2118	1242.6135	1242.6132	0.27	0	15	0.032	1	U	L.SQKFPKAEF.V + Glycation (K)
2979	219 - 233	692.3644	2074.0713	2074.0722	-0.41	1	50	1e-005	1	U	L.SQKFPKAEFVEVTKL.V + 2 Glycation (K)
2980	219 - 233	692.3644	2074.0713	2074.0722	-0.41	1	41	8e-005	1	U	L.SQKFPKAEFVEVTKL.V + 2 Glycation (K)
170	228 - 233	344.7154	687.4163	687.4167	-0.58	0	19	0.014	1	U	F.VEVTKL.V
171	228 - 233	344.7154	687.4163	687.4167	-0.58	0	19	0.014	1	U	F.VEVTKL.V
172	228 - 233	344.7155	687.4164	687.4167	-0.41	0	23	0.0045	1	U	F.VEVTKL.V
173	228 - 233	344.7155	687.4164	687.4167	-0.41	0	19	0.012	1	U	F.VEVTKL.V
174	228 - 233	344.7156	687.4165	687.4167	-0.20	0	21	0.0088	1	U	F.VEVTKL.V
466	228 - 233	425.7417	849.4688	849.4695	-0.85	0	23	0.005	1	U	F.VEVTKL.V + Glycation (K)
468	228 - 233	425.7419	849.4692	849.4695	-0.36	0	26	0.0026	1	U	F.VEVTKL.V + Glycation (K)
469	228 - 233	425.7419	849.4692	849.4695	-0.36	0	23	0.0045	1	U	F.VEVTKL.V + Glycation (K)
471	228 - 233	425.7420	849.4694	849.4695	-0.15	0	26	0.0027	1	U	F.VEVTKL.V + Glycation (K)
473	228 - 233	425.7420	849.4694	849.4695	-0.077	0	26	0.0024	1	U	F.VEVTKL.V + Glycation (K)
474	228 - 233	425.7420	849.4694	849.4695	-0.077	0	26	0.0023	1	U	F.VEVTKL.V + Glycation (K)
475	228 - 233	425.7421	849.4696	849.4695	0.16	0	25	0.003	1	U	F.VEVTKL.V + Glycation (K)
1438	228 - 237	639.8555	1277.6964	1277.6966	-0.19	1	26	0.0026	1	U	F.VEVTKLVTDL.T + Glycation (K)
4148	234 - 259	768.3539	3069.3867	3069.3954	-2.85	3	85	3e-009	1	U	L.VTDLTKVHKECCHGDLLECADDRADL.A
4149	234 - 259	614.8850	3069.3887	3069.3954	-2.20	3	22	0.0058	1	U	L.VTDLTKVHKECCHGDLLECADDRADL.A
4150	234 - 259	1024.1392	3069.3957	3069.3954	0.077	3	61	8.1e-007	1	U	L.VTDLTKVHKECCHGDLLECADDRADL.A
4287	234 - 259	808.8684	3231.4445	3231.4482	-1.15	3	64	4.2e-007	1	U	L.VTDLTKVHKECCHGDLLECADDRADL.A + Glyca
4288	234 - 259	808.8684	3231.4445	3231.4482	-1.15	3	79	1.3e-008	1	U	L.VTDLTKVHKECCHGDLLECADDRADL.A + Glyca
4289	234 - 259	808.8688	3231.4460	3231.4482	-0.70	3	85	3.1e-009	1	U	L.VTDLTKVHKECCHGDLLECADDRADL.A + Glyca
4290	234 - 259	647.2969	3231.4480	3231.4482	-0.069	3	30	0.00095	1	U	L.VTDLTKVHKECCHGDLLECADDRADL.A + Glyca
4291	234 - 259	647.2972	3231.4498	3231.4482	0.49	3	35	0.00031	1	U	L.VTDLTKVHKECCHGDLLECADDRADL.A + Glyca
4292	234 - 259	647.2976	3231.4517	3231.4482	1.06	3	35	0.00032	1	U	L.VTDLTKVHKECCHGDLLECADDRADL.A + Glyca
4293	234 - 259	1078.1592	3231.4557	3231.4482	2.31	3	112	5.9e-012	1	U	L.VTDLTKVHKECCHGDLLECADDRADL.A + Glyca
4367	234 - 259	679.7069	3393.4982	3393.5011	-0.85	3	38	0.00016	1	U	L.VTDLTKVHKECCHGDLLECADDRADL.A + 2 Gly
4368	234 - 259	849.3821	3393.4992	3393.5011	-0.55	3	84	4.1e-009	1	U	L.VTDLTKVHKECCHGDLLECADDRADL.A + 2 Gly
4379	234 - 262	858.9053	3431.5920	3431.5908	0.34	4	23	0.005	1	U	L.VTDLTKVHKECCHGDLLECADDRADLAKY.I
4411	234 - 262	899.4177	3593.6415	3593.6436	-0.58	4	14	0.036	1	U	L.VTDLTKVHKECCHGDLLECADDRADLAKY.I + Gl
4461	234 - 262	1252.9063	3755.6969	3755.6965	0.12	4	22	0.007	1	U	L.VTDLTKVHKECCHGDLLECADDRADLAKY.I + 2
4462	234 - 262	1252.9076	3755.7009	3755.6965	1.19	4	27	0.0019	1	U	L.VTDLTKVHKECCHGDLLECADDRADLAKY.I + 2
4337	238 - 262	666.5005	3327.4664	3327.4693	-0.89	3	16	0.027	1	U	L.TKVHKECCHGDLLECADDRADLAKY.I + 2 Glyc
960	251 - 259	532.7191	1063.4237	1063.4240	-0.32	0	23	0.0053	1	U	L.ECADDRADL.A
961	251 - 259	532.7192	1063.4239	1063.4240	-0.092	0	24	0.0041	1	U	L.ECADDRADL.A
962	251 - 259	532.7194	1063.4242	1063.4240	0.13	0	24	0.004	1	U	L.ECADDRADL.A
1826	251 - 262	713.8165	1425.6184	1425.6194	-0.72	1	30	0.001	1	U	L.ECADDRADLAKY.I
1827	251 - 262	713.8165	1425.6185	1425.6194	-0.64	1	15	0.03	1	U	L.ECADDRADLAKY.I
2207	251 - 262	794.8424	1587.6701	1587.6722	-1.32	1	18	0.016	1	U	L.ECADDRADLAKY.I + Glycation (K)
2210	251 - 262	530.2313	1587.6721	1587.6722	-0.069	1	18	0.017	1	U	L.ECADDRADLAKY.I + Glycation (K)
2213	251 - 262	530.2315	1587.6725	1587.6722	0.18	1	16	0.024	1	U	L.ECADDRADLAKY.I + Glycation (K)
1705	263 - 274	697.3331	1392.6517	1392.6555	-2.71	0	67	2.1e-007	1	U	Y.ICDNQDTISSKL.K
1706	263 - 274	697.3342	1392.6538	1392.6555	-1.22	0	54	4.2e-006	1	U	Y.ICDNQDTISSKL.K
1707	263 - 274	697.3342	1392.6539	1392.6555	-1.13	0	62	6.1e-007	1	U	Y.ICDNQDTISSKL.K
1709	263 - 274	697.3345	1392.6545	1392.6555	-0.70	0	61	7.7e-007	1	U	Y.ICDNQDTISSKL.K
1710	263 - 274	697.3345	1392.6545	1392.6555	-0.70	0	26	0.0023	1	U	Y.ICDNQDTISSKL.K
1711	263 - 274	697.3347	1392.6548	1392.6555	-0.52	0	67	2.2e-007	1	U	Y.ICDNQDTISSKL.K
1713	263 - 274	697.3348	1392.6550	1392.6555	-0.34	0	63	5e-007	1	U	Y.ICDNQDTISSKL.K
1714	263 - 274	697.3357	1392.6568	1392.6555	0.96	0	61	8.3e-007	1	U	Y.ICDNQDTISSKL.K
2133	263 - 274	778.3606	1554.7066	1554.7083	-1.07	0	37	0.00018	1	U	Y.ICDNQDTISSKL.K + Glycation (K)
2134	263 - 274	778.3607	1554.7068	1554.7083	-0.99	0	51	8.9e-006	1	U	Y.ICDNQDTISSKL.K + Glycation (K)
2135	263 - 274	778.3607	1554.7069	1554.7083	-0.92	0	45	3e-005	1	U	Y.ICDNQDTISSKL.K + Glycation (K)
2136	263 - 274	778.3608	1554.7070	1554.7083	-0.84	0	51	7.4e-006	1	U	Y.ICDNQDTISSKL.K + Glycation (K)
2137	263 - 274	778.3608	1554.7070	1554.7083	-0.84	0	40	0.0001	1	U	Y.ICDNQDTISSKL.K + Glycation (K)
2138	263 - 274	778.3610	1554.7074	1554.7083	-0.61	0	50	9e-006	1	U	Y.ICDNQDTISSKL.K + Glycation (K)
2139	263 - 274	778.3610	1554.7075	1554.7083	-0.53	0	53	5.4e-006	1	U	Y.ICDNQDTISSKL.K + Glycation (K)
2140	263 - 274	778.3622	1554.7098	1554.7083	0.96	0	38	0.00016	1	U	Y.ICDNQDTISSKL.K + Glycation (K)
1264	319 - 329	606.7983	1211.5820	1211.5822	-0.20	2	24	0.0039	1	U	Y.QEAKDAFLGSF.L
1265	319 - 329	606.7983	1211.5821	1211.5822	-0.097	2	27	0.0021	1	U	Y.QEAKDAFLGSF.L
1266	319 - 329	606.7984	1211.5822	1211.5822	0.0025	2	20	0.0094	1	U	Y.QEAKDAFLGSF.L
1267	319 - 329	606.7984	1211.5822	1211.5822	0.0025	2	20	0.0089	1	U	Y.QEAKDAFLGSF.L
1651	319 - 329	687.8244	1373.6342	1373.6351	-0.60	2	23	0.005	1	U	Y.QEAKDAFLGSF.L + Glycation (K)
1654	319 - 329	687.8245	1373.6344	1373.6351	-0.51	2	24	0.0042	1	U	Y.QEAKDAFLGSF.L + Glycation (K)
1655	319 - 329	687.8245	1373.6344	1373.6351	-0.51	2	26	0.0027	1	U	Y.QEAKDAFLGSF.L + Glycation (K)
1657	319 - 329	687.8246	1373.6346	1373.6351	-0.34	2	23	0.0048	1	U	Y.QEAKDAFLGSF.L + Glycation (K)
1658	319 - 329	687.8246	1373.6346	1373.6351	-0.34	2	31	0.00083	1	U	Y.QEAKDAFLGSF.L + Glycation (K)
1659	319 - 329	687.8247	1373.6347	1373.6351	-0.24	2	27	0.002	1	U	Y.QEAKDAFLGSF.L + Glycation (K)
1540	319 - 330	663.3401	1324.6657	1324.6663	-0.42	3	21	0.0084	1	U	Y.QEAKDAFLGSFL.Y
1969	319 - 331	744.8716	1487.7286	1487.7296	-0.69	4	24	0.0043	1	U	Y.QEAKDAFLGSFLY.E
1970	319 - 331	744.8716	1487.7287	1487.7296	-0.61	4	27	0.0019	1	U	Y.QEAKDAFLGSFLY.E
1971	319 - 331	744.8718	1487.7290	1487.7296	-0.43	4	19	0.014	1	U	Y.QEAKDAFLGSFLY.E
2319	319 - 331	825.8977	1649.7809	1649.7825	-0.96	4	20	0.0099	1	U	Y.QEAKDAFLGSFLY.E + Glycation (K)
2321	319 - 331	825.8978	1649.7811	1649.7825	-0.82	4	21	0.0085	1	U	Y.QEAKDAFLGSFLY.E + Glycation (K)

Query	Start - End	Observed	Mr(expt)	Mr(calc)	ppm	M	Score	Expect	Rank	U	Peptide
2322	319 - 331	825.8979	1649.7812	1649.7825	-0.74	4	22	0.0068	1	U	Y.QEAKDAFLGSFLY.E + Glycation (K)
2323	319 - 331	825.8983	1649.7821	1649.7825	-0.22	4	14	0.037	1	U	Y.QEAKDAFLGSFLY.E + Glycation (K)
569	346 - 352	446.7660	891.5174	891.5178	-0.37	2	26	0.0025	1	U	L.LRLAKEY.E
570	346 - 352	298.1799	891.5178	891.5178	0.024	2	21	0.0073	1	U	L.LRLAKEY.E
937	346 - 352	352.1972	1053.5698	1053.5706	-0.77	2	15	0.033	1	U	L.LRLAKEY.E + Glycation (K)
938	346 - 352	527.7922	1053.5699	1053.5706	-0.63	2	15	0.029	1	U	L.LRLAKEY.E + Glycation (K)
942	346 - 352	352.1974	1053.5704	1053.5706	-0.15	2	15	0.033	1	U	L.LRLAKEY.E + Glycation (K)
943	346 - 352	352.1975	1053.5705	1053.5706	-0.062	2	15	0.029	1	U	L.LRLAKEY.E + Glycation (K)
944	346 - 352	527.7925	1053.5705	1053.5706	-0.056	2	14	0.041	1	U	L.LRLAKEY.E + Glycation (K)
946	346 - 352	527.7926	1053.5706	1053.5706	0.058	2	14	0.041	1	U	L.LRLAKEY.E + Glycation (K)
4069	347 - 369	748.5740	2990.2668	2990.2732	-2.14	3	48	1.8e-005	1	U	L.RLAKEYEATLEECCAKDDPHACY.S + Glycatio
4070	347 - 369	748.5751	2990.2712	2990.2732	-0.67	3	53	5.5e-006	1	U	L.RLAKEYEATLEECCAKDDPHACY.S + Glycatio
4244	347 - 369	789.0865	3152.3171	3152.3260	-2.83	3	14	0.043	1	U	L.RLAKEYEATLEECCAKDDPHACY.S + 2 Glycat
3788	349 - 369	640.7662	2559.0359	2559.0352	0.26	2	27	0.0018	1	U	L.AKEYEATLEECCAKDDPHACY.S
3875	349 - 369	908.0356	2721.0851	2721.0880	-1.07	2	22	0.0062	1	U	L.AKEYEATLEECCAKDDPHACY.S + Glycation (
3876	349 - 369	681.2789	2721.0866	2721.0880	-0.51	2	20	0.011	1	U	L.AKEYEATLEECCAKDDPHACY.S + Glycation (
3877	349 - 369	681.2792	2721.0879	2721.0880	-0.058	2	15	0.029	1	U	L.AKEYEATLEECCAKDDPHACY.S + Glycation (
2966	353 - 369	1034.9049	2067.7953	2067.7972	-0.94	1	64	4.2e-007	1	U	Y.EATLEECCAKDDPHACY.S
2967	353 - 369	1034.9052	2067.7957	2067.7972	-0.71	1	61	7.2e-007	1	U	Y.EATLEECCAKDDPHACY.S
2968	353 - 369	690.2726	2067.7959	2067.7972	-0.63	1	16	0.025	1	U	Y.EATLEECCAKDDPHACY.S
2969	353 - 369	690.2728	2067.7965	2067.7972	-0.35	1	22	0.0058	1	U	Y.EATLEECCAKDDPHACY.S
2970	353 - 369	690.2728	2067.7967	2067.7972	-0.27	1	19	0.012	1	U	Y.EATLEECCAKDDPHACY.S
2971	353 - 369	1034.9059	2067.7972	2067.7972	-0.0015	1	56	2.4e-006	1	U	Y.EATLEECCAKDDPHACY.S
2972	353 - 369	690.2734	2067.7983	2067.7972	0.53	1	27	0.002	1	U	Y.EATLEECCAKDDPHACY.S
2973	353 - 369	1034.9065	2067.7984	2067.7972	0.59	1	54	3.9e-006	1	U	Y.EATLEECCAKDDPHACY.S
2974	353 - 369	690.2736	2067.7989	2067.7972	0.79	1	26	0.0025	1	U	Y.EATLEECCAKDDPHACY.S
3231	353 - 369	1115.9316	2229.8487	2229.8500	-0.59	1	32	0.00063	1	U	Y.EATLEECCAKDDPHACY.S + Glycation (K)
3235	353 - 369	1115.9326	2229.8507	2229.8500	0.29	1	33	0.00051	1	U	Y.EATLEECCAKDDPHACY.S + Glycation (K)
3236	353 - 369	1115.9329	2229.8512	2229.8500	0.51	1	33	0.00054	1	U	Y.EATLEECCAKDDPHACY.S + Glycation (K)
384	370 - 376	405.2237	808.4328	808.4331	-0.31	1	43	4.7e-005	1	U	Y.STVFDKL.K
385	370 - 376	405.2237	808.4329	808.4331	-0.23	1	40	0.0001	1	U	Y.STVFDKL.K
386	370 - 376	405.2238	808.4329	808.4331	-0.16	1	35	0.00035	1	U	Y.STVFDKL.K
387	370 - 376	405.2239	808.4332	808.4331	0.16	1	34	0.00036	1	U	Y.STVFDKL.K
752	370 - 376	486.2502	970.4857	970.4859	-0.16	1	22	0.007	1	U	Y.STVFDKL.K + Glycation (K)
753	370 - 376	486.2502	970.4857	970.4859	-0.16	1	32	0.00065	1	U	Y.STVFDKL.K + Glycation (K)
754	370 - 376	486.2502	970.4859	970.4859	-0.034	1	22	0.0064	1	U	Y.STVFDKL.K + Glycation (K)
755	370 - 376	486.2502	970.4859	970.4859	0.028	1	22	0.0063	1	U	Y.STVFDKL.K + Glycation (K)
756	370 - 376	486.2503	970.4861	970.4859	0.23	1	26	0.0024	1	U	Y.STVFDKL.K + Glycation (K)
757	370 - 376	486.2505	970.4864	970.4859	0.48	1	21	0.0087	1	U	Y.STVFDKL.K + Glycation (K)
758	370 - 376	486.2505	970.4865	970.4859	0.60	1	22	0.0064	1	U	Y.STVFDKL.K + Glycation (K)
3367	370 - 386	1154.0852	2306.1559	2306.1529	1.27	3	13	0.046	1	U	Y.STVFDKLKHLVDEPQNL.I + 2 Glycation (K)
2125	374 - 386	774.9203	1547.8261	1547.8307	-2.96	2	18	0.016	1	U	F.DKLKHLVDEPQNL.I
2127	374 - 386	774.9219	1547.8292	1547.8307	-0.99	2	52	6e-006	1	U	F.DKLKHLVDEPQNL.I
2128	374 - 386	774.9219	1547.8293	1547.8307	-0.91	2	36	0.00023	1	U	F.DKLKHLVDEPQNL.I
2129	374 - 386	516.9506	1547.8300	1547.8307	-0.45	2	17	0.019	1	U	F.DKLKHLVDEPQNL.I
2439	374 - 386	855.9464	1709.8781	1709.8836	-3.17	2	27	0.0021	1	U	F.DKLKHLVDEPQNL.I + Glycation (K)
2441	374 - 386	855.9482	1709.8819	1709.8836	-0.95	2	26	0.0024	1	U	F.DKLKHLVDEPQNL.I + Glycation (K)
2442	374 - 386	855.9485	1709.8824	1709.8836	-0.66	2	33	0.0005	1	U	F.DKLKHLVDEPQNL.I + Glycation (K)
2443	374 - 386	855.9485	1709.8825	1709.8836	-0.59	2	33	0.00046	1	U	F.DKLKHLVDEPQNL.I + Glycation (K)
2445	374 - 386	570.9682	1709.8828	1709.8836	-0.46	2	19	0.012	1	U	F.DKLKHLVDEPQNL.I + Glycation (K)
2446	374 - 386	855.9487	1709.8829	1709.8836	-0.38	2	27	0.0018	1	U	F.DKLKHLVDEPQNL.I + Glycation (K)
2447	374 - 386	570.9684	1709.8835	1709.8836	-0.040	2	16	0.025	1	U	F.DKLKHLVDEPQNL.I + Glycation (K)
2701	374 - 386	936.9739	1871.9332	1871.9364	-1.70	2	26	0.0027	1	U	F.DKLKHLVDEPQNL.I + 2 Glycation (K)
2702	374 - 386	936.9743	1871.9340	1871.9364	-1.25	2	14	0.036	1	U	F.DKLKHLVDEPQNL.I + 2 Glycation (K)
2703	374 - 386	936.9747	1871.9349	1871.9364	-0.79	2	30	0.0011	1	U	F.DKLKHLVDEPQNL.I + 2 Glycation (K)
2704	374 - 386	936.9749	1871.9353	1871.9364	-0.60	2	21	0.0072	1	U	F.DKLKHLVDEPQNL.I + 2 Glycation (K)
2707	374 - 386	936.9751	1871.9356	1871.9364	-0.39	2	24	0.0039	1	U	F.DKLKHLVDEPQNL.I + 2 Glycation (K)
2710	374 - 386	624.9858	1871.9357	1871.9364	-0.37	2	14	0.039	1	U	F.DKLKHLVDEPQNL.I + 2 Glycation (K)
1216	377 - 386	596.8192	1191.6239	1191.6248	-0.76	1	54	3.9e-006	1	U	L.KHLVDEPQNL.I
1217	377 - 386	596.8193	1191.6240	1191.6248	-0.66	1	49	1.3e-005	1	U	L.KHLVDEPQNL.I
1218	377 - 386	398.2153	1191.6242	1191.6248	-0.51	1	67	2e-007	1	U	L.KHLVDEPQNL.I
1219	377 - 386	398.2153	1191.6242	1191.6248	-0.51	1	67	1.9e-007	1	U	L.KHLVDEPQNL.I
1220	377 - 386	398.2154	1191.6243	1191.6248	-0.36	1	54	3.6e-006	1	U	L.KHLVDEPQNL.I
1221	377 - 386	596.8195	1191.6245	1191.6248	-0.24	1	57	1.9e-006	1	U	L.KHLVDEPQNL.I
1222	377 - 386	596.8197	1191.6248	1191.6248	0.065	1	62	5.9e-007	1	U	L.KHLVDEPQNL.I
1223	377 - 386	398.2159	1191.6260	1191.6248	1.02	1	37	0.00019	1	U	L.KHLVDEPQNL.I
1595	377 - 386	677.8453	1353.6761	1353.6776	-1.09	1	20	0.0097	1	U	L.KHLVDEPQNL.I + Glycation (K)
1596	377 - 386	677.8455	1353.6764	1353.6776	-0.91	1	23	0.0054	1	U	L.KHLVDEPQNL.I + Glycation (K)
1597	377 - 386	677.8456	1353.6766	1353.6776	-0.73	1	22	0.0062	1	U	L.KHLVDEPQNL.I + Glycation (K)
1599	377 - 386	452.2329	1353.6769	1353.6776	-0.51	1	15	0.032	1	U	L.KHLVDEPQNL.I + Glycation (K)
1600	377 - 386	452.2330	1353.6772	1353.6776	-0.31	1	31	0.00079	1	U	L.KHLVDEPQNL.I + Glycation (K)
1601	377 - 386	452.2330	1353.6772	1353.6776	-0.31	1	25	0.003	1	U	L.KHLVDEPQNL.I + Glycation (K)
1602	377 - 386	677.8460	1353.6775	1353.6776	-0.095	1	21	0.0078	1	U	L.KHLVDEPQNL.I + Glycation (K)
1603	377 - 386	452.2332	1353.6776	1353.6776	0.018	1	21	0.0078	1	U	L.KHLVDEPQNL.I + Glycation (K)
923	387 - 394	526.7451	1051.4757	1051.4757	0.010	0	23	0.0045	1	U	L.IKQNCDQF.E
924	387 - 394	526.7452	1051.4758	1051.4757	0.12	0	24	0.0037	1	U	L.IKQNCDQF.E
925	387 - 394	526.7452	1051.4758	1051.4757	0.12	0	25	0.0035	1	U	L.IKQNCDQF.E
1283	387 - 394	607.7712	1213.5279	1213.5285	-0.47	0	16	0.026	1	U	L.IKQNCDQF.E + Glycation (K)
1284	387 - 394	607.7714	1213.5283	1213.5285	-0.18	0	23	0.005	1	U	L.IKQNCDQF.E + Glycation (K)
1285	387 - 394	607.7714	1213.5283	1213.5285	-0.18	0	16	0.023	1	U	L.IKQNCDQF.E + Glycation (K)
1286	387 - 394	607.7714	1213.5283	1213.5285	-0.18	0	14	0.037	1	U	L.IKQNCDQF.E + Glycation (K)
2588	387 - 400	886.4189	1770.8232	1770.8247	-0.82	2	32	0.00068	1	U	L.IKQNCDQFEKLGEY.G
2589	387 - 400	886.4189	1770.8232	1770.8247	-0.82	2	43	5e-005	1	U	L.IKQNCDQFEKLGEY.G
2590	387 - 400	591.2819	1770.8239	1770.8247	-0.41	2	28	0.0014	1	U	L.IKQNCDQFEKLGEY.G
2591	387 - 400	591.2820	1770.8243	1770.8247	-0.21	2	25	0.003	1	U	L.IKQNCDQFEKLGEY.G
2592	387 - 400	886.4195	1770.8244	1770.8247	-0.13	2	35	0.00035	1	U	L.IKQNCDQFEKLGEY.G
2593	387 - 400	591.2822	1770.8247	1770.8247	0.012	2	44	4.3e-005	1	U	L.IKQNCDQFEKLGEY.G
2594	387 - 400	886.4197	1770.8248	1770.8247	0.083	2	36	0.00026	1	U	L.IKQNCDQFEKLGEY.G

Query	Start - End	Observed	Mr(expt)	Mr(calc)	ppm	M	Score	Expect	Rank	U	Peptide
2595	387 - 400	591.2822	1770.8249	1770.8247	0.11	2	19	0.012	1	U	L.IKQNCDQFEKLGEY.G
2781	387 - 400	967.4449	1932.8753	1932.8775	-1.11	2	26	0.0028	1	U	L.IKQNCDQFEKLGEY.G + Glycation (K)
2782	387 - 400	967.4450	1932.8755	1932.8775	-1.04	2	34	0.0004	1	U	L.IKQNCDQFEKLGEY.G + Glycation (K)
2783	387 - 400	967.4451	1932.8757	1932.8775	-0.92	2	27	0.0019	1	U	L.IKQNCDQFEKLGEY.G + Glycation (K)
2784	387 - 400	967.4451	1932.8757	1932.8775	-0.92	2	28	0.0017	1	U	L.IKQNCDQFEKLGEY.G + Glycation (K)
2785	387 - 400	967.4452	1932.8758	1932.8775	-0.86	2	28	0.0014	1	U	L.IKQNCDQFEKLGEY.G + Glycation (K)
2786	387 - 400	645.2994	1932.8763	1932.8775	-0.61	2	32	0.00066	1	U	L.IKQNCDQFEKLGEY.G + Glycation (K)
2787	387 - 400	645.2996	1932.8770	1932.8775	-0.24	2	32	0.00065	1	U	L.IKQNCDQFEKLGEY.G + Glycation (K)
2788	387 - 400	645.2998	1932.8776	1932.8775	0.044	2	29	0.0012	1	U	L.IKQNCDQFEKLGEY.G + Glycation (K)
2789	387 - 400	645.3001	1932.8783	1932.8775	0.43	2	28	0.0014	1	U	L.IKQNCDQFEKLGEY.G + Glycation (K)
2790	387 - 400	645.3001	1932.8785	1932.8775	0.53	2	32	0.00065	1	U	L.IKQNCDQFEKLGEY.G + Glycation (K)
2998	387 - 400	699.3168	2094.9285	2094.9303	-0.87	2	17	0.021	1	U	L.IKQNCDQFEKLGEY.G + 2 Glycation (K)
3000	387 - 400	699.3168	2094.9287	2094.9303	-0.79	2	27	0.0022	1	U	L.IKQNCDQFEKLGEY.G + 2 Glycation (K)
3001	387 - 400	699.3170	2094.9291	2094.9303	-0.60	2	17	0.018	1	U	L.IKQNCDQFEKLGEY.G + 2 Glycation (K)
3002	387 - 400	699.3171	2094.9294	2094.9303	-0.43	2	21	0.0072	1	U	L.IKQNCDQFEKLGEY.G + 2 Glycation (K)
1972	411 - 422	496.9464	1487.8175	1487.8195	-1.37	0	18	0.017	1	U	Y.TRKVPQVSTPTL.V + Glycation (K)
340	423 - 429	395.2268	788.4391	788.4392	-0.10	0	29	0.0014	1	U	L.VEVSRSL.G
341	423 - 429	395.2269	788.4392	788.4392	0.049	0	29	0.0014	1	U	L.VEVSRSL.G
342	423 - 429	395.2271	788.4397	788.4392	0.66	0	23	0.0048	1	U	L.VEVSRSL.G
321	457 - 462	387.7161	773.4176	773.4218	-5.45	1	13	0.046	1	U	L.NRLCVL.H
322	457 - 462	387.7177	773.4208	773.4218	-1.26	1	17	0.019	1	U	L.NRLCVL.H
323	457 - 462	387.7180	773.4214	773.4218	-0.54	1	16	0.028	1	U	L.NRLCVL.H
2105	488 - 501	769.8896	1537.7647	1537.7664	-1.09	2	34	0.00036	1	U	F.SALTPDETYVPKAF.D
2106	488 - 501	769.8898	1537.7650	1537.7664	-0.93	2	32	0.00065	1	U	F.SALTPDETYVPKAF.D
2107	488 - 501	769.8898	1537.7650	1537.7664	-0.93	2	36	0.00023	1	U	F.SALTPDETYVPKAF.D
2108	488 - 501	769.8904	1537.7663	1537.7664	-0.059	2	36	0.00025	1	U	F.SALTPDETYVPKAF.D
2413	488 - 501	850.9162	1699.8178	1699.8192	-0.82	2	26	0.0028	1	U	F.SALTPDETYVPKAF.D + Glycation (K)
2414	488 - 501	850.9162	1699.8178	1699.8192	-0.82	2	22	0.0063	1	U	F.SALTPDETYVPKAF.D + Glycation (K)
2415	488 - 501	850.9163	1699.8180	1699.8192	-0.75	2	15	0.028	1	U	F.SALTPDETYVPKAF.D + Glycation (K)
2416	488 - 501	850.9167	1699.8189	1699.8192	-0.17	2	21	0.0079	1	U	F.SALTPDETYVPKAF.D + Glycation (K)
3120	488 - 506	1086.0457	2170.0767	2170.0834	-3.06	4	33	0.00056	1	U	F.SALTPDETYVPKAFDEKLF.T
3121	488 - 506	724.3678	2170.0816	2170.0834	-0.84	4	22	0.0057	1	U	F.SALTPDETYVPKAFDEKLF.T
3122	488 - 506	724.3680	2170.0821	2170.0834	-0.59	4	24	0.0037	1	U	F.SALTPDETYVPKAFDEKLF.T
3123	488 - 506	724.3683	2170.0830	2170.0834	-0.16	4	19	0.011	1	U	F.SALTPDETYVPKAFDEKLF.T
3124	488 - 506	724.3685	2170.0836	2170.0834	0.089	4	29	0.0012	1	U	F.SALTPDETYVPKAFDEKLF.T
3125	488 - 506	1086.0492	2170.0838	2170.0834	0.20	4	36	0.00025	1	U	F.SALTPDETYVPKAFDEKLF.T
3126	488 - 506	724.3692	2170.0858	2170.0834	1.10	4	16	0.023	1	U	F.SALTPDETYVPKAFDEKLF.T
3127	488 - 506	1086.0505	2170.0865	2170.0834	1.45	4	37	0.00018	1	U	F.SALTPDETYVPKAFDEKLF.T
3128	488 - 506	1086.0518	2170.0890	2170.0834	2.57	4	21	0.0081	1	U	F.SALTPDETYVPKAFDEKLF.T
3414	488 - 506	1167.0725	2332.1305	2332.1362	-2.46	4	19	0.014	1	U	F.SALTPDETYVPKAFDEKLF.T + Glycation (K)
3416	488 - 506	1167.0745	2332.1344	2332.1362	-0.79	4	17	0.019	1	U	F.SALTPDETYVPKAFDEKLF.T + Glycation (K)
3421	488 - 506	1167.0759	2332.1373	2332.1362	0.47	4	20	0.0094	1	U	F.SALTPDETYVPKAFDEKLF.T + Glycation (K)
3698	488 - 506	1248.1044	2494.1942	2494.1890	2.06	4	15	0.033	1	U	F.SALTPDETYVPKAFDEKLF.T + 2 Glycation (
1398	491 - 501	634.3132	1266.6119	1266.6132	-1.04	1	40	0.0001	1	U	L.TPDETYVPKAF.D
1399	491 - 501	634.3135	1266.6124	1266.6132	-0.64	1	43	5.4e-005	1	U	L.TPDETYVPKAF.D
1400	491 - 501	634.3138	1266.6131	1266.6132	-0.073	1	42	5.9e-005	1	U	L.TPDETYVPKAF.D
1846	491 - 501	715.3399	1428.6652	1428.6660	-0.56	1	28	0.0018	1	U	L.TPDETYVPKAF.D + Glycation (K)
1847	491 - 501	715.3399	1428.6652	1428.6660	-0.56	1	32	0.00071	1	U	L.TPDETYVPKAF.D + Glycation (K)
2749	491 - 506	633.9840	1898.9302	1898.9302	0.0058	3	31	0.00088	1	U	L.TPDETYVPKAFDEKLF.T
2956	491 - 506	1031.4918	2060.9691	2060.9830	-6.76	3	20	0.01	1	U	L.TPDETYVPKAFDEKLF.T + Glycation (K)
2957	491 - 506	688.0012	2060.9818	2060.9830	-0.57	3	19	0.014	1	U	L.TPDETYVPKAFDEKLF.T + Glycation (K)
2958	491 - 506	688.0021	2060.9846	2060.9830	0.76	3	23	0.0049	1	U	L.TPDETYVPKAFDEKLF.T + Glycation (K)
2959	491 - 506	1031.5013	2060.9881	2060.9830	2.48	3	25	0.0029	1	U	L.TPDETYVPKAFDEKLF.T + Glycation (K)
247	497 - 501	362.1996	722.3847	722.3851	-0.51	0	13	0.045	1	U	Y.VPKAF.D + Glycation (K)
248	497 - 501	362.1997	722.3847	722.3851	-0.43	0	19	0.013	1	U	Y.VPKAF.D + Glycation (K)
249	497 - 501	362.1997	722.3849	722.3851	-0.26	0	19	0.013	1	U	Y.VPKAF.D + Glycation (K)
1227	497 - 506	597.3314	1192.6483	1192.6492	-0.76	2	31	0.00078	1	U	Y.VPKAFDEKLF.T
1228	497 - 506	398.5567	1192.6484	1192.6492	-0.70	2	20	0.01	1	U	Y.VPKAFDEKLF.T
1229	497 - 506	597.3316	1192.6486	1192.6492	-0.46	2	31	0.00089	1	U	Y.VPKAFDEKLF.T
1230	497 - 506	597.3317	1192.6489	1192.6492	-0.24	2	33	0.00048	1	U	Y.VPKAFDEKLF.T
1231	497 - 506	398.5569	1192.6490	1192.6492	-0.15	2	30	0.001	1	U	Y.VPKAFDEKLF.T
1232	497 - 506	597.3318	1192.6490	1192.6492	-0.14	2	46	2.6e-005	1	U	Y.VPKAFDEKLF.T
1233	497 - 506	398.5570	1192.6491	1192.6492	-0.074	2	31	0.00085	1	U	Y.VPKAFDEKLF.T
1234	497 - 506	398.5570	1192.6492	1192.6492	0.0017	2	20	0.0098	1	U	Y.VPKAFDEKLF.T
1235	497 - 506	398.5570	1192.6492	1192.6492	0.0017	2	24	0.0036	1	U	Y.VPKAFDEKLF.T
1606	497 - 506	678.3576	1354.7006	1354.7020	-1.02	2	23	0.0054	1	U	Y.VPKAFDEKLF.T + Glycation (K)
1607	497 - 506	678.3577	1354.7009	1354.7020	-0.83	2	22	0.0065	1	U	Y.VPKAFDEKLF.T + Glycation (K)
1608	497 - 506	678.3579	1354.7013	1354.7020	-0.56	2	23	0.0056	1	U	Y.VPKAFDEKLF.T + Glycation (K)
1609	497 - 506	452.5744	1354.7014	1354.7020	-0.48	2	24	0.004	1	U	Y.VPKAFDEKLF.T + Glycation (K)
1610	497 - 506	678.3581	1354.7016	1354.7020	-0.29	2	21	0.0084	1	U	Y.VPKAFDEKLF.T + Glycation (K)
1611	497 - 506	452.5745	1354.7016	1354.7020	-0.28	2	28	0.0014	1	U	Y.VPKAFDEKLF.T + Glycation (K)
1612	497 - 506	452.5746	1354.7019	1354.7020	-0.084	2	24	0.0041	1	U	Y.VPKAFDEKLF.T + Glycation (K)
1613	497 - 506	452.5747	1354.7021	1354.7020	0.071	2	23	0.0054	1	U	Y.VPKAFDEKLF.T + Glycation (K)
2058	497 - 506	506.5922	1516.7547	1516.7549	-0.073	2	20	0.011	1	U	Y.VPKAFDEKLF.T + 2 Glycation (K)
108	502 - 506	326.1707	650.3268	650.3275	-1.11	1	14	0.039	1	U	F.DEKLF.T
109	502 - 506	326.1710	650.3275	650.3275	-0.065	1	17	0.02	1	U	F.DEKLF.T
111	502 - 506	326.1710	650.3275	650.3275	0.028	1	15	0.035	1	U	F.DEKLF.T
392	502 - 506	407.1973	812.3800	812.3804	-0.45	1	14	0.039	1	U	F.DEKLF.T + Glycation (K)
394	502 - 506	407.1975	812.3803	812.3804	-0.0098	1	15	0.035	1	U	F.DEKLF.T + Glycation (K)
3979	507 - 528	721.3635	2881.4247	2881.4267	-0.67	1	14	0.041	1	U	F.TFHADICTLPDTEKQIKKQTAL.V + 2 Glycati
3980	507 - 528	721.3646	2881.4291	2881.4267	0.86	1	17	0.019	1	U	F.TFHADICTLPDTEKQIKKQTAL.V + 2 Glycati
4359	507 - 531	847.1769	3384.6787	3384.6746	1.21	2	18	0.014	1	U	F.TFHADICTLPDTEKQIKKQTALVEL.L + 3 Glyc
4254	507 - 532	794.4221	3173.6591	3173.6530	1.93	3	24	0.0038	1	U	F.TFHADICTLPDTEKQIKKQTALVELL.K + Glyca
4255	507 - 532	794.4224	3173.6603	3173.6530	2.32	3	34	0.00038	1	U	F.TFHADICTLPDTEKQIKKQTALVELL.K + Glyca
4342	507 - 532	668.1420	3335.6738	3335.7058	-9.60	3	26	0.0025	1	U	F.TFHADICTLPDTEKQIKKQTALVELL.K + 2 Gly
4343	507 - 532	1112.9048	3335.6925	3335.7058	-3.97	3	21	0.0078	1	U	F.TFHADICTLPDTEKQIKKQTALVELL.K + 2 Gly
4344	507 - 532	834.9332	3335.7038	3335.7058	-0.61	3	38	0.00015	1	U	F.TFHADICTLPDTEKQIKKQTALVELL.K + 2 Gly
4345	507 - 532	834.9337	3335.7056	3335.7058	-0.054	3	49	1.2e-005	1	U	F.TFHADICTLPDTEKQIKKQTALVELL.K + 2 Gly

Query	Start – End	Observed	Mr(expt)	Mr(calc)	ppm	M	Score	Expect	Rank	U	Peptide
4346	507 – 532	834.9338	3335.7059	3335.7058	0.042	3	37	0.00018	1	U	F.TFHADICTLPDTEKQIKKQTALVELL.K + 2 Gly
4347	507 – 532	834.9340	3335.7069	3335.7058	0.32	3	37	0.00018	1	U	F.TFHADICTLPDTEKQIKKQTALVELL.K + 2 Gly
4348	507 – 532	668.1491	3335.7092	3335.7058	1.01	3	31	0.00085	1	U	F.TFHADICTLPDTEKQIKKQTALVELL.K + 2 Gly
4349	507 – 532	834.9346	3335.7093	3335.7058	1.04	3	36	0.00023	1	U	F.TFHADICTLPDTEKQIKKQTALVELL.K + 2 Gly
4351	507 – 532	1112.9112	3335.7117	3335.7058	1.76	3	32	0.00065	1	U	F.TFHADICTLPDTEKQIKKQTALVELL.K + 2 Gly
4352	507 – 532	1112.9113	3335.7121	3335.7058	1.90	3	25	0.0029	1	U	F.TFHADICTLPDTEKQIKKQTALVELL.K + 2 Gly
4354	507 – 532	834.9367	3335.7175	3335.7058	3.52	3	46	2.5e-005	1	U	F.TFHADICTLPDTEKQIKKQTALVELL.K + 2 Gly
4385	507 – 532	875.4471	3497.7592	3497.7586	0.17	3	31	0.00076	1	U	F.TFHADICTLPDTEKQIKKQTALVELL.K + 3 Gly
4386	507 – 532	875.4482	3497.7636	3497.7586	1.43	3	33	0.00045	1	U	F.TFHADICTLPDTEKQIKKQTALVELL.K + 3 Gly
4388	507 – 532	875.4484	3497.7644	3497.7586	1.65	3	46	2.4e-005	1	U	F.TFHADICTLPDTEKQIKKQTALVELL.K + 3 Gly
4389	507 – 532	875.4485	3497.7651	3497.7586	1.85	3	50	9.1e-006	1	U	F.TFHADICTLPDTEKQIKKQTALVELL.K + 3 Gly
4392	507 – 532	1166.9295	3497.7666	3497.7586	2.27	3	33	0.00053	1	U	F.TFHADICTLPDTEKQIKKQTALVELL.K + 3 Gly
3654	509 – 528	824.7581	2471.2525	2471.2577	-2.11	0	51	8.6e-006	1	U	F.HADICTLPDTEKQIKKQTAL.V + Glycation (K
3655	509 – 528	824.7589	2471.2549	2471.2577	-1.15	0	50	1e-005	1	U	F.HADICTLPDTEKQIKKQTAL.V + Glycation (K
3656	509 – 528	618.8212	2471.2556	2471.2577	-0.87	0	34	0.00041	1	U	F.HADICTLPDTEKQIKKQTAL.V + Glycation (K
3657	509 – 528	618.8214	2471.2563	2471.2577	-0.58	0	27	0.002	1	U	F.HADICTLPDTEKQIKKQTAL.V + Glycation (K
3658	509 – 528	824.7599	2471.2578	2471.2577	0.043	0	20	0.009	1	U	F.HADICTLPDTEKQIKKQTAL.V + Glycation (K
3659	509 – 528	618.8221	2471.2595	2471.2577	0.70	0	31	0.00087	1	U	F.HADICTLPDTEKQIKKQTAL.V + Glycation (K
3829	509 – 528	878.7754	2633.3045	2633.3106	-2.29	0	61	8.9e-007	1	U	F.HADICTLPDTEKQIKKQTAL.V + 2 Glycation
3830	509 – 528	878.7761	2633.3065	2633.3106	-1.53	0	54	4.4e-006	1	U	F.HADICTLPDTEKQIKKQTAL.V + 2 Glycation
3831	509 – 528	878.7767	2633.3082	2633.3106	-0.90	0	49	1.2e-005	1	U	F.HADICTLPDTEKQIKKQTAL.V + 2 Glycation
3832	509 – 528	659.3346	2633.3093	2633.3106	-0.50	0	21	0.0079	1	U	F.HADICTLPDTEKQIKKQTAL.V + 2 Glycation
3833	509 – 528	659.3348	2633.3103	2633.3106	-0.12	0	21	0.0077	1	U	F.HADICTLPDTEKQIKKQTAL.V + 2 Glycation
3834	509 – 528	659.3354	2633.3125	2633.3106	0.72	0	21	0.0074	1	U	F.HADICTLPDTEKQIKKQTAL.V + 2 Glycation
3917	509 – 528	699.8481	2795.3635	2795.3634	0.024	0	14	0.04	1	U	F.HADICTLPDTEKQIKKQTAL.V + 3 Glycation
3921	509 – 531	938.4923	2812.4551	2812.4528	0.82	1	16	0.027	1	U	F.HADICTLPDTEKQIKKQTALVEL.L + Glycation
4047	509 – 531	992.5065	2974.4976	2974.5056	-2.71	1	43	4.7e-005	1	U	F.HADICTLPDTEKQIKKQTALVEL.L + 2 Glycat
4048	509 – 531	744.6323	2974.5002	2974.5056	-1.83	1	18	0.017	1	U	F.HADICTLPDTEKQIKKQTALVEL.L + 2 Glycat
4049	509 – 531	744.6328	2974.5021	2974.5056	-1.17	1	17	0.018	1	U	F.HADICTLPDTEKQIKKQTALVEL.L + 2 Glycat
4011	509 – 532	976.1851	2925.5334	2925.5369	-1.20	2	26	0.0026	1	U	F.HADICTLPDTEKQIKKQTALVELL.K + Glycati
4012	509 – 532	732.3908	2925.5339	2925.5369	-1.01	2	57	2.1e-006	1	U	F.HADICTLPDTEKQIKKQTALVELL.K + Glycati
4013	509 – 532	732.3910	2925.5347	2925.5369	-0.73	2	54	4.2e-006	1	U	F.HADICTLPDTEKQIKKQTALVELL.K + Glycati
4014	509 – 532	976.1863	2925.5372	2925.5369	0.11	2	32	0.00069	1	U	F.HADICTLPDTEKQIKKQTALVELL.K + Glycati
4015	509 – 532	732.3917	2925.5378	2925.5369	0.31	2	47	2e-005	1	U	F.HADICTLPDTEKQIKKQTALVELL.K + Glycati
4016	509 – 532	976.1866	2925.5381	2925.5369	0.43	2	48	1.6e-005	1	U	F.HADICTLPDTEKQIKKQTALVELL.K + Glycati
4017	509 – 532	732.3919	2925.5387	2925.5369	0.61	2	36	0.00024	1	U	F.HADICTLPDTEKQIKKQTALVELL.K + Glycati
4163	509 – 532	1030.2014	3087.5824	3087.5897	-2.35	2	54	4.2e-006	1	U	F.HADICTLPDTEKQIKKQTALVELL.K + 2 Glyca
4164	509 – 532	772.9037	3087.5859	3087.5897	-1.23	2	48	1.6e-005	1	U	F.HADICTLPDTEKQIKKQTALVELL.K + 2 Glyca
4165	509 – 532	1030.2031	3087.5876	3087.5897	-0.69	2	60	9.3e-007	1	U	F.HADICTLPDTEKQIKKQTALVELL.K + 2 Glyca
4166	509 – 532	772.9042	3087.5876	3087.5897	-0.69	2	37	0.0002	1	U	F.HADICTLPDTEKQIKKQTALVELL.K + 2 Glyca
4167	509 – 532	772.9043	3087.5881	3087.5897	-0.52	2	60	1e-006	1	U	F.HADICTLPDTEKQIKKQTALVELL.K + 2 Glyca
4168	509 – 532	1030.2034	3087.5883	3087.5897	-0.46	2	47	2e-005	1	U	F.HADICTLPDTEKQIKKQTALVELL.K + 2 Glyca
4169	509 – 532	772.9044	3087.5886	3087.5897	-0.36	2	69	1.3e-007	1	U	F.HADICTLPDTEKQIKKQTALVELL.K + 2 Glyca
4170	509 – 532	1030.2038	3087.5895	3087.5897	-0.077	2	25	0.003	1	U	F.HADICTLPDTEKQIKKQTALVELL.K + 2 Glyca
4171	509 – 532	772.9047	3087.5898	3087.5897	0.040	2	48	1.6e-005	1	U	F.HADICTLPDTEKQIKKQTALVELL.K + 2 Glyca
4172	509 – 532	1030.2042	3087.5908	3087.5897	0.37	2	50	1e-005	1	U	F.HADICTLPDTEKQIKKQTALVELL.K + 2 Glyca
4307	509 – 532	813.4174	3249.6403	3249.6425	-0.67	2	19	0.012	1	U	F.HADICTLPDTEKQIKKQTALVELL.K + 3 Glyca
4308	509 – 532	813.4175	3249.6408	3249.6425	-0.52	2	40	9.5e-005	1	U	F.HADICTLPDTEKQIKKQTALVELL.K + 3 Glyca
4309	509 – 532	813.4182	3249.6437	3249.6425	0.38	2	30	0.0011	1	U	F.HADICTLPDTEKQIKKQTALVELL.K + 3 Glyca
4310	509 – 532	1084.2225	3249.6456	3249.6425	0.96	2	49	1.3e-005	1	U	F.HADICTLPDTEKQIKKQTALVELL.K + 3 Glyca
4311	509 – 532	813.4191	3249.6472	3249.6425	1.43	2	67	1.8e-007	1	U	F.HADICTLPDTEKQIKKQTALVELL.K + 3 Glyca
509	544 – 550	434.7148	867.4151	867.4160	-1.11	0	15	0.033	1	U	L.KTVMENF.V
1584	544 – 553	674.3285	1346.6424	1346.6428	-0.28	1	45	3.3e-005	1	U	L.KTVMENFVAF.V + Glycation (K)
2888	551 – 567	1003.4251	2004.8357	2004.8380	-1.15	1	67	1.9e-007	1	U	F.VAFVDKCCAADDKEACF.A
2889	551 – 567	1003.4256	2004.8366	2004.8380	-0.67	1	67	1.9e-007	1	U	F.VAFVDKCCAADDKEACF.A
2890	551 – 567	669.2863	2004.8369	2004.8380	-0.53	1	47	1.9e-005	1	U	F.VAFVDKCCAADDKEACF.A
2891	551 – 567	669.2865	2004.8377	2004.8380	-0.15	1	43	5.1e-005	1	U	F.VAFVDKCCAADDKEACF.A
2892	551 – 567	669.2865	2004.8377	2004.8380	-0.15	1	33	0.0005	1	U	F.VAFVDKCCAADDKEACF.A
3113	551 – 567	723.3036	2166.8889	2166.8908	-0.86	1	26	0.0026	1	U	F.VAFVDKCCAADDKEACF.A + Glycation (K)
3114	551 – 567	723.3038	2166.8895	2166.8908	-0.61	1	34	0.00036	1	U	F.VAFVDKCCAADDKEACF.A + Glycation (K)
3115	551 – 567	1084.4522	2166.8897	2166.8908	-0.49	1	37	0.00019	1	U	F.VAFVDKCCAADDKEACF.A + Glycation (K)
3116	551 – 567	1084.4523	2166.8900	2166.8908	-0.38	1	42	6.4e-005	1	U	F.VAFVDKCCAADDKEACF.A + Glycation (K)
3117	551 – 567	723.3040	2166.8902	2166.8908	-0.27	1	32	0.00064	1	U	F.VAFVDKCCAADDKEACF.A + Glycation (K)
3394	551 – 567	777.3200	2328.9382	2328.9436	-2.33	1	18	0.017	1	U	F.VAFVDKCCAADDKEACF.A + 2 Glycation (K)
3395	551 – 567	777.3215	2328.9428	2328.9436	-0.37	1	22	0.0069	1	U	F.VAFVDKCCAADDKEACF.A + 2 Glycation (K)
2391	554 – 567	844.8383	1687.6620	1687.6640	-1.22	0	50	1.1e-005	1	U	F.VDKCCAADDKEACF.A
2392	554 – 567	844.8383	1687.6621	1687.6640	-1.15	0	40	9.9e-005	1	U	F.VDKCCAADDKEACF.A
2393	554 – 567	844.8387	1687.6628	1687.6640	-0.73	0	40	0.0001	1	U	F.VDKCCAADDKEACF.A
2394	554 – 567	563.5616	1687.6629	1687.6640	-0.66	0	36	0.00024	1	U	F.VDKCCAADDKEACF.A
2395	554 – 567	844.8388	1687.6629	1687.6640	-0.64	0	49	1.2e-005	1	U	F.VDKCCAADDKEACF.A
2397	554 – 567	563.5619	1687.6638	1687.6640	-0.11	0	14	0.037	1	U	F.VDKCCAADDKEACF.A
2398	554 – 567	563.5619	1687.6638	1687.6640	-0.11	0	20	0.011	1	U	F.VDKCCAADDKEACF.A
2399	554 – 567	563.5621	1687.6644	1687.6640	0.21	0	25	0.0029	1	U	F.VDKCCAADDKEACF.A
2400	554 – 567	563.5622	1687.6647	1687.6640	0.42	0	36	0.00025	1	U	F.VDKCCAADDKEACF.A
2669	554 – 567	925.8646	1849.7146	1849.7169	-1.24	0	32	0.00056	1	U	F.VDKCCAADDKEACF.A + Glycation (K)
2670	554 – 567	925.8646	1849.7146	1849.7169	-1.24	0	43	5.5e-005	1	U	F.VDKCCAADDKEACF.A + Glycation (K)
2671	554 – 567	925.8646	1849.7147	1849.7169	-1.17	0	31	0.00087	1	U	F.VDKCCAADDKEACF.A + Glycation (K)
2672	554 – 567	925.8646	1849.7147	1849.7169	-1.17	0	39	0.00013	1	U	F.VDKCCAADDKEACF.A + Glycation (K)
2673	554 – 567	617.5790	1849.7151	1849.7169	-0.94	0	25	0.0032	1	U	F.VDKCCAADDKEACF.A + Glycation (K)
2674	554 – 567	617.5792	1849.7158	1849.7169	-0.55	0	45	3.2e-005	1	U	F.VDKCCAADDKEACF.A + Glycation (K)
2675	554 – 567	617.5793	1849.7162	1849.7169	-0.34	0	55	3e-006	1	U	F.VDKCCAADDKEACF.A + Glycation (K)
2676	554 – 567	617.5794	1849.7164	1849.7169	-0.25	0	23	0.0045	1	U	F.VDKCCAADDKEACF.A + Glycation (K)
2677	554 – 567	617.5795	1849.7168	1849.7169	-0.052	0	24	0.004	1	U	F.VDKCCAADDKEACF.A + Glycation (K)
2678	554 – 567	617.5801	1849.7186	1849.7169	0.94	0	44	4.3e-005	1	U	F.VDKCCAADDKEACF.A + Glycation (K)
224	568 – 574	357.2131	712.4116	712.4119	-0.47	0	20	0.0097	1	U	F.AVEGPKL.V
2046	568 – 582	756.9343	1511.8540	1511.8559	-1.27	1	25	0.003	1	U	F.AVEGPKLVVSTQTAL.A
2366	568 – 582	837.9600	1673.9055	1673.9087	-1.94	1	14	0.038	1	U	F.AVEGPKLVVSTQTAL.A + Glycation (K)

Query	Start - End	Observed	Mr(expt)	Mr(calc)	ppm	M	Score	Expect	Rank	U	Peptide
2367	568 - 582	837.9609	1673.9072	1673.9087	-0.91	1	25	0.0032	1	U	F.AVEGPKLVVSTQTAL.A + Glycation (K)
2196	568 - 583	792.4531	1582.8917	1582.8930	-0.83	2	34	0.0004	1	U	F.AVEGPKLVVSTQTALA.-
2197	568 - 583	792.4533	1582.8919	1582.8930	-0.68	2	35	0.00032	1	U	F.AVEGPKLVVSTQTALA.-
2198	568 - 583	792.4534	1582.8922	1582.8930	-0.53	2	31	0.00084	1	U	F.AVEGPKLVVSTQTALA.-
2524	568 - 583	873.4792	1744.9438	1744.9458	-1.16	2	46	2.3e-005	1	U	F.AVEGPKLVVSTQTALA.- + Glycation (K)
2525	568 - 583	873.4792	1744.9439	1744.9458	-1.09	2	16	0.026	1	U	F.AVEGPKLVVSTQTALA.- + Glycation (K)
2526	568 - 583	873.4793	1744.9441	1744.9458	-1.02	2	41	8.5e-005	1	U	F.AVEGPKLVVSTQTALA.- + Glycation (K)
2527	568 - 583	873.4796	1744.9447	1744.9458	-0.68	2	18	0.015	1	U	F.AVEGPKLVVSTQTALA.- + Glycation (K)
2528	568 - 583	873.4799	1744.9452	1744.9458	-0.39	2	37	0.00022	1	U	F.AVEGPKLVVSTQTALA.- + Glycation (K)
2531	568 - 583	873.4813	1744.9481	1744.9458	1.28	2	32	0.00068	1	U	F.AVEGPKLVVSTQTALA.- + Glycation (K)

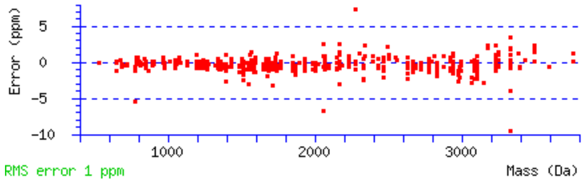

RMS error 1 ppm

Mascot: http://www.matrixscience.com/
