# Supplementary figures and images for: Advanced Glycation End Products of Bovine Serum Albumin Suppressed Th1/Th2 Cytokine but Enhanced Monocyte IL-6 Gene Expression via MAPK-ERK and MyD88 Transduced NF-κB p50 Signaling Pathways
Source: Molecules. 2019 Jul 4;24(13):2461. doi: 10.3390/molecules24132461 (PMC6652144; doi:10.3390/molecules24132461)

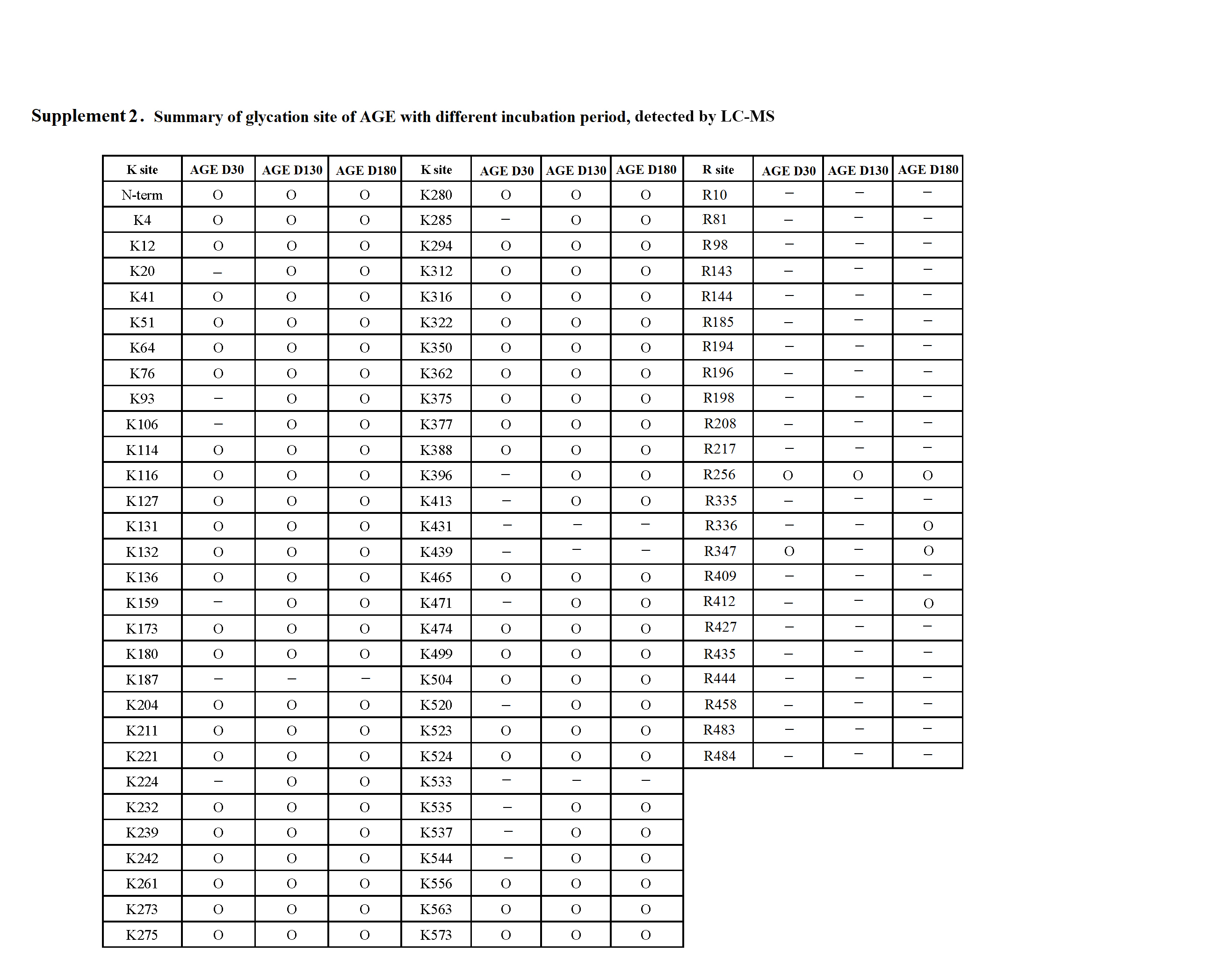

Supplement: Supplementary file 1 [file molecules-24-02461-s001.zip › Supplement 1 copy.jpg]

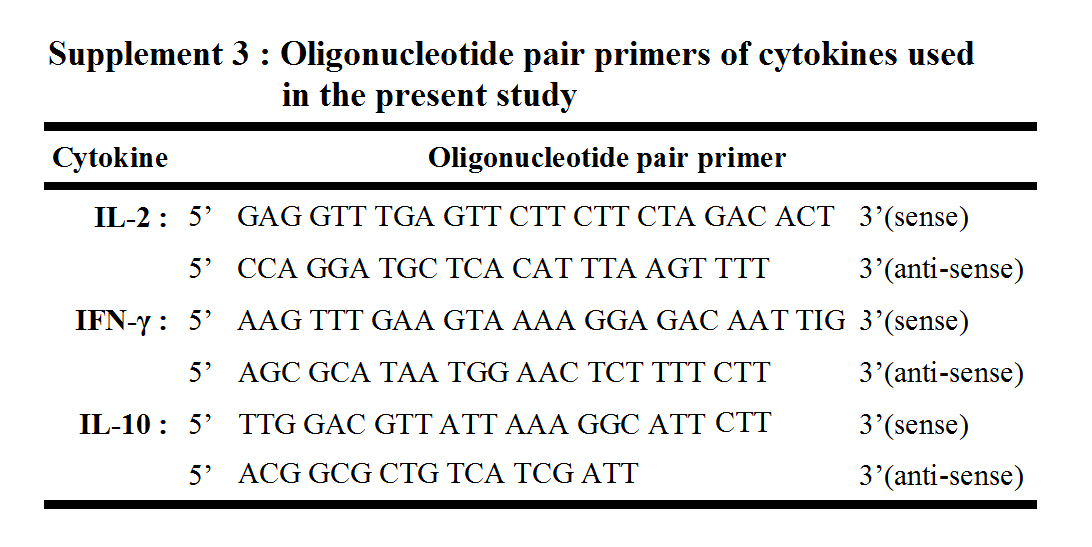

Supplement: Supplementary file 1 [file molecules-24-02461-s001.zip › Supplement 3 copy.jpg]
